# Generation of non-stabilized alkyl radicals from thianthrenium salts for C–B and C–C bond formation

**DOI:** 10.1038/s41467-021-24716-2

**Published:** 2021-07-26

**Authors:** Cheng Chen, Zheng-Jun Wang, Hongjian Lu, Yue Zhao, Zhuangzhi Shi

**Affiliations:** 1 State Key Laboratory of Coordination Chemistry, Chemistry and Biomedicine Innovation Center (ChemBIC), School of Chemistry and Chemical Engineering, Nanjing University, Nanjing, China; 2 School of Chemistry and Chemical Engineering, Henan Normal University, Xinxiang, Henan China

**Keywords:** Diversity-oriented synthesis, Synthetic chemistry methodology, Photocatalysis

## Abstract

Sulfonium salts bearing a positively charged sulfur atom with three organic substituents have intrigued chemists for more than a century for their unusual structures and high chemical reactivity. These compounds are known to undergo facile single-electron reduction to emerge as a valuable and alternative source of aryl radicals for organic synthesis. However, the generation of non-stabilized alkyl radicals from sulfonium salts has been a challenge for several decades. Here we report the treatment of *S*-(alkyl) thianthrenium salts to generate non-stabilized alkyl radicals as key intermediates granting the controlled and selective outcome of the ensuing reactions under mild photoredox conditions. The value of these reagents has been demonstrated through the efficient construction of alkylboronates and other transformations, including heteroarylation, alkylation, alkenylation, and alkynylation. The developed method is practical, and provides the opportunity to convert C–OH bond to C–B and C–C bonds.

## Abstract (web summary)

The generation of non-stabilized alkyl radicals from sulfonium salts has been a challenge for several decades. Here, the authors show the treatment of S-(alkyl) thianthrenium salts, which can generate non-stabilized alkyl radicals as key intermediates that enable controlled and selective reactions under mild photoredox conditions.

## Introduction

**Fig. 1:**
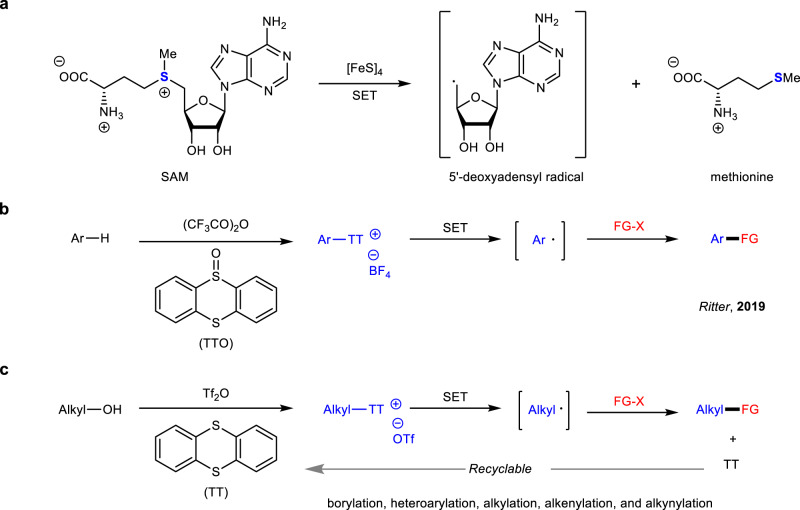
Generation of radicals from sulfonium salts. **a** Radical SAM cleavage in biosynthesis. **b** Generation of aryl radicals from *S*-(aryl) thianthrenium salts. **c** Generation of non-stabilized alkyl radicals from *S*-(alkyl) thianthrenium salts.

Sulfonium salts are among the most versatile of all reactive intermediates in organic chemistry^1–3^. In general, the reactivity of sulfonium salts is determined by the positive charge that they contain, which is mainly located at the sulfur atom. In this context, many sulfonium salt-based reagents including Umemoto’s reagent have been designed for electrophilic substitution^4–10^. In nature, enzymes catalyzing the generation of alkyl radicals from sulfonium salts known to date all belong to the radical *S*-adenosylmethionine (SAM) superfamily (Fig. 1a)^11–15^. In a synthetic chemistry setting, general methods that utilize sulfonium salts as radical precursors have been investigated over the past decades^16–18^. Recently, the emerging field of photoredox catalysis has also offered new possibilities for these single electron transfer (SET)-induced transformations under mild conditions^19–21^. Ritter and coworkers just reported the site-selective C–H functionalization of arenes to build *S*-(aryl) thianthrenium salts, which could be utilized in radical-involved transformations by photoredox catalysis (Fig. 1b)^22–26^. The Procter group also explored a one-pot strategy for the facile construction of (hetero)biaryl motifs with intermediate *S*-(aryl) dibenzothiophenium salts enabled by organic photoredox catalysts^27^. Despite these advances, the vast majority of the sulfonium salts that undergo SET process have been limited to form aryl radicals; The generation of alkyl radicals, especially the non-stabilized ones from sulfonium salts is synthetically challenging.

As precursors for *S*-ylides, *S*-(alkyl) sulfonium salts have been widely applied in cyclization^28,29^ and rearrangement reactions^30^. The early studies from the Kellogg group found the reduction of *S*-(alkyl) sulfonium salts under photoredox catalytic conditions^31,32^. However, the reported protocols have been limited for the generation of stabilized alkyl radicals (benzyl or with α-electron-withdrawing groups) so far^33–37^. To mimic the radical-involved biosynthesis from SAM, here we show *S*-(alkyl) thianthrenium salts, which are ready to engage in diverse SET-induced fragmentations and transformations, especially by mild photoredox processes (Fig. 1c). Moreover, the formed thianthrene (TT) reagent could be recycled after the reactions. A significant observation of the reactivity of these compounds is that diverse non-stabilized alkyl radicals can be effectively generated as key intermediates in these transformations.

## Results

### Synthesis of S-(alkyl) thianthrenium salts

**Fig. 2:**
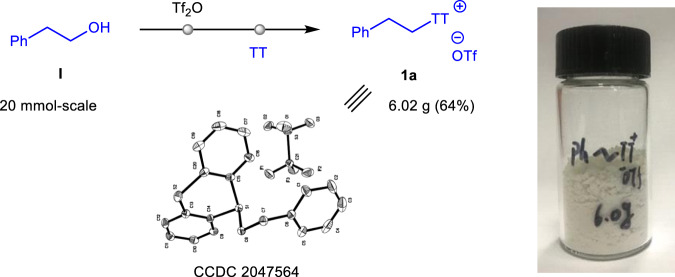
General procedures to access *S*-(alkyl) thianthrenium salts. Synthesis of *S*-(alkyl) thianthrenium salt **1a** from the related alcohol **I**.

Instead of the known methods to access *S*-(alkyl) thianthrenium salts by using alkyl-mercurial and alkyl-tin reagents in the 1990s^38,39^, they were easily prepared by an alternative method from the corresponding alcohols and TT in one-pot on the gram scale (for an example of **1a**, Fig. 2). A crystal of the substrate **1a** was generated and subjected to X-ray crystallographic analysis (see Supplementary Data 1).

### Reaction design

**Fig. 3:**
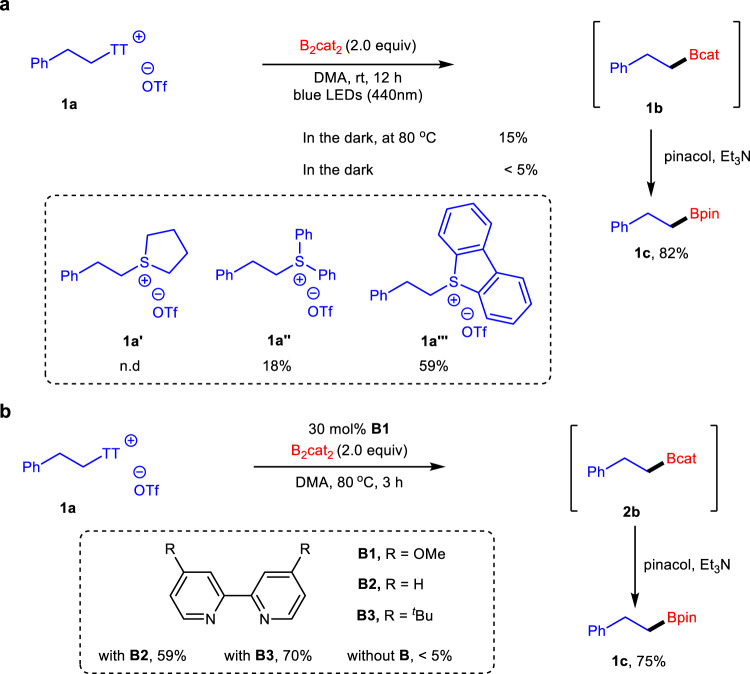
Desulfurative borylation of thianthrenium salt 1a. **a** Photoinduced desulfurative borylation. **b** Thermoinduced desulfurative borylation by Lewis base.

Alkylboronates are valuable building blocks in organic synthesis and can be transformed into various functional groups^40–45^. Traditional methods to prepare them include electrophilic borylation of organolithium or Grignard reagents and hydroboration of olefins^46^. The recent state-of-the-art synthesis of these compounds has evolved to utilize bench-stable alkyl radical precursors, especially redox-active alkyl halides^47–58^, *N*-hydroxyphthalimide (NHPI) esters^59–61^, Katritzky salts^62–65^, xanthates/oxalates^66^, thionocarbonates^67^, and even alkanes^68^. Given the importance of alkylboronic esters, we explored the desulfurative borylation of thianthrenium salts under photo- and thermoinduced conditions (Fig. 3). Treatment of **1a** with 2.0 equiv of bis(catecholato)diboron (B_2_cat_2_) in DMA under irradiation with a blue LED gave the best result of intermediate **1b**, which can be further converted into a stable boronic ester **1c** in 82% total yield with pinacol and NEt_3_. Rigorous control experiments demonstrated that light irradiation greatly facilitated borylation. Other sulfonium triflates such as **1a’** derived from tetrahydrothiophene completely failed, and borylation of diphenyl sulfonium salt **1a”** generated PhBpin as a major product. Dibenzothiophenium salt **1a”‘** was also a reliable alkyl radical precursor for this transformation, albeit with a lower reactivity (for details of optimization studies, see Supplementary Table 1). Our group recently developed the Lewis base-promoted deaminative borylation reaction of Katritzky salts^65^. When the reaction mixture was heated at 80 °C in the presence of 4,4’-dimethoxy-2,2’-bipyridine (**B1**), the desired product was also formed in a 75% yield. Other Lewis bases such as 2,2’-bipyridine (**B2**) and 4,4’-di-*tert*-butyl-2,2’-bipyridine (**B3**) exhibited lower reactivity, and the reaction in the absence of the Lewis base gave more than trace amounts of **1c** (for details of optimization studies, see Supplementary Table 2). Such thermoinduced conditions provided an alternative approach for this transformation.

### Scope of the methodology

**Fig. 4:**
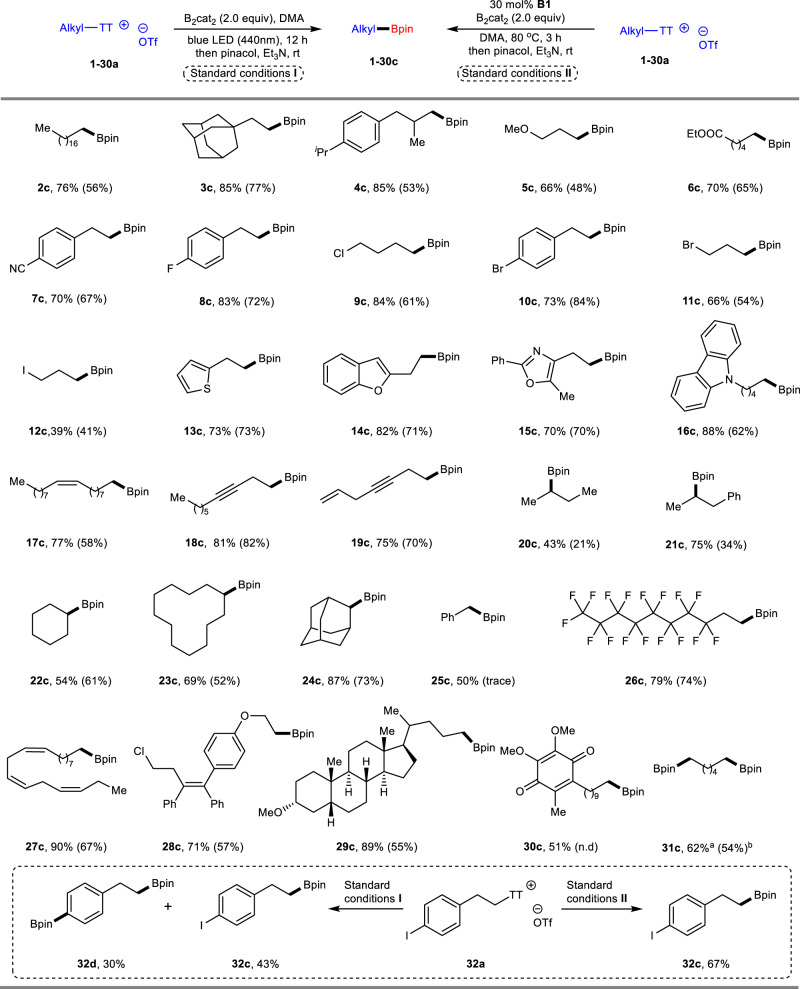
Substrate scope of *S*-(alkyl) thianthrenium salts under photo- and thermo-induced conditions. Standard conditions **I**: thianthrenium salts **1–32a** (0.40 mmol), B_2_cat_2_ (0.80 mmol) in 1.0 mL DMA, irradiation from a 40 W Kessil blue LED bulb (440 nm) for 12 h under Ar; then pinacol (1.60 mmol) in 1.0 mL Et_3_N was added to the mixture, 1 h, isolated yields; Standard conditions II: thianthrenium salts **1–32a** (0.40 mmol), B_2_cat_2_ (0.80 mmol), 30 mol% of **B1** in DMA (1.0 mL), 80 °C, 3 h, under Ar, isolated yields; The yields in standard conditions II were afforded in parentheses. ^a^B_2_cat_2_ (1.60 mmol) in DMA (1.5 mL). ^b^B_2_cat_2_ (1.60 mmol), 60 mol% of **B1** in DMA (1.5 mL).

With the optimized conditions in hand, the scope of the thianthrenium salts was explored under photo- and thermoinduced conditions (Fig. 4). Desulfurative borylation of primary alkyl-substituted thianthrenium salts with alkyl chains (**2–4a**), methoxy (**5a**), ester (**6a**), cyano (**7a**), halides (F, Cl, Br and I, **8–12a**), heterocycles (thiophene, benzofuran, oxazole and carbazole, **13–16a**), alkenes (**17a**) and alkynes (**18–19a**) were converted into the corresponding pinacol boronic esters **2–19c** in good to high yields. Secondary alkyl-substituted substrates, either appended to the acyclic chain (**20–21a**) or to a variety of rings (cyclohexyl, cyclo cyclododecyl and adamantan-2-y, **22–24a**), underwent smooth borylation under the two developed reaction conditions. Benzylic-substituted thianthrenium salt **25a** was also compatible under photoinduced conditions, but only trace amount of the desired product **25c** was observed under thermoinduced conditions. Borylation of thianthrenium salt **26a** containing a perfluoroalkyl chain was facile to form desired product **26c**. Thianthrenium salt **27a**, which is derived from α-linolenic acid with three *cis* double bonds, was compatible with excellent configuration retention. The functional group tolerance of both reaction systems was further tested with complex molecules **28-30a**. Among them, idebenone derivative **30a** was an eligible substrate to provide **30c** in a modest yield with visible light irradiation, but it did not work under the Lewis base system. In addition, substrate **31a** containing two sulfonium salt motifs could undergo double borylation in both reaction systems. In most of the above examples, the photochemical process showed better reactivity than that using the Lewis base. In the case of a thianthrenium salt **32a** with an aromatic C–I bond, the thermal reaction conditions showed excellent chemoselectivity for desulfurative borylation; however, the choice of the photochemical setup led to poor compatibility^52^.

### Synthetic applications

**Fig. 5:**
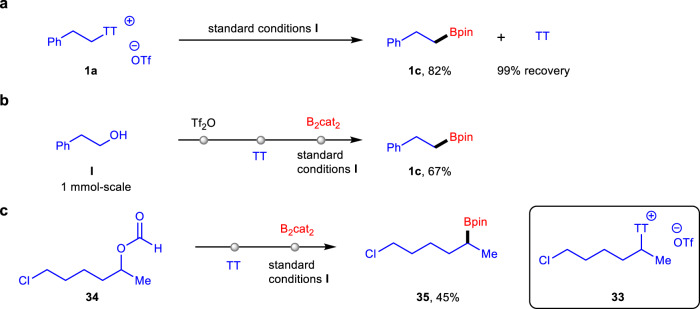
Further investigations. **a** Recovery of TT reagent. **b** One-pot protocol from alcohol **I** to alkylboronate **1c**. **c** One-pot protocol from formyloxy **34** to alkylboronate **35**.

We next turned our attention to improve the practicability and operability of this transformation (Fig. 5). Although using a catalytic amount of TT in our reaction remains challenging at this stage, this compound could, nevertheless, be recycled (Fig. 5a). After the reaction of **1a**, TT reagent was formed accompanying with the main product **1c**. The reaction mixture was then separated by flash column chromatography on silica gel affording the product **1c** in a good yield and regenerating TT reagent in nearly quantitative recovery. To further demonstrate the potential of this transformation to simplify synthesis, one-pot reactions were conducted. For example, the one-pot sulfonation and thianthrenation with alcohol **I** followed by borylation under irradiation conditions led to the generation of alkylboronate **1c** in 67% yield (Fig. 5b). The secondary alkyl-substituted thianthrenium salt **33** bearing a chloride group was not stable and difficult to prepare. Notably, one-pot transformation of formyloxy **34** could provide a convenient way toward the corresponding product **35** (Fig. 5c).

## Discussion

**Fig. 6:**
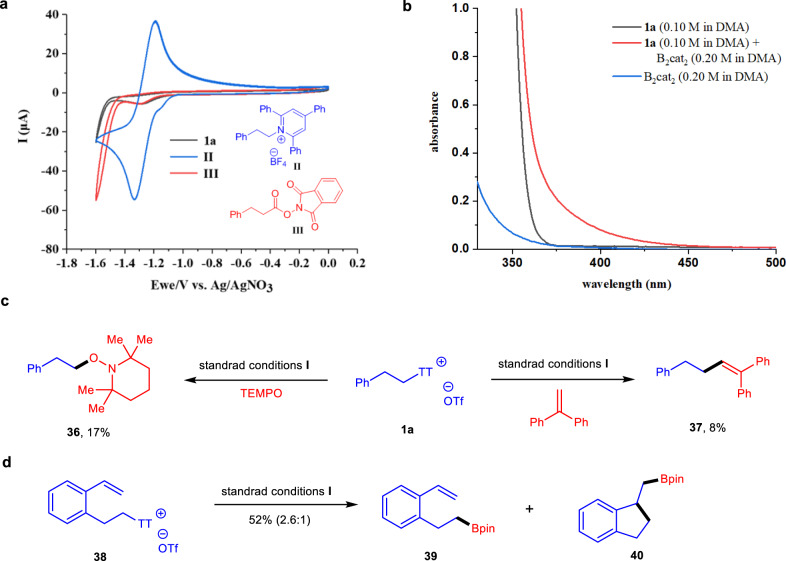
Mechanistic studies. **a** Cyclic voltammograms (CV) of thianthrenium salt **1a**, Katritzky salt **II** and NHPI ester **III**. **b** UV/visible absorption spectra of DMA solutions of **1a** (0.1 M), B_2_Cat_2_ (0.2 M), and a mixture of **1a** (0.1 M) and B_2_cat_2_ (0.2 M) in DMA. **c** Radical trapping experiments of thianthrenium salt **1a**. **d** Radical clock experiment of thianthrenium salt **38**. TEMPO 2,2,6,6-tetramethylpiperidinooxy.

Several mechanistic experiments were then performed to gain insight into the mechanism of this transformation (Fig. 6). We tested the CV of phenethyl-containing thianthrenium salt **1a**, Katritzky salt **II** and NHPI ester **III** (Fig. 6a). Thianthrenium salt **1a** showed irreversible reduction profiles, with *E*^red^ = −1.28 V (versus Ag/AgNO_3_) in DMA, which is more prone to be reduced than Katritzky salt **II** (*E*^red^ = −1.33 V, versus Ag/AgNO_3_) and NHPI ester **III** (*E*^red^ = −1.30 V, versus Ag/AgNO_3_). Then, the absorption spectra of **1a**, B_2_cat_2_, and a mixture of **1a** and B_2_cat_2_ were obtained (Fig. 6b). A bathochromic shift of the mixture proves the strong evidence for the existence of an electron-donor-acceptor (EDA) complex^60,63–65,68–71^. Furthermore, using TEMPO or 1,1-diphenylethylene as radical scavengers in the reactions with visible light irradiation, trapped products **36** and **37** were detected and isolated (Fig. 6c). In addition, desulfurative borylation of **38** could form major product **39** accompanied by cyclization product **40** (Fig. 6d)^72^. These results indicate that alkyl radicals are formed during the reaction. In addition, “light/dark” experiments further showed that visible light was a necessary component of the transformation. Finally, the quantum yield of the transformation to form **1c** was determined to be Ф = 46, indicating that a radical chain process was operative (for details, see Supplementary Information)^73^.

**Fig. 7:**
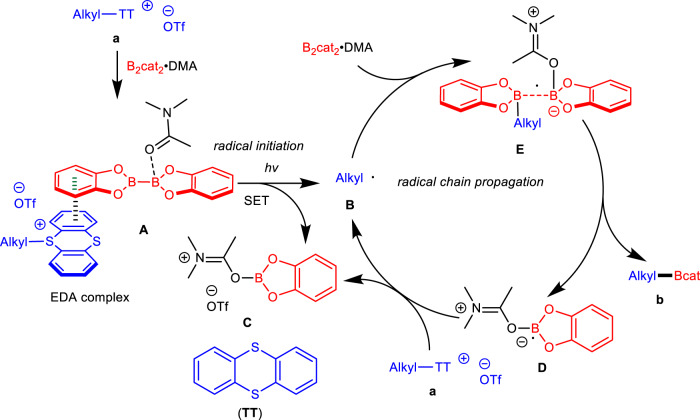
Proposed mechanism. Possible reaction mechanism of of photoinduced desulfurative borylation.

Based on the above observations, a proposed mechanism is shown in Fig. 7. Photoexcitation of EDA complex **A** derived from *S*-(alkyl) thianthrenium salts and B_2_cat_2_·DMA adduct initiates the radical chain reaction, forming alkyl radical **B**, DMA·Bcat adduct **C**, DMA-stabilized boron-centered radical **D**, and TT. Then, propagation of the desulfurative borylation probably proceeds through reaction of alkyl radical **B** with the B_2_cat_2_·DMA adduct to produce radical complex **E**. Cleavage of the B-B bond generates borylated products **b** and boryl radical **D**, which can further reduce substrates **a** to form DMA·Bcat adduct **C** and regenerate alkyl radical **B** (for the proposed mechanism of thermoinduced borylation, see the Supplementary Information).

**Fig. 8:**
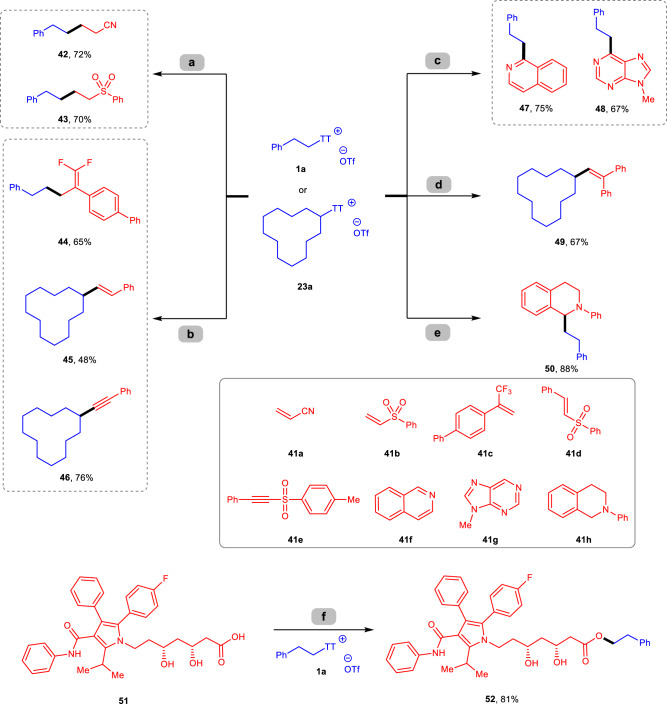
Compatibility of thianthrenium salts in diverse transformations. **a** **1a** (1.0 equiv), **41a-b** (3.0 equiv), Ir[(ppy)_2_dtbpy]PF_6_ (2 mol%), Hantzsch ester (2.0 equiv), DMA, rt, blue LED (440 nm); (**b**) **1a** or **23a** (1.0 equiv), **41c–e** (1.5 equiv), Ir[(ppy)_2_dtbpy]PF_6_ (2 mol%), DIPEA (3.0 equiv), CH_3_CN, rt, blue LED (440 nm); (**c**), for 46: **41f-g** (1.0 equiv), **1a** (1.5–2.0 equiv), Ir[(dFCF_3_ppy)_2_dtbpy]PF_6_ (2 mol%), TfOH, DMA, rt, blue LED (440 nm); (**d**) **23a** (1.0 equiv), 1,1-diphenylethylene (1.5 equiv), Ir[(ppy)_2_dtbpy]PF_6_ (2 mol%), DABCO (1.0 equiv), CH_3_CN, rt, blue LED (440 nm); (**e**) **41** **h** (1.0 equiv), **1a** (2.0 equiv), Ir[(ppy)_2_dtbpy]PF_6_ (2 mol%), KHCO_3_ (3.0 equiv), CH_3_CN, rt, blue LED (440 nm); (**f**) **51** (1.0 equiv), **1a** (1.0 equiv), K_2_CO_3_ (3.0 equiv), THF, rt.

In addition to desulfurative borylation under metal-free conditions, the reaction of thianthrenium salts with several different coupling partners can also be carried out by photoredox catalysis at ambient temperature (Fig. 8). For example, Giese radical addition of thianthrenium salt **1a** with electron-deficient olefins **41a–b** can be accessed to provide products **42**-**43** using Hantzsch ester as a reductant with a catalytic amount of Ir[(ppy)_2_dtbpy]PF_6_ under irradiation with a blue LED^74^. With the same photocatalyst, an efficient and mild defluorinative alkylation of α-trifluoromethyl alkene **41c** using thianthrenium salt **1a** as a radical precursor could produce gem-difluoroalkene **44** in a 65% yield^75^. Under the same reaction conditions, we also developed alkyl radical olefination (**45**) and alkynylation (**46**) reactions with secondary thianthrenium salt **23a** with sulfone reagents **41d**-**e**^76^. The photoredox Minisci-type functionalization of isoquinoline (**41** **f**) and 9-methyl-9*H*-purine (**41** **g**) with **1a** efficiently led to alkylation products **47–48** with catalytic amounts of Ir[dF(CF_3_)ppy]_2_(dtbpy)PF_6_ and triflate acid in DMA under a blue LED^77,78^. Treatment of 1,1-diphenylethylene with substrate **23a** with visible light irradiation could provide the trapped products **49** in 67% yield. Moreover, direct cross-coupling of C(sp^3^)−H bonds in *N*-aryl tetrahydroisoquinoline **41** **h** with thianthrenium salt **1a** by visible-light photoredox system could generate product **50** in an excellent yield^79^. In addition, *S*-(alkyl) thianthrenium salt can also serve as an electrophile reagent to provide an alkyl source with nucleophiles^80^. For example, when a complex molecule atorvastatin (**51**) with several possible reactive sites was examined with thianthrenium salt **1a**, the reaction showed excellent selectivity for esterification product **52** in a good yield.

In conclusion, the challenges associated with sulfonium salts forming nonstabilized alkyl radicals could be efficiently solved by using *S*-(alkyl) thianthrenium salts in SET-induced fragmentations and transformations, especially under mild photoredox systems. These thianthrenium salts bearing a large range of functional groups are suitable electrophiles for borylation, heteroarylation, alkylation, alkenylation, and alkynylation under photochemical conditions. Given the ready availability of alcohols, our approach provides an efficient way to stepwise conversion of OH group to other functional groups, and especially, one-pot manners can be performed with the almost complete recovery of the TT reagent. Extending the developed sulfonium salts to other challenging and useful transformations is currently being explored in our laboratory.

## Methods

### General procedures I

A 10.0 mL Schlenk tube with a stirring bar was added with alkyl sulfonium **1a** (0.4 mmol, 1.0 equiv), B_2_cat_2_ (190.3 mg, 0.8 mmol, 2.0 equiv) and DMA (1.0 mL) under argon. The resulting mixture was stirred at room temperature under blue LED irradiation for 12 h. Then pinacol (189.1 mg, 1.6 mmol, 4.0 equiv) was dissolved in Et_3_N (1.0 mL), added to the reaction mixture and stirred for 1 h. The mixture was cooled to 0 °C to precipitate thianthrene, which was removed by filtration. Then water was added, and the reaction mixture was extracted with EtOAc, dried over MgSO_4,_ and concentrated under reduced pressure. The crude product **1c** was purified by flash column chromatography.

### General procedures II

A 10.0 mL Schlenk tube with a stirring bar was added with alkyl sulfonium **1a** (0.4 mmol, 1.0 equiv), B_2_cat_2_ (190.3 mg, 0.8 mmol, 2.0 equiv), **B1** (26.0 mg, 30.0 mol %) and DMA (1.0 mL) under argon. The resulting mixture was stirred at 80 °C under Ar for 3 h. The mixture was cooled to room temperature. Pinacol (189.1 mg, 1.6 mmol, 4.0 equiv) was dissolved in Et_3_N (1.0 mL), added to the reaction mixture, and stirred for 1 h. The mixture was cooled to 0 °C to precipitate thianthrene, which was removed by filtration. Then water was added, and the reaction mixture was extracted with EtOAc, dried over MgSO_4,_ and concentrated under reduced pressure. The crude product **1c** was purified by flash column chromatography.

## Supplementary information

Supplementary Information

Description of Additional Supplementary Files

Supplementary Data 1

## Data Availability

All data supporting the findings of this study are available within the article and Supplementary Information file or from the corresponding author upon reasonable request. The crystallography data have been deposited at the Cambridge Crystallographic Data Center (CCDC) under accession number CCDC: 2047564 (**1a**) and can be obtained free of charge from www.ccdc.cam.ac.uk/data_request/cif.
