# Supplementary material for: Generation of non-stabilized alkyl radicals from thianthrenium salts for C–B and C–C bond formation
Source: Nat Commun. 2021 Jul 26;12:4526. doi: 10.1038/s41467-021-24716-2 (PMC8313578; doi:10.1038/s41467-021-24716-2)
Supplement: Supplementary file 1 — Supplementary Information [file 41467_2021_24716_MOESM1_ESM.pdf]

Supplementary Information

Generation of Non-Stabilized Alkyl Radicals from Thianthrenium Salts for C–B and C–C Bond Formation

Cheng Chen[1†], Zheng-Jun Wang[1†], Hongjian Lu[1]*, Yue Zhao[1], and Zhuangzhi Shi[1,2]*

Supplementary Methods

General Information

All new compounds were fully characterized. NMR spectra were recorded on Bruker 400 MHz, Varian 500 and calibrated using residual undeuterated solvent ($CHCl_3$ = 7.26 ppm ^{1}H NMR, 77.00 ppm ^{13}C NMR; DMSO = 2.50 ppm ^{1}H NMR, 39.50 ppm ^{13}C NMR; CH_3CN = 1.94 ppm ^{1}H NMR, 1.32 ppm ^{13}C NMR; acetone = 2.05 ppm ^{1}H NMR, 29.92 ppm ^{13}C NMR;) or TMS as an internal reference. ^{1}H NMR coupling constants were reported in Hz, and multiplicity was indicated as follows: s (singlet); d (doublet); t (triplet); q(quartet); quint (quintet); m (multiplet); dd (doublet of doublets); ddd (doublet of doublet of doublets); dddd (doublet of doublet of doublet of doublets); dt (doublet of triplets); td (triplet of doublets); ddt (doublet of doublet of triplets); dq (doublet of quartets); app (apparent); br (broad). Mass spectra were conducted at Mass spectra were conducted at Thermo Scientific LCQ Fleet ESI Mass Spectrometer. For the ReactIR kinetic experiments, the reaction spectra were recorded using an IC 10 and IC 15 from Mettler-Toledo AutoChem. Data manipulation was carried out using the iC IR software, version 4.2. Anhydrous solvents, such as dichloromethane (DCM), dichloroethane (DCE), were purchased from Aldrich. Flash column chromatography was carried out using silica gel (Silicycle, SiliaFlash® P60, 40-63 μm). All reactions were carried out in flame-dried schlenk tube (10 mL) with Teflon screw caps under argon. Unless otherwise noted, materials were obtained from commercial suppliers and used without further purification.

All Photoredox reactions were subjected to irradiation from a 40W Kessil blue LED bulb (440 nm), with the reaction tube placed approximately ~ 1 cm from the bulb in water bath to keep the temperature at 25 °C (Supplementary Figure 1).

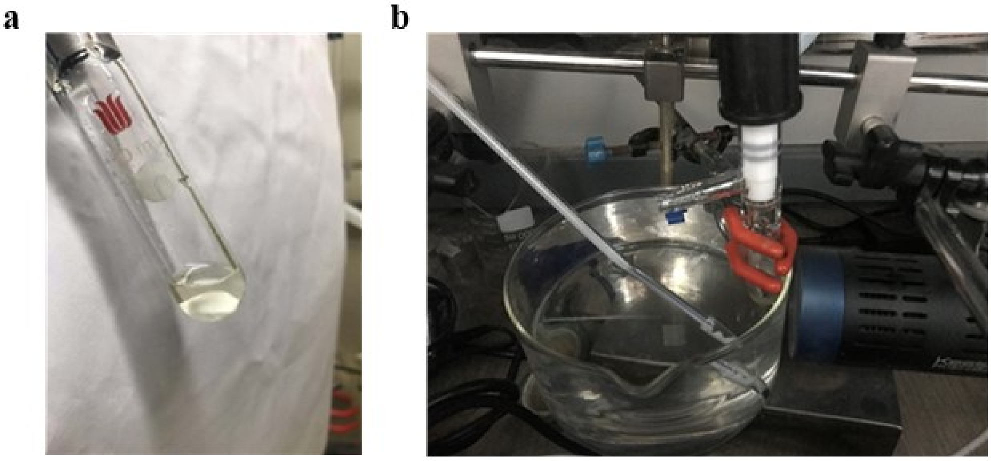

Supplementary Figure 1. Photoreaction setup. a, A flame-dried 10 mL schlenk tube charged with reaction mixture. **b**, Photoreaction setup.

Preparation of *S*-(alkyl)thianthrenium Salts

Method A:

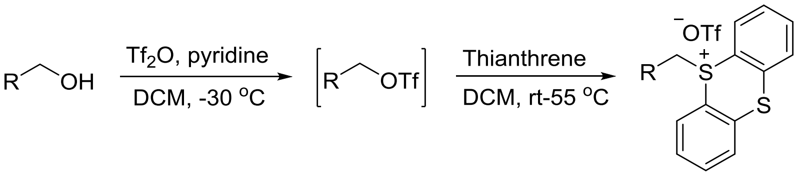

Supplementary Figure 2. Preparation of *S*-(alkyl) thianthrenium salts from the primary alcohols. General procedures for synthesis of *S*-(alkyl) thianthrenium salts from the primary alcohols.

A flame-dried 100 mL flask was placed under an atmosphere of nitrogen and charged with a stir bar and alcohol (5.0 mmol, 1.0 equiv). The alcohol was dissolved in CH_2Cl_2 (20.0 mL) and cooled to -30 °C before adding pyridine (483 μL, 6.0 mmol, 1.2 equiv). While stirring, triflic anhydride (1.0 mL, 29.3 mmol, 1.20 equiv) was added dropwise, and then the reaction mixture stirred for 3 h while remaining at -5 °C. While the flask was still in a -5 °C bath, 0.5 M H_2SO_4 (30 mL) was added. The flask was removed from the cold bath, and the mixture was transferred to a separatory funnel and extracted with 3×20 mL of CH_2Cl_2. The organic layers were combined and washed 1× 50mL of distilled water. The collected organic layers were then dried over $MgSO_4$, then filtered and concentrated to a 10 mL liquid under vacuum (without

heating), which was used directly in the next step.

Flame-dried 25 mL Schlenk tube was added thianthrene (1.08g, 5.0 mmol), then the above liquid was added. The mixture was stired at 55 °C for 24 h. The mixture was carefully condensed under reduced pressure at 25 ºC and purified by precipitation with Et_2O/DCM. Most of the unreacted thianthrene was removed by repeating the precipitation procedure 2 or 3 times. If the salt still did not precipitate, it was subjected to silica gel chromatography with acetone/DCM.

Method B: [1]

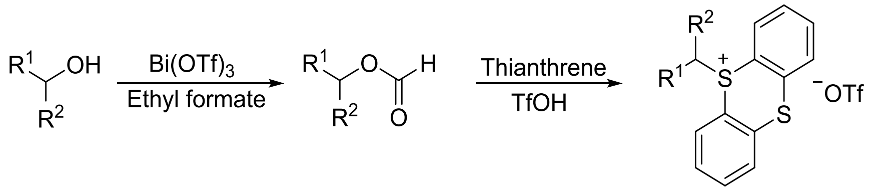

Supplementary Figure 3. Preparation of *S*-(alkyl) thianthrenium salts from the secondary alcohols. General procedures for synthesis of *S*-(alkyl) thianthrenium salts from the secondary alcohols.

A solution of alcohol (20 mmol, 1.0 equiv) in ethyl formate (60 ml, 37.3 equiv) was treated with of $Bi(OTf)_3$ (0.4 mmol, 0.02 equiv). The reaction mixture was stirred under reflux conditions for 5 h. Evaporation of the solvent followed by silica-gel chromatography gave the pure formate.[2]

To a stirred mixture of Thianthrene (1.08 g, 5.0 mmol, 1.0 equiv) and formate (10.0 mmol, 2.0 equiv), cooled in an ice-bath, was added 2.5 ml of trifluoromethanesulfonic acid. The mixture was removed from the ice-bath and stirred for 10 h at room temperature, after which it was poured into 100 ml of water. The resulting suspension was extracted with DCM. The collected organic layers were then dried over $MgSO_4$, then filtered and concentrated under reduced pressure at 25 ºC and purified by precipitation with Et_2O/DCM. Most of the unreacted dibenzothiophene was removed by repeating the precipitation procedure 2 or 3 times.

5-Phenethyl-5*H*-thianthren-5-ium trifluoromethanesulfonate (1a)

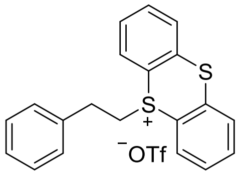

1a

According to Method **A**, **1a** was prepared from the corresponding alcohol (20.0 mmol) as a white solid (6.02 g, 64%): **^{1}H NMR (400 MHz, $CDCl_3$)** δ 8.11 (d, J = 7.4 Hz, 2H), 7.80 (d, J = 7.3 Hz, 2H), 7.71 (t, J = 7.7 Hz, 2H), 7.56 (t, J = 7.2 Hz, 2H), 7.15 (m, 3H), 7.11 – 7.04 (m, 2H), 4.00 (t, J = 7.7 Hz, 2H), 2.95 (t, J = 7.7 Hz, 2H); **^{13}C NMR (101 MHz, $CDCl_3$)** δ 135.6, 135.0, 134.4, 134.4, 130.1, 129.7, 128.9, 128.6, 127.5, 120.8 (q, J = 320.7 Hz), 117.3, 41.6, 30.5; **^{19}F NMR (376 MHz, $CDCl_3$)** δ -78.0; **ATR-FTIR (cm^{-1}):** 3063, 2921, 1569, 1450, 1255, 1149, 1027, 754, 635; **HRMS m/z (ESI)** calculated for $C_{20}H_{17}S_2$ [M – O_3SCF_3]$^+$ 321.0766, found 321.0766.

1-Phenethyltetrahydro-1*H*-thiophen-1-ium trifluoromethanesulfonate (1a')

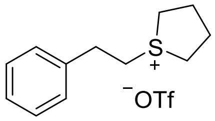

1a'

According to Method **A**, **1a'** was prepared from the corresponding alcohol (5.0 mmol) with tetrahydrothiophene as a white solid (1.45 g, 85%): **^{1}H NMR (400 MHz, CDCN)** δ 7.42 – 7.35 (m, 2H), 7.34 – 7.24 (m, 3H), 3.46 (dt, J = 13.7, 6.8 Hz, 2H), 3.41 – 3.36 (m, 2H), 3.31 (dt, J = 12.8, 6.2 Hz, 2H), 3.12 – 3.06 (m, 2H), 2.35 – 2.15 (m, 4H); **^{13}C NMR (101 MHz, CDCN)** δ 130.0, 129.8, 128.5, 122.1 (q, J = 317.0 Hz), 44.3, 44.2, 31.7, 29.3, 1.9, 1.7; **^{19}F NMR (376 MHz, CDCN)** δ -79.3; **ATR-FTIR (cm^{-1}):** 1263, 1154, 1029, 731, 701, 637; **HRMS m/z (ESI)** calculated for $C_{12}H_{17}S$ [M – O_3SCF_3]$^+$ 193.1045, found 193.1045.

Phenethyldiphenylsulfonium trifluoromethanesulfonate (1a'')

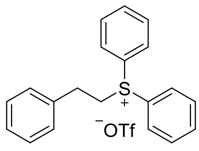

1a''

According to Method **A**, **1a''** was prepared from the corresponding alcohol (5.0 mmol) with diphenylsulfane as a white solid (1.32 g, 60%): **^{1}H NMR (400 MHz, $CDCl_3$)** δ 8.00 – 7.92 (m, 4H), 7.65 (m, 6H), 7.28 – 7.16 (m, 5H), 4.53 (t, J = 7.1 Hz, 2H), 3.13 (t, J = 7.0 Hz, 2H); **^{13}C NMR (101 MHz, $CDCl_3$)** δ 135.4, 134.7, 131.6, 130.7, 129.1, 128.8, 127.8, 124.2, 47.0, 30.7; **^{19}F NMR (376 MHz, $CDCl_3$)** δ -78.1; **ATR-FTIR (cm^{-1}):** 1447, 1254, 1151, 1028, 742, 636; **HRMS m/z (ESI)** calculated for $C_{20}H_{19}S$ [M – O_3SCF_3]$^+$ 291.1202, found 291.1205.

The carbon NMR signal of triflate anion was not observed.

Phenethyldiphenylsulfonium trifluoromethanesulfonate (1a''')

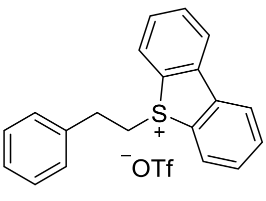

1a'''

According to Method **B**, **1a'''** was prepared from the corresponding formate (10.0 mmol) with dibenzo[*b*,*d*]thiophene (5.0 mmol) as a white solid (942.7 mg, 43%): **^{1}H NMR (500 MHz, DMSO-*d₆*)** δ 8.41 – 8.32 (m, 2H), 8.03 (m, 2H), 7.57 – 7.46 (m, 4H), 7.30 (m, 5H), 4.51 (t, J = 6.8 Hz, 2H), 3.01 (t, J = 6.7 Hz, 2H); **^{13}C NMR (126 MHz, DMSO-d_6)** δ 138.5, 136.5, 135.0, 129.0, 128.5, 127.1, 126.8, 124.7, 123.0, 122.0, 76.4, 35.2; **^{19}F NMR (471 MHz, DMSO-d_6)** δ -77.8; **ATR-FTIR (cm^{-1}):** 1445, 1256, 1151, 1138, 1098, 904, 729; **HRMS m/z (ESI)** calculated for $C_{20}H_{17}S$ [M – O_3SCF_3]$^+$ 289.1045, found 289.1041. All spectral data are in accordance with the literature.[3]
The carbon NMR signal of triflate anion was not observed.

5-Octadecyl-5*H*-thianthren-5-ium trifluoromethanesulfonate (2a)

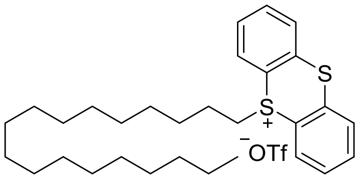

2a

According to the Method **A**, **2a** was prepared from the corresponding alcohol (5.0 mmol) as a gray solid (1.39 g, 45%): **^{1}H NMR (400 MHz, $CDCl_3$)** δ 8.28 (d, J = 7.9 Hz, 2H), 7.83 (dd, J = 7.9, 1.2 Hz, 2H), 7.75 (td, J = 7.7, 1.4 Hz, 2H), 7.65 (td, J = 7.8, 1.3 Hz, 2H), 3.79 – 3.69 (m, 2H), 1.51 (p, J = 7.6 Hz, 2H), 1.35 – 1.13 (m, 30H), 0.84 (t, J = 6.8 Hz, 3H); **^{13}C NMR (101 MHz, $CDCl_3$)** δ 135.6, 134.6, 134.4, 130.0, 129.8, 120.8 (q, J = 321.9 Hz), 117.3, 40.3, 29.6, 29.6, 29.6, 29.5, 29.4, 29.3, 29.0, 28.7, 27.8, 24.4, 22.6, 14.0; **^{19}F NMR (376 MHz, $CDCl_3$)** δ -78.1; **ATR-FTIR (cm^{-1}):** 2922, 2852, 1451, 1257, 1154, 1029, 637; **HRMS m/z (ESI)** calculated for $C_{30}H_{45}S_2$ [M – O_3SCF_3]$^+$ 469.2957, found 469.2952.

5-(2-((3*r*,5*r*,7*r*)-Adamantan-1-yl)ethyl)-5*H*-thianthren-5-ium trifluoromethanesulfonate (3a)

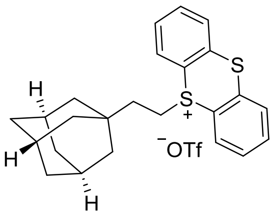

3a

According to the Method **A**, **3a** was prepared from the

corresponding alcohol (5.0 mmol) as a white solid (1.45 g, 55%): **^{1}H NMR (400 MHz, CD_3CN)** δ 8.10 (d, J = 7.9 Hz, 2H), 7.97 – 7.90 (m, 2H), 7.81 (t, J = 7.7 Hz, 2H), 7.70 (t, J = 7.7 Hz, 2H), 3.68 – 3.61 (m, 2H), 1.84 (s, 3H), 1.63 (d, J = 12.0 Hz, 3H), 1.52 (d, J = 11.9 Hz, 3H), 1.33 (s, 6H), 1.22 – 1.15 (m, 2H); **^{13}C NMR (101 MHz, CD_3CN)** δ 135.6, 135.4, 131.2, 130.6, 122.2 (q, J = 322.2 Hz), 42.1, 38.2, 37.2, 36.8, 29.4; **^{19}F NMR (376 MHz, CD_3CN)** δ -79.2; **ATR-FTIR (cm^{-1}):** 2901, 2847, 1450, 1258, 1153, 1029, 636; **HRMS m/z (ESI)** calculated for $C_{24}H_{27}S_2$ [M – O_3SCF_3]$^+$ 379.1549, found 379.1544.

5-(3-(4-Isopropylphenyl)-2-methylpropyl)-5*H*-thianthren-5-ium trifluoromethanesulfonate (4a)

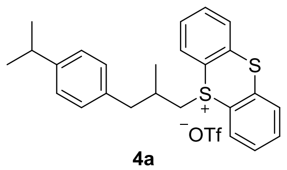

4a

According to the Method **A**, **4a** was prepared from the corresponding alcohol (5.0 mmol) as a gray oil (1.11 g, 41%): **^{1}H NMR (400 MHz, CD_3CN)** δ 8.05 (dt, J = 8.0, 1.3 Hz, 2H), 7.85 (dd, J = 8.0, 1.2 Hz, 1H), 7.83 – 7.78 (m, 2H), 7.77 (dd, J = 7.9, 1.4 Hz, 1H), 7.73 – 7.64 (m, 2H), 7.10 (d, J = 8.0 Hz, 2H), 6.82 (d, J = 8.1 Hz, 2H), 3.84 (dd, J = 12.3, 4.2 Hz, 1H), 3.23 (dd, J = 12.3, 8.9 Hz, 1H), 2.89 (p, J = 6.9 Hz, 1H), 2.63 (dd, J = 13.7, 6.5 Hz, 1H), 2.49 (dd, J = 13.7, 8.6 Hz, 1H), 1.67 (m, 1H), 1.25 (dd, J = 6.9, 1.5 Hz, 6H), 1.15 (d, J = 6.7 Hz, 3H); **^{13}C NMR (101 MHz, CD_3CN)** δ 136.8, 135.6, 135.3, 135.0, 131.3, 131.2, 130.6, 130.6, 129.8, 127.7, 45.7, 41.6, 34.5, 24.4, 19.1; **^{19}F NMR (376 MHz, $CDCl_3$)** δ -79.3; **ATR-FTIR (cm^{-1}):** 2961, 1263, 1154, 1028, 732, 636; **HRMS m/z (ESI)** calculated for $C_{25}H_{27}S_2$ [M – O_3SCF_3]$^+$ 391.1549, found 391.1548.

The carbon NMR signal of triflate anion was not observed.

5-(3-Methoxypropyl)-5*H*-thianthren-5-ium trifluoromethanesulfonate (5a)

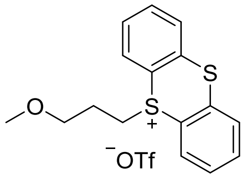

5a

According to the Method **A**, **5a** was prepared from the corresponding alcohol (5.0 mmol) as a white solid (753 mg, 34%): **^{1}H NMR (500 MHz, $CDCl_3$)** δ 8.17 (d, J = 7.9 Hz, 2H), 7.79 (d, J = 7.8 Hz, 2H), 7.71 (t, J = 7.5 Hz, 2H), 7.60 (t, J = 7.6 Hz, 2H), 3.84 (t, J = 6.7 Hz, 2H), 3.47 (t, J = 5.3 Hz, 2H), 3.30 (s, 3H), 1.74 (p, J =

6.2 Hz, 2H); **^{13}C NMR (126 MHz, $CDCl_3$)** 35.5, 134.7, 134.3, 130.0, 129.5, 120.6 (q, J = 321.4 Hz), 117.2, 69.9, 58.5, 39.1, 24.7; **^{19}F NMR (471 MHz, $CDCl_3$)** δ -78.1; **ATR-FTIR (cm^{-1}):** 2931, 1253, 1150, 1028, 761, 635; **HRMS m/z (ESI)** calculated for $C_{16}H_{17}OS_2$ [M – O_3SCF_3]$^+$ 289.0715, found 289.0713.

5-(6-Ethoxy-6-oxohexyl)-5*H*-thianthren-5-ium trifluoromethanesulfonate (6a)

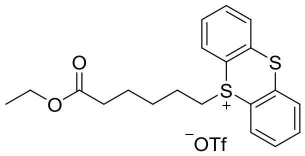

6a

According to Method **A**, **6a** was prepared from the corresponding alcohol (5.0 mmol) as a corlorless oil (1.53 g, 60%): **^{1}H NMR (400 MHz, $CDCl_3$)** δ 8.32 – 8.26 (m, 2H), 7.82 (dd, J = 7.9, 1.3 Hz, 2H), 7.75 (td, J = 7.7, 1.4 Hz, 2H), 7.66 (td, J = 7.7, 1.3 Hz, 2H), 4.05 (q, J = 7.2 Hz, 2H), 3.82 – 3.72 (m, 2H), 2.21 (t, J = 7.1 Hz, 2H), 1.53 (m, 4H), 1.46 – 1.37 (m, 2H), 1.20 (t, J = 7.1 Hz, 3H); **^{13}C NMR (101 MHz, $CDCl_3$)** δ 135.6, 134.8, 130.0, 129.9, 120.7 (q, J = 321.8 Hz), 60.3, 40.1, 33.4, 27.1, 24.2, 23.9, 14.1; **^{19}F NMR (376 MHz, $CDCl_3$)** δ -78.2; **ATR-FTIR (cm^{-1}):** 1725, 1263, 1029, 730, 637; **HRMS m/z (ESI)** calculated for $C_{20}H_{23}O_2S_2$ [M – O_3SCF_3]$^+$ 359.1134, found 359.1137.

5-(4-Cyanophenethyl)-5*H*-thianthren-5-ium trifluoromethanesulfonate (7a)

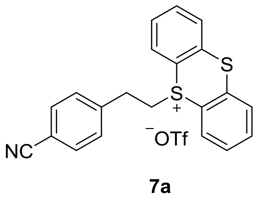

7a

According to Method **A**, **7a** was prepared from the corresponding alcohol (5.0 mmol) as a white solid (990 mg, 40%): **^{1}H NMR (400 MHz, CD_3CN)** δ 8.01 (dd, J = 7.9, 1.2 Hz, 2H), 7.88 (dd, J = 8.0, 1.0 Hz, 2H), 7.77 (td, J = 7.8, 1.3 Hz, 2H), 7.64 (td, J = 7.7, 1.3 Hz, 2H), 7.57 (d, J = 8.3 Hz, 2H), 7.28 (d, J = 8.4 Hz, 2H), 3.97 (t, J = 7.5 Hz, 2H), 3.02 (t, J = 7.4 Hz, 2H); **^{13}C NMR (101 MHz, CD_3CN)** δ 142.4, 137.0, 135.6, 135.2, 133.5, 131.3, 130.6, 130.6, 122.1 (q, J = 319.7 Hz), 119.4, 117.8, 41.4, 31.0, 1.7; **^{19}F NMR (471 MHz, CD_3CN)** δ -79.1; **ATR-FTIR (cm^{-1}):** 3060, 2228, 1450, 1262, 1028, 732, 636; **HRMS m/z (ESI)** calculated for $C_{21}H_{16}NS_2$ [M – O_3SCF_3]$^+$ 346.0719, found 346.0718.

5-(4-Fluorophenethyl)-5*H*-thianthren-5-ium trifluoromethanesulfonate (8a)

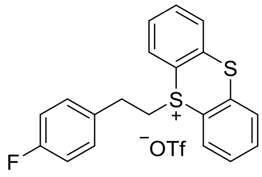

8a

According to Method **A**, **8a** was prepared from the corresponding alcohol (5.0 mmol) as a white solid (1.29 g, 53%): **^{1}H NMR (500 MHz, CDCl$_3$)** δ 8.07 (d, J = 7.8 Hz, 2H), 7.79 (d, J = 7.8 Hz, 2H), 7.70 (t, J = 7.5 Hz, 2H), 7.55 (t, J = 7.5 Hz, 2H), 7.13 – 6.96 (m, 2H), 6.80 (t, J = 8.4 Hz, 2H), 4.04 – 3.81 (m, 2H), 2.99 – 2.77 (m, 2H); **^{13}C NMR (126 MHz, CDCl$_3$)** δ 161.8 (d, J = 246.3 Hz), 135.6, 134.4, 134.1, 130.7 (d, J = 3.1 Hz), 130.3 (d, J = 8.1 Hz), 130.1, 129.6, 120.6 (q, J = 321.4 Hz), 116.9, 115.5 (d, J = 21.4 Hz), 41.3, 29.5; **^{19}F NMR (471 MHz, CDCl$_3$)** δ -77.9, -114.5; **ATR-FTIR (cm^{-1}):** 3070, 2999, 1509, 1261, 1151, 1028, 635; **HRMS m/z (ESI)** calculated for $C_{20}H_{16}FS_2$ [M – O_3SCF_3]$^+$ 339.0672, found 339.0671.

5-(4-Chlorobutyl)-5*H*-thianthren-5-ium trifluoromethanesulfonate (9a)

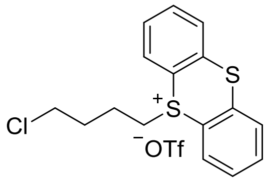

9a

According to Method **A**, **9a** was prepared from the corresponding alcohol (5.0 mmol) as a faint yellow oil (1.19 g, 52%): **^{1}H NMR (400 MHz, CDCl$_3$)** δ 8.30 (d, J = 7.9 Hz, 2H), 7.84 (d, J = 7.9 Hz, 2H), 7.76 (t, J = 7.2 Hz, 2H), 7.67 (t, J = 8.1 Hz, 2H), 3.94 – 3.81 (m, 2H), 3.49 (t, J = 6.1 Hz, 2H), 1.91 (dt, J = 12.5, 6.2 Hz, 2H), 1.73 (p, J = 7.5, 7.1 Hz, 2H); **^{13}C NMR (101 MHz, CDCl$_3$)** δ 135.7, 134.8, 134.6, 130.1, 129.9, 120.7 (q, J = 321.6 Hz), 117.0, 43.7, 39.6, 30.0, 21.8; **^{19}F NMR (376 MHz, CDCl$_3$)** δ -78.2; **ATR-FTIR (cm^{-1}):** 1405, 1262, 1153, 1028, 756, 636; **HRMS m/z (ESI)** calculated for $C_{16}H_{16}ClS_2$ [M – O_3SCF_3]$^+$ 307.0376, found 307.0376.

5-(4-Bromophenethyl)-5*H*-thianthren-5-ium trifluoromethanesulfonate (10a)

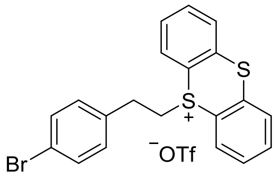

10a

According to Method **A**, **10a** was prepared from the corresponding alcohol (5.0 mmol) as a white solid (1.51 g, 55%): **^{1}H NMR (500 MHz, CDCl$_3$)** δ 8.08 (d, J = 7.9 Hz, 2H), 7.79 (d, J = 7.9 Hz, 2H), 7.71 (t, J = 7.7 Hz, 2H), 7.56 (t, J = 7.7 Hz, 2H), 7.22 (d, J = 7.8 Hz, 2H), 6.99 (d, J = 8.1 Hz, 2H), 3.97 (t, J = 7.6 Hz,

2H), 2.91 (t, J = 7.6 Hz, 2H); **^{13}C NMR (126 MHz, $CDCl_3$)** δ 135.6, 134.4, 134.2, 134.1, 131.8, 130.4, 130.1, 129.7, 121.4, 120.7 (q, J = 320.7 Hz), 116.9, 41.1, 29.8; **^{19}F NMR (471 MHz, $CDCl_3$)** δ -77.9; **ATR-FTIR (cm^{-1}):** 3061, 1450, 1255, 1153, 1028, 636; **HRMS m/z (ESI)** calculated for $C_{20}H_{16}BrS_2$ $[M - O_3SCF_3]^+$ 398.9871, 400.9854; found 398.9867, 400.9843.

5-(3-Bromopropyl)-5*H*-thianthren-5-ium trifluoromethanesulfonate (11a)

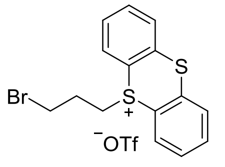

11a

According to Method **A**, **11a** was prepared from the corresponding alcohol (5.0 mmol) as a corlorless oil (1.83 g, 75%): **^{1}H NMR (400 MHz, $CDCl_3$)** δ 8.29 (d, J = 7.8 Hz, 2H), 7.89 (d, J = 7.8 Hz, 2H), 7.81 (t, J = 7.6 Hz, 2H), 7.70 (t, J = 7.7 Hz, 2H), 4.02 – 3.91 (m, 2H), 3.45 (t, J = 6.2 Hz, 2H), 2.19 (dt, J = 12.9, 6.3 Hz, 2H); **^{13}C NMR (101 MHz, $CDCl_3$)** δ 135.8, 134.7, 134.4, 130.2, 129.9, 120.7 (q, J = 320.5 Hz), 116.6, 39.1, 29.8, 27.1; **^{19}F NMR (376 MHz, $CDCl_3$)** δ -78.1; **ATR-FTIR (cm^{-1}):** 1405, 1263, 731, 663; **HRMS m/z (ESI)** calculated for $C_{15}H_{14}BrS_2$ $[M - O_3SCF_3]^+$ 336.9715, found 336.9716.

5-(3-Iodopropyl)-5*H*-thianthren-5-ium trifluoromethanesulfonate (12a)

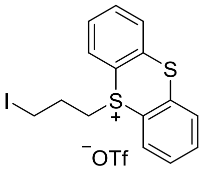

12a

According to the Method **A**, **12a** was prepared from the corresponding alcohol (5.0 mmol) as a gray solid (1.10 g, 41%): **^{1}H NMR (400 MHz, $CDCl_3$)** δ 8.28 (dd, J = 7.9, 1.1 Hz, 2H), 7.86 (dd, J = 7.9, 1.1 Hz, 2H), 7.77 (td, J = 7.7, 1.3 Hz, 2H), 7.67 (td, J = 7.7, 1.3 Hz, 2H), 3.97 – 3.78 (m, 2H), 3.16 (t, J = 6.5 Hz, 2H), 2.15 – 2.04 (m, 2H); **^{13}C NMR (101 MHz, $CDCl_3$)** δ 135.8, 134.7, 134.6, 130.2, 130.0, 120.7 (q, J = 320.5 Hz),116.8, 41.03, 27.7, 1.4; **^{19}F NMR (376 MHz, $CDCl_3$)** δ -78.1; **ATR-FTIR (cm^{-1}):** 1406, 1258, 1153, 1028, 758, 636; **HRMS m/z (ESI)** calculated for $C_{15}H_{14}IS_2$ $[M - O_3SCF_3]^+$ 384.9576, found 384.9575.

5-(2-(Thiophen-2-yl)ethyl)-5*H*-thianthren-5-ium trifluoromethanesulfonate (13a)

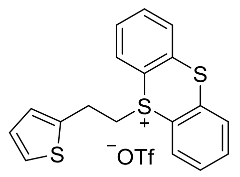

13a

According to the Method **A**, **13a** was prepared from the corresponding alcohol (5.0 mmol) as a white solid (1.60 g, 67%): **^{1}H NMR (400 MHz, CDCl$_3$)** δ 8.07 (dd, J = 7.9, 1.1 Hz, 2H), 7.80 (dd, J = 7.9, 1.0 Hz, 2H), 7.70 (td, J = 7.8, 1.3 Hz, 2H), 7.56 (td, J = 7.8, 1.2 Hz, 2H), 7.05 (dd, J = 5.1, 1.1 Hz, 1H), 6.93 (d, J = 2.7 Hz, 1H), 6.82 (dd, J = 5.1, 3.5 Hz, 1H), 4.05 (t, J = 7.2 Hz, 2H), 3.27 (t, J = 7.2 Hz, 2H); **^{13}C NMR (101 MHz, CDCl$_3$)** δ 137.0, 135.7, 134.4, 134.2, 130.1, 129.8, 127.4, 127.2, 125.5, 120.7 (q, J = 320.7 Hz), 117.3, 42.3, 25.1; **^{19}F NMR (376 MHz, CDCl$_3$)** δ -78.1; **ATR-FTIR (cm^{-1}):** 3077, 1255, 1152, 1028, 757, 635; **HRMS m/z (ESI)** calculated for $C_{18}H_{15}S_3$ [M – O_3SCF_3]$^+$ 327.0330, found 327.0330.

5-(2-(Benzofuran-2-yl)ethyl)-5*H*-thianthren-5-ium trifluoromethanesulfonate (14a)

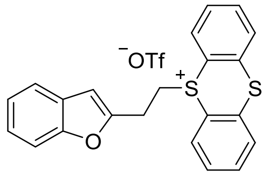

14a

According to the Method **A**, **14a** was prepared from the corresponding alcohol (5.0 mmol) as a gray solid (1.43 g, 56%): **^{1}H NMR (400 MHz, CD$_3$CN)** δ 7.99 (dd, J = 8.0, 1.2 Hz, 2H), 7.87 (d, J = 8.0 Hz, 2H), 7.69 (t, J = 7.5 Hz, 2H), 7.58 – 7.50 (m, 3H), 7.35 (d, J = 8.0 Hz, 1H), 7.30 – 7.20 (m, 2H), 6.64 (s, 1H), 4.08 (t, J = 6.6 Hz, 2H), 3.19 (t, J = 6.6 Hz, 2H); **^{13}C NMR (101 MHz, CD$_3$CN)** δ 155.7, 153.3, 137.0, 135.4, 135.3, 131.2, 130.4, 129.2, 125.4, 124.1, 122.0, 111.8, 117.6, 106.4, 39.9, 24.6; **^{19}F NMR (376 MHz, CD$_3$CN)** δ -79.2; **ATR-FTIR (cm^{-1}):** 3062, 1263, 1156, 1029, 753, 637; **HRMS m/z (ESI)** calculated for $C_{22}H_{17}OS_2$ [M – O_3SCF_3]$^+$ 361.0715, found 361.0718.

The carbon NMR signal of triflate anion was not observed.

5-(2-(5-Methyl-2-phenyloxazol-4-yl)ethyl)-5*H*-thianthren-5-ium trifluoromethanesulfonate (15a)

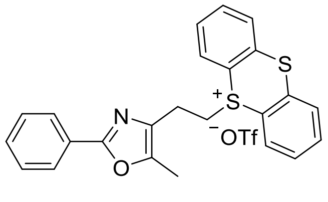

15a

According to the Method **A**, **15a** was prepared from the corresponding alcohol (5.0 mmol) as a yellow oil (1.38 g, 50%): **^{1}H NMR (400 MHz, CD$_3$CN)** δ 8.06 (dd, J = 7.9,

1.3 Hz, 2H), 7.94 – 7.88 (m, 2H), 7.86 (dd, *J* = 7.9, 1.2 Hz, 2H), 7.68 (td, *J* = 7.7, 1.4 Hz, 2H), 7.61 (td, *J* = 7.7, 1.3 Hz, 2H), 7.51 (dd, *J* = 5.0, 1.9 Hz, 3H), 4.04 (t, *J* = 6.2 Hz, 2H), 2.86 (t, *J* = 6.2 Hz, 2H), 2.28 (s, 3H); **^{13}C NMR (101 MHz, CD_3CN)** δ 160.4, 146.6, 137.1, 135.4, 135.2, 131.8, 131.3, 131.1, 130.5, 130.0, 128.2, 126.8, 118.1, 41.1, 21.9, 10.3; **^{19}F NMR (376 MHz, CD_3CN)** δ -79.2; **ATR-FTIR (cm^{-1}):** 3057, 1263, 1153, 1029, 730; **HRMS m/z (ESI)** calculated for $C_{24}H_{20}NOS_2$ [M – O_3SCF_3]$^+$ 402.0981, found 402.0972.

The carbon NMR signal of triflate anion was not observed.

5-(5-(9*H*-carbazol-9-yl)pentyl)-5*H*-thianthren-5-ium trifluoromethanesulfonate (16a)

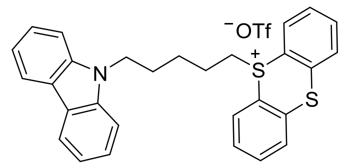

16a

According to the Method **A**, **16a** was prepared from the corresponding alcohol (5.0 mmol) as a white solid (602 mg, 20%): **^{1}H NMR (400 MHz, CD_3CN)** δ 8.11 (d, *J* = 7.7 Hz, 2H), 7.90 (dd, *J* = 7.9, 1.2 Hz, 2H), 7.83 (dd, *J* = 8.0, 1.2 Hz, 2H), 7.74 (td, *J* = 7.8, 1.3 Hz, 2H), 7.59 (td, *J* = 7.7, 1.3 Hz, 2H), 7.49 – 7.42 (m, 4H), 7.22 (ddd, *J* = 7.9, 5.8, 2.3 Hz, 2H), 4.29 (t, *J* = 6.8 Hz, 2H), 3.54 – 3.46 (m, 2H), 1.75 (dt, *J* = 14.4, 6.9 Hz, 2H), 1.48 – 1.42 (m, 2H), 1.36 – 1.29 (m, 2H).; **^{13}C NMR (101 MHz, CD_3CN)** δ 141.3, 136.9, 135.6, 135.0, 131.2, 130.5, 123.5, 121.2, 119.9, 117.7, 110.0, 43.1, 41.1, 28.8, 25.8, 24.9; **^{19}F NMR (376 MHz, CD_3CN)** δ -79.3; **ATR-FTIR (cm^{-1}):** 3056, 2930, 1452, 1262, 728; **HRMS m/z (ESI)** calculated for $C_{29}H_{26}NS_2$ [M – O_3SCF_3]$^+$ 452.1501, found 452.1502.

The carbon NMR signal of triflate anion was not observed.

(*Z*)-5-(octadec-9-en-1-yl)-5*H*-thianthren-5-ium trifluoromethanesulfonate (17a)

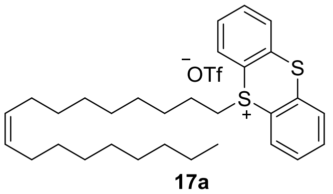

17a

According to Method **A**, **17a** was prepared from the corresponding alcohol (5.0 mmol) at room temperature as a corlorless oil (648 mg, 21%): **^{1}H NMR (400 MHz, $CDCl_3$)** δ 8.32 (dd, *J* = 7.9, 1.1 Hz, 2H), 7.82 (dd, *J* = 7.9, 1.2 Hz, 2H), 7.75 (td, *J* = 7.7, 1.3 Hz, 2H), 7.66 (td, *J* = 7.7, 1.3 Hz, 2H), 5.34 – 5.26 (m, 2H), 3.80 – 3.73 (m,

2H), 1.98 – 1.94 (m, 2H), 1.53 (p, J = 7.6 Hz, 2H), 1.44 – 0.99 (m, 24H), 0.86 (t, J = 6.8 Hz, 3H); **^{13}C NMR (101 MHz, $CDCl_3$)** δ 135.6, 134.8, 134.4, 130.0, 129.9, 129.6, 120.8 (q, J = 320.6 Hz), 117.5, 40.4, 31.8, 29.7, 29.6, 29.5, 29.3, 29.0, 28.7, 27.8, 27.2, 27.1, 24.4, 22.6, 14.1; **^{19}F NMR (376 MHz, $CDCl_3$)** δ -78.2; **ATR-FTIR (cm^{-1}):** 2923, 2853, 1256, 1154, 1029, 637; **HRMS m/z (ESI)** calculated for $C_{30}H_{43}S_2$ [M – O_3SCF_3]$^+$ 467.2801, found 467.2791.

5-(Dec-3-yn-1-yl)-5*H*-thianthren-5-ium trifluoromethanesulfonate (18a)

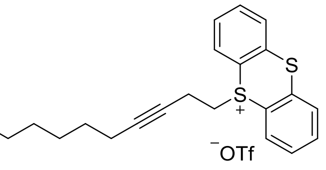

18a

According to Method **A**, **18a** was prepared from the corresponding alcohol (5.0 mmol) as a gray oil (879 mg, 35%): **^{1}H NMR (400 MHz, $CDCl_3$)** δ 8.20 (d, J = 7.9 Hz, 2H), 7.82 (d, J = 7.9 Hz, 2H), 7.73 (t, J = 7.7 Hz, 2H), 7.61 (t, J = 7.6 Hz, 2H), 3.84 (t, J = 6.4 Hz, 2H), 2.54 (t, J = 6.0 Hz, 2H), 2.01 – 1.90 (m, 2H), 1.34 (m, 2H), 1.27 – 1.13 (m, 6H), 0.81 (t, J = 6.8 Hz, 3H); **^{13}C NMR (101 MHz, $CDCl_3$)** δ 136.0, 134.6, 134.4, 130.1, 129.6, 120.6 (q, J = 320.9 Hz), 116.6, 86.0, 73.0, 40.1, 31.0, 28.3, 28.2, 22.3, 18.4, 15.3, 13.8; **^{19}F NMR (376 MHz, $CDCl_3$)** δ -78.1; **ATR-FTIR (cm^{-1}):** 2930, 1450, 1253, 1028, 761, 636; **HRMS m/z (ESI)** calculated for $C_{22}H_{25}S_2$ [M – O_3SCF_3]$^+$ 353.1392, found 353.1393.

5-(Hept-6-en-3-yn-1-yl)-5*H*-thianthren-5-ium trifluoromethanesulfonate (19a)

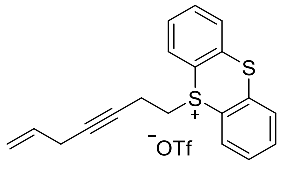

19a

According to the Method **A**, **19a** was prepared from the corresponding alcohol (5.0 mmol) as a gray solid (871 mg, 38%): **^{1}H NMR (400 MHz, $CDCl_3$)** δ 8.28 (dd, J = 7.9, 1.1 Hz, 2H), 7.84 (dd, J = 7.9, 1.2 Hz, 2H), 7.75 (td, J = 7.7, 1.4 Hz, 2H), 7.64 (td, J = 7.7, 1.3 Hz, 2H), 5.82 – 5.54 (m, 1H), 5.17 (dq, J = 17.0, 1.7 Hz, 1H), 5.07 (dq, J = 10.0, 1.6 Hz, 1H), 3.92 (t, J = 6.5 Hz, 2H), 2.80 (dp, J = 5.9, 2.1 Hz, 2H), 2.66 (ddd, J = 8.8, 6.4, 2.3 Hz, 2H); **^{13}C NMR (101 MHz, $CDCl_3$)** δ 136.1, 134.8, 134.6, 131.9, 130.1, 129.8, 120.7 (q, J = 320.5 Hz), 116.9, 116.3, 82.4, 75.6, 40.0, 22.9, 15.5; **^{19}F NMR (376 MHz, $CDCl_3$)** δ -78.1; **ATR-FTIR (cm^{-1}):**

3079, 3001, 1262, 1154, 1029, 636; **HRMS m/z (ESI)** calculated for $C_{19}H_{17}S_2$ [M – O_3SCF_3]$^+$ 309.0766, found 309.0767.

5-(*Sec*-butyl)-5*H*-thianthren-5-ium trifluoromethanesulfonate (20a)

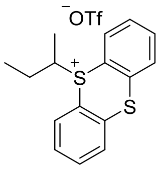

20a

According to the Method **B**, **20a** was prepared from the corresponding formate (10.0 mmol) as a white solid (845 mg, 40%): **^{1}H NMR (400 MHz, CD_3CN)** δ 8.14 – 8.03 (m, 2H), 7.95 (d, *J* = 8.0 Hz, 2H), 7.84 (td, *J* = 7.8, 1.3 Hz, 2H), 7.71 (tt, *J* = 7.8, 1.5 Hz, 2H), 4.45 – 4.36 (m, 1H), 1.63 – 1.56 (m, 2H), 1.21 (d, *J* = 6.8 Hz, 3H), 0.95 (t, *J* = 7.4 Hz, 3H); **^{13}C NMR (101 MHz, CD_3CN)** δ137.1, 135.9, 135.8, 135.7, 131.3, 131.2, 130.6, 117.7, 117.5, 54.0, 25.3, 15.1, 9.9, 1.7; **^{19}F NMR (376 MHz, CD_3CN)** δ -79.2; **ATR-FTIR (cm $^{-1}$):** 3054， 2998， 1264， 731， 703; **HRMS m/z (ESI)** calculated for $C_{16}H_{17}S_2$ [M – O_3SCF_3]$^+$ 273.0766, found 273.0762. All spectral data are in accordance with the literature.[1]

The carbon NMR signal of triflate anion was not observed.

5-(1-Phenylpropan-2-yl)-5*H*-thianthren-5-ium trifluoromethanesulfonate (21a)

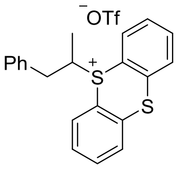

21a

According to the Method **B**, **21a** was prepared from the corresponding formate (10.0 mmol) as a semi-solid (872 mg, 36%): **^{1}H NMR (400 MHz, CD_3CN)** δ 8.07 (d, *J* = 7.9 Hz, 1H), 7.88 (d, *J* = 7.9 Hz, 1H), 7.84 – 7.76 (m, 3H), 7.73 – 7.65 (m, 2H), 7.54 (t, *J* = 8.2 Hz, 1H), 7.23 – 7.17 (m, 3H), 7.03 (dd, *J* = 6.4, 2.8 Hz, 2H), 4.59 (dq, *J* = 13.4, 6.7 Hz, 1H), 3.03 (dd, *J* = 14.1, 8.3 Hz, 1H), 2.90 (dd, *J* = 14.1, 6.3 Hz, 1H), 1.13 (d, *J* = 6.8 Hz, 3H); **^{13}C NMR (101 MHz, CD_3CN)** δ 135.8, 135.6, 135.4, 131.3, 130.6, 130.3, 129.9, 129.6, 129.0, 128.6, 118.0, 54.5, 38.8, 16.7; **^{19}F NMR (376 MHz, CD_3CN)** δ -79.1; **ATR-FTIR (cm $^{-1}$):** 3044, 3038, 2990, 1265, 1114, 730, 622; **HRMS m/z (ESI)** calculated for $C_{21}H_{19}S_2$ [M – O_3SCF_3]$^+$ 335.0923, found 335.0925.

The carbon NMR signal of triflate anion was not observed.

5-Cyclohexyl-5*H*-thianthren-5-ium trifluoromethanesulfonate (22a)

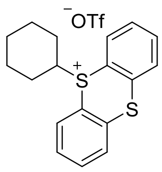

22a

According to the Method **B**, **22a** was prepared from the corresponding formate (10.0 mmol) as a white solid (1.85 g, 76%): **^{1}H NMR (400 MHz, CD_3CN)** δ 8.05 (d, J = 7.9 Hz, 2H), 7.94 (d, J = 8.0 Hz, 2H), 7.83 (td, J = 7.8, 1.3 Hz, 2H), 7.70 (td, J = 7.7, 1.2 Hz, 2H), 4.31 (p, J = 8.4, 7.8 Hz, 1H), 1.82 – 1.74 (m, 2H), 1.62 – 1.54 (m, 5H), 1.28 (m, 3H); **^{13}C NMR (101 MHz, CD_3CN)** δ 137.0, 135.8, 135.7, 131.2, 130.5, 117.0, 55.1, 28.8, 25.3, 25.1; **^{19}F NMR (376 MHz, CD_3CN)** δ -79.2; **ATR-FTIR (cm $^{-1}$):** 2950, 2931, 2250, 1315, 1031, 906, 737; **HRMS m/z (ESI)** calculated for $C_{18}H_{19}S_2$ [M – O_3SCF_3]$^+$ 299.0923, found 299.0921. All spectral data are in accordance with the literature.[1]

The carbon NMR signal of triflate anion was not observed.

5-Cyclododecyl-5*H*-thianthren-5-ium trifluoromethanesulfonate (23a)

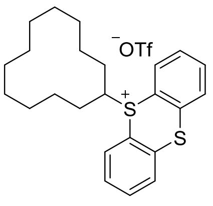

23a

According to the Method **B**, **23a** was prepared from the corresponding formate as a white solid (1.86 g, 70%): **^{1}H NMR (400 MHz, CD_3CN)** δ 8.11 (d, J = 7.6 Hz, 2H), 7.95 (d, J = 6.3 Hz, 2H), 7.83 (t, J = 7.4 Hz, 2H), 7.71 (t, J = 7.4 Hz, 2H), 4.50 (br, 1H), 1.77 – 1.65 (m, 2H), 1.34 (m, 20H); **^{13}C NMR (101 MHz, CD_3CN)** δ 137.2, 135.8, 135.7, 131.4, 130.7, 117.8, 54.6, 26.2, 25.4, 25.1, 23.7, 23.5, 21.7; **^{19}F NMR (376 MHz, CD_3CN)** δ -79.2; **ATR-FTIR (cm $^{-1}$):** 2930, 2860, 1261, 1029, 737; **HRMS m/z (ESI)** calculated for $C_{24}H_{31}S_2$ [M – O_2SCF_3]$^+$ 383.1862, found 383.1866.

The carbon NMR signal of triflate anion was not observed.

5-((1*r*,3*s*,5*R*,7*S*)-adamantan-2-yl)-5*H*-thianthren-5-ium trifluoromethanesulfonate (24a)

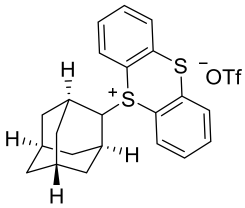

24a

According to the Method **B**, **24a** was prepared from the corresponding formate as a white solid (1.20 g, 48%): **^{1}H NMR (400 MHz, CD_3CN)** δ 8.08 (dd, J = 7.9, 1.1 Hz, 2H), 7.94 (dd, J = 8.0, 2.8 Hz, 2H), 7.82 (td, J = 7.8, 1.3 Hz, 2H), 7.69 (td, J = 7.9, 1.3 Hz, 2H), 4.70 (s, 1H), 2.32 (d, J = 13.6

Hz, 2H), 2.08 (s, 1H), 1.91 – 1.82 (m, 3H), 1.78 (d, *J* = 13.3 Hz, 4H), 1.63 (d, *J* = 12.1 Hz, 4H).; **^{13}C NMR (101 MHz, CD_3CN)** δ 137.1, 135.7, 135.5, 131.5, 130.6, 122.2 (q, *J* = 321.0 Hz), 116.8, 63.1, 37.8, 37.0, 31.7, 30.0, 28.0, 27.4; **^{19}F NMR (376 MHz, CD_3CN)** δ -79.2; **ATR-FTIR (cm^{-1}):** 2913, 1263, 1150, 1029, 731; **HRMS m/z (ESI)** calculated for $C_{22}H_{23}S_2$ [M – O_3SCF_3]$^+$ 351.1236, found 351.1243.

5-Benzyl-5*H*-thianthren-5-ium tetrafluoroborate (25a)

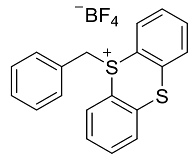

25a

A oven dried 25 mL screw capped vial was charged with thianthrene (4 mmol, 684 mg) , silver trifluoromethanesulfonate (4 mmol, 778.6 mg). The atmosphere of the vial was eliminated and filled with argon three times. 6 mL DCE was added. Finally (bromomethyl)benzene (5 mmol, 855 mg) was added dropwise. The mixture was stirred for 4 h at room temperature in dark. Then the mixture was filtered and reprecipitated from MeCN with diethylether, giving the **25a** as a white solid (978 mg, 62%): **^{1}H NMR (400 MHz, CD_3CN)** δ 7.95 (d, J = 7.9 Hz, 2H), 7.81 – 7.75 (m, 4H), 7.54 (t, J = 7.7 Hz, 2H), 7.38 (t, J = 7.4 Hz, 1H), 7.27 (t, J = 7.7 Hz, 2H), 7.08 (d, J = 7.4 Hz, 2H), 4.92 (s, 2H); **^{13}C NMR (101 MHz, CD_3CN)** δ 136.9, 135.7, 135.4, 131.4, 131.0, 130.3, 127.3, 117.2, 46.8; **^{11}B NMR (128 MHz, CD_3CN)** δ -1.1; **ATR-FTIR (cm^{-1}):** 2960, 2831, 1316, 1031, 903, 722; **HRMS m/z (ESI)** calculated for $C_{19}H_{15}S_2$ [M – BF_4]$^+$ 307.0610, found 307.0611.

5-(3,3,4,4,5,5,6,6,7,7,8,8,9,9,10,10,10-Heptadecafluorodecyl)-5*H*-thianthren-5-ium trifluoromethanesulfonate (26a)

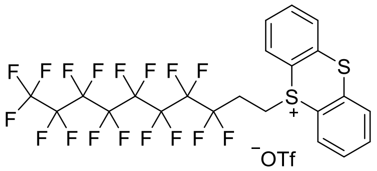

26a

According to the Method **A**, thianthrene (1.08g, 5.0 mmol), triflate (crude, 5.0 mmol) and DCE 10 mL. The mixture was stired at 68 °C for 48 h. The mixture was purified by precipitation with Et_2O/DCM to afford the product **26a** as a white solid (528 mg, 13%): **^{1}H NMR (400 MHz, $CDCl_3$)** δ 8.42 (d, *J* = 7.8 Hz, 2H), 7.88 (d, *J* = 7.9 Hz, 2H), 7.80 (t, *J* = 7.6 Hz,

2H), 7.72 (t, J = 8.2 Hz, 2H), 4.11 – 3.99 (m, 2H), 2.76 – 2.53 (m, 2H); **^{13}C NMR (101 MHz, $CDCl_3$)** δ 135.8, 135.2, 134.9, 130.3, 130.2, 120.6 (q, J = 319.8 Hz), 116.6, 26.5 (t, J = 22.4 Hz); **^{19}F NMR (376 MHz, $CDCl_3$)** δ -78.46 (s, 3F), -80.27 – -81.28 (m, 3F), -112.73 (s, 2F), -121.76 (s, 2F), -121.98 (s, 4F), -122.78 (s, 2F), -123.01 (s, 2F), -126.17 (s, 2F); **ATR-FTIR (cm^{-1}):** 1264, 731, 702, 638; **HRMS m/z (ESI)** calculated for $C_{22}H_{12}F_{17}S_2$ [M – O_3SCF_3]$^+$ 663.0103, found 663.0100.

5-((9*Z*,12*Z*,15*Z*)-octadeca-9,12,15-trien-1-yl)-5*H*-thianthren-5-ium trifluoromethanesulfonate (27a)

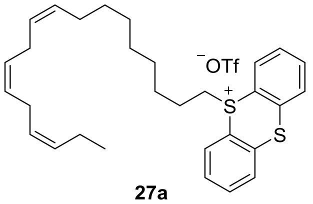

27a

According to the Method **A**, **27a** was prepared from the corresponding alcohol (5.0 mmol) at room temperature as a gray oil (766 mg, 25%): **^{1}H NMR (400 MHz, $CDCl_3$)** δ 8.31 (d, J = 7.8 Hz, 2H), 7.82 (dd, J = 7.9, 1.1 Hz, 2H), 7.78 – 7.72 (m, 2H), 7.66 (td, J = 7.7, 1.2 Hz, 2H), 5.39 – 5.27 (m, 6H), 3.80 – 3.72 (m, 2H), 2.76 (m, 4H), 2.02 (m, 4H), 1.53 (p, J = 7.6 Hz, 2H), 1.28 (m, 10H), 0.95 (t, J = 7.5 Hz, 3H); **^{13}C NMR (101 MHz, $CDCl_3$)** δ 135.6, 134.9, 134.4, 132.0, 130.2, 130.0, 129.9, 128.3, 128.2, 127.7, 127.1, 117.5, 40.4, 29.5, 29.0, 28.8, 27.9, 27.2, 25.6, 25.5, 24.5, 20.6, 14.3; **^{19}F NMR (376 MHz, $CDCl_3$)** δ -78.2; **ATR-FTIR (cm^{-1}):** 3008, 2926, 2854, 1569, 1451, 1257, 1154, 1029, 636; **HRMS m/z (ESI)** calculated for $C_{30}H_{39}S_2$ [M – O_3SCF_3]$^+$ 463.2488, found 575.3371.

The carbon NMR signal of triflate anion was not observed.

(*E*)-5-(2-(4-(4-chloro-1,2-diphenylbut-1-en-1-yl)phenoxy)ethyl)-5*H*-thianthren-5-ium trifluoromethanesulfonate (28a)

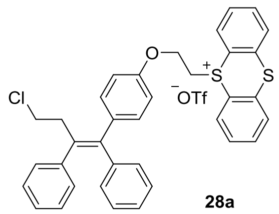

28a

According to the Method **A**, **28a** was prepared from the corresponding alcohol (5.0 mmol) at room temperature as a white solid (1.93 g, 53%): **^{1}H NMR (400 MHz, CD_3CN)** δ 8.01 (d, J = 7.9 Hz, 2H), 7.92 (d, J = 8.0 Hz, 2H), 7.77 (t, J = 7.7 Hz, 2H), 7.58 (t, J = 8.2 Hz, 2H), 7.45 – 7.39 (m, 2H), 7.33 (t, J = 6.7 Hz, 3H), 7.27 – 7.17 (m, 5H), 6.85 (d, J = 8.7 Hz, 2H), 6.45 (d, J = 8.6 Hz, 2H), 4.04 (s, 4H), 3.42 (t, J = 7.3 Hz,

2H), 2.85 (t, J = 7.3 Hz, 2H); **^{13}C NMR (101 MHz, CD_3CN)** δ 156.3, 143.8, 142.4, 141.8, 137.5, 137.3, 135.6, 135.2, 132.4, 131.3, 130.6, 130.5, 130.1, 129.5, 129.1, 128.0, 127.7, 114.4, 62.8, 43.7, 42.1, 39.2; **^{19}F NMR (376 MHz, CD_3CN)** δ -79.3; **ATR-FTIR (cm^{-1}):** 3055, 2921, 1507, 1263, 1154, 1029, 732, 637; **HRMS m/z (ESI)** calculated for $C_{36}H_{30}ClOS_2$ $[M - O_3SCF_3]^+$ 577.1421, found 557.1423.

The carbon NMR signal of triflate anion was not observed.

5-(4-((3*R*,5*R*,8*R*,9*S*,10*S*,13*R*,14*S*,17*R*)-3-methoxy-10,13-dimethylhexadecahydro-1*H*-cyclopenta[*a*]phenanthren-17-yl)pentyl)-5*H*-thianthren-5-ium trifluoromethanesulfonate (29a)

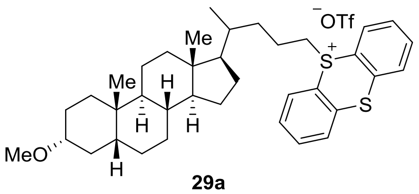

29a

According to the Method **A**, **29a** was prepared from the corresponding alcohol (5.0 mmol) as a white solid (1.59 g, 44%): **^{1}H NMR (400 MHz, CD_3CN)** δ 8.09 (d, J = 7.9 Hz, 2H), 7.94 (d, J = 8.0 Hz, 2H), 7.82 (t, J = 7.7 Hz, 2H), 7.70 (t, J = 7.7 Hz, 2H), 3.67 (ddd, J = 12.1, 9.0, 5.6 Hz, 1H), 3.56 (ddd, J = 12.0, 8.9, 5.8 Hz, 1H), 3.25 (s, 3H), 3.15 – 3.05 (m, 1H), 1.92 – 1.81 (m, 2H), 1.80 – 1.65 (m, 3H), 1.59 – 1.50 (m, 3H), 1.44 – 1.28 (m, 8H), 1.27 – 0.92 (m, 12H), 0.90 (s, 3H), 0.79 (d, J = 6.5 Hz, 3H), 0.60 (s, 3H); **^{13}C NMR (101 MHz, CD_3CN)** δ 136.9, 135.6, 135.1, 131.3, 130.6, 118.3, 81.1, 57.2, 56.7, 55.6, 43.5, 42.9, 41.9, 41.3, 40.9, 36.7, 36.2, 36.0, 35.6, 34.6, 33.7, 28.9, 28.1, 27.7, 27.2, 24.9, 23.9, 22.1, 21.6, 18.7, 12.4; **^{19}F NMR (376 MHz, CD_3CN)** δ -79.2; **ATR-FTIR (cm^{-1}):** 2929, 2864, 1261, 1153, 1029, 732, 636; **HRMS m/z (ESI)** calculated for $C_{37}H_{51}OS_2$ $[M - O_3SCF_3]^+$ 575.3376, found 575.3376.

The carbon NMR signal of triflate anion was not observed.

5-(10-(4,5-Dimethoxy-2-methyl-3,6-dioxocyclohexa-1,4-dien-1-yl)decyl)-5*H*-thianthren-5-ium trifluoromethanesulfonate (30a)

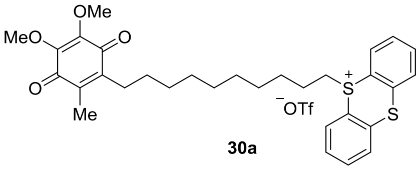

30a

According to the Method **A**, **30a** was prepared from the corresponding alcohol (5.0 mmol) as a brown oil (2.09 g, 61%): **^{1}H NMR (400 MHz,**

CD_3CN) δ 8.56 (dd, J = 7.9, 1.2 Hz, 2H), 8.39 (dd, J = 8.0, 1.1 Hz, 2H), 8.27 (td, J = 7.7, 1.4 Hz, 2H), 8.19 – 8.13 (m, 2H), 4.36 (s, 3H), 4.36 (s, 3H), 4.13 – 4.07 (m, 2H), 2.88 – 2.83 (m, 2H), 2.40 (s, 3H), 1.87 – 1.55 (m, 16H); **^{13}C NMR (101 MHz, CD_3CN)** δ 185.6, 185.1, 145.6, 143.5, 139.6, 136.9, 135.6, 135.2, 131.2, 130.6, 117.9, 61.6, 41.4, 30.3, 29.9, 29.6, 29.2, 28.1, 26.8, 25.0, 12.1; **^{19}F NMR (376 MHz, CD_3CN)** δ -79.1; **ATR-FTIR (cm^{-1}):** 2927, 2854, 1644，1609，1256，1028，635; **HRMS m/z (ESI)** calculated for $C_{31}H_{37}O_4S_2$ [M – O_3SCF_3]$^+$ 537.2128, found 537.2124.

The carbon NMR signal of triflate anion was not observed.

5,5'-(Hexane-1,6-diyl)bis(5*H*-thianthren-5-ium) trifluoromethanesulfonate (31a)

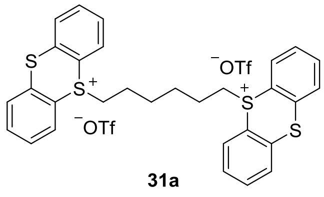

31a

According to the Method **A**, **29a** was prepared from the corresponding alcohol (5.0 mmol) with thianthrene (1.08g, 5.0 mmol) as a white solid (998 mg, 49%): **^1H NMR (400 MHz, CD_3CN)** δ 8.09 (dd, J = 7.9, 1.2 Hz, 4H), 7.90 (dd, J = 8.0, 1.1 Hz, 4H), 7.80 (td, J = 7.7, 1.3 Hz, 4H), 7.67 (td, J = 7.8, 1.3 Hz, 4H), 3.63 – 3.54 (m, 4H), 1.53 – 1.37 (m, 4H), 1.27 (m, 4H); **^{13}C NMR (101 MHz, CD_3CN)** δ 136.8, 135.6, 135.2, 131.2, 130.6, 122.1 (q, J = 324.6 Hz), 117.8, 41.1, 27.3, 24.6; **^{19}F NMR (376 MHz, CD_3CN)** δ -79.1; **ATR-FTIR (cm^{-1}):** 1451, 1258, 1153, 1028, 636; **HRMS m/z (ESI)** calculated for $C_{30}H_{28}S_4$ [M – $O_6S_2C_2F_6$]$^{2+}$ 258.0531, found 258.0536.

5-(4-Iodophenethyl)-5*H*-thianthren-5-ium trifluoromethanesulfonate (32a)

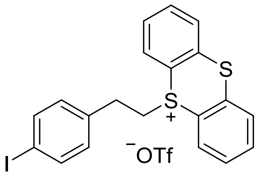

32a

According to the Method **A**, **32a** was prepared from the corresponding alcohol (5.0 mmol) as a white solid (1.49 g, 50%): **^1H NMR (400 MHz, $CDCl_3$)** δ 8.11 (dd, J = 7.9, 1.2 Hz, 2H), 7.80 (dd, J = 8.0, 1.2 Hz, 2H), 7.72 (td, J = 7.7, 1.3 Hz, 2H), 7.58 (td, J = 7.8, 1.3 Hz, 2H), 7.44 (d, J = 8.4 Hz, 2H), 6.87 (d, J = 8.4 Hz, 2H), 4.05 – 3.94 (m, 2H), 2.94 (t, J = 7.7 Hz, 2H); **^{13}C NMR (101 MHz, $CDCl_3$)** δ 137.8, 135.7, 134.8, 134.4, 134.4, 130.7, 130.1, 129.8, 120.7 (q, J = 320.7 Hz), 117.2, 93.1, 41.2, 30.0; **^{19}F NMR (376 MHz, $CDCl_3$)** δ -78.0; **ATR-FTIR (cm^{-1}):** 1449,

1250, 1150, 1027, 756, 635; **HRMS m/z (ESI)** calculated for $C_{20}H_{16}IS_2$ [M – $O_3SCF_3]^+$ 446.9733, found 446.9726.

5-(2-Vinylphenethyl)-5*H*-thianthren-5-ium trifluoromethanesulfonate (38)

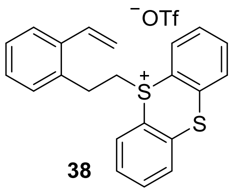

38

According to the Method **A**, **38** was prepared from the corresponding alcohol (5.0 mmol) at room temperature as a gray solid (794 mg, 16%): **^{1}H NMR (400 MHz, CDCl$_3$)** δ 8.17 (d, J = 7.9 Hz, 2H), 7.79 (d, J = 7.9 Hz, 2H), 7.71 (t, J = 8.1 Hz, 2H), 7.58 (t, J = 8.1 Hz, 2H), 7.23 (dt, J = 5.7, 3.1 Hz, 2H), 7.16 (dd, J = 5.6, 3.4 Hz, 2H), 6.43 (dd, J = 17.2, 10.9 Hz, 1H), 5.37 (d, J = 17.2 Hz, 1H), 5.16 (d, J = 10.9 Hz, 1H), 3.98 – 3.92 (m, 2H), 3.16 – 3.10 (m, 2H); **^{13}C NMR (101 MHz, CDCl$_3$)** δ 136.8, 135.7, 134.6, 134.2, 133.0, 132.7, 130.4, 129.8, 128.5, 128.2, 126.5, 120.8 (q, J = 320.5 Hz), 117.7, 41.1, 28.1; **^{19}F NMR (376 MHz, CDCl$_3$)** δ -78.0; **ATR-FTIR (cm^{-1}):** 3074, 2929, 1450, 1256, 1157, 1029, 759, 636; **HRMS m/z (ESI)** calculated for $C_{22}H_{19}S_2$ [M – $O_3SCF_3]^+$ 347.0923, found 347.0927.

Experimental Procedures and Characterization of Products

Supplementary Table 1. Optimization studies of photoinduced desulfurative borylation. [a]

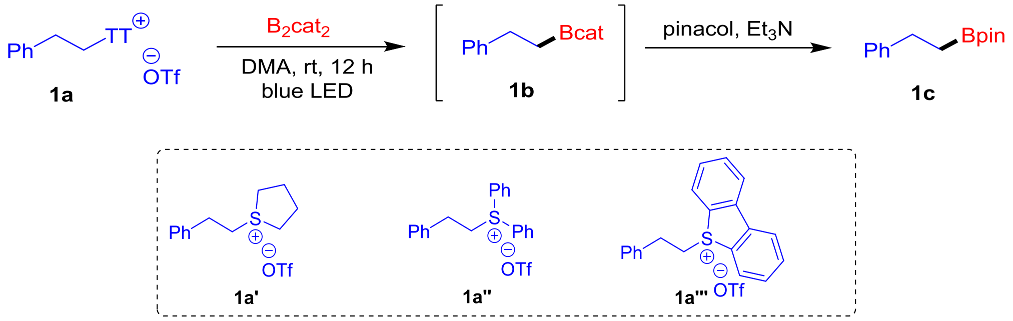

Entry	Variation from the standard conditions	Yield of **1c** (%)[b]
1	None	93 (82)[c]
2	Using **1a'** instead of **1a**	<5
3	Using **1a''** instead of **1a**	32 (18) [c]
4	Using **1a'''** instead of **1a**	70 (59) [c]
5	0.5 equiv. B_2cat_2	46%
6	1.0 equiv. B_2cat_2	85%
7	1.5 equiv. B_2cat_2	88%

8	Using Cl-Cat instead of B_2cat_2	nd
9	Using B_2pin_2 instead of B_2cat_2	nd
10	Using DMF instead of DMA	90 (80) [c]
11	Using DCM instead of DMA	<5
12	At 60 °C	15
13	In the dark	<5
14	Purple LED (390 nm, 40W) instead of blue LED	93 (82)
15	Red light (620-625 nm, 40 W) instead of blue LED	31

[a]Standard conditions: **1a** (0.40 mmol), B_2Cat_2 (0.80 mmol) in 1.0 mL DMA at RT, 12 hours, under Ar. [b]Determined by ^{1}HNMR analysis of the crude reaction mixture. [c] Yield of isolated **1c**.

Supplementary Table 2. Optimization studies of thermoinduced desulfurative borylation by Lewis base.[a]

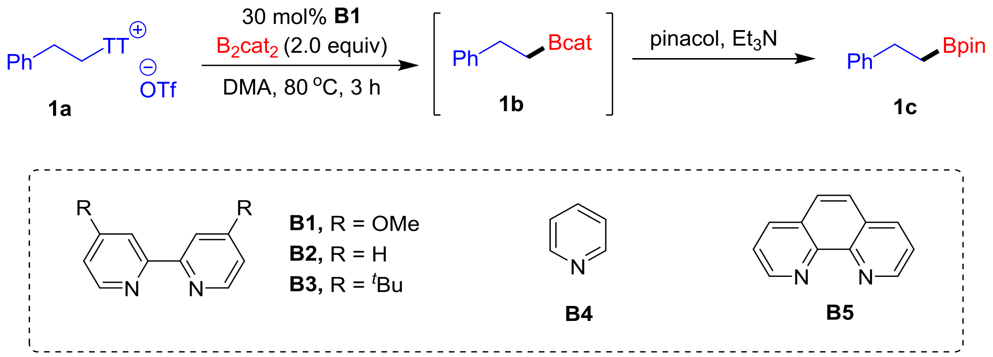

Entry	Variation from the standard conditions	Yield of **1c** (%)[b]
1	None	86 (75)[c]
2	without **B1**	<5
3	10 mol% **B1**	55
4	**B2** instead of **B1**	68 (59) [c]
5	**B3** instead of **B1**	81 (70) [c]
6	**B4** instead of **B1**	<5
7	**B5** instead of **B1**	<5
8	At room temperature	50
9	In the dark	85 (73) [c]

[a]Standard conditions: **1a** (0.40 mmol), B_2Cat_2 (0.80 mmol) in 1.0 mL DMA at 80 °C, 3 hours, under Ar. [b]Determined by ^{1}HNMR analysis of the crude reaction mixture. [c] Yield of isolated **1c**.

General Procedure I:

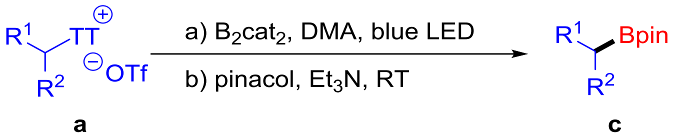

Supplementary Figure 4. Photoinduced desulfurative borylation. General procedures for photoinduced desulfurative borylation.

A 10.0 mL Schlenk tube with a stirring bar was added with alkyl sulfonium (0.4 mmol, 1.0 equiv), B_2cat_2 (190.3 mg, 0.8 mmol, 2.0 equiv) and DMA (1.0 mL) under argon. The resulting mixture was stirred at room temperature under blue LED irradiation for 12 h. Then pinacol (189.1 mg, 1.6 mmol, 4.0 equiv) was dissolved in Et_3N (1.0 mL), added to the reaction mixture and stirred for 1 h. The mixture was cooled to 0 ^{o}C to precipitate thianthrene, which was removed by filtration. Then water was added, and the reaction mixture was extracted with EtOAc, dried over $MgSO_4$ and concentrated under reduced pressure. The crude product was purified by flash column chromatography.

General Procedure II:

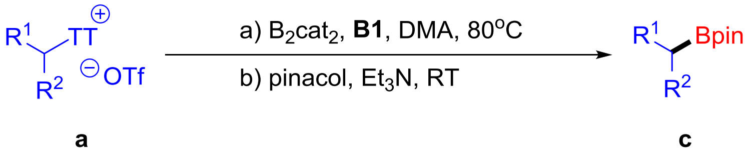

Supplementary Figure 5. Thermoinduced desulfurative borylation by Lewis base. General procedures for thermoinduced desulfurative borylation by Lewis base.

A 10.0 mL Schlenk tube with a stirring bar was added with alkyl sulfonium (0.4 mmol, 1.0 equiv), B_2cat_2 (190.3 mg, 0.8 mmol, 2.0 equiv), dMeObpy (26.0 mg, 30.0 mol %) and DMA (1.0 mL) under argon. The resulting mixture was stirred at 80 ^{o}C under Ar for 3 h. The mixture was cooled to room temperature. Pinacol (189.1 mg, 1.6 mmol, 4.0 equiv) was dissolved in Et_3N (1.0 mL), added to the reaction mixture and stirred for 1 h. The mixture was cooled to 0 ^{o}C to precipitate thianthrene, which was

removed by filtration. Then water was added, and the reaction mixture was extracted with EtOAc, dried over $MgSO_4$ and concentrated under reduced pressure. The crude product was purified by flash column chromatography.

4,4,5,5-Tetramethyl-2-phenethyl-1,3,2-dioxaborolane (1c)

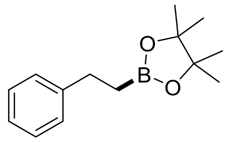

1c

According to General Procedure I, the reaction was carried out with **1a** (188.2 mg, 0.4 mmol) and B_2cat_2 (190.3 mg, 0.8 mmol) in DMA (1.0 mL). The crude product was purified by flash column chromatography on silica gel (toluene/PE = 1:1, 100 mL, then EA/PE = 30:1) to afford 76.1 mg (82%) of **1c** as a colourless oil. According to General Procedure II, afford 69.6 mg (75%) of **1c**: **^{1}H NMR (400 MHz, $CDCl_3$)** δ 7.27 – 7.19 (m, 4H), 7.18 – 7.08 (m, 1H), 2.79 – 2.69 (m, 2H), 1.21 (s, 12H), 1.18 – 1.10 (m, 2H); **^{13}C NMR (101 MHz, $CDCl_3$)** δ 144.4, 128.1, 128.0, 125.4, 83.0, 29.9, 24.8, 21.8 (br.); **^{11}B NMR (128 MHz, $CDCl_3$)** δ 33.6; **ATR-FTIR (cm $^{-1}$):** 3026, 2977, 2930, 1369, 1319, 1142, 697; **HRMS m/z (ESI)** calcd for $C_{14}H_{22}BO_2$ $(M + H)^+$ 233.1707, found 233.1707. All spectral data are in accordance with the literature.[4]

4,4,5,5-Tetramethyl-2-octadecyl-1,3,2-dioxaborolane (2c)

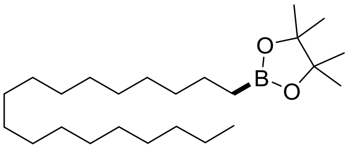

2c

According to General Procedure I, the reaction was carried out with **2a** (247.6 mg, 0.4 mmol) and B_2cat_2 (190.3 mg, 0.8 mmol) in DMA (1.0 mL). The crude product was purified by flash column chromatography on silica gel (toluene/PE = 1:1, 100 mL, then EA/PE = 30:1) to afford 115.7 mg (76%) of **2c** as a colourless oil. According to General Procedure II, afford 85.2 mg (56%) of **2c**: **^{1}H NMR (400 MHz, $CDCl_3$)** δ 1.37 (q, J = 7.2 Hz, 3H), 1.29 – 1.17 (m, 41H), 0.86 (t, J = 6.8 Hz, 3H), 0.74 (t, J = 7.7 Hz, 2H); **^{13}C NMR (101 MHz, $CDCl_3$)** δ 82.7, 32.4, 31.9, 29.7, 29.7, 29.7, 29.7, 29.6, 29.4, 29.4, 24.8, 24.0, 22.7. 11.1 (br.); **^{11}B NMR (128 MHz, $CDCl_3$)** δ 34.2; **ATR-FTIR (cm $^{-1}$):** 2922, 2852, 1371, 1144, 906; **HRMS m/z (ESI)** calcd for $C_{24}H_{50}BO_2$ $(M + H)^+$ 381.3898, found 381.3898.

2-(2-((3*r*,5*r*,7*r*)-Adamantan-1-yl)ethyl)-4,4,5,5-tetramethyl-1,3,2-dioxaborolane (3c)

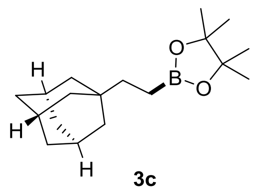

3c

According to General Procedure I, the reaction was carried out with **3a** (211.5 mg, 0.4 mmol) and B_2cat_2 (190.3 mg, 0.8 mmol) in DMA (1.0 mL). The crude product was purified by flash column chromatography on silica gel (toluene/PE = 1:1, 100 mL, then EA/PE = 30:1) to afford 98.7 mg (85%) of **3c** as a white solid. According to General Procedure II, afford 89.4 mg (77%) of **3c**: **^{1}H NMR (500 MHz, CDCl$_3$)** δ 1.91 (s, 3H), 1.66 (d, *J* = 12.0 Hz, 3H), 1.58 (d, *J* = 11.4 Hz, 3H), 1.41 (d, *J* = 2.3 Hz, 6H), 1.22 (s, 12H), 1.18 – 1.12 (m, 2H), 0.72 – 0.59 (m, 2H); **^{13}C NMR (126 MHz, CDCl$_3$)** δ 82.8, 41.9, 38.0, 37.3, 32.5, 28.7, 24.8, 3.4 (br.); **^{11}B NMR (160 MHz, CDCl$_3$)** δ 34.5; **ATR-FTIR (cm $^{-1}$):** 2900, 2845, 1371, 1145, 905, 729; **HRMS m/z (ESI)** calcd for $C_{18}H_{32}BO_2$ $(M + H)^+$ 291.2490, found 291.2490.

2-(3-(4-Isopropylphenyl)-2-methylpropyl)-4,4,5,5-tetramethyl-1,3,2-dioxaborolane (4c)

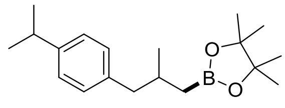

4c

According to General Procedure I, the reaction was carried out with **4a** (198.2 mg, 0.4 mmol) and B_2cat_2 (190.3 mg, 0.8 mmol) in DMA (1.0 mL). The crude product was purified by flash column chromatography on silica gel (toluene/PE = 1:1, 100 mL, then EA/PE = 20:1) to afford 102.8 mg (85%) of **4c** as a colorless oil. According to General Procedure II, afford 64.1 mg (53%) of **4c**: **^{1}H NMR (400 MHz, CDCl$_3$)** δ 7.14 (d, *J* = 8.1 Hz, 2H), 7.10 (d, *J* = 8.1 Hz, 2H), 2.89 (p, *J* = 6.9 Hz, 1H), 2.59 (dd, *J* = 13.2, 6.4 Hz, 1H), 2.42 (dd, *J* = 13.2, 7.8 Hz, 1H), 2.01 (dq, *J* = 14.2, 6.7 Hz, 1H), 1.26 (t, *J* = 3.5 Hz, 18H), 0.91 (dd, *J* = 17.3, 6.2 Hz, 4H), 0.70 (dd, *J* = 15.4, 8.6 Hz, 1H); **^{13}C NMR (101 MHz, CDCl$_3$)** δ 146.0, 138.9, 129.2, 126.0, 82.8, 45.6, 33.6, 31.7, 24.9, 24.8, 24.1, 22.1, 19.5 (br.); **^{11}B NMR (128 MHz, CDCl$_3$)** δ 34.0; **ATR-FTIR (cm $^{-1}$):** 2958, 2724, 1367, 1312, 1142, 847; **HRMS m/z (ESI)** calcd for $C_{19}H_{32}BO_2$ $(M + H)^+$ 303.2490, found 303.2488.

2-(3-Methoxypropyl)-4,4,5,5-tetramethyl-1,3,2-dioxaborolane (5c)

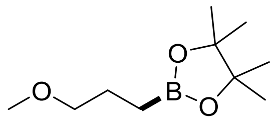

5c

According to General Procedure I, the reaction was carried out with **5a** (175.4 mg, 0.4 mmol) and B_2cat_2 (190.3 mg, 0.8 mmol) in DMA (1.0 mL). The crude product was purified by flash column chromatography on silica gel (toluene/PE = 1:1, 100 mL, then EA/PE = 20:1) to afford 52.8 mg (66%) of **5c** as a colorless oil. According to General Procedure II, afford 38.4 mg (48%) of **5c**: **^{1}H NMR (500 MHz, $CDCl_3$)** δ 3.31 (t, J = 6.7 Hz, 2H), 3.28 (s, 3H), 1.65 (p, J = 7.1 Hz, 2H), 1.21 (s, 12H), 0.75 (t, J = 7.7 Hz, 2H); **^{13}C NMR (126 MHz, $CDCl_3$)** δ 82.9, 74.5, 58.3, 24.7, 23.9; **^{11}B NMR (160 MHz, $CDCl_3$)** δ 34.0; **ATR-FTIR (cm^{-1}):** 2977, 2928, 1369, 1315, 1116; **HRMS m/z (ESI)** calcd for $C_{10}H_{22}BO_3$ $(M + H)^+$ 201.1657, found 201.1657. All spectral data are in accordance with the literature.[5]

^{13}C NMR C–B not detected due to quadropolar relaxation.

Ethyl 6-(4,4,5,5-tetramethyl-1,3,2-dioxaborolan-2-yl)hexanoate (6c)

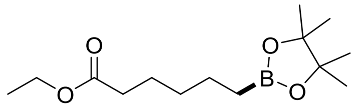

6c

According to General Procedure I, the reaction was carried out with **6a** (203.4 mg, 0.4 mmol) and B_2cat_2 (190.3 mg, 0.8 mmol) in DMA (1.0 mL). The crude product was purified by flash column chromatography on silica gel (toluene/PE = 1:1, 100 mL, then EA/PE = 20:1) to afford 75.6 mg (70%) of **6c** as a colorless oil. According to General Procedure II, afford 70.2 mg (65%) of **6c**: **^{1}H NMR (400 MHz, $CDCl_3$)** δ 4.06 (q, J = 7.1 Hz, 2H), 2.22 (t, J = 7.6 Hz, 2H), 1.56 (p, J = 7.5 Hz, 2H), 1.40 – 1.33 (m, 2H), 1.30 – 1.24 (m, 2H), 1.20 (s, 3H), 1.18 (s, 12H), 0.71 (t, J = 7.7 Hz, 2H); **^{13}C NMR (101 MHz, $CDCl_3$)** δ 173.7, 82.8, 60.0, 34.2, 31.7, 24.9, 24.8, 24.7, 23.5, 14.1; **^{11}B NMR (128 MHz, $CDCl_3$)** δ 33.6; **ATR-FTIR (cm^{-1}):** 2978, 2931, 1734, 1370, 1141, 967; **HRMS m/z (ESI)** calcd for $C_{14}H_{28}BO_4$ $(M + H)^+$ 271.2075, found 271.2071.

^{13}C NMR C–B not detected due to quadropolar relaxation.

4-(2-(4,4,5,5-Tetramethyl-1,3,2-dioxaborolan-2-yl)ethyl)benzonitrile (7c)

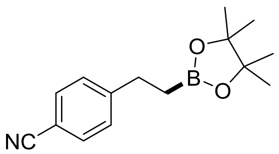

7c

According to General Procedure I, the reaction was carried out with **7a** (198.2 mg, 0.4 mmol) and B_2cat_2 (190.3 mg, 0.8 mmol) in DMA (1.0 mL). The crude product was purified by flash column chromatography on silica gel (toluene/PE = 1:1, 100 mL, then EA/PE = 10:1) to afford 72.0 mg (70%) of **7c** as a white solid. According to General Procedure II, afford 68.9 mg (67%) of **7c**: **^{1}H NMR (500 MHz, CDCl$_3$)** δ 7.52 (d, J = 8.2 Hz, 2H), 7.29 (d, J = 8.2 Hz, 2H), 2.77 (t, J = 8.0 Hz, 2H), 1.18 (s, 12H), 1.14 – 1.09 (m, 2H); **^{13}C NMR (126 MHz, CDCl$_3$)** δ 150.0, 132.0, 128.8, 119.1, 109.3, 83.2, 30.1, 24.7, 12.3 (br.); **^{11}B NMR (128 MHz, CDCl$_3$)** δ 33.6; **ATR-FTIR (cm^{-1}):** 2978, 2924, 2226, 1605, 1370, 1322, 1141, 546; **HRMS m/z (ESI)** calcd for $C_{15}H_{21}BNO_2$ $(M + H)^+$ 258.1660, found 258.1658. All spectral data are in accordance with the literature.[4]

2-(4-Fluorophenethyl)-4,4,5,5-tetramethyl-1,3,2-dioxaborolane (8c)

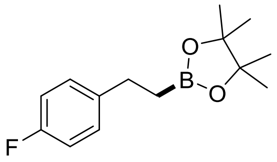

8c

According to General Procedure I, the reaction was carried out with **8a** (195.4 mg, 0.4 mmol) and B_2cat_2 (190.3 mg, 0.8 mmol) in DMA (1.0 mL). The crude product was purified by flash column chromatography on silica gel (toluene/PE = 1:1, 100 mL, then EA/PE = 30:1) to afford 83.0 mg (83%) of **8c** as a colourless oil. According to General Procedure II, afford 72.0 mg (72%) of **8c**: **^{1}H NMR (400 MHz, CDCl$_3$)** δ 7.15 (dd, J = 8.6, 5.5 Hz, 2H), 6.93 (t, J = 8.8 Hz, 2H), 2.71 (t, J = 8.1 Hz, 2H), 1.20 (s, 12H), 1.14 – 1.08 (m, 2H); **^{13}C NMR (101 MHz, CDCl$_3$)** δ 161.0 (d, J = 242.7 Hz), 139.9 (d, J = 3.1 Hz), 129.2 (d, J = 7.7 Hz), 114.7 (d, J = 21.0 Hz), 83.1, 29.1, 24.7; **^{19}F NMR (376 MHz, CDCl$_3$)** δ -118.3; **^{11}B NMR (128 MHz, CDCl$_3$)** δ 33.8; **ATR-FTIR (cm^{-1}):** 2978, 2931, 1508, 1370, 1316, 1142, 830; **HRMS m/z (ESI)** calcd for $C_{14}H_{21}BFO_2$ $(M + H)^+$ 251.1613, found 251.1610. All spectral data are in accordance with the literature.[6]

^{13}C NMR C–B not detected due to quadropolar relaxation.

2-(4-Chlorobutyl)-4,4,5,5-tetramethyl-1,3,2-dioxaborolane (9c)

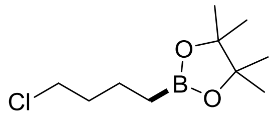

9c

According to General Procedure I, the reaction was carried out with **9a** (182.8 mg, 0.4 mmol) and B_2cat_2 (190.3 mg, 0.8 mmol) in DMA (1.0 mL). The crude product was purified by flash column chromatography on silica gel (toluene/PE = 1:1, 100 mL, then EA/PE = 20:1) to afford 73.4 mg (84%) of **9c** as a colorless oil. According to General Procedure II, afford 53.3 mg (61%) of **9c**: **^{1}H NMR (400 MHz, CDCl$_3$)** δ 3.49 (t, J = 6.8 Hz, 2H), 1.78 – 1.72 (m, 2H), 1.51 (p, J = 7.7 Hz, 2H), 1.21 (s, 12H), 0.76 (t, J = 7.9 Hz, 2H); **^{13}C NMR (126 MHz, CDCl$_3$)** δ 82.9, 44.8, 35.0, 24.7, 21.3; **^{11}B NMR (128 MHz, CDCl$_3$)** δ 33.8; **ATR-FTIR (cm $^{-1}$):** 2935, 1408, 1371, 1321, 1143, 729; **HRMS m/z (ESI)** calcd for $C_{10}H_{21}BClO_2$ $(M + H)^+$ 219.1318, found 291.1316. All spectral data are in accordance with the literature.[7]

^{13}C NMR C–B not detected due to quadropolar relaxation.

2-(4-Bromophenethyl)-4,4,5,5-tetramethyl-1,3,2-dioxaborolane (10c)

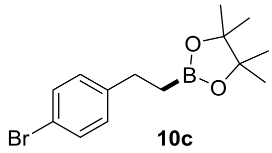

10c

According to General Procedure I, the reaction was carried out with **10a** (219.8 mg, 0.4 mmol) and B_2cat_2 (190.3 mg, 0.8 mmol) in DMA (1.0 mL). The crude product was purified by flash column chromatography on silica gel (toluene/PE = 1:1, 100 mL, then EA/PE = 20:1) to afford 90.8 mg (73%) of **10c** as a white solid. According to General Procedure II, afford 104.5 mg (84%) of **10c**: **^{1}H NMR (400 MHz, CDCl$_3$)** δ 7.36 (d, J = 8.0, 2H), 7.08 (d, J = 8.0 Hz, 2H), 2.69 (t, J = 8.1 Hz, 2H), 1.20 (s, 12H), 1.11 (t, J = 8.1 Hz, 2H); **^{13}C NMR (101 MHz, CDCl$_3$)** δ 143.3, 131.1, 129.7, 119.1, 83.1, 29.3, 24.7; **^{11}B NMR (128 MHz, CDCl$_3$)** δ 33.9; **ATR-FTIR (cm $^{-1}$):** 2977, 2930, 1370, 1315, 1142; **HRMS m/z (ESI)** calcd for $C_{14}H_{21}BBrO_2$ $(M + H)^+$ 311.0812, found 311.0812. All spectral data are in accordance with the literature.[8]

^{13}C NMR C–B not detected due to quadropolar relaxation.

2-(3-Bromopropyl)-4,4,5,5-tetramethyl-1,3,2-dioxaborolane (11c)

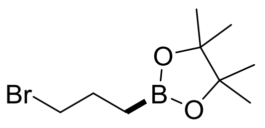

11c

According to General Procedure I, the reaction was carried out with **11a** (194.9 mg, 0.4 mmol) and B_2cat_2 (190.3

mg, 0.8 mmol) in DMA (1.0 mL). The crude product was purified by flash column chromatography on silica gel (toluene/PE = 1:1, 100 mL, then EA/PE = 20:1) to afford 65.7 mg (66%) of **11c** as a colorless oil. According to General Procedure II, afford 53.8 mg (54%) of **11c**: **^{1}H NMR (400 MHz, CDCl$_3$)** δ 3.40 (t, J = 6.9 Hz, 2H), 1.94 (p, J = 7.0 Hz, 2H), 1.22 (s, 12H), 0.90 (t, J = 7.8 Hz, 2H); **^{13}C NMR (101 MHz, CDCl$_3$)** δ 83.1, 36.2, 27.5, 24.8; **^{11}B NMR (128 MHz, CDCl$_3$)** δ 33.5; **ATR-FTIR (cm $^{-1}$):** 2977, 2932, 1370, 1315, 1142, 968; **HRMS m/z (ESI)** calcd for $C_9H_{19}BBrO_2$ $(M + H)^+$ 249.0656, found 249.0656. All spectral data are in accordance with the literature.[9]

^{13}C NMR C–B not detected due to quadropolar relaxation.

2-(3-Iodopropyl)-4,4,5,5-tetramethyl-1,3,2-dioxaborolane (12c)

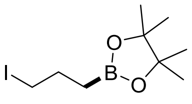

12c

According to General Procedure I, the reaction was carried out with **12a** (213.8 mg, 0.4 mmol) and B_2cat_2 (190.3 mg, 0.8 mmol) in DMA (1.0 mL). The crude product was purified by flash column chromatography on silica gel (toluene/PE = 1:1, 100 mL, then EA/PE = 20:1) to afford 46.2 mg (39%) of **12c** as a colorless oil. According to General Procedure II, afford 48.5 mg (41%) of **12c** a colorless oil: **^{1}H NMR (400 MHz, CDCl$_3$)** δ 3.20 (t, J = 7.1 Hz, 2H), 1.98 – 1.87 (m, 2H), 1.23 (s, 12H), 0.89 – 0.81 (m, 2H); **^{13}C NMR (101 MHz, CDCl$_3$)** δ 83.1, 28.3, 24.8, 10.0; **^{11}B NMR (128 MHz, CDCl$_3$)** δ 33.3; **ATR-FTIR (cm $^{-1}$):** 2976, 2929, 1369, 1312, 1141, 846; **HRMS m/z (ESI)** calcd for $C_9H_{19}BIO_2$ $(M + H)^+$ 297.0517, found 297.0519. All spectral data are in accordance with the literature.[15]

^{13}C NMR C–B not detected due to quadropolar relaxation.

4,4,5,5-Tetramethyl-2-(2-(thiophen-2-yl)ethyl)-1,3,2-dioxaborolane (13c)

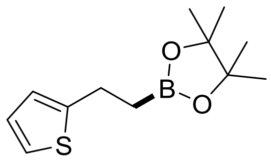

13c

According to General Procedure I, the reaction was carried out with **13a** (190.6 mg, 0.4 mmol) and B_2cat_2 (190.3 mg, 0.8 mmol) in DMA (1.0 mL). The crude product was purified by flash column chromatography on silica gel (toluene/PE = 1:1, 100 mL, then EA/PE = 20:1) to afford 69.7 mg (73%) of

13c as a white solid. According to General Procedure II, afford 69.6 mg (73%) of **13c**: **^{1}H NMR (400 MHz, CDCl$_3$)** δ 7.08 (d, J = 5.1 Hz, 1H), 6.89 (dd, J = 5.0, 3.5 Hz, 1H), 6.80 (d, J = 3.0 Hz, 1H), 2.99 – 2.93 (m, 2H), 1.23 (s, 14H); **^{13}C NMR (101 MHz, CDCl$_3$)** δ 161.1, 154.6, 129.0, 147.7, 126.5, 123.4, 122.5, 83.2, 24.7, 24.3, 13.5 (br.); **^{11}B NMR (128 MHz, CDCl$_3$)** δ 33.9; **ATR-FTIR (cm $^{-1}$):** 2977, 2928, 1370, 1314, 1142, 689; **HRMS m/z (ESI)** calcd for $C_{12}H_{20}BO_2S$ $(M + H)^+$ 239.1272, found 239,1270. All spectral data are in accordance with the literature.[4]

2-(2-(Benzofuran-2-yl)ethyl)-4,4,5,5-tetramethyl-1,3,2-dioxaborolane (14c)

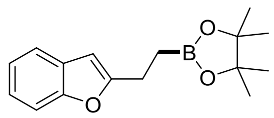

14c

According to General Procedure I, the reaction was carried out with **14a** (204.2 mg, 0.4 mmol) and B_2cat_2 (190.3 mg, 0.8 mmol) in DMA (1.0 mL). The crude product was purified by flash column chromatography on silica gel (toluene/PE = 1:1, 100 mL, then EA/PE = 20:1) to afford 89.3 mg (82%) of **14c** as a white solid. According to General Procedure II, afford 77.3 mg (71%) of **14c**: **^{1}H NMR (400 MHz, CDCl$_3$)** δ 7.49 (dd, J = 6.5, 2.2 Hz, 1H), 7.41 (d, J = 7.5 Hz, 1H), 7.23 – 7.16 (m, 2H), 6.39 (s, 1H), 2.93 (t, J = 7.9 Hz, 2H), 1.26 (s, 12H); **^{13}C NMR (101 MHz, CDCl$_3$)** δ 122.9, 122.2, 120.1, 110.6, 101.0, 83.2, 24.7, 22.9, 9.0 (br.); **^{11}B NMR (128 MHz, CDCl$_3$)** δ 33.6; **ATR-FTIR (cm $^{-1}$):** 2977, 2929, 1371, 1321, 1141, 738; **HRMS m/z (ESI)** calcd for $C_{16}H_{22}BO_3$ $(M + H)^+$ 273.1657, found 273.1653.

5-Methyl-2-phenyl-4-(2-(4,4,5,5-tetramethyl-1,3,2-dioxaborolan-2-yl)ethyl)oxazole (15c)

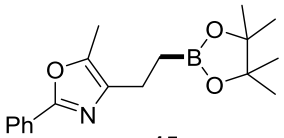

15c

According to General Procedure I, the reaction was carried out with **15a** (220.6 mg, 0.4 mmol) and B_2cat_2 (190.3 mg, 0.8 mmol) in DMA (1.0 mL). The crude product was purified by flash column chromatography on silica gel (toluene/PE = 1:1, 100 mL, then EA/PE = 20:1) to afford 87.5 mg (70%) of **15c** as a colorless oil. According to General Procedure II, afford 87.7 mg (70%) of **15c**: **^{1}H NMR (400 MHz, CDCl$_3$)** δ 7.99 – 7.93 (m, 2H), 7.43 – 7.35 (m, 3H), 2.59 (t, J = 7.8 Hz, 2H), 2.31 (s, 3H), 1.22 (s, 12H), 1.17 (t, J = 7.8 Hz, 2H); **^{13}C NMR (101 MHz, CDCl$_3$)** δ 159.0, 142.6, 137.7,

129.4, 128.5, 128.0, 125.8, 83.0, 24.8, 20.1, 10.2; **^{11}B NMR (128 MHz, CDCl$_3$)** δ 34.1; **ATR-FTIR (cm $^{-1}$):** 2977, 2923, 1636, 1371, 1142; **HRMS m/z (ESI)** calcd for $C_{18}H_{25}BNO_3$ (M + H)$^+$ 314.1922, found 314.1921.

^{13}C NMR C–B not detected due to quadropolar relaxation.

9-(5-(4,4,5,5-Tetramethyl-1,3,2-dioxaborolan-2-yl)pentyl)-9*H*-carbazole (16c)

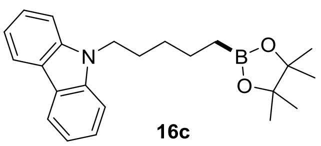

16c

According to General Procedure I, the reaction was carried out with **16a** (240.7 mg, 0.4 mmol) and B_2cat_2 (190.3 mg, 0.8 mmol) in DMA (1.0 mL). The crude product was purified by flash column chromatography on silica gel (toluene/PE = 1:1, 100 mL, then EA/PE = 20:1) to afford 127.9 mg (88%) of **16c** as a colorless oil. According to General Procedure II, afford 90.1 mg (62%) of **16c**: **^{1}H NMR (400 MHz, CDCl$_3$)** δ 8.06 (d, J = 7.7 Hz, 2H), 7.42 (t, J = 7.6 Hz, 2H), 7.35 (d, J = 8.1 Hz, 2H), 7.19 (t, J = 7.4 Hz, 2H), 4.22 (t, J = 7.2 Hz, 2H), 1.83 (p, J = 7.3 Hz, 2H), 1.47 – 1.41 (m, 2H), 1.36 (q, J = 8.0 Hz, 2H), 1.15 (s, 12H), 0.75 (t, J = 7.5 Hz, 2H); **^{13}C NMR (101 MHz, CDCl$_3$)** δ 140.3, 125.4, 122.7, 120.2, 118.6, 108.6, 82.8, 42.9, 29.8, 28.7, 24.6, 23.7, 10.9 (br.); **^{11}B NMR (128 MHz, CDCl$_3$)** δ 34.2; **ATR-FTIR (cm $^{-1}$):** 2976, 2928, 1483, 1323, 1142, 721; **HRMS m/z (ESI)** calcd for $C_{23}H_{31}BNO_2$ (M + H)$^+$ 364.2442, found 364.2441.

(*Z*)-4,4,5,5-tetramethyl-2-(octadec-9-en-1-yl)-1,3,2-dioxaborolane (17c)

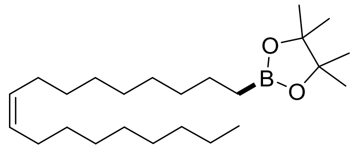

17c

According to General Procedure I, the reaction was carried out with **17a** (246.7 mg, 0.4 mmol) and B_2cat_2 (190.3 mg, 0.8 mmol) in DMA (1.0 mL). The crude product was purified by flash column chromatography on silica gel (toluene/PE = 1:1, 100 mL, then EA/PE = 20:1) to afford 116.6 mg (77%) of **17c** as a colorless oil. According to General Procedure II, afford According to General Procedure II, afford 87.8 mg (58%) of **17c**: **^{1}H NMR (400 MHz, CDCl$_3$)** δ 5.41 – 5.26 (m, 2H), 2.00 (m, 4H), 1.42 – 1.25 (m, 24H), 1.23 (s, 12H), 0.87 (t, J = 6.8 Hz, 3H), 0.75 (t, J = 7.7 Hz, 2H); **^{13}C NMR (101 MHz, CDCl$_3$)** δ 129.9, 129.8, 82.8, 32.4, 31.9, 29.8, 29.7, 29.6, 29.5, 29.5, 29.4, 29.3, 27.2, 27.2, 24.8, 24.0, 22.7,

14.1; **^{11}B NMR (128 MHz, $CDCl_3$)** δ 34.4; **ATR-FTIR (cm $^{-1}$):** 2922, 2852, 1370, 1316, 1145; **HRMS m/z (ESI)** calcd for $C_{24}H_{48}BO_2$ (M + H)$^+$ 379.3742, found 379.3738.

^{13}C NMR C–B not detected due to quadropolar relaxation.

2-(Dec-3-yn-1-yl)-4,4,5,5-tetramethyl-1,3,2-dioxaborolane (18c)

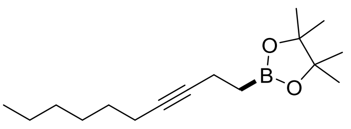

18c

According to General Procedure I, the reaction was carried out with **18a** (201.1 mg, 0.4 mmol) and B_2cat_2 (190.3 mg, 0.8 mmol) in DMA (1.0 mL). The crude product was purified by flash column chromatography on silica gel (toluene/PE = 1:1, 100 mL, then EA/PE = 20:1) to afford 85.6 mg (81%) of **18c** as a colorless oil. According to General Procedure II, afford 86.7 mg (82%) of **18c**: **^{1}H NMR (400 MHz, $CDCl_3$)** δ 8.20 (d, J = 7.9 Hz, 2H), 7.82 (d, J = 7.9 Hz, 2H), 7.73 (t, J = 7.7 Hz, 2H), 7.61 (t, J = 7.6 Hz, 2H), 3.84 (t, J = 6.4 Hz, 2H), 2.54 (t, J = 6.0 Hz, 2H), 2.01 – 1.90 (m, 2H), 1.34 (m, 2H), 1.27 – 1.13 (m, 6H), 0.81 (t, J = 6.8 Hz, 3H); **^{13}C NMR (101 MHz, $CDCl_3$)** δ 83.1, 81.6, 79.4, 31.4, 29.1, 28.5, 24.7, 22.5, 18.7, 14.0, 13.4, 11.5 (br.); **^{11}B NMR (128 MHz, $CDCl_3$)** δ 33.7; **ATR-FTIR (cm $^{-1}$):** 2929, 2857, 1370, 1245, 1146; **HRMS m/z (ESI)** calcd for $C_{16}H_{30}BO_2$ (M + H)$^+$ 265.2333, found 265.2330.

2-(Hept-6-en-3-yn-1-yl)-4,4,5,5-tetramethyl-1,3,2-dioxaborolane (19c)

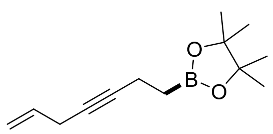

19c

According to General Procedure I, the reaction was carried out with **19a** (183.4 mg, 0.4 mmol) and B_2cat_2 (190.3 mg, 0.8 mmol) in DMA (1.0 mL). The crude product was purified by flash column chromatography on silica gel (toluene/PE = 1:1, 100 mL, then EA/PE = 20:1) to afford 66.0 mg (75%) of **19c** as a colorless oil. According to General Procedure II, afford 61.6 mg (70%) of **19c**: **^{1}H NMR (400 MHz, $CDCl_3$)** δ 5.76 (ddt, J = 16.9, 10.2, 5.2 Hz, 1H), 5.27 (dq, J = 16.9, 1.8 Hz, 1H), 5.02 (dq, J = 10.0, 1.7 Hz, 1H), 2.87 (tt, J = 4.1, 2.1 Hz, 2H), 2.25 (tt, J = 7.6, 2.3 Hz, 2H), 1.20 (s, 12H), 1.01 (d, J = 7.6 Hz, 2H); **^{13}C NMR (101 MHz, $CDCl_3$)** δ 133.3, 115.5, 84.3, 83.1, 75.7, 24.7, 23.0, 13.4; **^{11}B NMR (128 MHz, $CDCl_3$)** δ 33.5; **ATR-FTIR (cm $^{-1}$):**

2979, 2929, 2249, 1737, 1371, 1308, 910; **HRMS m/z (ESI)** calcd for $C_{13}H_{22}BO_2$ (M + H)$^+$ 221.1707, found 221.1706.

^{13}C NMR C–B not detected due to quadropolar relaxation.

2-(*Sec*-butyl)-4,4,5,5-tetramethyl-1,3,2-dioxaborolane (20c)

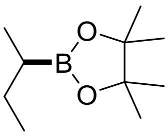

20a

According to General Procedure I, the reaction was carried out with **20a** (169.0 mg, 0.4 mmol) and B_2cat_2 (190.3 mg, 0.8 mmol) in DMA (1.0 mL). The crude product was purified by flash column chromatography on silica gel (toluene/PE = 1:1, 100 mL, then EA/PE = 20:1) to afford 31.7 mg (43%) of **20c** as a colorless oil. According to General Procedure II, afford 15.5 mg (21%) of **20c**: **^{1}H NMR (400 MHz, CDCl$_3$)** δ 1.52 – 1.42 (m, 1H), 1.33 (m, 1H), 1.23 (s, 12H), 1.00 – 0.93 (m, 4H), 0.90 (t, J = 7.4 Hz, 3H); **^{13}C NMR (101 MHz, CDCl$_3$)** δ 82.8, 26.1, 24.7, 24.7, 15.2, 13.4; **^{11}B NMR (128 MHz, CDCl$_3$)** δ 34.5; **ATR-FTIR (cm^{-1}):** 2979, 1381, 1141, 903, 728; **HRMS m/z (ESI)** calcd for $C_{10}H_{22}BO_2$ (M + H)$^+$ 185.1707, found 185.1704. All spectral data are in accordance with the literature.[10]

4,4,5,5-Tetramethyl-2-(1-phenylpropan-2-yl)-1,3,2-dioxaborolane (21c)

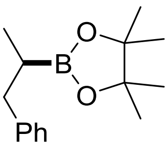

21c

According to General Procedure I, the reaction was carried out with **1a** (193.8 mg, 0.4 mmol) and B_2cat_2 (190.3 mg, 0.8 mmol) in DMA (1.0 mL). The crude product was purified by flash column chromatography on silica gel (toluene/PE = 1:1 to 2:1) to afford 73.8 mg (75%) of **21c** as a colorless oil. According to General Procedure II, afford 33.0 mg (34%) of **21c**: **^{1}H NMR (400 MHz, CDCl$_3$)** δ 7.27 – 7.18 (m, 4H), 7.15 (tt, J = 6.2, 1.5 Hz, 1H), 2.81 (dd, J = 13.5, 7.4 Hz, 1H), 2.54 (dd, J = 13.6, 8.4 Hz, 1H), 1.37 (m, 1H), 1.18 (d, J = 4.4 Hz, 12H), 0.96 (d, J = 7.4 Hz, 3H); **^{13}C NMR (101 MHz, CDCl$_3$)** δ 142.3, 128.9, 128.0, 125.5, 83.0, 39.0, 24.7, 15.2; **^{11}B NMR (128 MHz, CDCl$_3$)** δ 34.5; **HRMS m/z (ESI)** calcd for $C_{15}H_{24}BO_2$ (M + H)$^+$ 247.1864, found 247.1866. All spectral data are in accordance with the literature.[11]

2-Cyclohexyl-4,4,5,5-tetramethyl-1,3,2-dioxaborolane (22c)

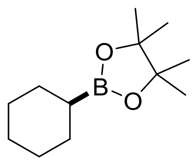

22c

According to General Procedure I, the reaction was carried out with **20a** (195.4 mg, 0.4 mmol) and B_2cat_2 (190.3 mg, 0.8 mmol) in DMA (1.0 mL). The crude product was purified by flash column chromatography on silica gel (toluene/PE = 1:1, 100 mL, then EA/PE = 20:1) to afford 45.4 mg (54%) of **22c** as a colorless oil. According to General Procedure II, afford 51.3 mg (61%) of **22c**: **^{1}H NMR (500 MHz, $CDCl_3$)** δ 1.70 – 1.54 (m, 5H), 1.31 (dt, J = 16.9, 6.7 Hz, 5H), 1.22 (s, 12H), 0.97 (tt, J = 9.8, 7.3, 5.4 Hz, 1H); **^{13}C NMR (126 MHz, $CDCl_3$)** δ 82.7, 27.9, 27.1, 26.7, 24.7; **^{11}B NMR (160 MHz, $CDCl_3$)** δ 34.0; **ATR-FTIR (cm $^{-1}$):** 2977, 2920, 2849, 1379, 1308, 1143, 853; **HRMS m/z (ESI)** calcd for $C_{12}H_{24}BO_2$ $(M + H)^+$ 211.1864, found 211.1864. All spectral data are in accordance with the literature.[4]

^{13}C NMR C–B not detected due to quadropolar relaxation.

2-Cyclododecyl-4,4,5,5-tetramethyl-1,3,2-dioxaborolane (23c)

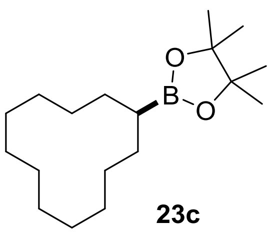

23c

According to General Procedure I, the reaction was carried out with **23a** (213.1 mg, 0.4 mmol) and B_2cat_2 (190.3 mg, 0.8 mmol) in DMA (1.0 mL). The crude product was purified by flash column chromatography on silica gel (toluene/PE = 1:1, 100 mL, then EA/PE = 20:1) to afford 81.2 mg (69%) of **23c** as a colorless oil. According to General Procedure II, afford 61.2 mg (52%) of **23c**: **^{1}H NMR (500 MHz, $CDCl_3$)** δ 1.36 (m, 22H), 1.22 (s, 12H), 1.04 (dt, J = 12.3, 6.5 Hz, 1H); **^{13}C NMR (126 MHz, $CDCl_3$)** δ 82.7, 24.8, 24.7, 24.2, 24.1, 23.4, 23.4, 23.3; **^{11}B NMR (160 MHz, $CDCl_3$)** δ 34.6; **ATR-FTIR (cm $^{-1}$):** 2927, 2861, 1384, 1143; **HRMS m/z (ESI)** calcd for $C_{18}H_{35}BNaO_2$ $(M + Na)^+$ 317.2622, found 317.2621. All spectral data are in accordance with the literature.[4]

^{13}C NMR C–B not detected due to quadropolar relaxation.

2-((1*r*,3*s*,5*R*,7*S*)-Adamantan-2-yl)-4,4,5,5-tetramethyl-1,3,2-dioxaborolane (24c)

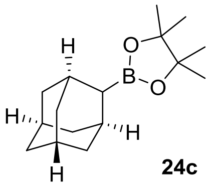

24c

According to General Procedure I, the reaction was carried out with **24a** (200.2 mg, 0.4 mmol) and B_2cat_2 (190.3 mg, 0.8

mmol) in DMA (1.0 mL). The crude product was purified by flash column chromatography on silica gel (toluene/PE = 1:1, 100 mL, then EA/PE = 20:1) to afford 91.2 mg (87%) of **24c** as a colorless oil. According to General Procedure II, afford 76.7 mg (73%) of **24c**: **^{1}H NMR (400 MHz, $CDCl_3$)** δ 2.04 (s, 2H), 1.90 – 1.65 (m, 12H), 1.35 (s, 1H), 1.24 (s, 12H); **^{13}C NMR (101 MHz, $CDCl_3$)** δ 82.7, 39.3, 37.7, 36.2, 29.3, 28.2, 28.1, 24.8; **^{11}B NMR (128 MHz, $CDCl_3$)** δ 33.9; **ATR-FTIR (cm^{-1}):**2246, 1727, 1412, 1590, 1515, 1462, 1171, 1064, 775; **HRMS m/z (ESI)** calcd for $C_{16}H_{28}BO_2$ $(M + H)^+$ 263.2177, found 263.2175. All spectral data are in accordance with the literature.[12]

^{13}C NMR C–B not detected due to quadropolar relaxation.

Benzyl-4,4,5,5-tetramethyl-1,3,2-dioxaborolane(25c)

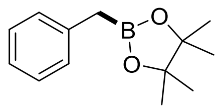

25c

An 8.0 mL dry tube with a stirring bar was added with benzyl-substituted thianthrenium salt **25a** (0.1 mmol, 80 mg) and B_2cat_2 (47.6 mg, 0.2 mmol). The tube was placed in a water bath under blue LED irradiation, then DMA (1.0 mL) was added. The mixture was stirred under argon for 12 h. Then pinacol (47.3 mg, 0.4 mmol) was dissolved in Et_3N (0.5 mL), added to the reaction mixture and stirred for 1 h. Then water was added, and the reaction mixture was extracted with EtOAc three times, dried over $MgSO_4$ and concentrated under reduced pressure. The crude product was purified by flash column chromatography on silica gel (toluene/PE = 1:1, then EA/PE = 20:1) to afford 11.0 mg (50%) of **25c** as a semi-solid. According to General Procedure II, afford trace of **25c**: **^{1}H NMR (500 MHz, $CDCl_3$)** δ 7.26 – 7.21 (m, 2H), 7.21 – 7.16 (m, 2H), 7.15 – 7.10 (m, 1H), 2.30 (s, 2H), 1.23 (s, 12H). **^{13}C NMR (126 MHz, $CDCl_3$)** δ 138.6, 129.0, 128.2, 124.8, 83.4, 24.7. **^{11}B NMR (160 MHz, $CDCl_3$)** δ 33.1. All spectral data are in accordance with the literature.[29]

2-(3,3,4,4,5,5,6,6,7,7,8,8,9,9,10,10,10-Heptadecafluorodecyl)-4,4,5,5-tetramethyl-1,3,2-dioxaborolane (26c)

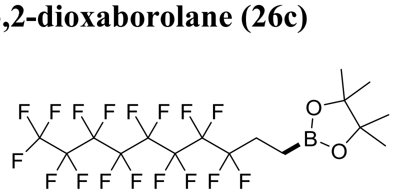

26c

According to General Procedure I, the reaction was carried out with **26a** (345.0 mg, 0.4 mmol) and

B_2cat_2 (190.3 mg, 0.8 mmol) in DMA (1.0 mL). The crude product was purified by flash column chromatography on silica gel (toluene/PE = 1:1, 100 mL, then EA/PE = 20:1) to afford 181.4 mg (79%) of **26c** as a colorless oil. According to General Procedure II, afford 170.0 mg (74%) of **26c**: **^{1}H NMR (400 MHz, $CDCl_3$)** δ 2.19 (tt, J = 18.5, 8.2 Hz, 2H), 1.25 (s, 12H), 1.04 (t, J = 8.0, 2H); **^{13}C NMR (101 MHz, $CDCl_3$)** δ 83.6, 25.7 (t, J = 23.0 Hz), 24.7; **^{19}F NMR (376 MHz, $CDCl_3$)** δ -80.9 (t, J = 10.0 Hz, 2F), -116.1 – -116.3 (m, 2F), -121.8 (s, 2F), -122.0 (s, 2F), -122.8 (s, 2F), -123.6 (s, 2F), -126.23 – 126.3 (m, 2F); **^{11}B NMR (128 MHz, $CDCl_3$)** δ 33.3; **ATR-FTIR (cm $^{-1}$):**2246, 1727, 1412, 1590, 1515, 1462, 1171, 1064, 775; **HRMS m/z (ESI)** calcd for $C_{16}H_{17}BF_{17}O_2$ $(M + H)^+$ 575.1045, found 575.1042. All spectral data are in accordance with the literature.[13]

^{13}C NMR C–B not detected due to quadropolar relaxation. The carbon atoms bearing fluorine atoms were observed (see NMR spectrum) but were not assigned.

4,4,5,5-Tetramethyl-2-((9*Z*,12*Z*,15*Z*)-octadeca-9,12,15-trien-1-yl)-1,3,2-dioxaborolane (27c)

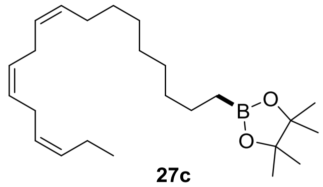

27c

According to General Procedure I, the reaction was carried out with **27a** (245.1 mg, 0.4 mmol) and B_2cat_2 (190.3 mg, 0.8 mmol) in DMA (1.0 mL). The crude product was purified by flash column chromatography on silica gel (toluene/PE = 1:1, 100 mL, then EA/PE = 20:1) to afford 134.8 mg (90%) of **27c** as a colorless oil. According to General Procedure II, afford 100.3 mg (67%) of **27c**: **^{1}H NMR (400 MHz, $CDCl_3$)** δ 5.45 – 5.25 (m, 6H), 2.79 (m, 4H), 2.05 (m, 4H), 1.40 – 1.26 (m, 12H), 1.23 (s, 12H), 0.97 (t, J = 7.5 Hz, 3H), 0.75 (t, J = 7.7 Hz, 2H); **^{13}C NMR (101 MHz, $CDCl_3$)** δ 131.9, 130.4, 130.2, 130.1, 128.3, 128.2, 127.9, 127.9, 127.6, 127.1, 82.8, 32.4, 29.6, 29.4, 29.4, 29.3, 27.2, 25.6, 25.5, 24.8, 24.0, 20.5, 14.2; **^{11}B NMR (128 MHz, $CDCl_3$)** δ 34.0; **ATR-FTIR (cm $^{-1}$):** 2976, 2923, 2853, 1370, 1145; **HRMS m/z (ESI)** calcd for $C_{24}H_{43}BNaO_2$ $(M + H)^+$ 397.3248, found 397.3241.

^{13}C NMR C–B not detected due to quadropolar relaxation.

(*E*)-2-(2-(4-(4-chloro-1,2-diphenylbut-1-en-1-yl)phenoxy)ethyl)-4,4,5,5-tetramethyl-1,3,2-dioxaborolane (28c)

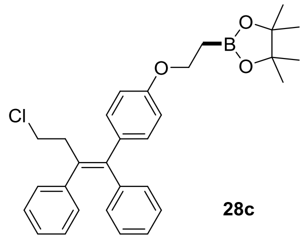

28c

According to General Procedure I, the reaction was carried out with **28a** (290.9 mg, 0.4 mmol) and B_2cat_2 (190.3 mg, 0.8 mmol) in DMA (1.0 mL). The crude product was purified by flash column chromatography on silica gel (toluene/PE = 1:1, 100 mL, then EA/PE = 20:1) to afford 138.8 mg (71%) of **28c** as a colorless oil. According to General Procedure II, afford 111.5 mg (57%) of **28c**: **^{1}H NMR (400 MHz, $CDCl_3$)** δ 7.42 – 7.35 (m, 2H), 7.31 (d, J = 7.3 Hz, 3H), 7.23 – 7.14 (m, 5H), 6.78 (d, J = 8.7 Hz, 2H), 6.57 (d, J = 8.7 Hz, 2H), 3.99 (t, J = 7.8 Hz, 2H), 3.43 (t, J = 7.5 Hz, 2H), 2.94 (t, J = 7.5 Hz, 2H), 1.33 – 1.29 (m, 2H), 1.25 (s, 12H); **^{13}C NMR (101 MHz, $CDCl_3$)** δ 157.3, 142.9, 141.8, 141.0, 134.9, 134.4, 131.5, 129.5, 129.3, 128.3, 128.1, 126.8, 126.5, 113.5, 83.3, 64.6, 42.8, 38.6, 24.7; **^{11}B NMR (128 MHz, $CDCl_3$)** δ 33.1; **ATR-FTIR (cm $^{-1}$):** 2978, 2250, 1665, 1323, 905; **HRMS m/z (ESI)** calcd for $C_{30}H_{34}BClNaO_3$ $(M + H)^+$ 511.2182, found 511.2182.

^{13}C NMR C–B not detected due to quadropolar relaxation.

2-(4-((3*R*,5*R*,8*R*,9*S*,10*S*,13*R*,14*S*,17*R*)-3-methoxy-10,13-dimethylhexadecahydro-1*H*-cyclopenta[*a*]phenanthren-17-yl)pentyl)-4,4,5,5-tetramethyl-1,3,2-dioxaborolane (29c)

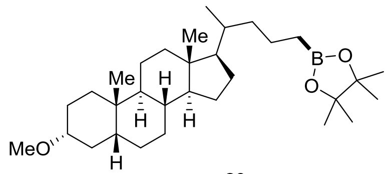

29c

According to General Procedure I, the reaction was carried out with **29a** (290.0 mg, 0.4 mmol) and B_2cat_2 (190.3 mg, 0.8 mmol) in DMA (1.0 mL). The crude product was purified by flash column chromatography on silica gel (toluene/PE = 1:1, 100 mL, then EA/PE = 20:1) to afford 173.2 mg (89%) of **29c** as a colorless oil. According to General Procedure II, afford 107.1 mg (55%) of **29c**: **^{1}H NMR (500 MHz, $CDCl_3$)** δ 3.32 (s, 3H), 3.13 (tt, J = 10.9, 4.5 Hz, 1H), 1.95 – 1.90 (m, 1H), 1.87 – 1.69 (m, 5H), 1.68 – 1.61 (m, 1H), 1.58 – 1.49 (m, 2H), 1.38 – 1.30 (m, 7H), 1.22 (s, 18H), 1.10 – 0.98 (m, 6H), 0.89 (s,

3H), 0.86 (d, J = 6.5 Hz, 3H), 0.74 – 0.65 (m, 2H), 0.60 (s, 3H); **^{13}C NMR (126 MHz, $CDCl_3$)** δ 82.7, 80.4, 56.4, 56.1, 55.5, 42.6, 42.0, 40.3, 40.1, 38.8, 35.8, 35.6, 35.3, 34.8, 32.7, 28.2, 27.3, 26.7, 26.4, 24.8, 24.2, 23.4, 20.8, 20.5, 18.7, 11.9; **^{11}B NMR (160 MHz, $CDCl_3$)** δ 34.6; **ATR-FTIR (cm $^{-1}$):** 2928, 2864, 1370, 1098, 737; **HRMS m/z (ESI)** calcd for $C_{31}H_{55}BNaO_3$ (M + Na)$^+$ 509.4136, found 509.4133.

^{13}C NMR C–B not detected due to quadropolar relaxation.

4,4,5,5-Tetramethyl-2-((9*Z*,12*Z*,15*Z*)-octadeca-9,12,15-trien-1-yl)-1,3,2-dioxaborolane (30c)

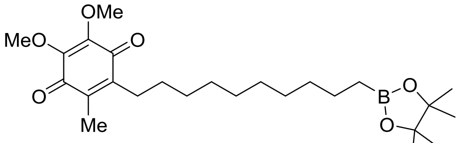

30c

According to General Procedure I, the reaction was carried out with **30a** (274.7 mg, 0.4 mmol) and B_2cat_2 (190.3 mg, 0.8 mmol) in DMA (1.0 mL). The crude product was purified by flash column chromatography on silica gel (toluene/PE = 1:1, 100 mL, then EA/PE = 10:1) to afford 91.5 mg (51%) of **30c** as a colorless oil. According to General Procedure II, **30c** was not detected: **^{1}H NMR (400 MHz, $CDCl_3$)** δ 3.97 (s, 6H), 2.46 – 2.37 (m, 2H), 1.99 (s, 3H), 1.42 – 1.23 (m, 16H), 1.22 (s, 12H), 0.74 (t, J = 7.7 Hz, 2H); **^{13}C NMR (101 MHz, $CDCl_3$)** δ 184.7, 184.1, 144.3, 143.1, 138.6, 82.8, 61.1, 32.4, 29.8, 29.5, 29.5, 29.3, 29.3, 28.7, 26.4, 24.8, 23.9, 11.9; **^{11}B NMR (128 MHz, $CDCl_3$)** δ 34.3; **ATR-FTIR (cm $^{-1}$):** 2977, 2924, 2853, 1648, 1610, 1265; **HRMS m/z (ESI)** calcd for $C_{25}H_{42}BO_6$ (M + H)$^+$ 449.3069, found 449.3064.

1,6-Bis(4,4,5,5-tetramethyl-1,3,2-dioxaborolan-2-yl)hexane (31c)

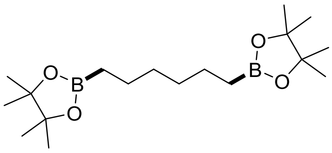

31c

According to General Procedure I, the reaction was carried out with **31a** (326.0 mg, 0.4 mmol) and B_2cat_2 (1.6 mmol, 381.6 mg) in DMA (1.5 mL). The crude product was purified by flash column chromatography on silica gel (toluene/PE = 1:1, 100 mL, then EA/PE = 20:1) to afford 83.8 mg (62%) of **31c** as a colorless oil. According to General Procedure II, the reaction was carried out with **31a** (326.0 mg, 0.4 mmol), **B1** (52.0 mg, 0.24 mmol) and B_2cat_2 (381.6 mg, 1.6 mmol) in DMA (1.5 mL) afford 73.0 mg (54%) of **31c**: **^{1}H NMR (400 MHz, $CDCl_3$)** δ 1.38 – 1.31 (m,

4H), 1.24 (d, J = 6.8 Hz, 4H), 1.19 (s, 24H), 0.71 (t, J = 7.7 Hz, 4H); **^{13}C NMR (101 MHz, CDCl$_3$)** δ 82.7, 32.1, 24.7, 23.9, 11.1 (br.); **^{11}B NMR (128 MHz, CDCl$_3$)** δ 34.0; **ATR-FTIR (cm $^{-1}$):** 2977, 2926, 1370, 1315, 1143; **HRMS m/z (ESI)** calcd for $C_{18}H_{37}B_2O_4$ $(M + H)^+$ 339.2872, found 339.2870. All spectral data are in accordance with the literature.[14]

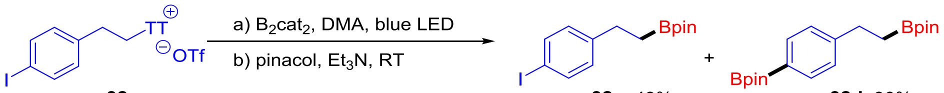

According to General Procedure I, the reaction was carried out with **32a** (219.8 mg, 0.4 mmol) and B_2cat_2 (190.3 mg, 0.8 mmol) in DMA (1.0 mL). The crude product was purified by flash column chromatography on silica gel (toluene/PE = 1:1, 100 mL, then EA/PE = 20:1) to afford 61.6 mg (43%) of **32c** and 42.8 mg (30%) of **32d** as white solids.

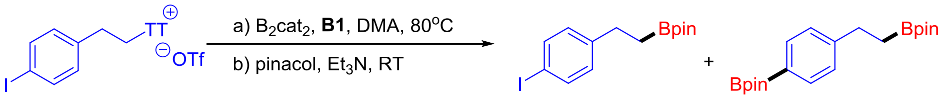

According to General Procedure II, the reaction was carried out with **32a** (326.0 mg, 0.4 mmol), **B1** (26.0 mg, 0.12 mmol) and B_2cat_2 (190.3 mg, 0.8 mmol) in DMA (1.0 mL) afford 96.0 mg (67%) of **32c**. The double borylation product **32d** was not detected.

2-(4-Iodophenethyl)-4,4,5,5-tetramethyl-1,3,2-dioxaborolane (32c)

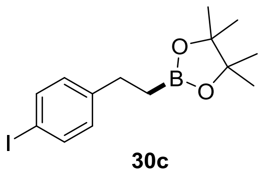

30c

^{1}H NMR (500 MHz, CDCl$_3$) δ 7.56 (d, J = 8.3 Hz, 2H), 6.96 (d, J = 8.3 Hz, 2H), 2.71 – 2.64 (m, 2H), 1.21 (s, 12H), 1.13 – 1.08 (m, 2H); **^{13}C NMR (126 MHz, CDCl$_3$)** δ 144.0, 137.1, 130.1, 90.4, 83.1, 29.4, 24.8; **^{11}B NMR (160 MHz, CDCl$_3$)** δ 33.8; **ATR-FTIR (cm $^{-1}$):** 2976, 2928, 1369, 1315, 1141, 849; **HRMS m/z (ESI)** calcd for $C_{14}H_{21}BIO_2$ $(M + H)^+$ 359.0674, found 359.0675.

^{13}C NMR C–B not detected due to quadropolar relaxation.

4,4,5,5-Tetramethyl-2-(4-(2-(4,4,5,5-tetramethyl-1,3,2-dioxaborolan-2-yl)ethyl)ph

enyl)-1,3,2-dioxaborolane (32d)

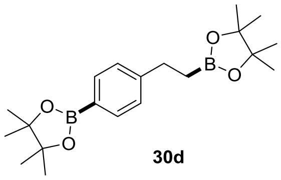

^{1}H NMR (500 MHz, CDCl$_3$) δ 7.71 (d, J = 7.9 Hz, 2H), 7.23 (d, J = 7.8 Hz, 2H), 2.78 – 2.73 (m, 2H), 1.33 (s, 12H), 1.22 (s, 12H), 1.15 – 1.11 (m, 2H); **^{13}C NMR (126 MHz, CDCl$_3$)** δ 147.9, 134.8, 127.4, 83.6, 83.1, 30.2, 24.8, 24.8; **^{11}B NMR (160 MHz, CDCl$_3$)** δ 34.2, 31.3; **ATR-FTIR (cm $^{-1}$):** 2979, 2921, 1398, 1355, 1320, 1140, 1089; **HRMS m/z (ESI)** calcd for $C_{20}H_{33}B_2O_4$ $(M + H)^+$ 359.2559, found 359.2555. All spectral data are in accordance with the literature.[16]

^{13}C NMR C–B not detected due to quadropolar relaxation.

Recovery of Thianthrene and One-pot Protocol

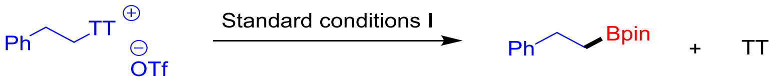

A 10.0 mL Schlenk tube with a stirring bar was added with **1a** (188.2 mg, 0.4 mmol), B_2cat_2 (190.3 mg, 0.8 mmol, 2.0 equiv) and DMA (1.0 mL) under argon. The resulting mixture was stirred at room temperature under blue LED irradiation for 12 h. Then pinacol (189.1 mg, 1.6 mmol, 4.0 equiv) was dissolved in Et_3N (1.0 mL), added to the reaction mixture and stirred for 1 h. Then water was added, and the reaction mixture was extracted with EtOAc three times, dried over $MgSO_4$ and concentrated under reduced pressure. The crude product was purified by flash column chromatography on silica gel (toluene/PE = 1:1, then EA/PE = 20:1) to afford 85.7 mg (99%) of thianthrene as a white solid and 76.1 mg (82%) of **1c**.

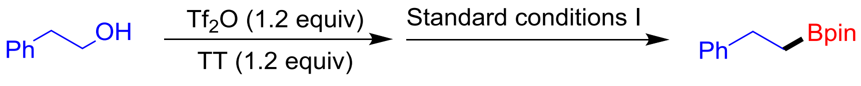

A flame-dried 10 mL flask was placed under an atmosphere of nitrogen and charged with a stir bar and alcohol **I** (1.0 mmol, 122.2 mg). The alcohol was dissolved in CH_2Cl_2 (4.0 mL) and cooled to -30 °C before adding pyridine (95 mg, 1.2 mmol). While stirring, triflic anhydride (338.6 mg, 1.2 mmol) was added dropwise, and then the reaction mixture stirred for 3 h while remaining at -5 °C. While the flask was still

in a -5 °C bath, 0.5 M H_2SO_4 (5 mL) was added. The flask was removed from the cold bath, and the mixture was transferred to a separatory funnel and extracted with 3×5 mL of CH_2Cl_2. The organic layers were combined and washed 1× 5mL of distilled water. The collected organic layers were then dried over $MgSO_4$, then filtered and concentrated to a 2 mL liquid under vacuum (without heating), then thianthrene (260 mg, 1.2 mmol) was added. Then mixture was stired at 60 °C for 24 h.

The mixture was cooled to room temperature and the solvent was removed concentrated under vacuum. Then B_2cat_2 (190.3 mg, 0.8 mmol, 2.0 equiv) and DMA (1.0 mL) under argon. The resulting mixture was stirred at room temperature under blue LED irradiation for 12 h. Then pinacol (473.0 mg, 4.0 mmol) was dissolved in Et_3N (2.0 mL), added to the reaction mixture and stirred for 1 h. Then water was added, and the reaction mixture was extracted with EtOAc three times, dried over $MgSO_4$ and concentrated under reduced pressure. The crude product was purified by flash column chromatography on silica gel (toluene/PE = 1:1, then EA/PE = 20:1) to afford 155.5 mg (67%) of **1c**.

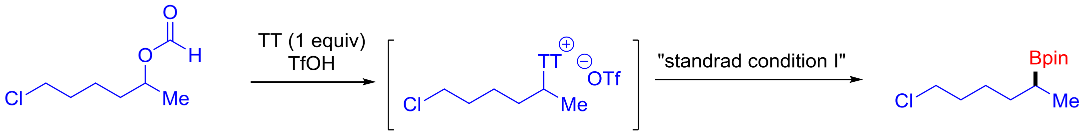

34 (2 equiv) **33** **35**, 45%

To a stirred mixture of thianthrene (216.3 mg, 1.0 mmol) and formate **34** (2.0 mmol, 329.3 mg), cooled in an ice-bath, was added 0.5 ml of trifluoromethanesulfonic acid. The mixture was removed from the ice-bath and stirred for 10 h at room temperature, after which it was poured into 20 mL of water. The resulting suspension was extracted with DCM. The collected organic layers were then dried over $MgSO_4$, then filtered and concentrated under reduced pressure at 25 ºC. The resulted oil was washed with ether and transferred to 8 mL vial. Then the vial was charged with a stir bar and B_2cat_2 (475.8 mg, 2.0 mmol). The tube was placed in a water bath under blue LED irradiation, then DMA (2.5 mL) was added. The mixture was stirred under argon for 12 h. Then pinacol (473.0 mg, 4.0 mmol) was dissolved in Et_3N (2.0 mL), added to the reaction mixture and stirred for 1 h. Then water was added, and the reaction mixture was extracted with EtOAc three times, dried over $MgSO_4$ and concentrated

under reduced pressure. The crude product was purified by flash column chromatography on silica gel (toluene/PE = 1:1) to afford 110.0 mg (45%) of **35** as a colorless oil.

2-(6-Chlorohexan-2-yl)-4,4,5,5-tetramethyl-1,3,2-dioxaborolane (35)

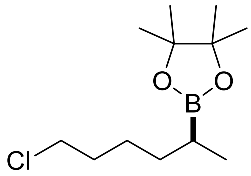

35

^{1}H NMR (400 MHz, $CDCl_3$) δ 3.51 (t, J = 6.8 Hz, 2H), 1.82 – 1.71 (m, 2H), 1.46 (dtd, J = 29.7, 14.0, 6.0 Hz, 4H), 1.24 (s, 12H), 0.90 (t, J = 7.4 Hz, 4H). **^{13}C NMR (101 MHz, $CDCl_3$)** δ 83.0, 45.3, 32.3, 28.2, 24.8, 24.1, 13.5. **^{11}B NMR (128 MHz, $CDCl_3$)** δ 33.9. **ATR-FTIR (cm $^{-1}$):** 2929, 1400, 1321, 1121, 624; **HRMS m/z (ESI)** calcd for $C_{12}H_{25}BClO_2$ $(M + H)^+$ 247.1631, found 247.1633. All spectral data are in accordance with the literature.[30]

^{13}C NMR C–B not detected due to quadropolar relaxation.

Supplementary discussion

Mechanistic Studies

Pyridinium salt **II** and NHPI ester **III** were were prepared according to the literature.

Pyridinium salt **II**

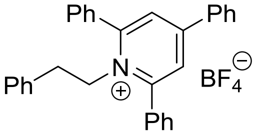

II

: **^{1}H NMR (400 MHz, Acetone-d_6)** δ 8.44 (s, 2H), 8.27 – 8.15 (m, 2H), 7.97 – 7.86 (m, 4H), 7.83 – 7.71 (m, 6H), 7.71 – 7.60 (m, 3H), 7.26 – 7.01 (m, 3H), 6.51 – 6.41 (m, 2H), 4.82 – 4.75 (m, 2H), 2.87 – 2.82 (m, 2H); **^{13}C NMR (101 MHz, Acetone-d_6)** δ 157.8, 156.6, 136.7, 134.8, 134.2, 133.4, 132.1, 130.7, 130.3, 129.7, 129.5, 129.2, 128.2, 127.6, 57.1, 36.2. **^{19}F NMR (376 MHz, Acetone-d_6)** δ 25.85, 25.80. All spectral data are in accordance with the literature.[4]

NHPI ester **III**

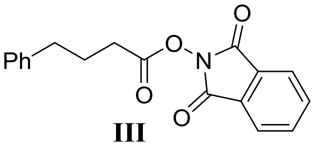

III

: **^{1}H NMR (400 MHz, Acetone-d_6)** δ 7.94 (s, 4H), 7.34 (dt, J = 15.0, 7.4 Hz, 4H), 7.23 (t, J = 7.0 Hz, 1H), 3.07 (q, J = 7.0, 6.1 Hz, 4H); **^{13}C NMR (101 MHz, Acetone-d_6)** δ 170.1, 162.7, 140.6, 136.1, 129.8, 129.4, 129.3, 127.4, 124.7, 33.0, 31.2. All spectral

data are in accordance with the literature.[17]

Cyclic Voltammetry Studies for 1a, II and III

Cyclic voltammograms were carried out in DMA, polished Pt plate as the working electrode, platinum wire as counter electrode, and Ag-$AgNO_3$ (0.1 M) in CH_3CN as the reference electrode with a scan rate of 100 mV/s. The solution of sample (5.0 x 10^{-2} M) and tetra-*n*-butylammonium hexafluorophosphate (0.1 M) in dry DMA was deaerated by N_2 gas bubbling before the measurement, and the cyclic voltammetry was carried out under an N_2 gas atmosphere at room temperature. The $E_{1/2}$ value of the ferrocene–ferrocenium (Fc/Fc^+) in DMA was +0.10 V vs. Ag/$AgNO_3$ with this setup.

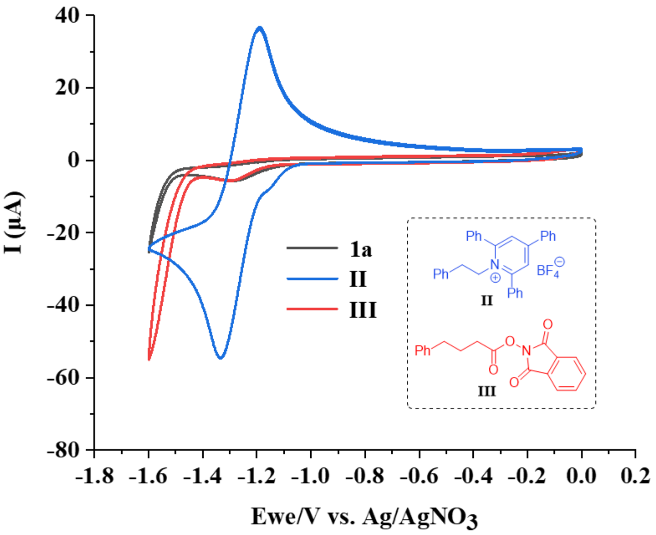

Supplementary Figure 6. Cyclic voltammograms. Cyclic voltammograms of thianthrenium salt **1a**, Katritzky salt **II** and NHPI ester **III**

E_P^{red} were obtained from the graphs as the potential corresponding to the maximum current observed. **1a** shows a completely irreversible reduction wave with E_P^{red} = -1.28 V (vs Ag/$AgNO_3$). **II** shows a completely irreversible reduction wave with E_P^{red} = -1.33 V (vs Ag/Ag NO_3). **III** shows a completely irreversible reduction wave with E_P^{red} = -1.30 V (vs Ag/Ag NO_3).

UV/Vis Absorption Spectra

The UV/Vis absorption spectra of DMA solutions of sulfonium salt **1a** (0.10 M),

B_2cat_2 (0.20 M), and a mixture of **1a** (0.10 M) and B_2cat_2 (0.20 M) are shown in Figure S1. The bathochromic shift is indicative of EDA complex formation.

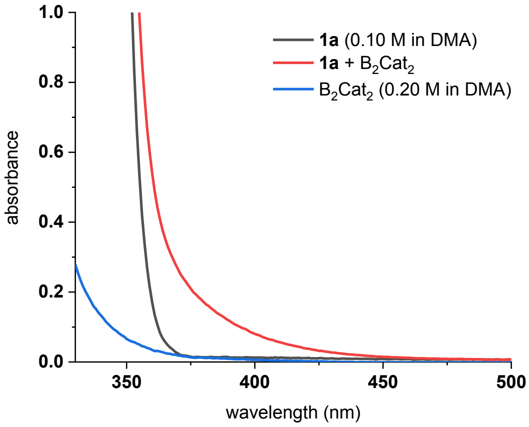

Supplementary figure 7. UV/visible absorption. UV/visible absorption spectra of DMA solution of **1a** (0.1 M), B_2Cat_2 (0.2 M), and a mixture of **1a** (0.1 M) and B_2cat_2 (0.2 M).

Determination of the light intensity at 440 nm:

According to the works by Yoon and co-workers,[18] the photon flux of the LED (λmax = 440 nm) was determined by standard ferrioxalate actinometry. The photon flux of the spectrophotometer was determined by standard ferrioxalate actinometry. A 0.15 M solution of ferrioxalate was prepared by dissolving 737 mg of potassium ferrioxalate hydrate in 10 mL of 0.05 M H_2SO_4. A buffered solution of phenanthroline was prepared by dissolving 25 mg of phenanthroline and 5.63 g of sodium acetate in 25 mL of 0.5 M H_2SO_4. Both solutions were stored in the dark. To determine the photon flux of the spectrophotometer, 2.0 mL of the ferrioxalate solution was placed in a quartz cuvette (path length: l = 1.0 cm) and irradiated for 90.0 seconds at λ = 440 nm. After irradiation, 0.35 mL of the phenanthroline solution was added to the cuvette. The solution was then allowed to rest for 1 h to allow the ferrous ions to completely coordinate to the phenanthroline. The absorbance of the solution was measured at 510 nm. A non-irradiated sample was also prepared and the absorbance at 510 nm measured. Conversion was calculated using eq 1.

$$\text{mol Fe}^{2+} = \frac{V \times \Delta A}{1 \times \varepsilon} \quad (1)$$

Where V is the total volume (0.00235 L) of the solution after addition of phenanthroline, ΔA is the difference in absorbance at 510 nm between the irradiated and non-irradiated solutions, l is the path length (1.000 cm), and ε is the molar absorptivity at 510 nm (11,100 L mol^{-1} cm^{-1}). mol Fe^{2+} = $\frac{0.00235 \times (1.4444 - 0.6384)}{1 \times 11100}$ = 1.7×10^{-7} mol

The photon flux can be calculated using eq 2.

$$\text{photon flux} = \frac{\text{mol Fe}^{2+}}{\Phi \times t \times f} \quad (2)$$

Where Φ is the quantum yield for the ferrioxalate actinometer (1.01 for a 0.15 M solution at λ = 440 nm),[19] t is the time (90.0 s), and f is the fraction of light absorbed at λ = 440 nm (0.9978). The photon flux was calculated (average of three experiments) to be 1.87×10^{-9} einstein s^{-1}. photon flux = $\frac{1.7 \times 10^{-7}}{1.01 \times 90 \times 0.9978}$ = 1.87×10^{-9} einstein s^{-1}

Quantum Yield Measurement:

To a quartz cuvette (path length, l = 1 cm) equipped with a magnetic stir bar was added **1a** (188.2 mg, 0.4 mmol), B_2cat_2 (190.8 mg, 0.8 mmol) and DMA (1.0 mL). the cuvette was further sealed with parafilm. The cuvette was positioned in a water bath and 2 cm away from a single 40 W blue LED and the reaction was stirred and irradiated for 1 h. Irradiation was stopped, a solution of pinacol (189.1 mg, 1.6 mmol) in triethylamine (1.0 mL) was added, and the mixture was stirred at room temperature for 1 h. The yield was determined by nmr, using CH_2Br_2 as an internal standard, to be 77.0% (3.08×10^{-4} mol).

$$\Phi = \frac{\text{mol product}}{\text{photonflux} \times t \times f} \quad (3)$$

Where t is the time (3600 s) and f is the fraction of light absorbed by the reaction mixture at λ = 440 nm, where f = $1-10^{-A}$. Φ = $\frac{3.08 \times 10^{-4}}{1.87 \times 10^{-9} \times 3600 \times 0.9978}$ = 46

Radical trapping experiment with TEMPO

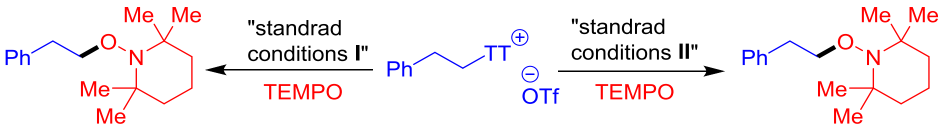

A 10.0 mL Schlenk tube with a stirring bar was added with **1a** (188.2 mg, 0.4 mmol), B_2cat_2 (190.3 mg, 0.8 mmol, 2.0 equiv), TEMPO (0.8 mmol, 125.0 mg) and DMA (1.0 mL) under argon. The resulting mixture was stirred at room temperature under blue LED irradiation for 12 h. Then pinacol (189.1 mg, 1.6 mmol, 4.0 equiv) was dissolved in Et_3N (1.0 mL). Then water was added, and the reaction mixture was extracted with EtOAc, dried over $MgSO_4$ and concentrated under reduced pressure. The crude product was purified by flash column chromatography. The crude product was purified by flash column chromatography on silica gel (toluene/PE = 1:1, then EA/PE = 20:1) to afford 10.2 mg (11%) of **1c** and 18.0 mg (17%) of **36** as a colorless oil.

A 10.0 mL Schlenk tube with a stirring bar was added with **1a** (188.2 mg, 0.4 mmol), B_2cat_2 (190.3 mg, 0.8 mmol, 2.0 equiv), **B1** (26.0 mg, 30.0 mol %), TEMPO (0.8 mmol, 125.0 mg) and DMA (1.0 mL) under argon. The resulting mixture was stirred at 80 °C under Ar for 3 h. The mixture was cooled to room temperature. Then pinacol (189.1 mg, 1.6 mmol, 4.0 equiv) was dissolved in Et_3N (1.0 mL). Then water was added, and the reaction mixture was extracted with EtOAc, dried over $MgSO_4$ and concentrated under reduced pressure. The crude product was purified by flash column chromatography. The crude product was purified by flash column chromatography on silica gel (toluene/PE = 1:1, then EA/PE = 20:1) to afford 19.5 mg (21%) of **1c** and 20.0 mg (19%) of **36** as a colorless oil.

2,2,6,6-Tetramethyl-1-phenethoxypiperidine (36)

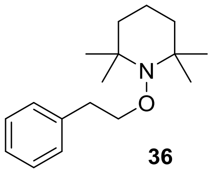
36

^{1}H NMR (400 MHz, CDCl$_3$) δ 7.29 – 7.17 (m, 5H), 3.94 (t, J = 7.0 Hz, 2H), 2.82 (t, J = 7.0 Hz, 2H), 1.41 (d, J = 5.6 Hz, 4H), 1.33 – 1.19 (m, 2H), 1.07 (s, 12H); **^{13}C NMR (101 MHz, CDCl$_3$)** δ 139.6, 129.1, 128.1, 125.9, 77.5, 59.7, 39.6, 35.4, 32.9, 20.1, 17.1; **ATR-FTIR (cm^{-1}):** 2974, 2928, 1453, 1373, 1031, 904, 729; **HRMS m/z (ESI)**

calcd for $C_{17}H_{28}NO$ (M + H)$^+$ 262.2165, found 262.2161. All spectral data are in accordance with the literature.[20]

Radical trapping experiment with 1,1-diphenylethylene

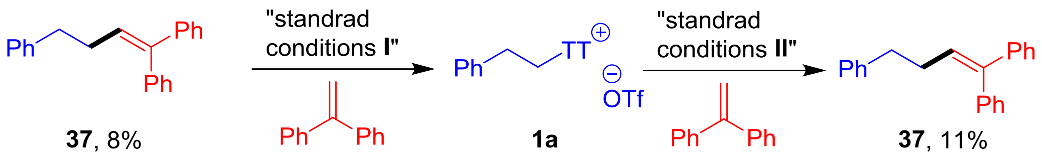

A 10.0 mL Schlenk tube with a stirring bar was added with **1a** (188.2 mg, 0.4 mmol), B_2cat_2 (190.3 mg, 0.8 mmol, 2.0 equiv), 1,1-diphenylethylene (0.8 mmol, 144.2 mg) and DMA (1.0 mL) under argon. The resulting mixture was stirred at room temperature under blue LED irradiation for 12 h. Then pinacol (189.1 mg, 1.6 mmol, 4.0 equiv) was dissolved in Et_3N (1.0 mL). Then water was added, and the reaction mixture was extracted with EtOAc, dried over $MgSO_4$ and concentrated under reduced pressure. The crude product was purified by flash column chromatography. The crude product was purified by flash column chromatography on silica gel with PE to afford 9.1 mg (8%) of **37** as a colorless oil.

A 10.0 mL Schlenk tube with a stirring bar was added with **1a** (188.2 mg, 0.4 mmol), B_2cat_2 (190.3 mg, 0.8 mmol, 2.0 equiv), **B1** (26.0 mg, 30.0 mol %), 1,1-diphenylethylene (0.8 mmol, 144.2 mg) and DMA (1.0 mL) under argon. The resulting mixture was stirred at 80 ^{o}C under Ar for 3 h. Then pinacol (189.1 mg, 1.6 mmol, 4.0 equiv) was dissolved in Et_3N (1.0 mL). Then water was added, and the reaction mixture was extracted with EtOAc, dried over $MgSO_4$ and concentrated under reduced pressure. The crude product was purified by flash column chromatography. The crude product was purified by flash column chromatography on silica gel with PE to afford 12.5 mg (11%) of **37** as a colorless oil.

2,2,6,6-Tetramethyl-1-phenethoxypiperidine (37)

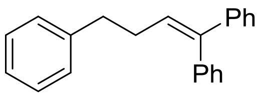

37

^{1}H NMR (400 MHz, $CDCl_3$) δ 7.36 – 7.16 (m, 11H), 7.13 (t, J = 6.2 Hz, 2H), 7.10 – 7.03 (m, 2H), 6.11 (t, J = 7.4 Hz, 1H), 2.74 (t, J = 7.7 Hz, 2H), 2.43 (q, J = 7.6 Hz, 2H); **^{13}C**

NMR (101 MHz, CDCl3) δ 142.6, 142.2, 141.7, 129.8, 128.8, 128.5, 128.3, 128.1, 128.1, 127.2, 126.9, 126.9, 125.8, 36.2, 31.6; **ATR-FTIR (cm $^{-1}$):** 2930, 2861, 1469, 907, 743; **HRMS m/z (ESI)** calcd for $C_{22}H_{21}$ $(M + H)^+$ 285.1638, found 285.1633. All spectral data are in accordance with the literature.[21]

Cyclization with Substrate 38

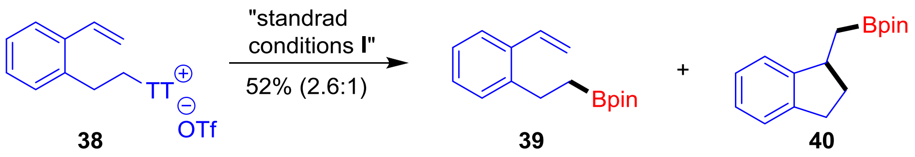

A 10.0 mL Schlenk tube with a stirring bar was added with **38** (198.6 mg, 0.4 mmol), B_2cat_2 (190.3 mg, 0.8 mmol) and DMA (1.0 mL) under argon. The resulting mixture was stirred at room temperature under blue LED irradiation for 12 h. Then pinacol (189.1 mg, 1.6 mmol, 4.0 equiv) was dissolved in Et_3N (1.0 mL). Then water was added, and the reaction mixture was extracted with EtOAc, dried over $MgSO_4$ and concentrated under reduced pressure. The crude product was purified by flash column chromatography on silica gel (toluene/PE = 1:1, then EA/PE = 20:1) to afford a 2.6:1 mixture of linear: cyclized boronic esters **39** and **40** (53.7 mg, 52%) as a colorless oil. **HRMS m/z (ESI)** calcd for $C_{16}H_{24}BO_2$ $(M + H)^+$ 259.1864, found 259.1862.

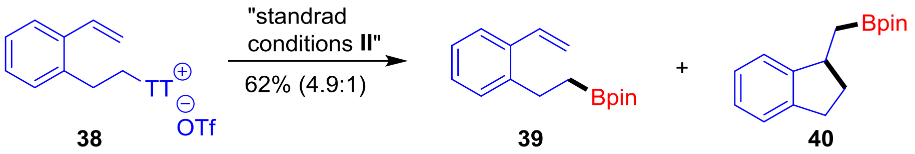

A 10.0 mL Schlenk tube with a stirring bar was added with **38** (198.6 mg, 0.4 mmol), B_2cat_2 (190.3 mg, 0.8 mmol), **B1** (26.0 mg, 30.0 mol %) and DMA (1.0 mL) under argon. The resulting mixture was stirred at 80 ^{o}C under Ar for 3 h. Then pinacol (189.1 mg, 1.6 mmol, 4.0 equiv) was dissolved in Et_3N (1.0 mL). Then water was added, and the reaction mixture was extracted with EtOAc, dried over $MgSO_4$ and concentrated under reduced pressure. The crude product was purified by flash column chromatography on silica gel (toluene/PE = 1:1, then EA/PE = 20:1) to afford a 4.9:1 mixture of linear: cyclized boronic esters **39** and **40** (64.0 mg, 62%) as a colorless oil.

HRMS m/z (ESI) calcd for $C_{16}H_{24}BO_2$ $(M + H)^+$ 259.1864, found 259.1863.

Linear boronic esters **38**

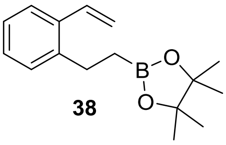

38

^{1}H NMR (400 MHz, CDCl$_3$) δ 7.50 – 7.44 (m, 1H), 7.24 – 7.10 (m, 3H), 7.03 (dd, J = 17.4, 11.0 Hz, 1H), 5.64 (dd, J = 17.4, 1.3 Hz, 1H), 5.28 (dd, J = 11.0, 1.3 Hz, 1H), 2.80 (t, J = 8.2, 2H), 1.24 (s, 12H), 1.10 (t, J = 8.2, 2H).

Cyclized boronic esters **39**

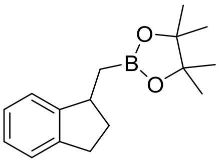

39

^{1}H NMR (400 MHz, CDCl$_3$) δ 7.23 – 7.09 (m, 4H), 3.31 (m, 1H), 2.88 (m, 2H), 2.36 (dtd, J = 11.7, 7.6, 3.9 Hz, 1H), 1.70 – 1.63 (m, 1H), 1.28 (s, 6H), 1.27 (s, 6H), 0.98 (dd, J = 15.6, 9.5 Hz, 2H). All spectral data are in accordance with the literature.[22]

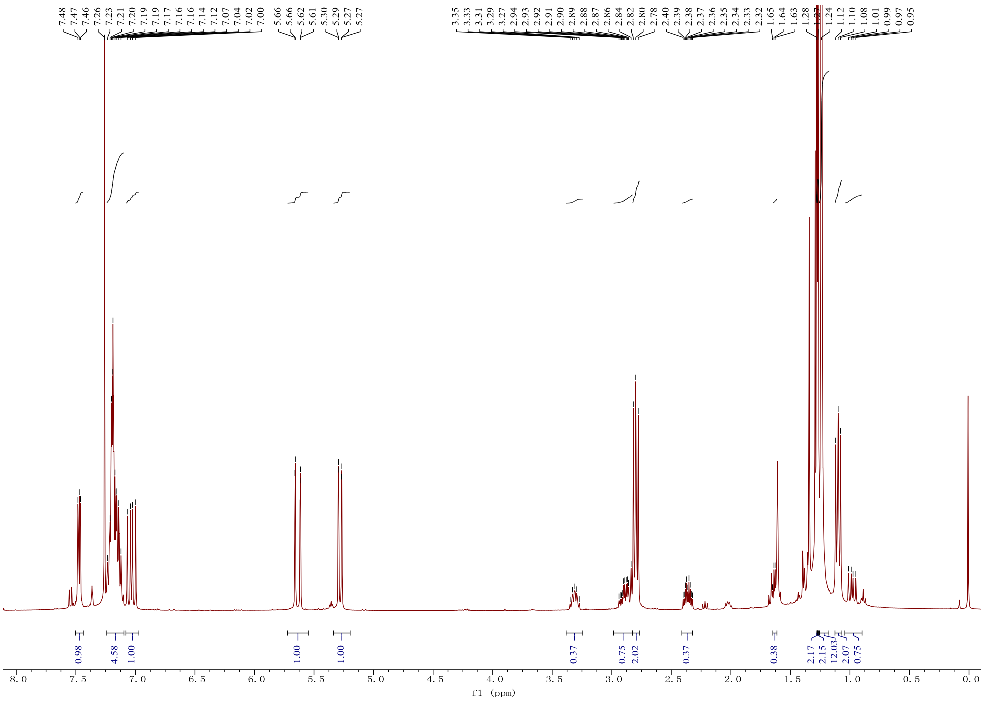

When the concentration was dilute to 0.01 M, the mixture of the linear: cyclized boronic esters was obtained in a ratio of ~1: 3.5 (**39:40**) under blue LED irradiation.

"light on/off" experiments

An 8.0 mL oven dried tube with a stirring bar was added with **1a** (188.2 mg, 0.4 mmol), B_2cat_2 (190.3 mg, 0.8 mmol), DMA (1.0 mL) and *n*-dodecane (120 μL) as the internal standard under argon. The resulting mixture was stirred at room temperature under blue LED irradiation or in dark. Take samples every ten minutes, which was

quenched and analyzed by GC.

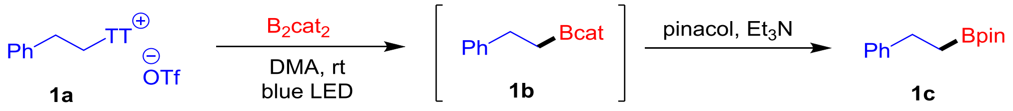

Yield of **1c** detected by GC-MS with *n*-dodecane as internal standard:

Time	10 min	20 min	30 min	40 min	50 min	60 min	12h
Yield	38%	41%	53%	54%	65%	66%	90%
Blue LED	on	off	on	off	on	off	on

Time	10 min	20 min	30 min	40 min	50 min	60 min	12h
Yield	33%	34%	35%	35%	35%	35%	35%
Blue LED	on	off	off	off	off	off	off

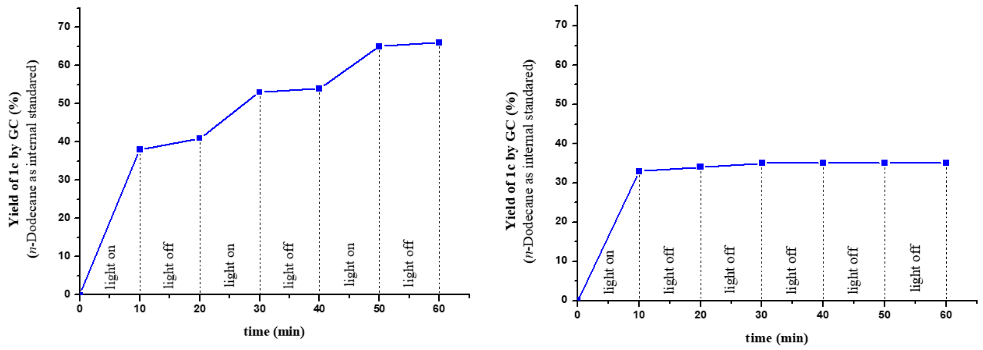

Supplementary Figure 8. "light on/off" experiments. "light on/off" experiments of photoinduced desulfurative borylation

The proposed mechanism of thermoinduced borylation:

Combined our previous studies on borylation of Katritzky salts, a proposed mechanism is outlined in Figure S5.[24] First, dMeObpy reacts with B_2Cat_2 to produce the ate complex (**I**). As our previous study, the **I** may combine the solvent DMAc and subsequent B-B bond cleavage to form bipyridinylidene derivative (**II**). The **II** may be a strong reductant, which reduces sulfonium triflates (**a**) via single electron transfer (SET), producing the alkyl radical intermediate (**b**). **II** can also provide stabilized boron radical, which combined the alkyl radical to provide the borylation product **b**.

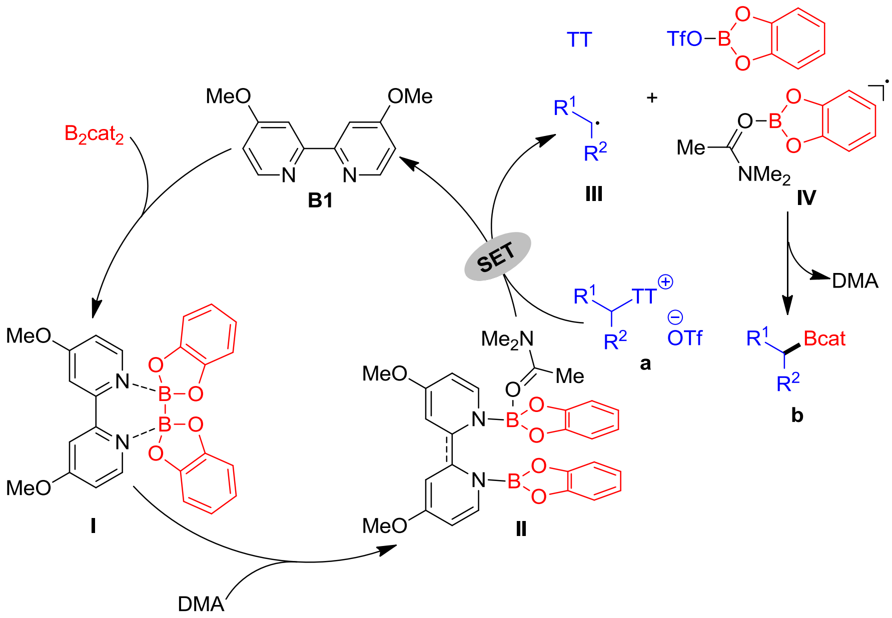

Supplementary Figure 9. Proposed mechanism. The proposed mechanism of thermoinduced borylation.

Diverse Transformations of Thianthrenium Salts

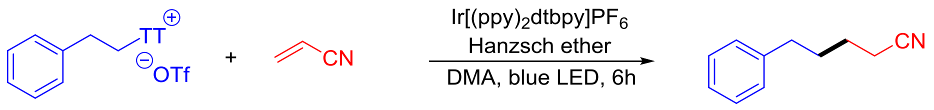

1a **41a** **42**, 72%

5-Phenylpentanenitrile (42)

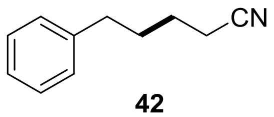

42

A 10.0 mL Schlenk tube with a stirring bar was added with **1a** (0.2 mmol, 94.1 mg), [Ir(dtbbpy)(ppy)$_2$]PF$_6$ (3.7 mg, 0.004 mmol) and hantzsch ether (0.4 mmol, 101.3 mg). Then acrylonitrile (0.6 mmol, 31.8 mg) and DMA (0.5 mL) were added under argon. The tube was placed in water bath and stirred under irradiation of a 40W blue LEDs at room temperature for 6 h. Ethyl acetate (30 mL) were added to the reaction mixture. The resulting solution was washed with water (20 mL) and brine (20 mL) and dried over MgSO$_4$. After filtration the solvent was removed under reduced pressure. The crude product was purified by flash column chromatography on silica gel (EA/PE = 1:5) to afford 23.0 mg (72%) of **42** as a corlorless oil: **^{1}H NMR (400 MHz, CDCl$_3$)** δ 7.30 (t, J = 7.3 Hz, 2H), 7.24 – 7.10 (m, 3H), 2.67 (t, J = 7.4 Hz, 2H), 2.35 (t, J = 7.0 Hz, 2H), 1.83 – 1.74 (m, 2H), 1.73 – 1.65 (m, 2H); **^{13}C NMR (101 MHz, CDCl$_3$)** δ

141.2, 128.4, 128.3, 126.0, 119.6, 34.9, 30.2, 24.8, 17.0; **ATR-FTIR (cm $^{-1}$):** 2934, 2860, 2249, 1453, 1029, 698; **HRMS m/z (ESI)** calcd for $C_{11}H_{14}N$ $(M + H)^+$ 160.1121, found 160.1120. All spectral data are in accordance with the literature.[22]

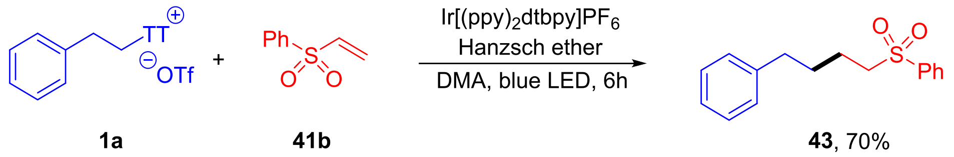

((4-Phenylbutyl)sulfonyl)benzene (43)

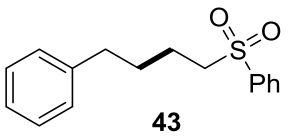

43

A 10.0 mL Schlenk tube with a stirring bar was added with **1a** (0.2 mmol, 94.1 mg), $[Ir(dtbbpy)(ppy)_2]PF_6$ (3.7 mg, 0.004 mmol) and hantzsch ether (0.4 mmol, 101.3 mg). Then (vinylsulfonyl)benzene (0.6 mmol, 100.9 mg) and DMA (0.5 mL) were added under argon. The tube was placed in water bath and stirred under irradiation of a 40W blue LEDs at room temperature for 6 h. Ethyl acetate (30 mL) were added to the reaction mixture. The resulting solution was washed with water (20 mL) and brine (20 mL) and dried over $MgSO_4$. After filtration the solvent was removed under reduced pressure. The crude product was purified by flash column chromatography on silica gel (EA/PE = 1:3) to afford 38.4 mg (70%) of **43** as a white solid: **^{1}H NMR (400 MHz, $CDCl_3$)** δ 7.97 – 7.81 (m, 2H), 7.65 (t, J = 7.4 Hz, 1H), 7.55 (t, J = 7.7 Hz, 2H), 7.25 (t, J = 7.4 Hz, 2H), 7.17 (t, J = 7.3 Hz, 1H), 7.09 (d, J = 7.2 Hz, 2H), 3.21 – 2.94 (m, 2H), 2.58 (t, J = 7.3 Hz, 2H), 1.80 – 1.65 (m, 4H); **^{13}C NMR (101 MHz, $CDCl_3$)** δ 141.2, 139.1, 133.6, 129.2, 128.4, 128.2, 128.0, 126.0, 56.0, 35.2, 29.9, 22.2; **ATR-FTIR (cm $^{-1}$):** 1306, 1149, 903, 772; **HRMS m/z (ESI)** calcd for $C_{16}H_{19}O_2S$ $(M + H)^+$ 275.1100, found 275.1097. All spectral data are in accordance with the literature.[25]

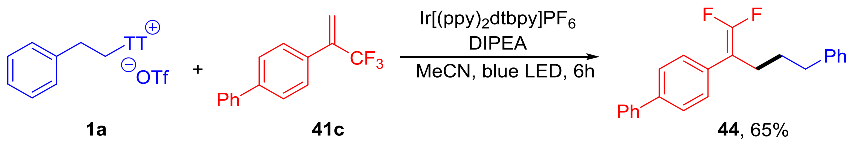

4-(1,1-Difluoro-5-phenylpent-1-en-2-yl)-1,1'-biphenyl (44)

A 10.0 mL Schlenk tube with a stirring bar was added with **1a** (0.10 mmol, 47.1

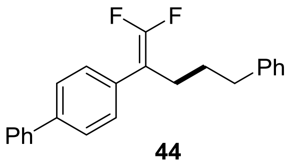

44

mg), [Ir(dtbbpy)(ppy)$_2$]PF_6 (1.8 mg, 0.002 mmol) and 4-(3,3,3-trifluoroprop-1-en-2-yl)-1,1'-biphenyl (0.15 mmol, 37.2 mg). Then DIPEA (0.3 mmol, 39.0 mg) and CH_3CN (0.5 mL) were added under argon. The tube was placed in water bath and stirred under irradiation of a 40W blue LEDs at room temperature for 6h. Ethyl acetate (30 mL) were added to the reaction mixture. The resulting solution was washed with water (20 mL) and brine (20 mL) and dried over $MgSO_4$. After filtration the solvent was removed under reduced pressure. The crude product was purified by flash column chromatography on silica gel (EA/PE = 1:30) to afford 21.7 mg (65%) of **44** as a colorless oil: **^{1}H NMR (400 MHz, $CDCl_3$)** δ 7.35 (t, J = 7.2 Hz, 2H), 7.31 – 7.15 (m, 8H), 5.92 (d, J = 10.2 Hz, 1H), 2.36 (dp, J = 11.9, 6.2 Hz, 1H), 1.51 (m, 2H), 1.33 – 0.93 (m, 20H); **^{13}C NMR (101 MHz, $CDCl_3$)** δ 153.7 (dd, J = 288.7, 285.5 Hz), 141.8, 140.6, 140.0, 132.5 (dd, J = 3.4, 2.6 Hz), 128.8, 128.6 (t, J = 3.3 Hz), 128.4, 128.3, 127.4, 127.1, 127.0, 125.8, 91.9 (dd, J = 21.0, 13.5 Hz), 35.2, 29.4 (t, J = 2.4 Hz), 27.1; **^{19}F NMR (376 MHz, $CDCl_3$)** δ -90.8 (d, J = 43.9 Hz), -91.0 (d, J = 43.8 Hz). **ATR-FTIR (cm $^{-1}$):** 3028, 2928, 1723, 1233, 905, 696; **HRMS m/z (ESI)** calcd for $C_{23}H_{20}F_2Na$ $(M + Na)^+$ 357.1425, found 357.1422.

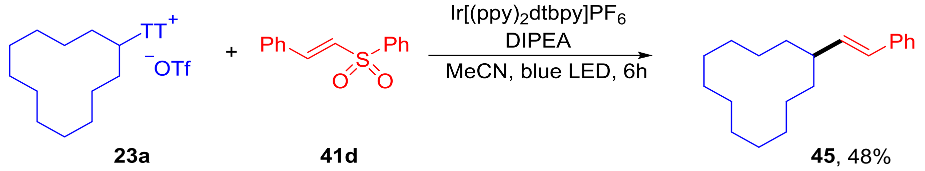

(*E*)-styrylcyclododecane (45)

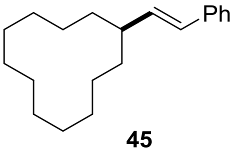

45

A 10.0 mL Schlenk tube with a stirring bar was added with **23a** (0.20 mmol, 106.5 mg), [Ir(dtbbpy)(ppy)$_2$]PF_6 (1.8 mg, 0.002 mmol) and (*E*)-(2-(phenylsulfonyl)vinyl)benzene (0.30 mmol, 73.3 mg). Then DIPEA (0.6 mmol, 77.5 mg) and CH_3CN (1.0 mL) were added under argon. The tube was placed in water bath and stirred under irradiation of a 40W blue LEDs at room temperature for 6h. Ethyl acetate (30 mL) were added to the reaction mixture. The resulting solution was washed with water (20 mL) and brine (20 mL) and dried over $MgSO_4$. After filtration the solvent was removed under reduced

pressure. The crude product was purified by flash column chromatography on silica gel with PE to afford 26.0 mg (48%) of **45** as a white solid: **^{1}H NMR (400 MHz, $CDCl_3$)** δ 7.37 (d, J = 7.3 Hz, 2H), 7.30 (t, J = 7.6 Hz, 2H), 7.19 (t, J = 7.2 Hz, 1H), 6.36 (d, J = 15.9 Hz, 1H), 6.12 (dd, J = 15.9, 8.1 Hz, 1H), 2.36 (h, J = 6.5 Hz, 1H), 1.60 (dt, J = 13.3, 5.8 Hz, 2H), 1.47 – 1.33 (m, 20H); **^{13}C NMR (101 MHz, $CDCl_3$)** δ 138.0, 136.6, 128.4, 128.2, 126.7, 125.9, 37.6, 30.0, 26.2, 23.8, 23.8, 23.4, 22.3; **ATR-FTIR (cm^{-1}):** 2927, 2859, 1468, 962, 745; **HRMS m/z (ESI)** calcd for $C_{20}H_{31}$ $(M + H)^+$ 271.2420, found 271.2416. All spectral data are in accordance with the literature.[27]

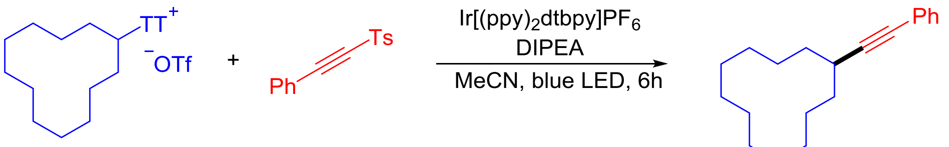

(Phenylethynyl)cyclododecane (46)

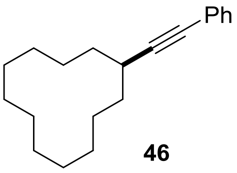

A 10.0 mL Schlenk tube with a stirring bar was added with **23a** (0.20 mmol, 106.5 mg), [Ir(dtbbpy)(ppy)$_2$]PF_6 (1.8 mg, 0.002 mmol) and 1-methyl-4-((phenylethynyl)sulfonyl)benzene (0.30 mmol, 76.8 mg). Then DIPEA (0.6 mmol, 77.5 mg) and CH_3CN (1.0 mL) were added under argon. The tube was placed in water bath and stirred under irradiation of a 40W blue LEDs at room temperature for 6h. Ethyl acetate (30 mL) were added to the reaction mixture. The resulting solution was washed with water (20 mL) and brine (20 mL) and dried over $MgSO_4$. After filtration the solvent was removed under reduced pressure. The crude product was purified by flash column chromatography on silica gel with PE to afford 41.0 mg (76%) of **46** as a white solid: **^{1}H NMR (400 MHz, $CDCl_3$)** δ 7.38 (dd, J = 7.6, 2.0 Hz, 2H), 7.31 – 7.18 (m, 3H), 2.68 (ddd, J = 12.2, 7.1, 5.1 Hz, 1H), 1.69 (m, 2H), 1.57 (m, 4H), 1.47 – 1.29 (m, 16H); **^{13}C NMR (101 MHz, $CDCl_3$)** δ 131.6, 128.1, 127.3, 124.2, 94.9, 80.2, 29.9, 27.4, 23.9, 23.8, 23.5, 23.4, 22.2; **ATR-FTIR (cm^{-1}):** 2929, 2861, 1489, 1469, 905, 690; **HRMS m/z (ESI)** calcd for $C_{20}H_{29}$ $(M + H)^+$ 269.2264, found 269.2261. All spectral data are in accordance

with the literature.[28]

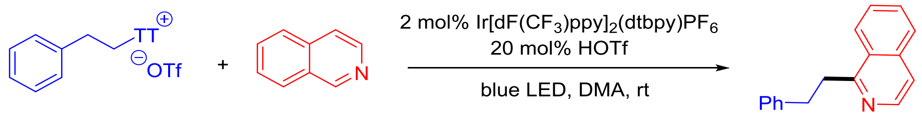

1a **41f** **47**, 75%

1-Phenethylisoquinoline (47)

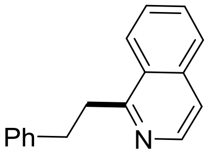

47

A 10.0 mL Schlenk tube with a stirring bar was added with **1a** (0.4 mmol, 188.2 mg), Ir[dF(CF_3)ppy]$_2$(dtbpy)PF_6 Ir[dF(CF_3)ppy]$_2$(dtbbpy)PF_6 (4.5 mg, 0.004 mmol) and DMA (2.0 mL) under argon. Then, isoquinoline (0.20 mmol, 25.8 mg) and Trifluoromethanesulfonic acid (0.04 mmol, 0.2 equiv) was added via syringe under argon atmosphere. The tube was placed in water bath and stirred under irradiation of a 40W blue LEDs at room temperature for 5h. Triethylamine (0.3 mL) and ethyl acetate (30 mL) were added to the reaction mixture. The resulting solution was washed with water (20 mL) and brine (20 mL) and dried over $MgSO_4$. After filtration the solvent was removed under reduced pressure. The crude product was purified by flash column chromatography on silica gel (EA/PE = 1:5) to afford 35.0 mg (75%) of **47** as a yellowish oil: **^{1}H NMR (400 MHz, CDCl$_3$)** δ 8.48 (d, J = 5.7 Hz, 1H), 8.16 (d, J = 8.5 Hz, 1H), 7.83 (d, J = 8.2 Hz, 1H), 7.71 – 7.64 (m, 1H), 7.59 (ddd, J = 8.2, 6.9, 1.2 Hz, 1H), 7.54 (d, J = 5.7 Hz, 1H), 7.23 (dt, J = 8.8, 4.4 Hz, 1H), 3.65 – 3.57 (m, 2H), 3.25 – 3.18 (m, 2H); **^{13}C NMR (101 MHz, CDCl$_3$)** δ 161.0, 141.8, 136.2, 129.8, 128.4, 127.4, 127.1, 126.0, 125.0, 37.2, 35.5; **ATR-FTIR (cm $^{-1}$):** 3051, 3025, 2924, 1561, 1495, 821, 742, 698; **HRMS m/z (ESI)** calcd for $C_{17}H_{16}N$ $(M + H)^+$ 234.1277, found 234.1277. All spectral data are in accordance with the literature.[26]

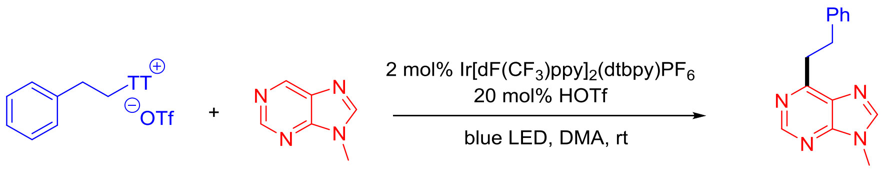

1a **41g** **48**, 67%

9-Methyl-6-phenethyl-9*H*-purine (48)

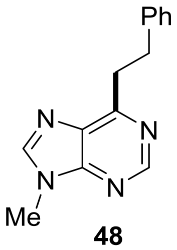

48

A 10.0 mL Schlenk tube with a stirring bar was added with **1a** (0.3 mmol, 141.2 mg), Ir[dF(CF_3)ppy]$_2$(dtbbpy)PF_6 (4.5 mg, 0.004 mmol), 9-methyl-9*H*-purine (0.20 mmol, 26.8 mg) and DMA (2.0 mL) under argon. Then trifluoromethanesulfonic acid (0.04 mmol, 0.2 equiv) was added via syringe under argon atmosphere. The tube was placed in water bath and stirred under irradiation of a 40W blue LEDs at room temperature for 5h. Triethylamine (0.3 mL) and ethyl acetate (30 mL) were added to the reaction mixture. The resulting solution was washed with water (20 mL) and brine (20 mL) and dried over $MgSO_4$. After filtration the solvent was removed under reduced pressure. The crude product was purified by flash column chromatography on silica gel with EA to afford 32.0 mg (67%) of **48** as a corlorless oil: **^{1}H NMR (400 MHz, CD_3CN)** δ 8.80 (s, 1H), 8.11 (s, 1H), 7.23 (d, J = 4.5 Hz, 4H), 7.15 (dd, J = 9.4, 3.6 Hz, 1H), 3.80 (s, 3H), 3.43 (dd, J = 8.9, 6.7 Hz, 2H), 3.22 (dd, J = 8.9, 6.7 Hz, 2H); **^{13}C NMR (101 MHz, CD_3CN)** δ 161.6, 152.8, 146.5, 142.6, 129.4, 129.3, 126.9, 35.2, 34.4, 30.2; **ATR-FTIR (cm $^{-1}$):** 3091, 2357, 1595, 1330, 1193, 1037, 688; **HRMS m/z (ESI)** calcd for $C_{14}H_{15}N_4$ $(M + H)^+$ 239.1291, found 239.1290.

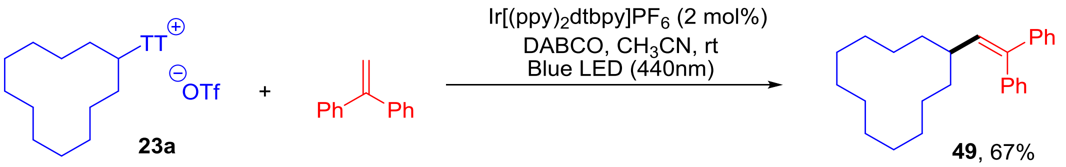

(2,2-Diphenylvinyl)cyclododecane (49)

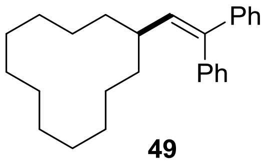

49

A 10.0 mL Schlenk tube with a stirring bar was added with **23a** (0.2 mmol, 106.5 mg), [Ir(dtbbpy)(ppy)$_2$]PF_6 (3.7 mg, 0.004 mmol), DABCO (0.2 mmol, 22.4 mg). Then 1,1-diphenylethylene (0.3 mmol, 54.1 mg) and MeCN (2.0 mL) were added under argon. The tube was placed in water bath and stirred under irradiation of a 40W blue LEDs at room temperature for 6h. Ethyl acetate (30 mL) were added to the reaction mixture. The resulting solution was washed with water (20 mL) and brine (20 mL) and dried over $MgSO_4$. After filtration the solvent was removed under reduced pressure. The crude product was purified by flash column

chromatography on silica gel (PE) to afford 46.4 mg (67%) of **49** as a corlorless oil: **^{1}H NMR (400 MHz, $CDCl_3$)** δ 7.35 (t, J = 7.2 Hz, 2H), 7.31 – 7.15 (m, 8H), 5.92 (d, J = 10.2 Hz, 1H), 2.36 (dp, J = 11.9, 6.2 Hz, 1H), 1.51 (m, 2H), 1.33 – 0.93 (m, 20H); **^{13}C NMR (101 MHz, $CDCl_3$)** δ 136.0, 129.830, 128.1, 128.0, 126.9, 126.7, 126.7, 33.5, 30.8, 23.9, 23.7, 23.16, 22.5; **HRMS m/z (ESI)** calcd for $C_{26}H_{35}$ $(M + H)^+$ 347.2433, found 347.2436. All spectral data are in accordance with the literature.[31]

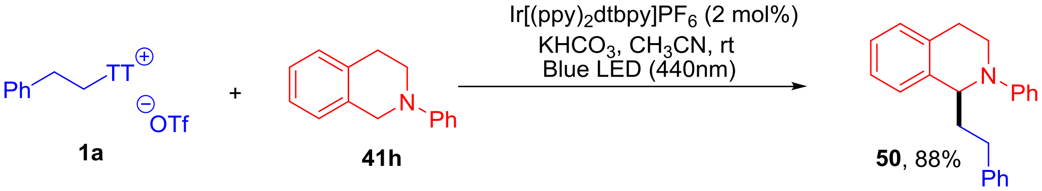

1-Phenethyl-2-phenyl-1,2,3,4-tetrahydroisoquinoline (50)

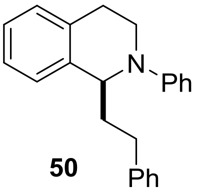

50

A 10.0 mL Schlenk tube with a stirring bar was added with **1a** (188.2 mg, 0.4 mmol), **41h** (42.0 mg, 0.2 mmol) $[Ir(dtbbpy)(ppy)_2]PF_6$ (3.7 mg, 0.004 mmol), $KHCO_3$ (0.6 mmol, 60.1 mg) and MeCN (1.0 mL). The tube was placed in water bath and stirred under irradiation of a 40W blue LEDs at room temperature for 6h. Then the solvent was removed under reduced pressure. The crude product was purified by flash column chromatography on silica gel (PE:EA = 10:1) to afford 55.2 mg (88%) of **50** as a corlorless oil: **^{1}H NMR (400 MHz, $CDCl_3$)** δ 7.27 – 7.21 (m, 3H), 7.20 – 7.10 (m, 8H), 6.82 (d, J = 8.5 Hz, 2H), 6.71 (t, J = 7.2 Hz, 1H), 4.67 (t, J = 7.0 Hz, 1H), 3.62 (dd, J = 6.9, 5.3 Hz, 2H), 3.00 (m, 1H), 2.83 – 2.70 (m, 3H), 2.26 (m, 1H), 2.04 (m, 1H); **^{13}C NMR (101 MHz, $CDCl_3$)** δ 149.6, 141.9, 138.7, 135.0, 129.2, 128.6, 128.5, 128.3, 127.2, 126.5, 125.8, 117.3, 114.2, 58.4, 41.8, 38.3, 32.9, 26.8; **HRMS m/z (ESI)** calcd for $C_{23}H_{24}N$ $(M + H)^+$ 314.1903, found 314.1903. All spectral data are in accordance with the literature.[32]

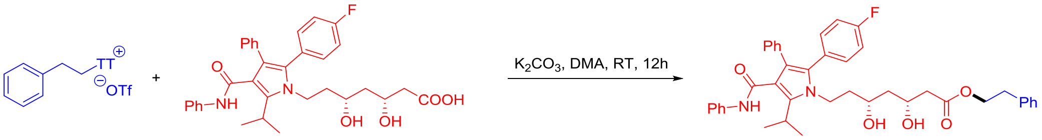

Phenethyl (3*R*,5*R*)-7-(2-(4-fluorophenyl)-5-isopropyl-3-phenyl-4-(phenylcarbamoyl)-1*H*-pyrrol-1-yl)-3,5-dihydroxyheptanoate (52)

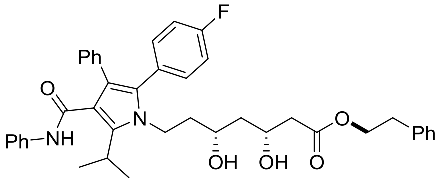

52

A 10.0 mL Schlenk tube with a stirring bar was added with atorvastatin (0.20 mmol, 111.7 mg), K_2CO_3 (0.40 mmol, 55.3 mg) and THF (1.0 mL) were added under argon. After stirring for 15 min, **1a** (0.20 mmol, 94.1 mg) was added. The mixture at room temperature was stired for 12h. The solvent was removed under reduced pressure. The crude product was purified by flash column chromatography on silica gel (MeOH/DCM = 1:20) to afford 107.4 mg (81%) of **52** as a colorless oil: **^{1}H NMR (400 MHz, $CDCl_3$)** δ 7.29 (m, 2H), 7.24 – 7.11 (m, 12H), 7.06 (d, J = 7.8 Hz, 2H), 7.02 – 6.93 (m, 3H), 6.88 (s, 1H), 4.32 (t, J = 7.0 Hz, 2H), 4.10 (tt, J = 10.5, 5.3 Hz, 2H), 3.93 (ddd, J = 14.6, 10.5, 5.6 Hz, 1H), 3.68 (m, 3H), 3.57 (p, J = 7.4 Hz, 1H), 3.00 – 2.90 (m, 3H), 2.44 – 2.29 (m, 2H), 1.66 (m, 2H), 1.53 (d, J = 7.1 Hz, 6H), 1.43 (m, 1H); **^{13}C NMR (101 MHz, $CDCl_3$)** δ 172.2, 164.8, 162.2 (d, J = 247.8 Hz), 141.4, 138.3, 134.6, 133.1 (d, J = 8.1 Hz), 130.4, 128.7, 128.6, 128.5, 128.3, 126.7, 126.5, 123.5, 119.5, 115.3 (d, J = 21.3 Hz), 69.5, 68.8, 65.2, 41.7, 41.4, 41.2, 39.0, 34.9, 26.1, 21.7, 21.6; **^{19}F NMR (376 MHz, $CDCl_3$)** δ -113.6. **ATR-FTIR (cm $^{-1}$):** 3047, 2979, 1267, 1054, 1027, 727, 699; **HRMS m/z (ESI)** calcd for $C_{41}H_{44}FN_2O_5$ $(M + H)^+$ 663.3229, found 663.3228.

Supplementary References

1. Liu, B.; Shine, H. J., Reactions of 5-(Alkyl)thianthrenium and Other sulfonium Salts with Nucleophiles. *J. Phys. Org. Chem.* **14**, 81-89 (2001).
2. Mohammadpoor-Baltork, I.; Khosropour, A. R.; Aliyan, H., A Convenient and Chemoselective Acetylation and Formylation of Alcohols and Phenols Using Acetic acid and Ethyl formate in The Presence of Bi(III) salts. *J. Chem Res-S* 280-282 (2001).
3. Simko, D. C.; Elekes, P.; Pazmandi, V.; Novak, Z., Sulfonium Salts as Alkylating Agents for Palladium-catalyzed Direct Ortho Alkylation of Anilides and Aromatic Ureas. *Org. Lett.* **20**, 676-679 (2018).
4. Wu, J.; He, L.; Noble, A.; Aggarwal, V. K., Photoinduced Deaminative Borylation of Alkylamines. *J. Am. Chem. Soc.* **140**, 10700-10704 (2018).
5. Mu, X.; Axtell, J. C.; Bernier, N. A.; Kirlikovali, K. O.; Jung, D.; Umanzor, A.; Qian, K.; Chen, X.; Bay, K. L.; Kirollos, M.; Rheingold, A. L.; Houk, K. N.; Spokoyny, A. M., Sterically Unprotected Nucleophilic Boron Cluster Reagents. *Chem* **5**, 2461-2469 (2019).
6. Zhang, L.; Zuo, Z.; Leng, X.; Huang, Z., A Cobalt-Catalyzed Alkene Hydroboration with Pinacolborane. *Angew. Chem. Int. Ed.* **53**, 2696-2700 (2014).
7. Hu, D.; Wang, L.; Li, P., Decarboxylative Borylation of Aliphatic Esters under Visible-Light Photoredox Conditions. *Org. Lett.* **19**, 2770-2773 (2017).
8. Fawcett, A.; Pradeilles, J.; Wang, Y.; Mutsuga, T.; Myers, E. L.; Aggarwal, V. K., Photoinduced Decarboxylative Borylation of Carboxylic Acids. *Science* **357**, 6348 (2017).
9. Bismuto, A.; Cowley, M. J.; Thomas, S. P., Aluminum-Catalyzed Hydroboration of Alkenes. *ACS Catalysis* **8**, 2001-2005 (2018).
10. Sandfort, F.; Strieth-Kalthoff, F.; Klauck, F. J. R.; James, M. J.; Glorius, F., Deaminative Borylation of Aliphatic Amines Enabled by Visible Light Excitation of an Electron Donor-Acceptor Complex. *Chem. Eur. J.* **24**, 17210-17214 (2018).
11. Lee, Y.; Hoveyda, A. H., Efficient Boron−Copper Additions to Aryl-Substituted

Alkenes Promoted by NHC−Based Catalysts. Enantioselective Cu-Catalyzed Hydroboration Reactions. *J. Am. Chem. Soc.* **131**, 3160-3161 (2009).

12. Zhang, L.; Wu, Z.-Q.; Jiao, L., Photoinduced Radical Borylation of Alkyl Bromides Catalyzed by 4-Phenylpyridine. *Angew. Chem. Int. Ed.* **59**, 2095-2099 (2020).
13. Cheng, Y.; Mueck-Lichtenfeld, C.; Studer, A., Metal-Free Radical Borylation of Alkyl and Aryl Iodides. *Angew. Chem. Int. Ed.* **57**, 16832-16836 (2018).
14. Atack, T. C.; Cook, S. P., Manganese-Catalyzed Borylation of Unactivated Alkyl Chlorides. *J. Am. Chem. Soc.* **138**, 6139-6142 (2016).
15. Melanson, J. A.; Vogels, C. M.; Decken, A.; Westcott, S. A., Catalytic Hydroboration of vinylarenes Using a Zwitterionic Arylspiroboronate Ester Iridium Complex. *Inorg. Chem. Commun.* **13**, 1396-1398 (2010).
16. Levin, V. V.; Zemtsov, A. A.; Struchkova, M. I.; Dilman, A. D., Reactions of Difluorocarbene with Organozinc Reagents. *Org. Lett.* **15**, 917-919 (2013).
17. Mills, L. R.; Zhou, C.; Fung, E.; Rousseaux, S. A. L., Ni-Catalyzed β-Alkylation of Cyclopropanol-Derived Homoenolates. *Org. Lett.* **21**, 8805-8809 (2019).
18. Cismesia, M. A.; Yoon, T. P., Characterizing chain processes in visible light photoredox catalysis. *Chem. Sci.* **6**, 5426-5434 (2015).
19. Hatchard, C. G.; Parker, C. A.; Bowen, E. J., A new sensitive chemical actinometer - II. Potassium ferrioxalate as a standard chemical actinometer. *Proc. R. Soc. Lond. A*. **235**, 518-536 (1956).
20. Wang, T.; Wang, D.-H., Potassium Alkylpentafluorosilicates, Primary Alkyl Radical Precursors in the C-1 Alkylation of Tetrahydroisoquinolines. *Org. Lett.* **21**, 3981-3985 (2019).
21. Liwosz, T. W.; Chemler, S. R., Copper-Catalyzed Oxidative Heck Reactions between Alkyltrifluoroborates and Vinyl Arenes. *Org. Lett.* **15**, 3034-3037 (2013).
22. Bose, S. K.; Marder, T. B., Efficient Synthesis of Aryl Boronates via Zinc-Catalyzed Cross-Coupling of Alkoxy Diboron Reagents with Aryl Halides at Room Temperature. *Org. Lett.* **16**, 4562-4565 (2014).
23. Hu, J.; Wang, G.; Li, S.; Shi, Z., Selective C-N Borylation of Alkyl Amines

Promoted by Lewis Base. *Angew. Chem. Int. Ed.* **57**, 15227-15231 (2018).

24. Vechorkin, O.; Proust, V.; Hu, X., Functional Group Tolerant Kumada−Corriu−Tamao Coupling of Nonactivated Alkyl Halides with Aryl and Heteroaryl Nucleophiles: Catalysis by a Nickel Pincer Complex Permits the Coupling of Functionalized Grignard Reagents. *J. Am. Chem. Soc.* **131**, 9756-9766 (2009).
25. Wang, J.-J.; Yu, W., Hydrosulfonylation of Unactivated Alkenes by Visible Light Photoredox Catalysis. *Org. Lett.* **21**, 9236-9240 (2019).
26. Jin, J.; MacMillan, D. W. C., Alcohols as alkylating agents in heteroarene C–H functionalization. *Nature* **525**, 87-90 (2015).
27. Ji, J.; Liu, P.; Sun, P., Peroxide promoted tunable decarboxylative alkylation of cinnamic acids to form alkenes or ketones under metal-free conditions. *Chem. Commun.* **51**, 7546-7549 (2015).
28. Ociepa, M.; Turkowska, J.; Gryko, D., Redox-Activated Amines in C(sp(3))-C(sp) and C(sp(3))-C(sp(2)) Bond Formation Enabled by Metal-Free Photoredox Catalysis. *ACS Catal.* **8**, 11362-11367 (2018).
29. Atack, T. C.; Lecker, R. M.; Cook, S. P., Iron-Catalyzed Borylation of Alkyl Electrophiles. *J. Am. Chem. Soc.* **136**, 9521-9523 (2014).
30. Lu, X.; Zhang, Z.-Q.; Yu, L.; Zhang, B.; Wang, B.; Gong, T.-J.; Tian, C.-L.; Xiao, B.; Fu, Y., Free Radical Pathway Cleavage of C—O Bonds for the Synthesis of Alkylboron Compounds. *Chin. J. Chem.* **37**, 11-18 (2019).
31. Jiang, X.; Zhang, M.-M.; Xiong, W.; Lu, L.-Q.; Xiao, W.-J., Deaminative (Carbonylative) Alkyl-Heck-type Reactions Enabled by Photocatalytic C−N Bond Activation. *Angew. Chem. Int. Ed.* **58**, 2402-2406 (2019).
32. Zhou, W.-J.; Cao, G.-M.; Shen, G.; Zhu, X.-Y.; Gui, Y.-Y.; Ye, J.-H.; Sun, L.; Liao, L.-L.; Li, J.; Yu, D.-G., Visible-Light-Driven Palladium-Catalyzed Radical Alkylation of C−H Bonds with Unactivated Alkyl Bromides. *Angew. Chem. Int. Ed.* **56**, 15683-15687 (2017).

Supplementary Copies of NMR Spectra

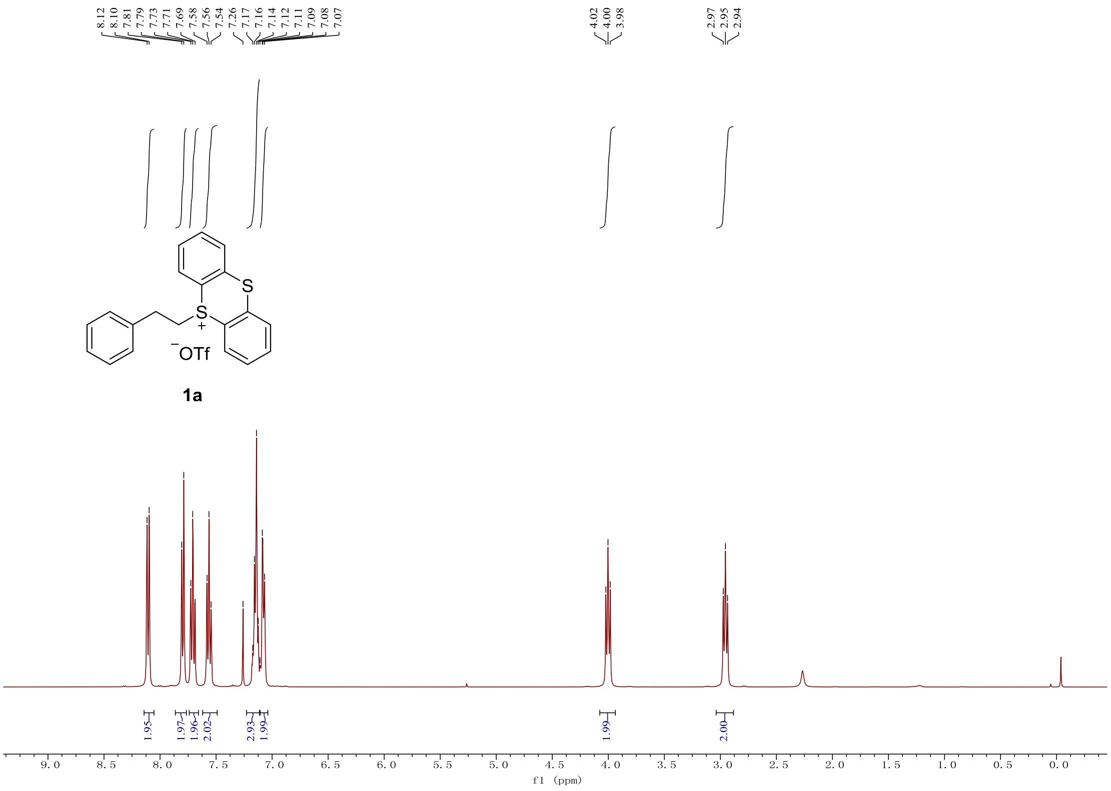

Supplementary Figure 10. ^{1}H NMR spectrum for 1a.

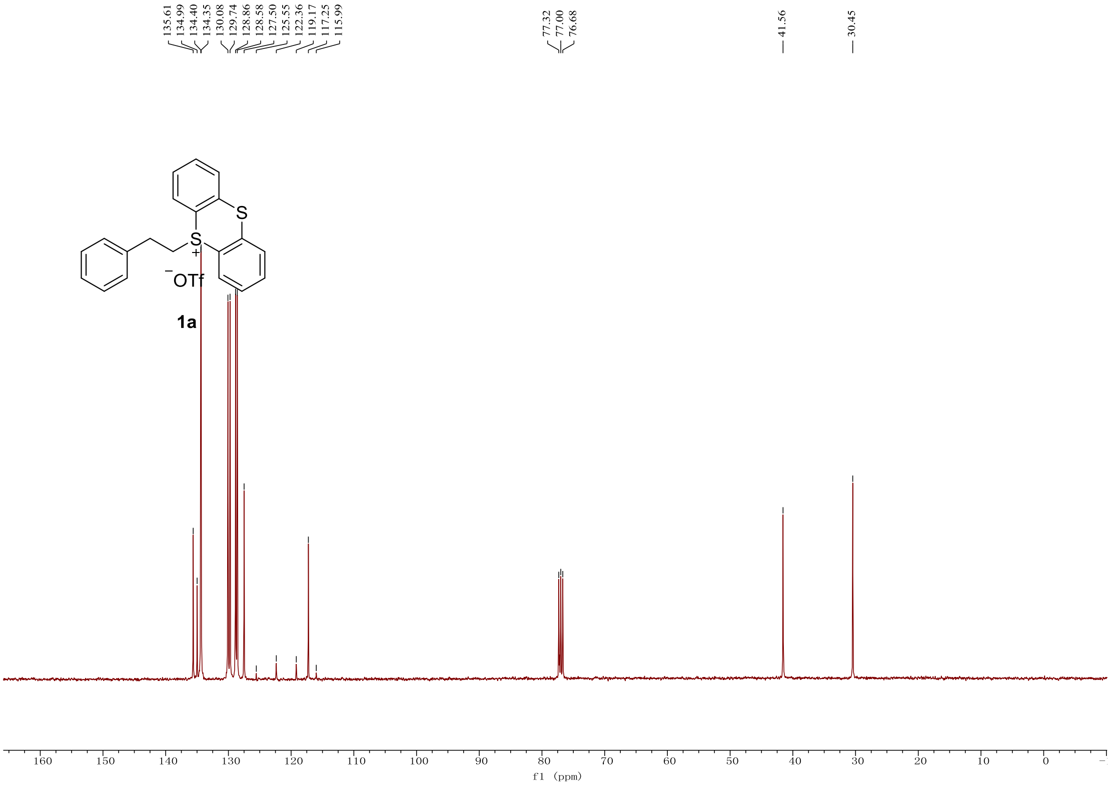

Supplementary Figures 11. ^{13}C NMR spectrum for 1a.

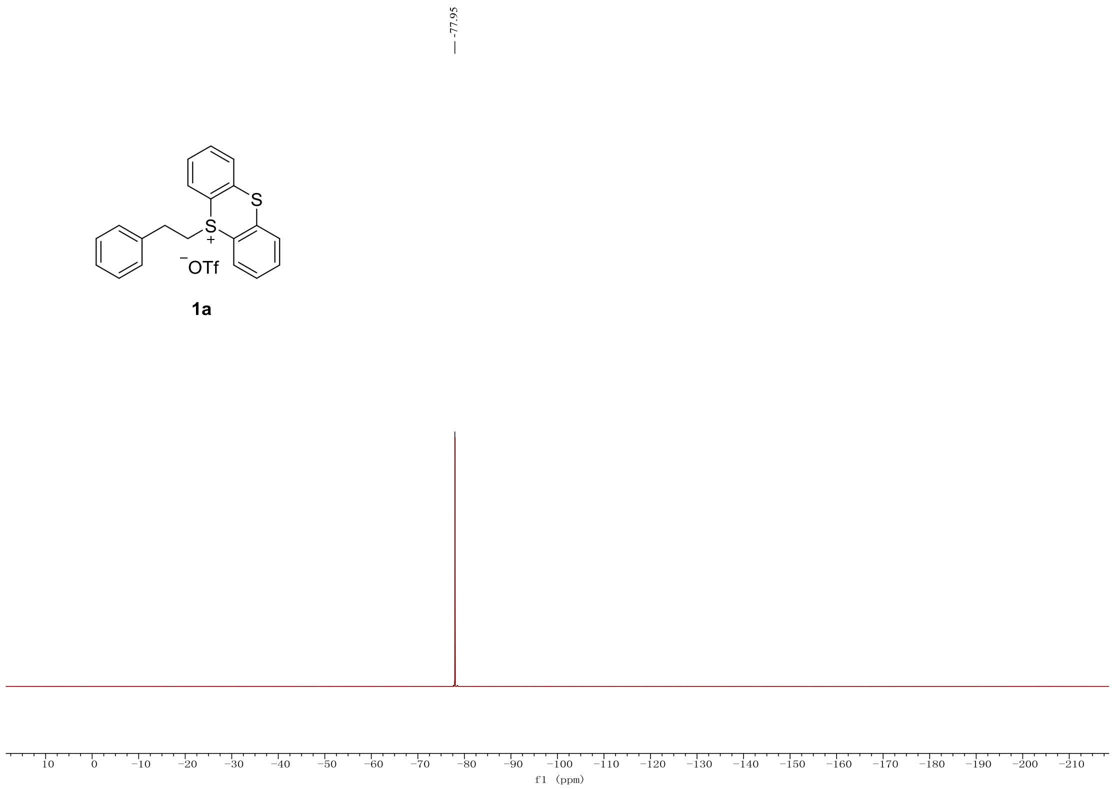

Supplementary Figure 12. ^{19}F NMR spectrum for 1a.

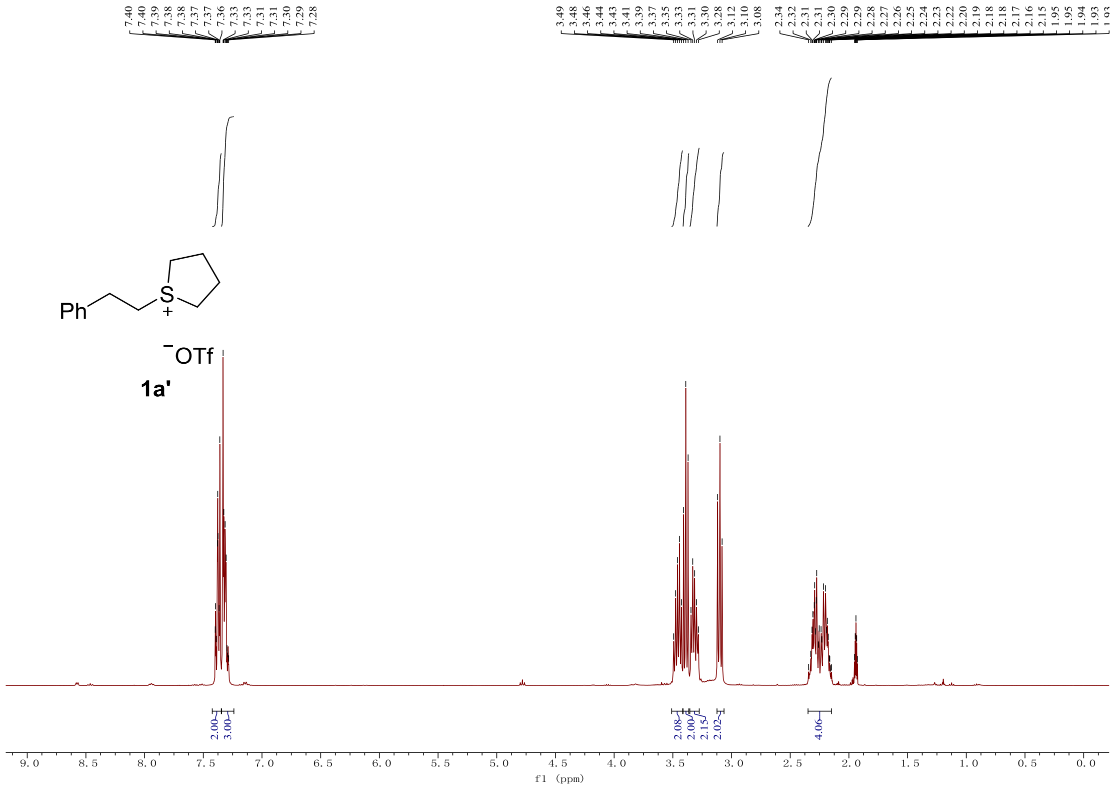

Supplementary Figure 13. ^{1}H NMR spectrum for 1a'.

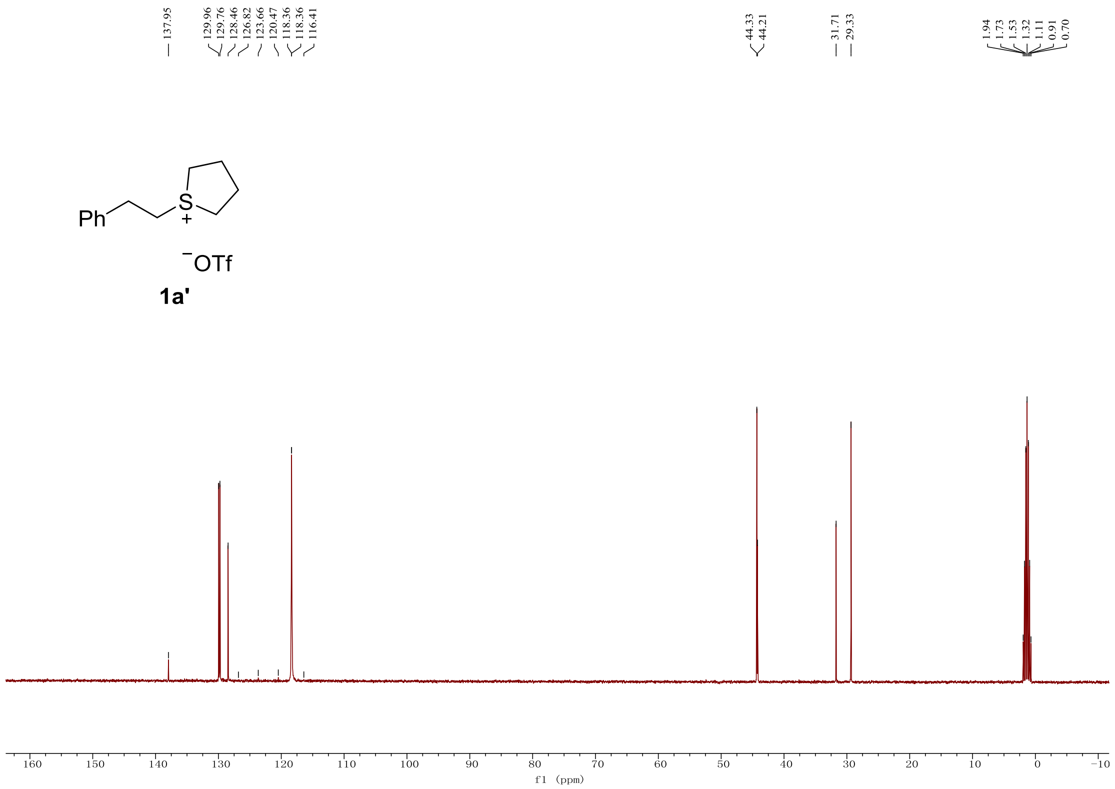

Supplementary Figures 14. ^{13}C NMR spectrum for 1a'.

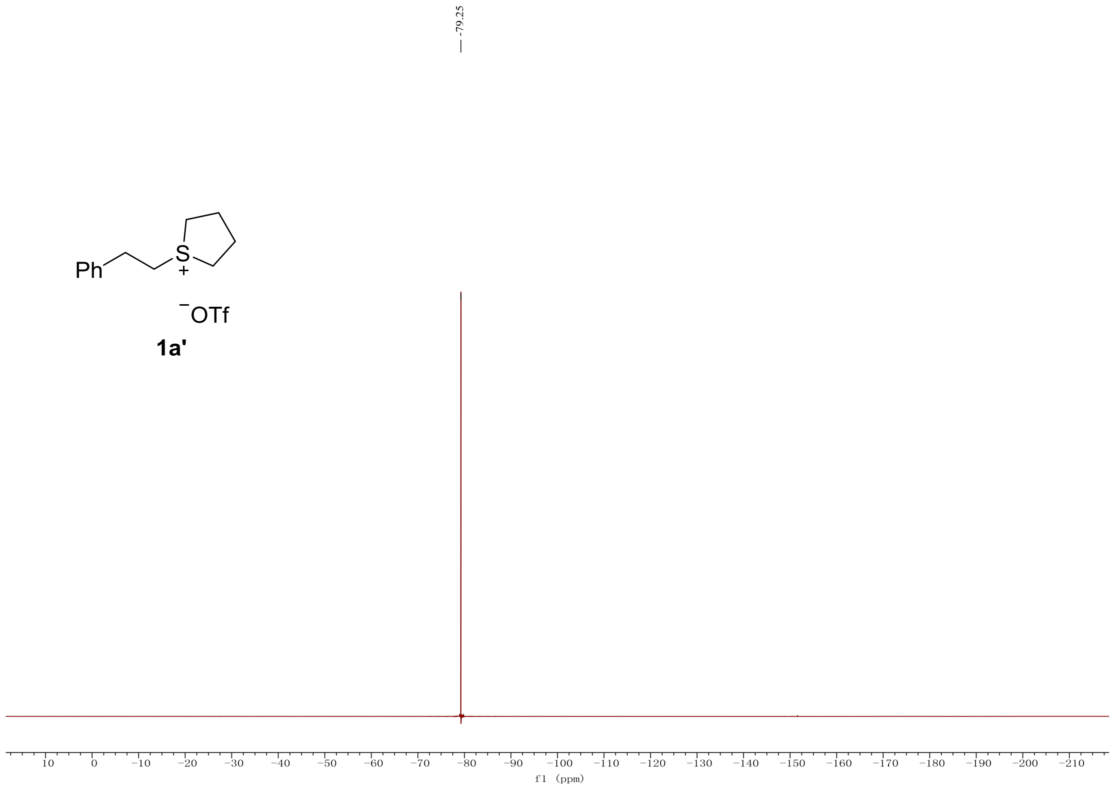

Supplementary Figure 15. ^{19}F NMR spectrum for 1a'.

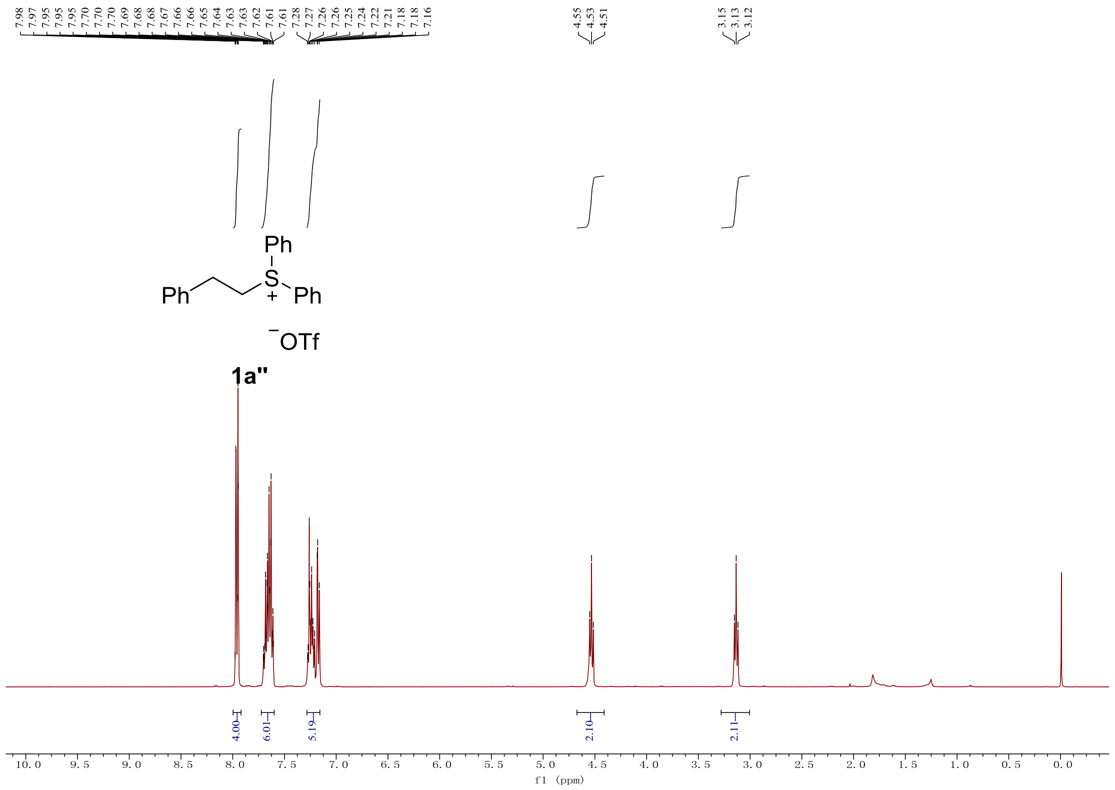

Supplementary Figure 16. ^{1}H NMR spectrum for 1a''.

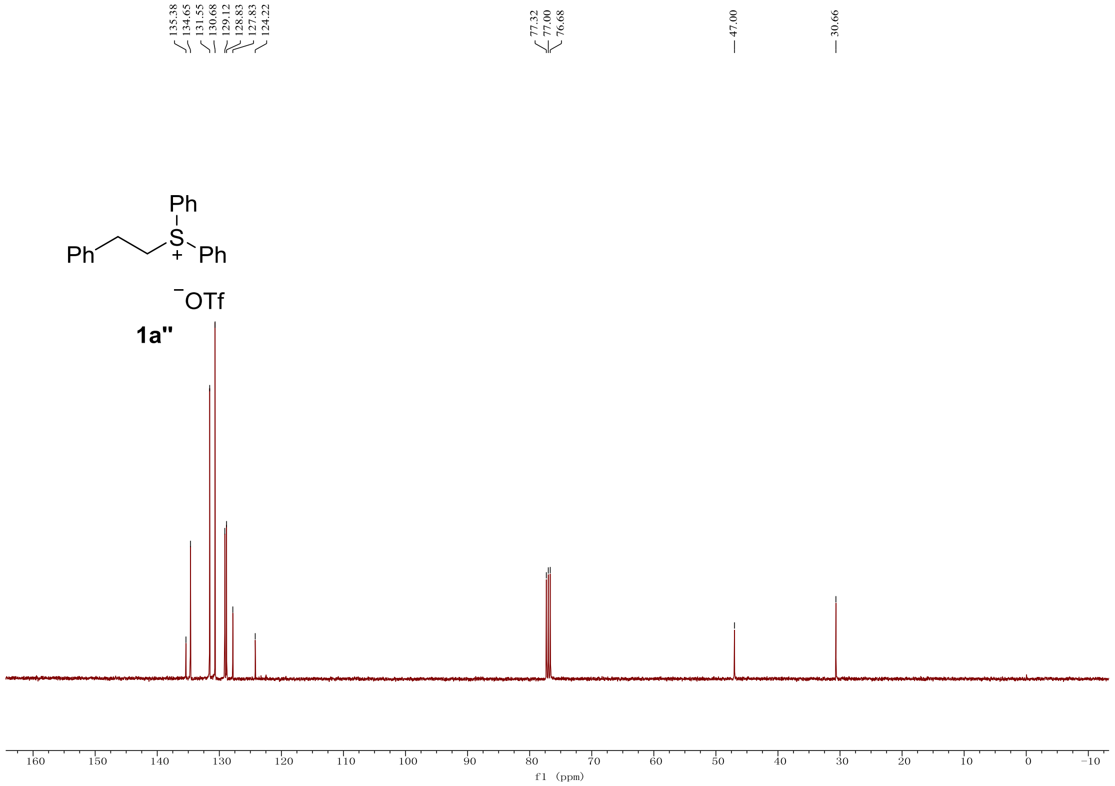

Supplementary Figures 17. ^{13}C NMR spectrum for 1a''.

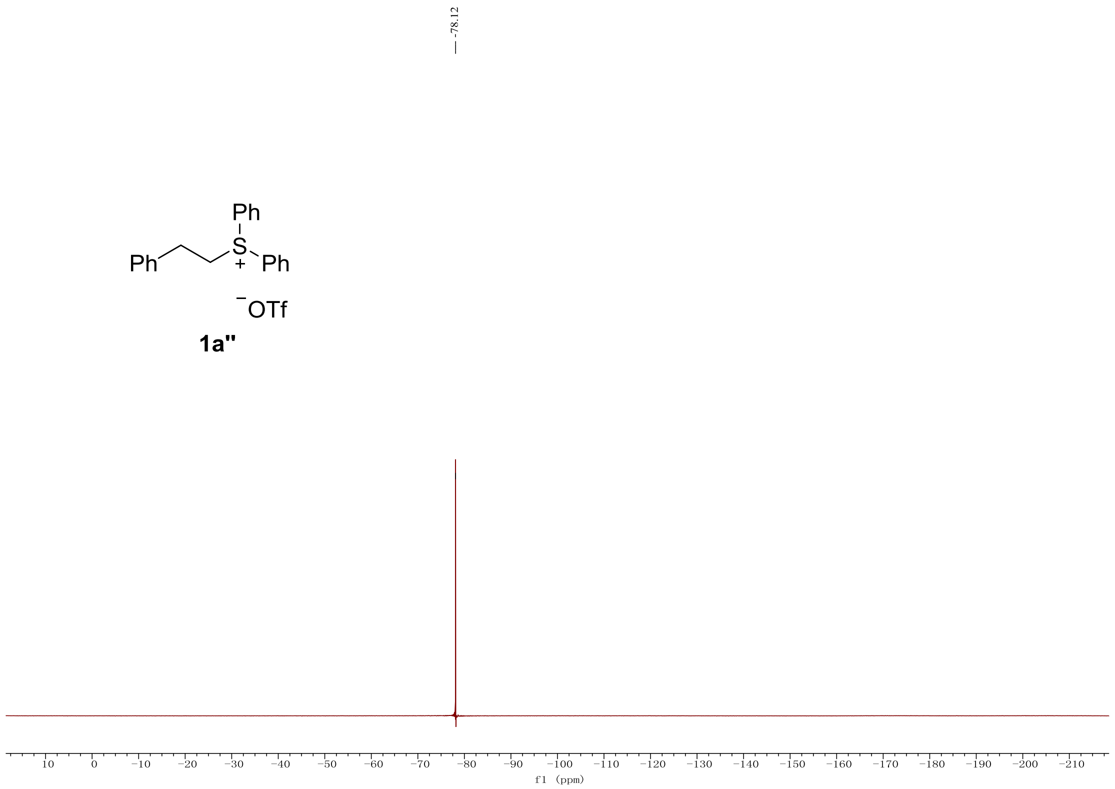

Supplementary Figure 18. ^{19}**F NMR spectrum for 1a''.**

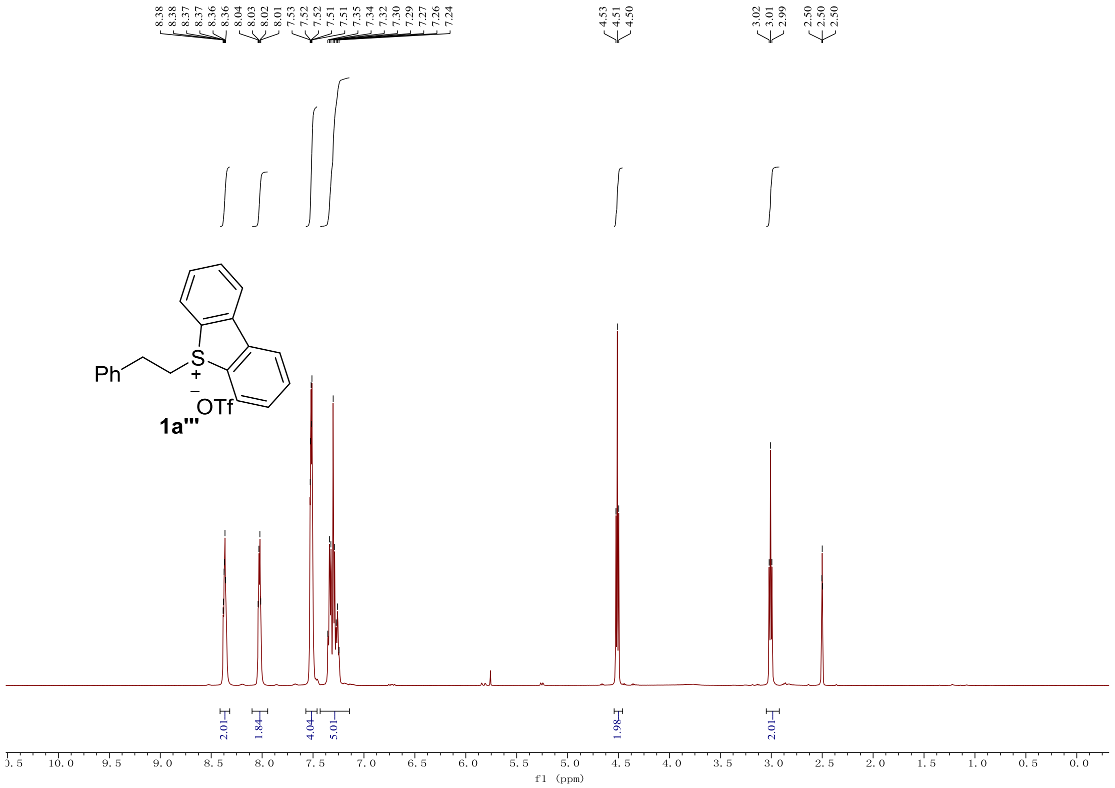

Supplementary Figure 19. ^{1}**H NMR spectrum for 1a'''.**

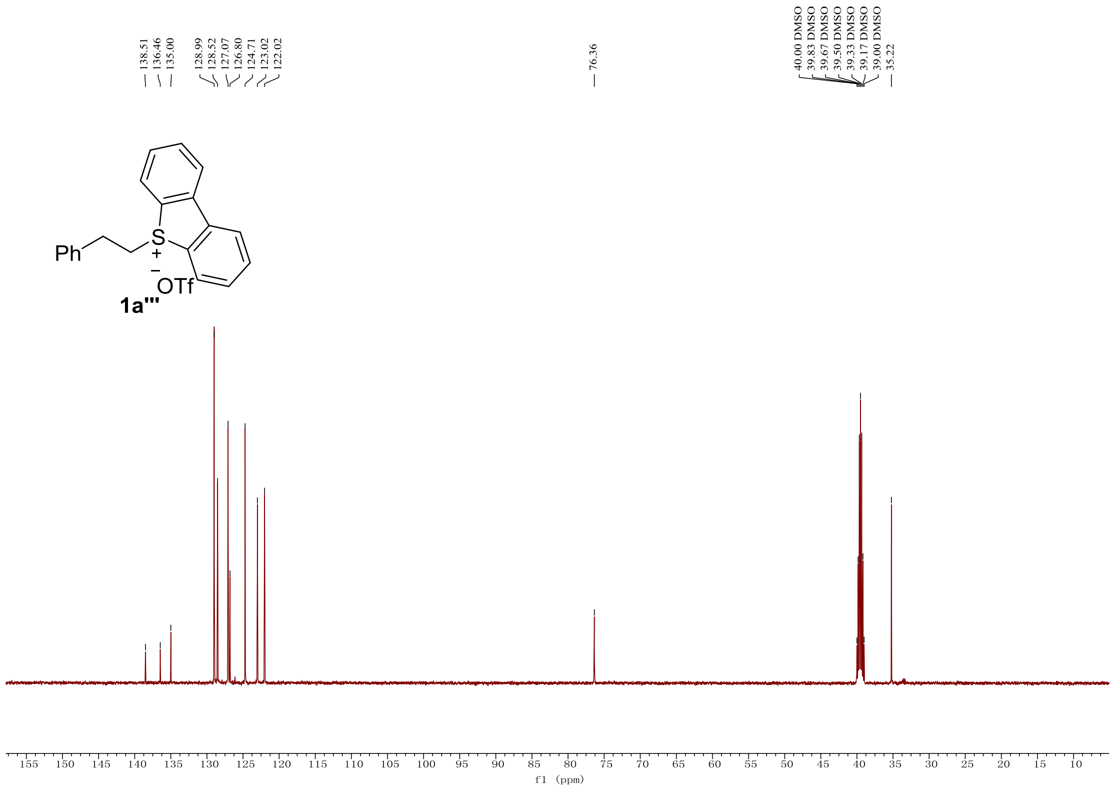

Supplementary Figures 20. ^{13}C NMR spectrum for 1a'''.

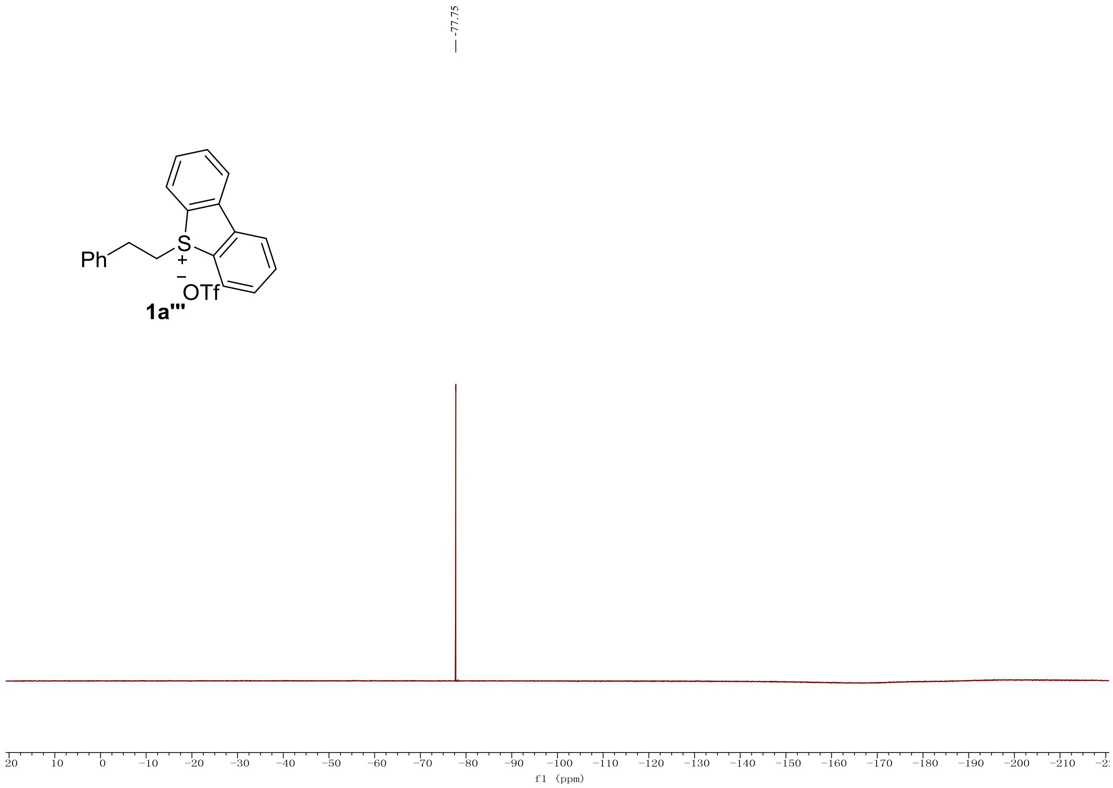

Supplementary Figure 21. ^{19}F NMR spectrum for 1a'''.

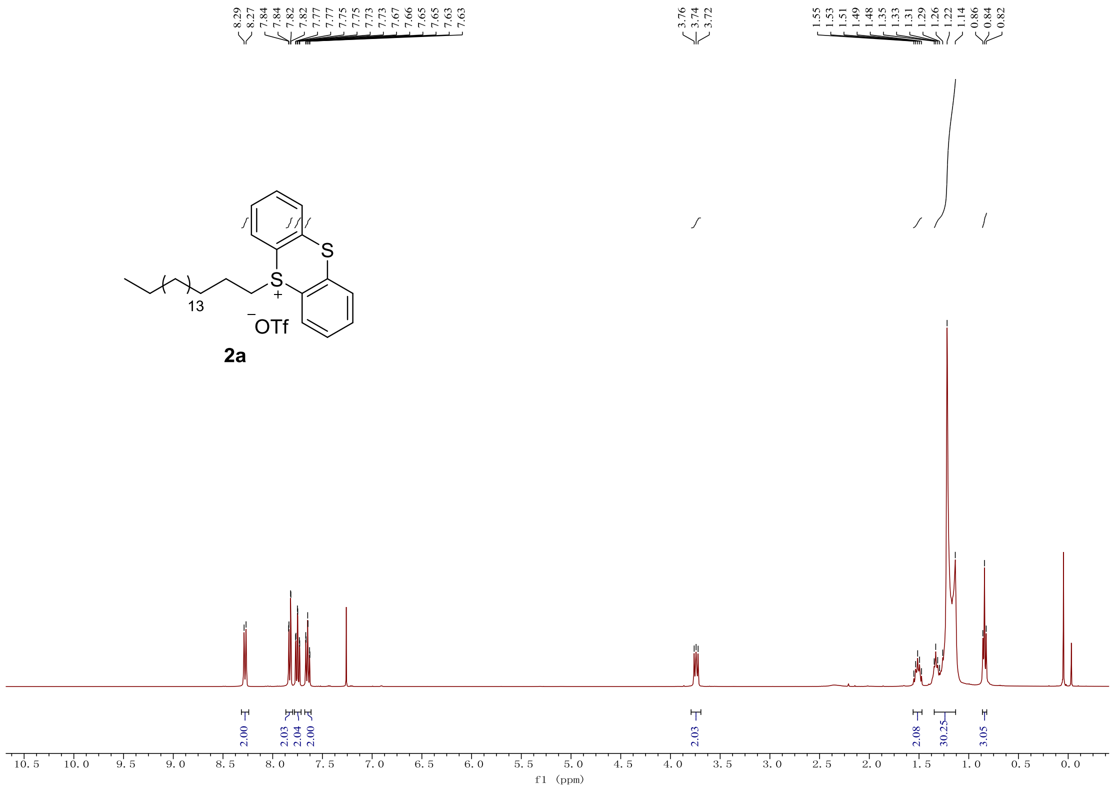

Supplementary Figure 22. ^{1}H NMR spectrum for 2a.

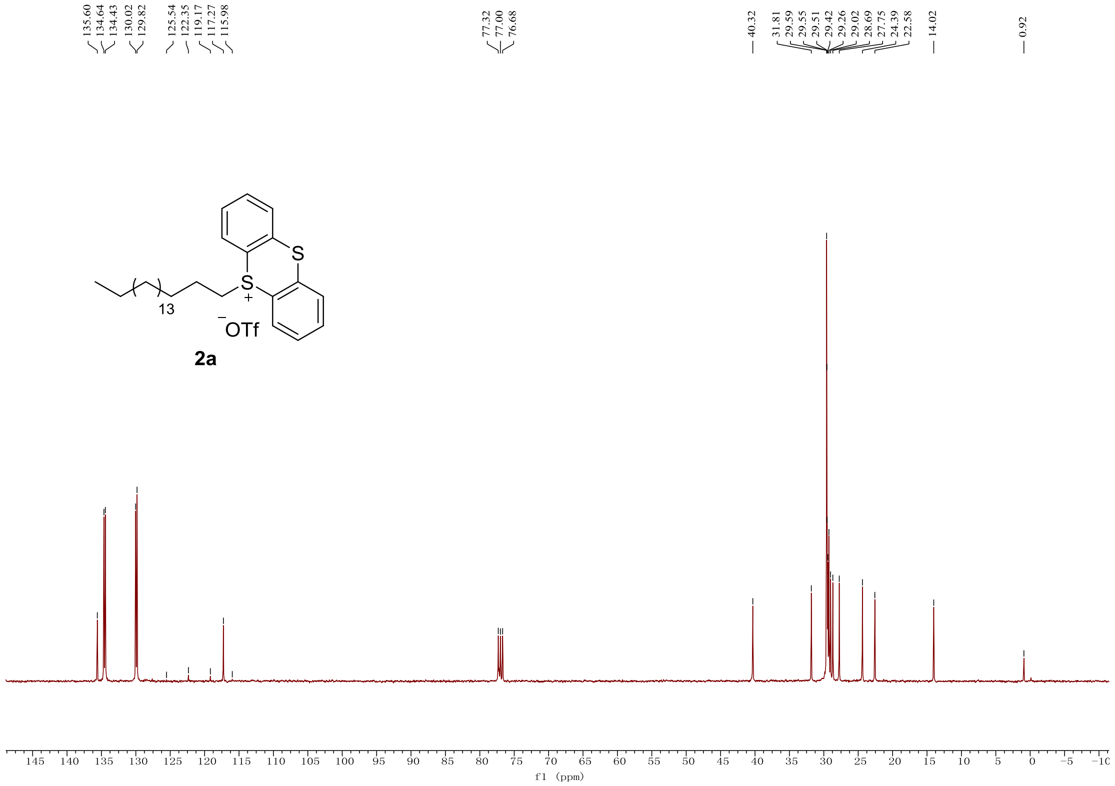

Supplementary Figures 23. ^{13}C NMR spectrum for 2a.

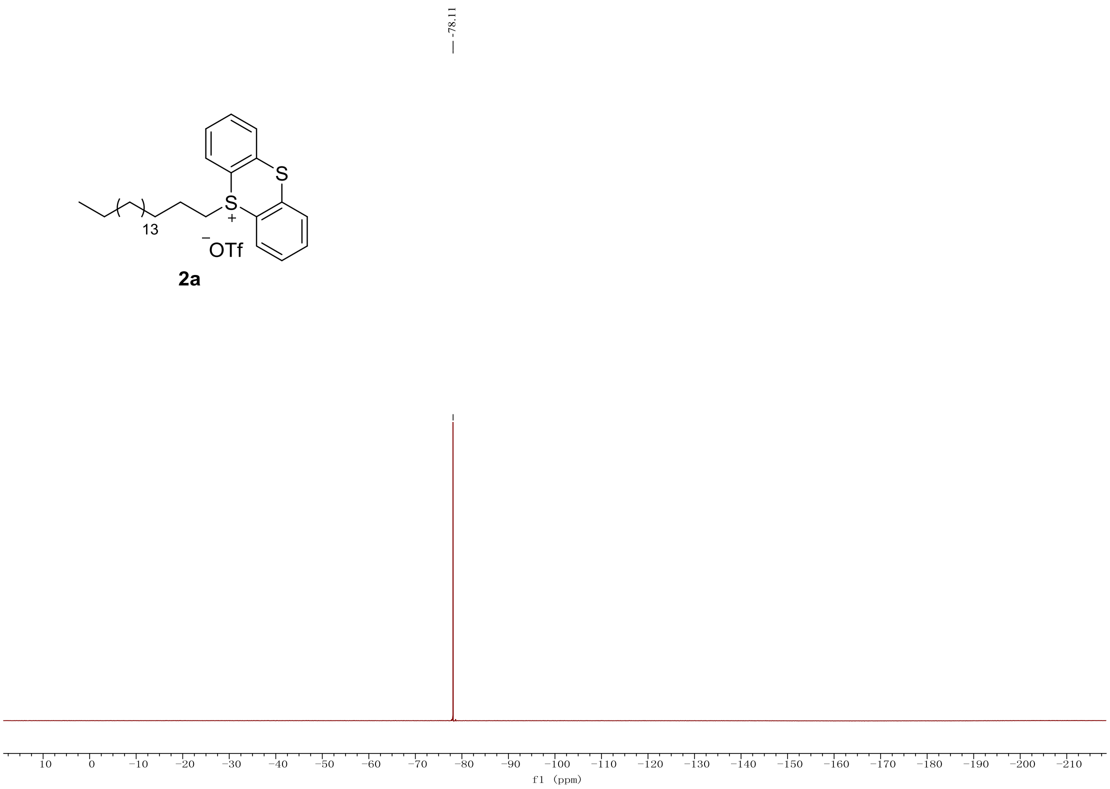

Supplementary Figure 24. ^{19}F NMR spectrum for 2a.

^{1}H NMR (400 MHz, CD_3CN)

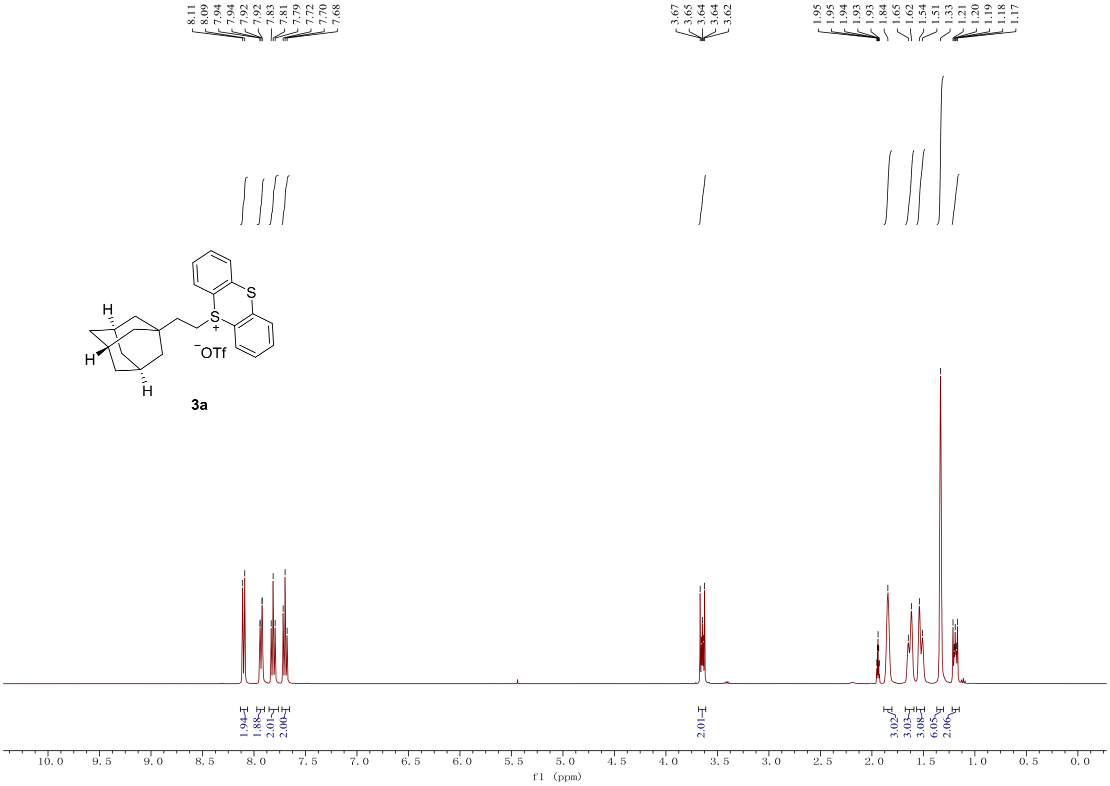

Supplementary Figure 25. ^{1}H NMR spectrum for 3a.

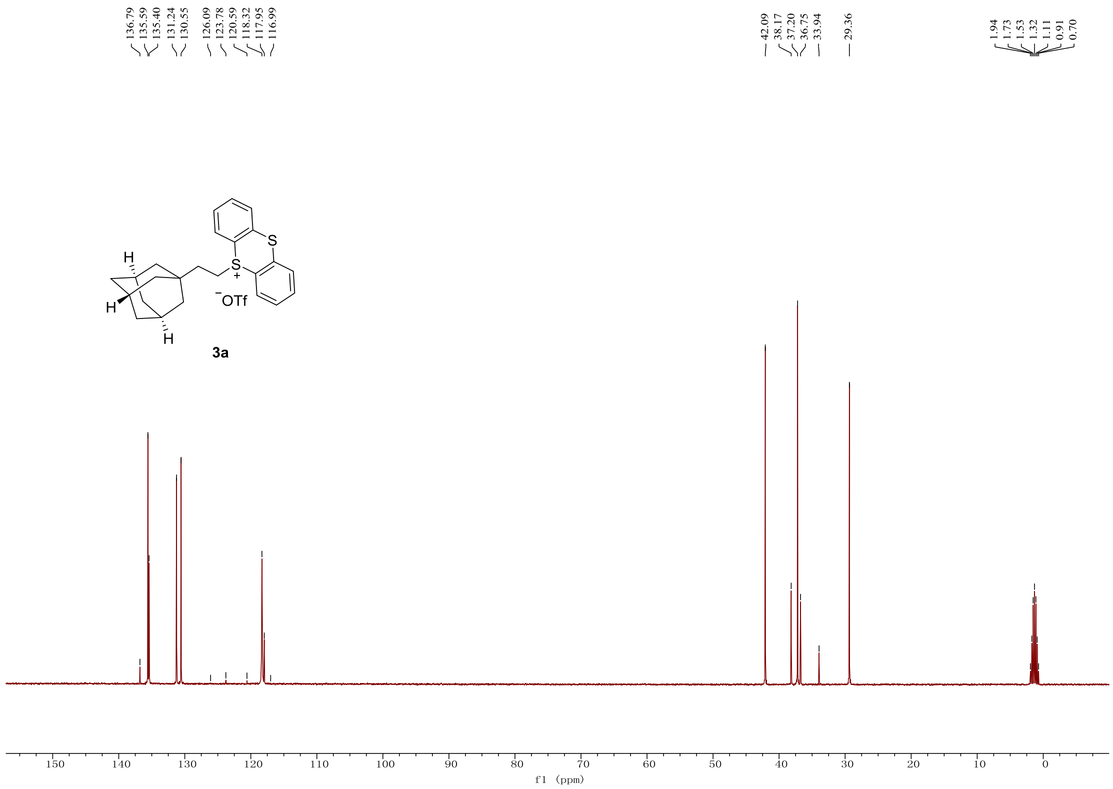

Supplementary Figures 26. ^{13}C NMR spectrum for 3a.

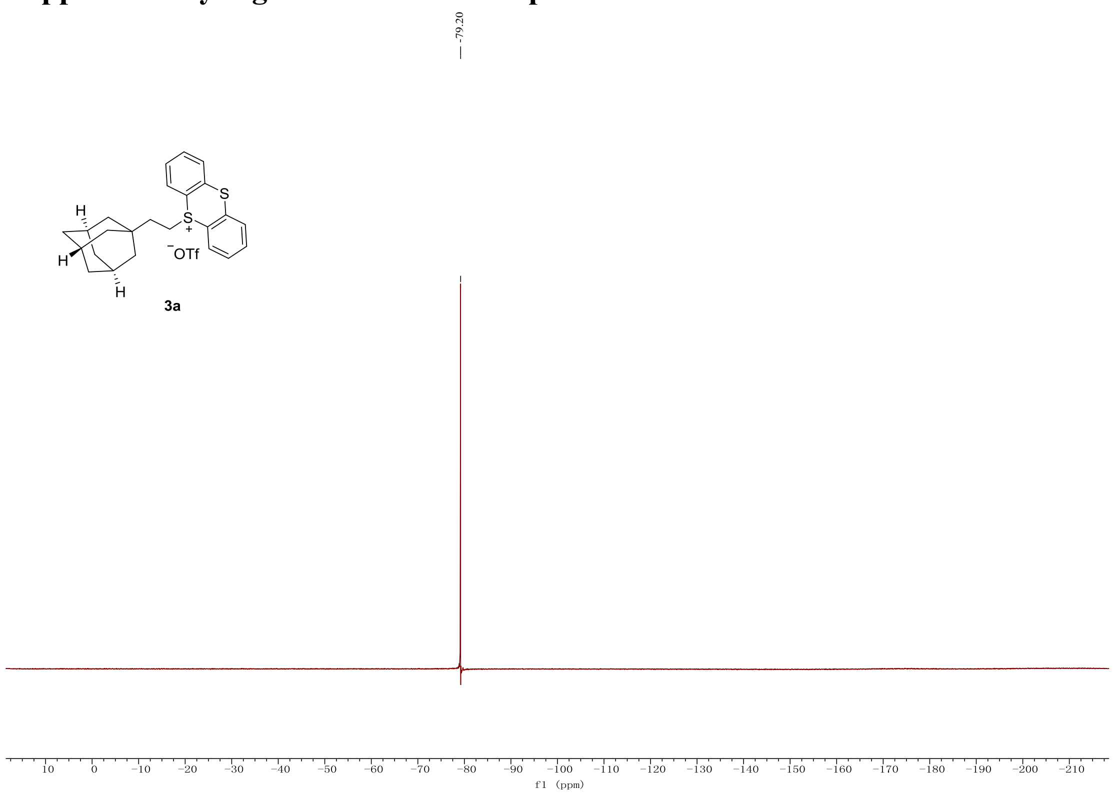

Supplementary Figure 27. ^{19}F NMR spectrum for 3a.

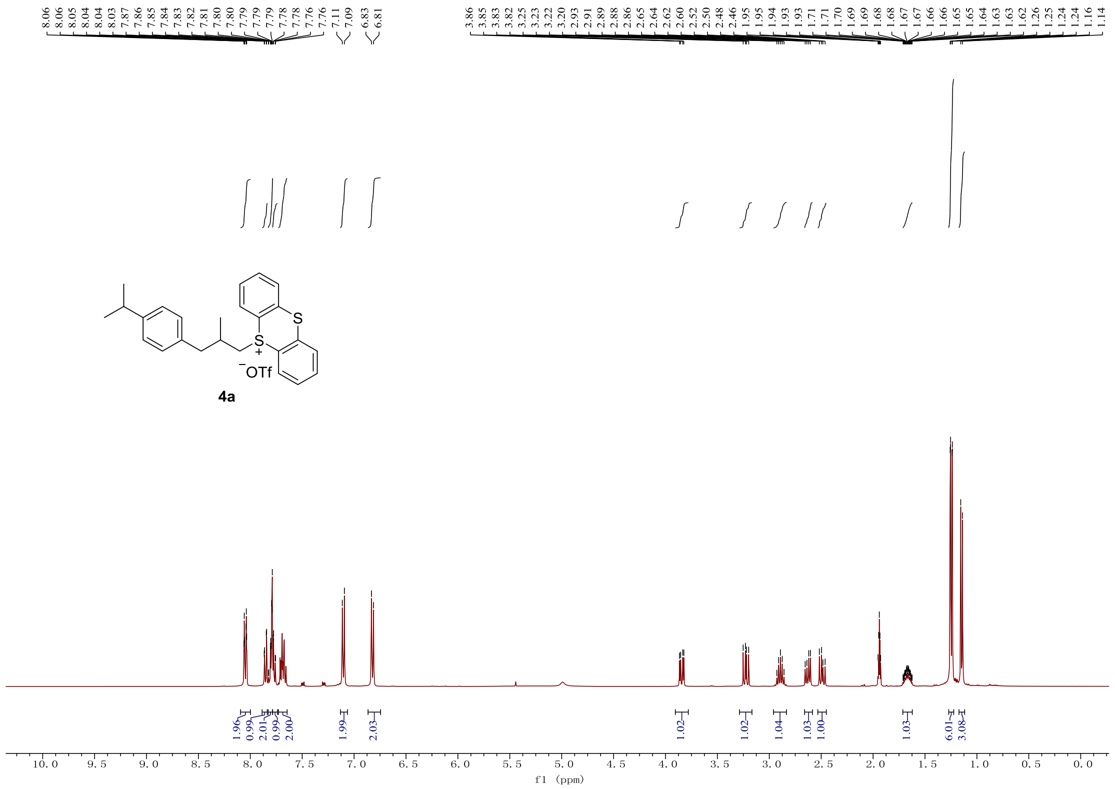

Supplementary Figure 28. ^{1}H NMR spectrum for 4a.

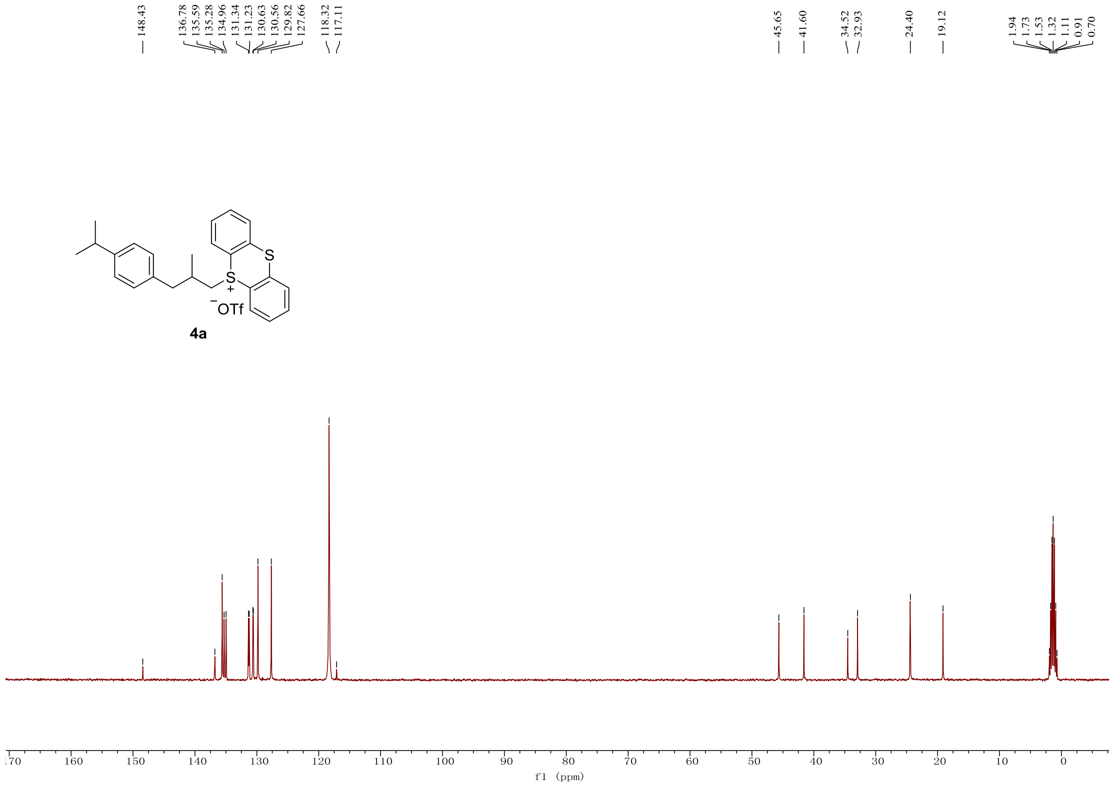

Supplementary Figures 29. ^{13}C NMR spectrum for 4a.

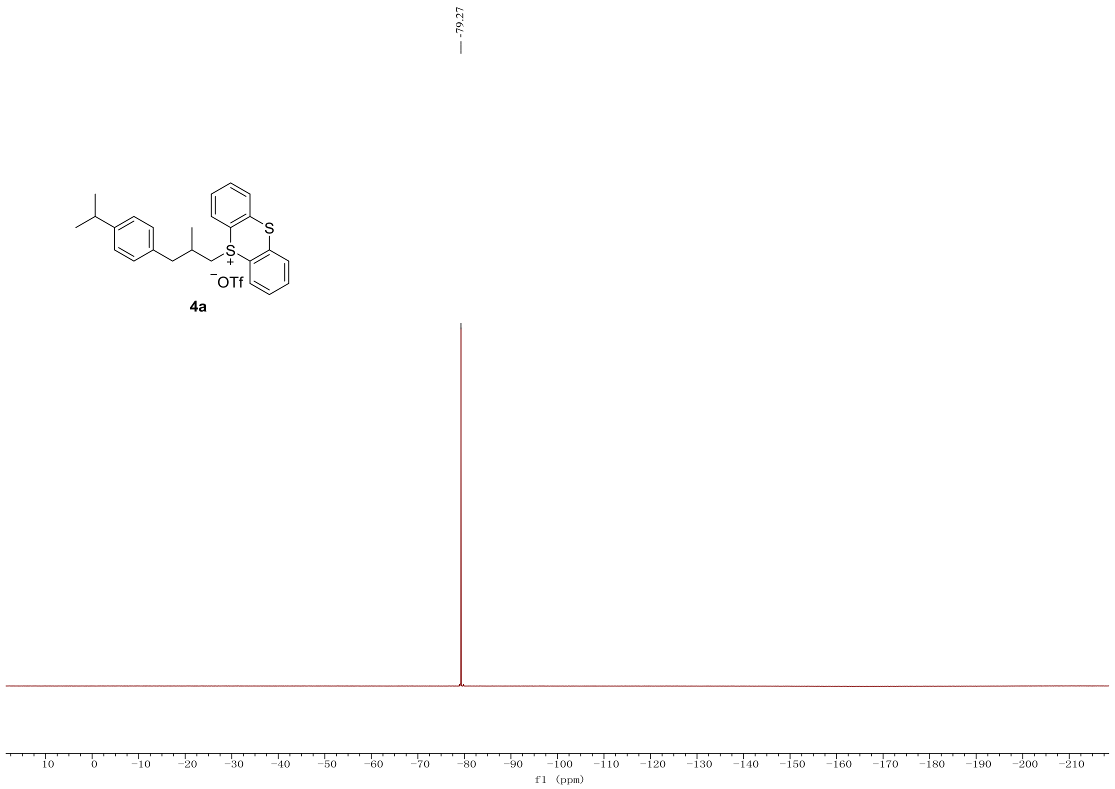

Supplementary Figure 30. ^{19}F NMR spectrum for 4a.

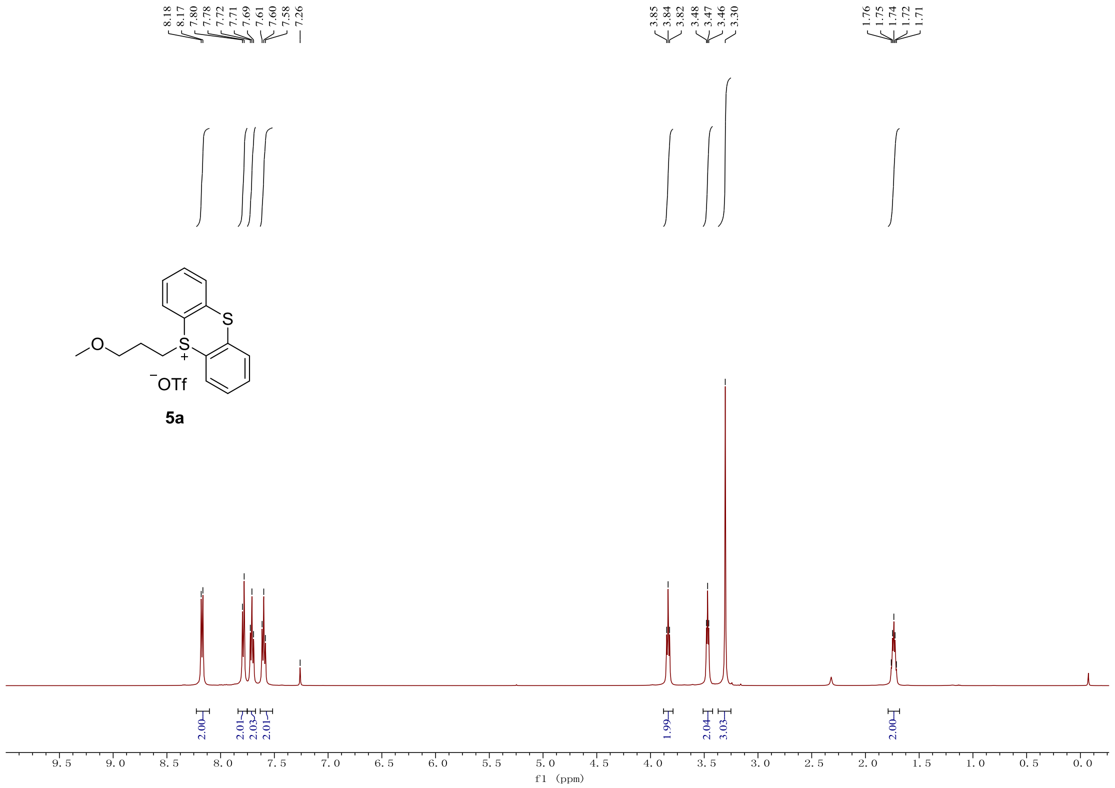

Supplementary Figure 31. ^{1}H NMR spectrum for 5a.

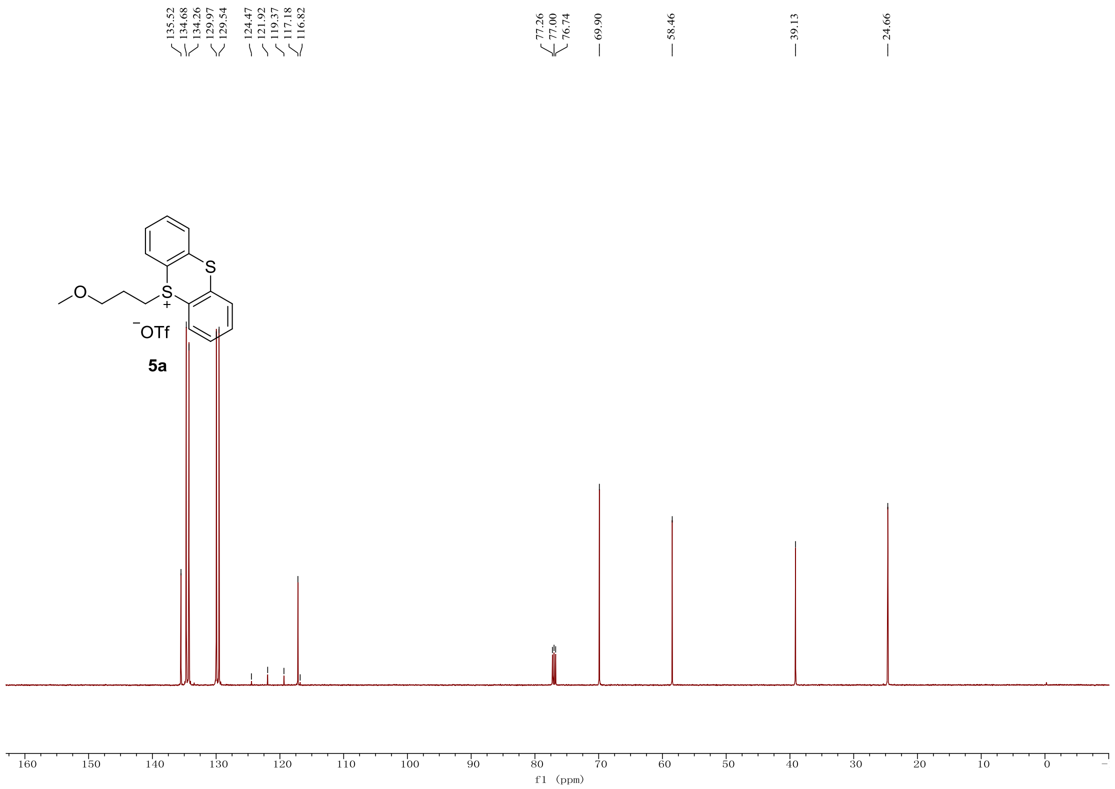

Supplementary Figures 32. ^{13}C NMR spectrum for 5a.

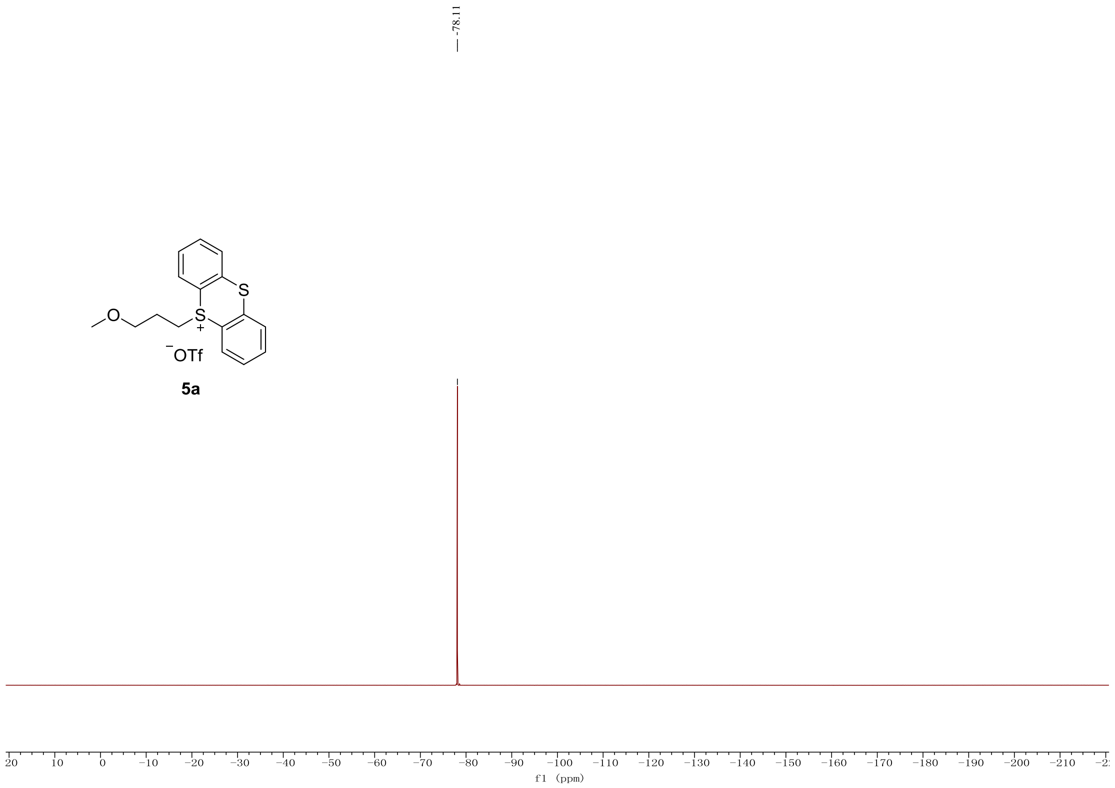

Supplementary Figure 33. ^{19}F NMR spectrum for 5a.

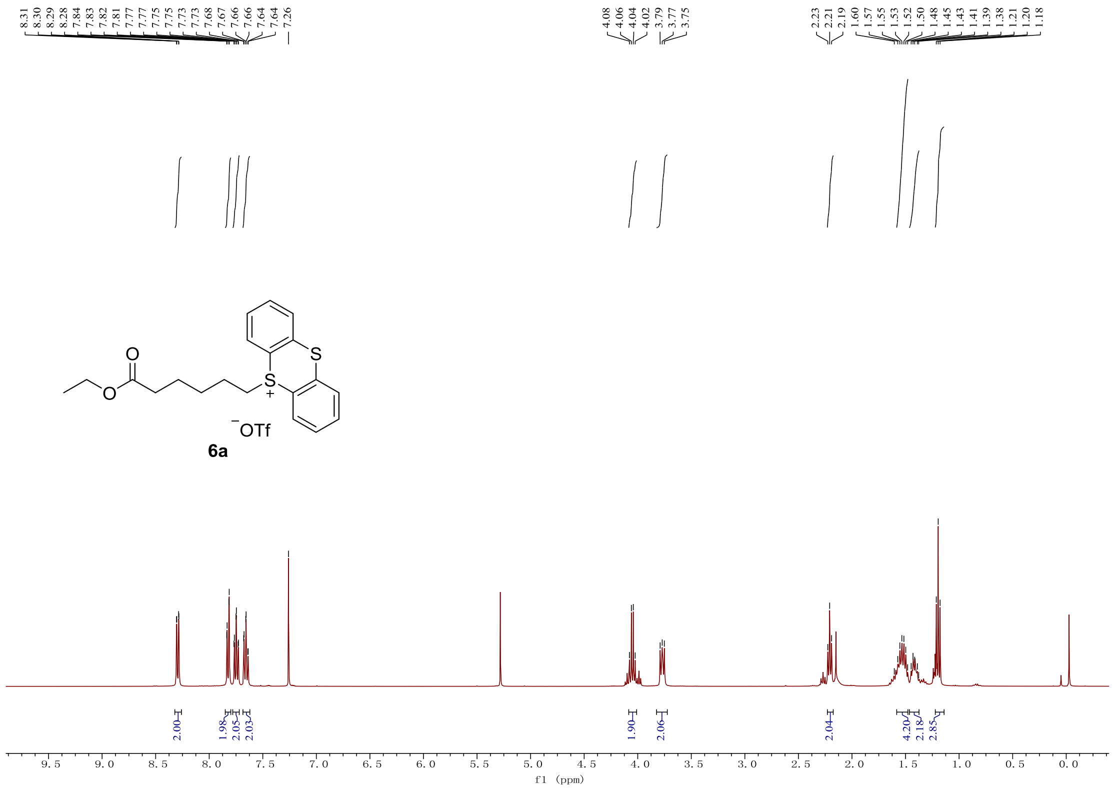

Supplementary Figure 34. ^{1}H NMR spectrum for 6a.

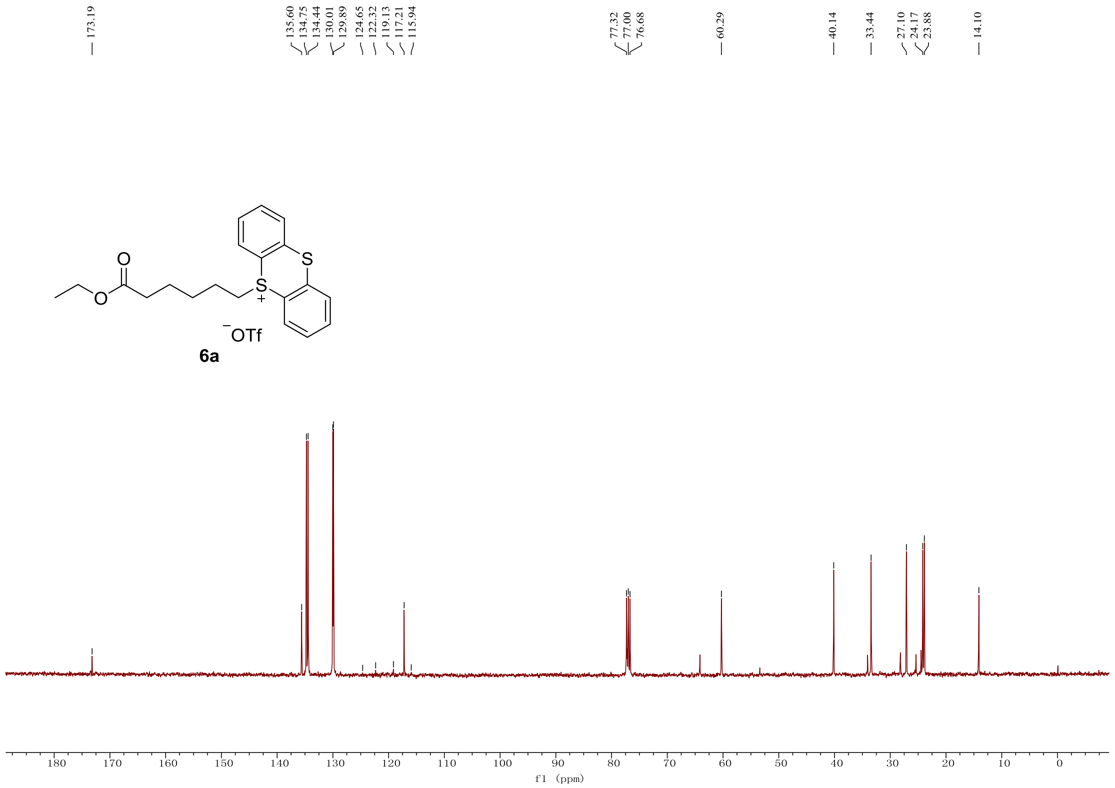

Supplementary Figures 35. ^{13}C NMR spectrum for 6a.

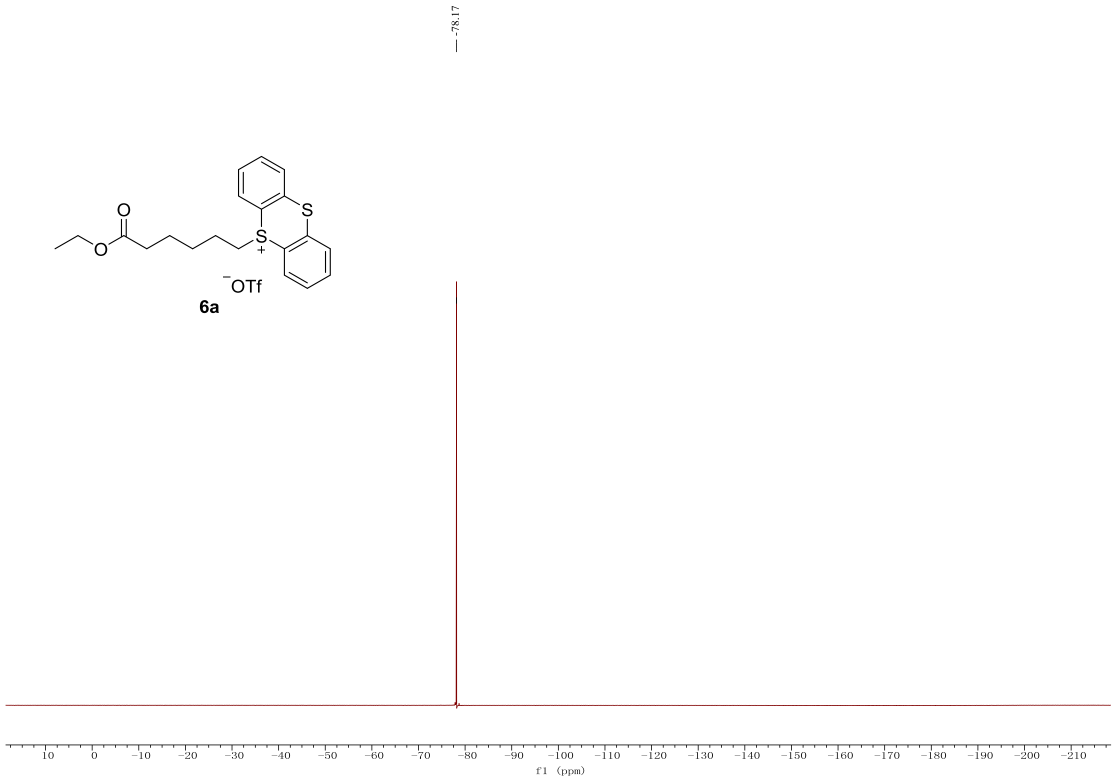

Supplementary Figure 36. ^{19}F NMR spectrum for 6a.

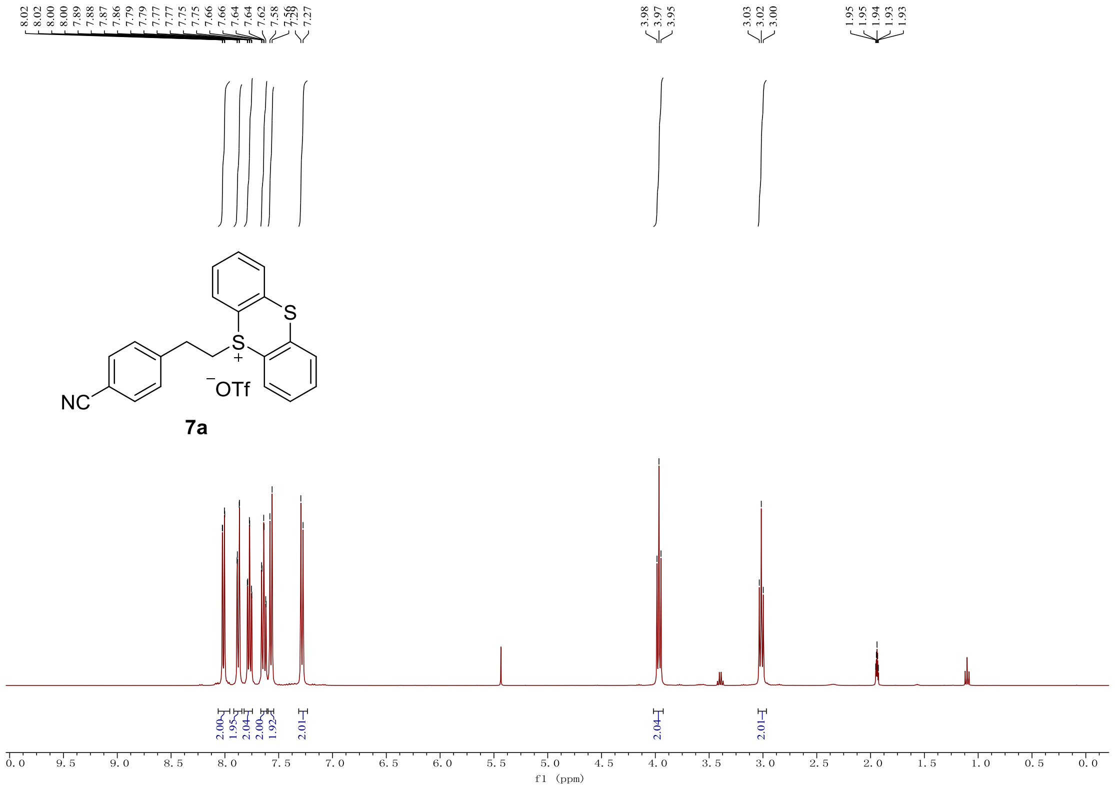

Supplementary Figure 37. ^{1}H NMR spectrum for 7a.

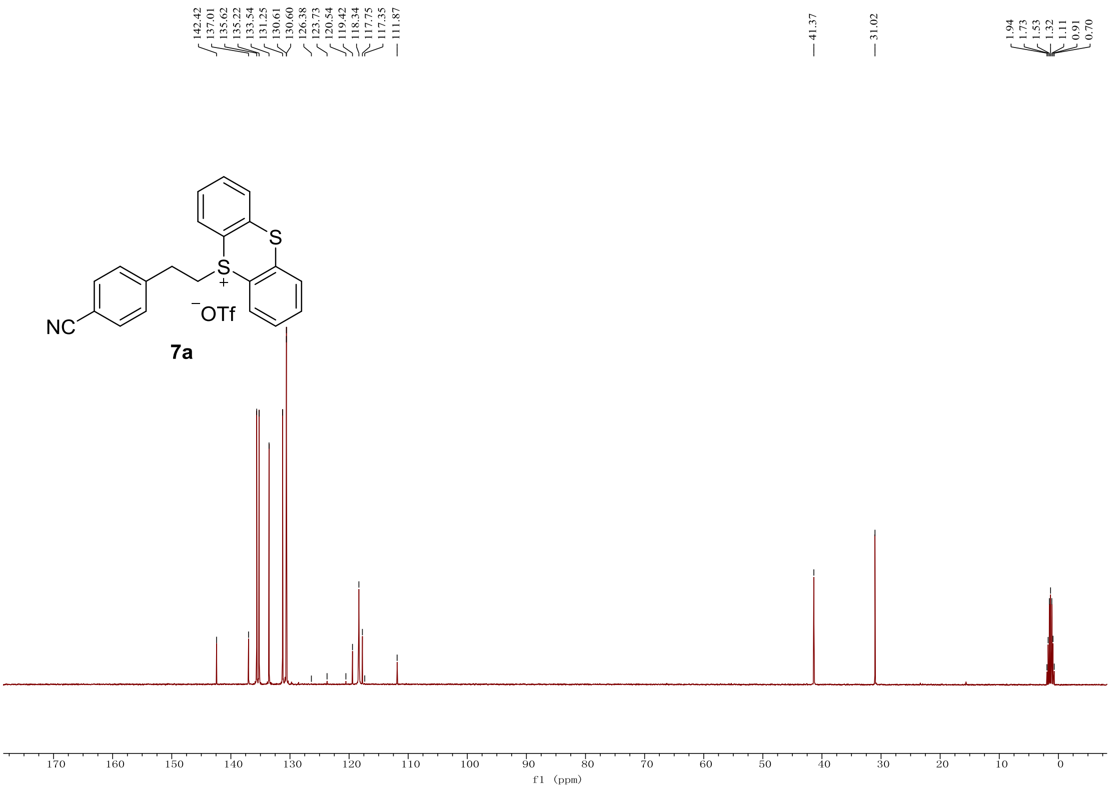

Supplementary Figures 38. ^{13}C NMR spectrum for 7a.

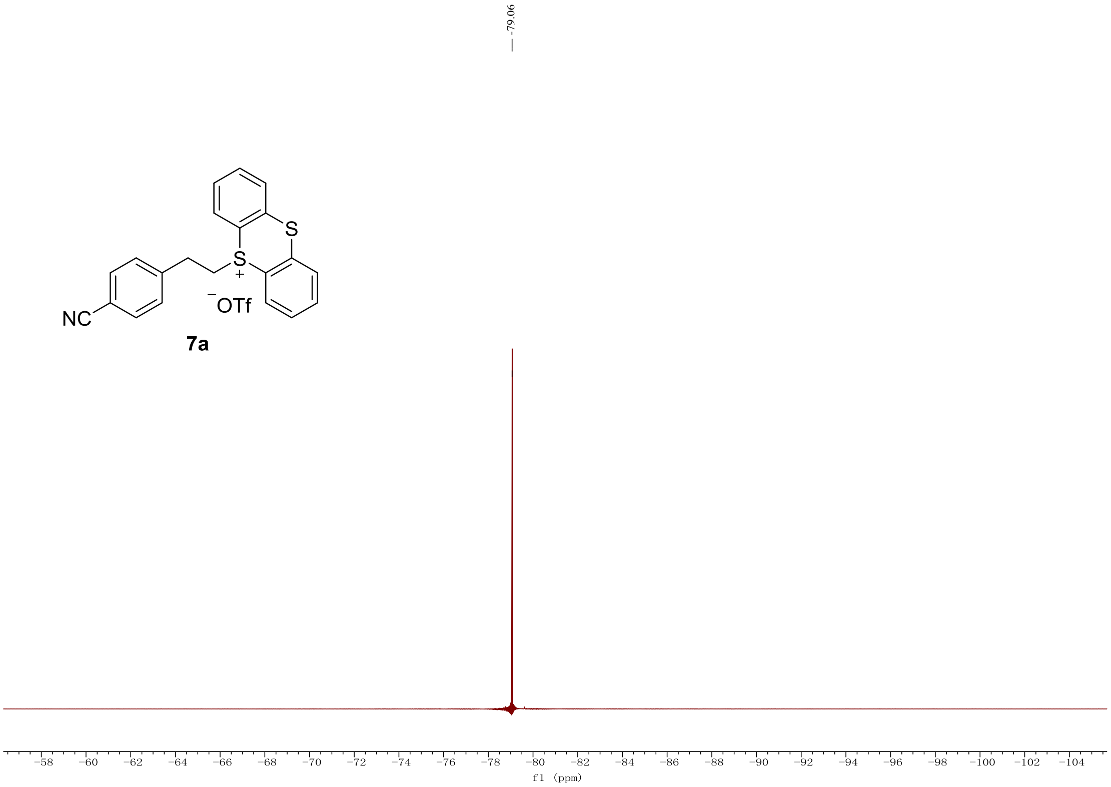

Supplementary Figure 39. ^{19}F NMR spectrum for 7a.

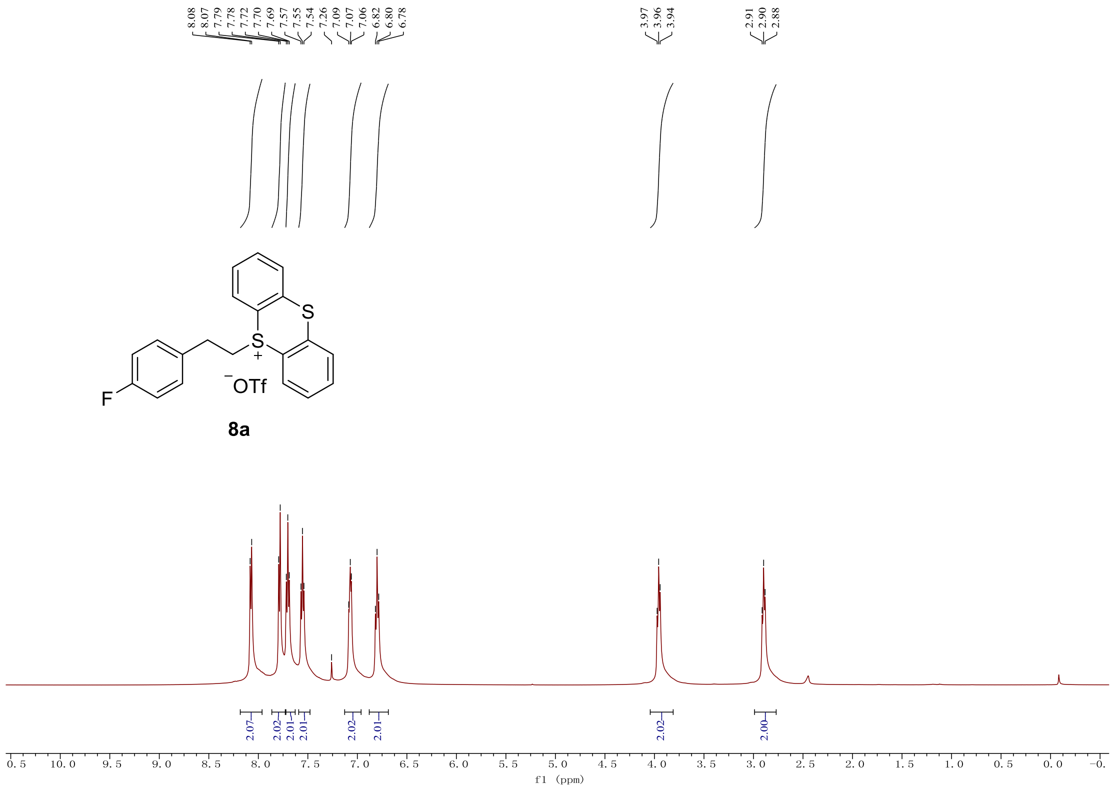

Supplementary Figure 40. ^{1}H NMR spectrum for 8a.

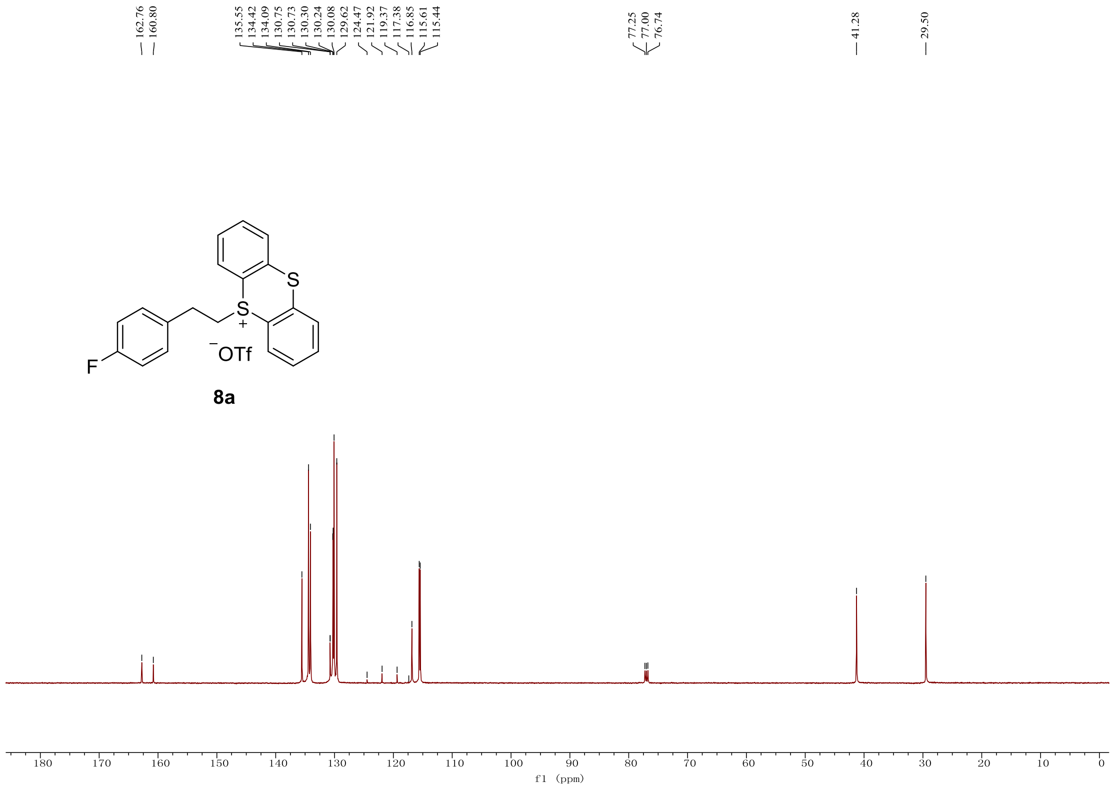

Supplementary Figures 41. ^{13}C NMR spectrum for 8a.

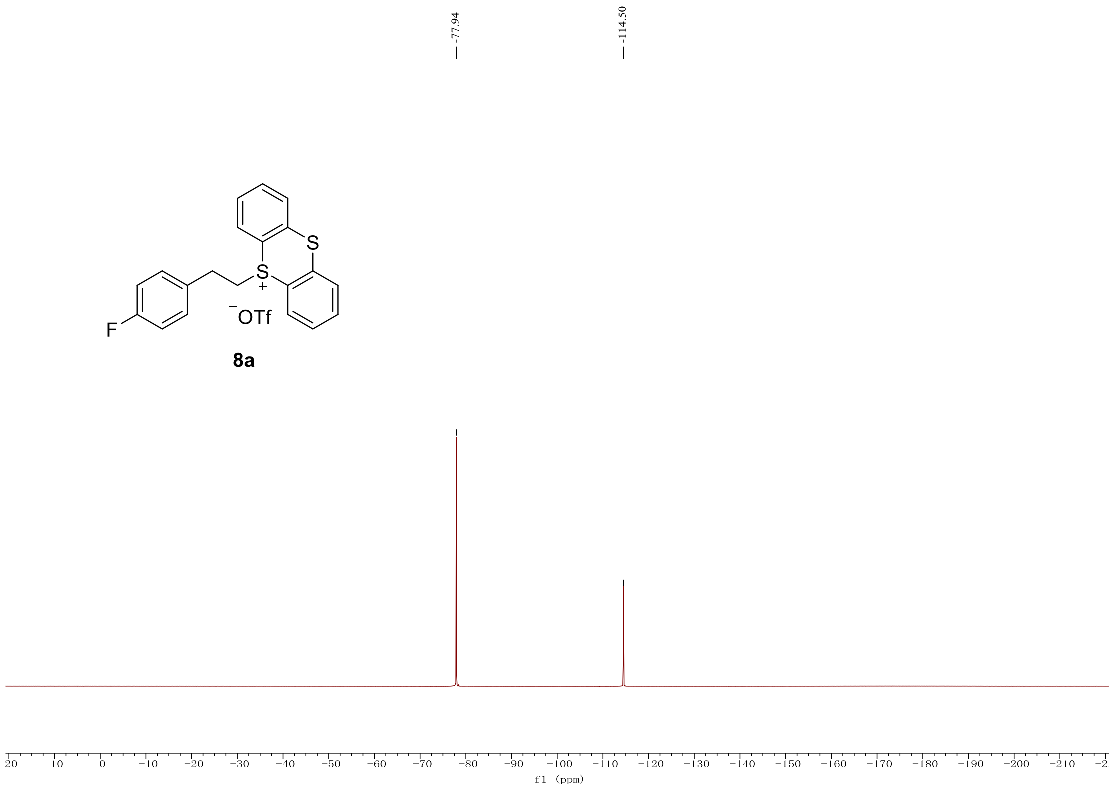

Supplementary Figure 42. ^{19}**F NMR spectrum for 8a.**

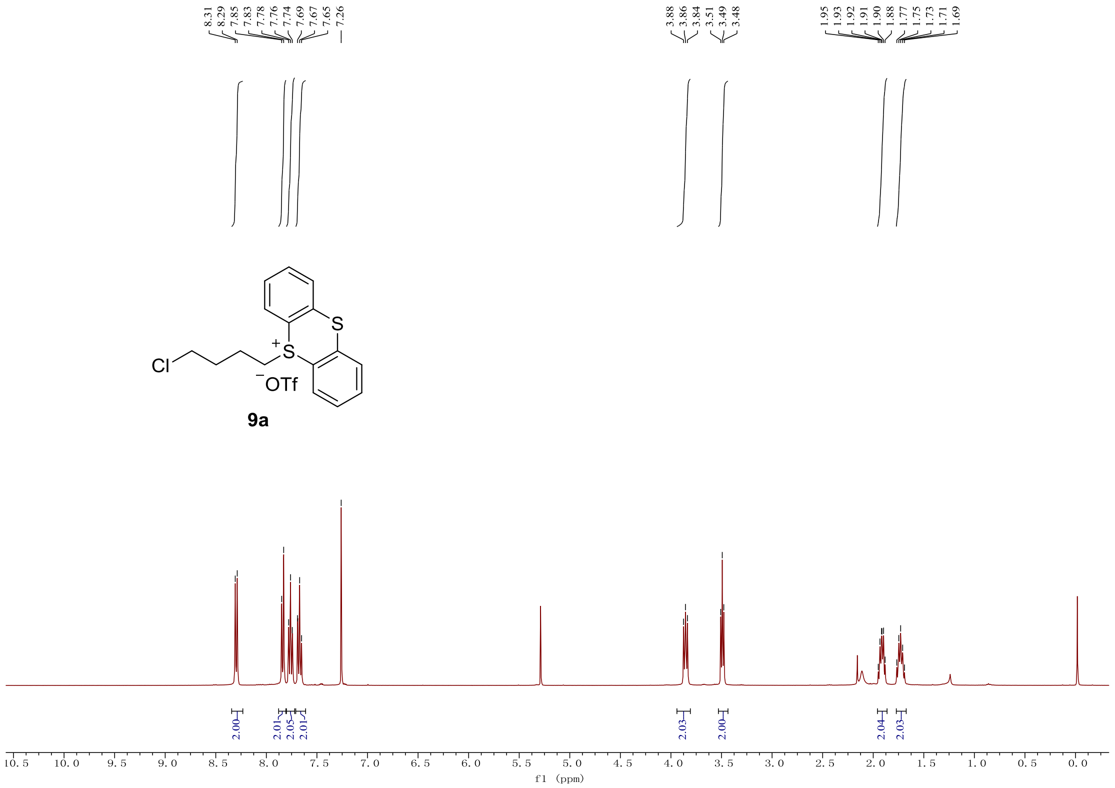

Supplementary Figure 43. ^{1}**H NMR spectrum for 9a.**

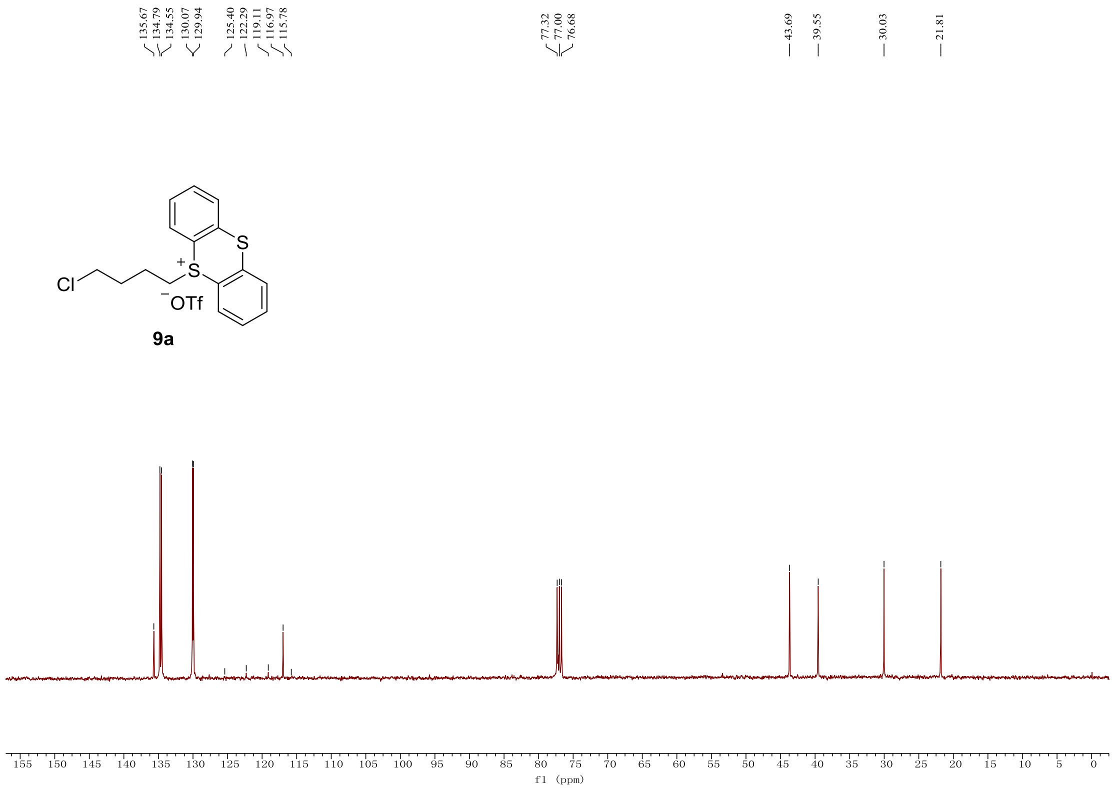

Supplementary Figures 44. ^{13}C NMR spectrum for 9a.

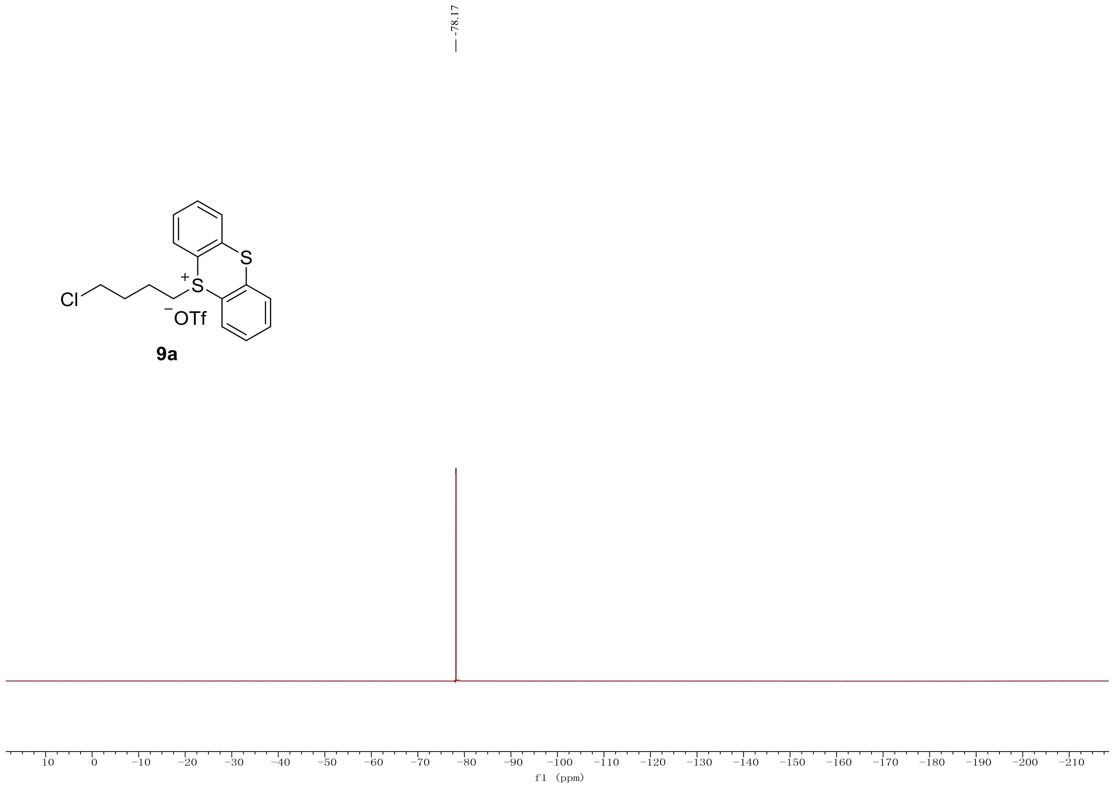

Supplementary Figure 45. ^{19}F NMR spectrum for 9a.

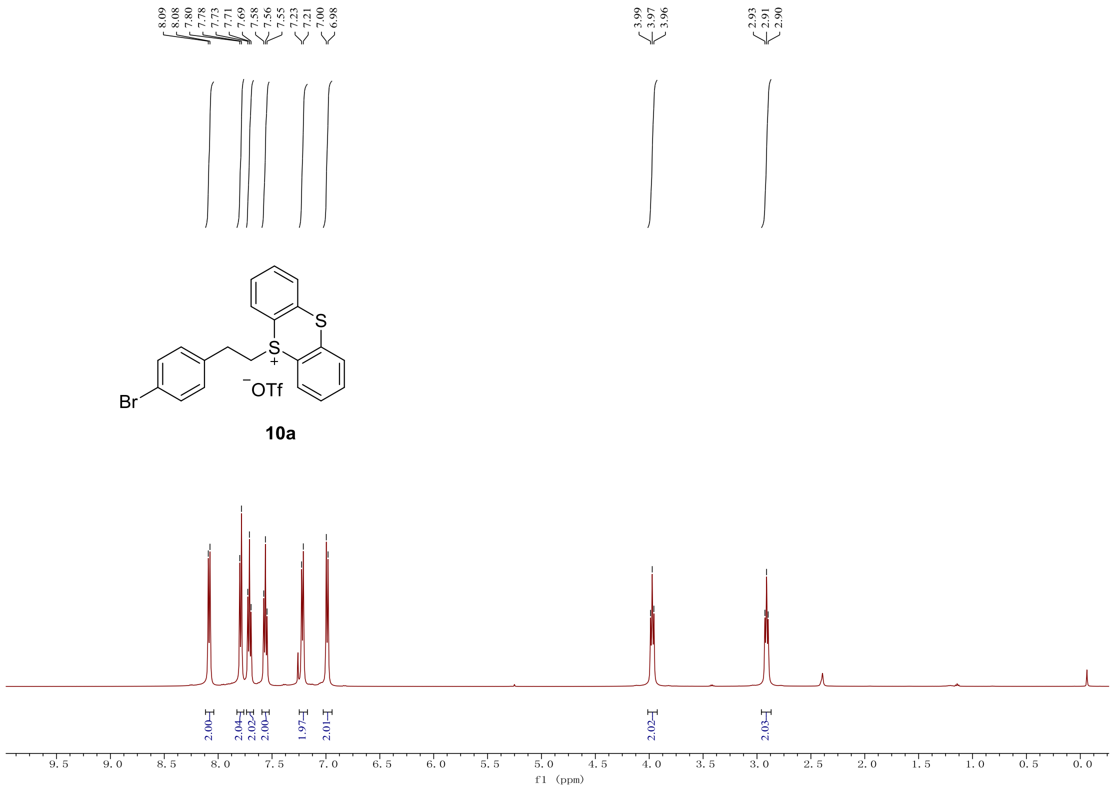

Supplementary Figure 46. ^{1}H NMR spectrum for 10a.

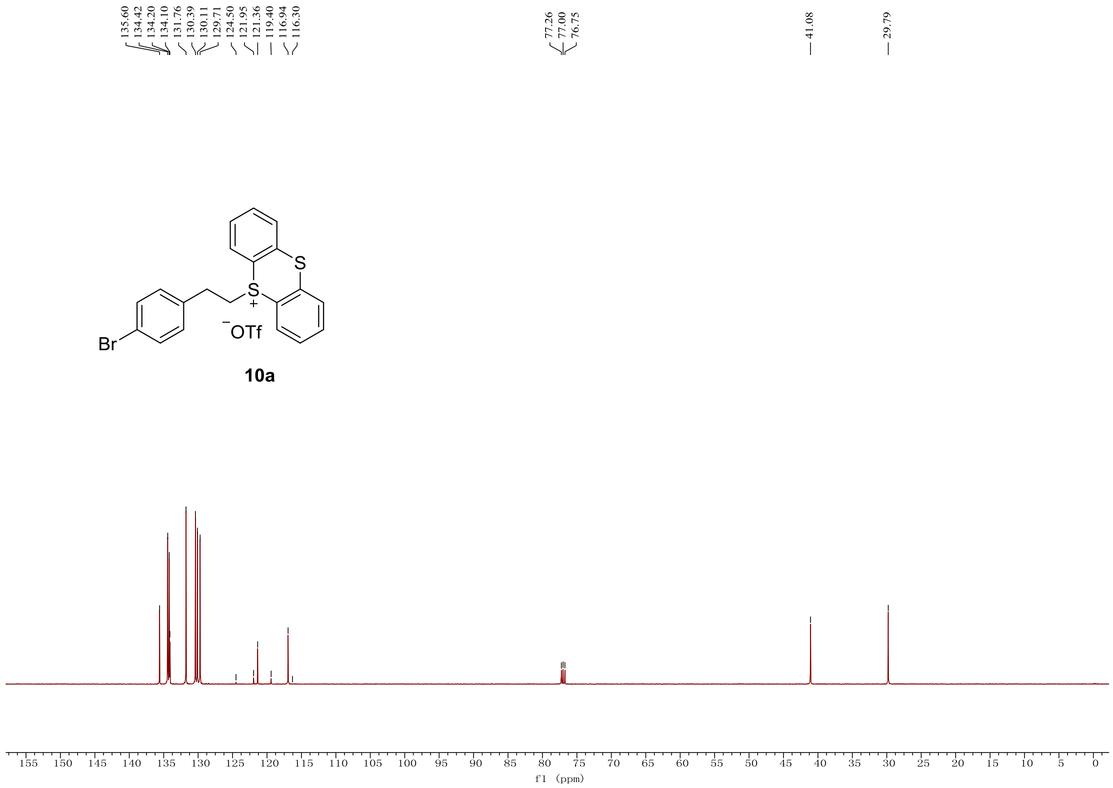

Supplementary Figures 47. ^{13}C NMR spectrum for 10a.

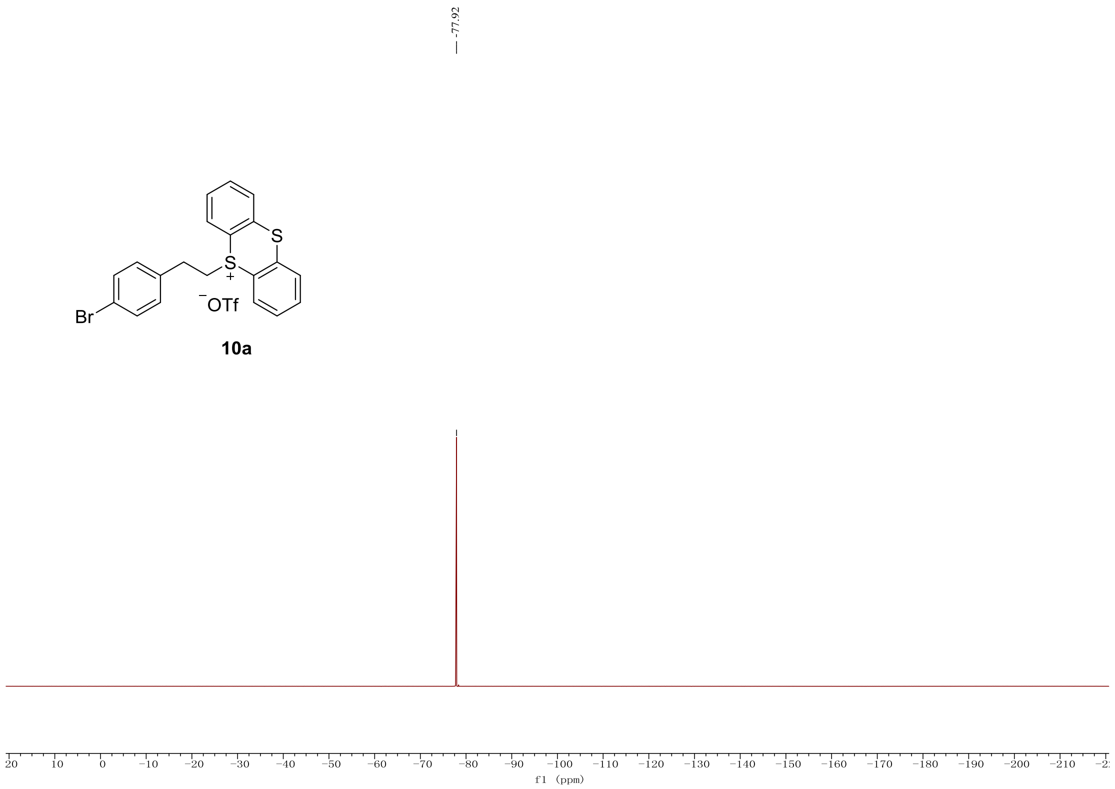

Supplementary Figure 48. ^{19}**F NMR spectrum for 10a.**

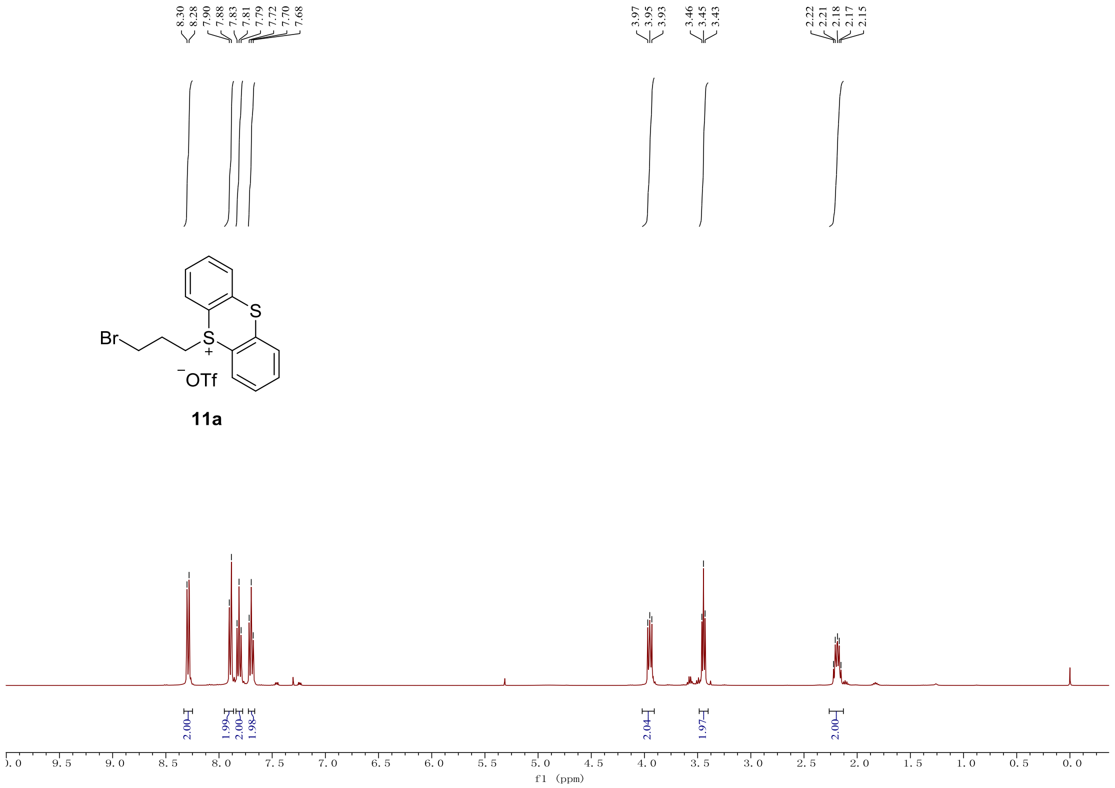

Supplementary Figure 49. ^{1}**H NMR spectrum for 11a.**

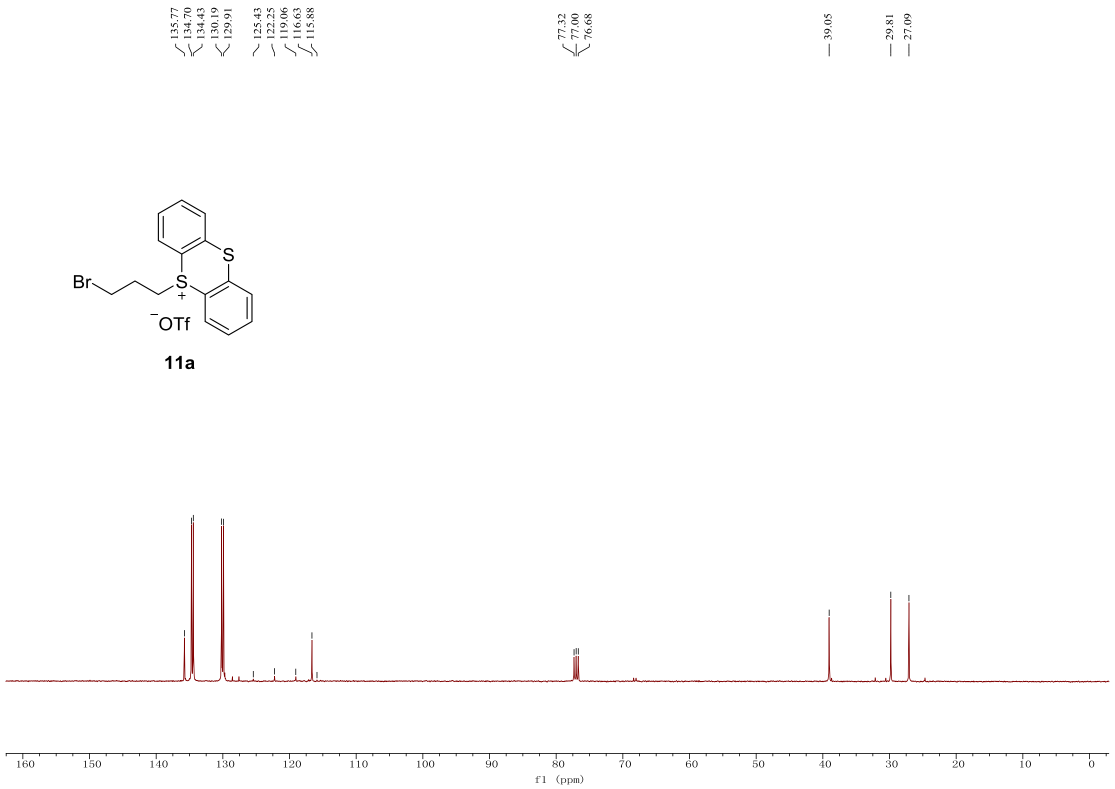

Supplementary Figures 50. ^{13}C NMR spectrum for 11a.

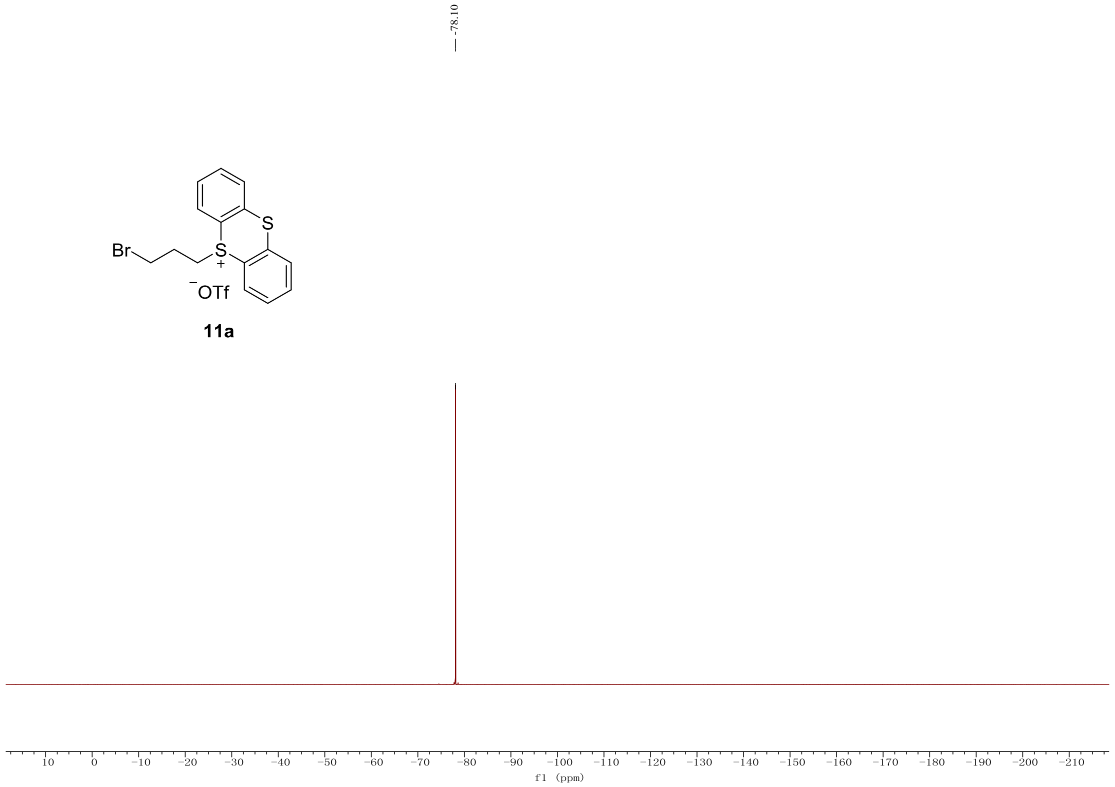

Supplementary Figure 51. ^{19}F NMR spectrum for 11a.

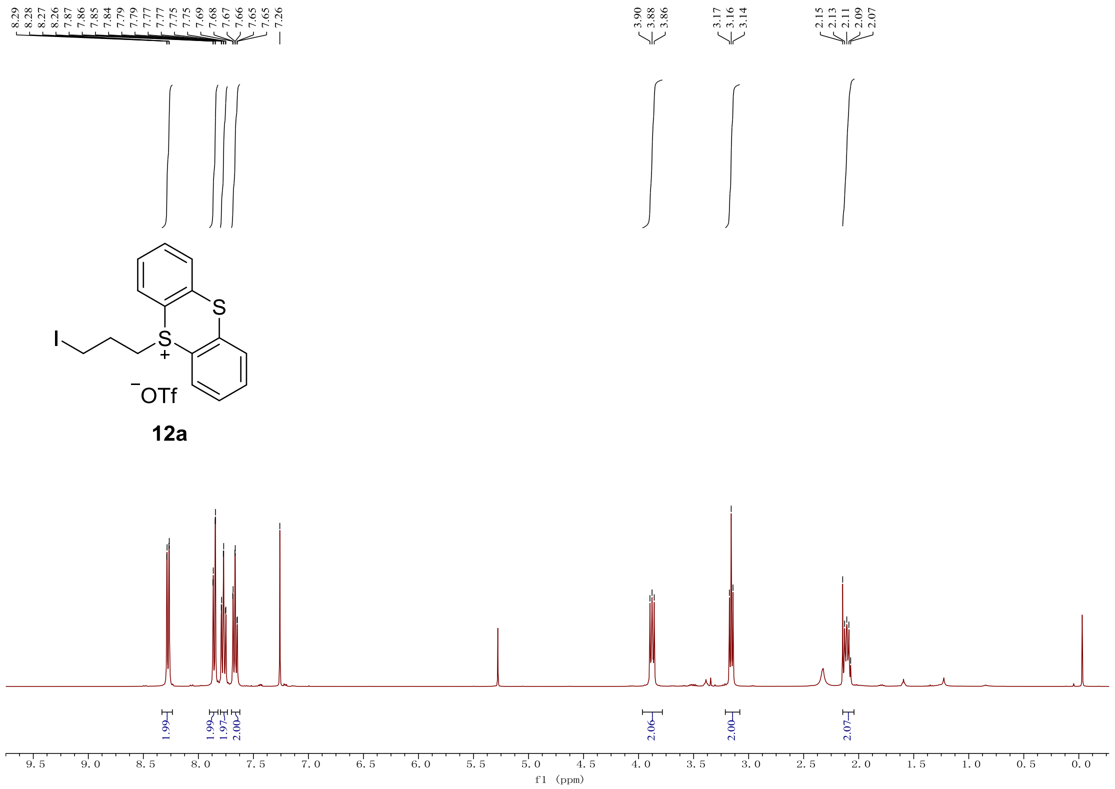

Supplementary Figure 52. ^{1}H NMR spectrum for 12a.

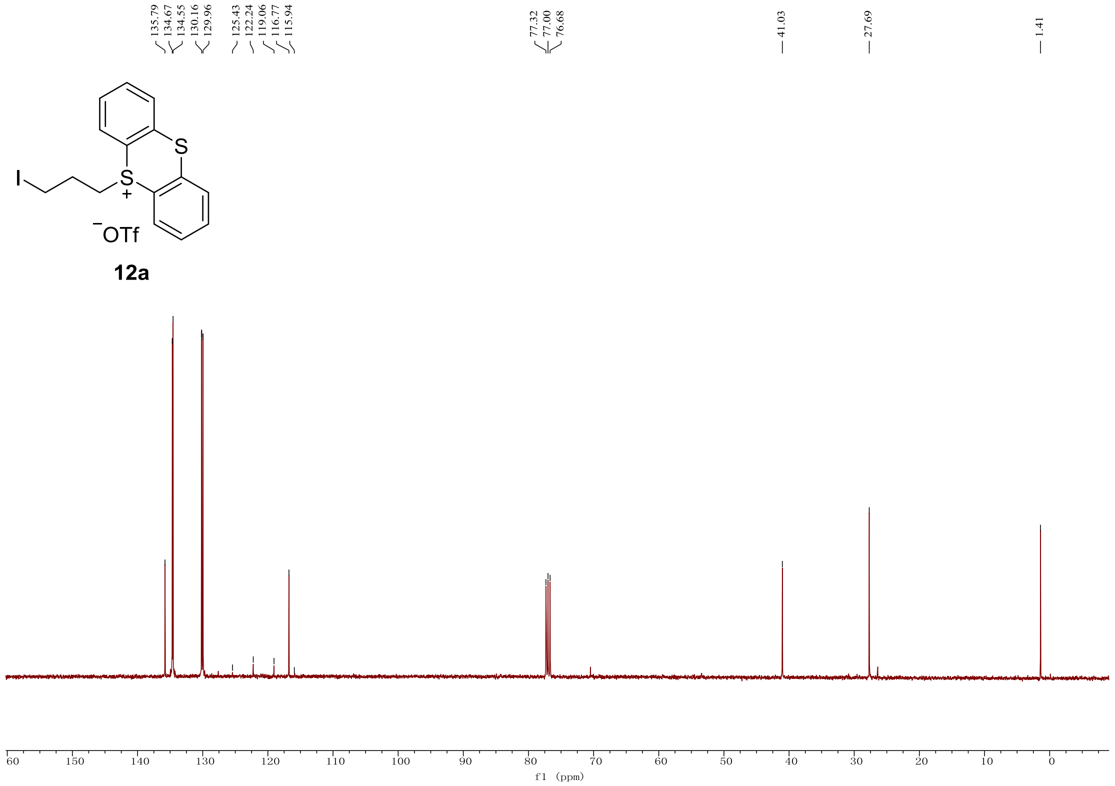

Supplementary Figures 53. ^{13}C NMR spectrum for 12a.

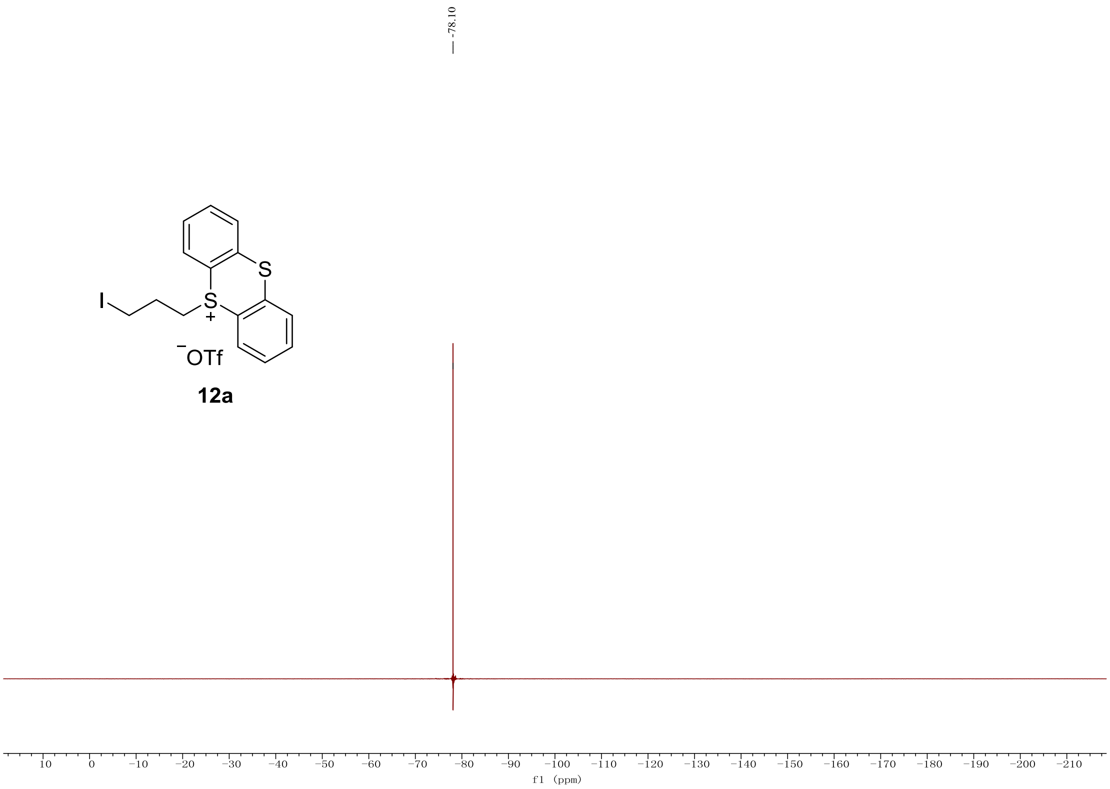

Supplementary Figure 54. ^{19}F NMR spectrum for 12a.

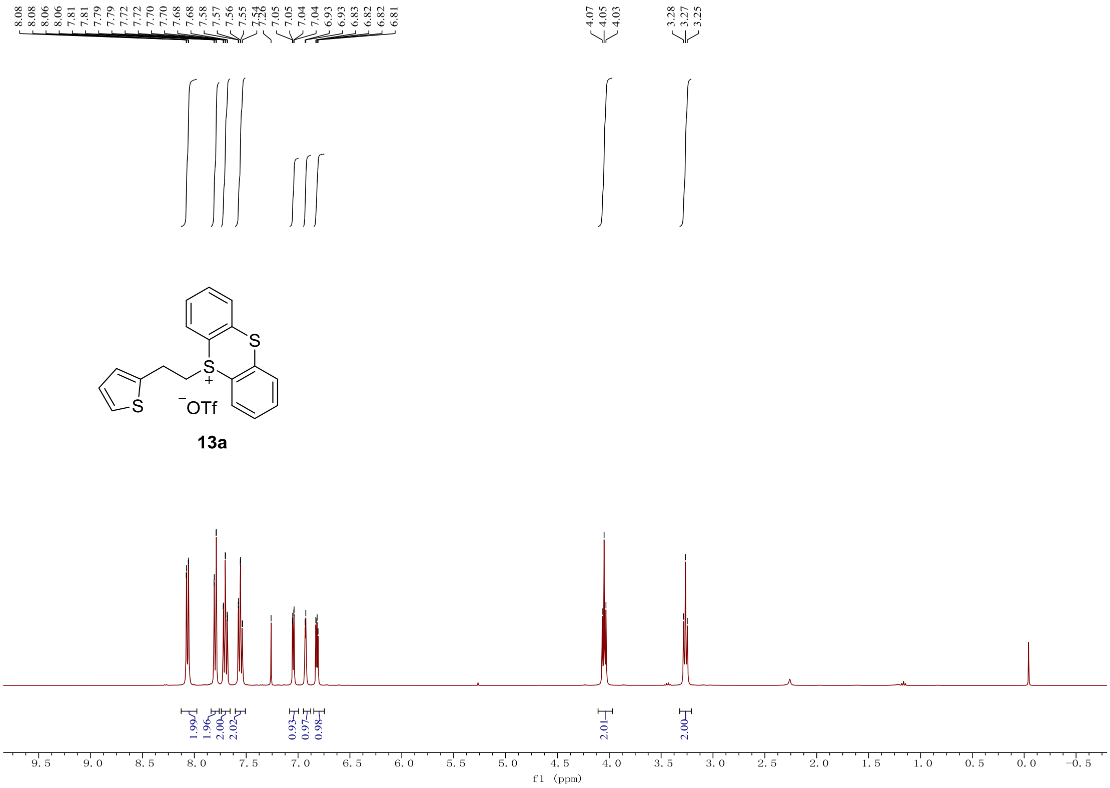

Supplementary Figure 55. ^{1}H NMR spectrum for 13a.

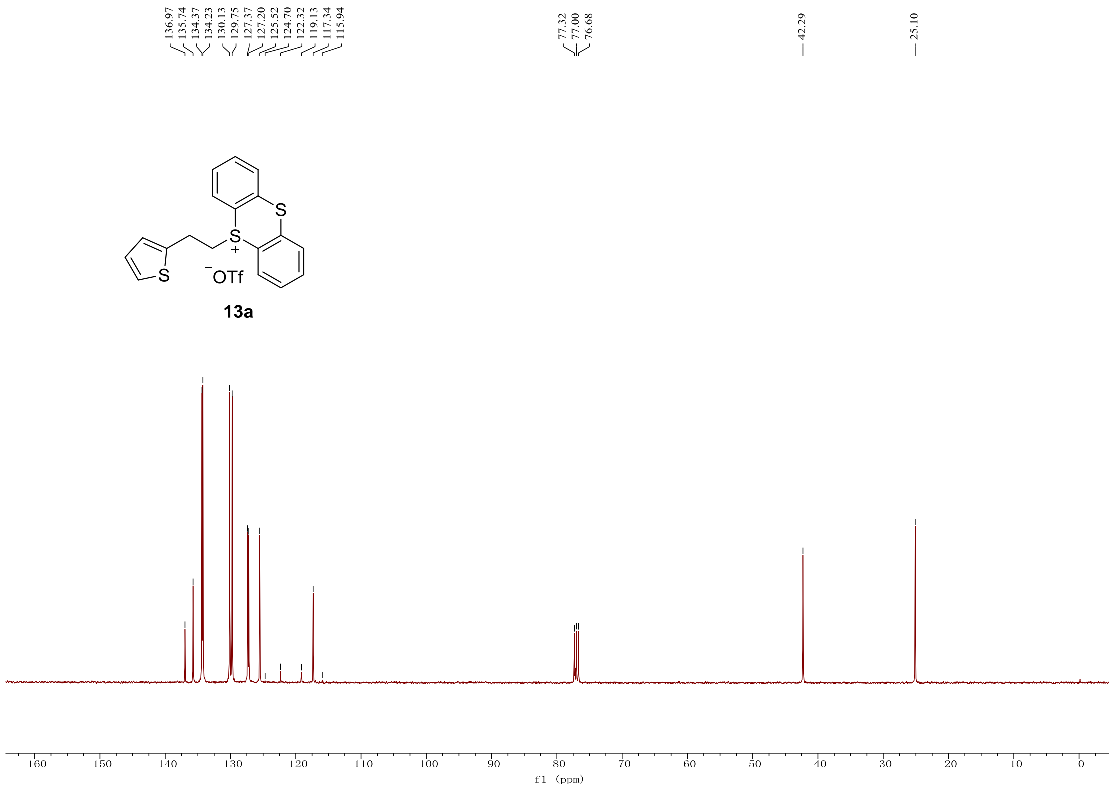

Supplementary Figures 56. ^{13}**C NMR spectrum for 13a.**

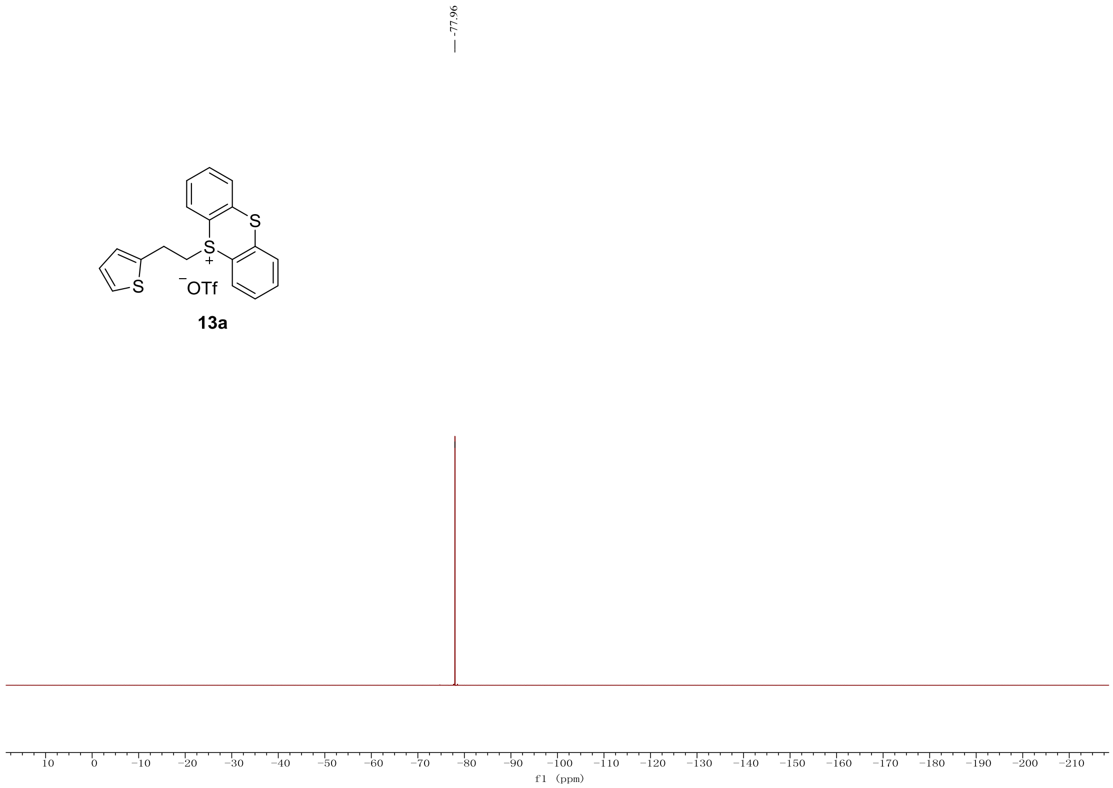

Supplementary Figure 57. ^{19}**F NMR spectrum for 13a.**

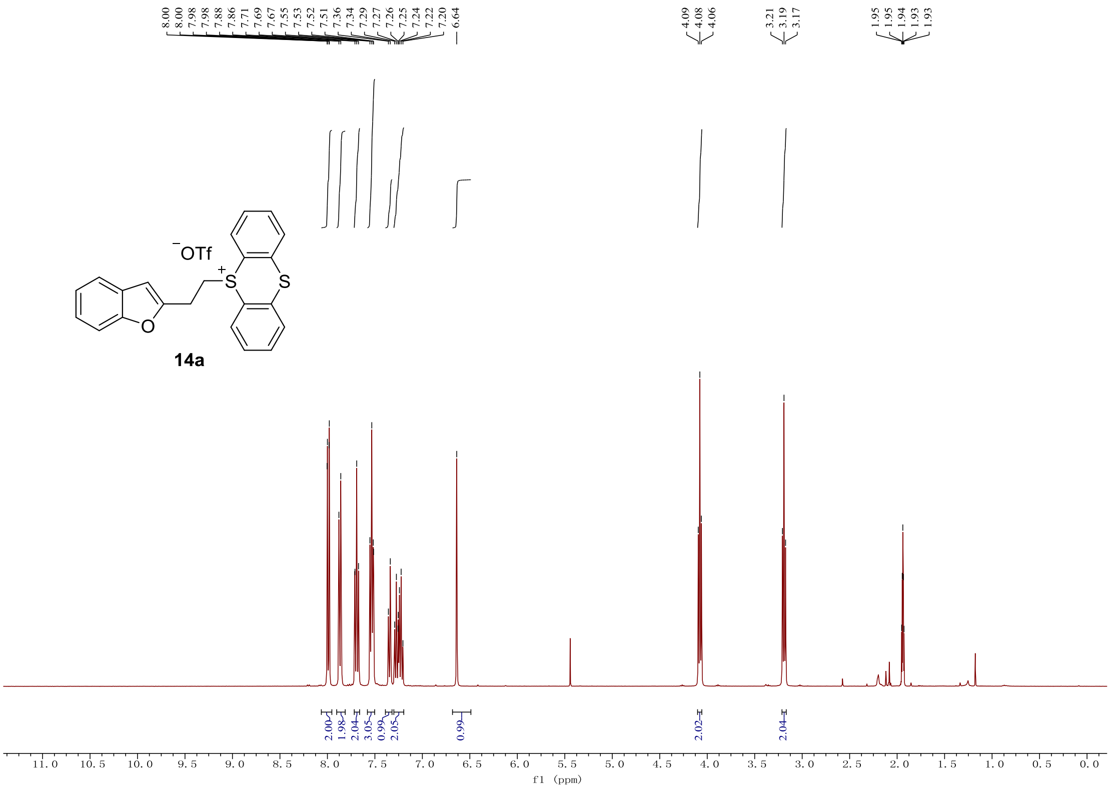

Supplementary Figure 58. ^{1}H NMR spectrum for 14a.

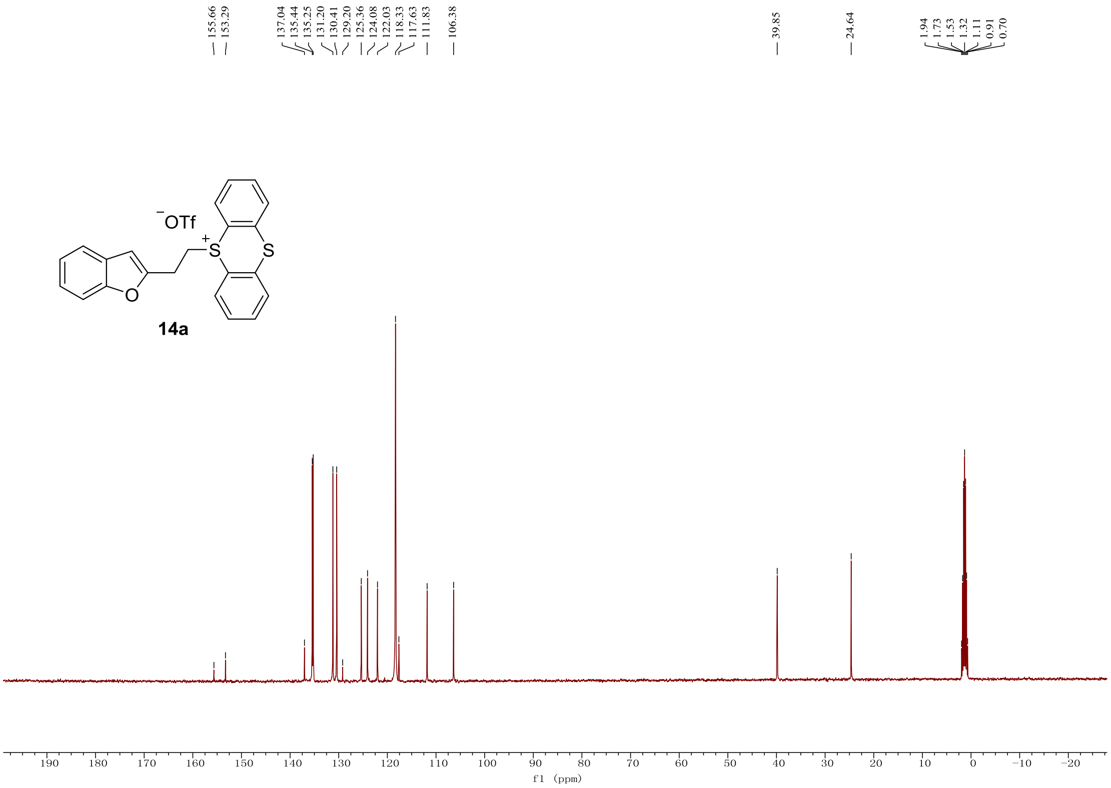

Supplementary Figures 59. ^{13}C NMR spectrum for 14a.

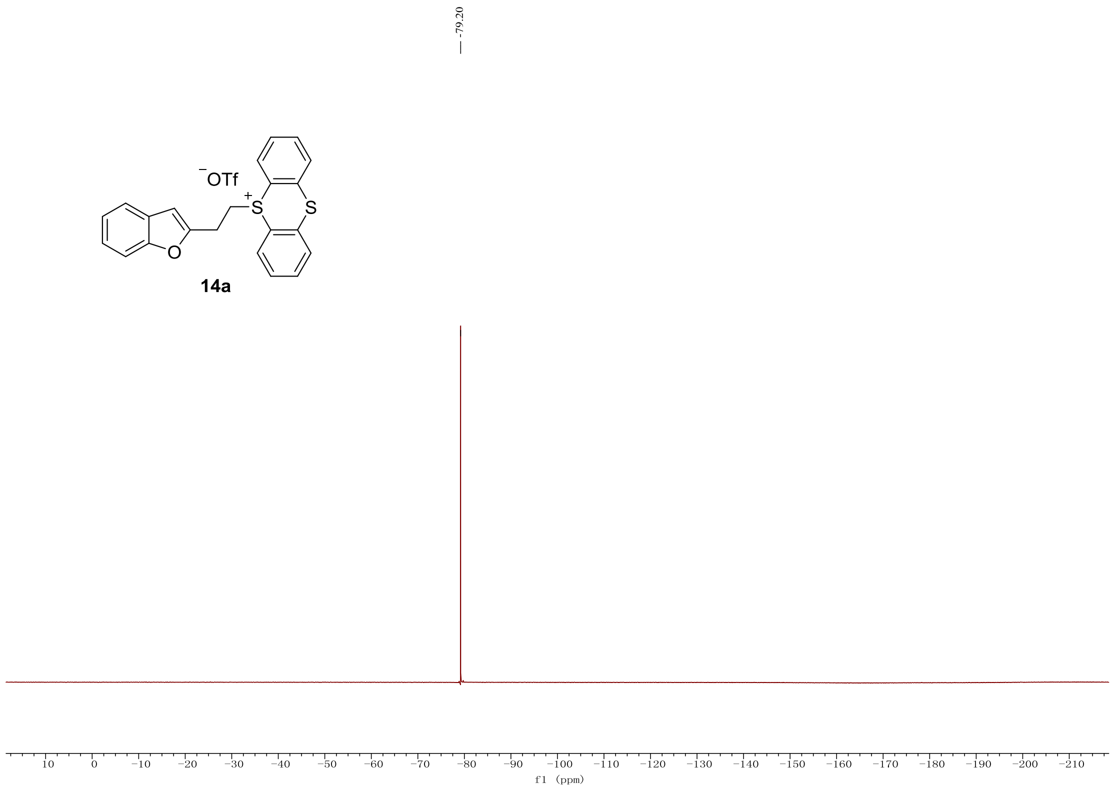

Supplementary Figure 60. ^{19}F NMR spectrum for 14a.

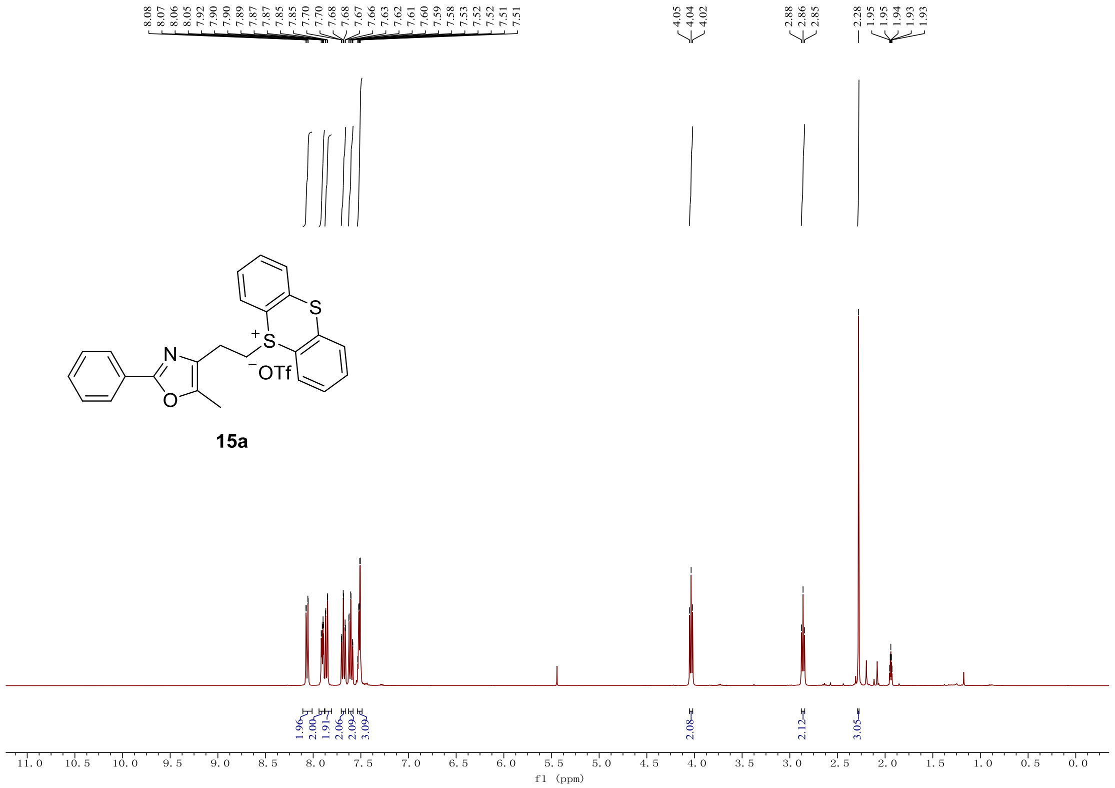

Supplementary Figure 61. ^{1}H NMR spectrum for 15a.

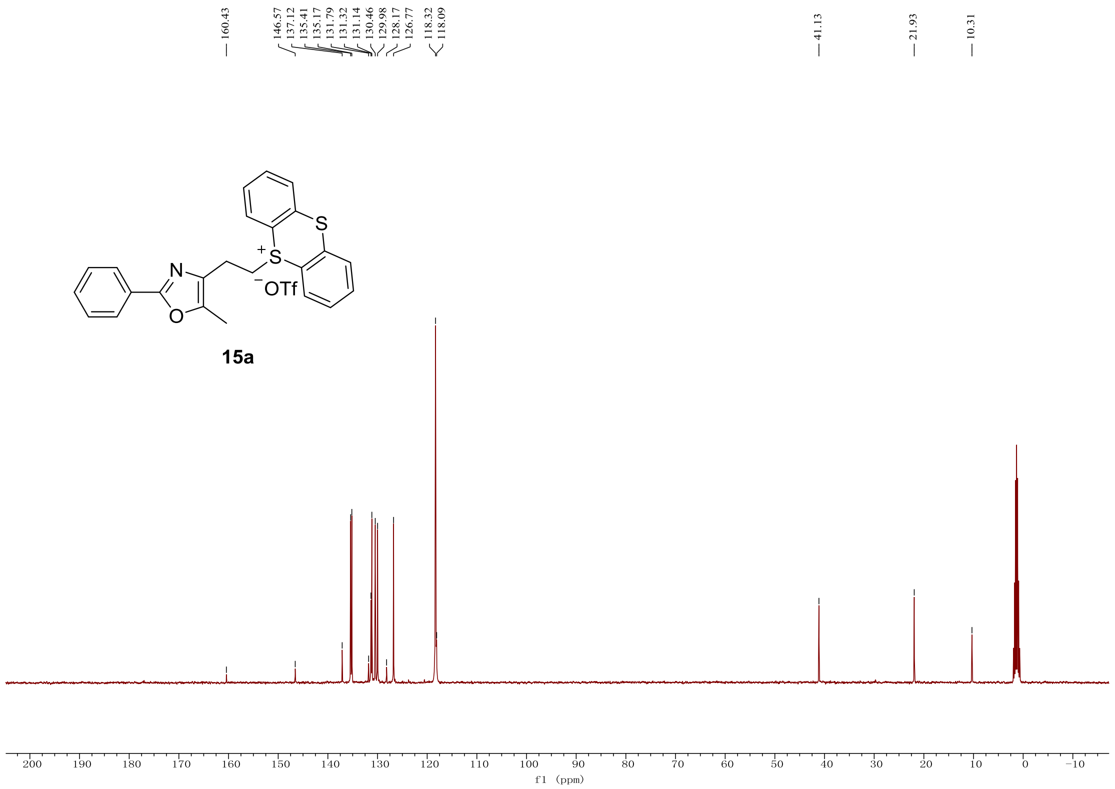

Supplementary Figures 62. ^{13}C NMR spectrum for 15a.

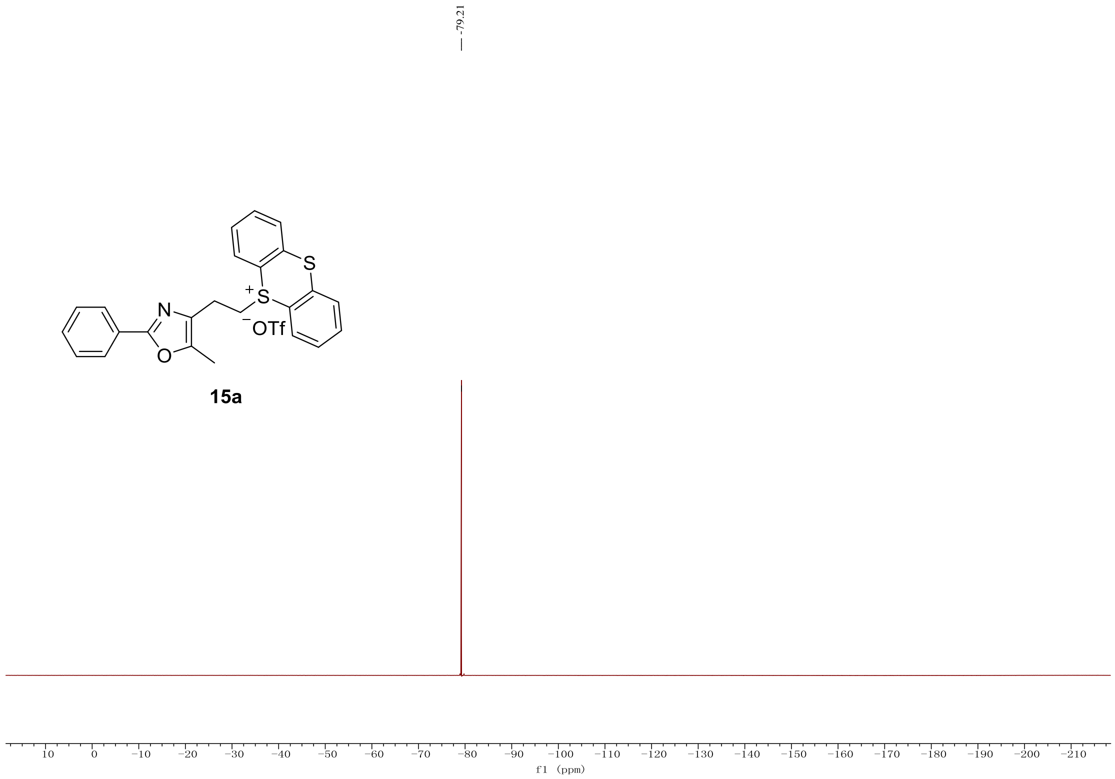

Supplementary Figure 63. ^{19}F NMR spectrum for 15a.

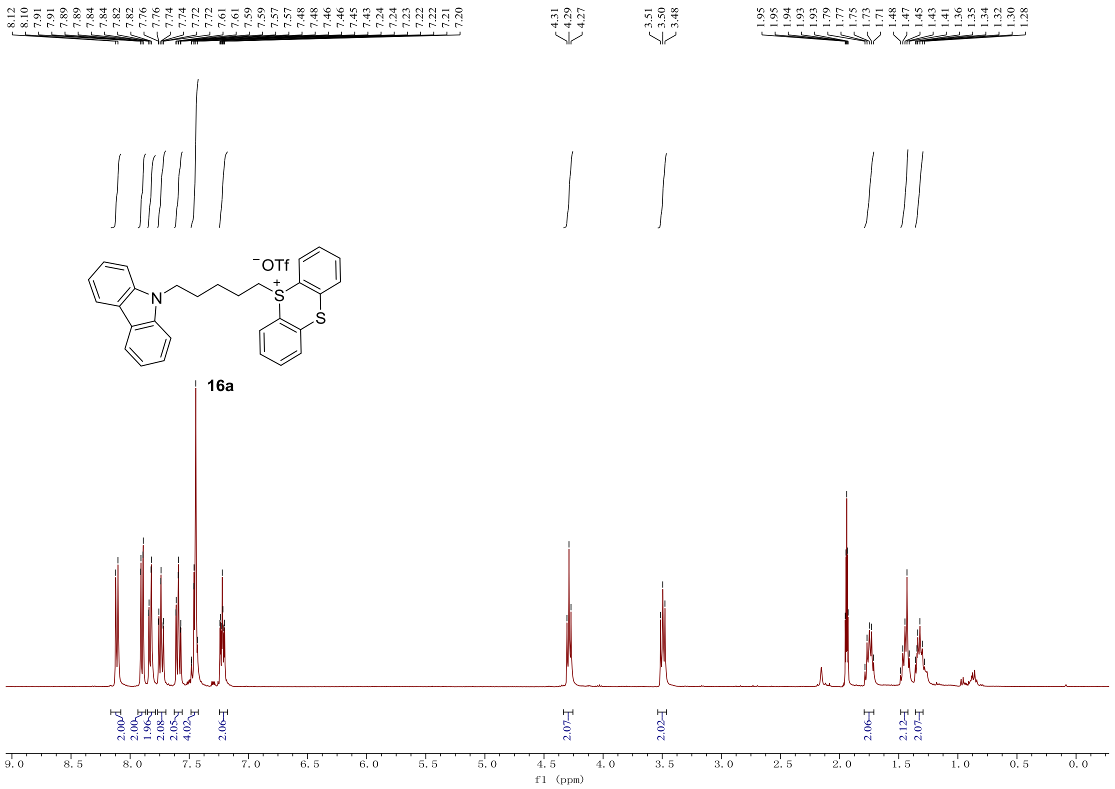

Supplementary Figure 64. ^{1}H NMR spectrum for 16a.

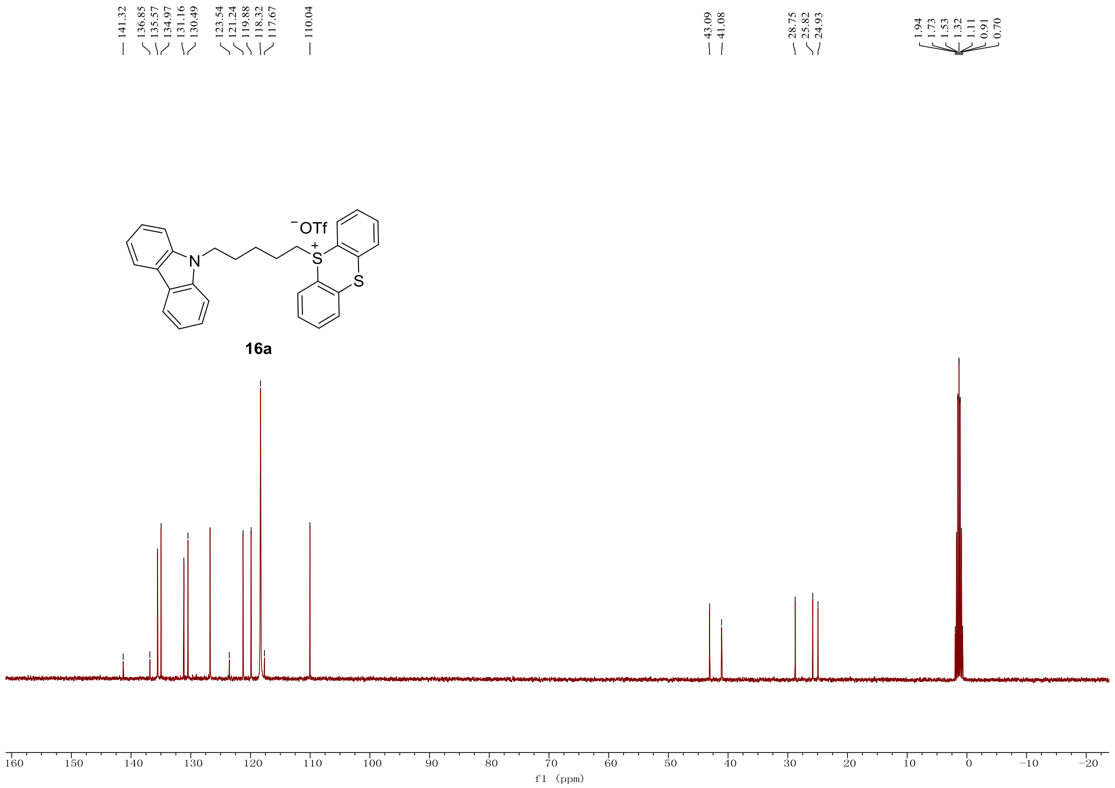

Supplementary Figures 65. ^{13}C NMR spectrum for 16a.

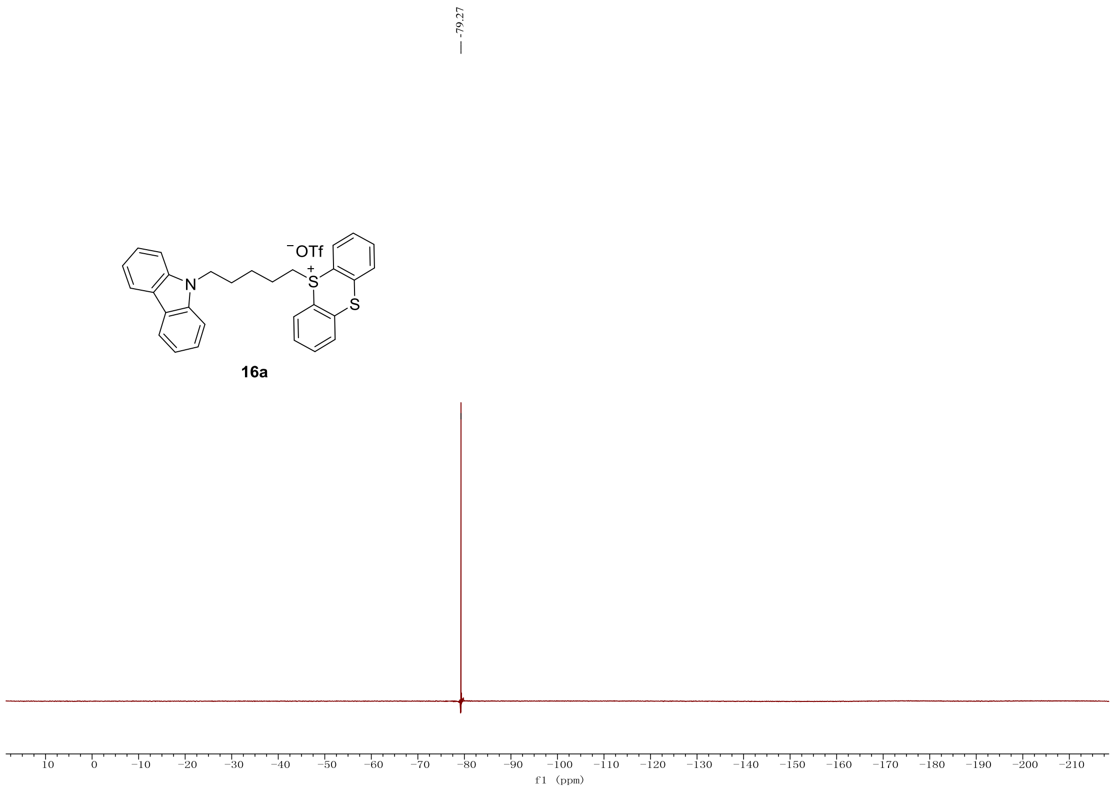

Supplementary Figure 66. ^{19}F NMR spectrum for 16a.

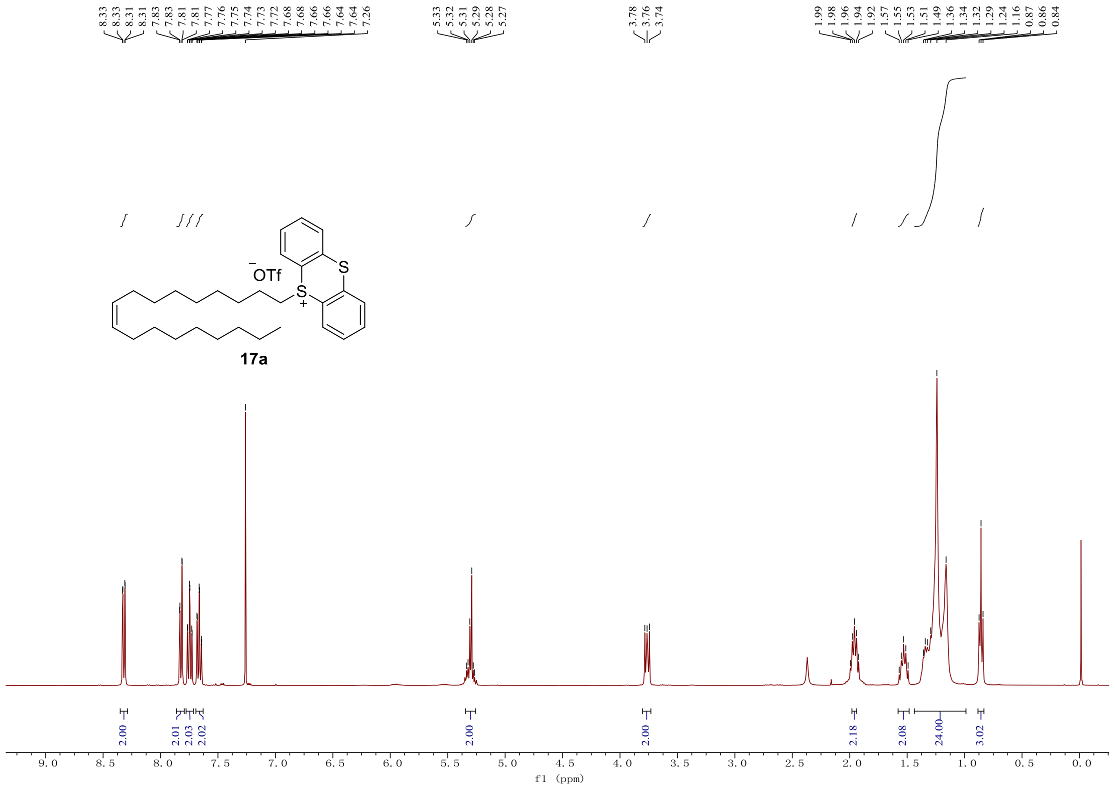

Supplementary Figure 67. ^{1}H NMR spectrum for 17a.

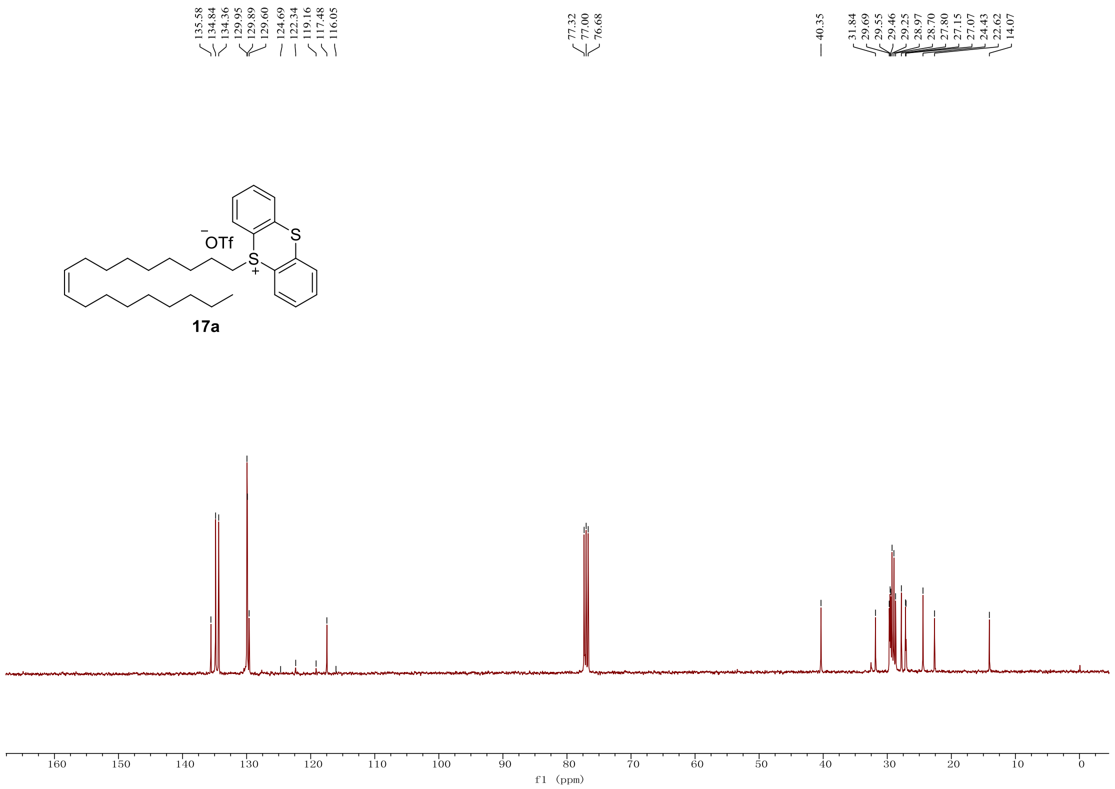

Supplementary Figures 68. ^{13}C NMR spectrum for 17a.

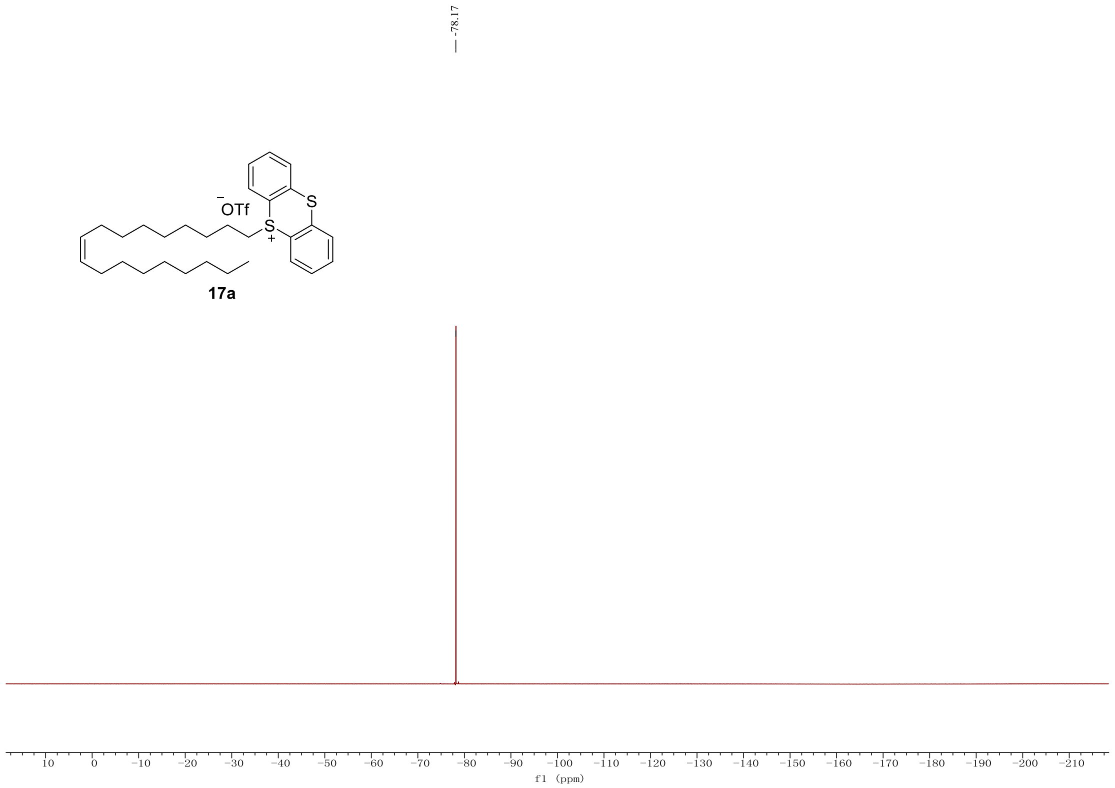

Supplementary Figure 69. ^{19}F NMR spectrum for 17a.

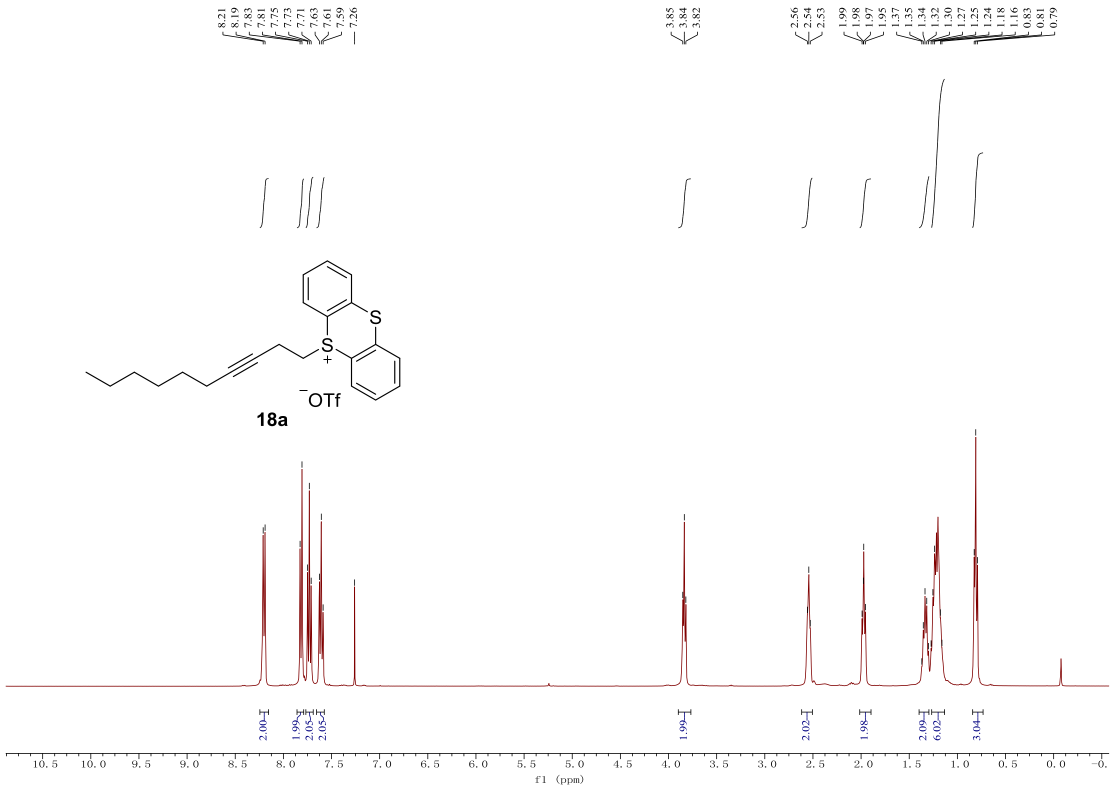

Supplementary Figure 70. ^{1}H NMR spectrum for 18a.

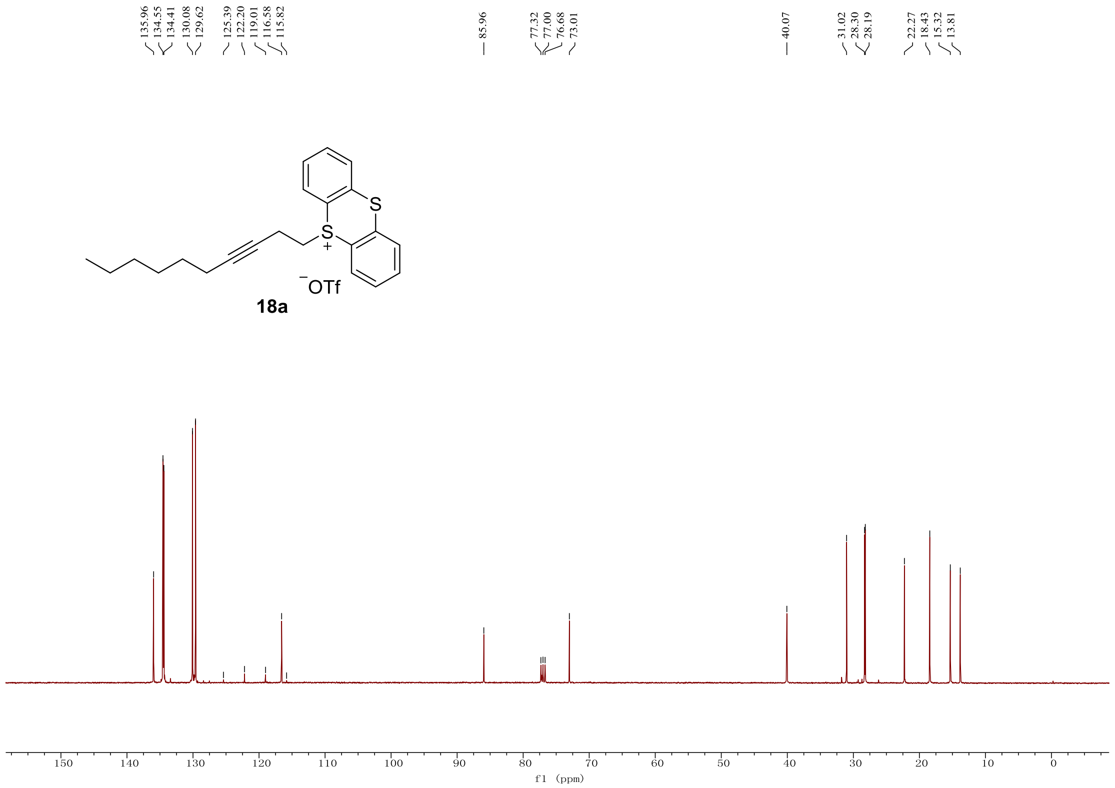

Supplementary Figures 71. ^{13}C NMR spectrum for 18a.

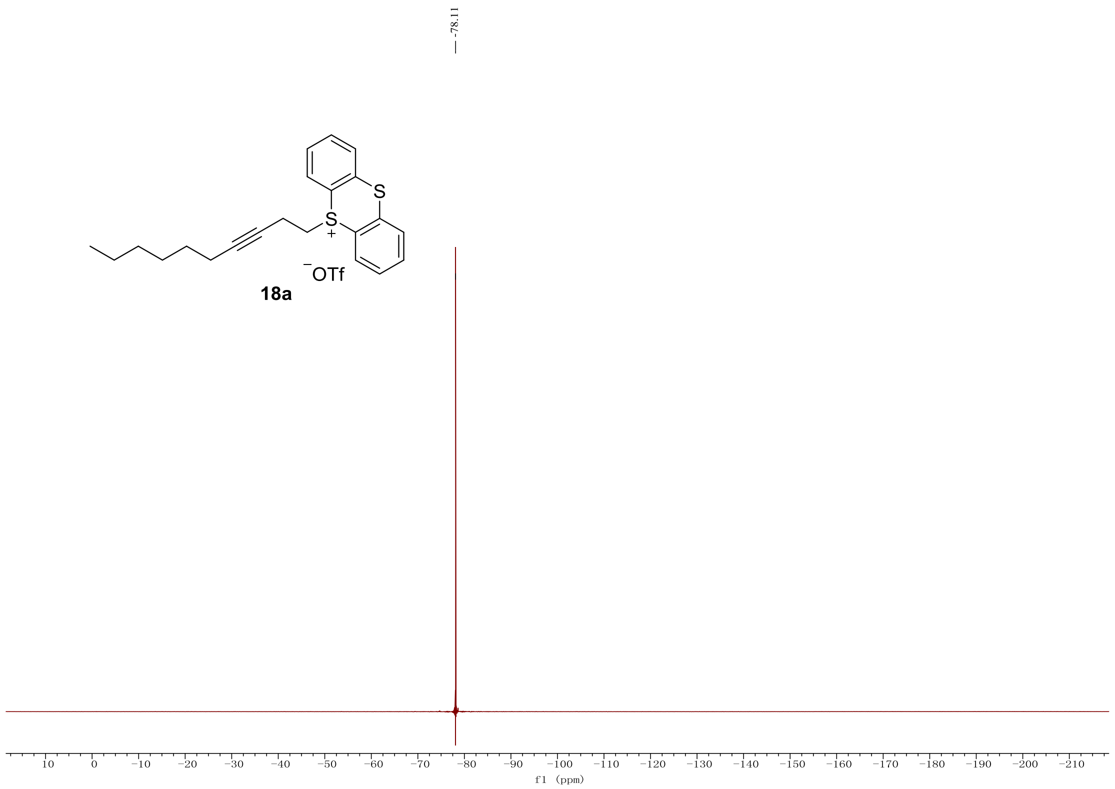

Supplementary Figure 72. ^{19}F NMR spectrum for 18a.

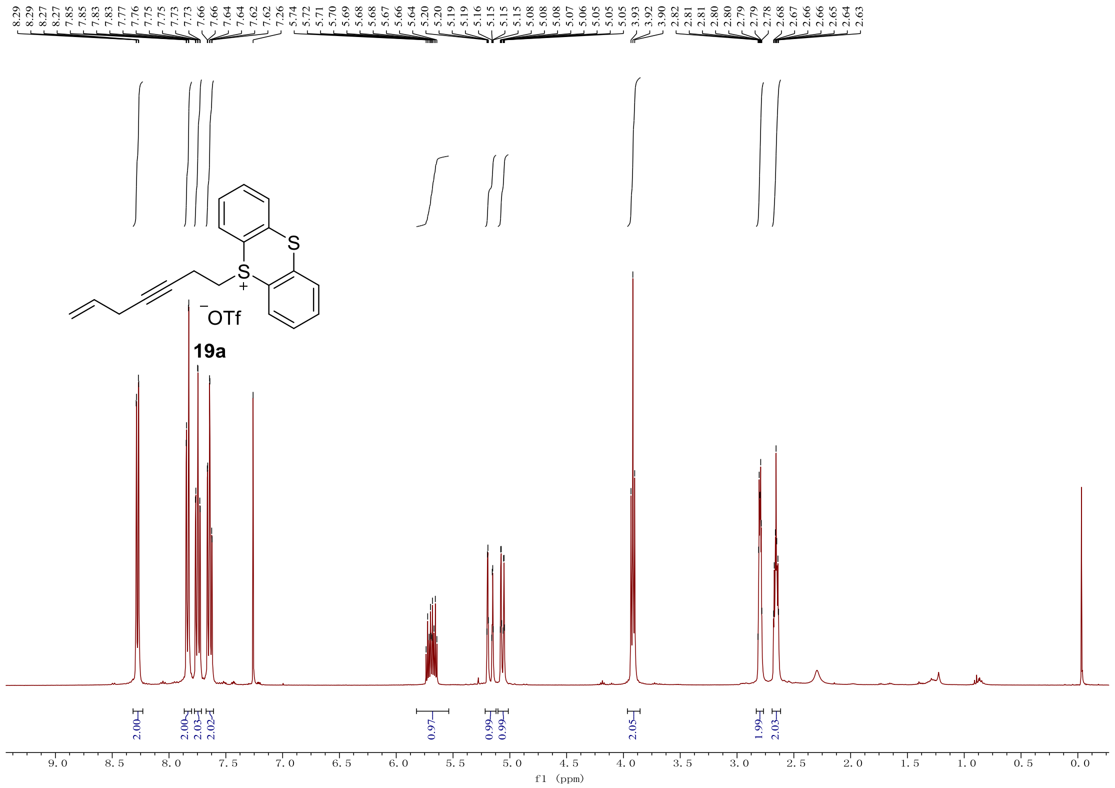

Supplementary Figure 73. ^{1}H NMR spectrum for 19a.

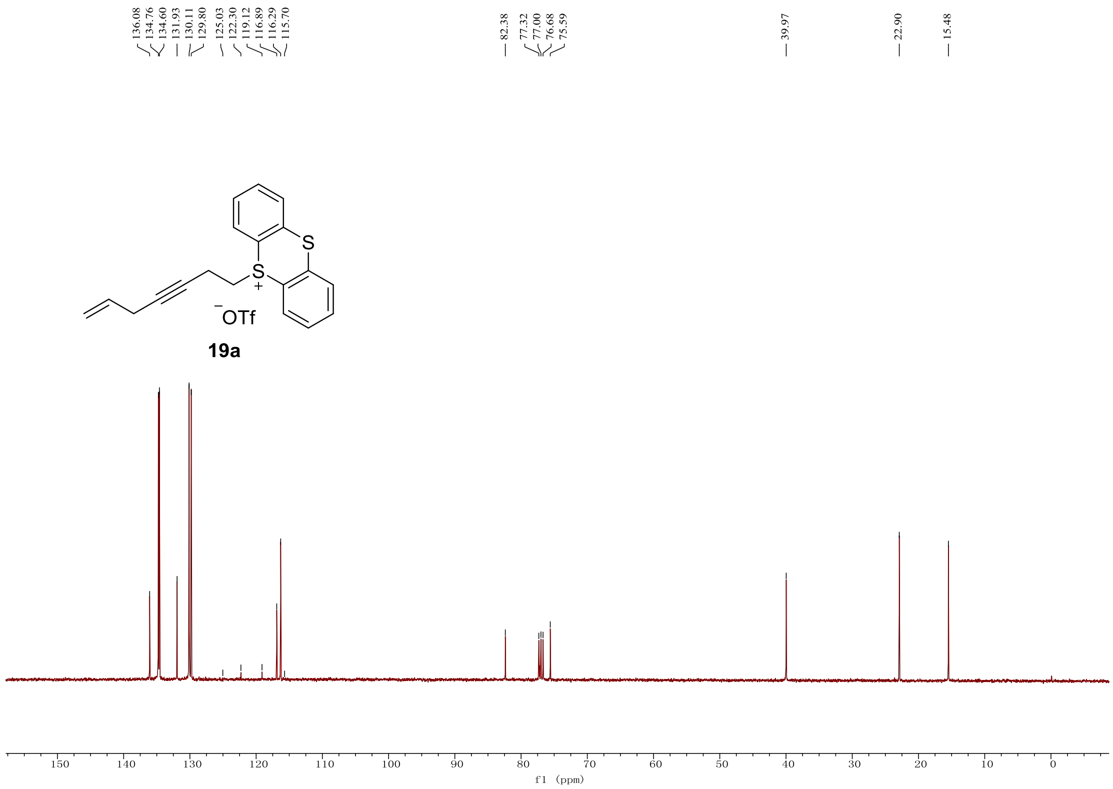

Supplementary Figures 74. ^{13}C NMR spectrum for 19a.

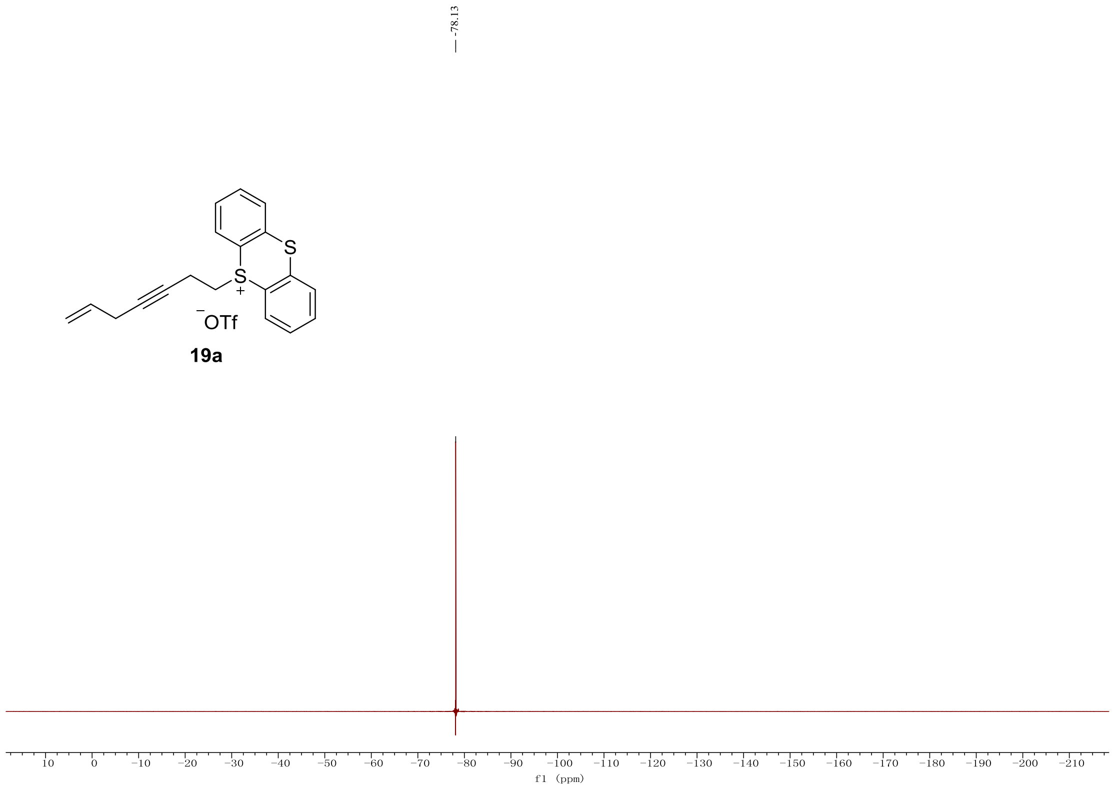

Supplementary Figure 75. ^{19}F NMR spectrum for 19a.

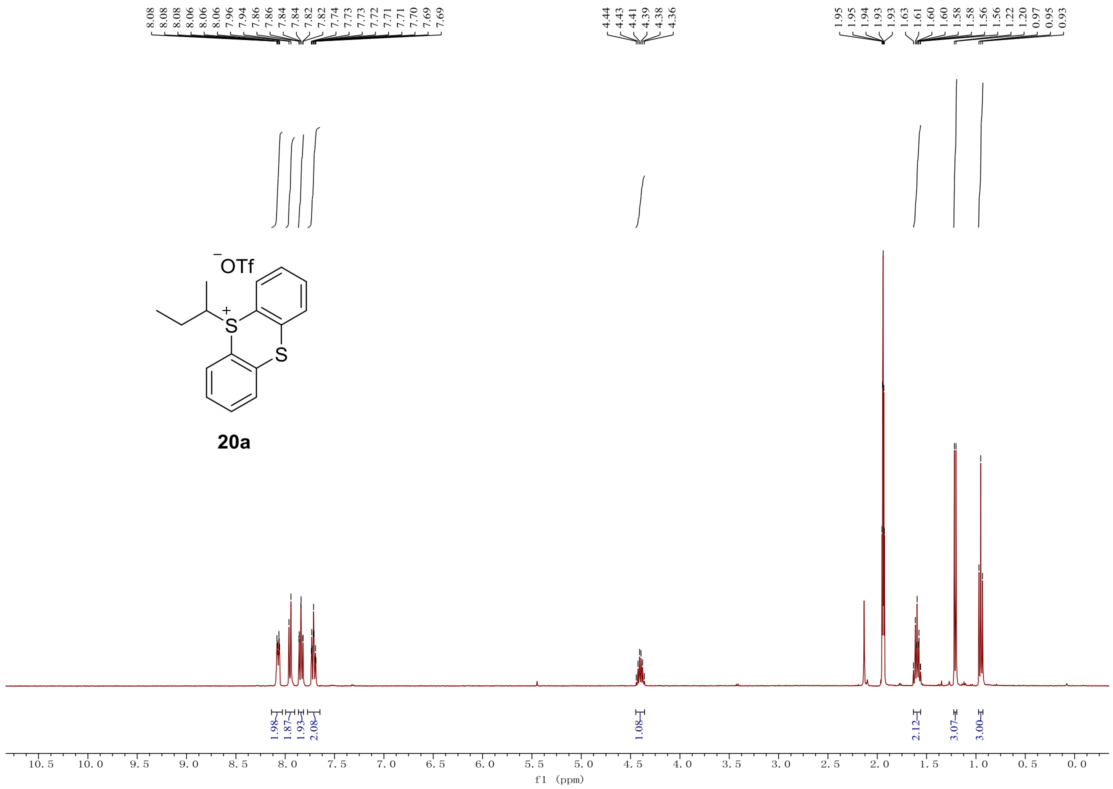

Supplementary Figure 76. ^{1}H NMR spectrum for 20a.

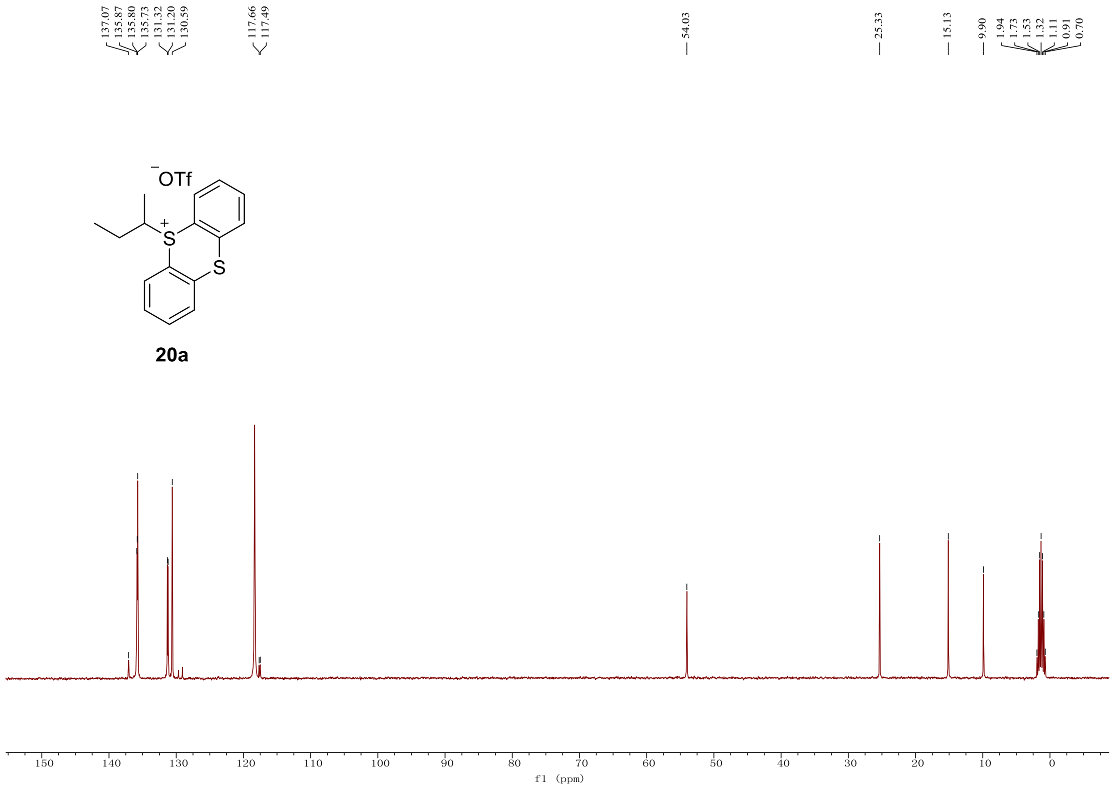

Supplementary Figures 77. ^{13}C NMR spectrum for 20a.

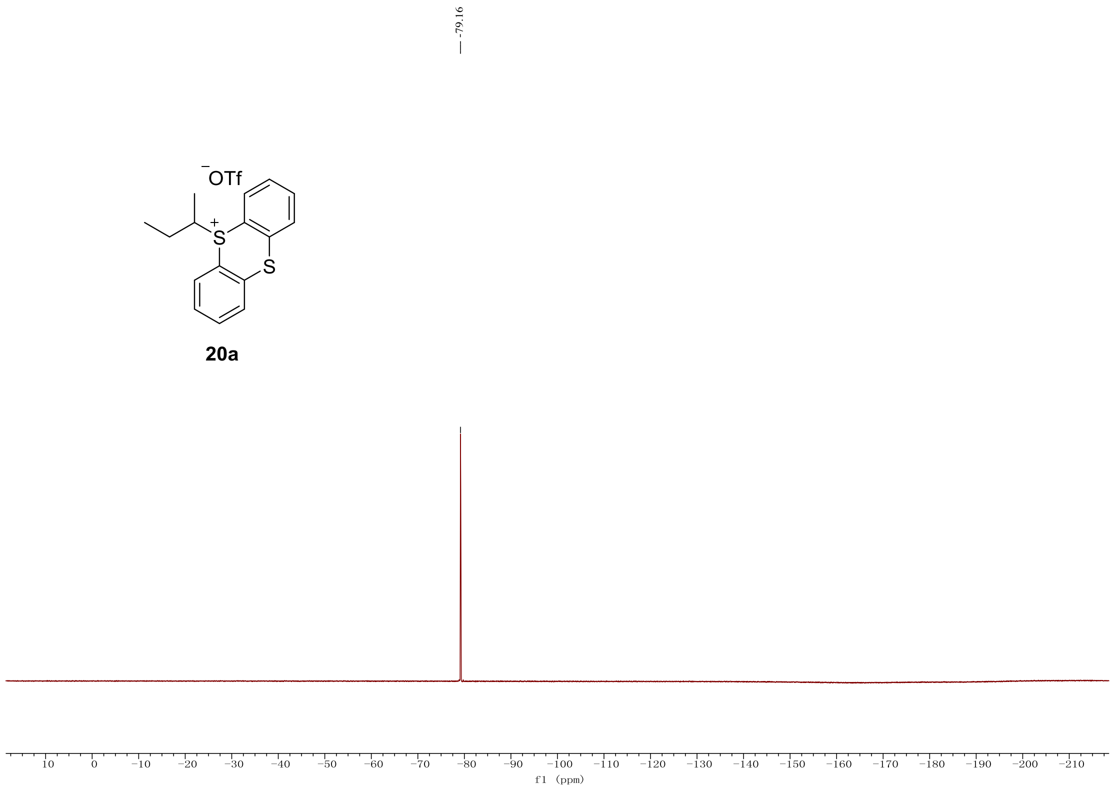

Supplementary Figure 78. ^{19}**F NMR spectrum for 20a.**

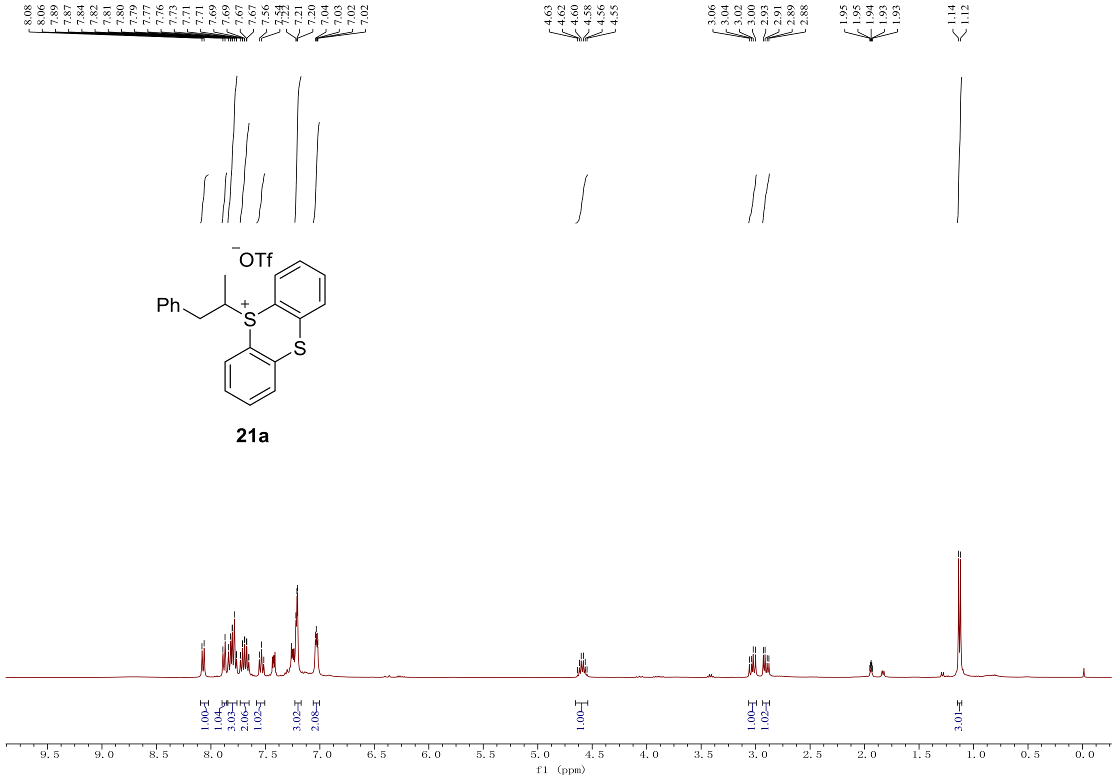

Supplementary Figure 79. ^{1}**H NMR spectrum for 21a.**

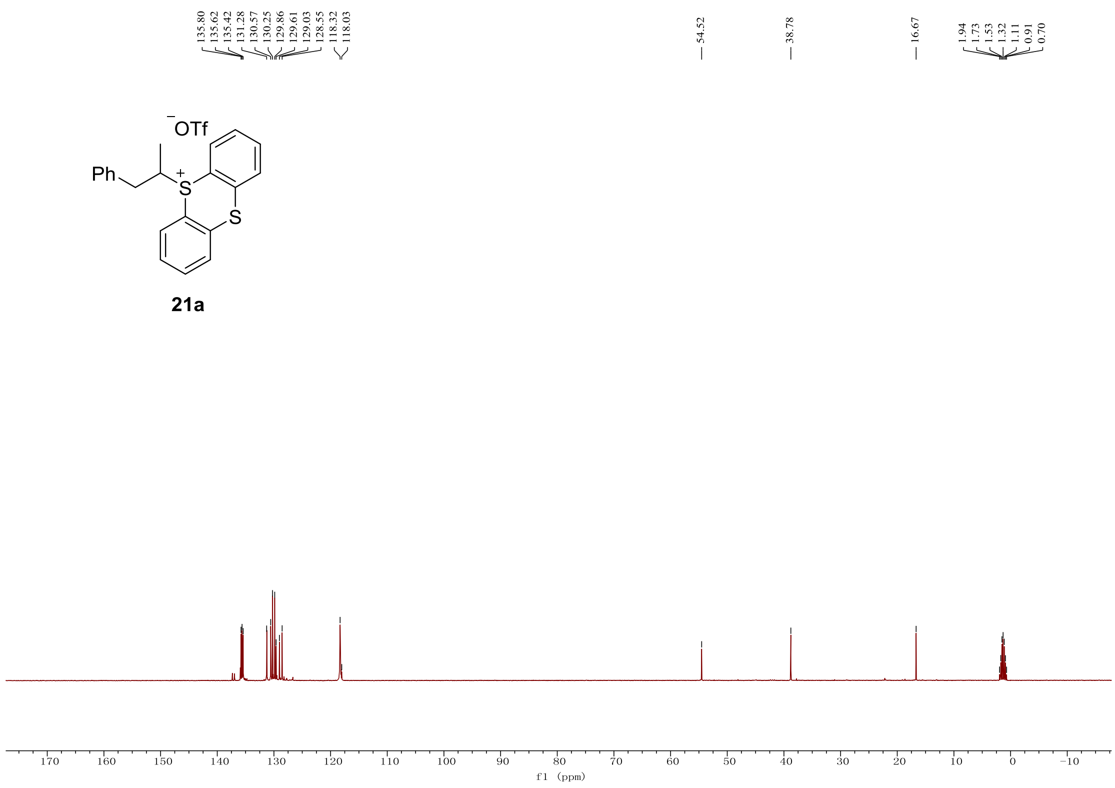

Supplementary Figures 80. ^{13}**C NMR spectrum for 21a.**

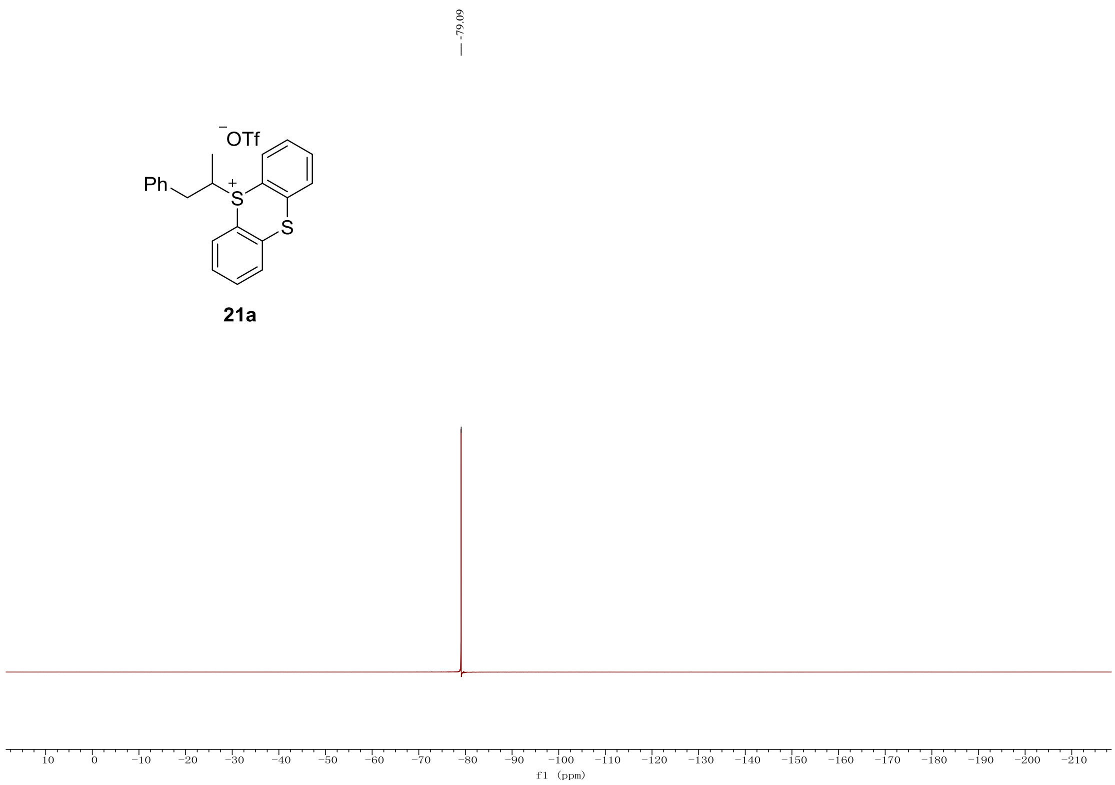

Supplementary Figure 81. ^{19}**F NMR spectrum for 21a.**

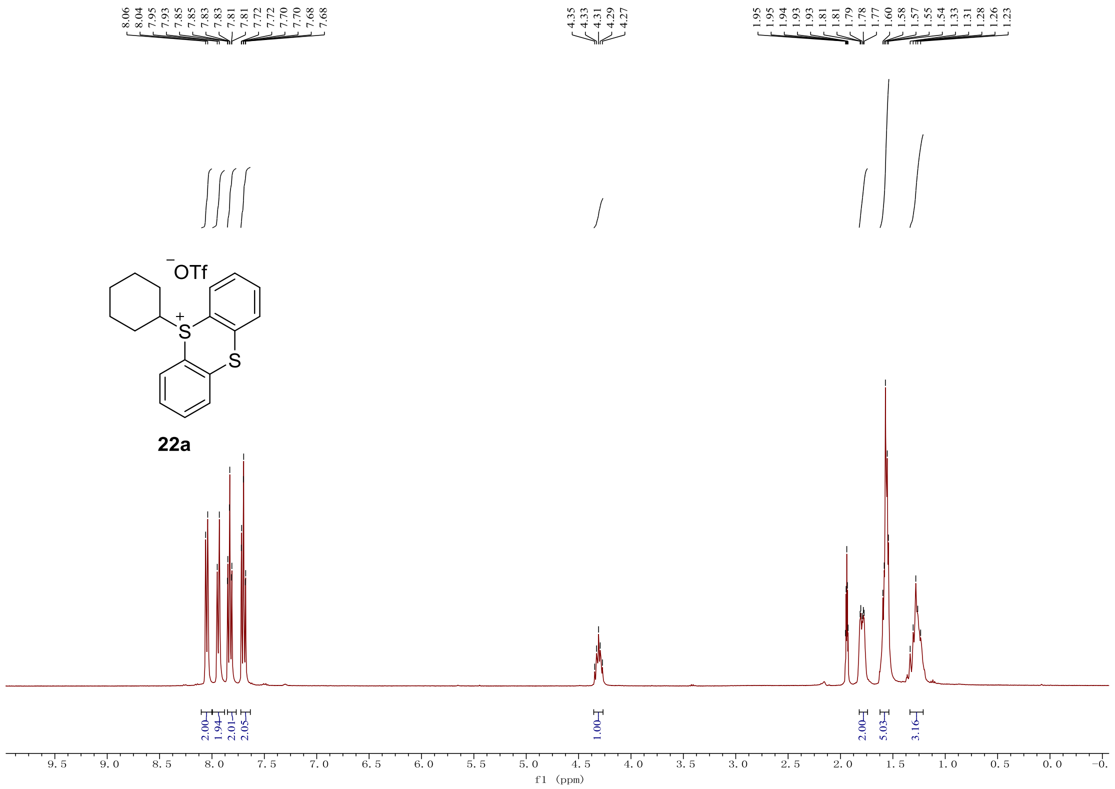

Supplementary Figure 82. ^{1}H NMR spectrum for 22a.

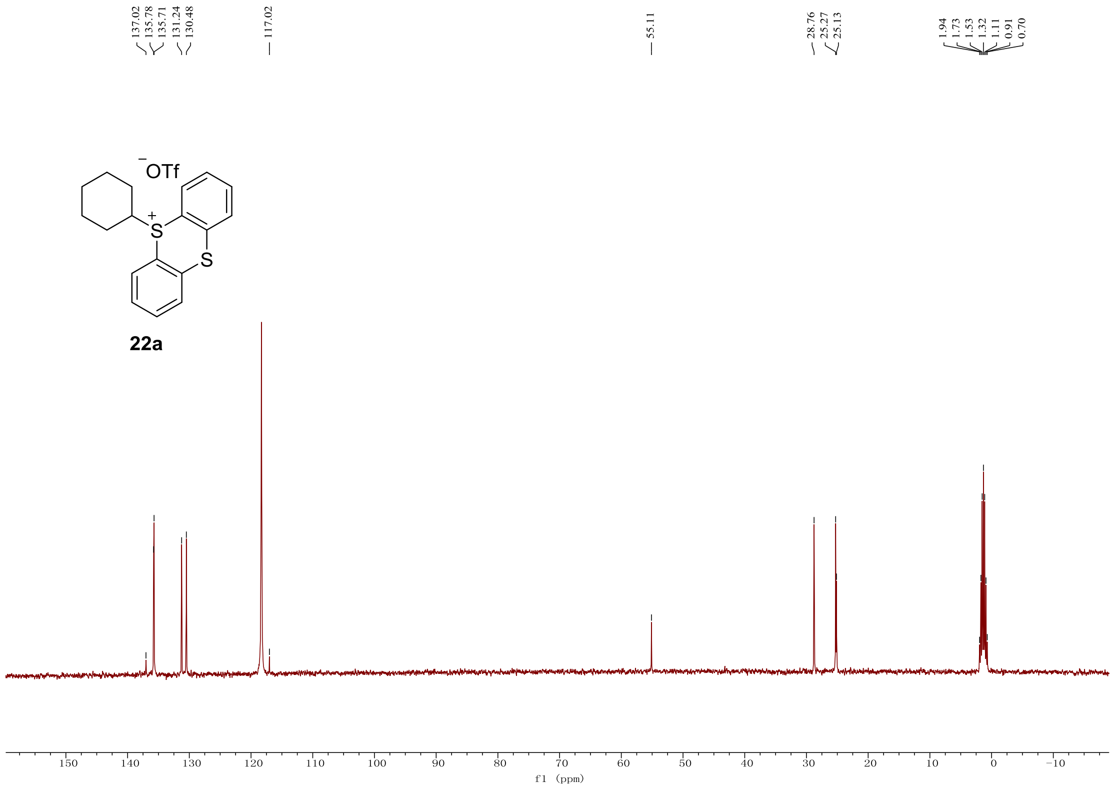

Supplementary Figures 83. ^{13}C NMR spectrum for 22a.

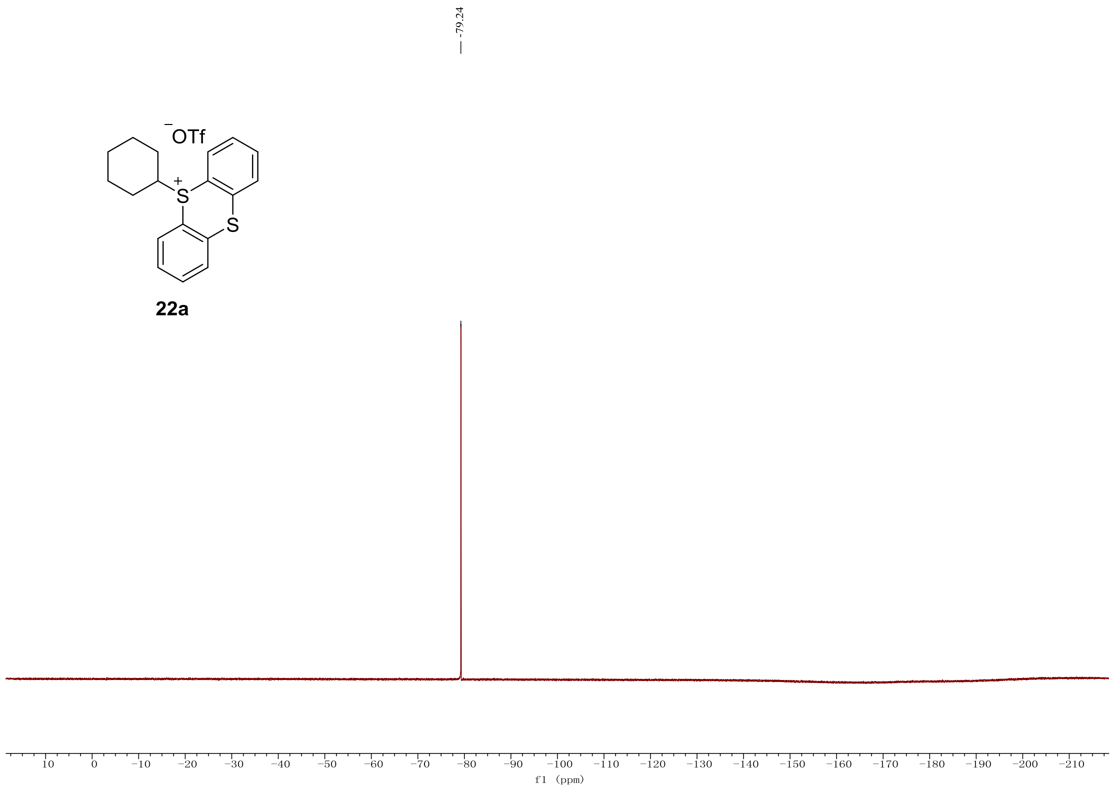

Supplementary Figure 84. ^{19}F NMR spectrum for 22a.

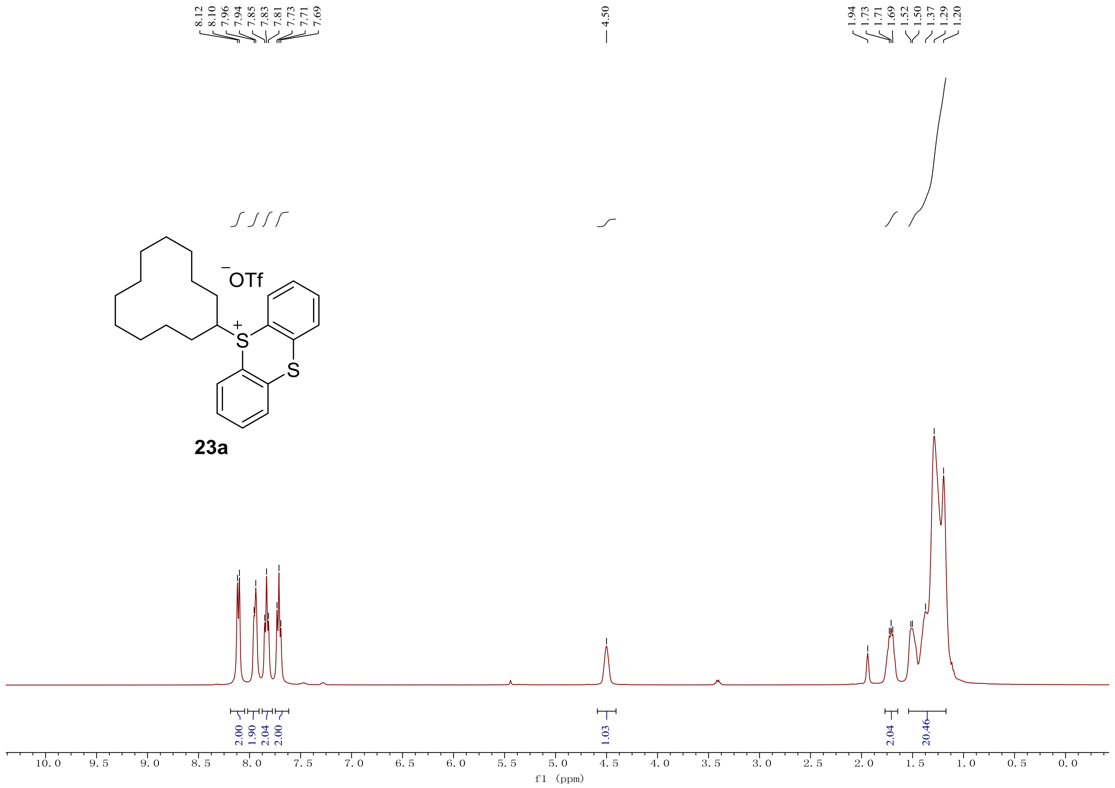

Supplementary Figure 85. ^{1}H NMR spectrum for 23a.

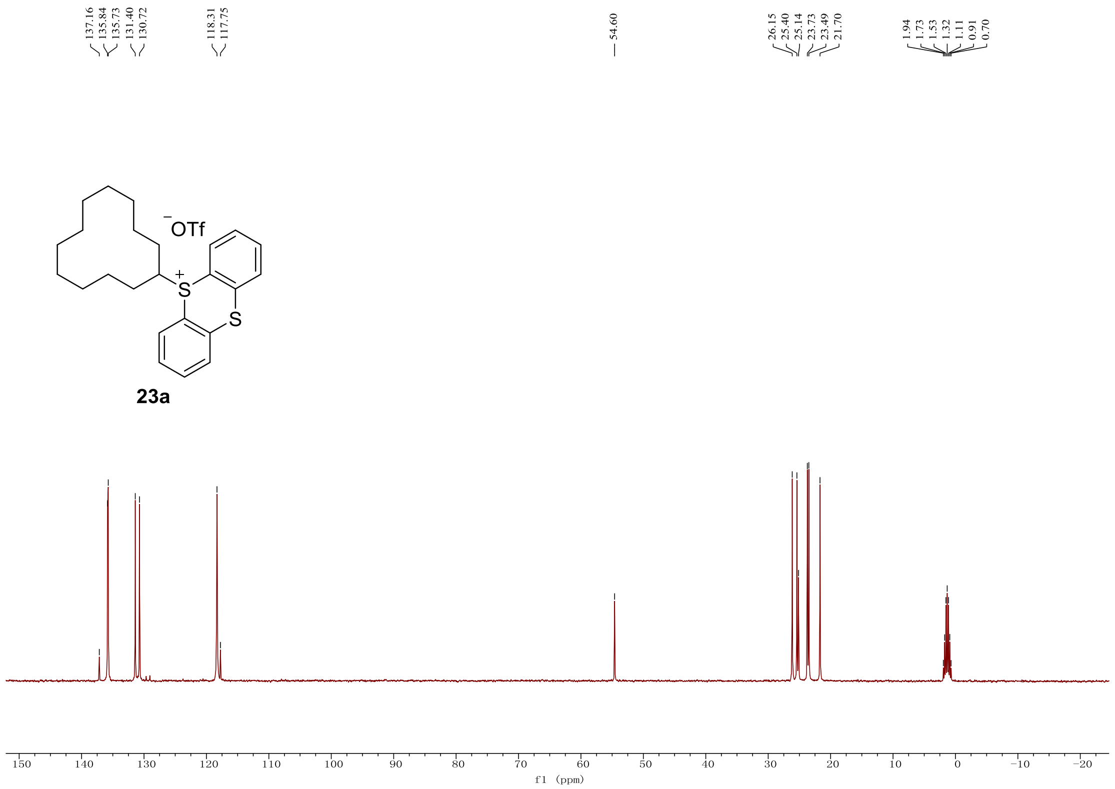

Supplementary Figures 86. ^{13}C NMR spectrum for 23a.

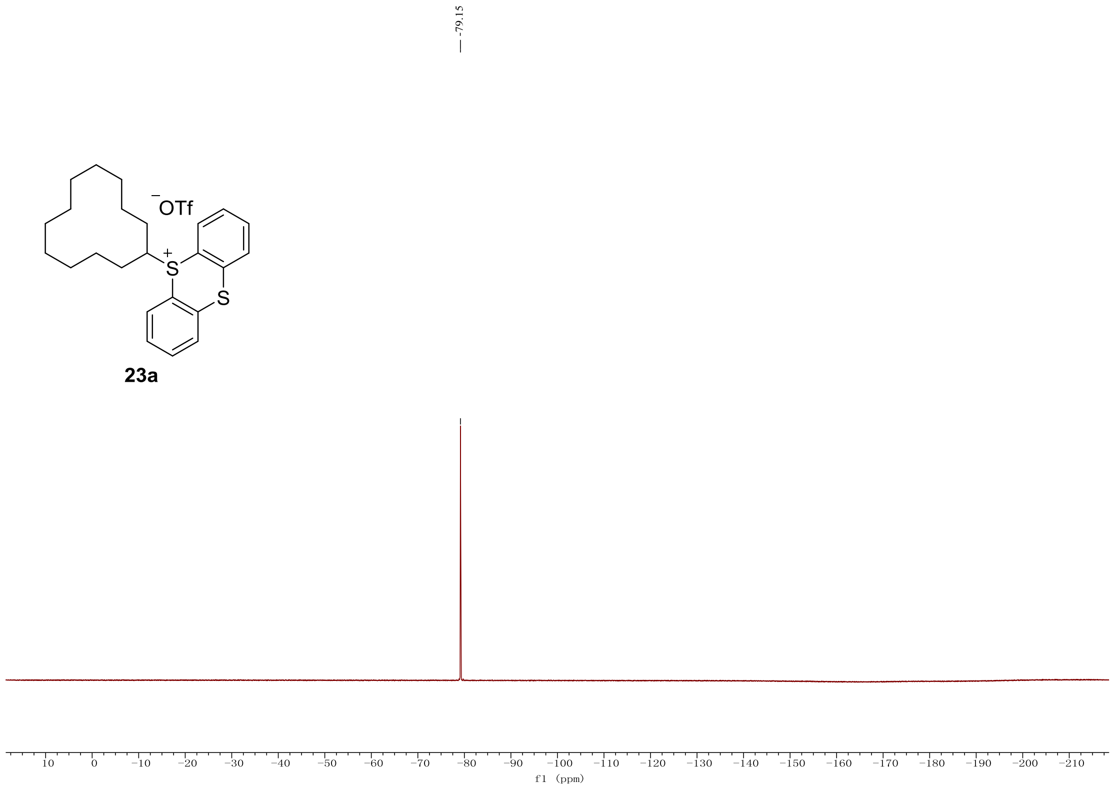

Supplementary Figure 87. ^{19}F NMR spectrum for 23a.

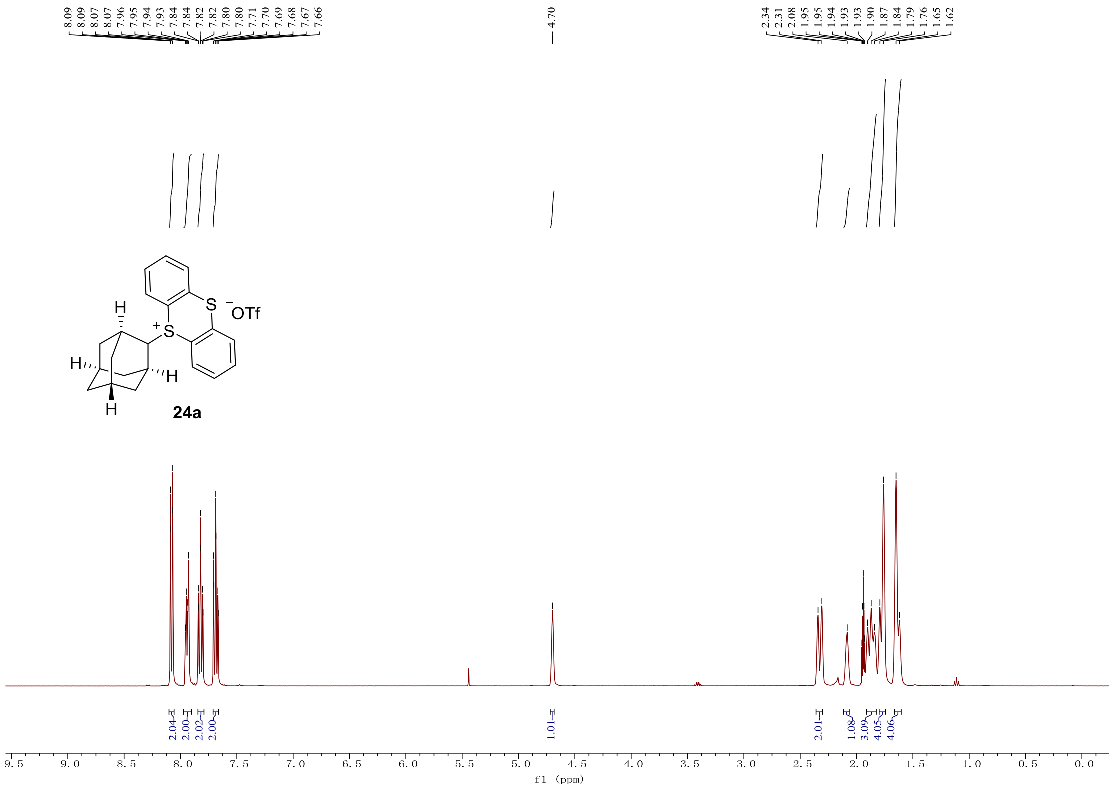

Supplementary Figure 88. ^{1}H NMR spectrum for 24a.

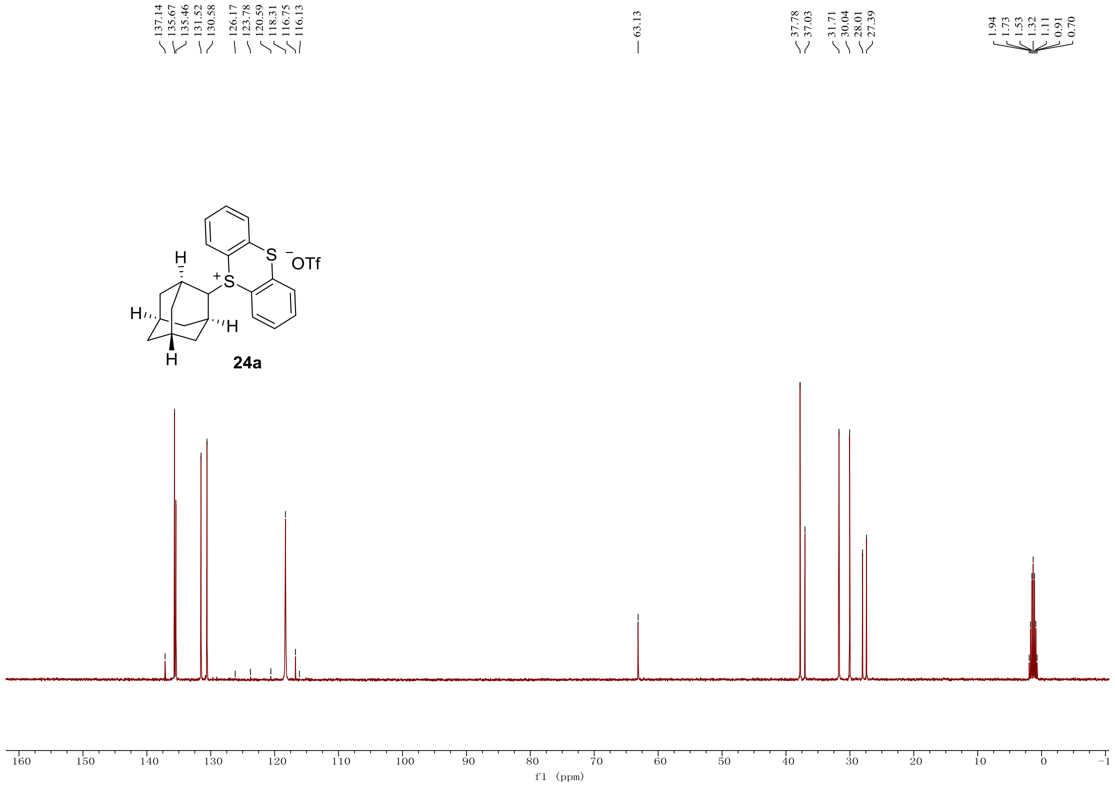

Supplementary Figures 89. ^{13}C NMR spectrum for 24a.

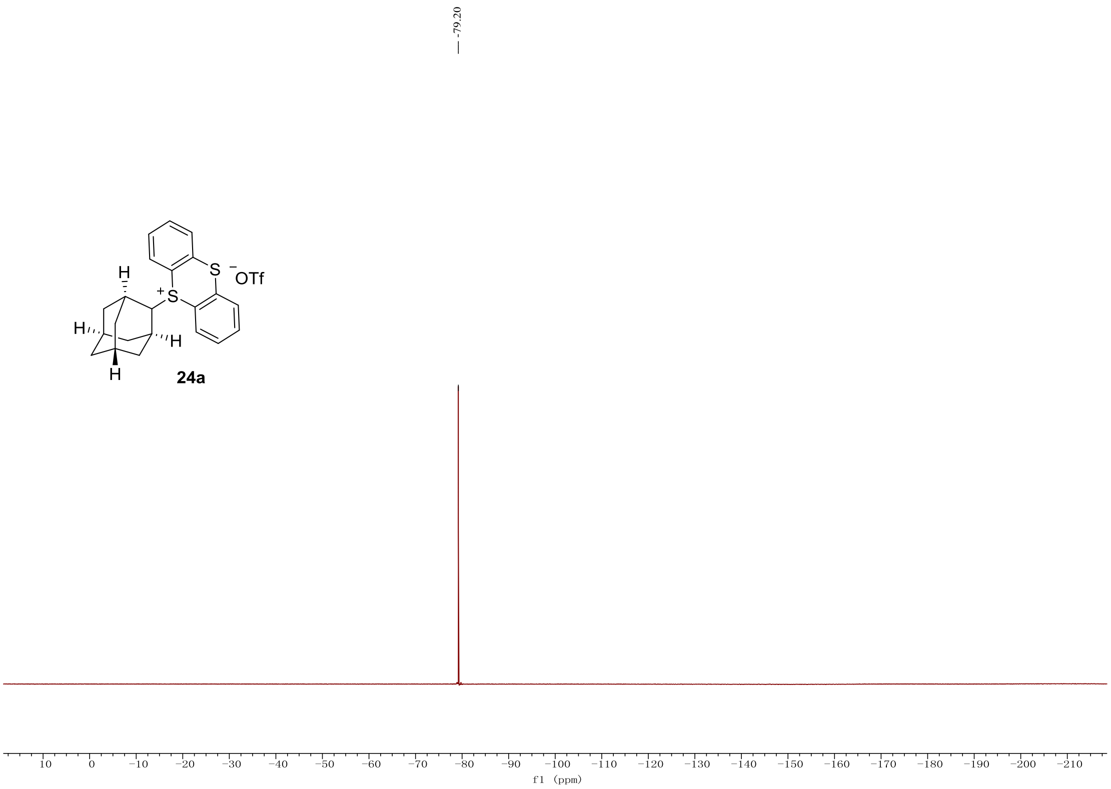

Supplementary Figure 90. ^{19}F NMR spectrum for 24a.

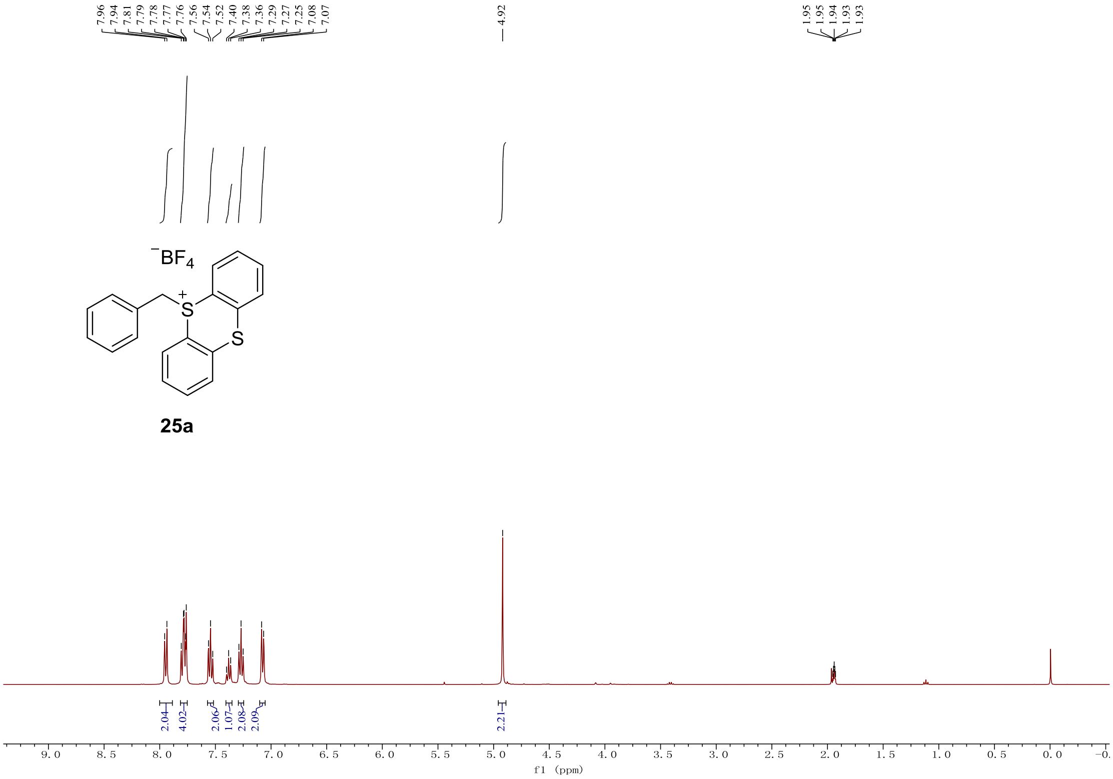

Supplementary Figure 91. ^{1}H NMR spectrum for 25a.

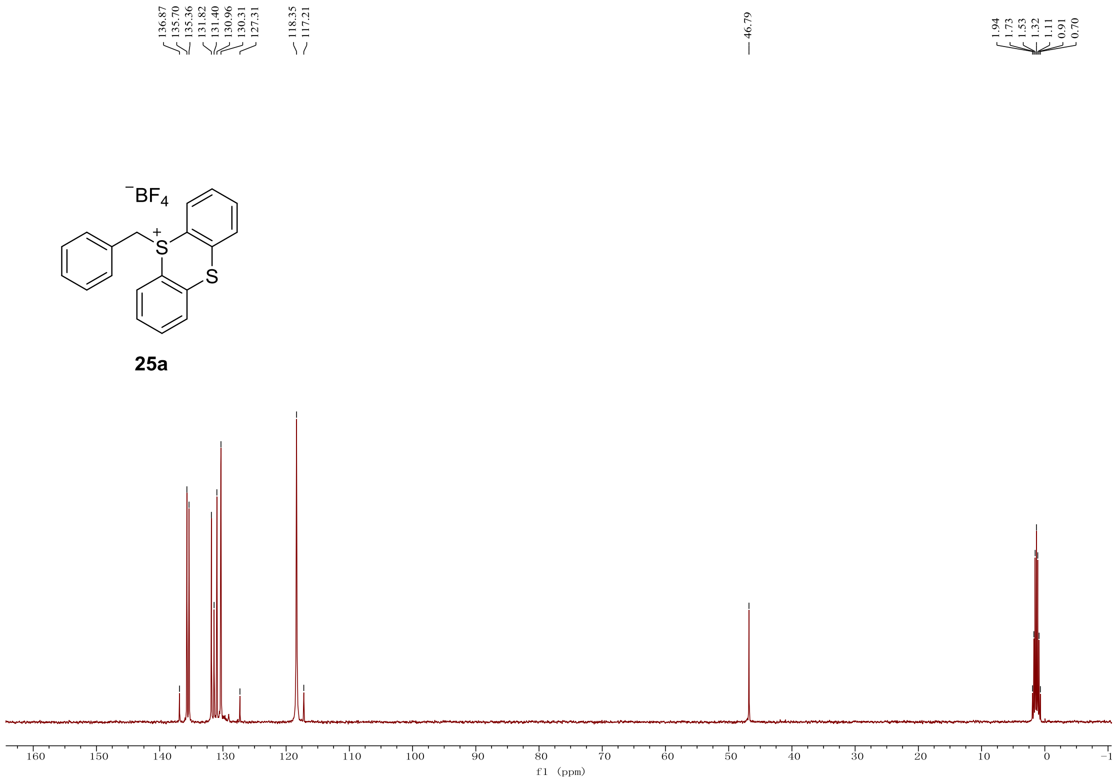

Supplementary Figures 92. ^{13}C NMR spectrum for 25a.

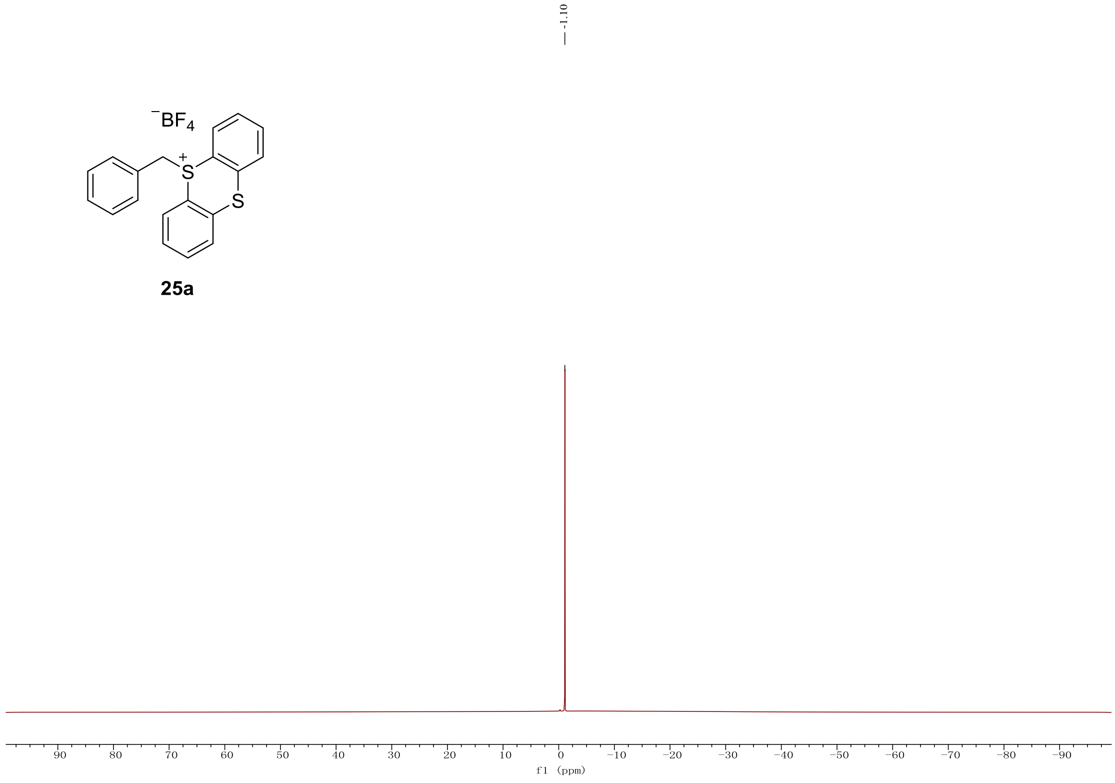

Supplementary Figure 93. ^{19}F NMR spectrum for 25a.

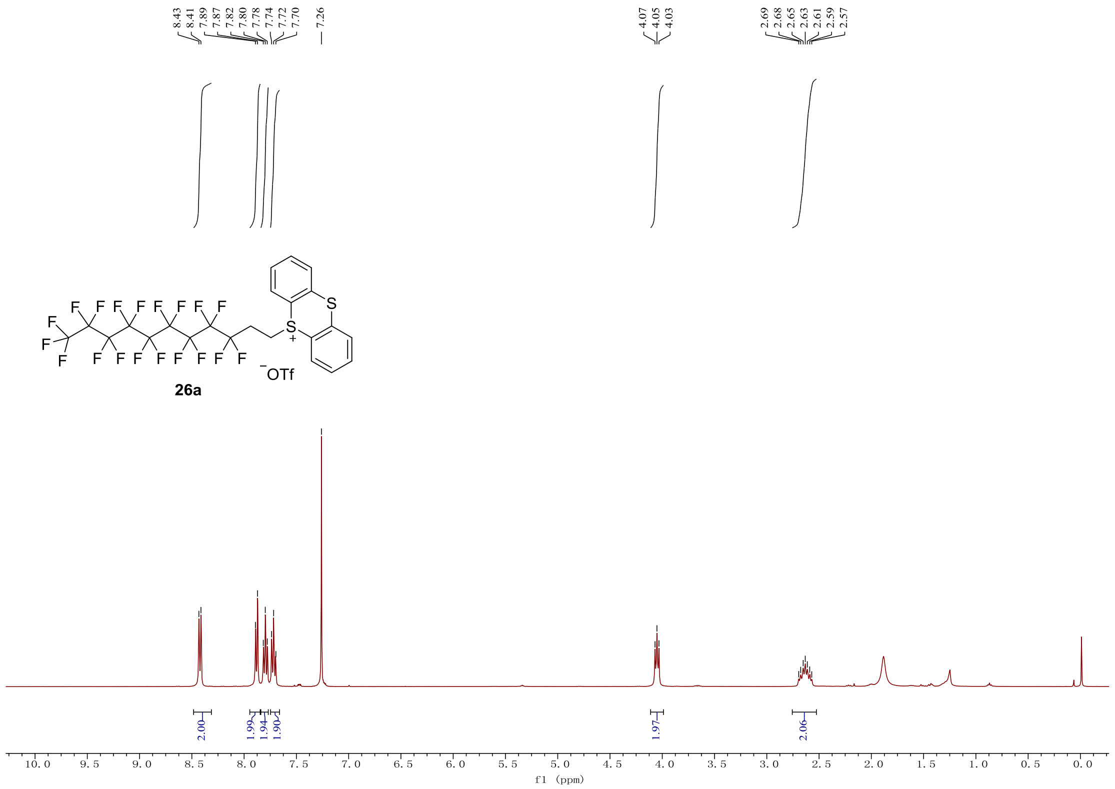

Supplementary Figure 94. ^{1}H NMR spectrum for 26a.

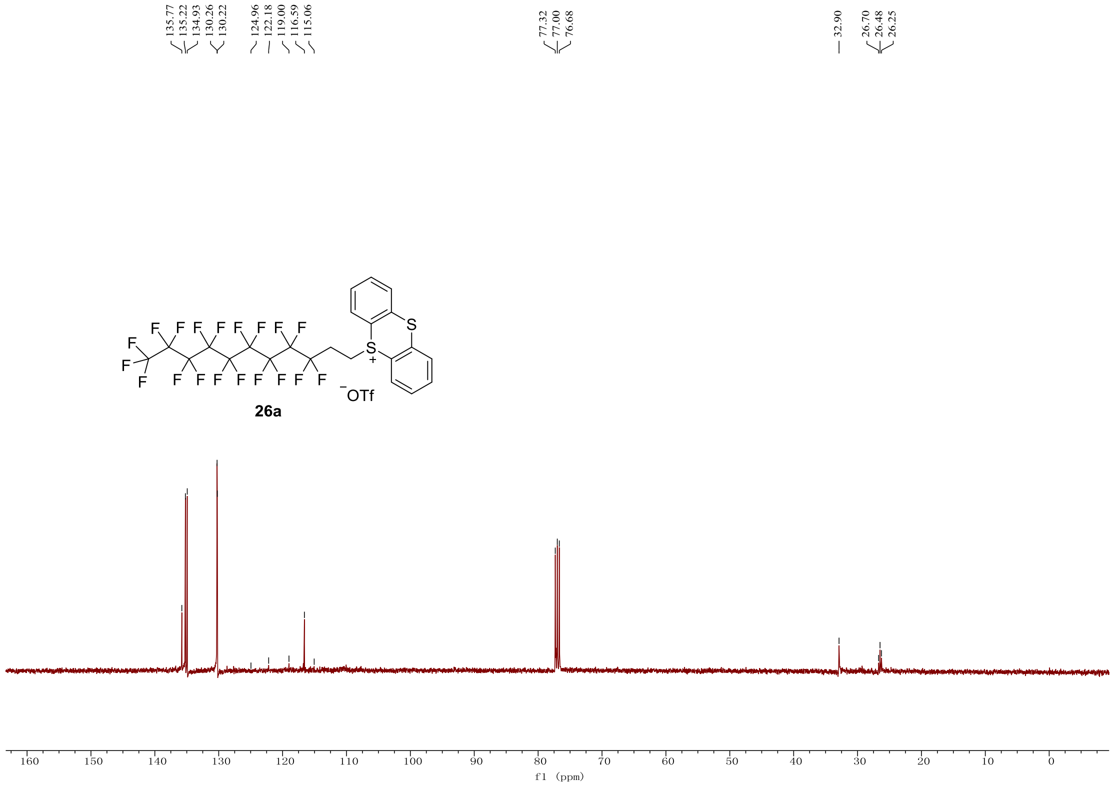

Supplementary Figures 95. ^{13}C NMR spectrum for 26a.

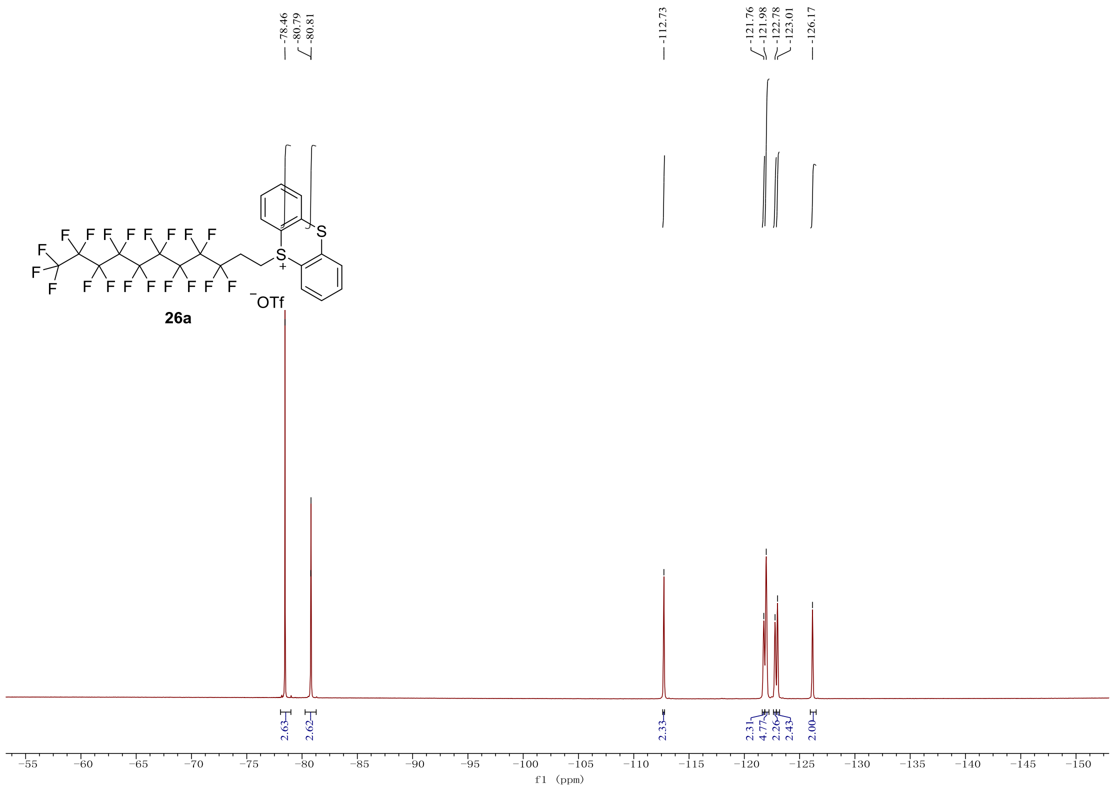

Supplementary Figure 96. ^{19}F NMR spectrum for 26a.

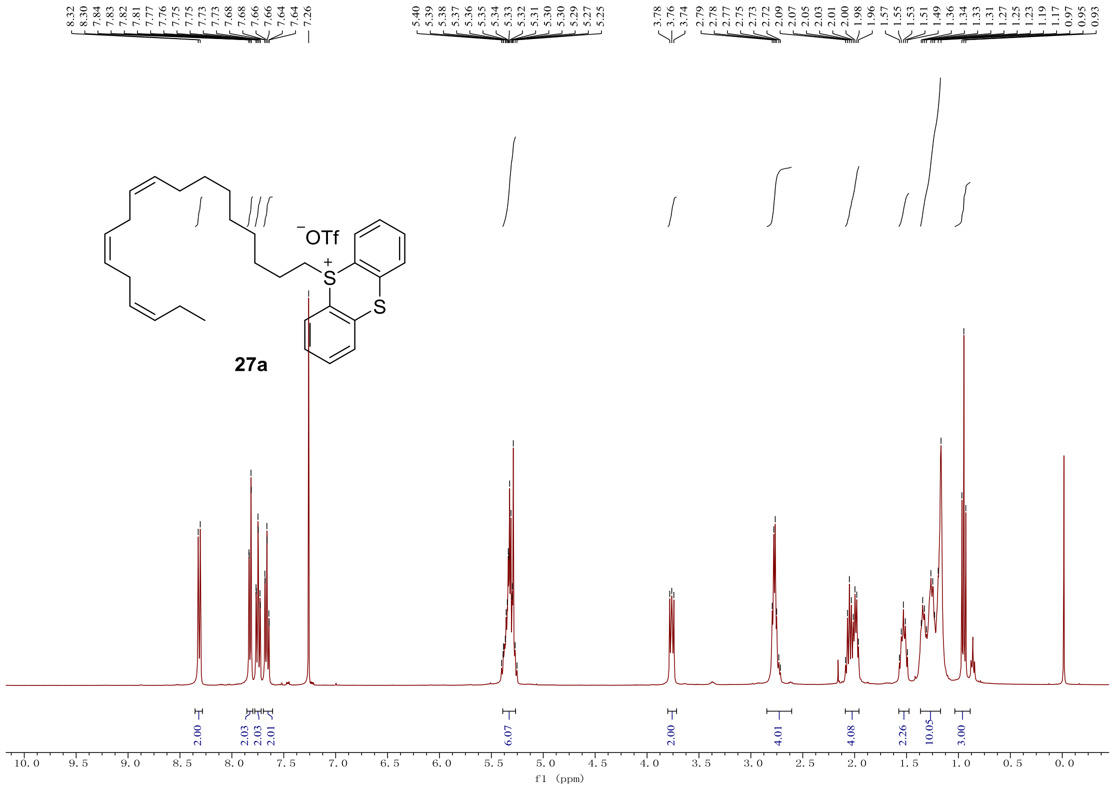

Supplementary Figure 97. ^{1}H NMR spectrum for 27a.

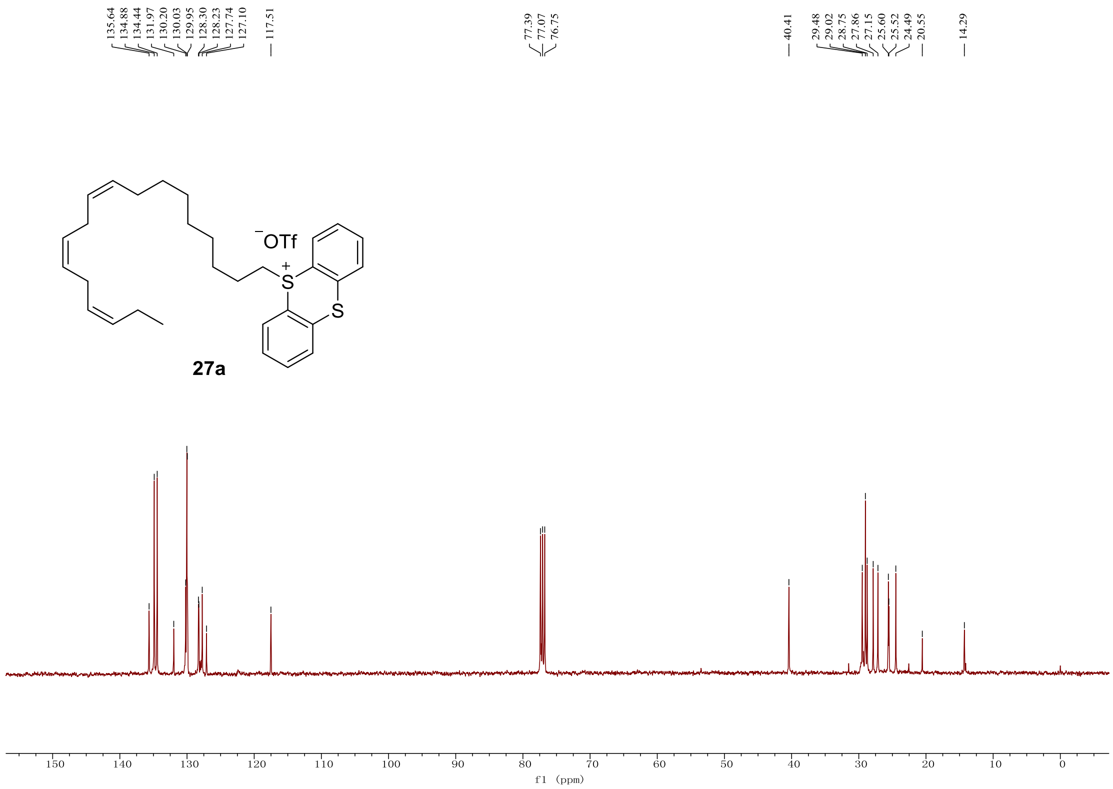

Supplementary Figures 98. ^{13}C NMR spectrum for 27a.

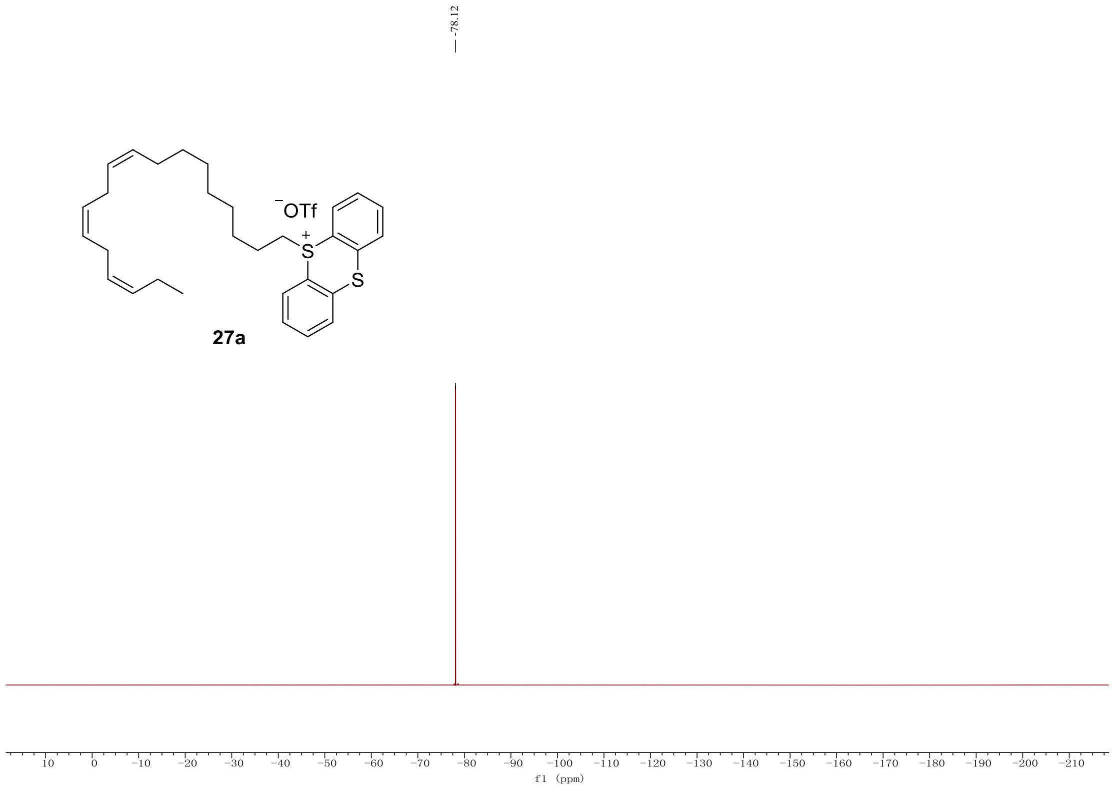

Supplementary Figure 99. ^{19}F NMR spectrum for 27a.

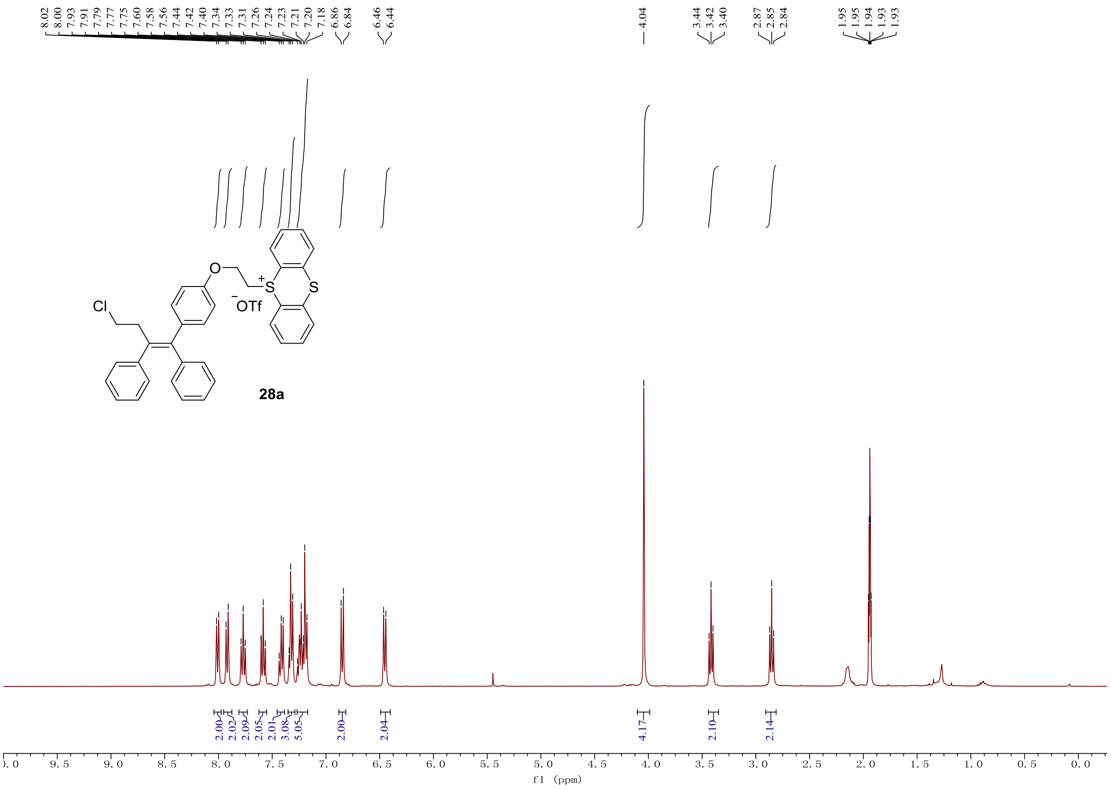

Supplementary Figure 100. ^{1}H NMR spectrum for 28a.

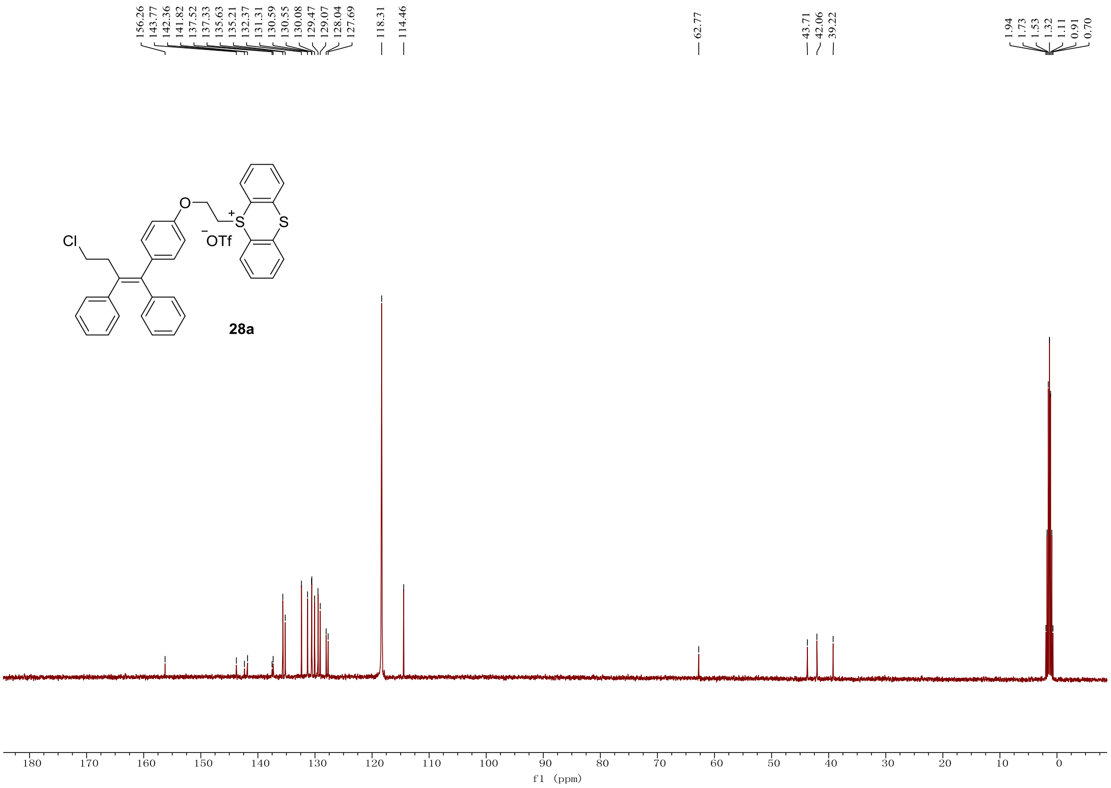

Supplementary Figures 101. ^{13}C NMR spectrum for 28a.

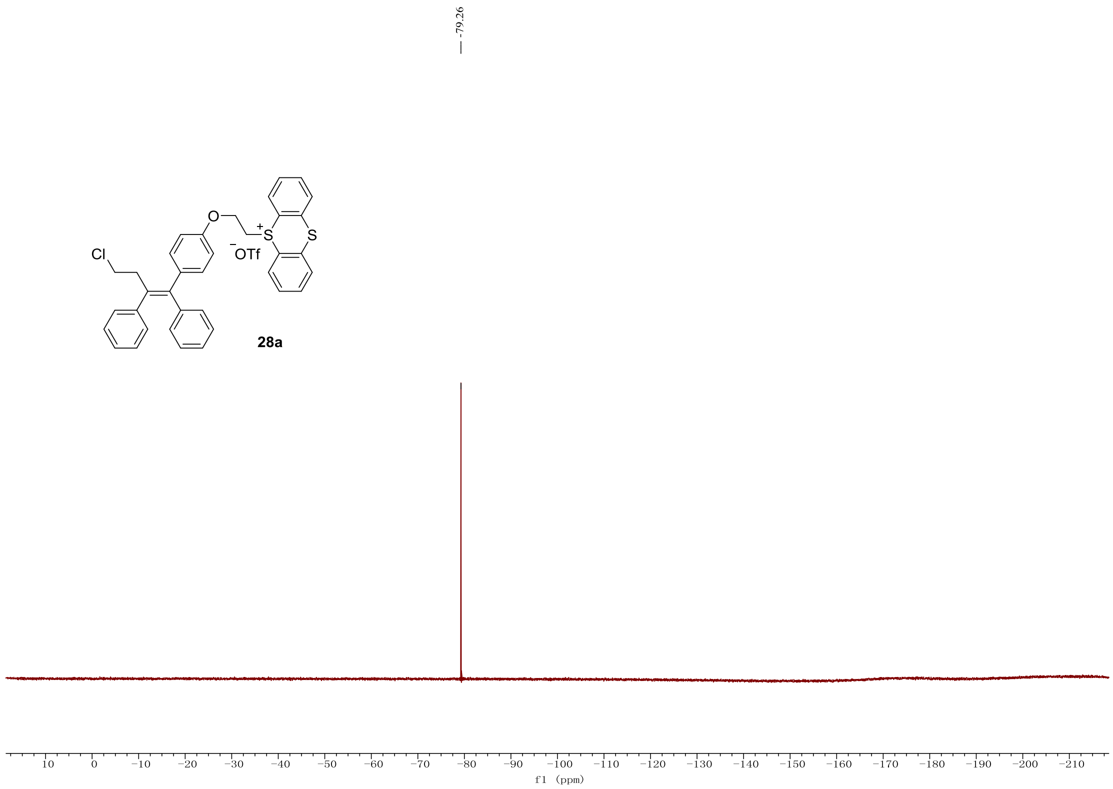

Supplementary Figure 102. ^{19}F NMR spectrum for 28a.

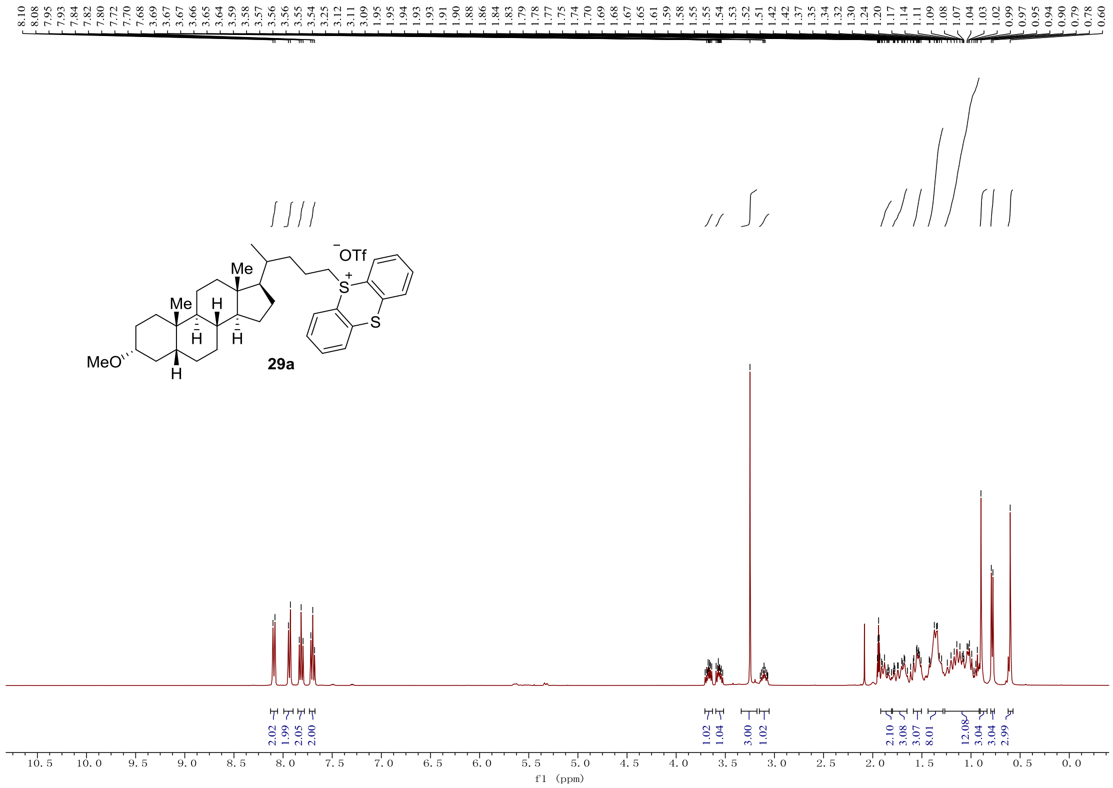

Supplementary Figure 103. ^{1}H NMR spectrum for 29a.

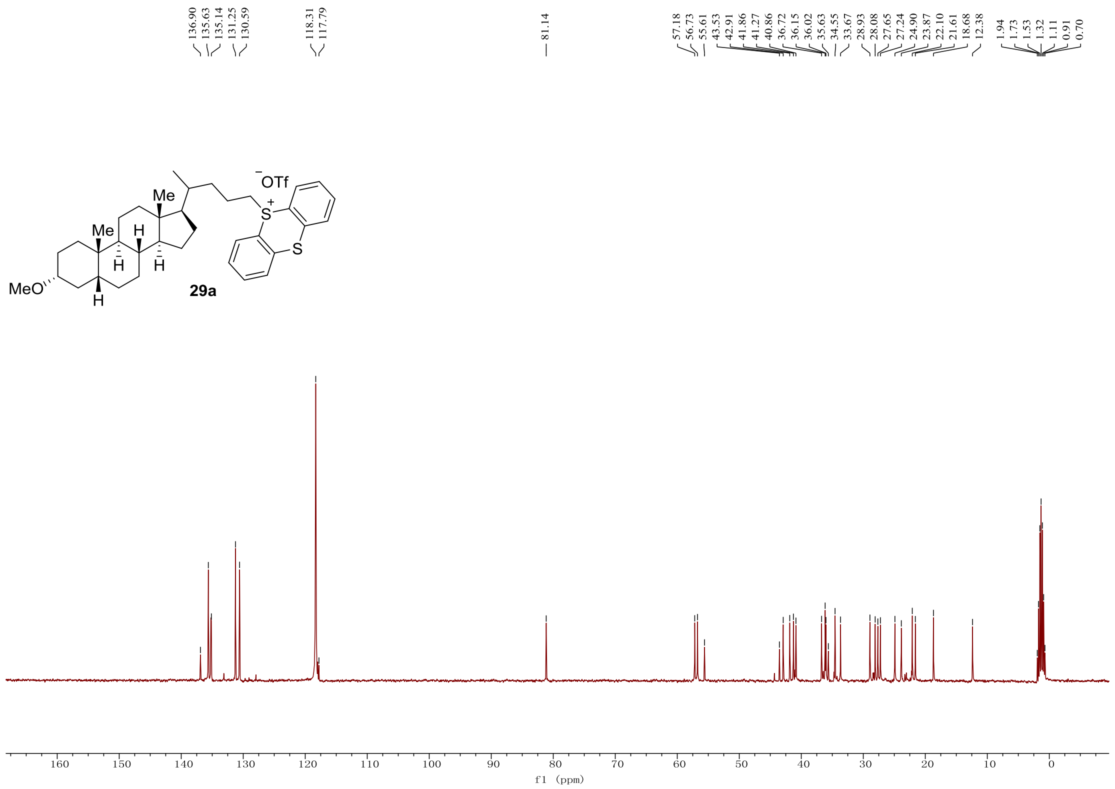

Supplementary Figures 104. ^{13}C NMR spectrum for 29a.

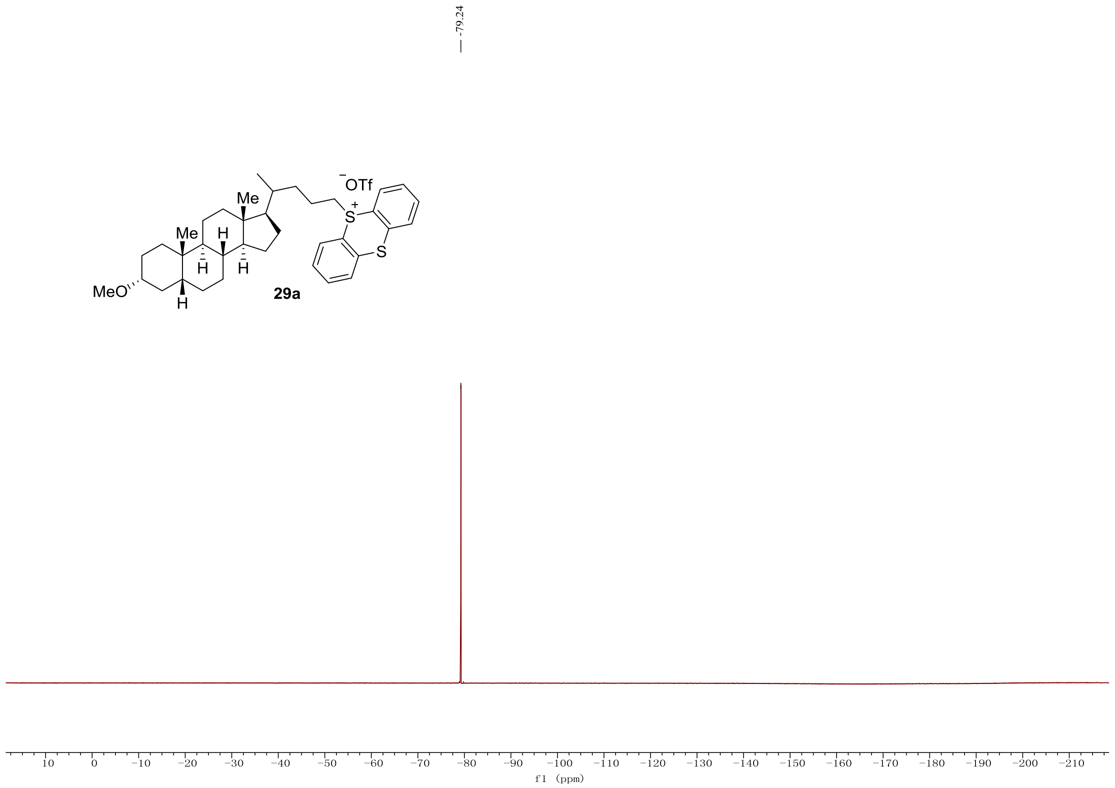

Supplementary Figure 105. ^{19}F NMR spectrum for 29a.

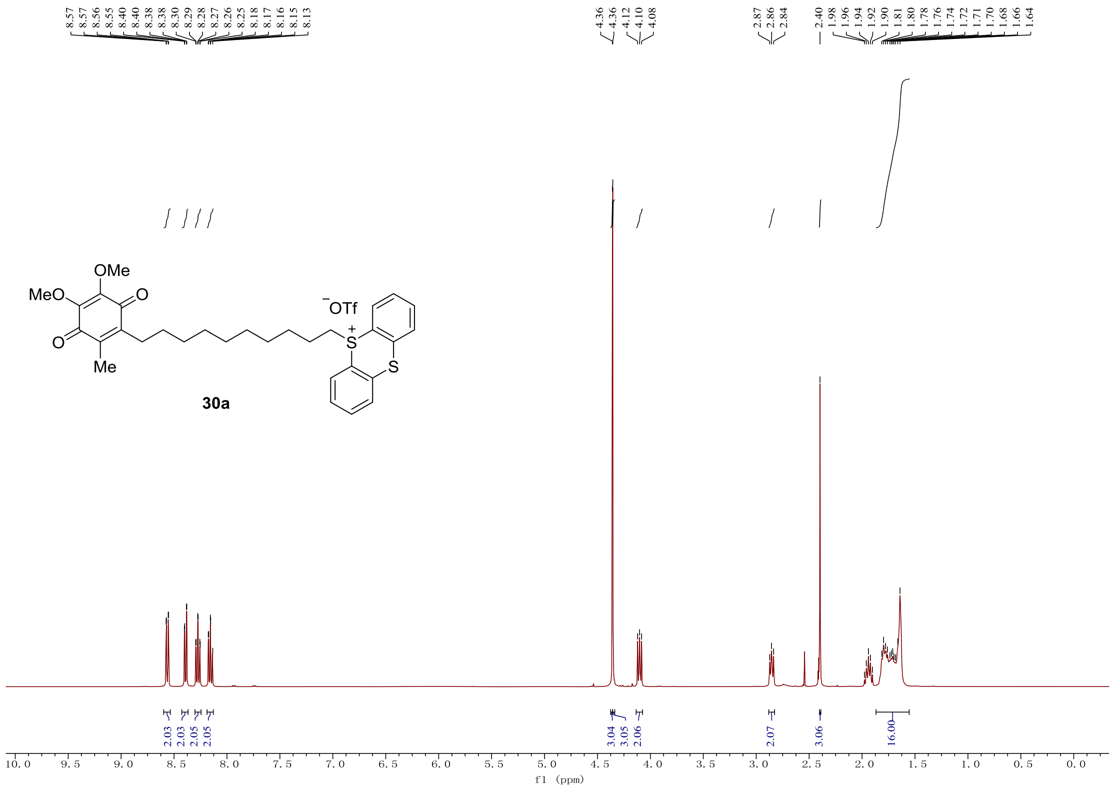

Supplementary Figure 106. ^{1}H NMR spectrum for 30a.

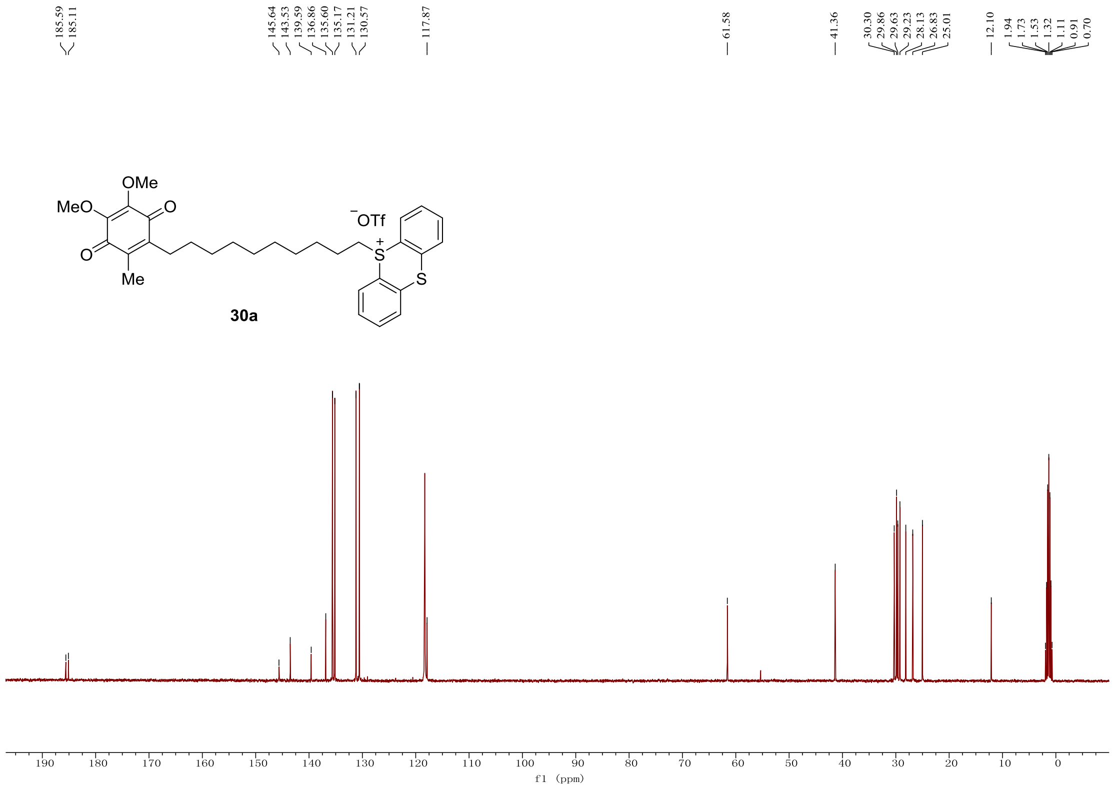

Supplementary Figures 107. ^{13}C NMR spectrum for 30a.

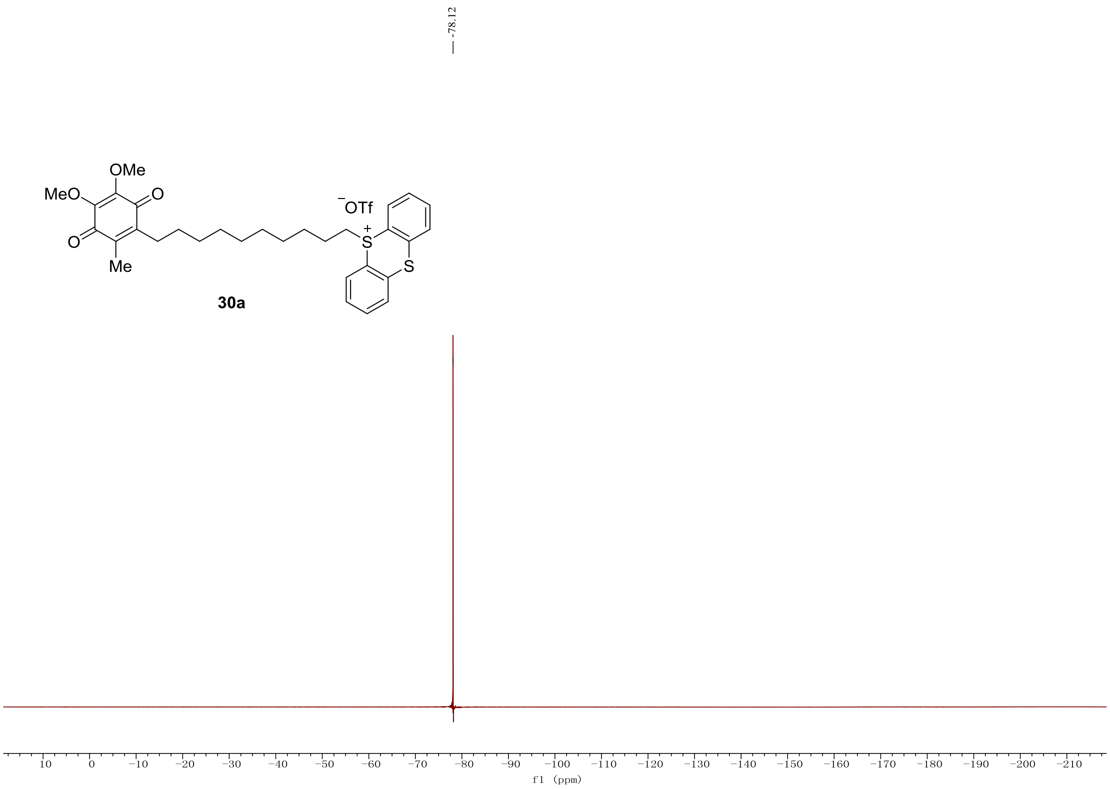

Supplementary Figure 108. ^{19}F NMR spectrum for 30a.

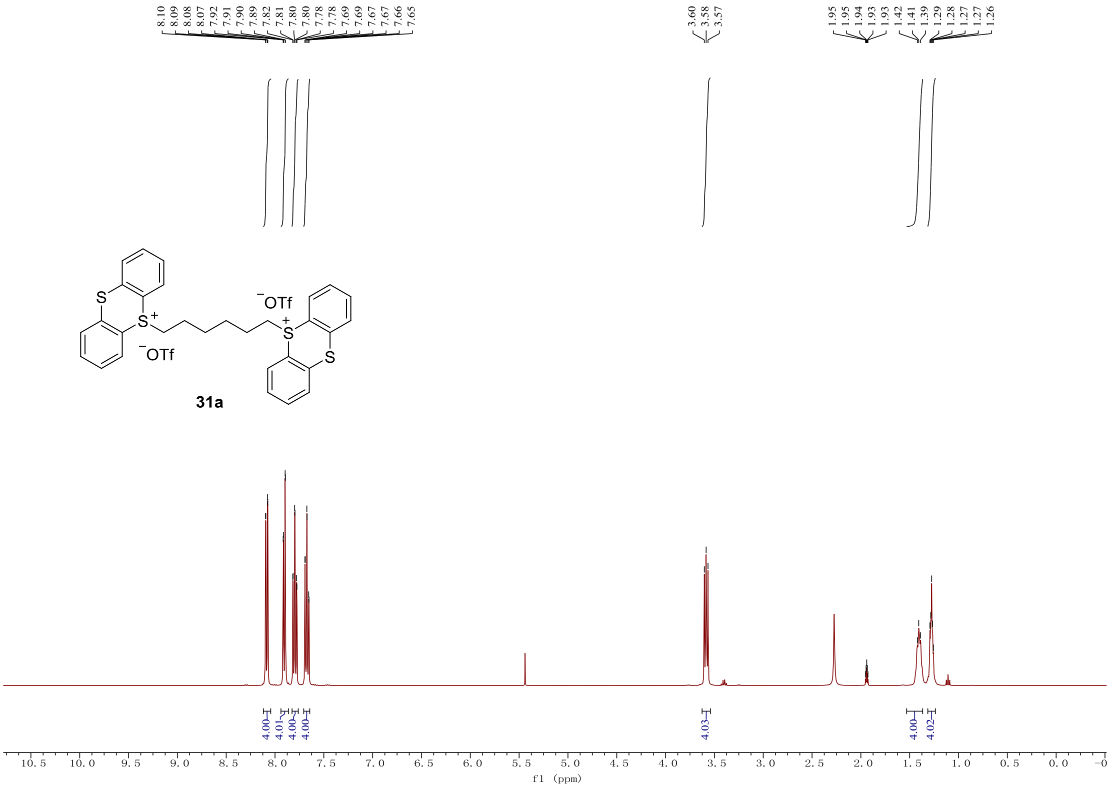

Supplementary Figure 109. ^{1}H NMR spectrum for 31a.

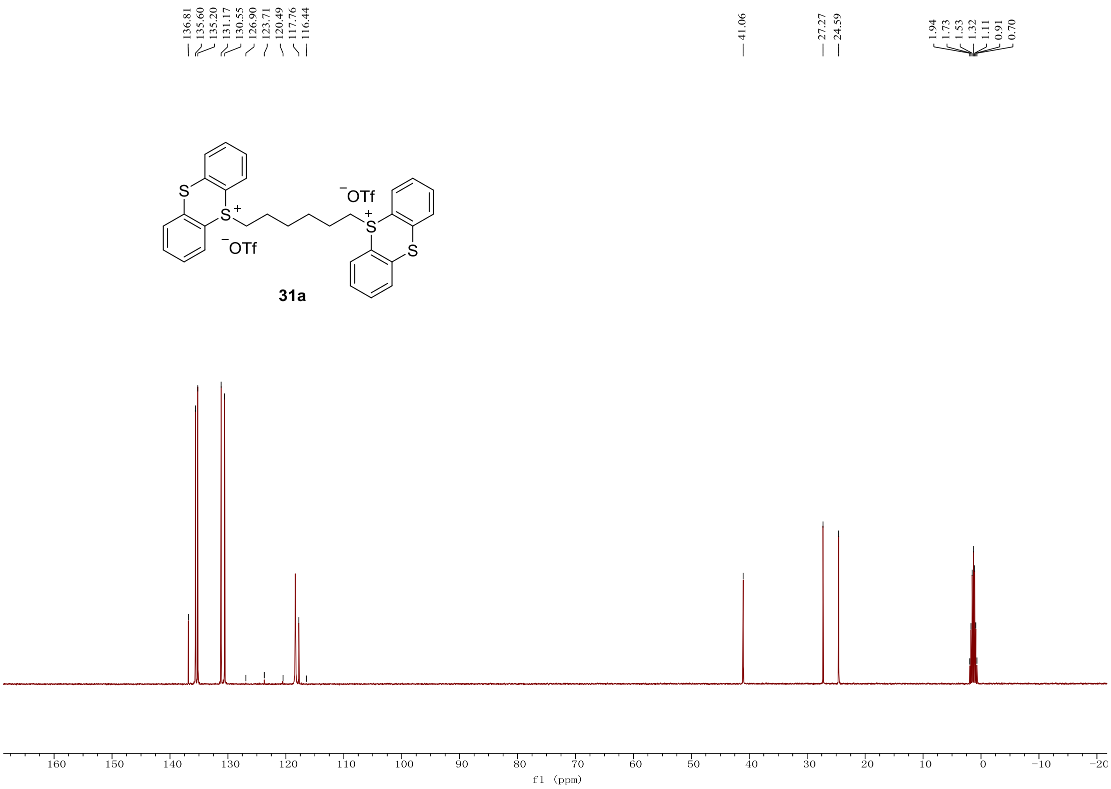

Supplementary Figures 110. ^{13}C NMR spectrum for 31a.

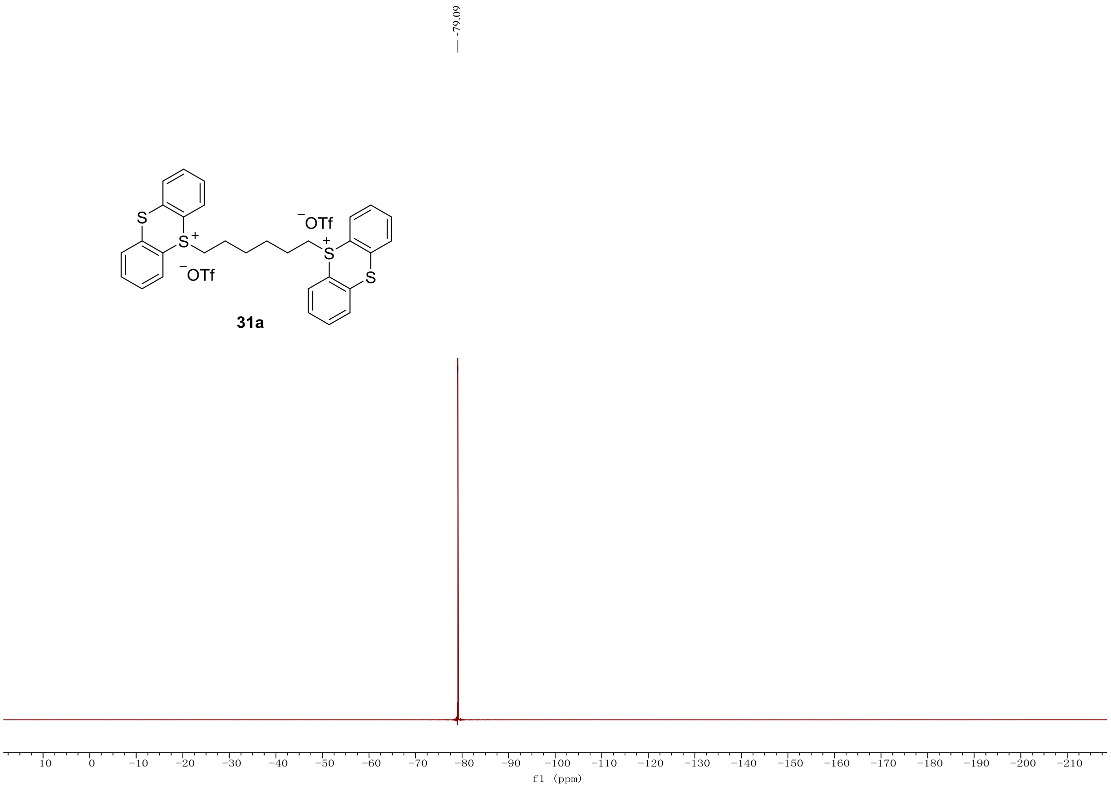

Supplementary Figure 111. ^{19}F NMR spectrum for 31a.

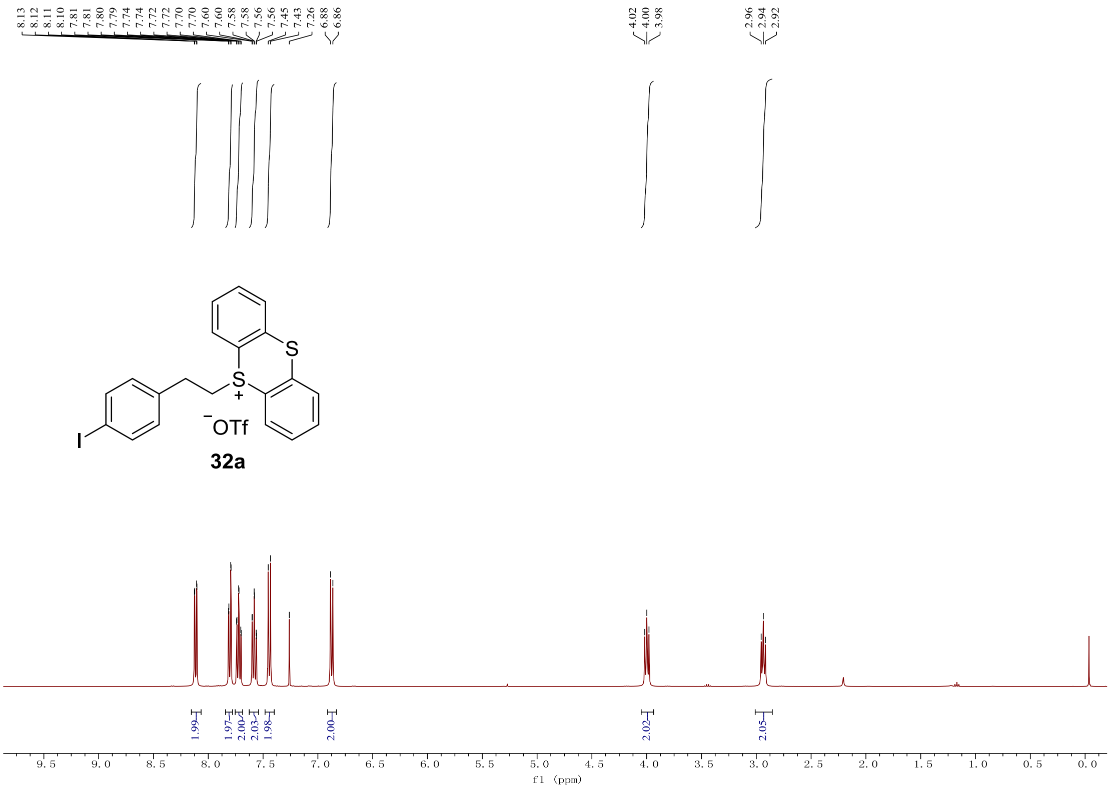

Supplementary Figure 112. ^{1}H NMR spectrum for 32a.

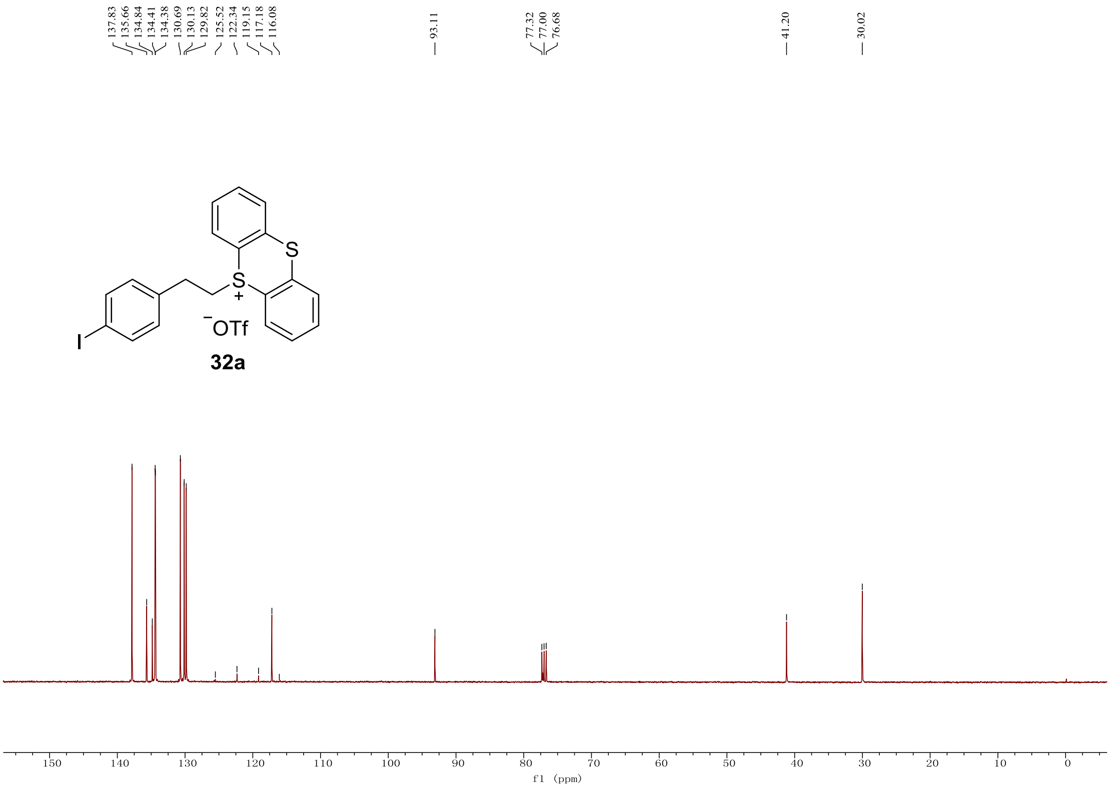

Supplementary Figures 113. ^{13}C NMR spectrum for 32a.

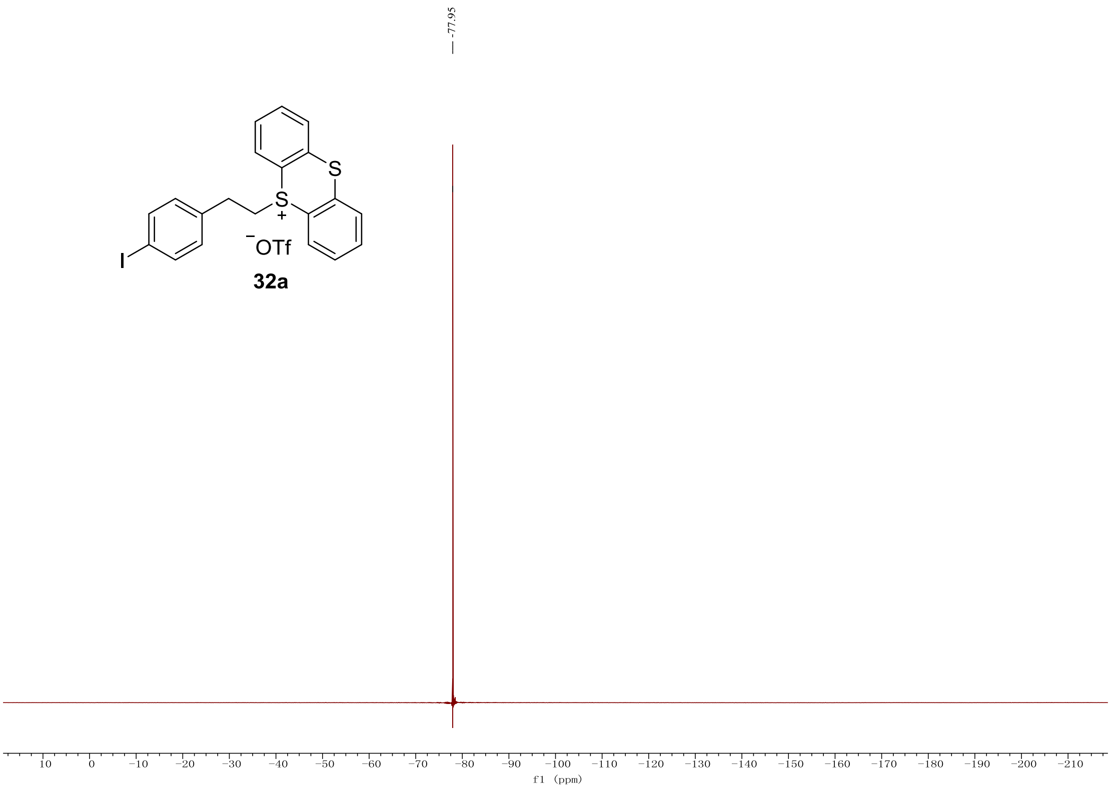

Supplementary Figure 114. ^{19}**F NMR spectrum for 32a.**

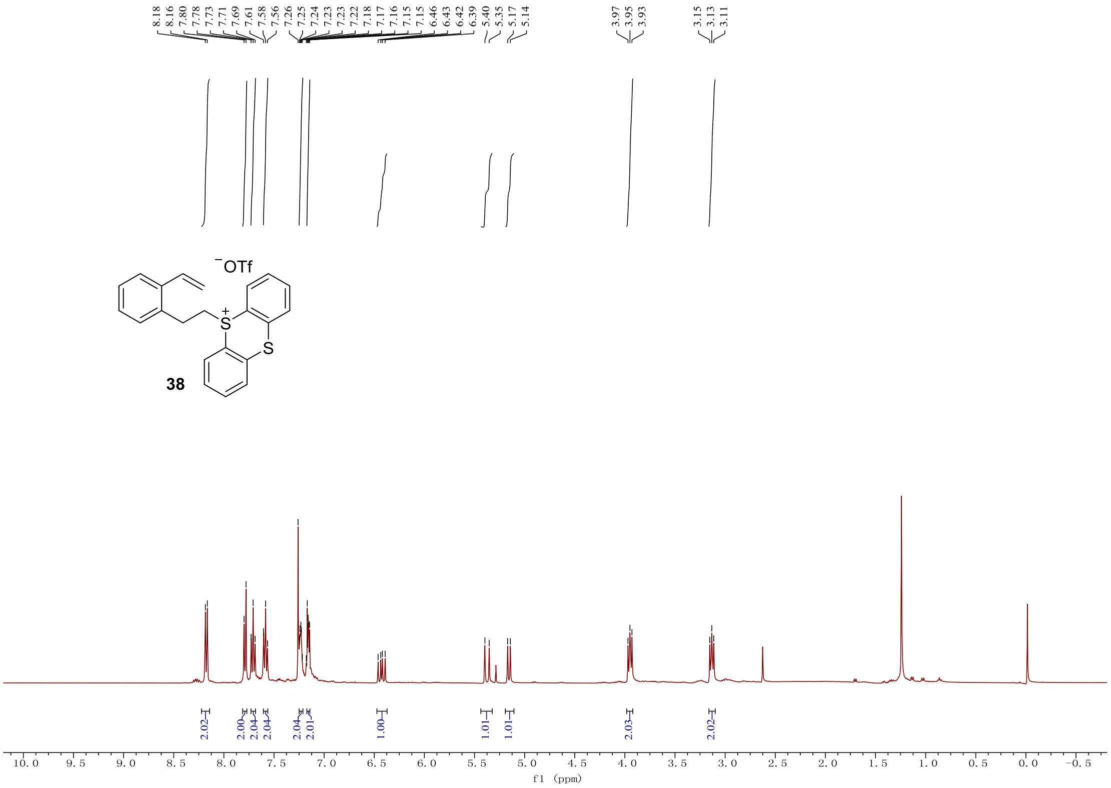

Supplementary Figure 115. ^{1}**H NMR spectrum for 38.**

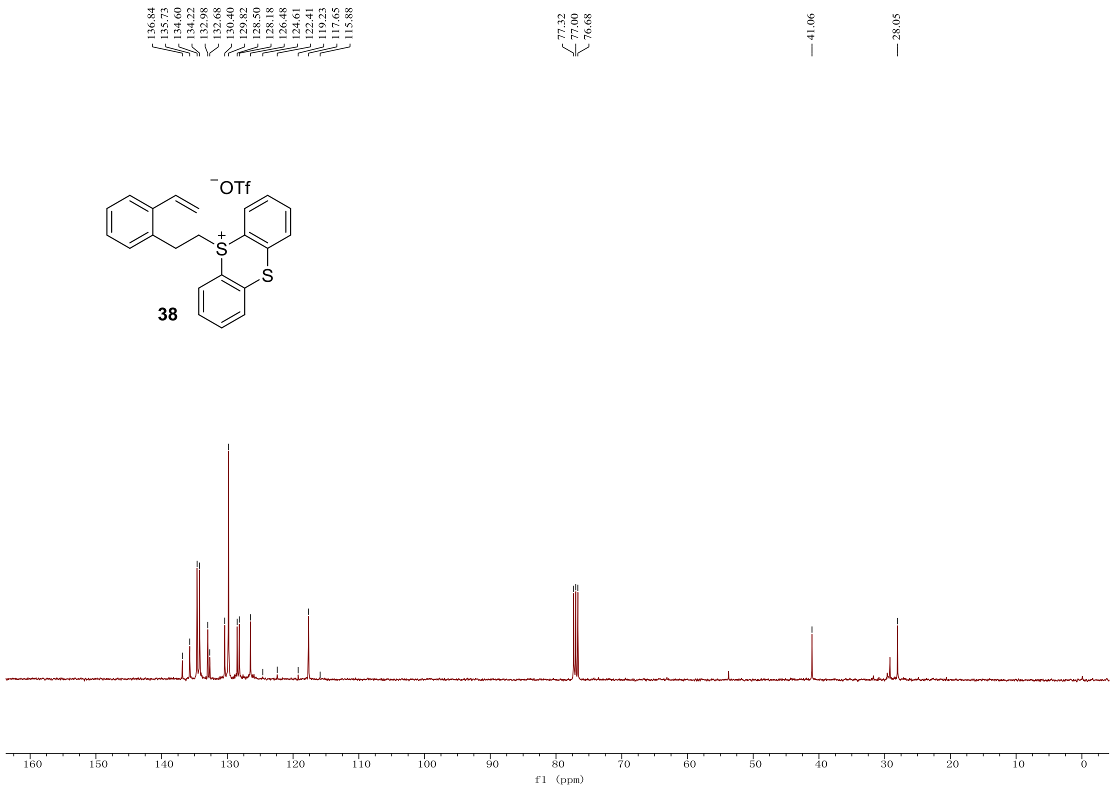

Supplementary Figures 116. ^{13}C NMR spectrum for 38.

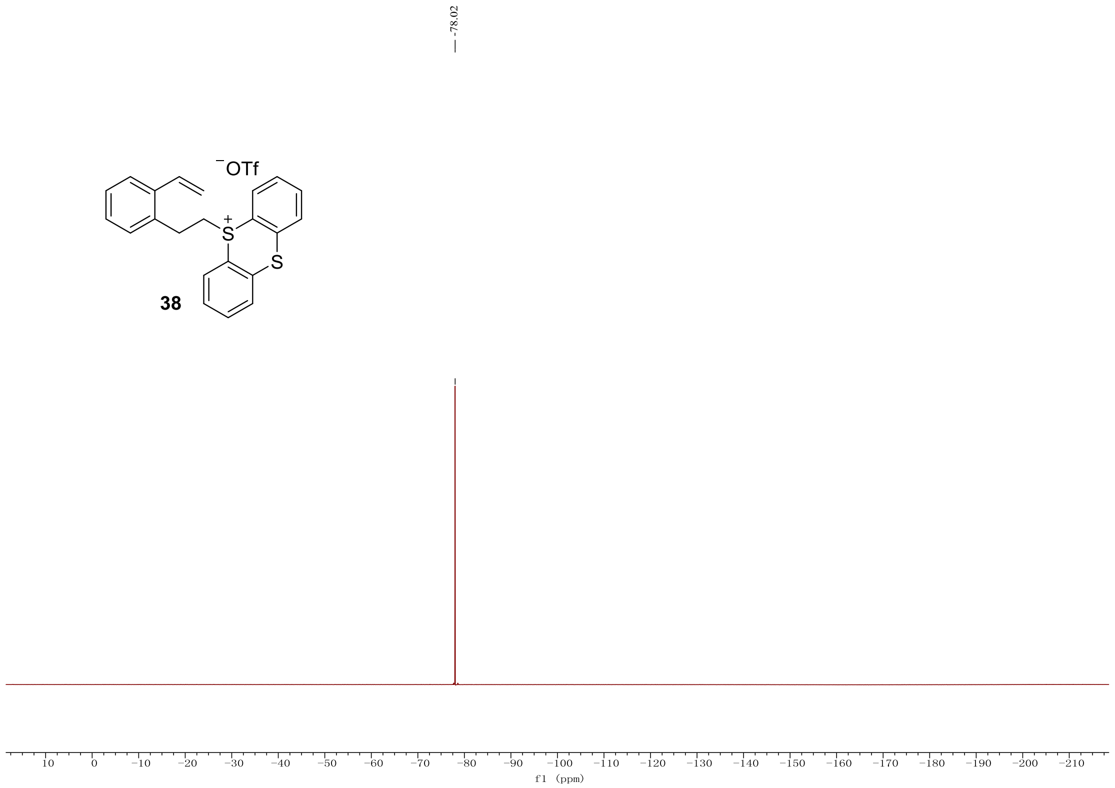

Supplementary Figure 117. ^{19}F NMR spectrum for 38.

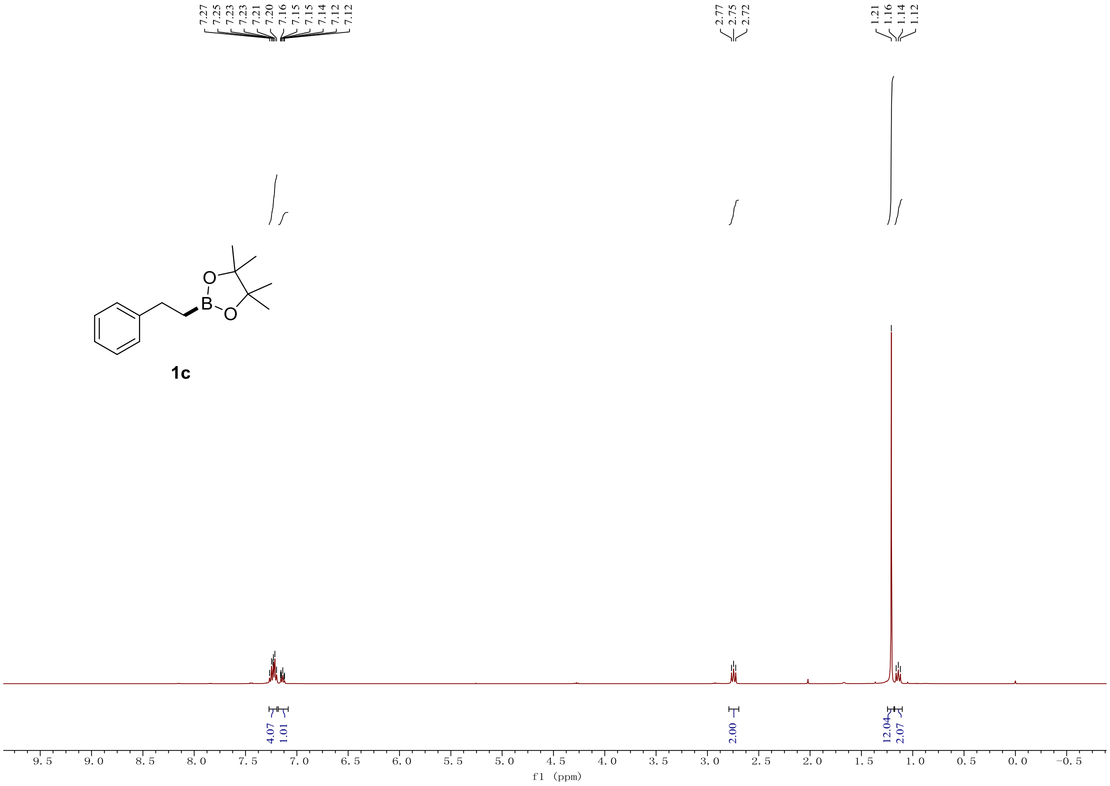

Supplementary Figure 118. ^{1}H NMR spectrum for 1c.

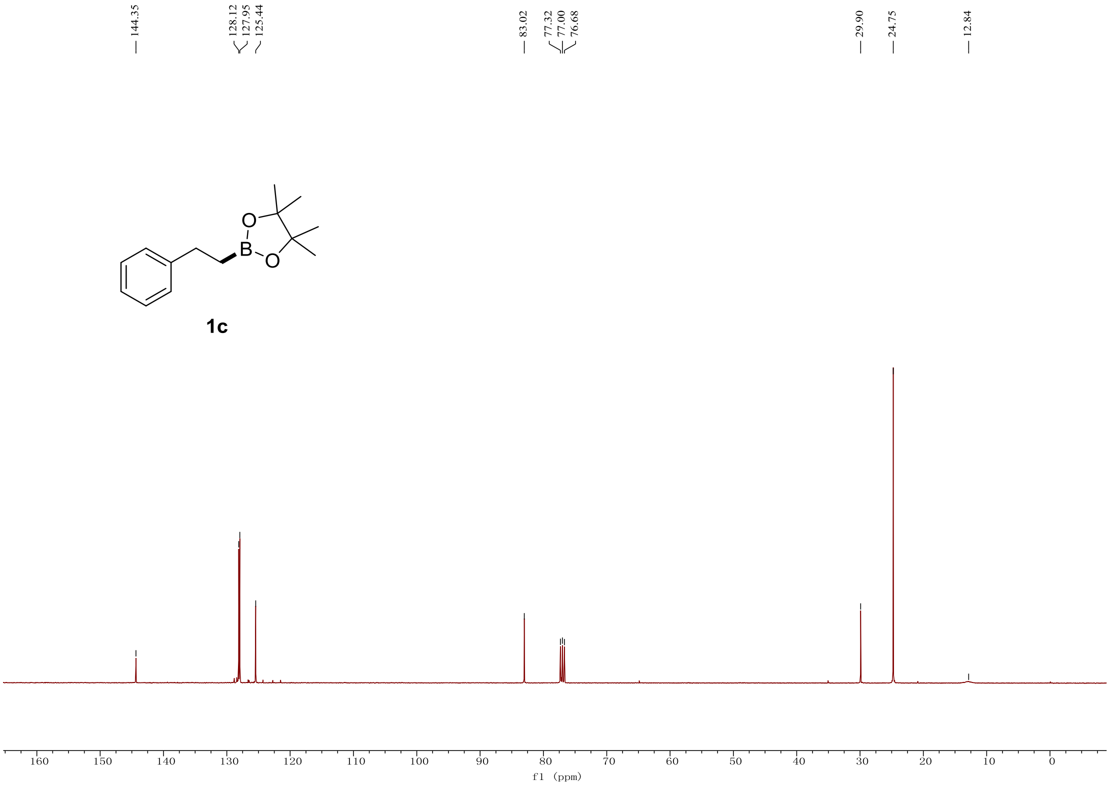

Supplementary Figures 119. ^{13}C NMR spectrum for 1c.

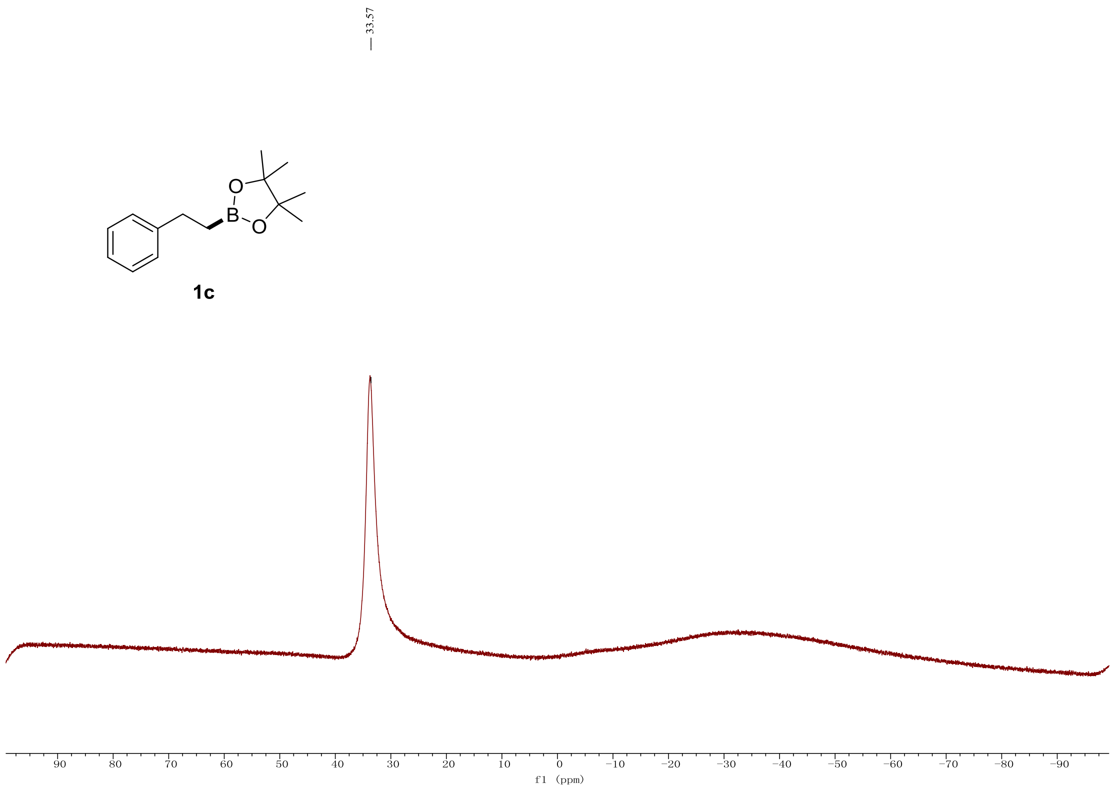

Supplementary Figure 120. ^{11}B NMR spectrum for 1c.

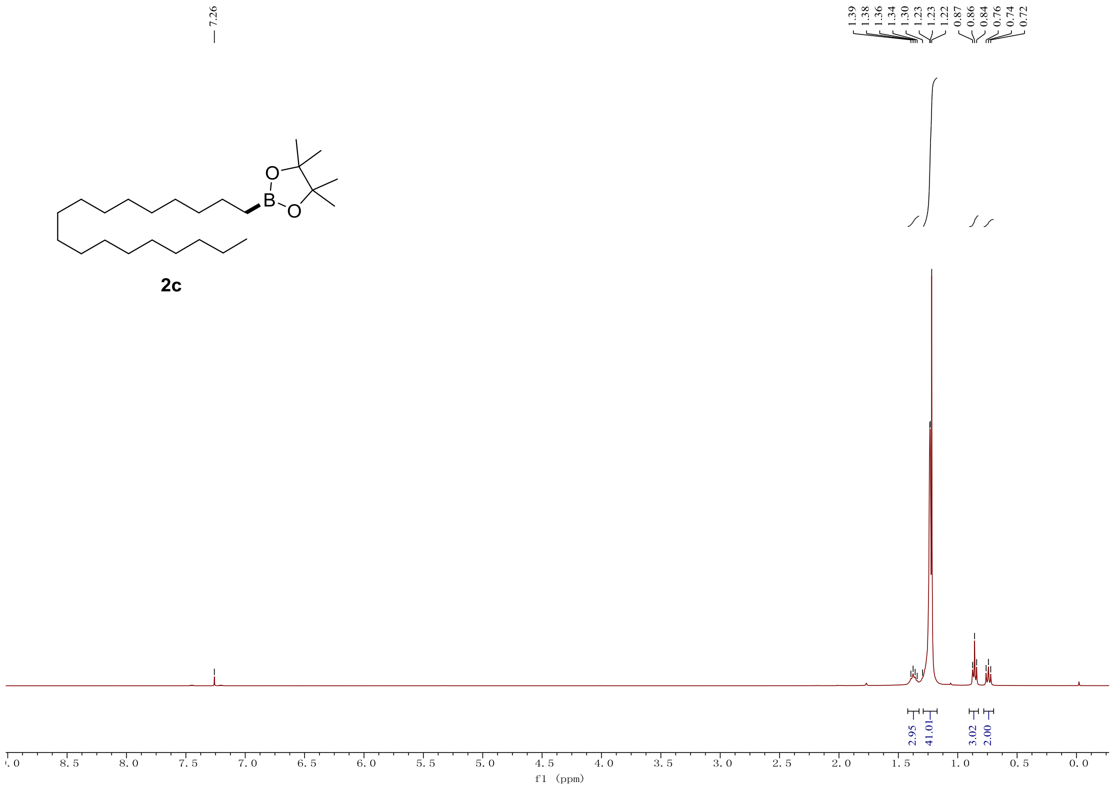

Supplementary Figure 121. ^{1}H NMR spectrum for 2c.

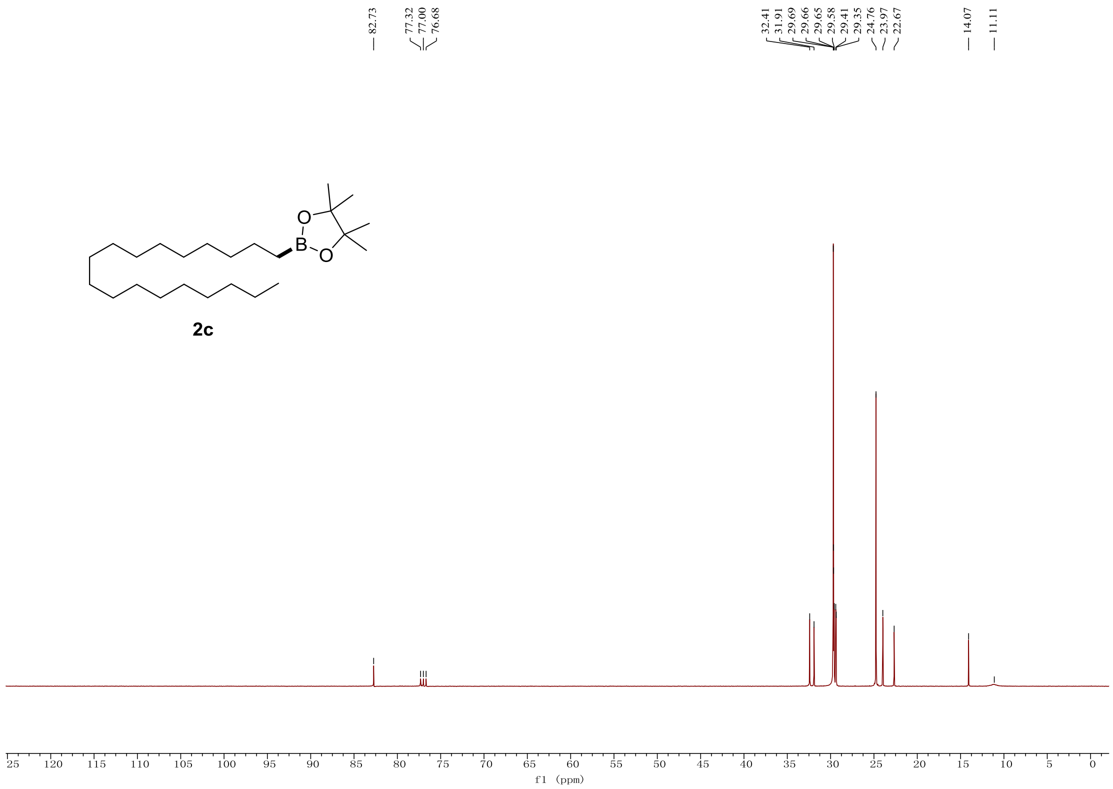

Supplementary Figures 122. ^{13}**C NMR spectrum for 2c.**

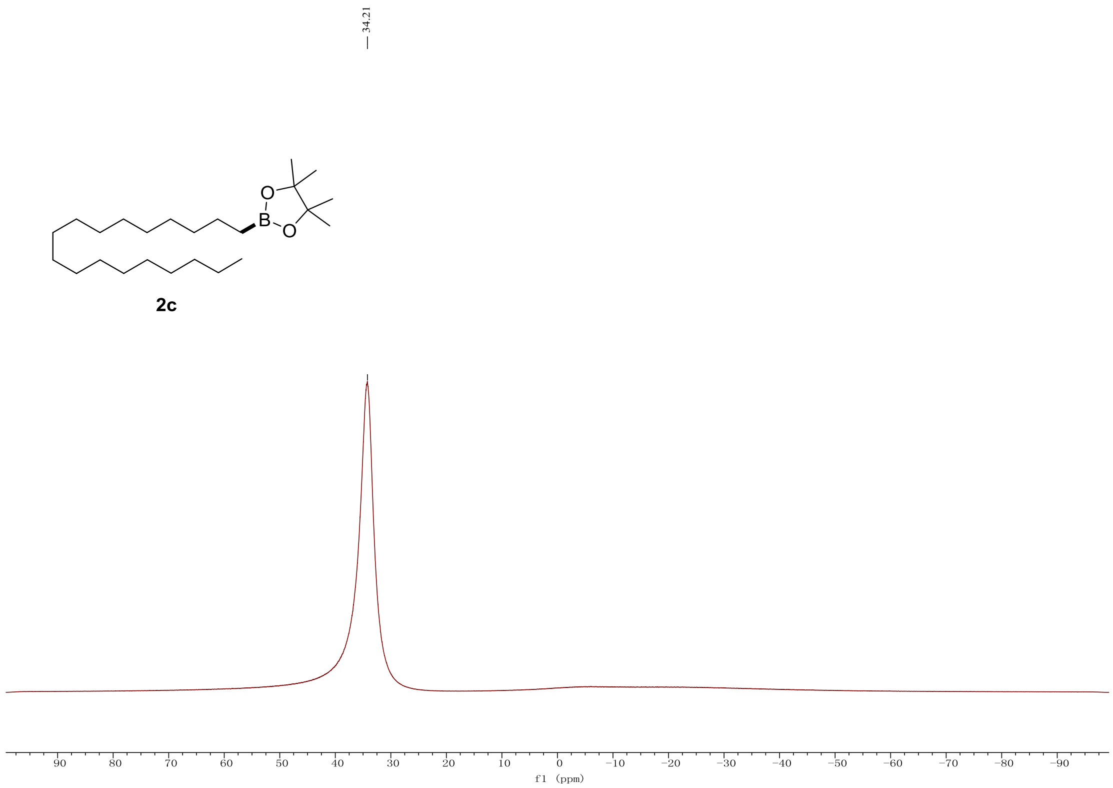

Supplementary Figure 123. ^{11}**B NMR spectrum for 2c.**

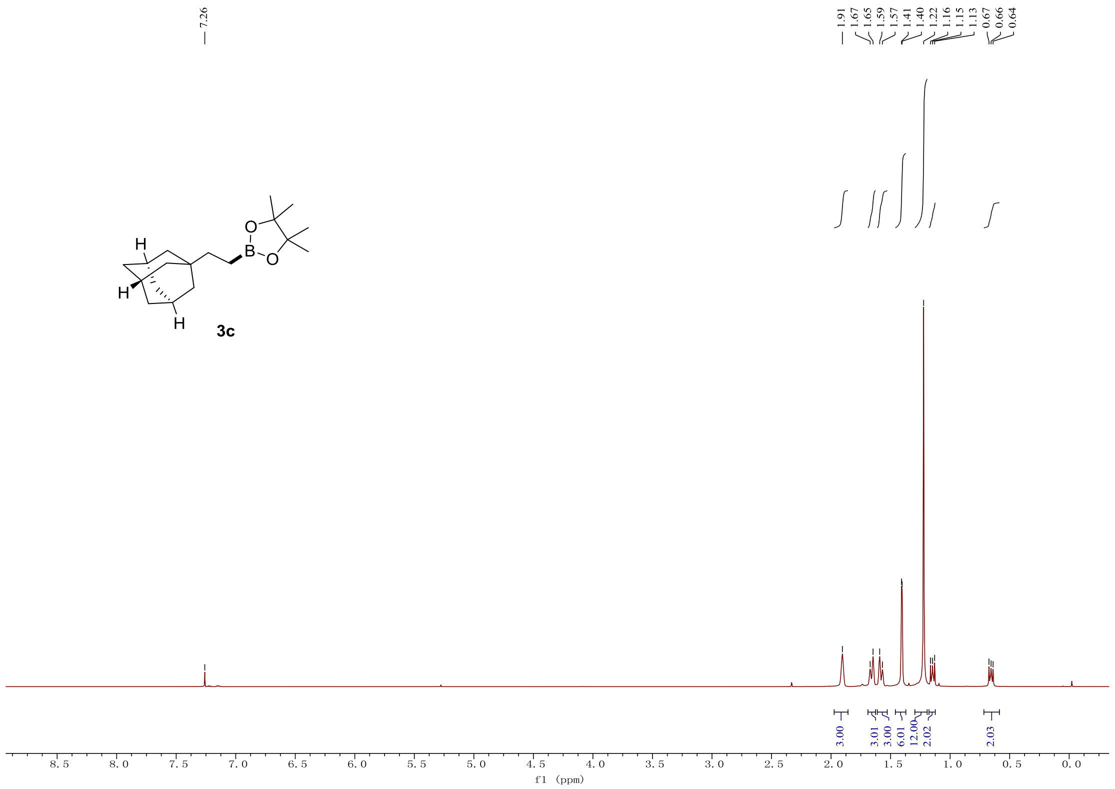

Supplementary Figure 124. ^{1}H NMR spectrum for 3c.

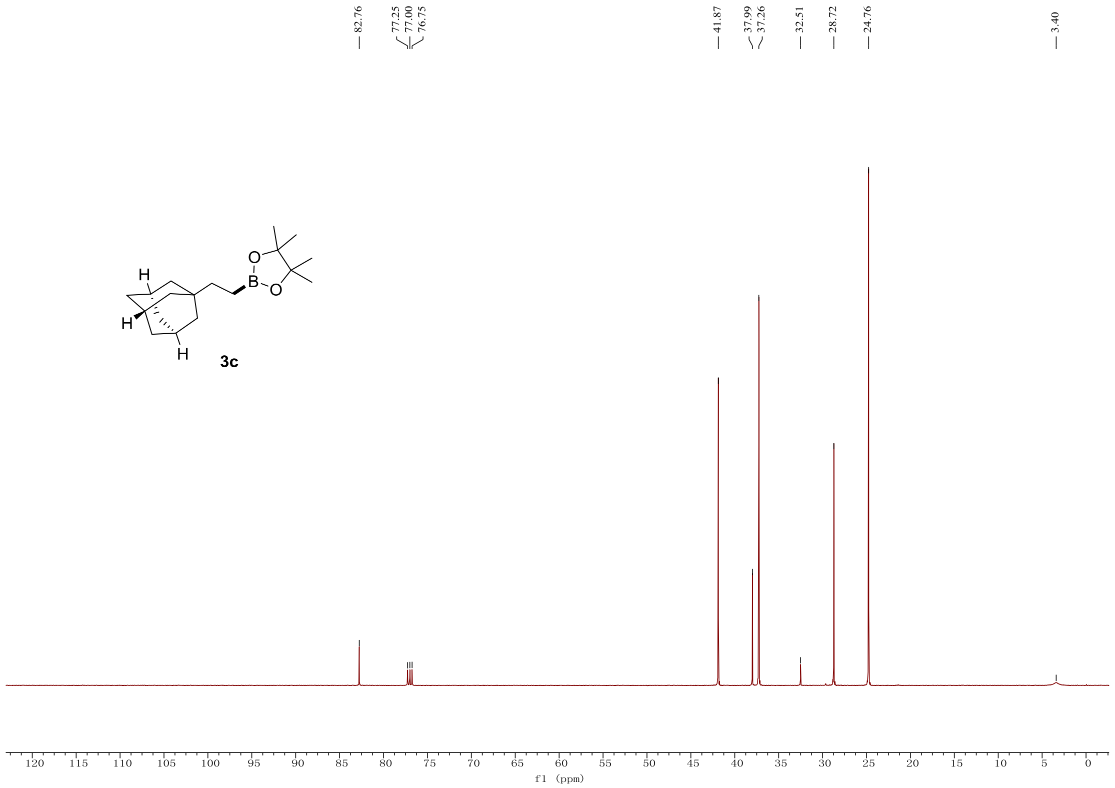

Supplementary Figures 125. ^{13}C NMR spectrum for 3c.

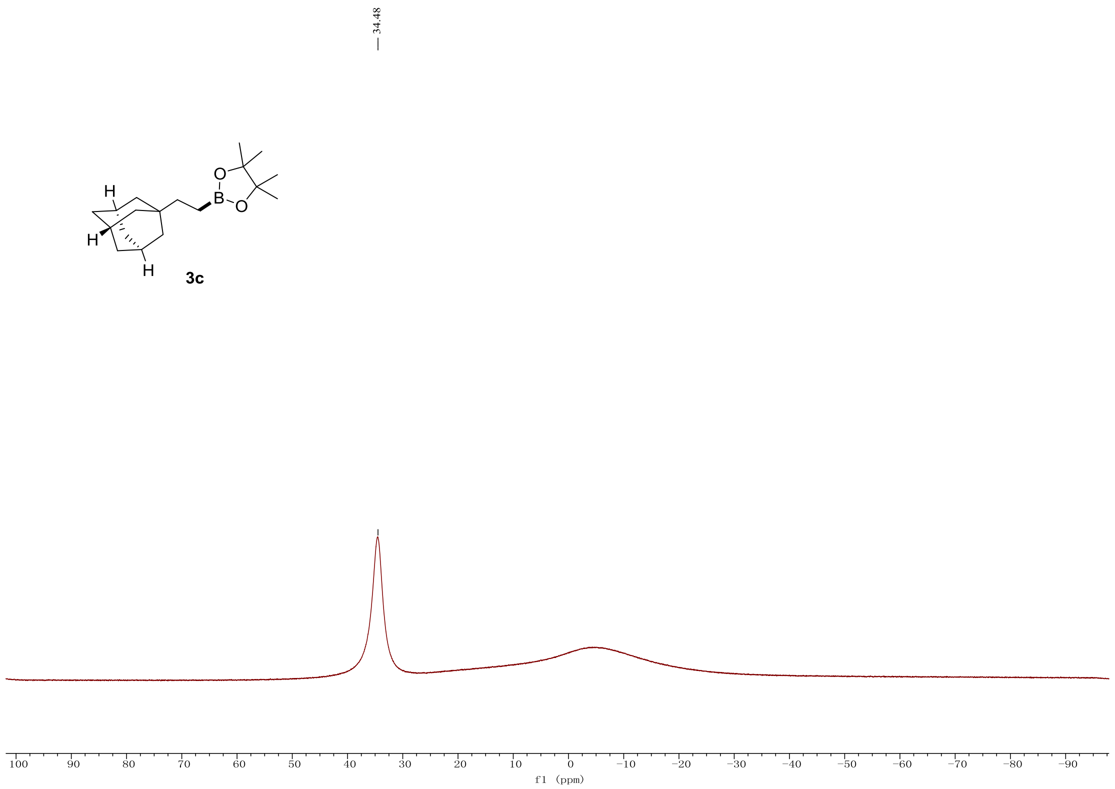

Supplementary Figure 126. ^{11}B NMR spectrum for 3c.

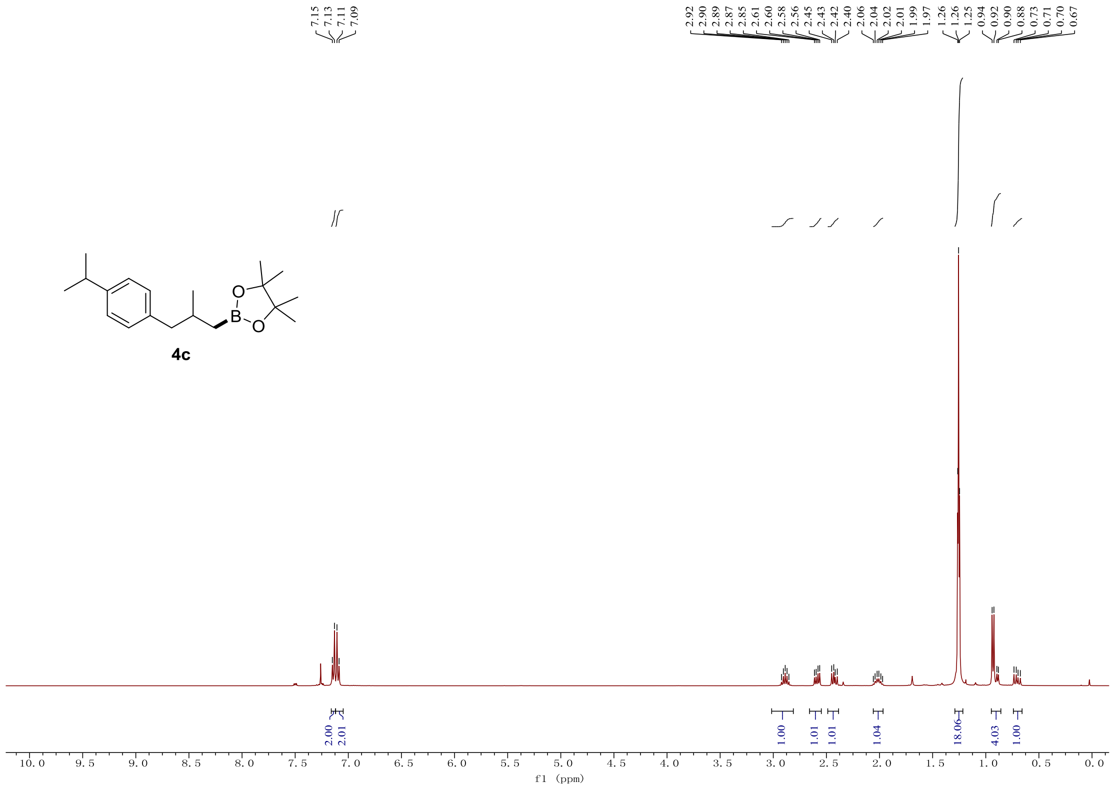

Supplementary Figure 127. ^{1}H NMR spectrum for 4c.

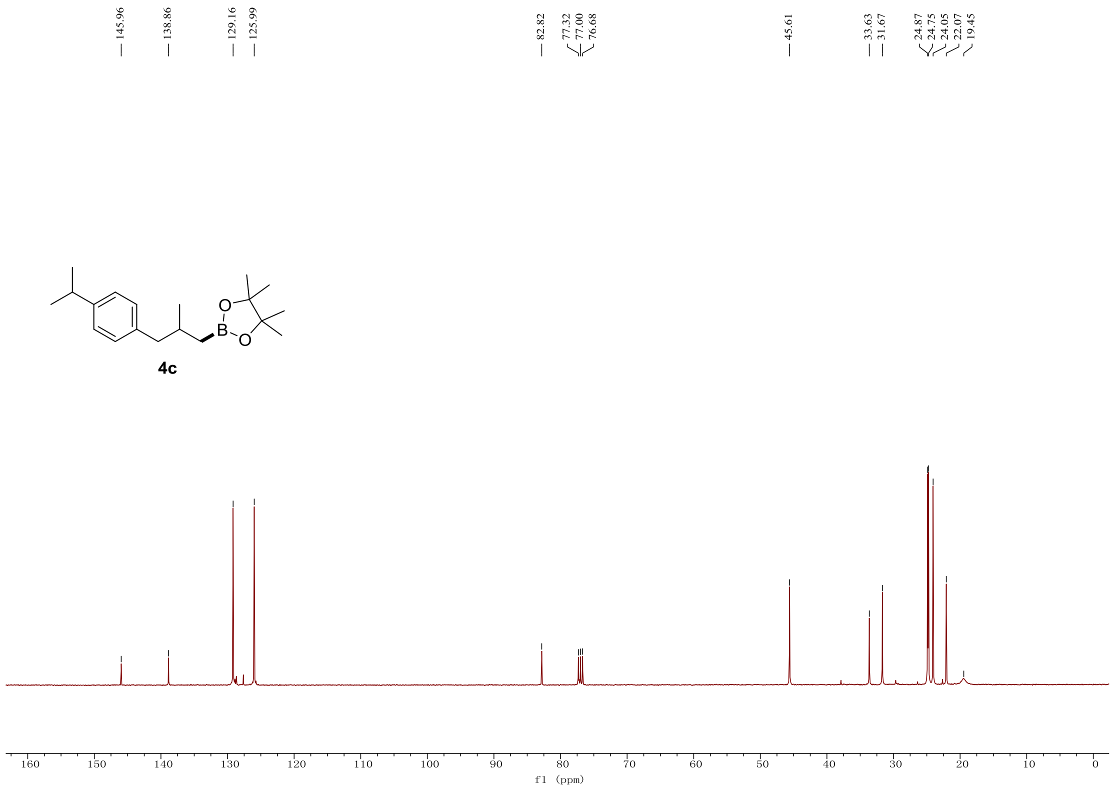

Supplementary Figures 128. ^{13}**C NMR spectrum for 4c.**

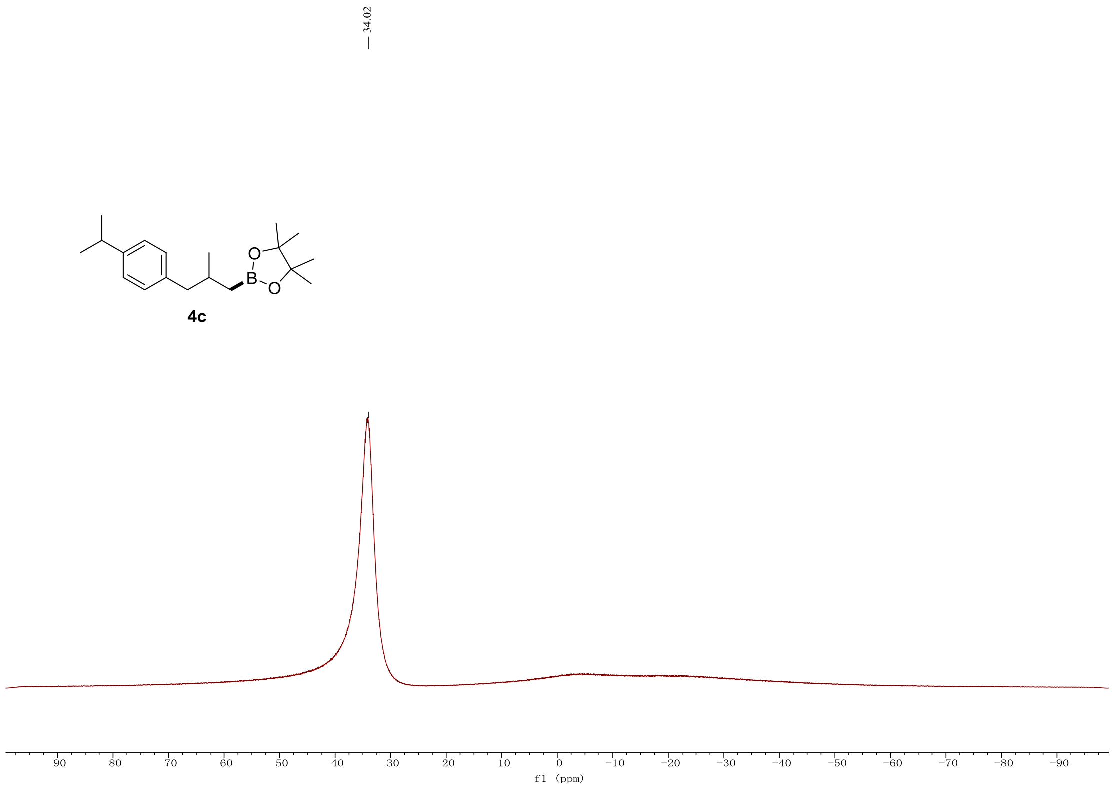

Supplementary Figure 129. ^{11}**B NMR spectrum for 4c.**

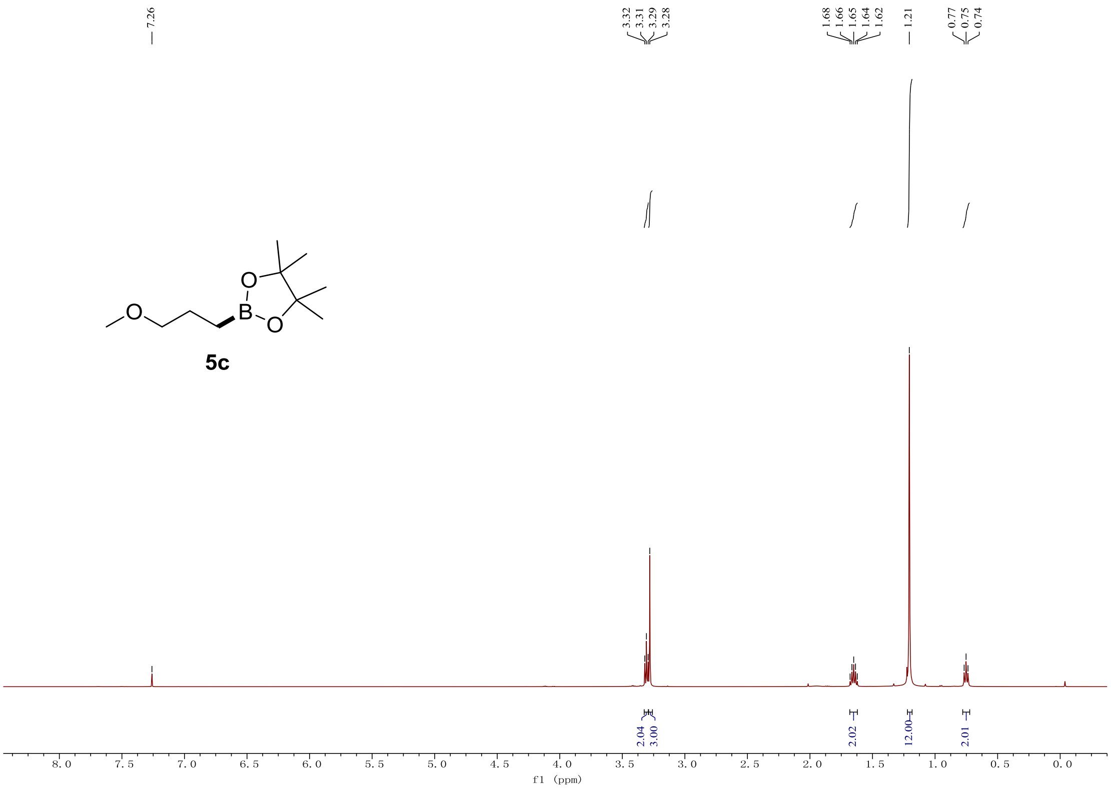

Supplementary Figure 130. ^{1}**H NMR spectrum for 5c.**

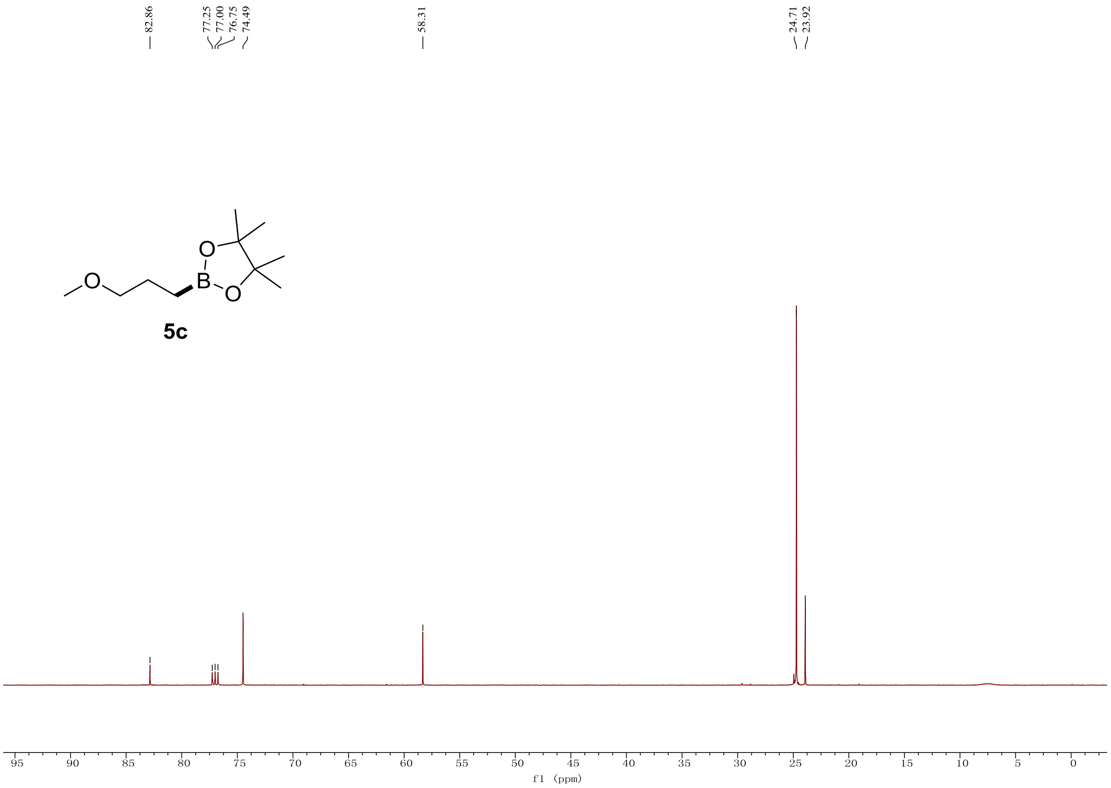

Supplementary Figures 131. ^{13}**C NMR spectrum for 5c.**

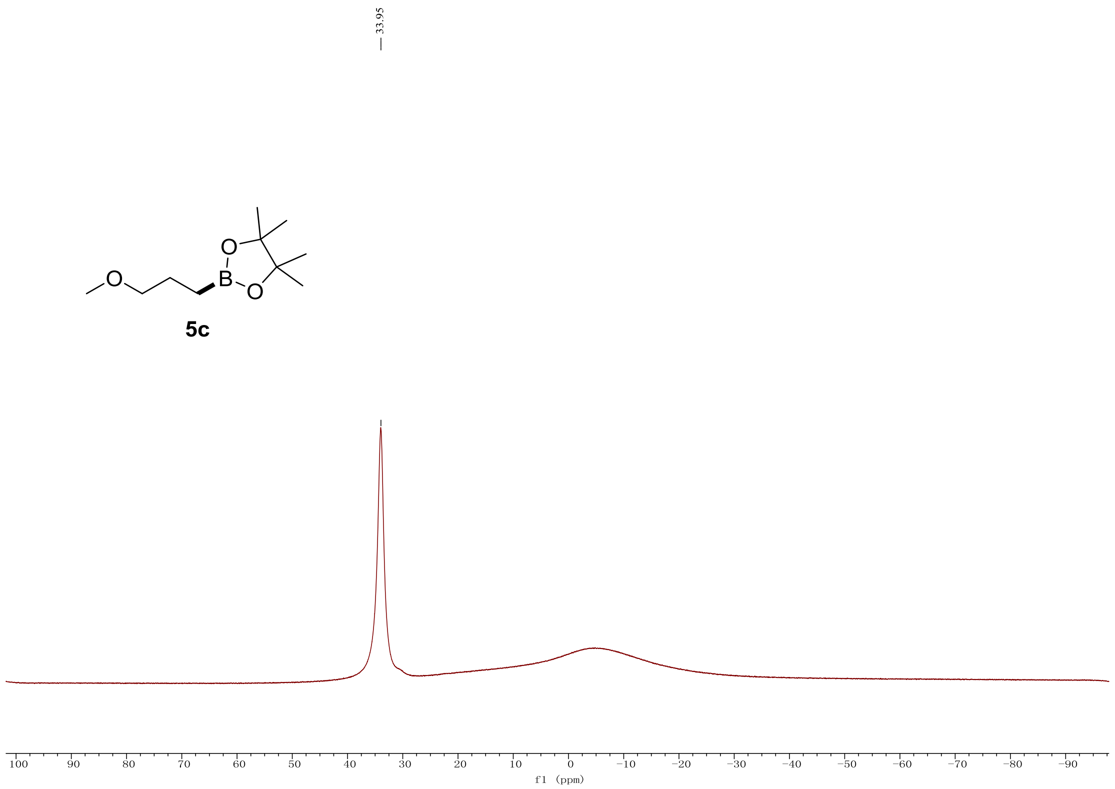

Supplementary Figure 132. ^{11}B NMR spectrum for 5c.

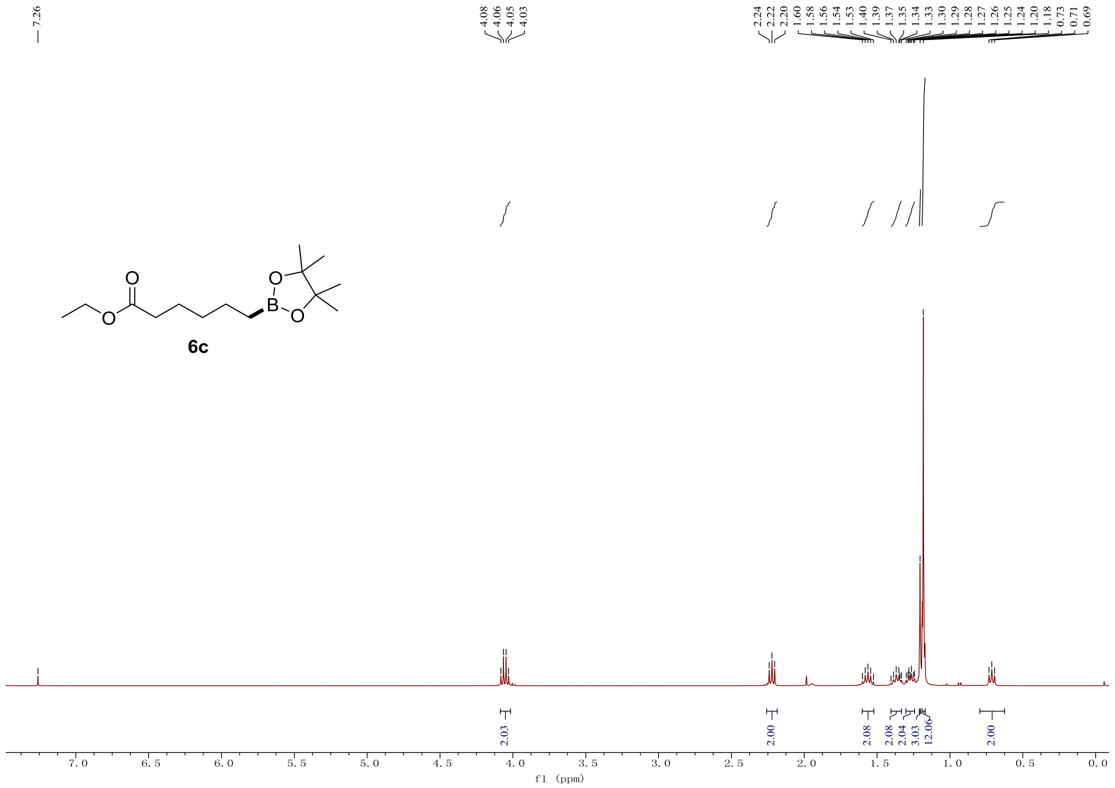

Supplementary Figure 133. ^{1}H NMR spectrum for 6c.

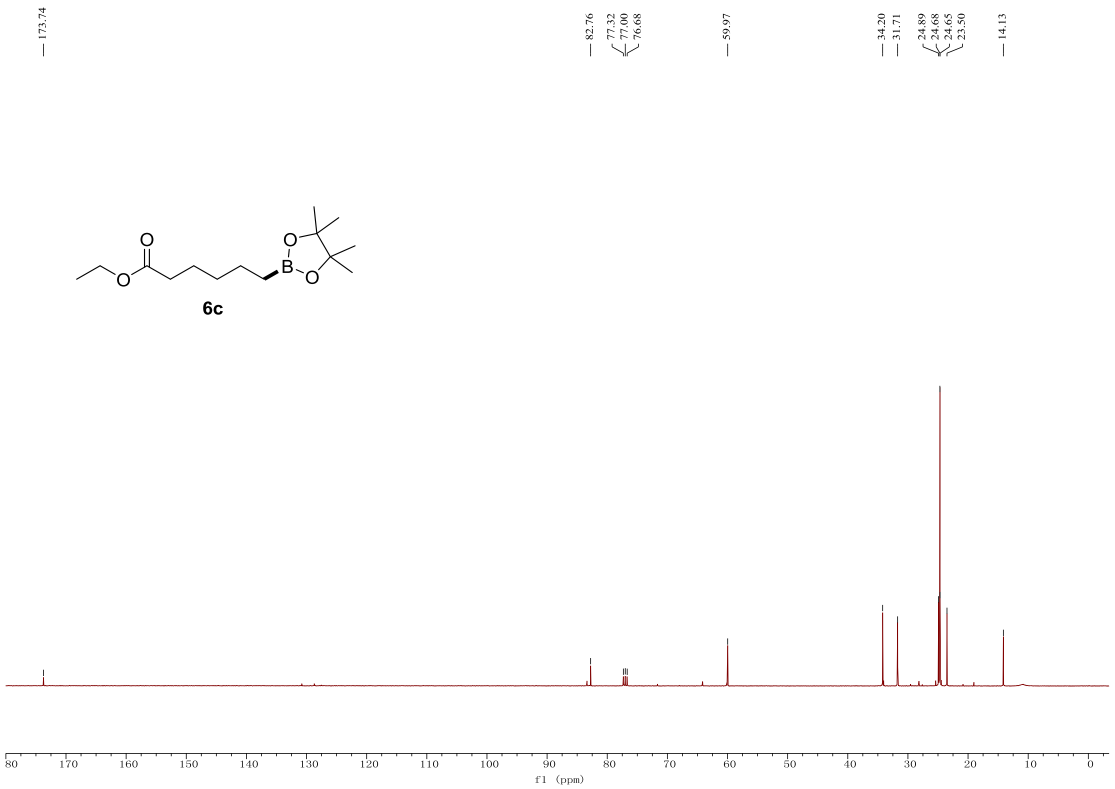

Supplementary Figures 134. ^{13}C NMR spectrum for 6c.

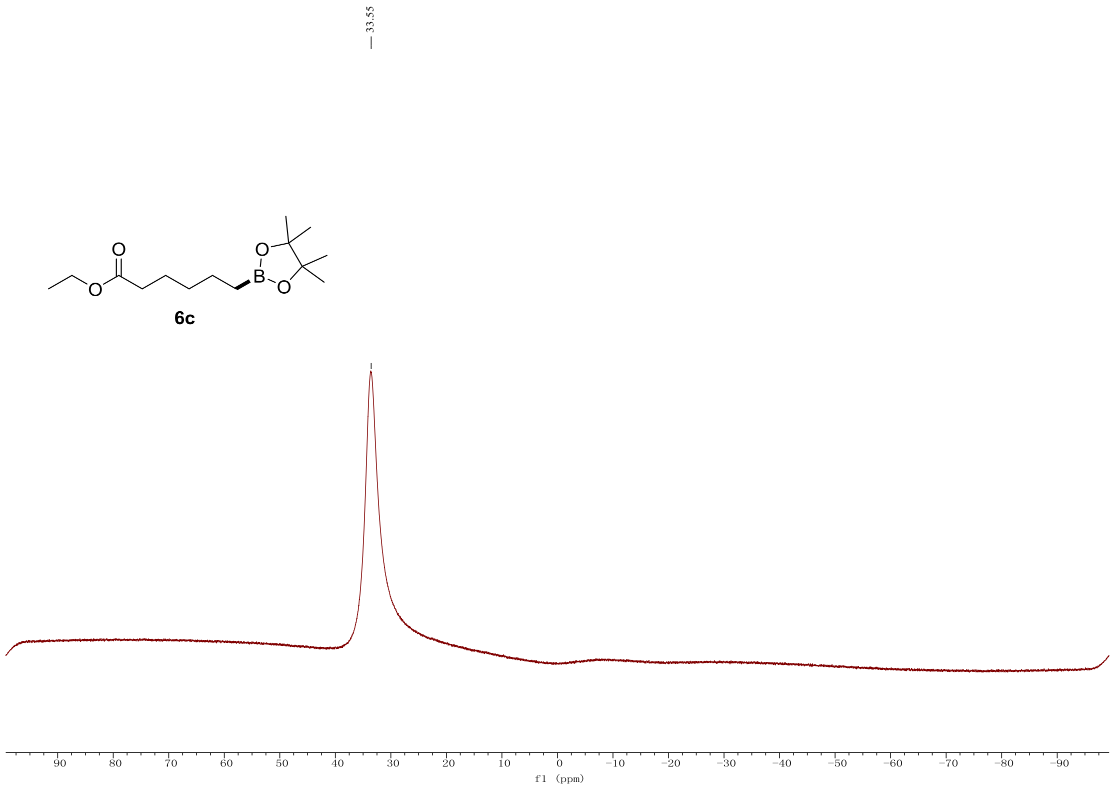

Supplementary Figure 135. ^{11}B NMR spectrum for 6c.

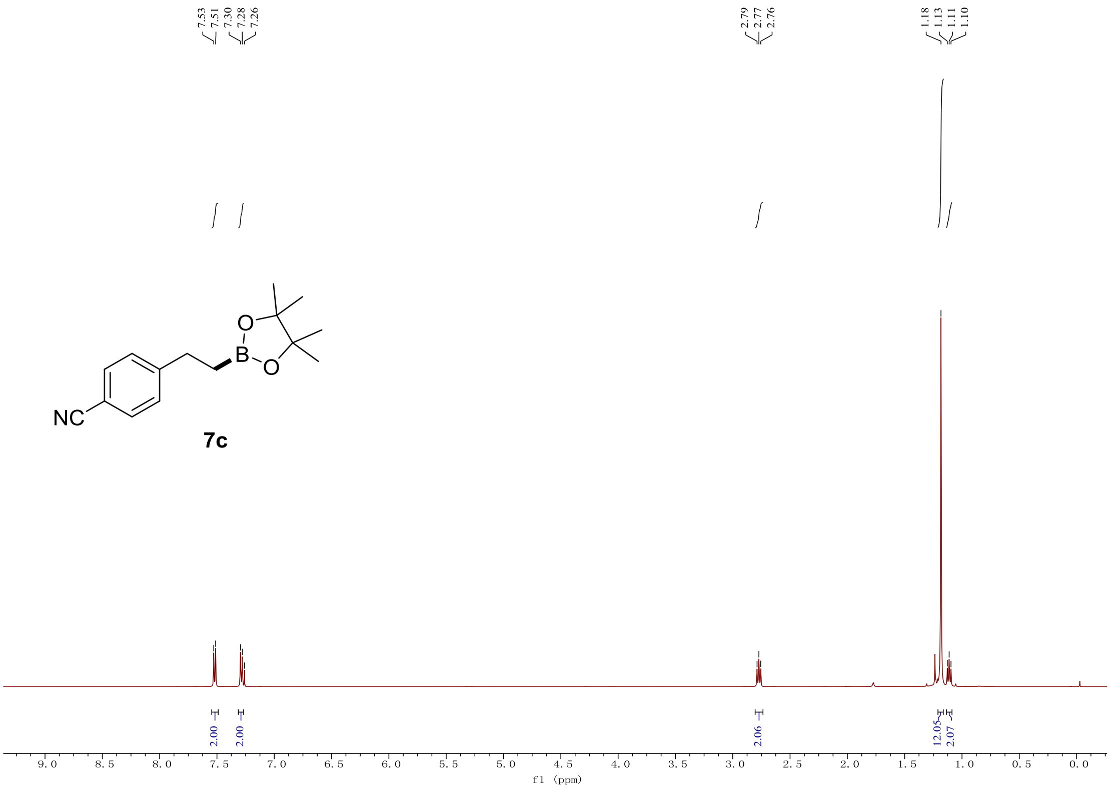

Supplementary Figure 136. ^{1}H NMR spectrum for 7c.

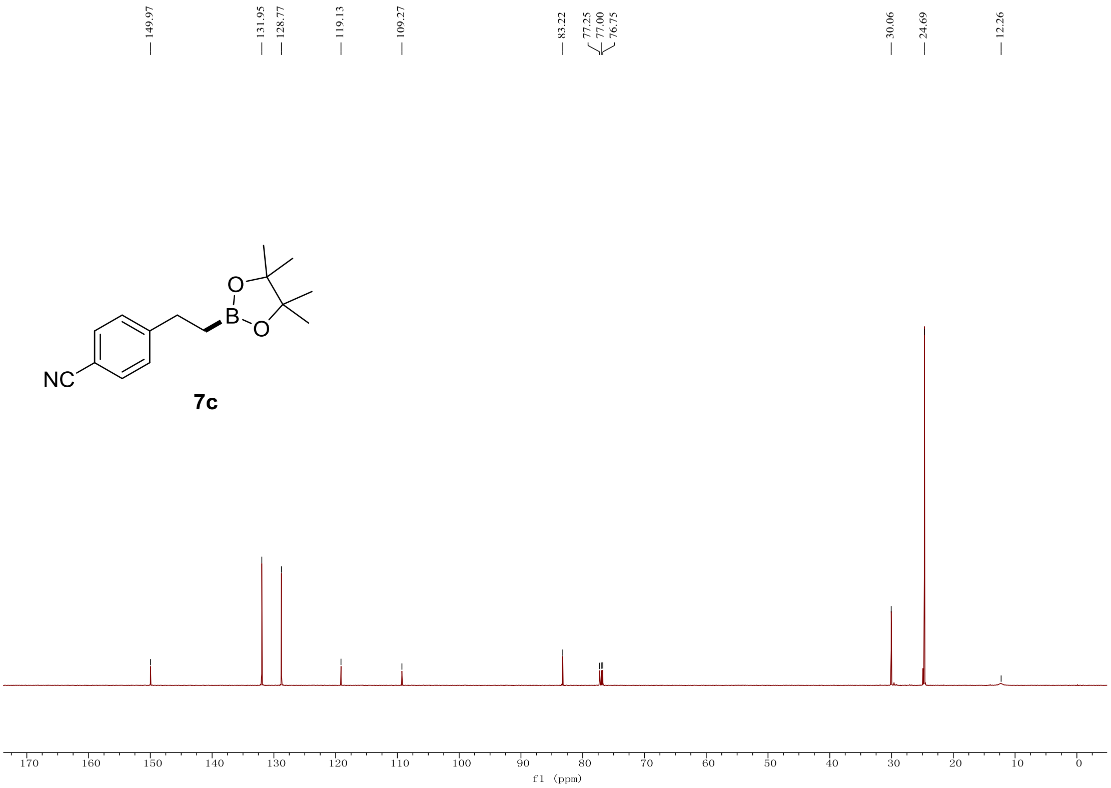

Supplementary Figures 137. ^{13}C NMR spectrum for 7c.

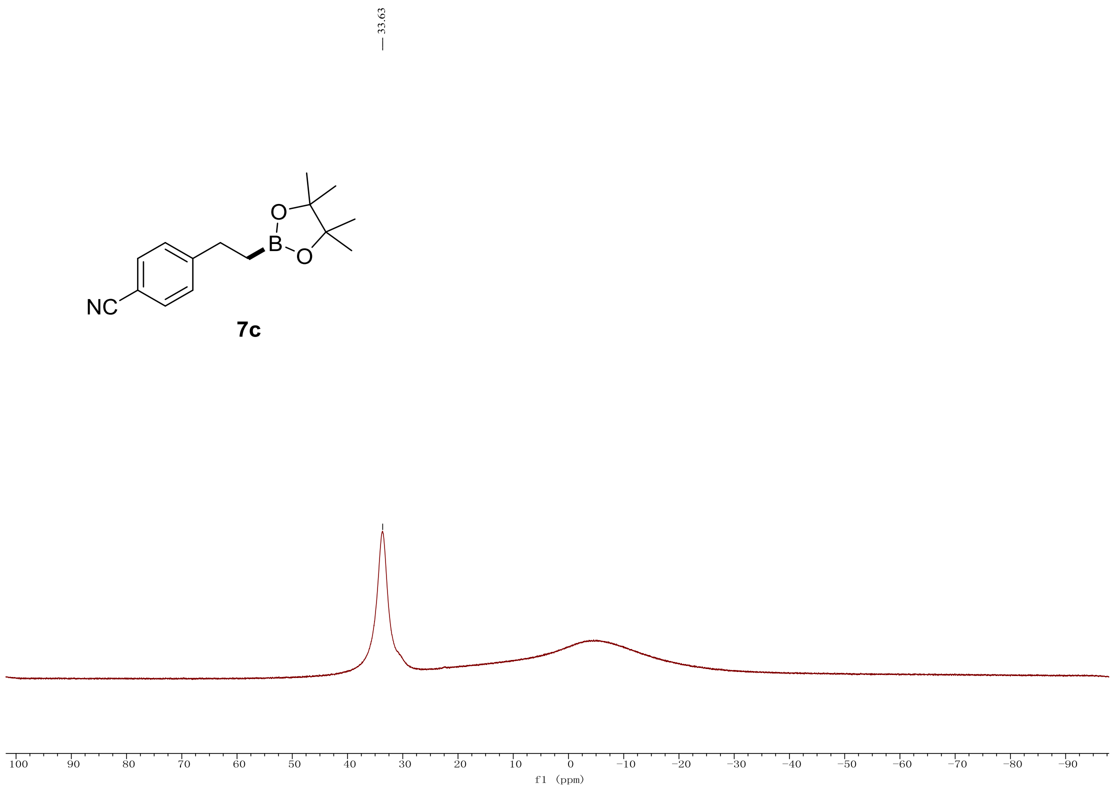

Supplementary Figure 138. ^{11}B NMR spectrum for 7c.

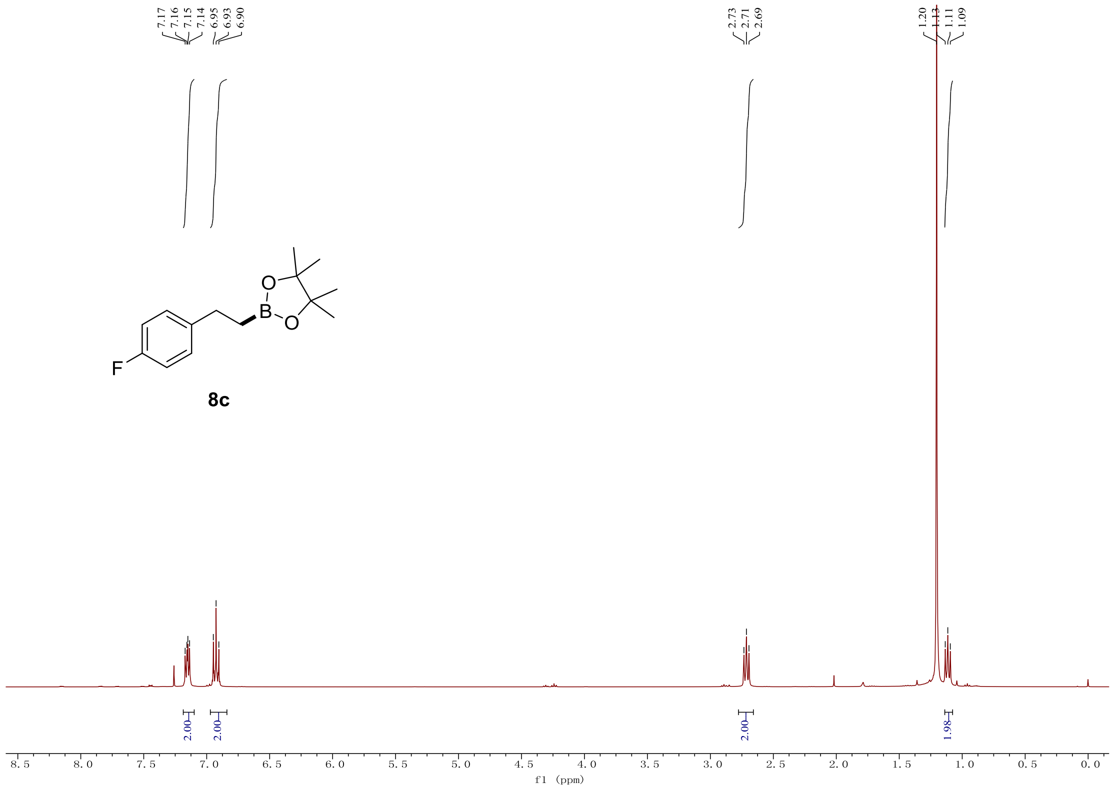

Supplementary Figure 139. ^{1}H NMR spectrum for 8c.

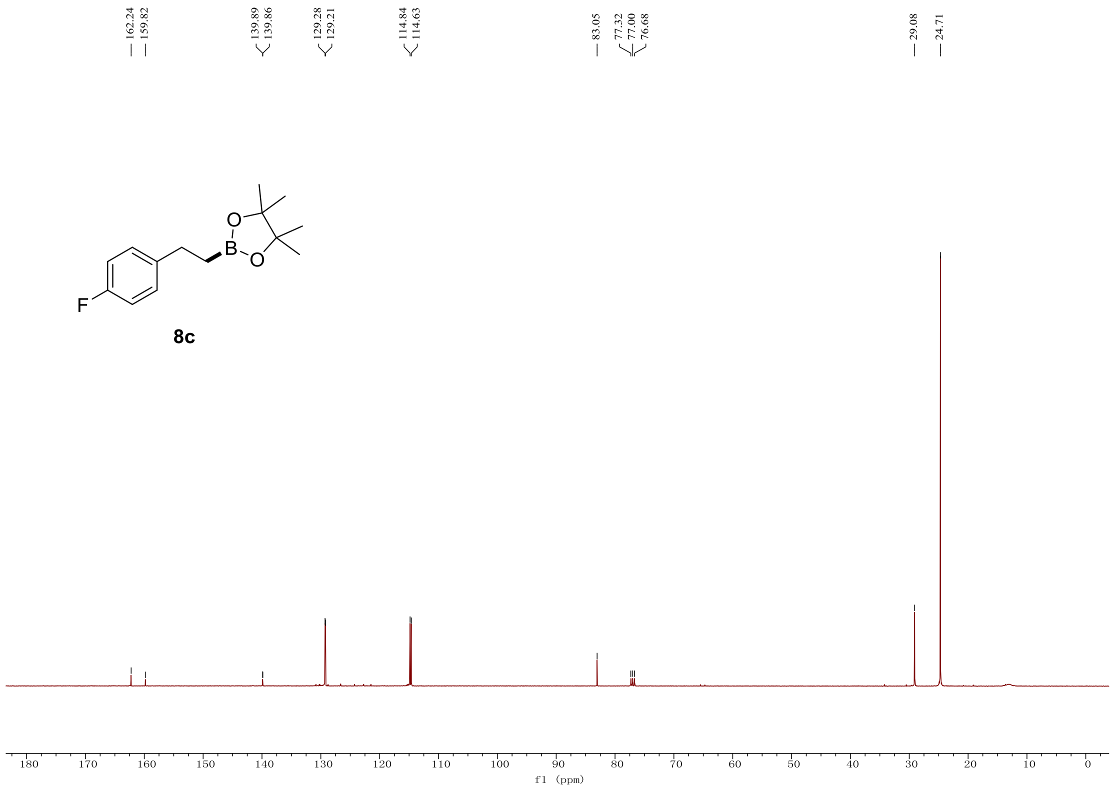

Supplementary Figures 140. ^{13}C NMR spectrum for 8c.

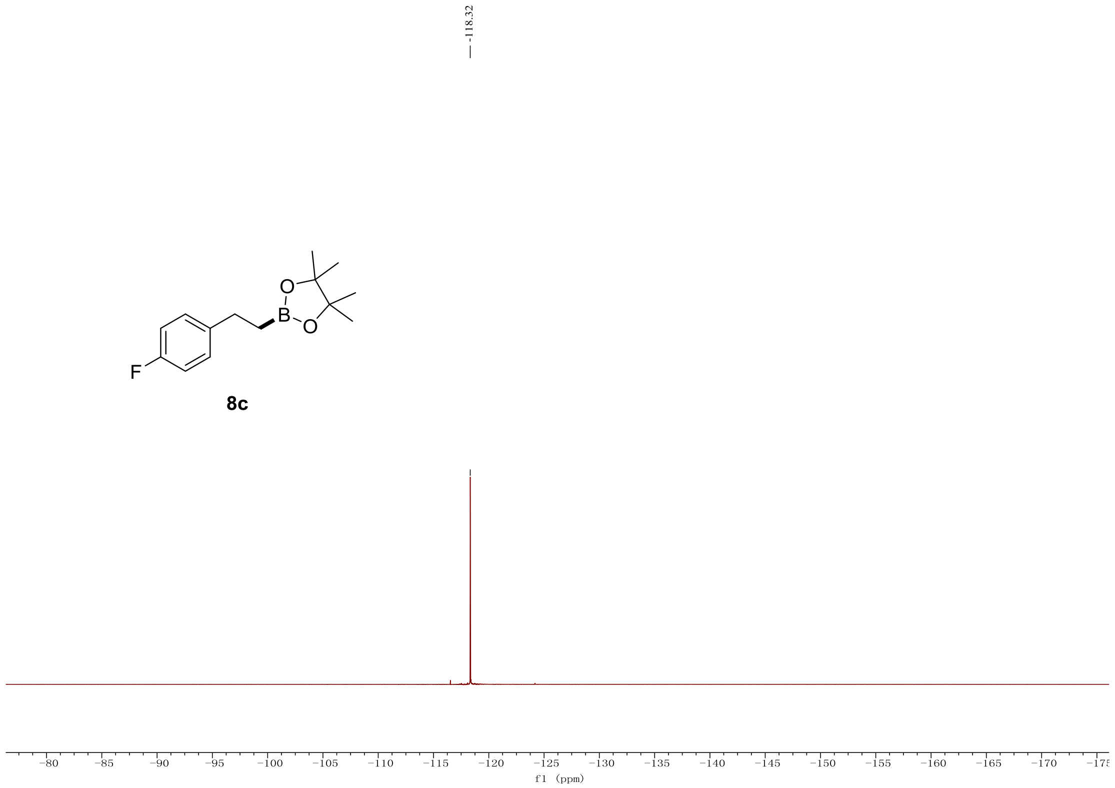

Supplementary Figure 141. ^{19}F NMR spectrum for 8c.

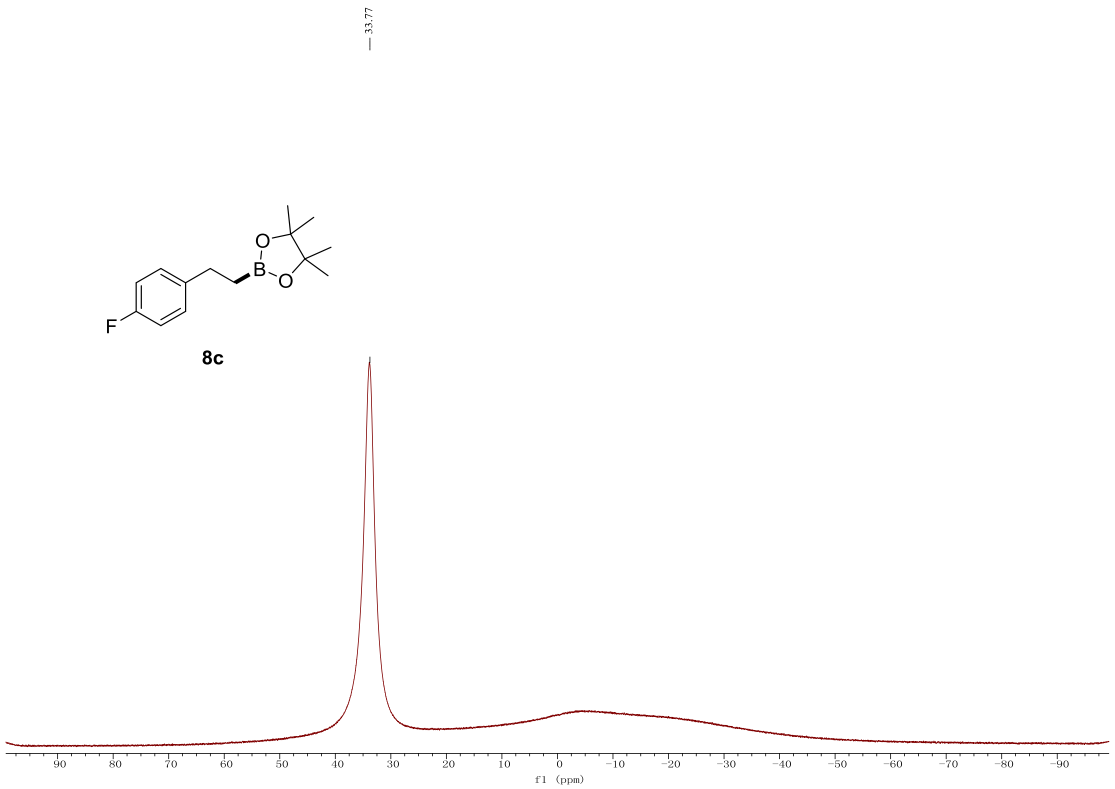

Supplementary Figure 142. ^{11}B NMR spectrum for 8c.

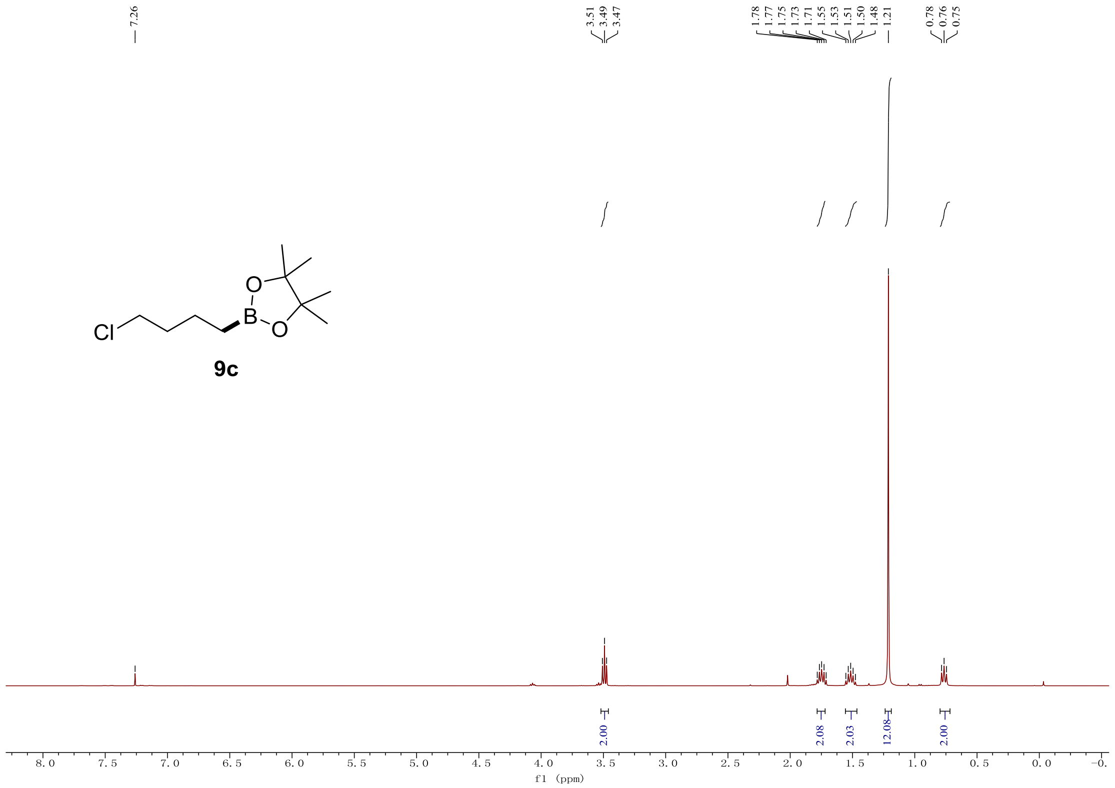

Supplementary Figure 143. ^{1}H NMR spectrum for 9a.

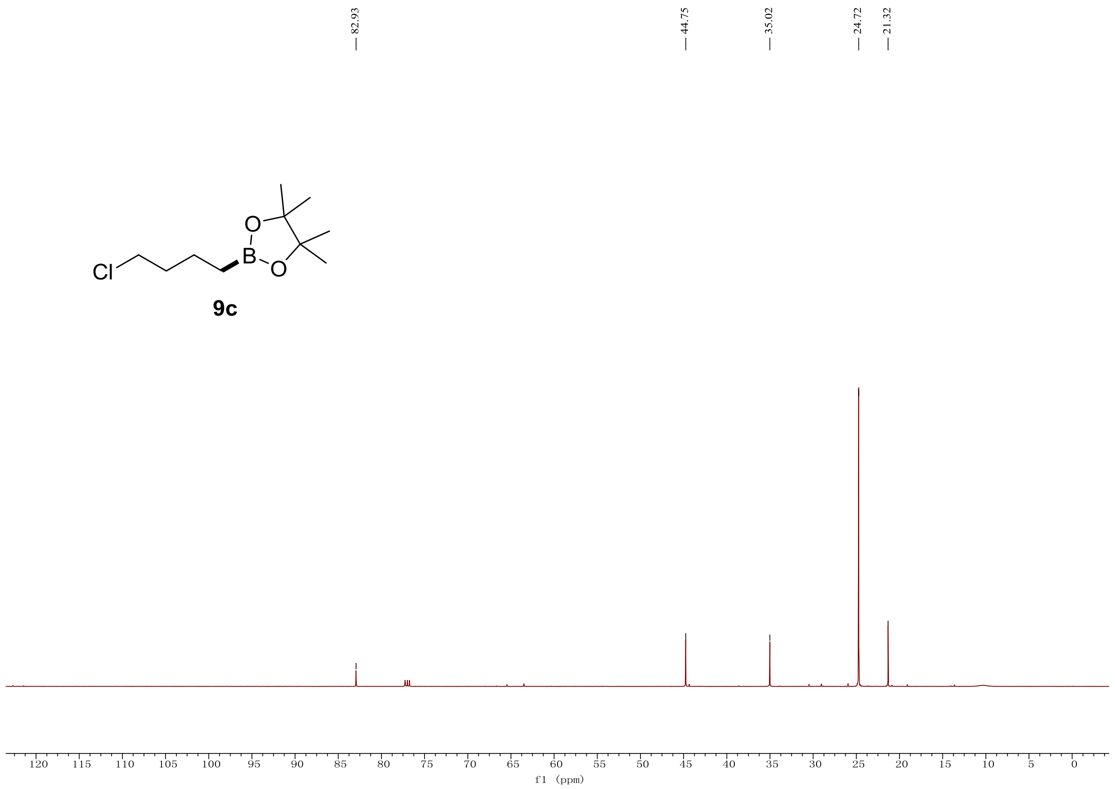

Supplementary Figures 144. ^{13}C NMR spectrum for 9c.

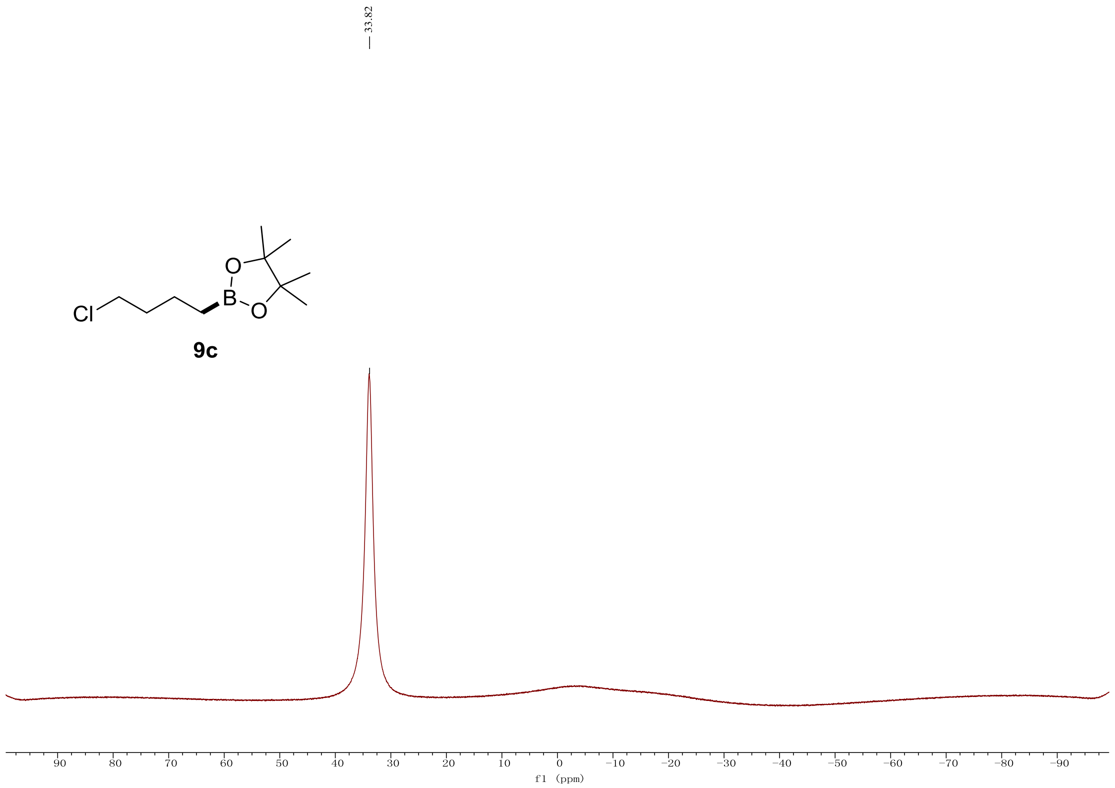

Supplementary Figure 145. ^{11}B NMR spectrum for 9c.

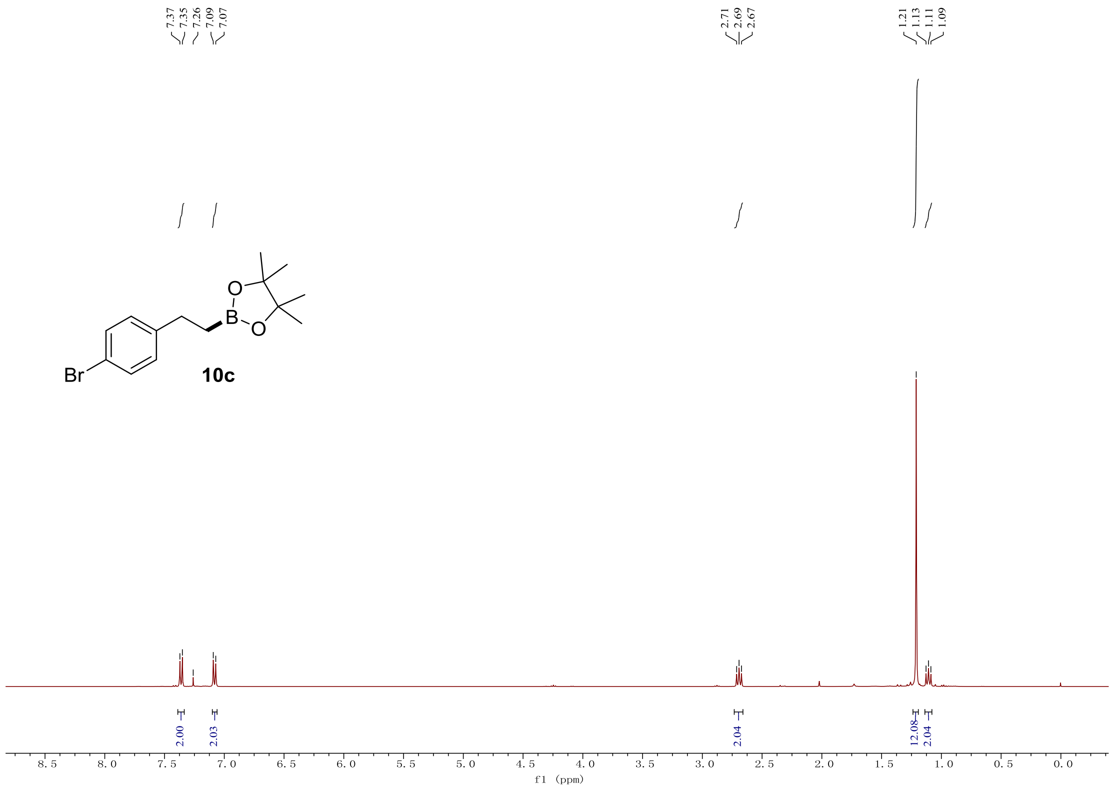

Supplementary Figure 146. ^{1}H NMR spectrum for 10c.

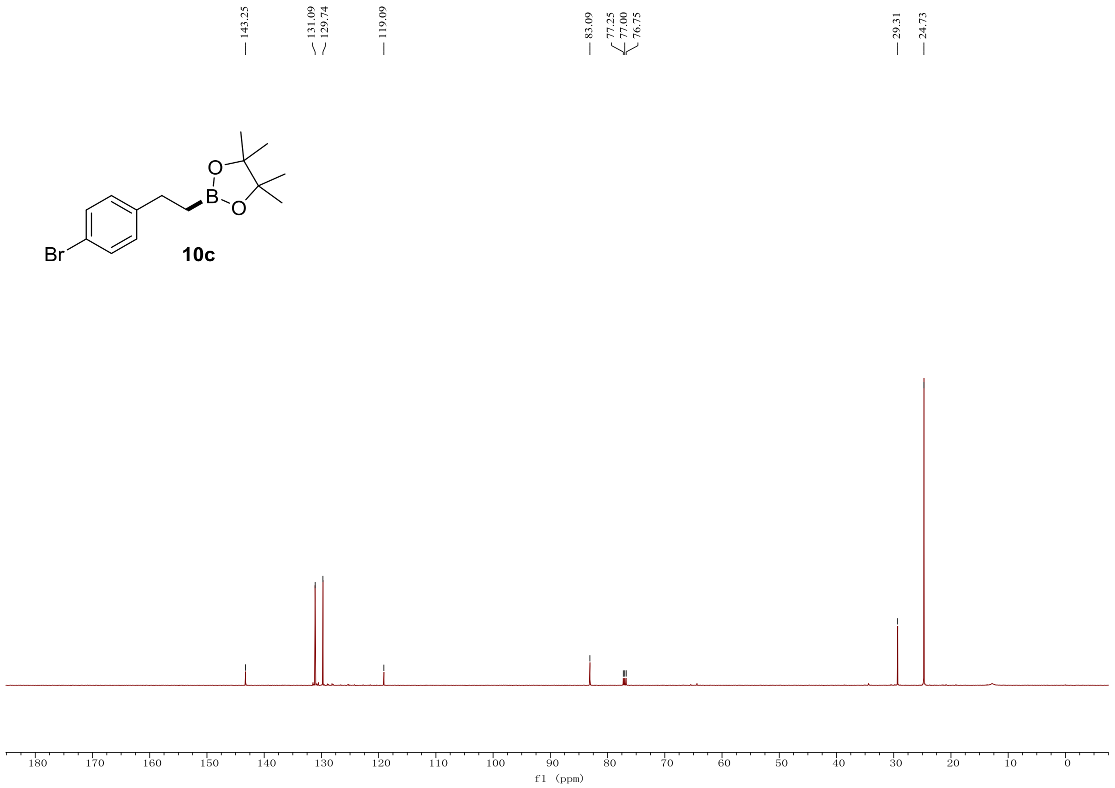

Supplementary Figures 147. ^{13}C NMR spectrum for 10c.

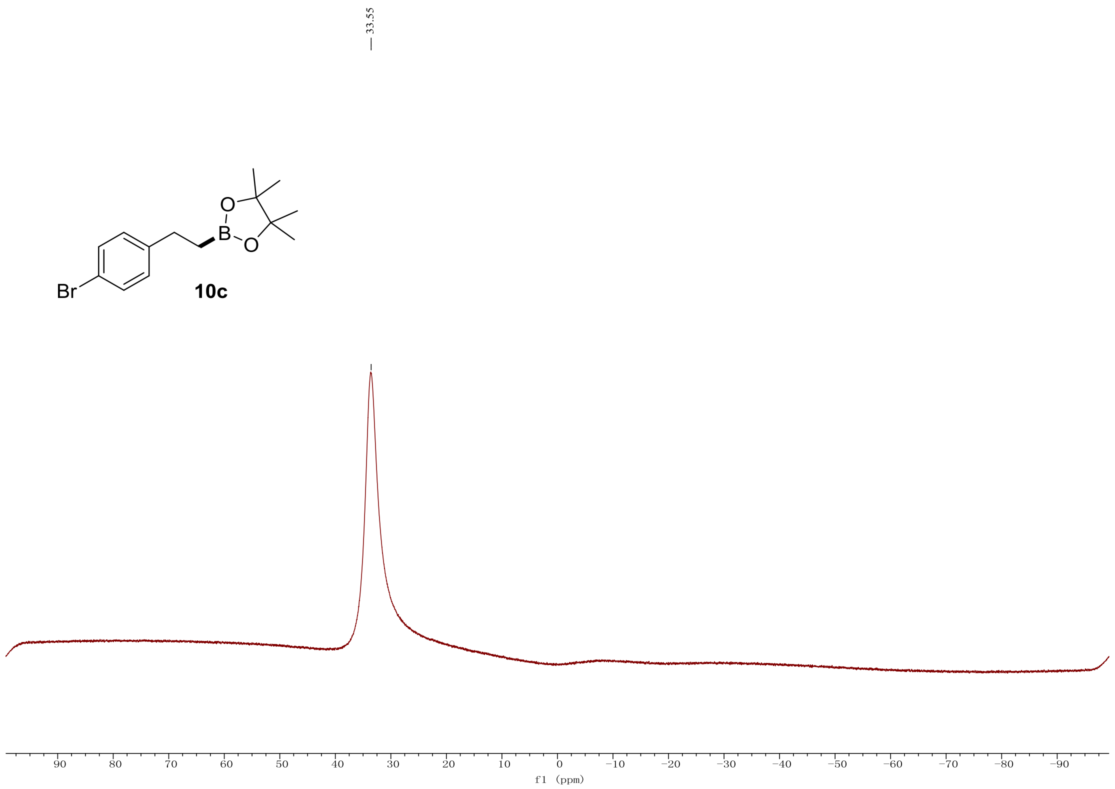

Supplementary Figure 148. ^{11}B NMR spectrum for 10c.

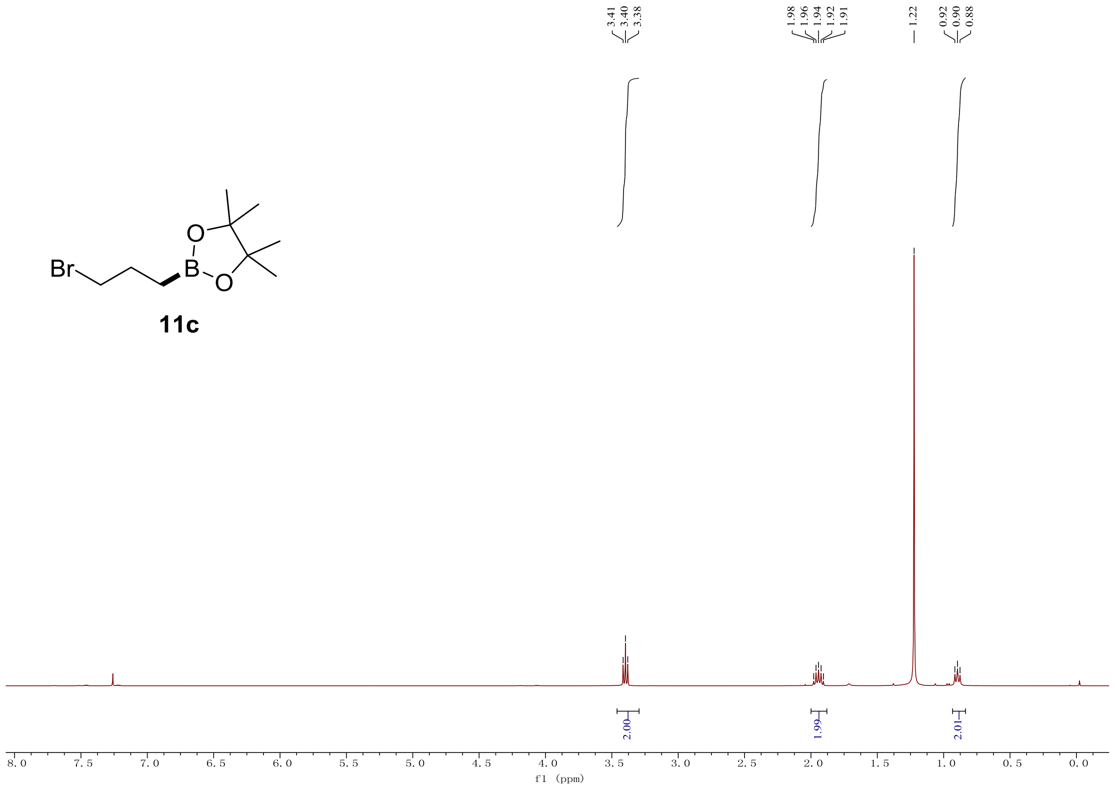

Supplementary Figure 149. ^{1}H NMR spectrum for 11c.

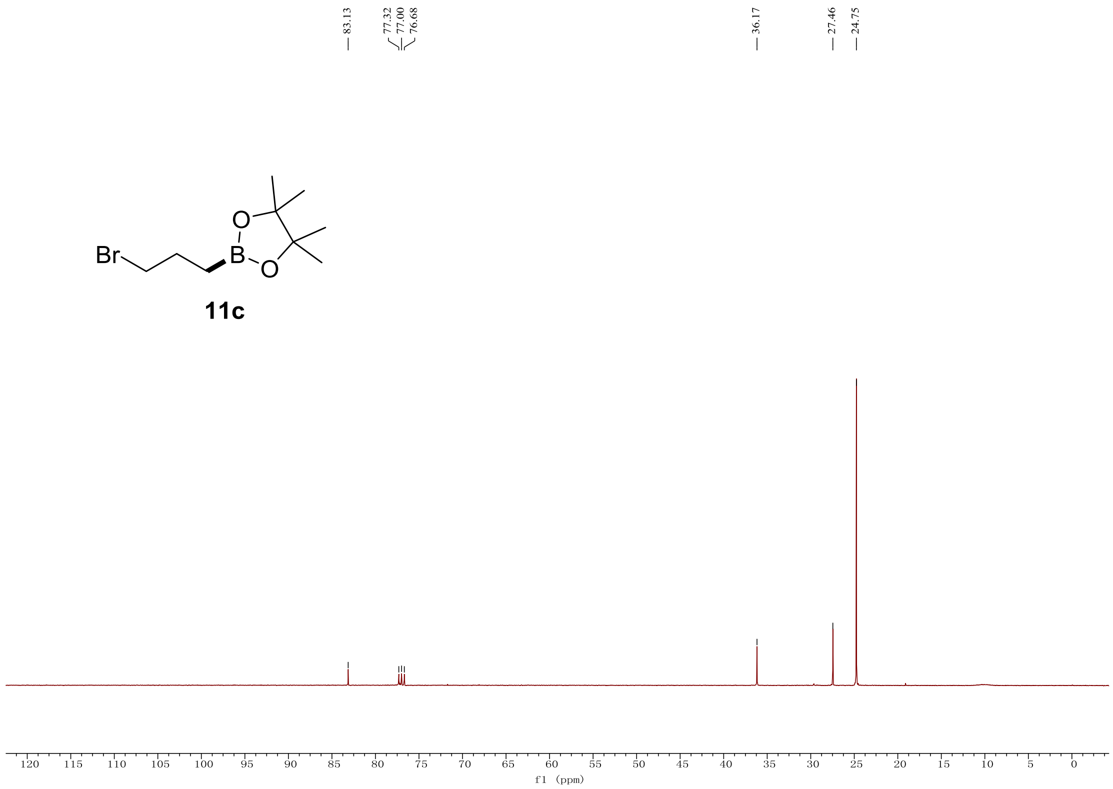

Supplementary Figures 150. ^{13}C NMR spectrum for 11c.

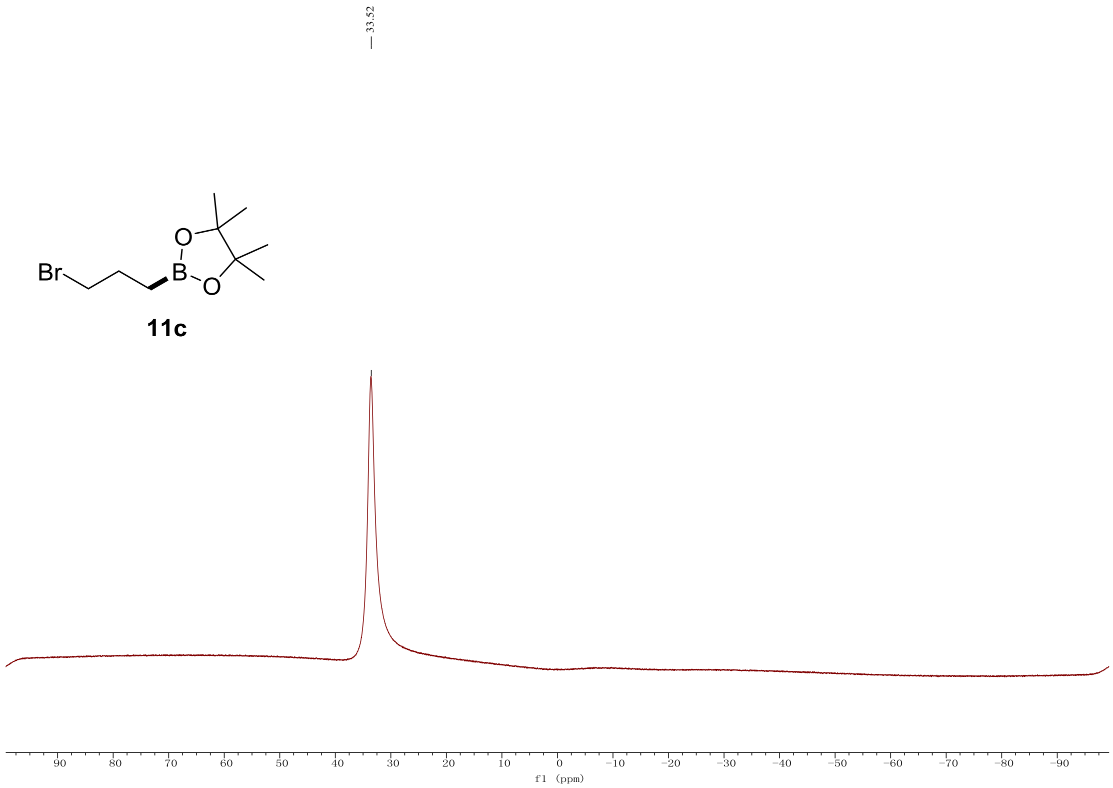

Supplementary Figure 151. ^{11}B NMR spectrum for 11c.

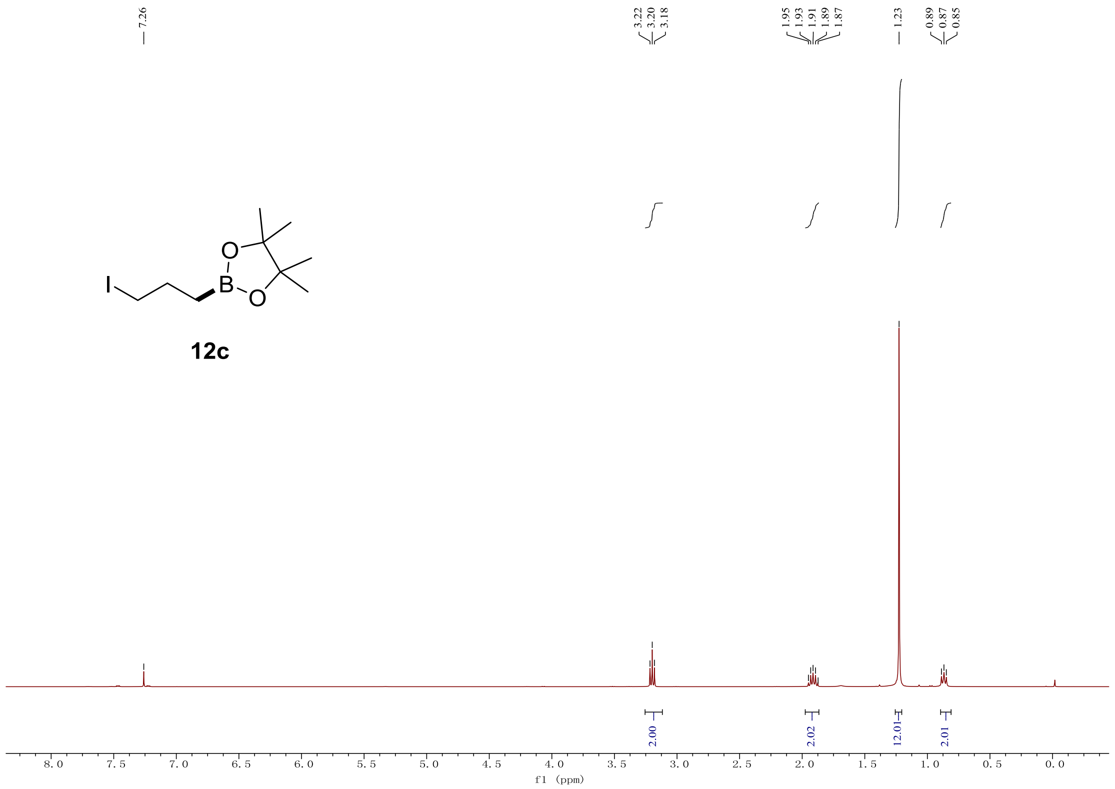

Supplementary Figure 152. ^{1}**H NMR spectrum for 12c.**

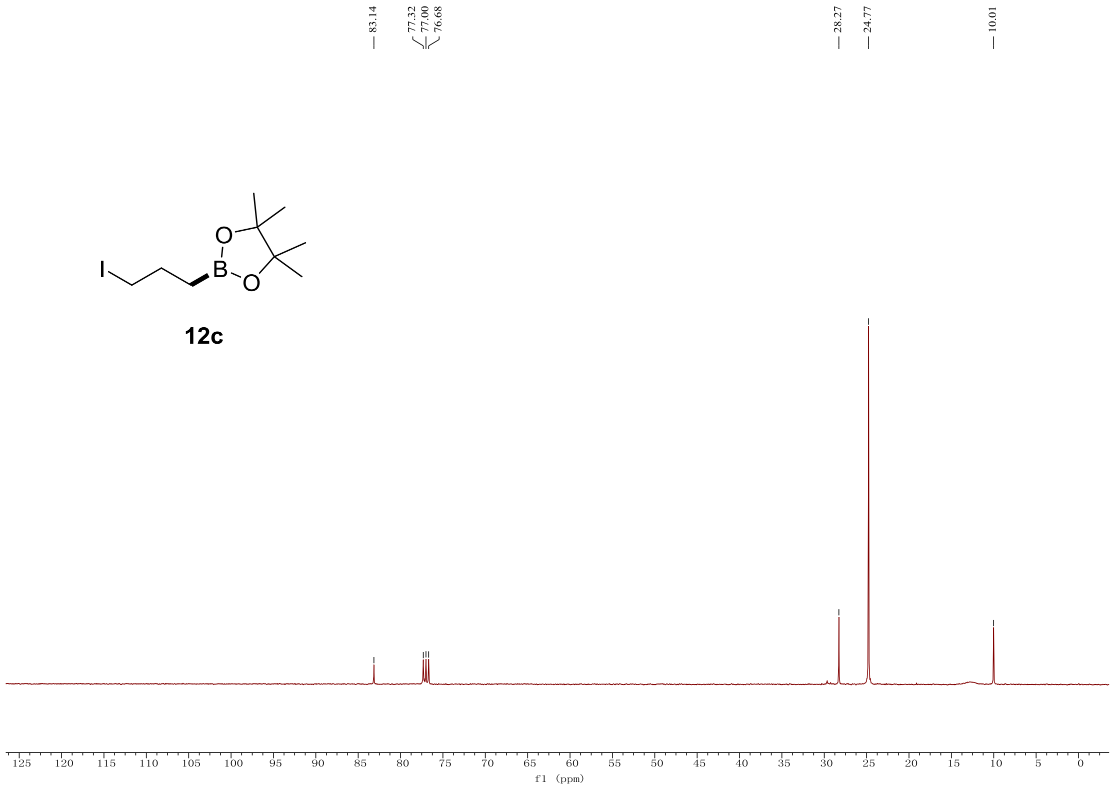

Supplementary Figures 153. ^{13}**C NMR spectrum for 12c.**

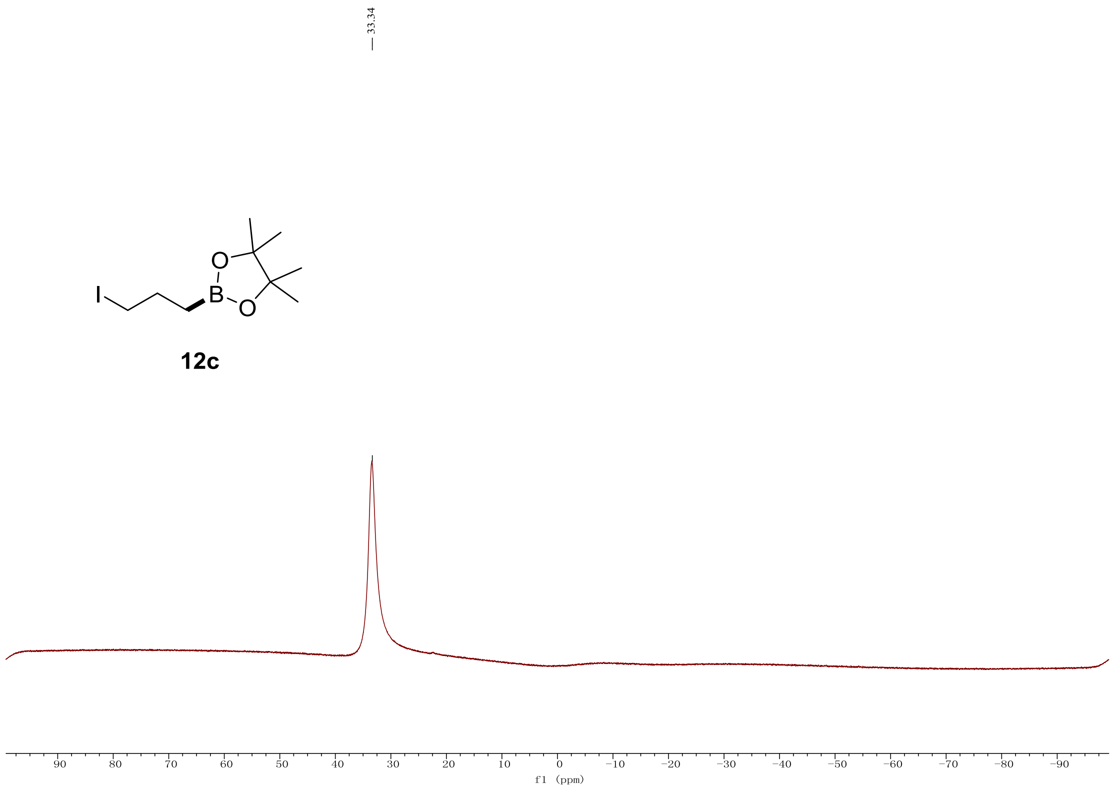

Supplementary Figure 154. ^{11}B NMR spectrum for 12c.

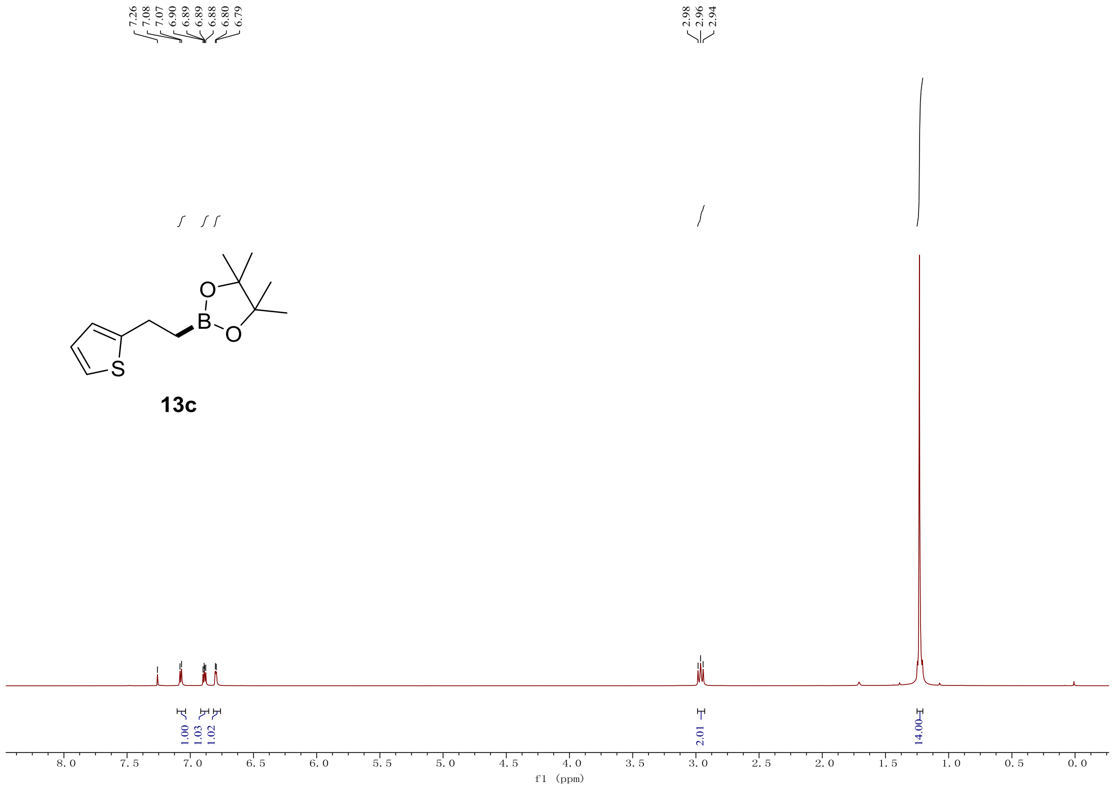

Supplementary Figure 155. ^{1}H NMR spectrum for 13c.

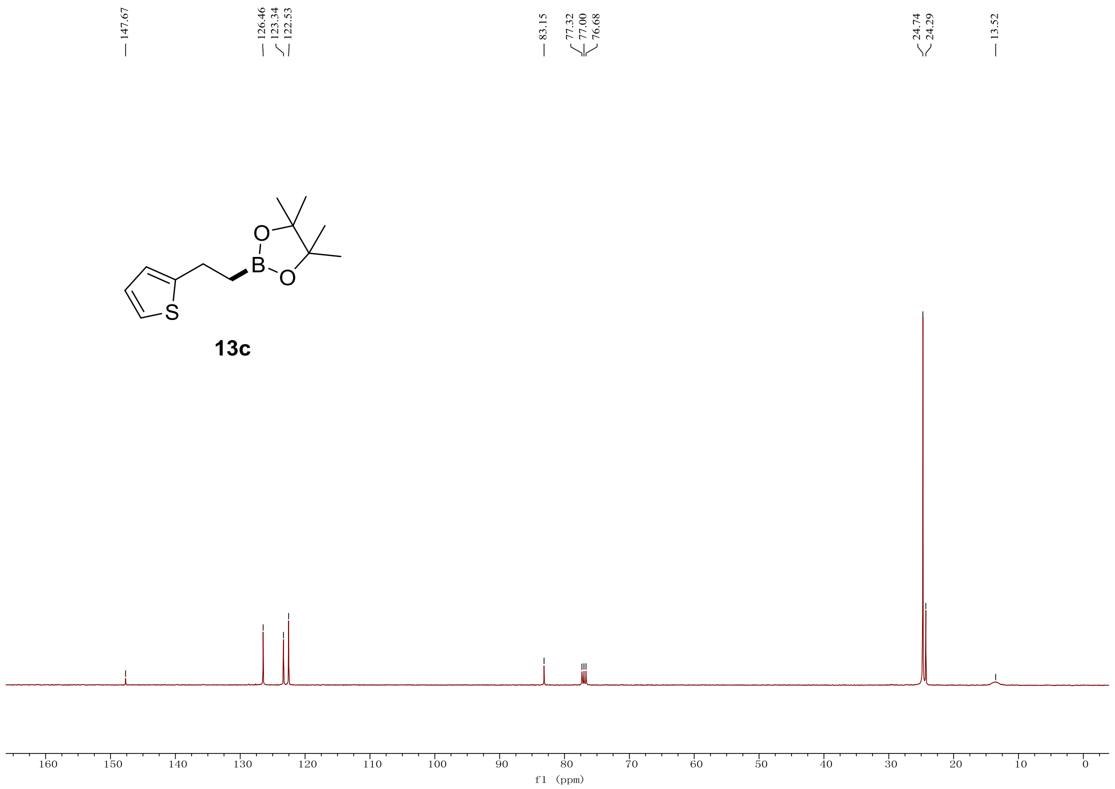

Supplementary Figures 156. ^{13}C NMR spectrum for 13c.

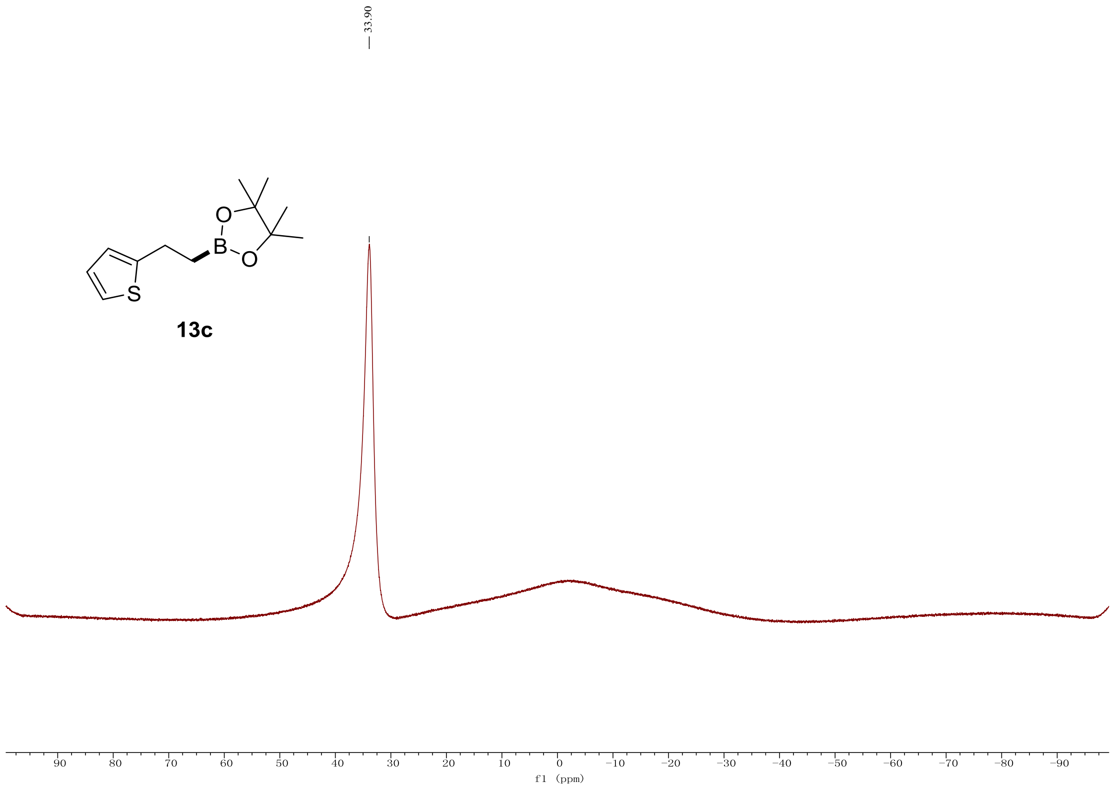

Supplementary Figure 157. ^{11}B NMR spectrum for 13c.

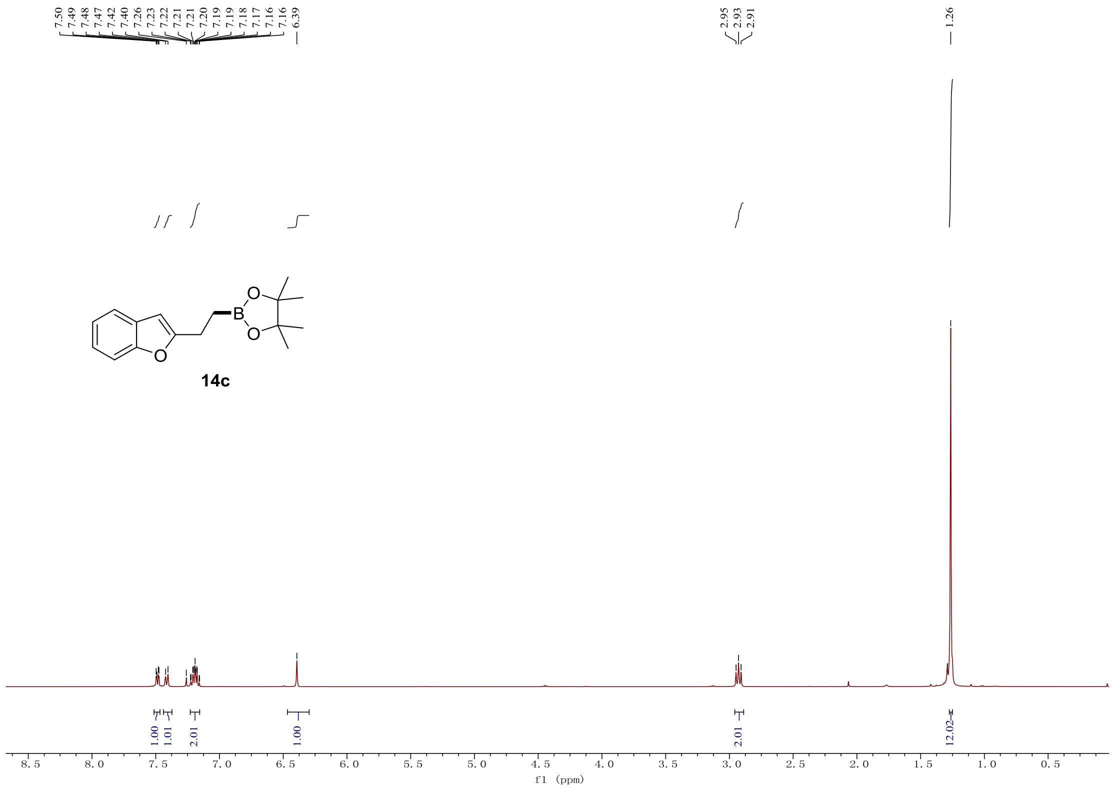

Supplementary Figure 158. ^{1}H NMR spectrum for 14c.

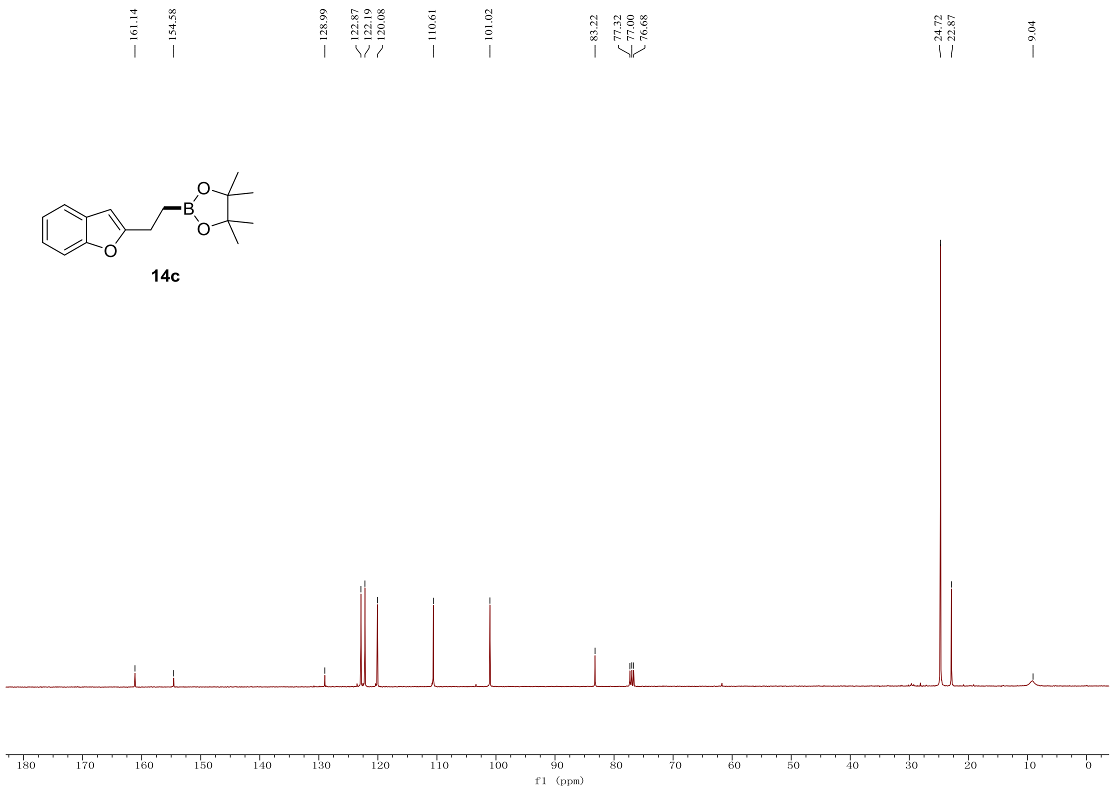

Supplementary Figures 159. ^{13}C NMR spectrum for 14c.

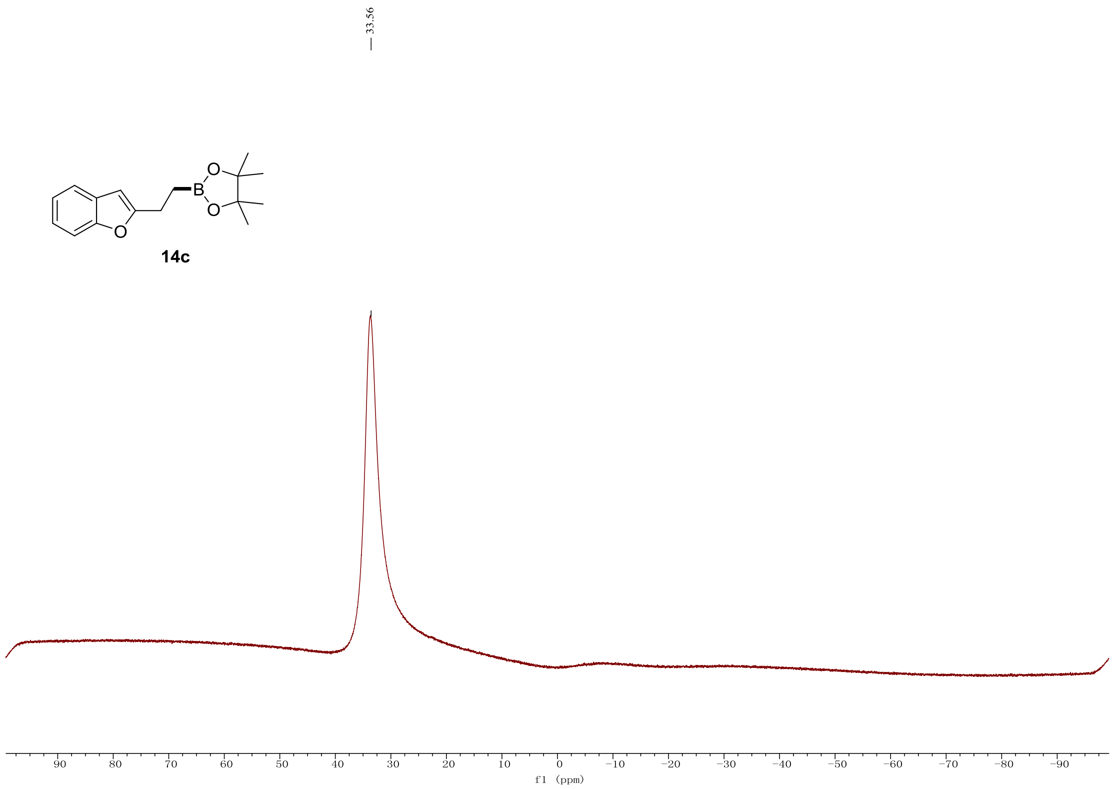

Supplementary Figure 160. ^{11}B NMR spectrum for 14c.

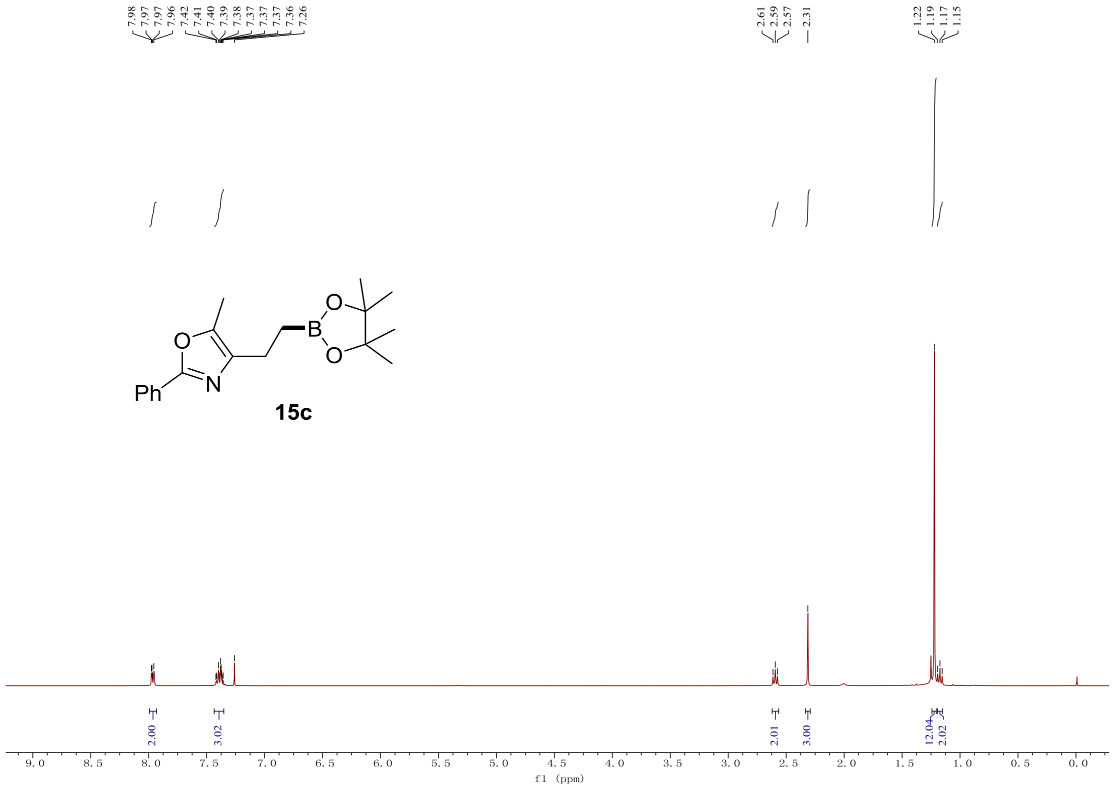

Supplementary Figure 161. ^{1}H NMR spectrum for 15c.

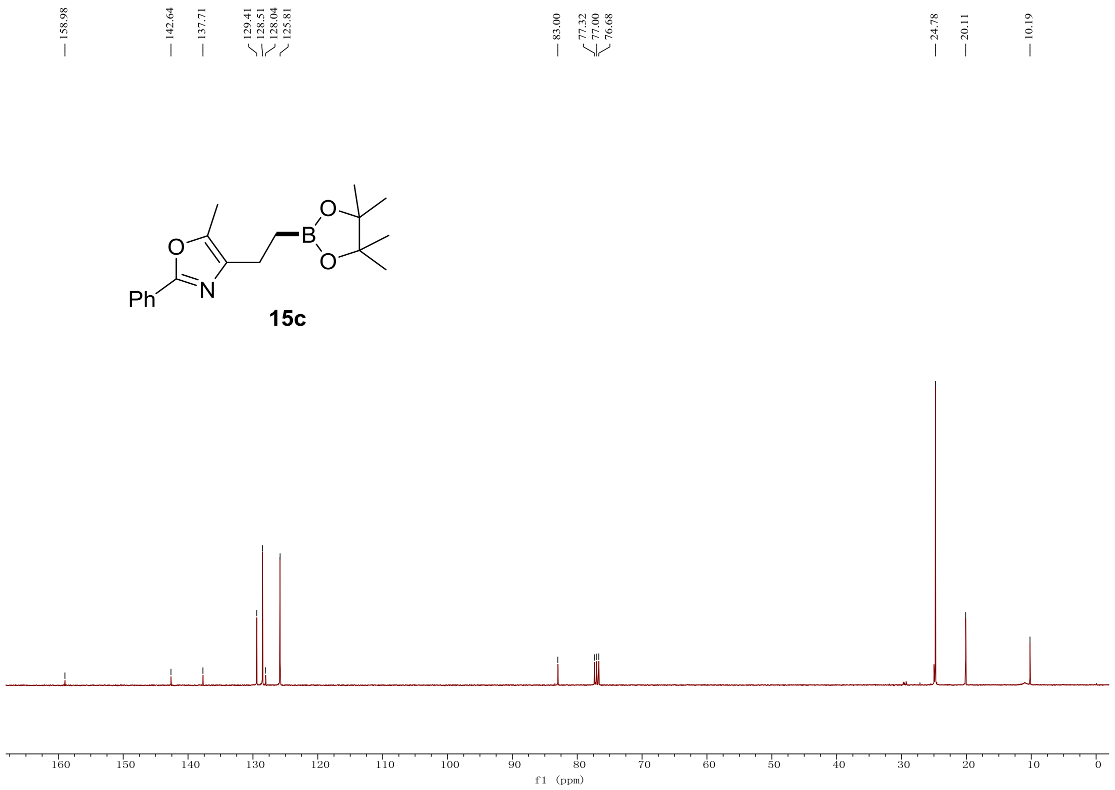

Supplementary Figures 162. ^{13}C NMR spectrum for 15c.

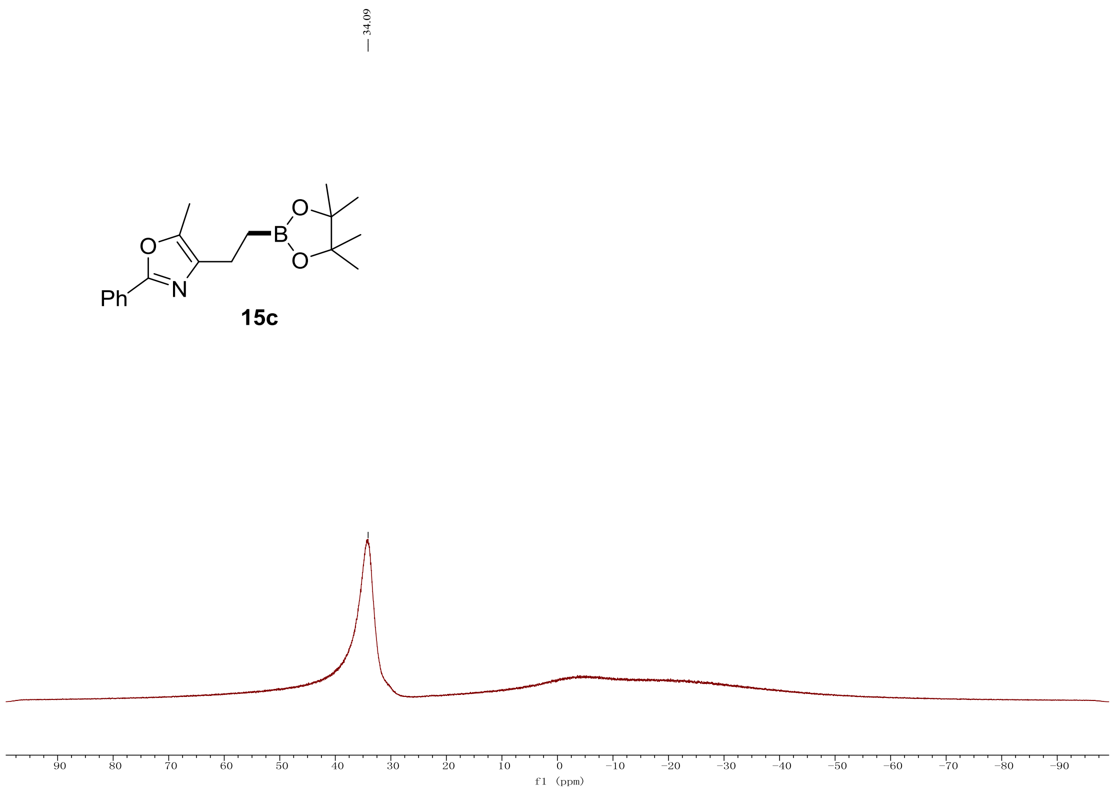

Supplementary Figure 163. ^{11}B NMR spectrum for 15c.

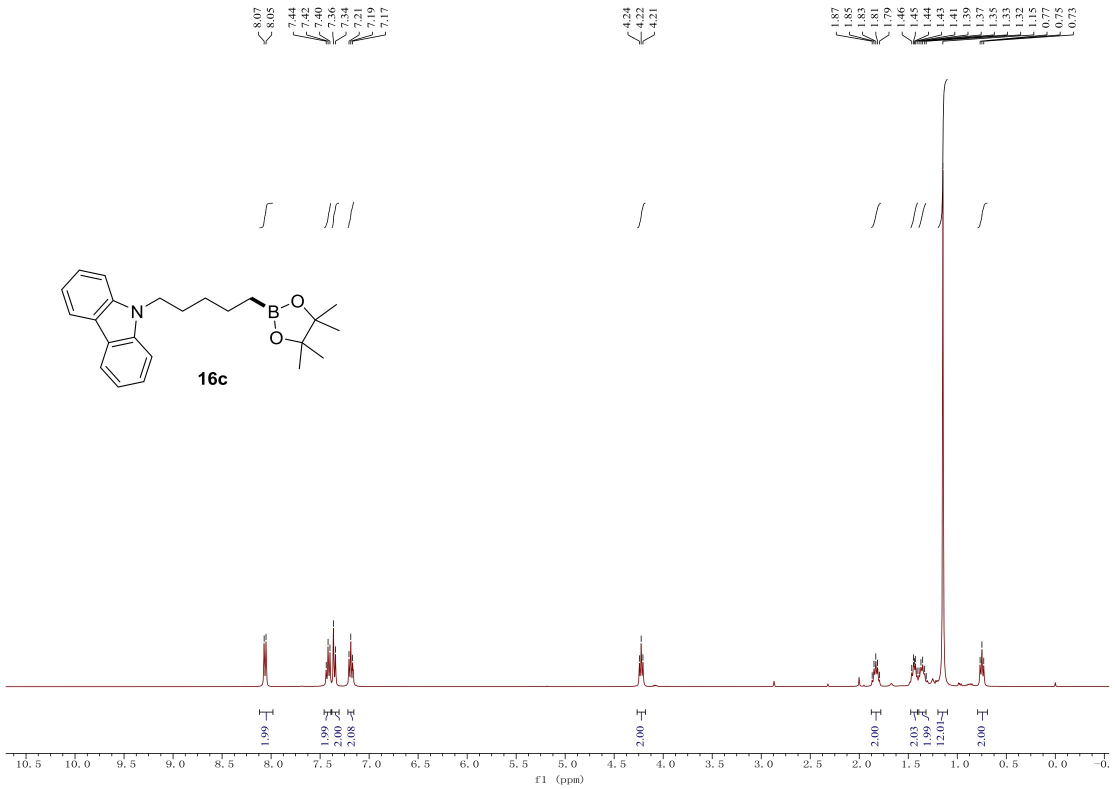

Supplementary Figure 164. ^{1}H NMR spectrum for 16c.

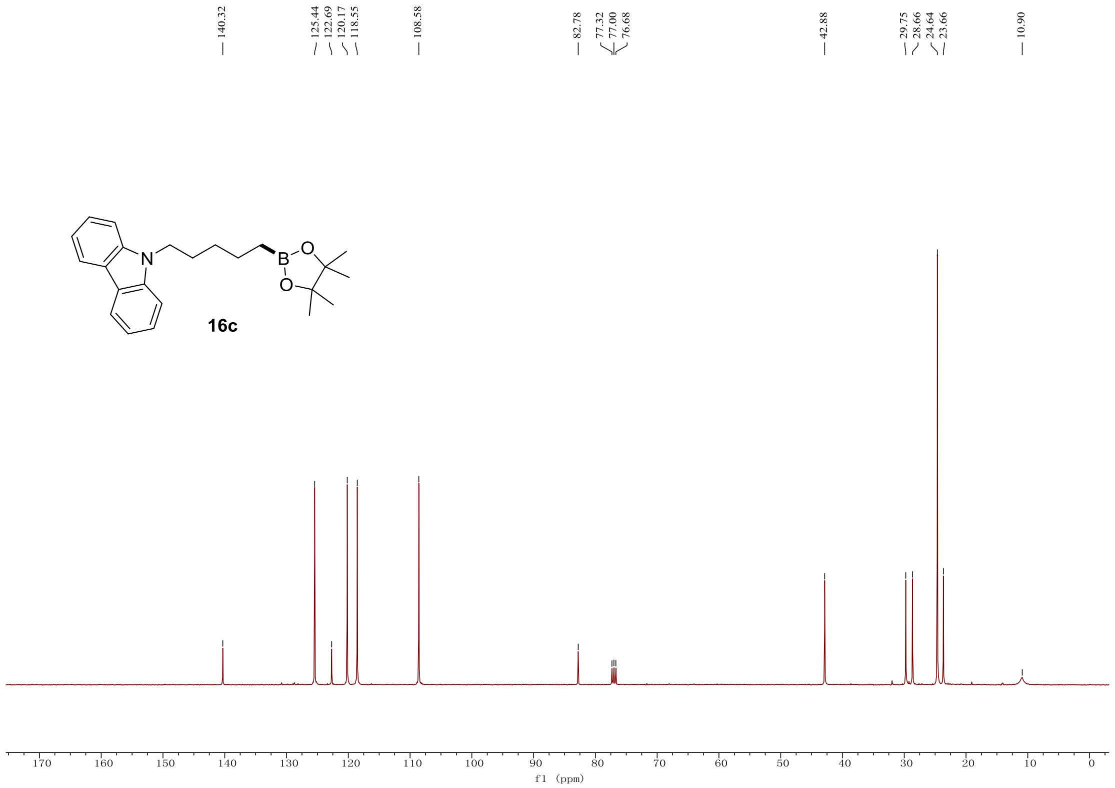

Supplementary Figures 165. ^{13}C NMR spectrum for 16c.

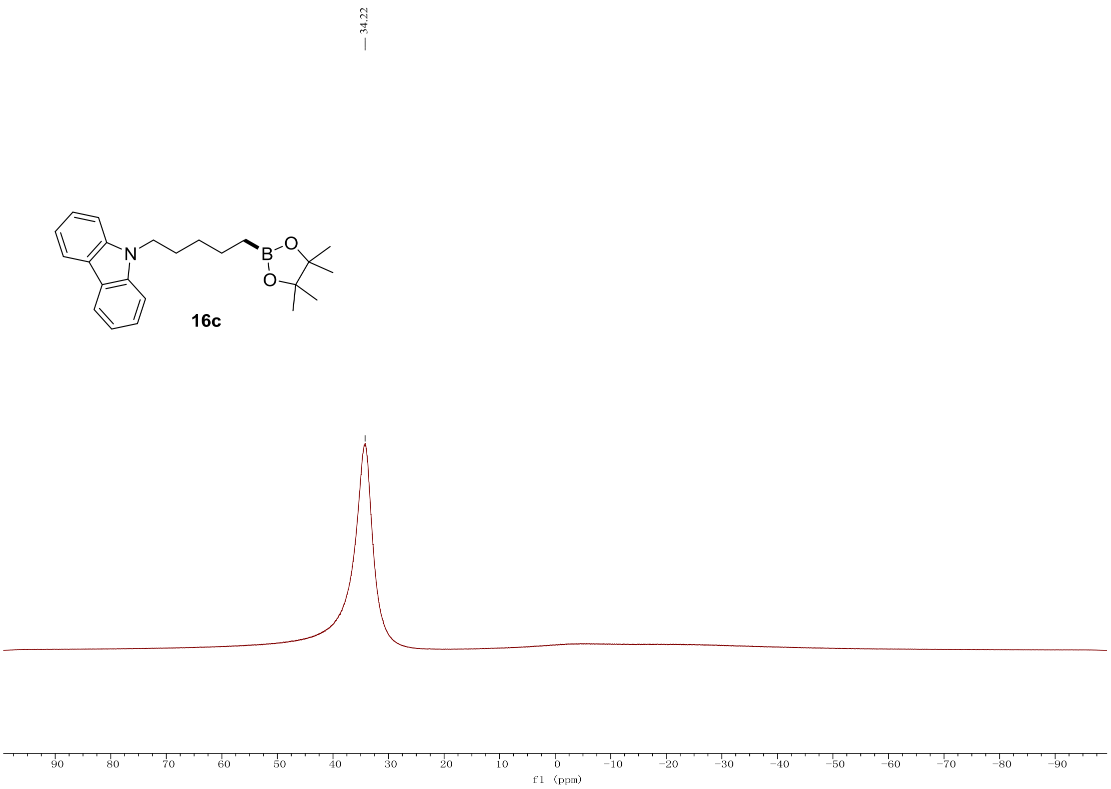

Supplementary Figure 166. ^{11}B NMR spectrum for 16c.

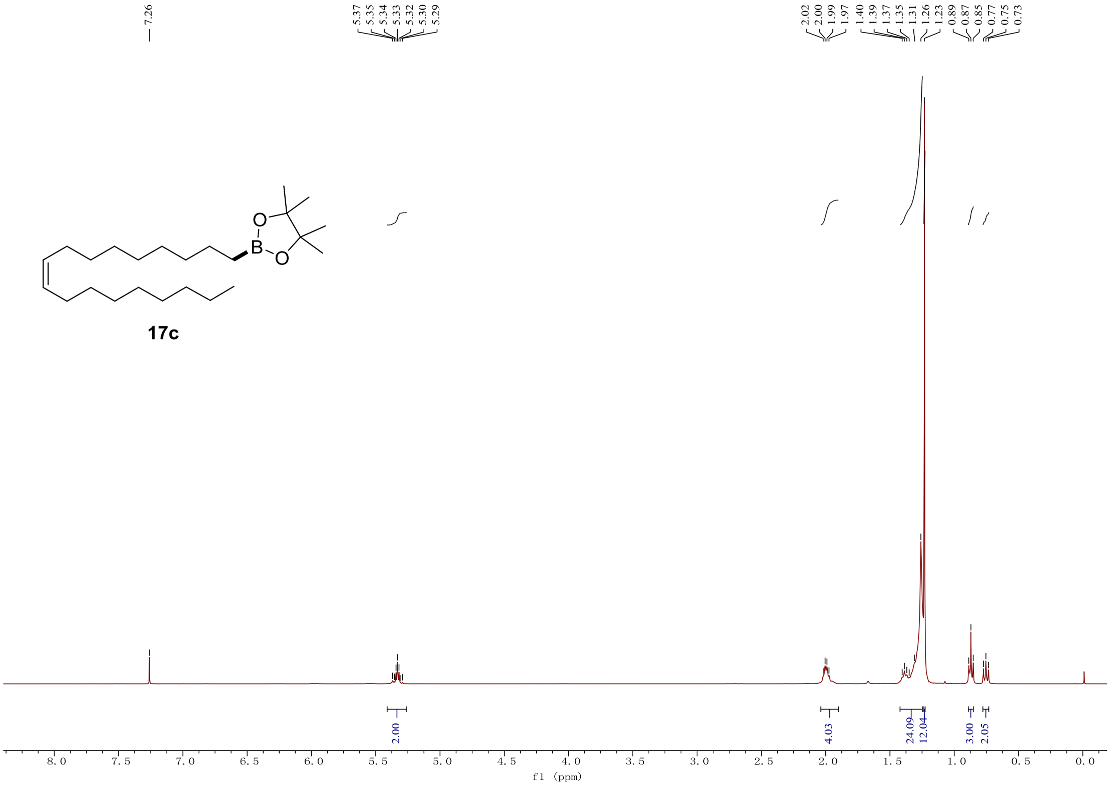

Supplementary Figure 167. ^{1}H NMR spectrum for 17c.

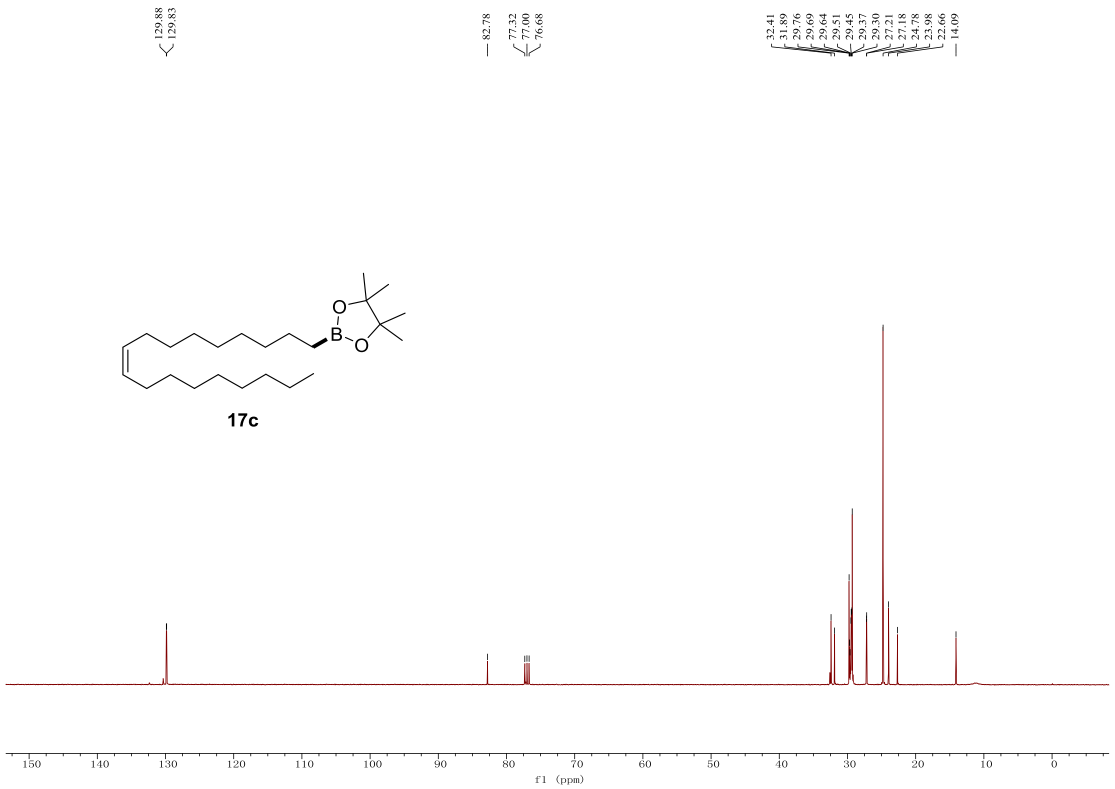

Supplementary Figures 168. ^{13}C NMR spectrum for 17c.

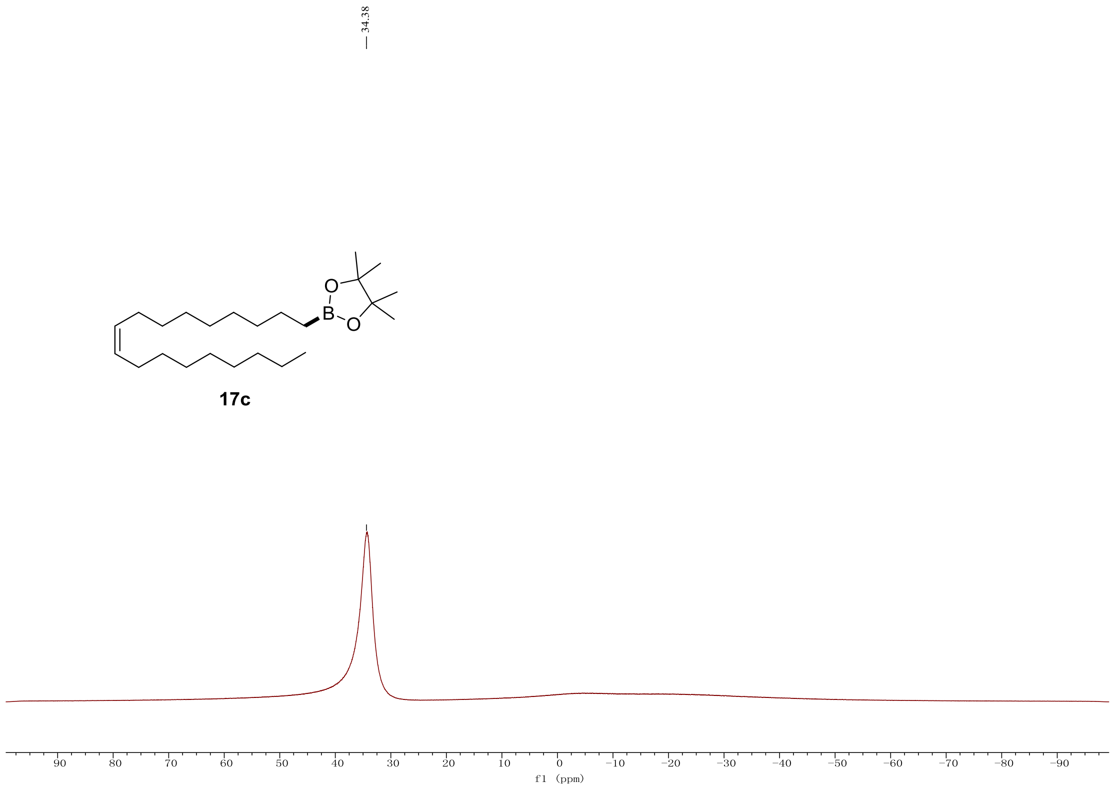

Supplementary Figure 169. ^{11}B NMR spectrum for 17c.

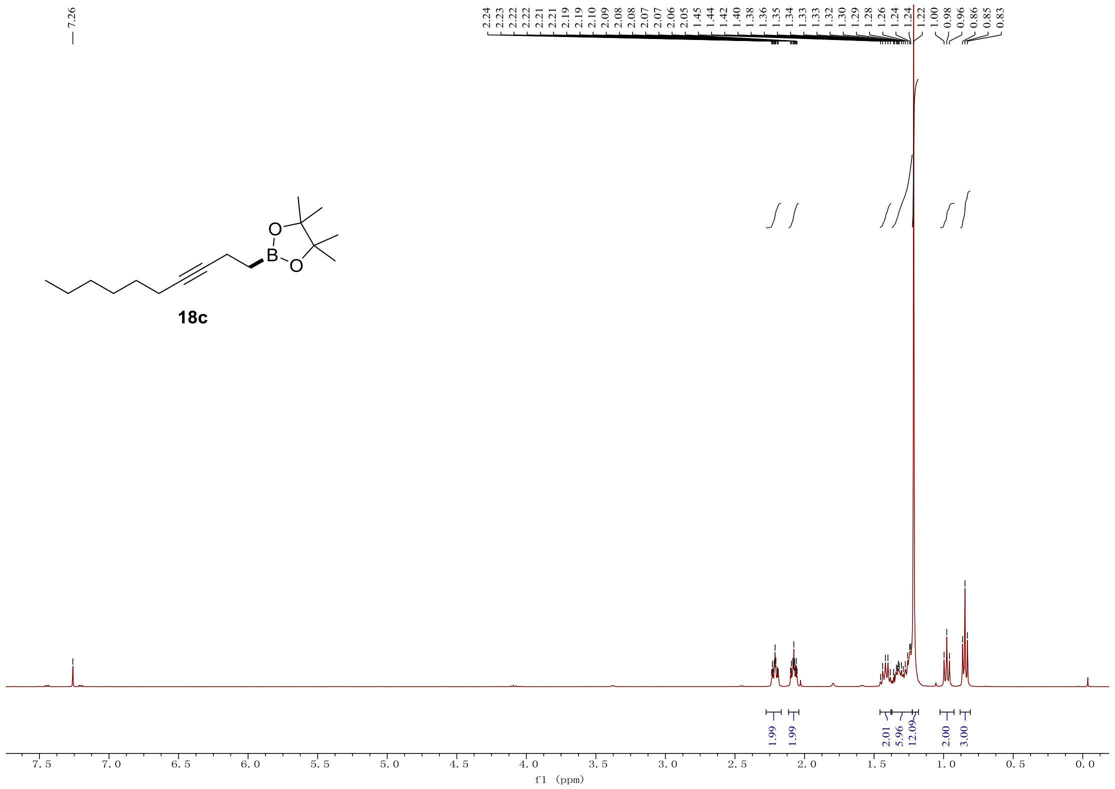

Supplementary Figure 170. ^{1}H NMR spectrum for 18c.

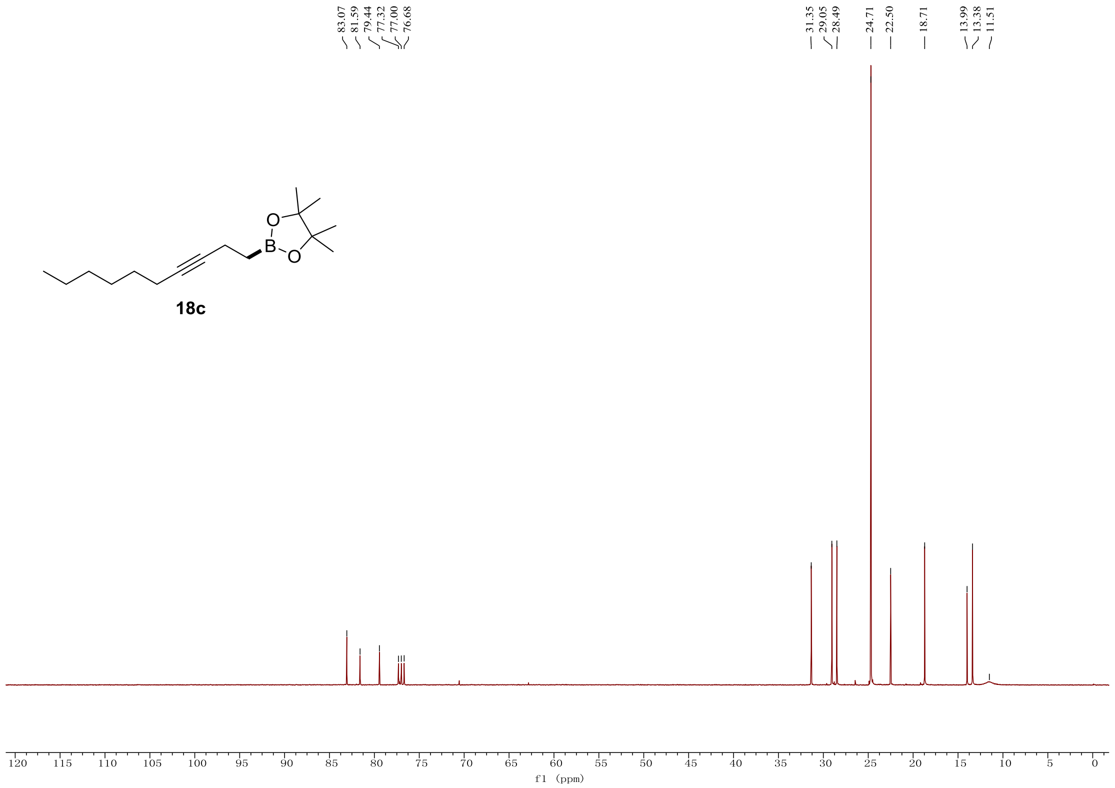

Supplementary Figure 171. ^{13}C NMR spectrum for 18c.

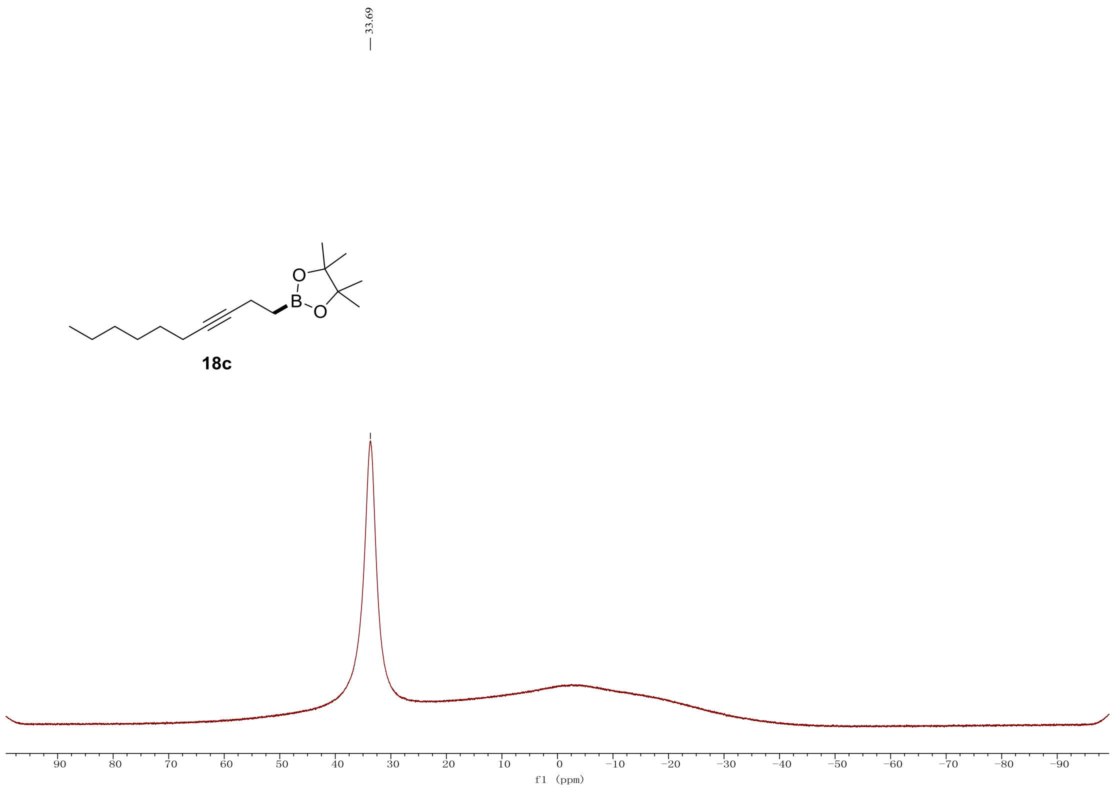

Supplementary Figure 172. ^{11}B NMR spectrum for 18c.

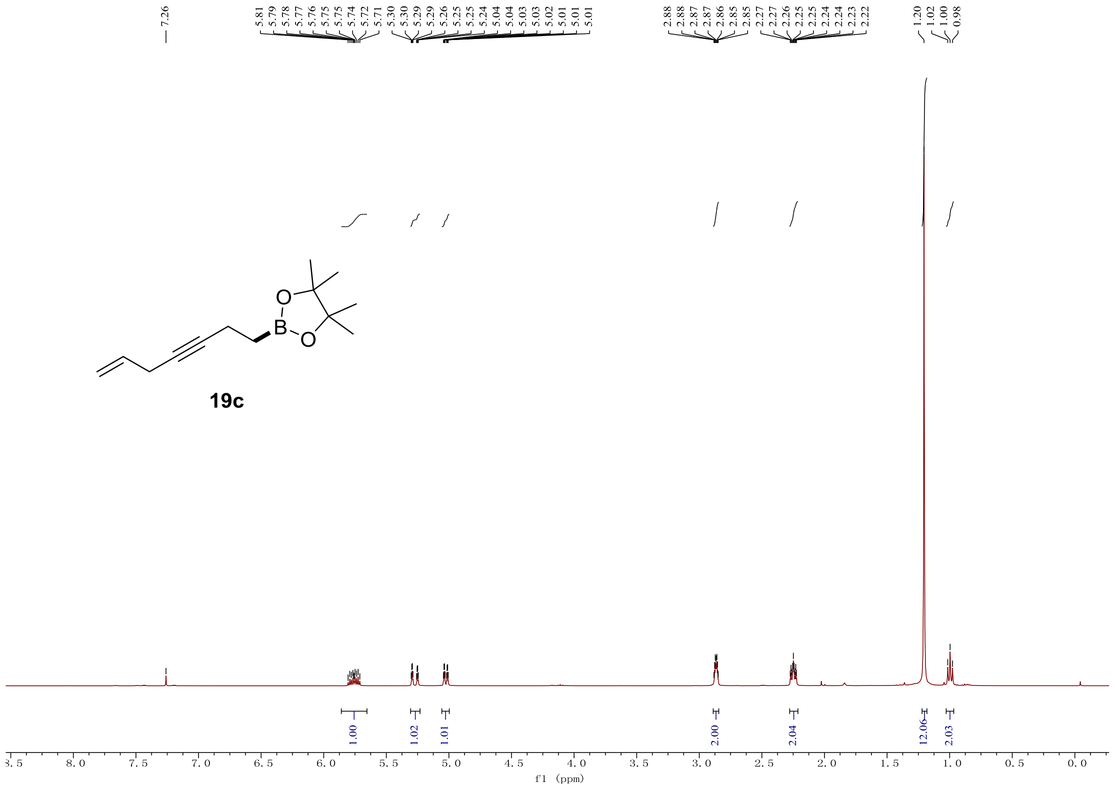

Supplementary Figure 173. ^{1}H NMR spectrum for 19c.

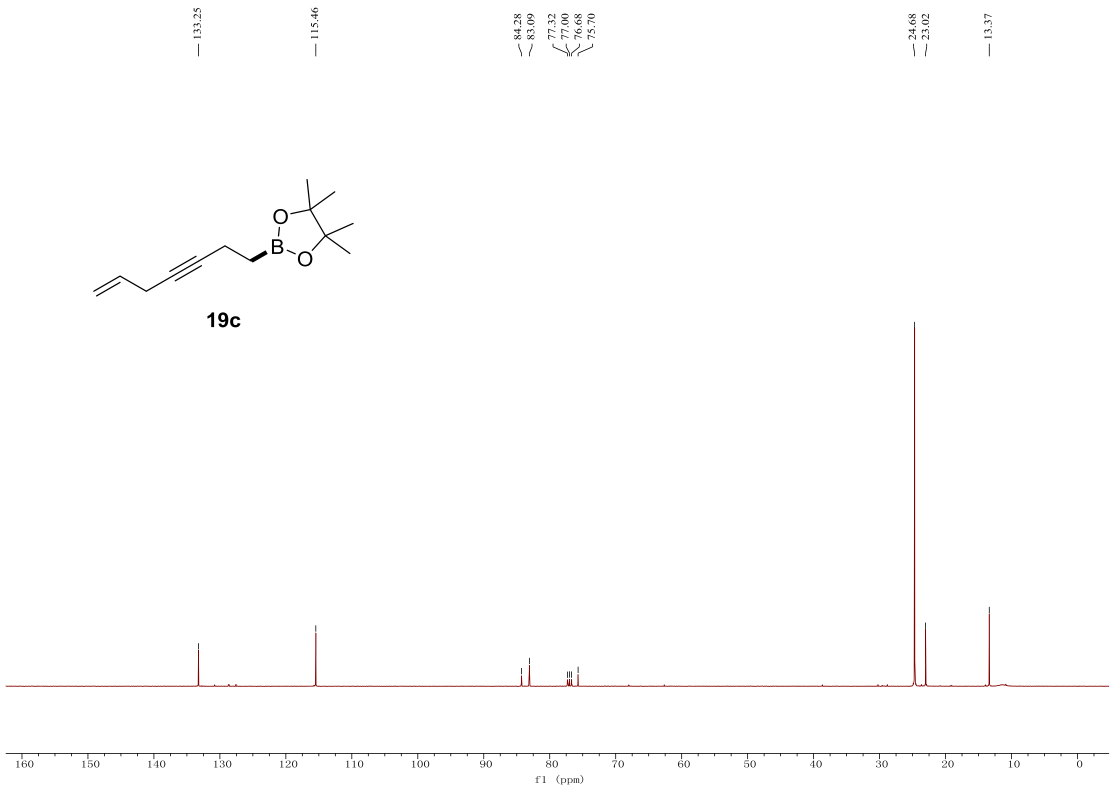

Supplementary Figure 174. ^{13}**C NMR spectrum for 19c.**

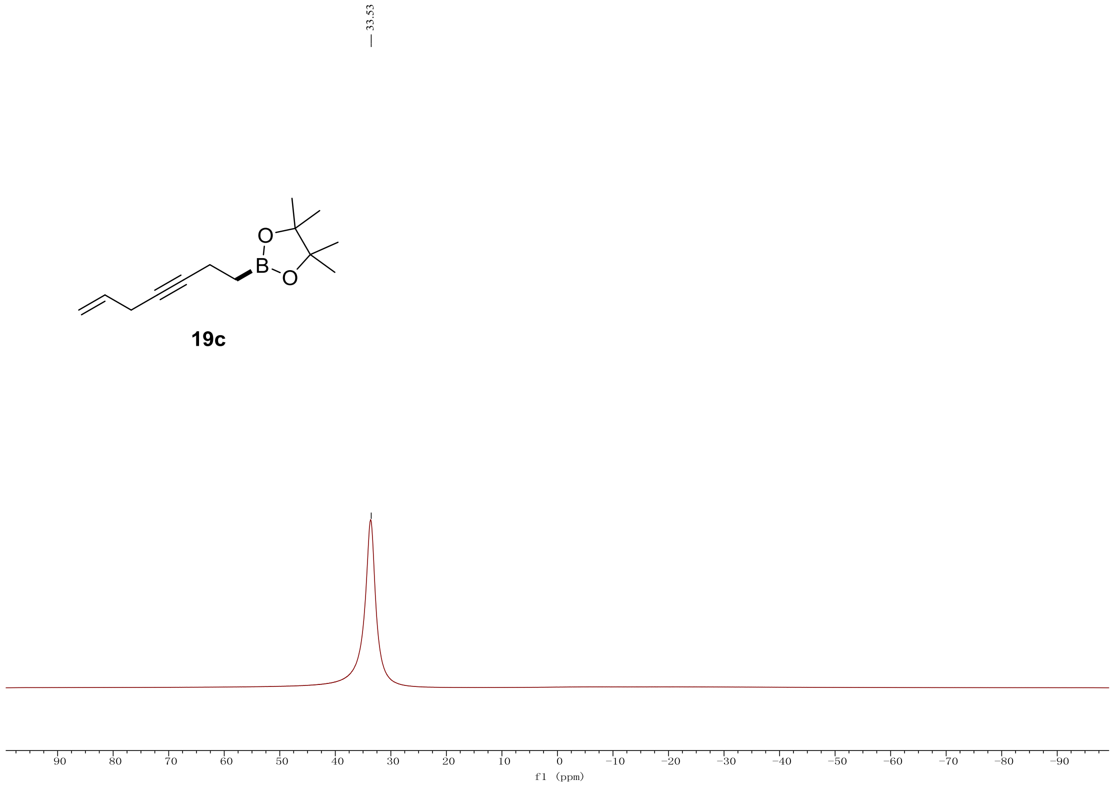

Supplementary Figure 175. ^{11}**B NMR spectrum for 19c.**

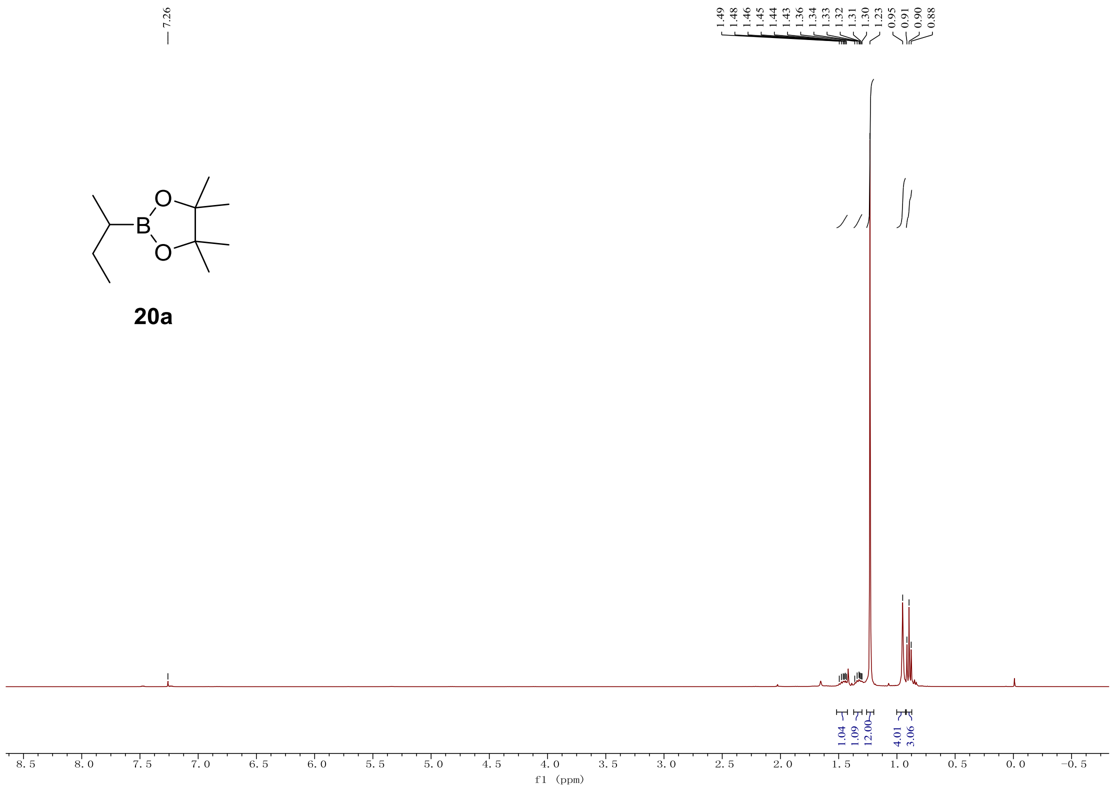

Supplementary Figure 176. ^{1}H NMR spectrum for 20a.

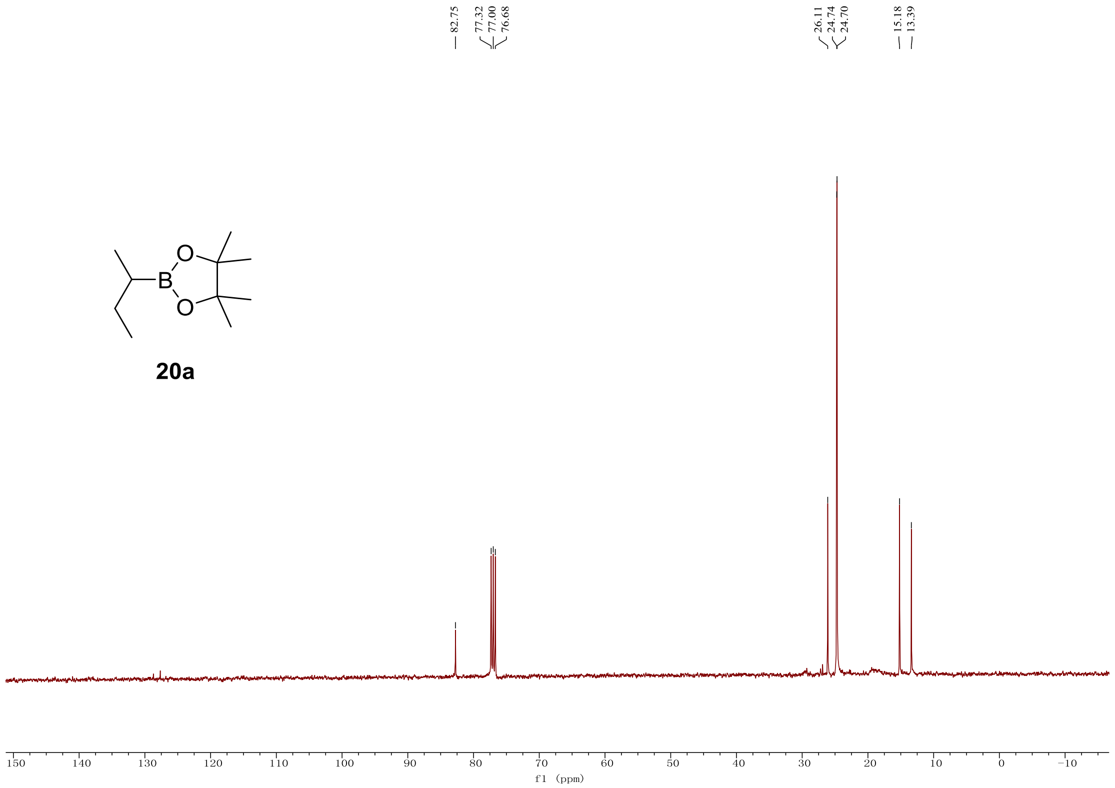

Supplementary Figure 177. ^{13}C NMR spectrum for 20a.

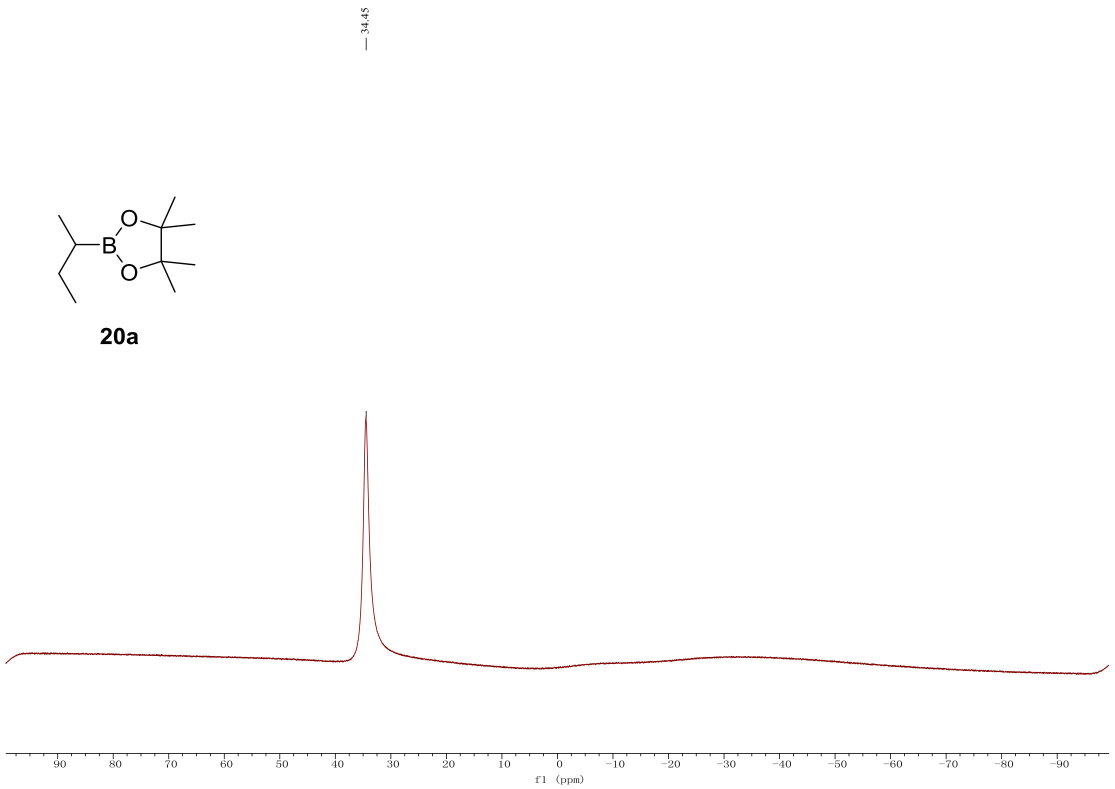

Supplementary Figure 178. ^{11}**B NMR spectrum for 20a.**

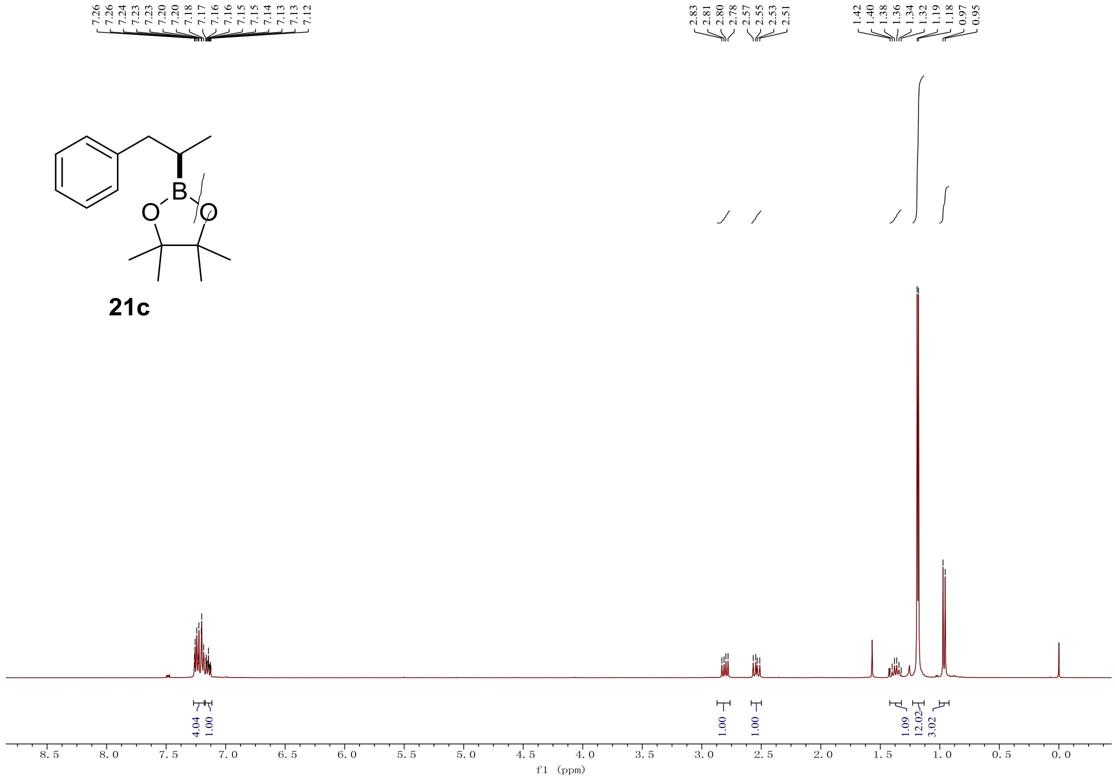

Supplementary Figure 179. ^{1}**H NMR spectrum for 21c.**

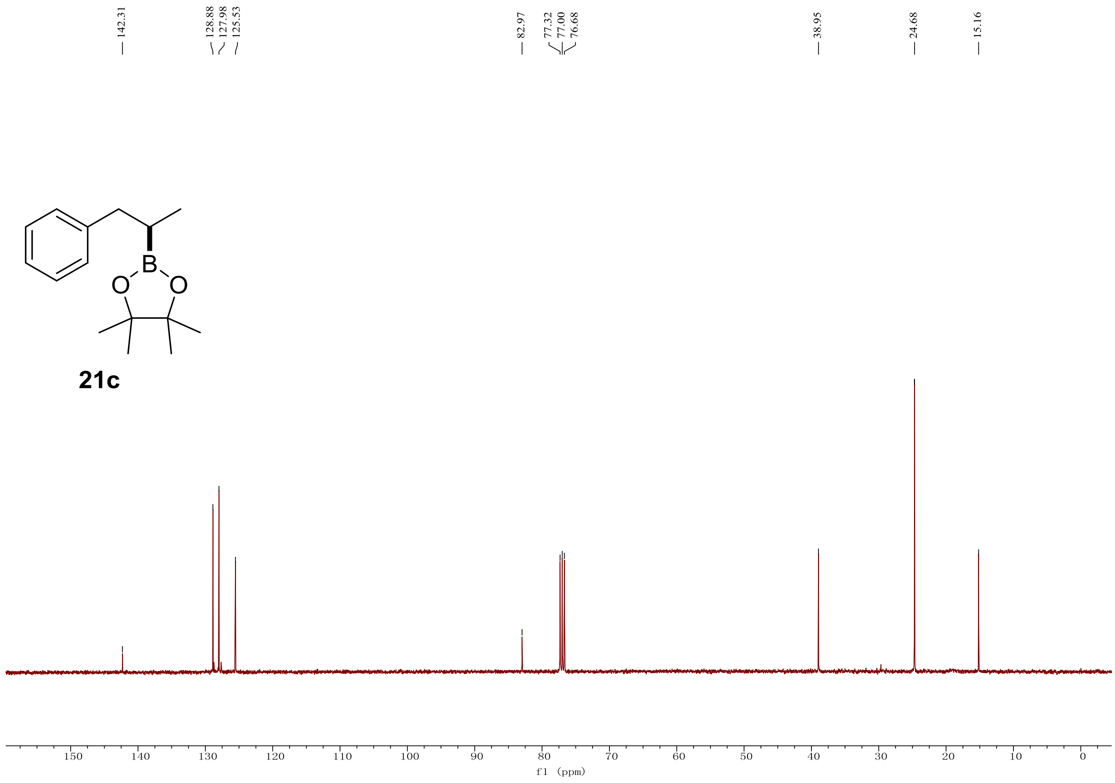

Supplementary Figure 180. ^{13}**C NMR spectrum for 21c.**

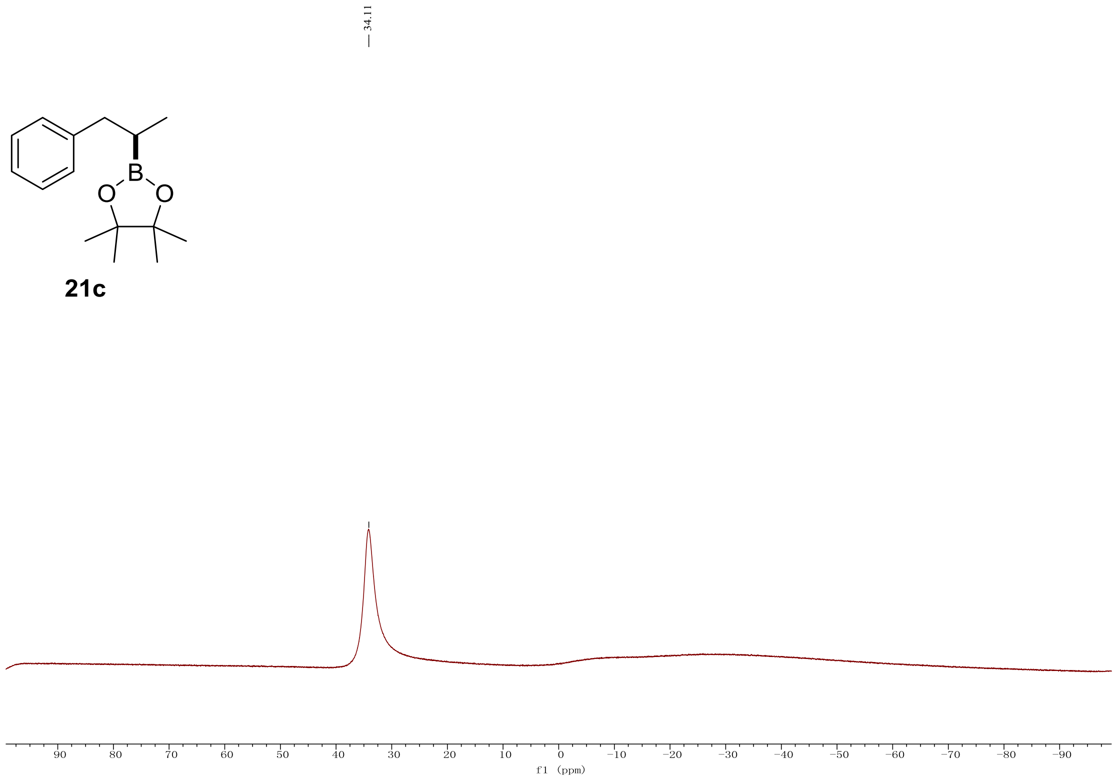

Supplementary Figure 181. ^{11}**B NMR spectrum for 21c.**

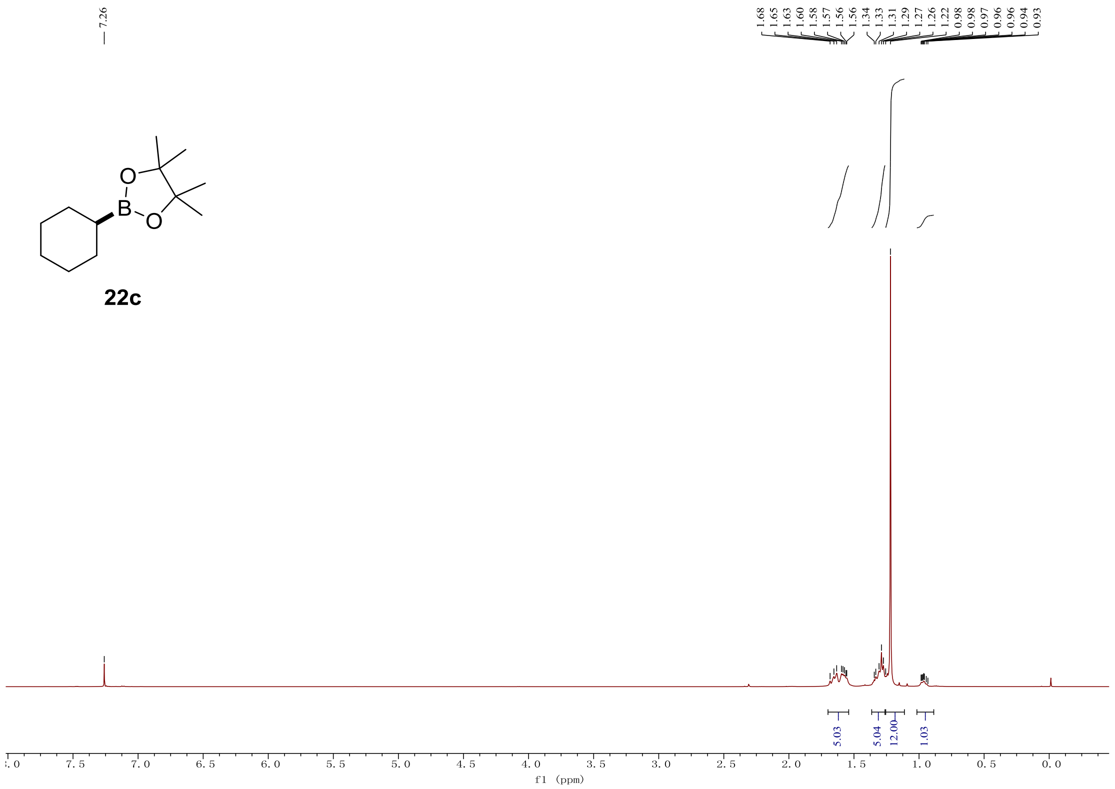

Supplementary Figure 182. ^{1}H NMR spectrum for 22c.

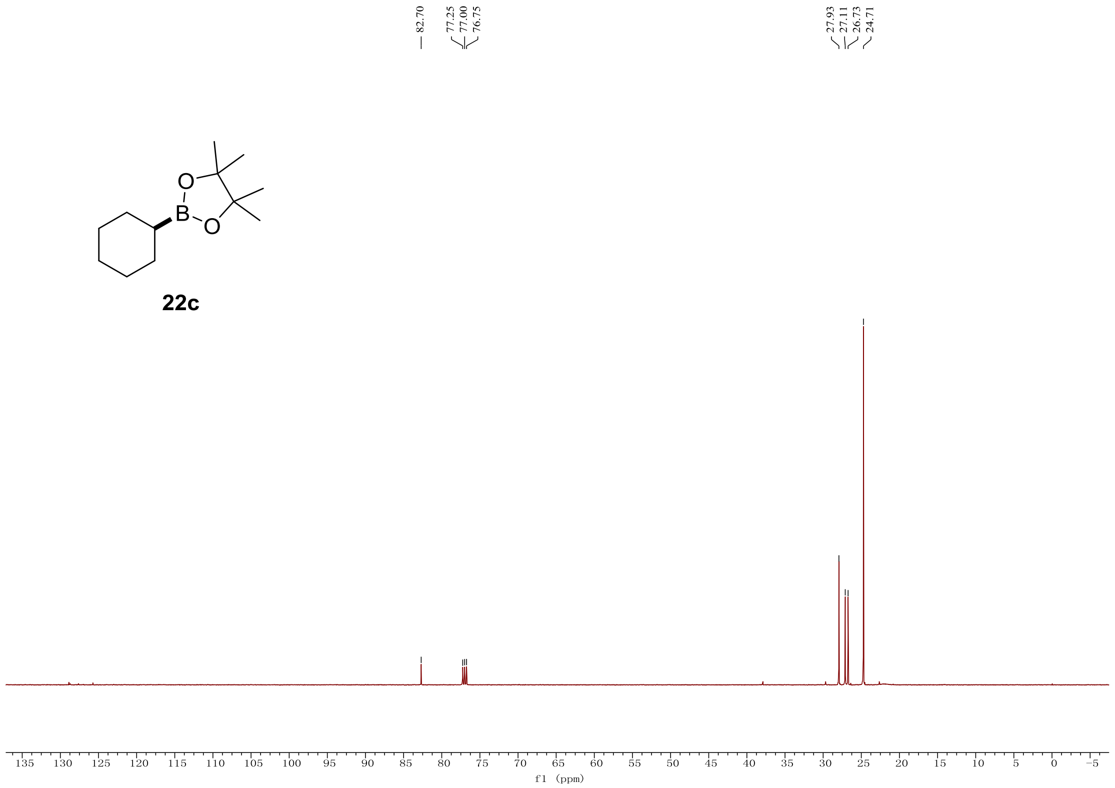

Supplementary Figure 183. ^{13}C NMR spectrum for 22c.

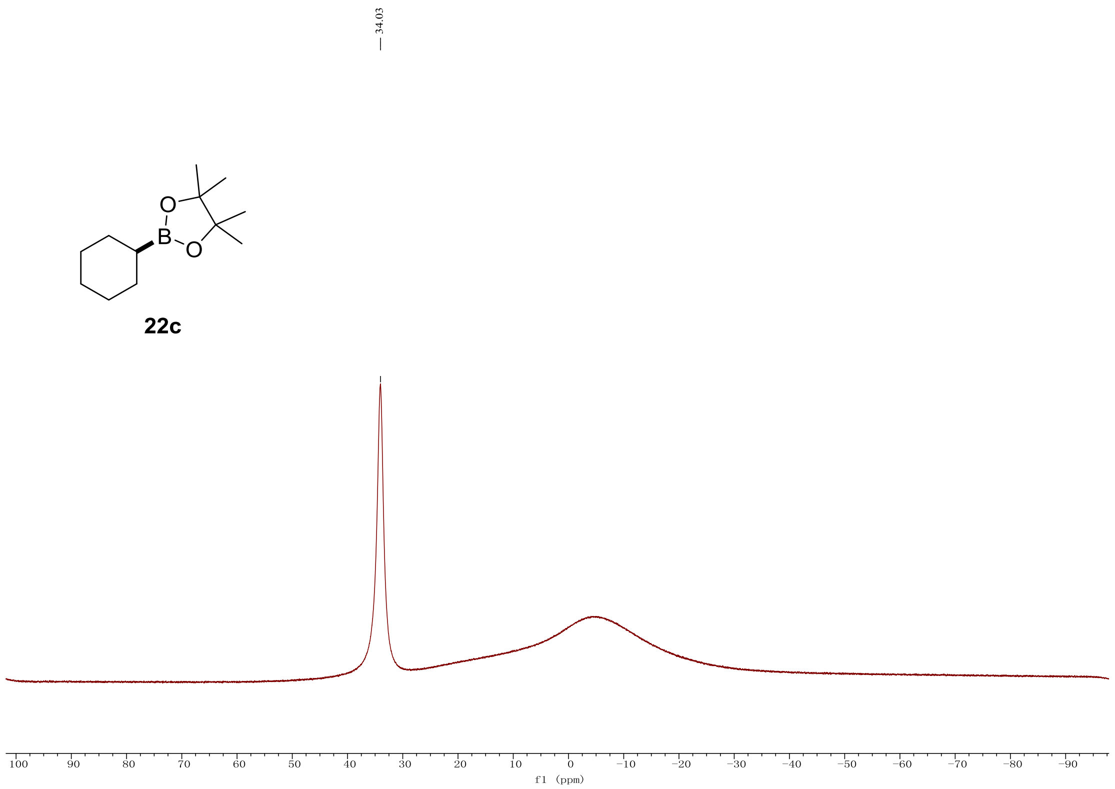

Supplementary Figure 184. ^{11}B NMR spectrum for 22c.

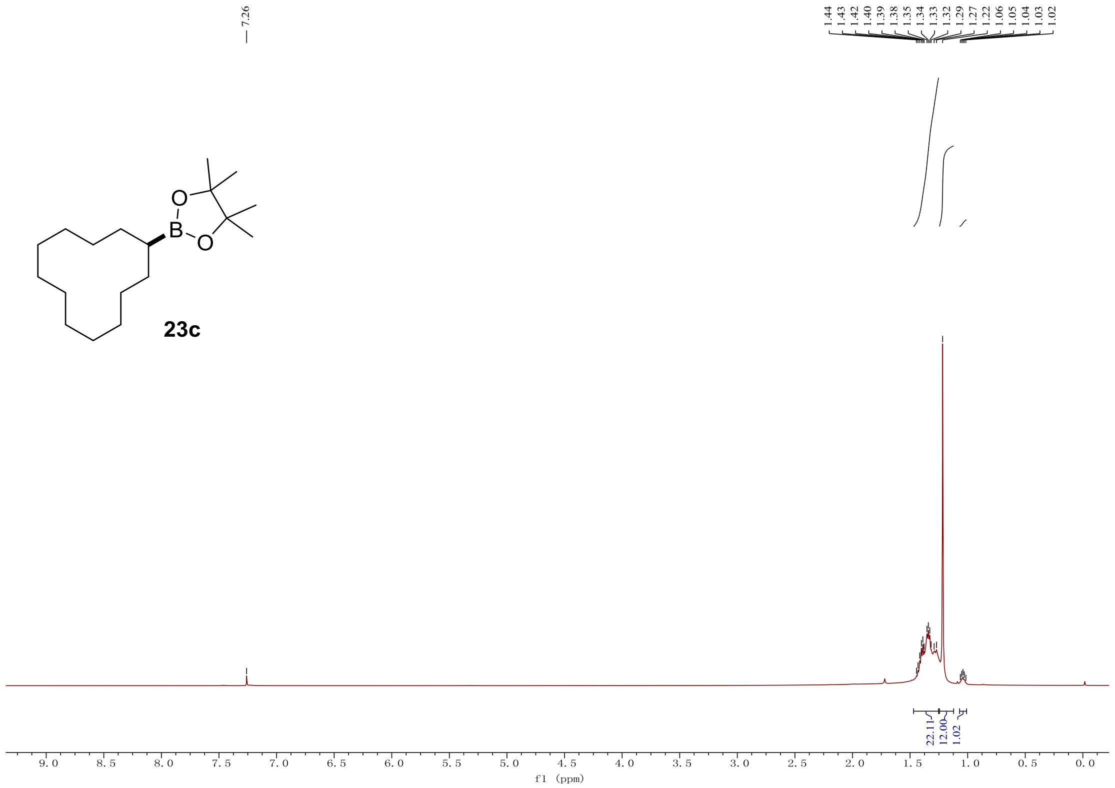

Supplementary Figure 185. ^{1}H NMR spectrum for 23c.

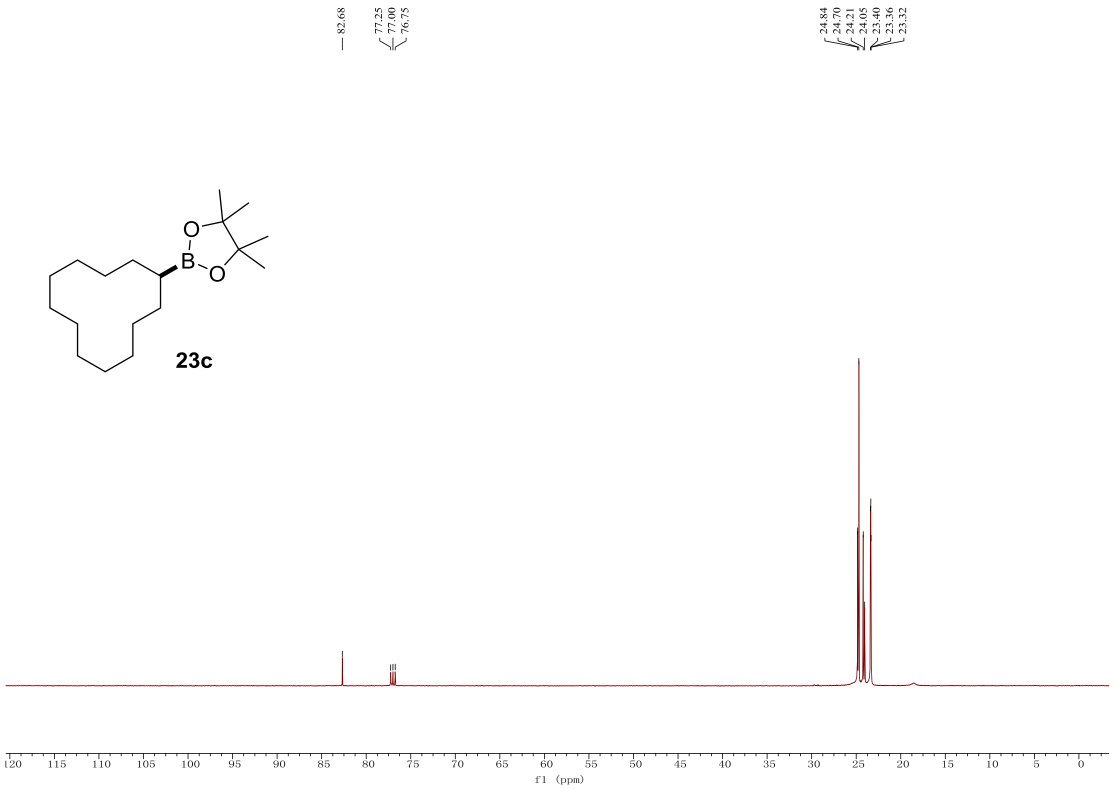

Supplementary Figure 186. ^{13}**C NMR spectrum for 23c.**

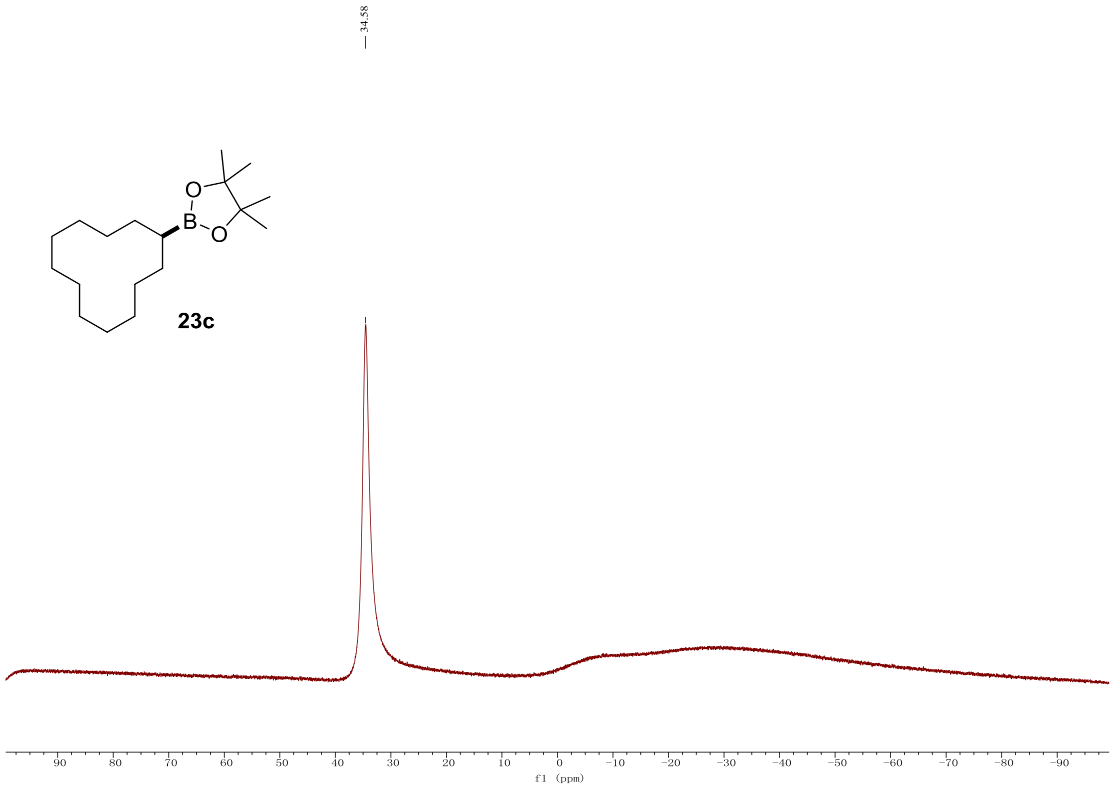

Supplementary Figure 187. ^{11}**B NMR spectrum for 23c.**

)

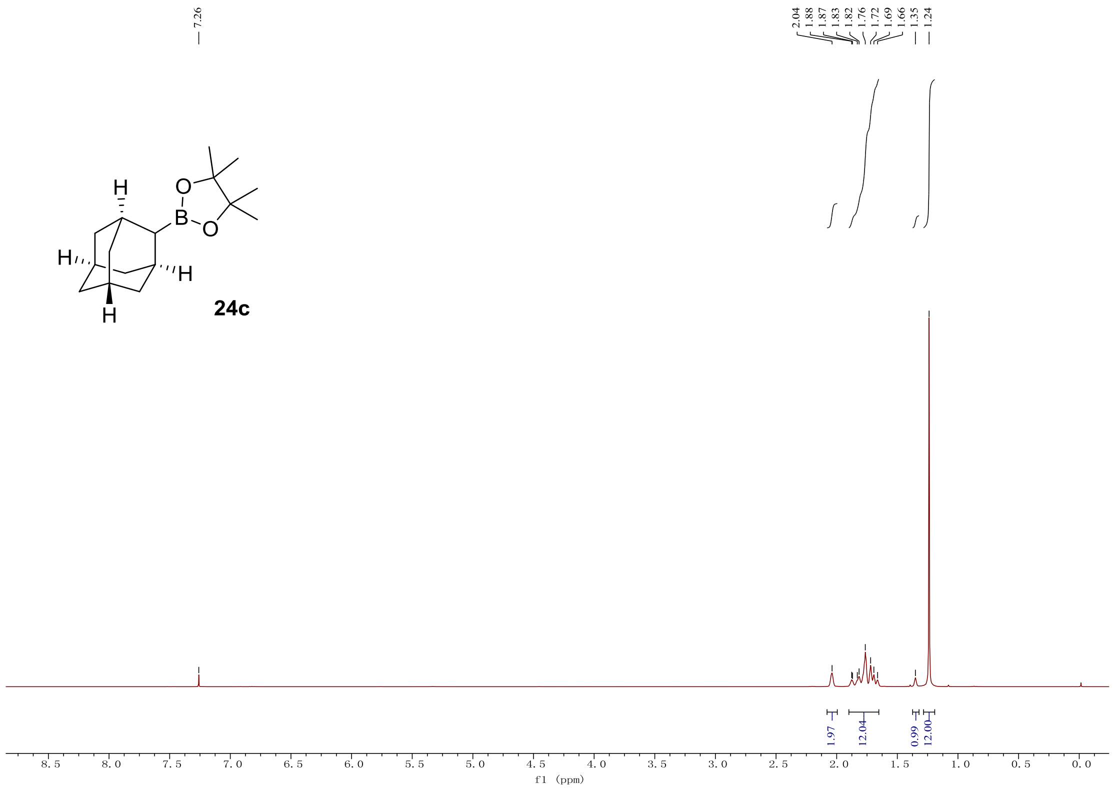

Supplementary Figure 188. ^{1}H NMR spectrum for 24c.

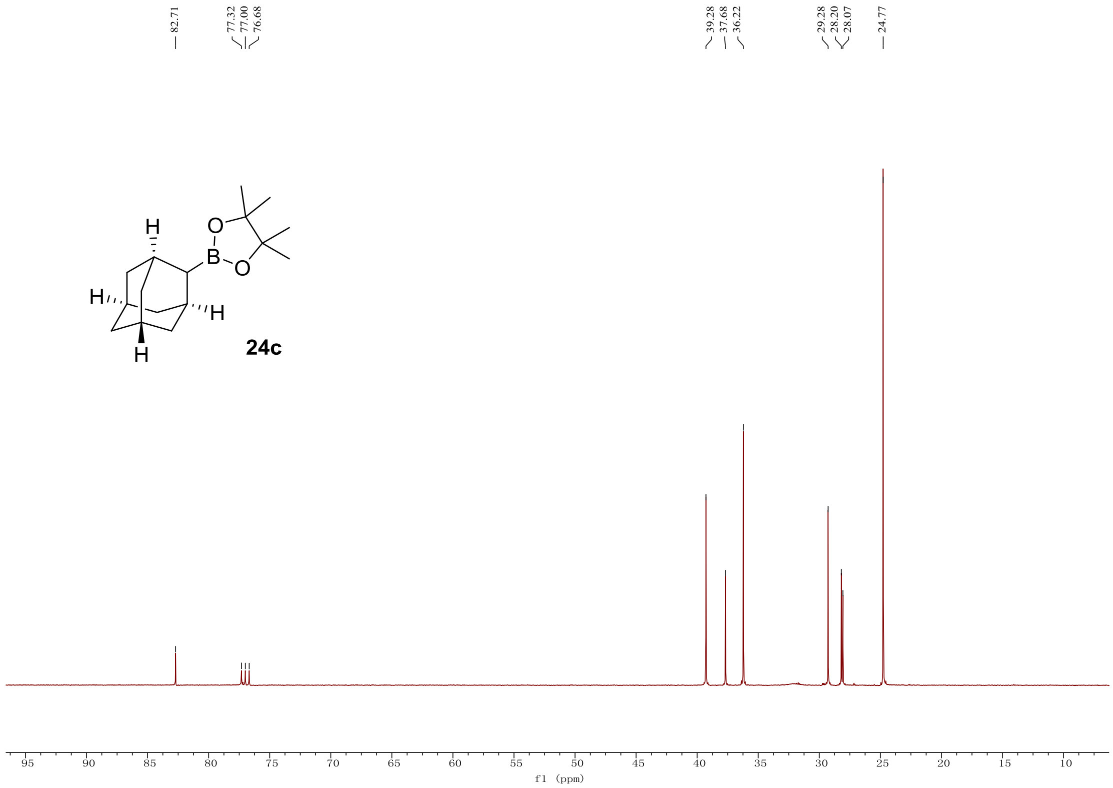

Supplementary Figure 189. ^{13}C NMR spectrum for 24c.

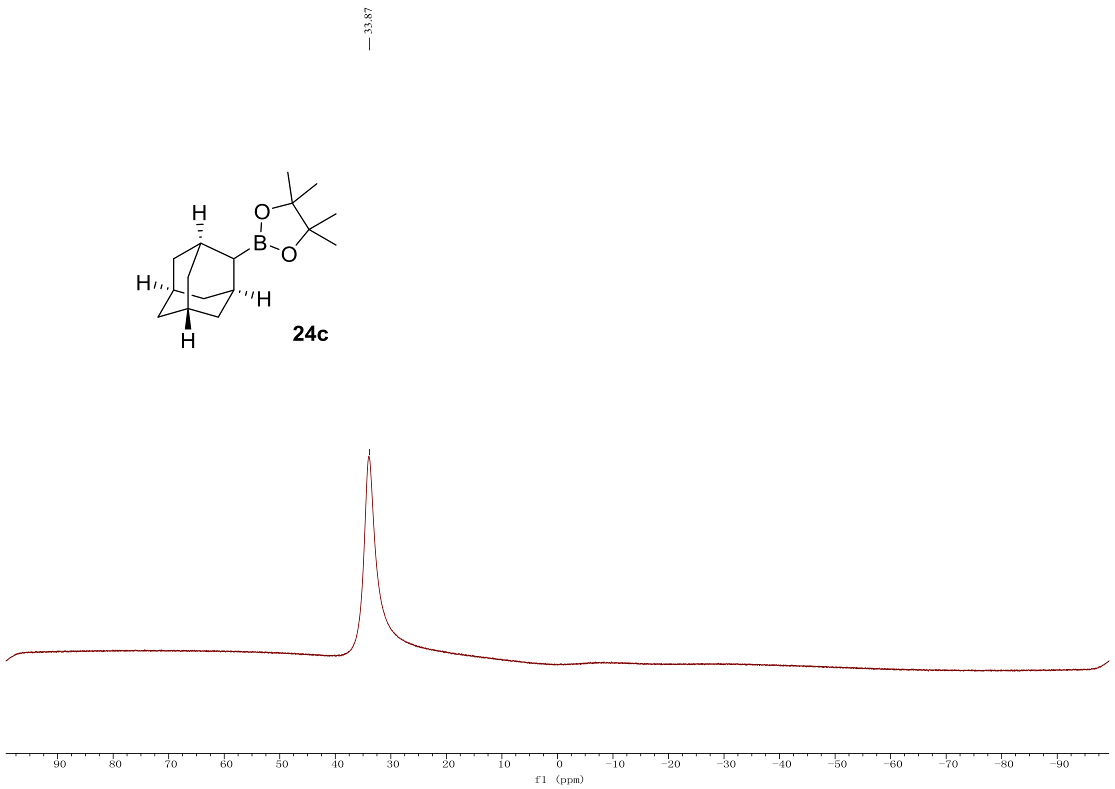

Supplementary Figure 190. ^{11}B NMR spectrum for 24c.

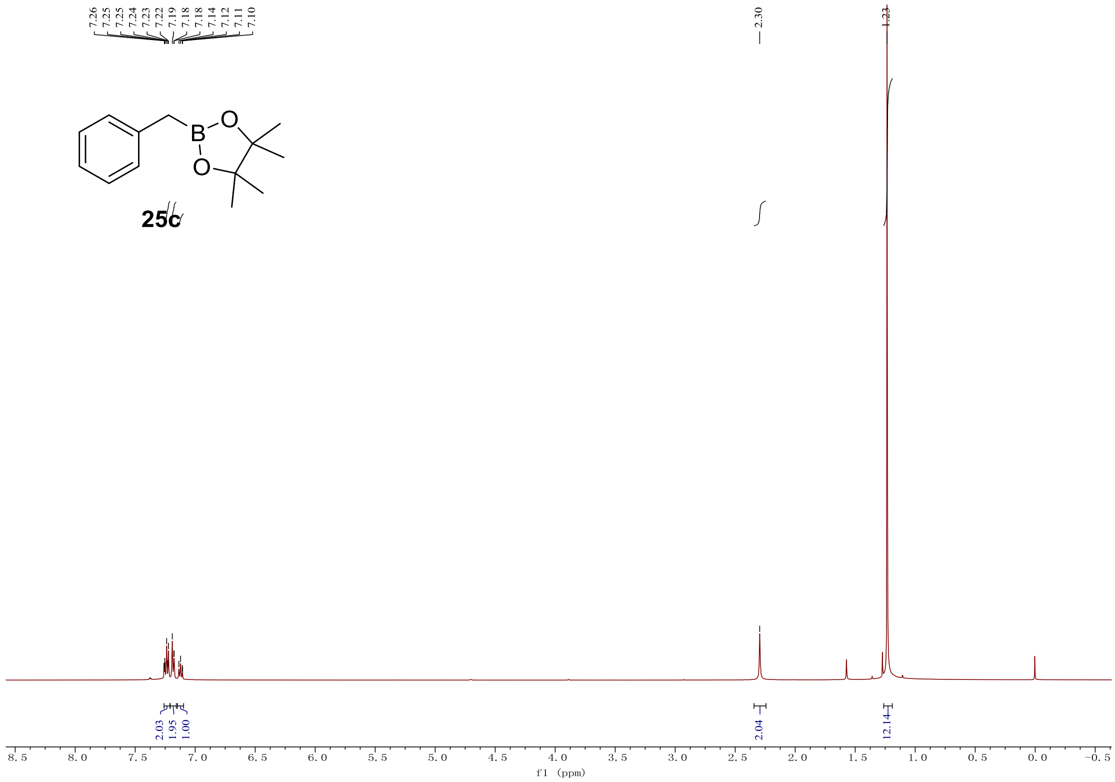

Supplementary Figure 191. ^{1}H NMR spectrum for 25c.

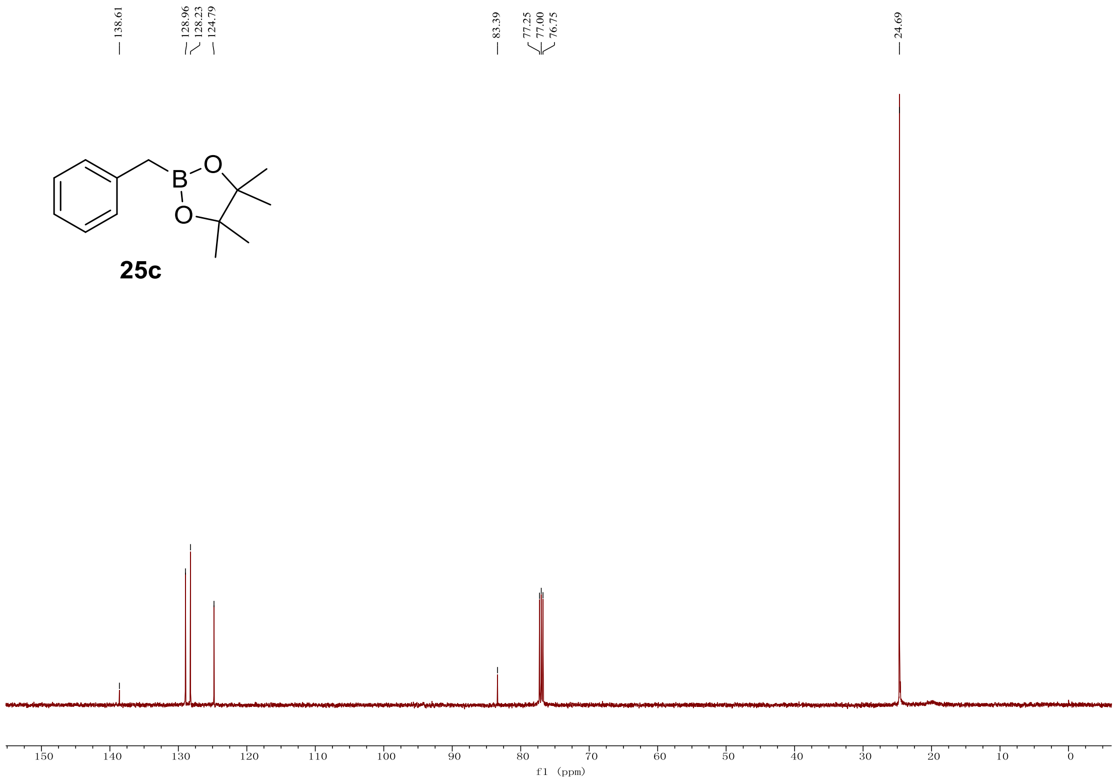

Supplementary Figure 192. ^{13}C NMR spectrum for 25c

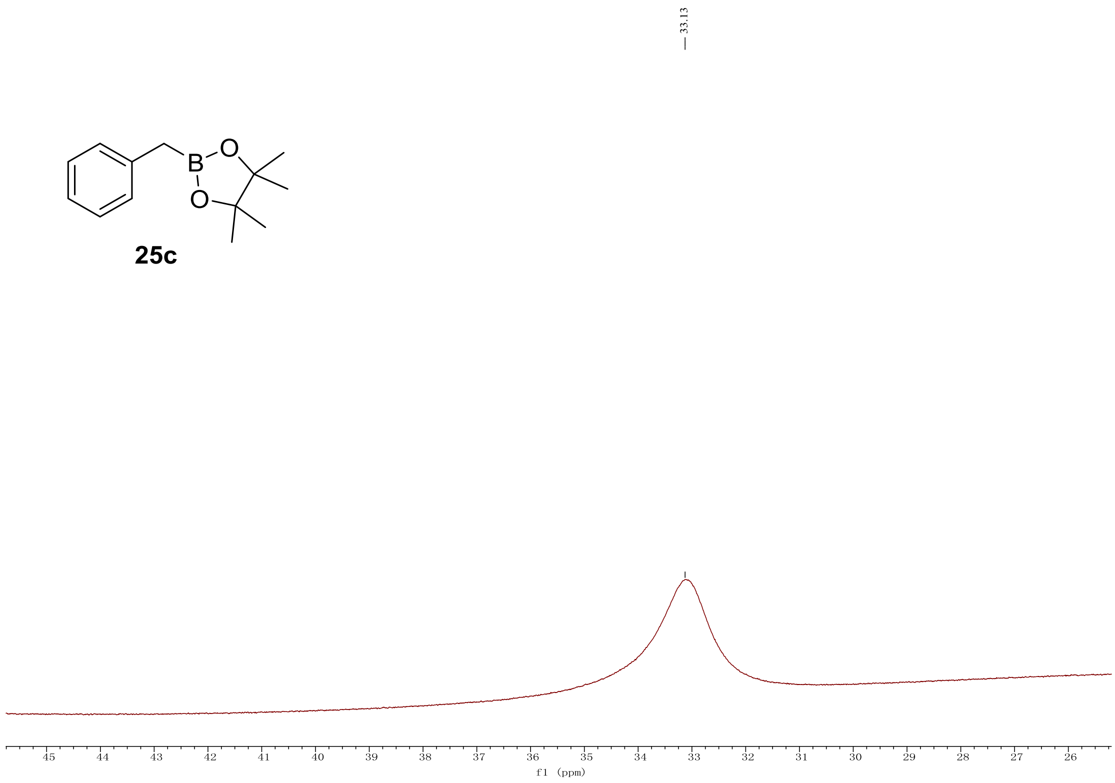

Supplementary Figure 193. ^{11}B NMR spectrum for 26c.

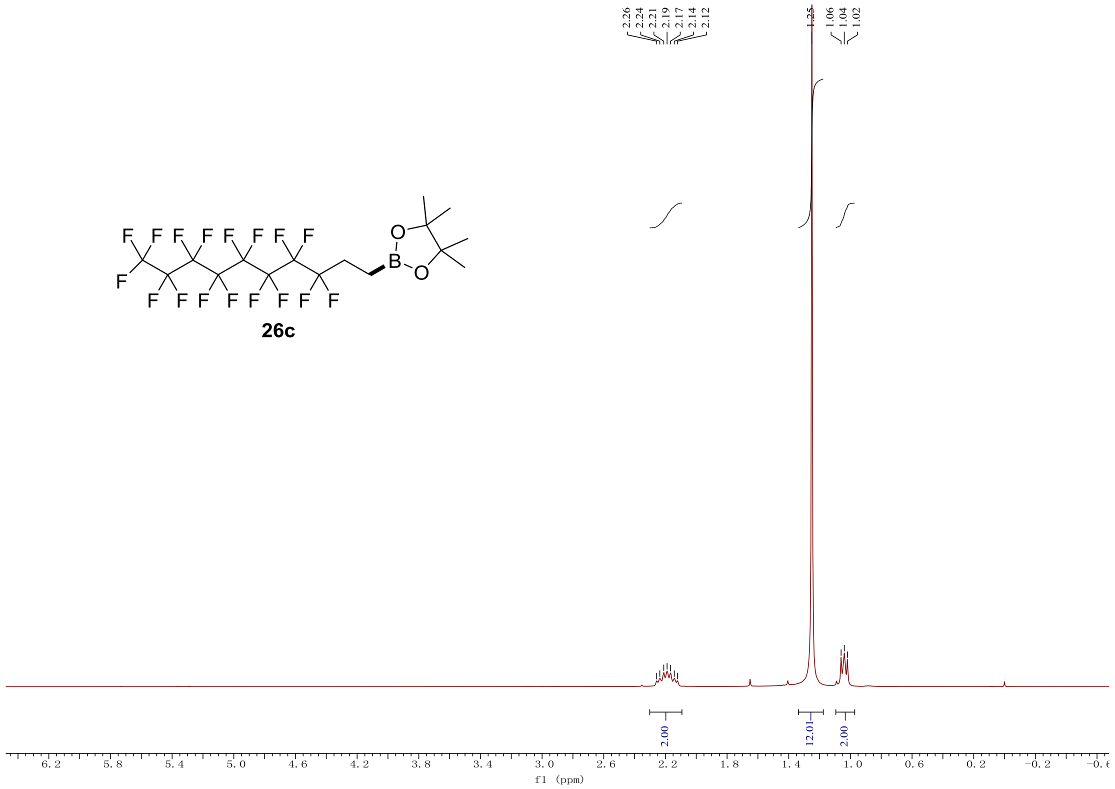

Supplementary Figure 194. ^{1}H NMR spectrum for **26c.**

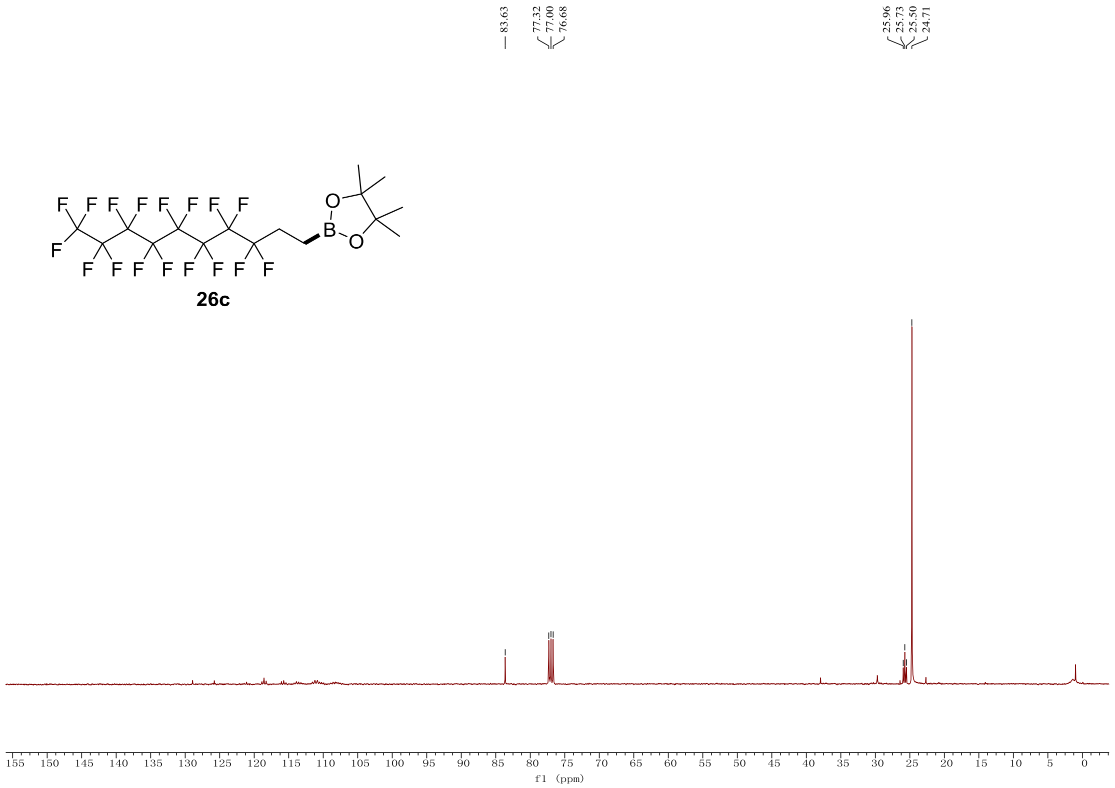

Supplementary Figure 195. ^{13}C NMR spectrum for **26c**

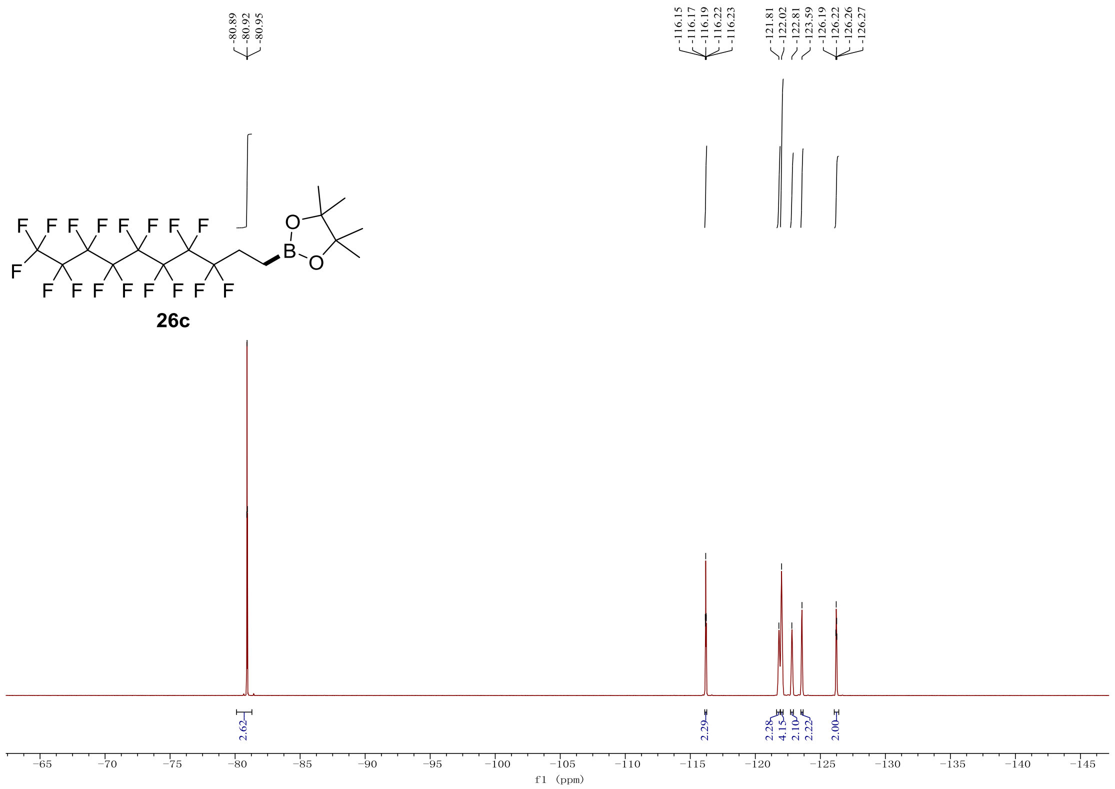

Supplementary Figure 196. ^{19}F NMR spectrum for **26c.**

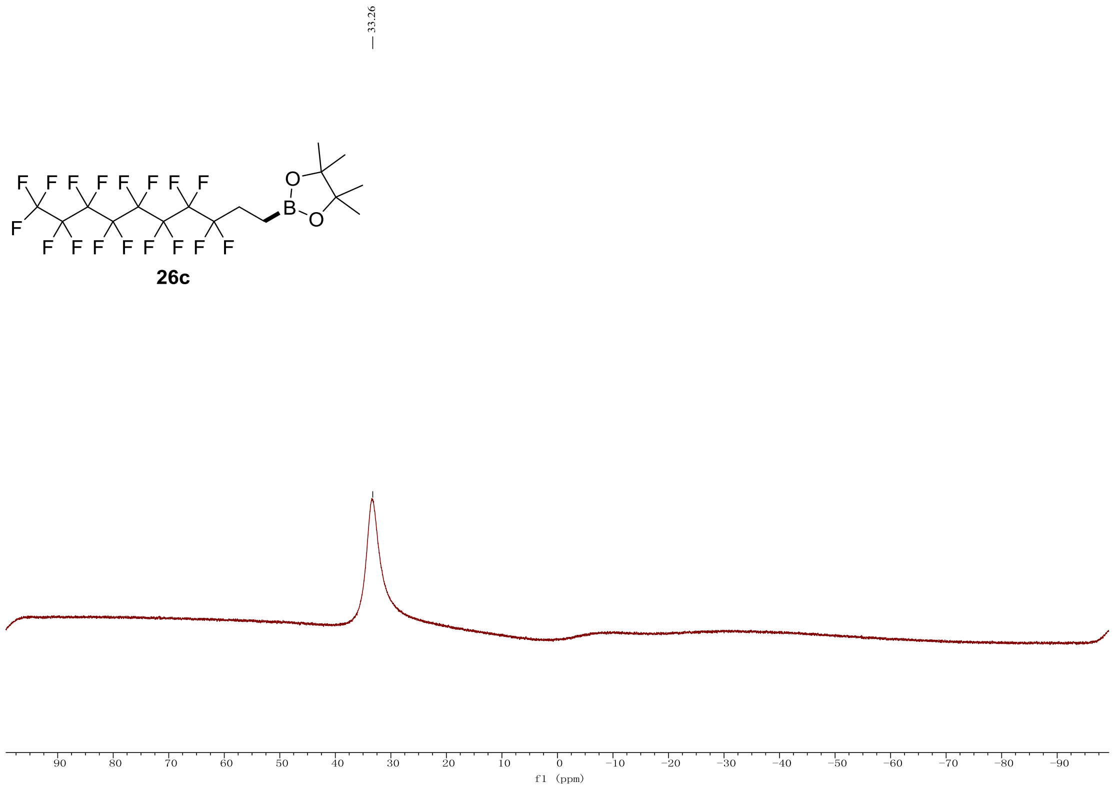

Supplementary Figure 197. ^{11}B NMR spectrum for **26c.**

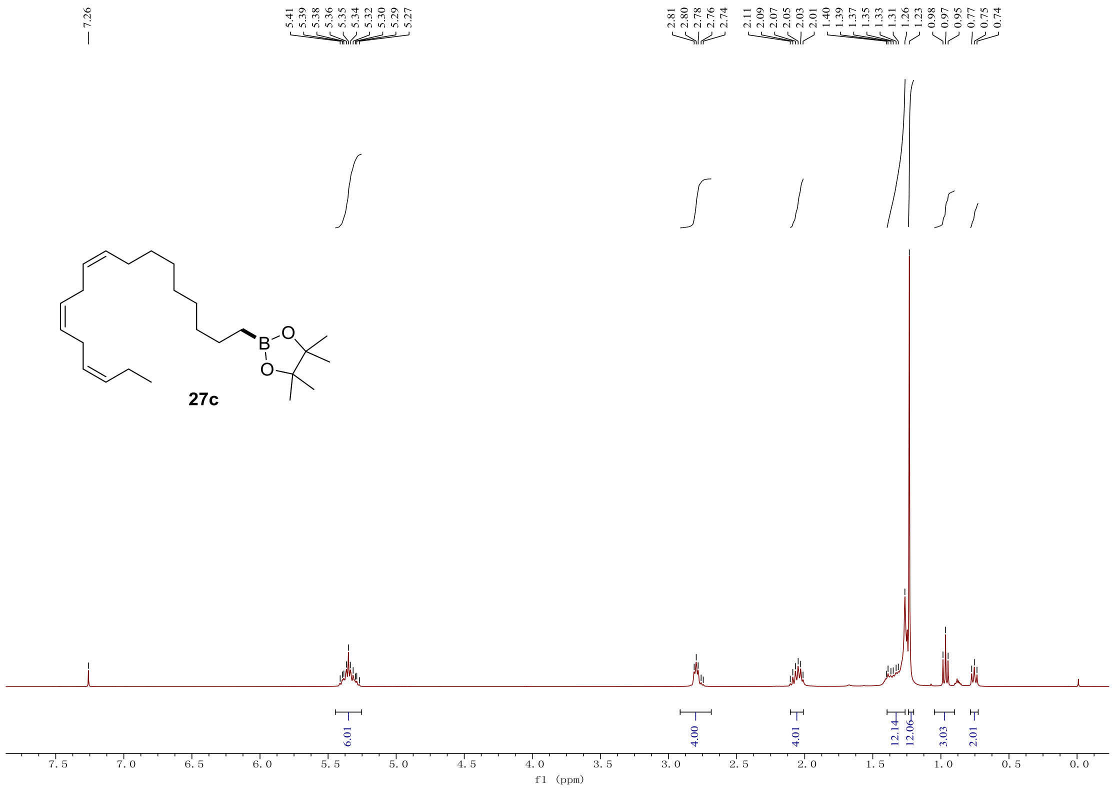

Supplementary Figure 198. ^{1}H NMR spectrum for **27c.**

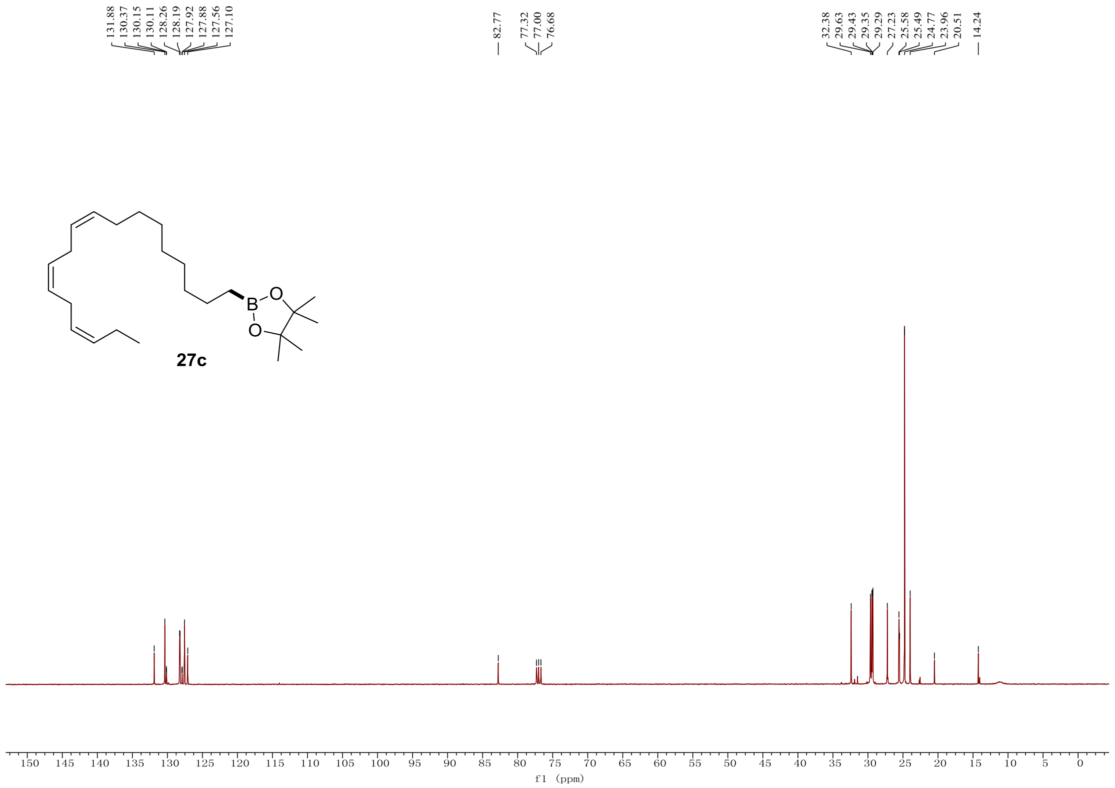

Supplementary Figure 199. ^{13}C NMR spectrum for **27c**

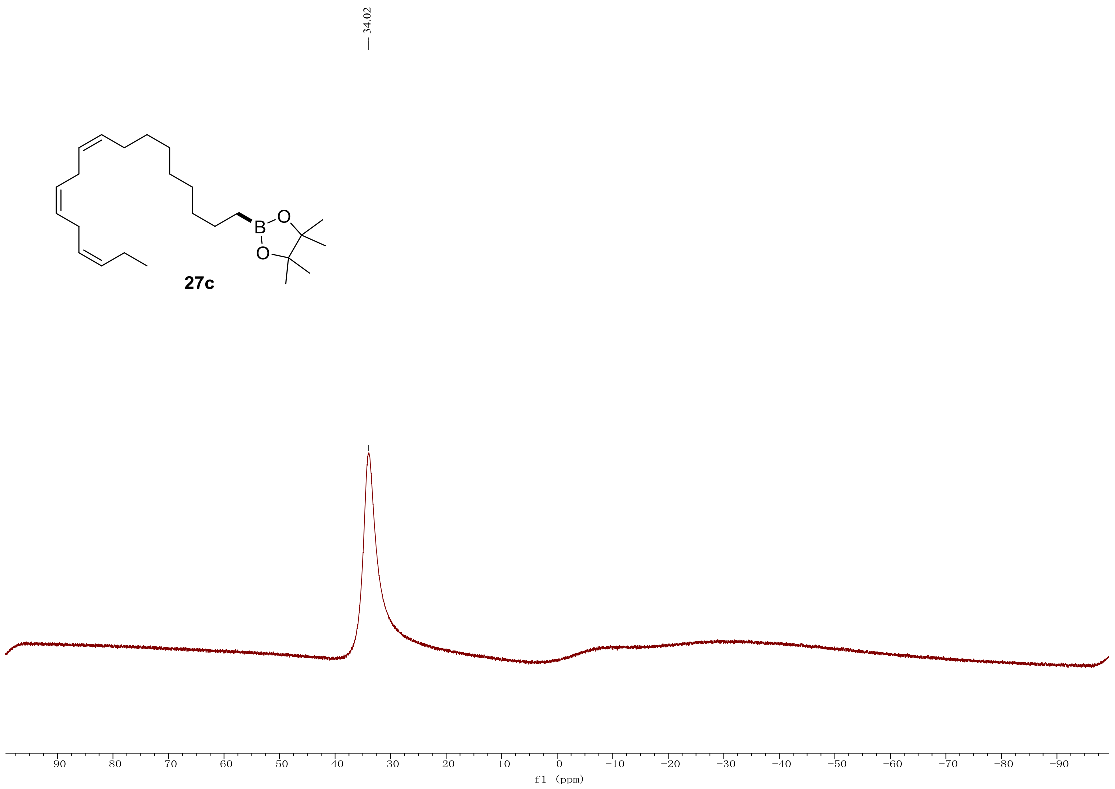

Supplementary Figure 200. ^{11}B NMR spectrum for 27c.

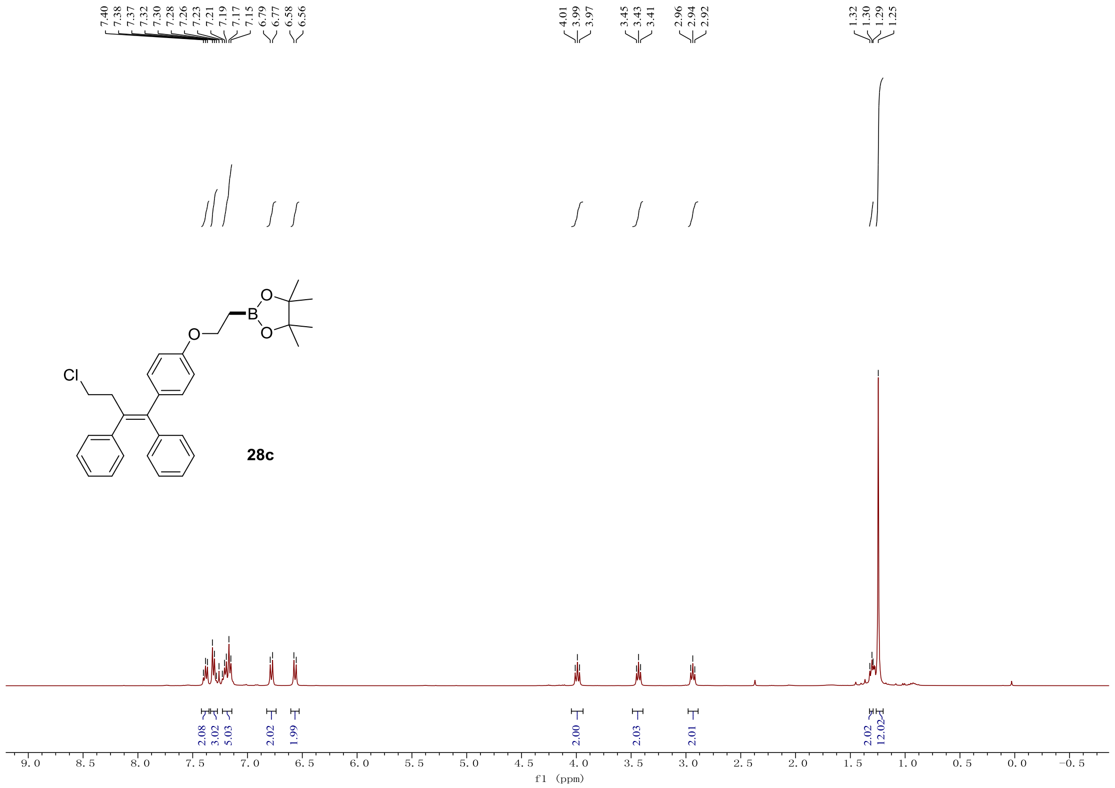

Supplementary Figure 201. ^{1}H NMR spectrum for 28c.

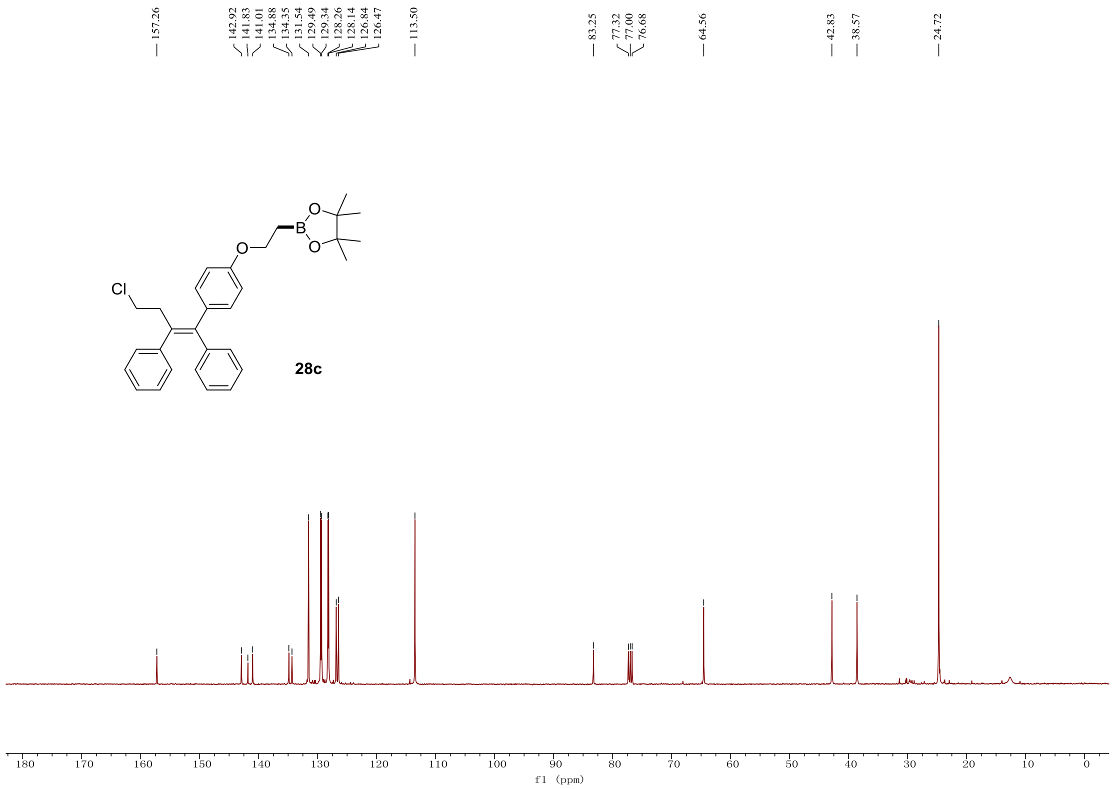

Supplementary Figure 202. ^{13}C NMR spectrum for 28c.

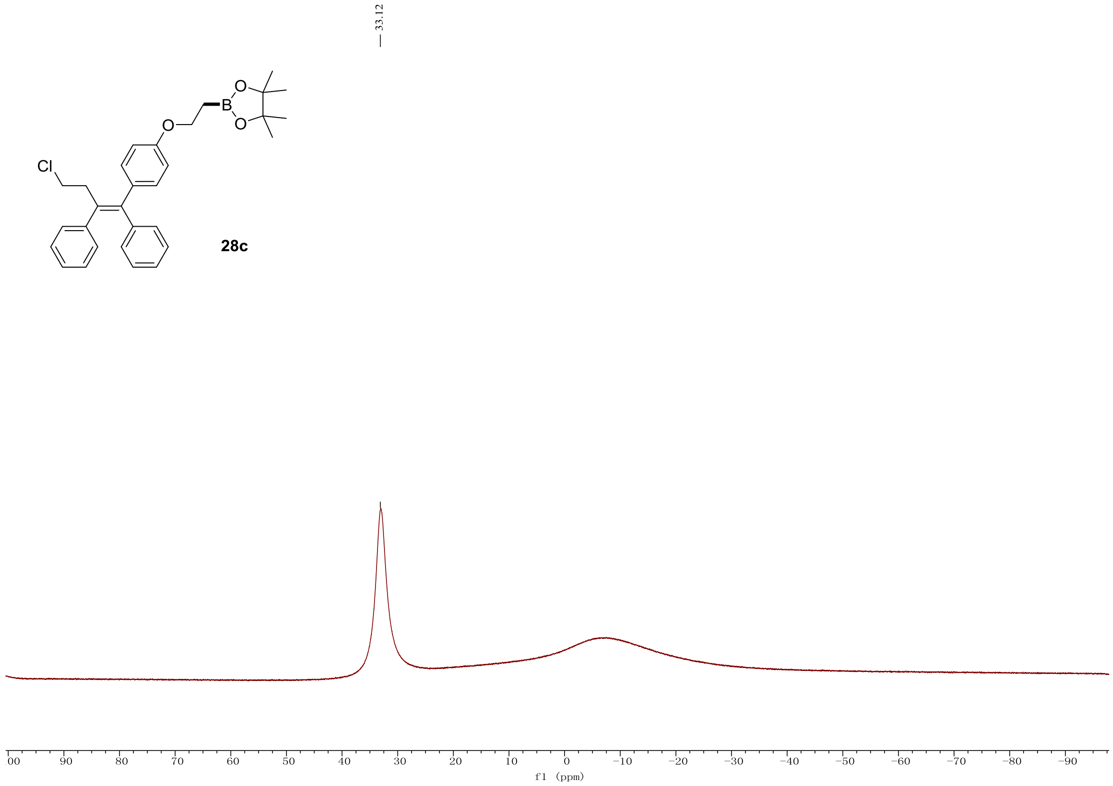

Supplementary Figure 203. ^{11}B NMR spectrum for 28c.

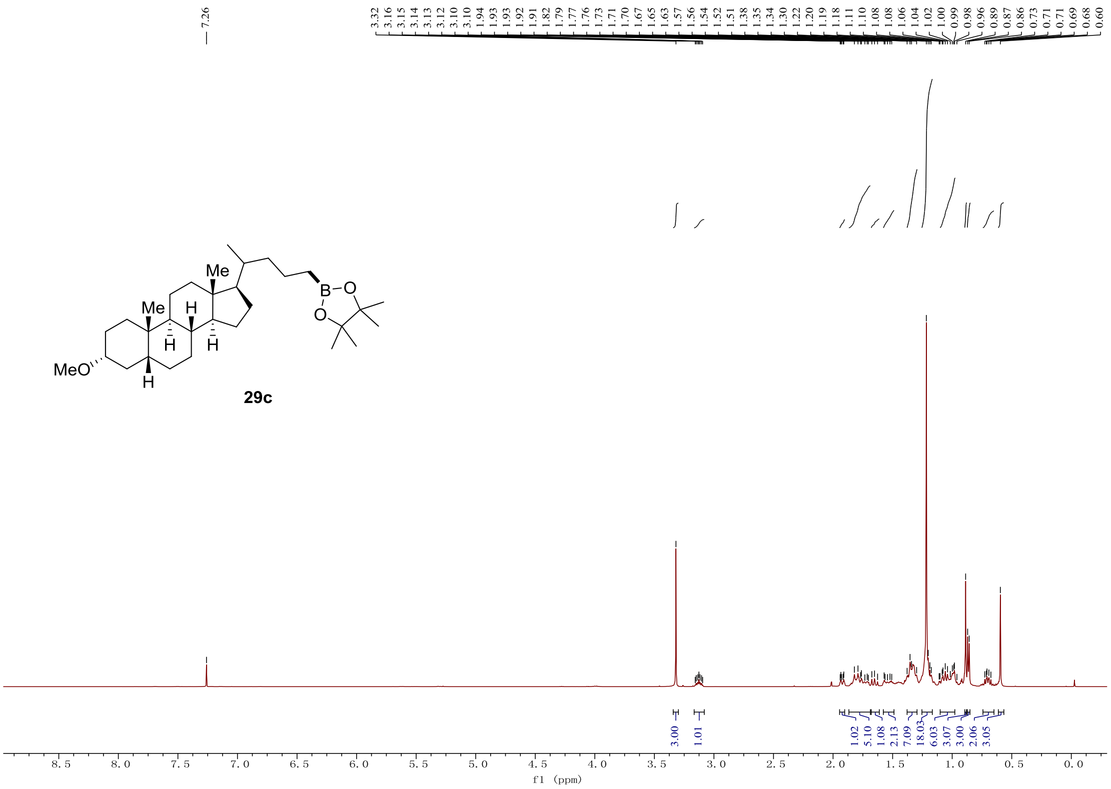

Supplementary Figure 204. ^{1}**H NMR spectrum for 29c.**

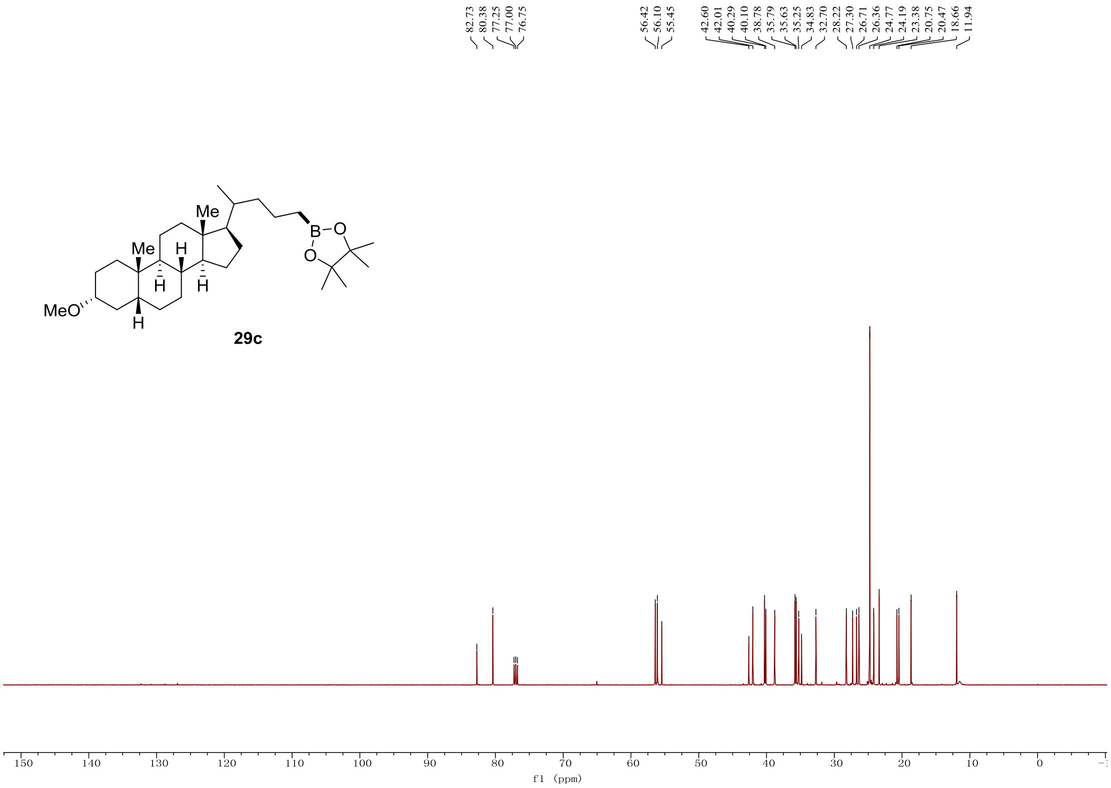

Supplementary Figure 205. ^{13}**C NMR spectrum for 29c.**

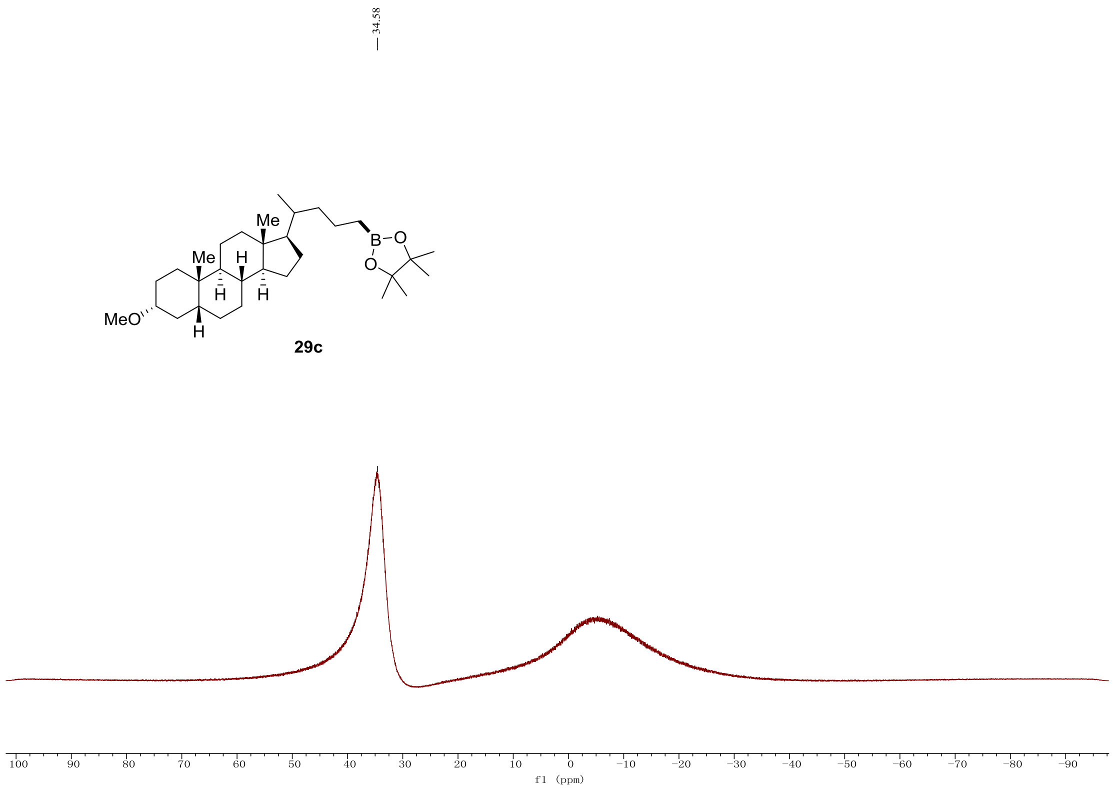

Supplementary Figure 206. ^{11}B NMR spectrum for 29c.

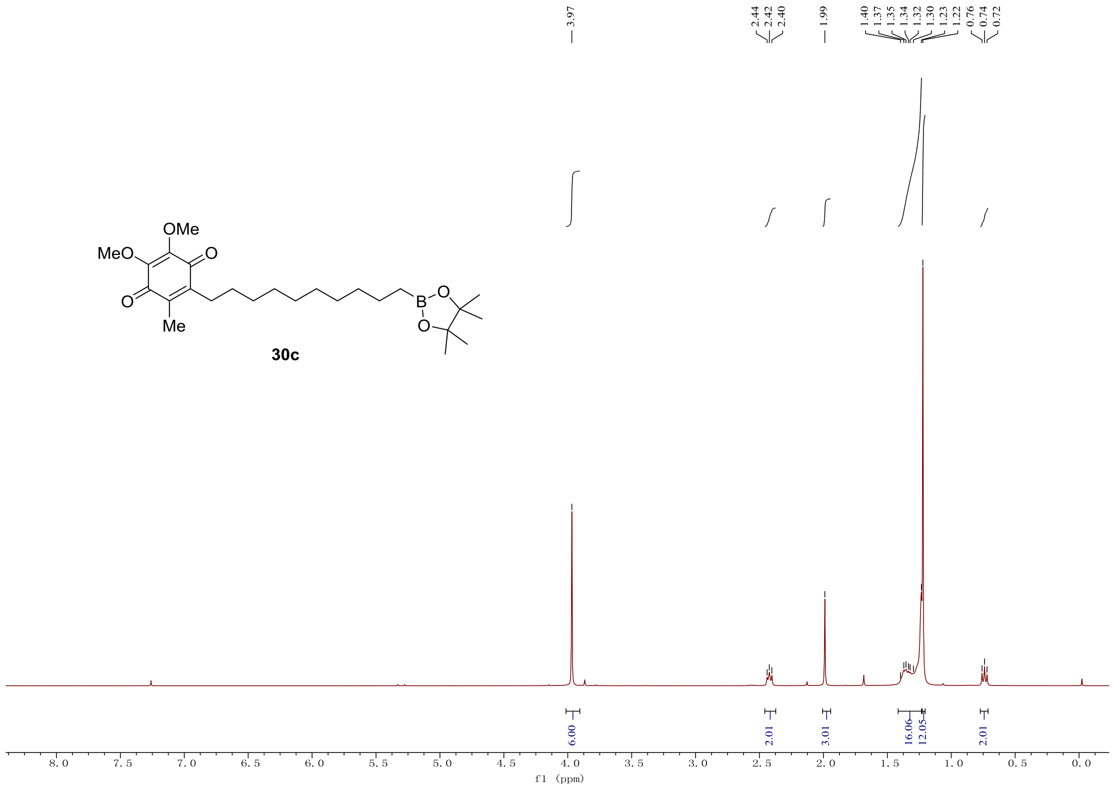

Supplementary Figure 207. ^{1}H NMR spectrum for 30c.

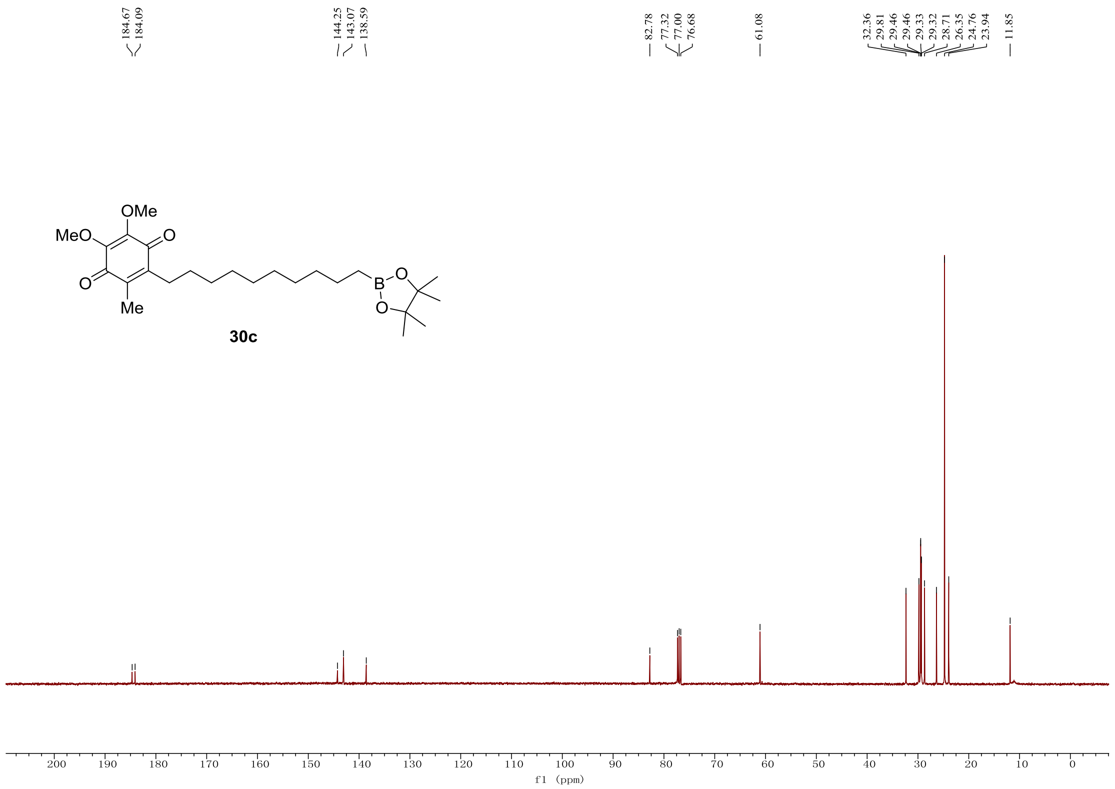

Supplementary Figure 208. ^{13}C NMR spectrum for 30c.

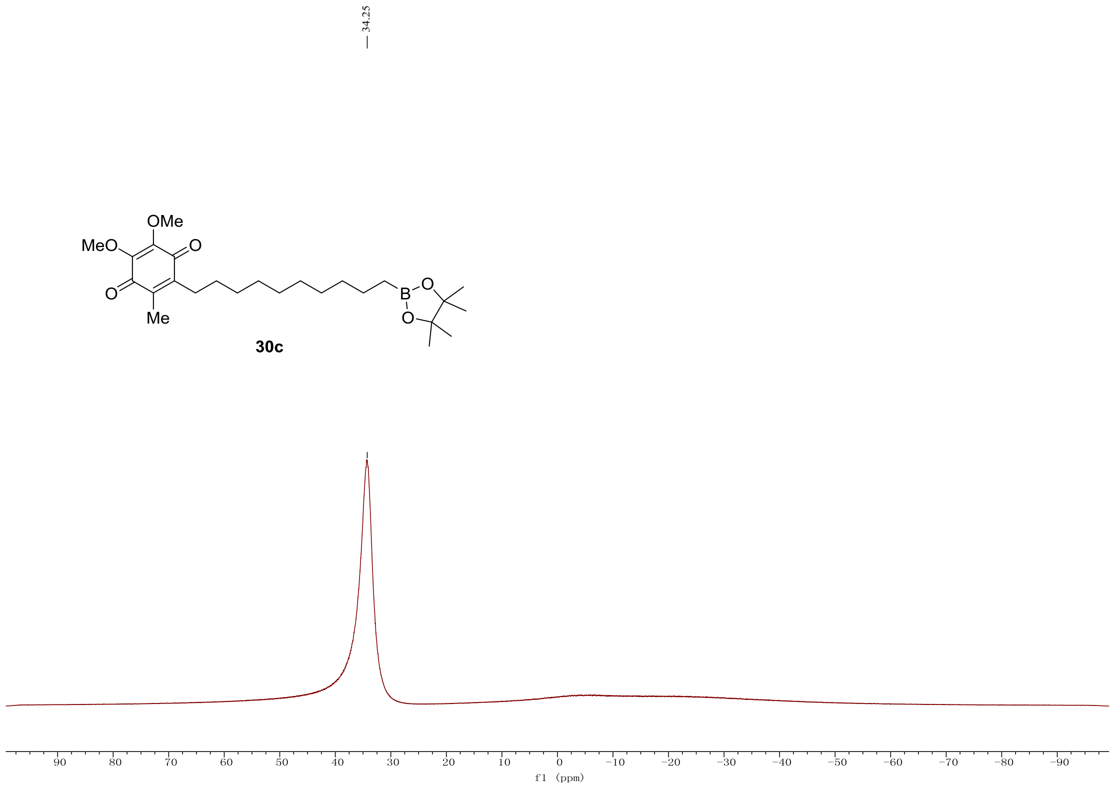

Supplementary Figure 209. ^{11}B NMR spectrum for 30c.

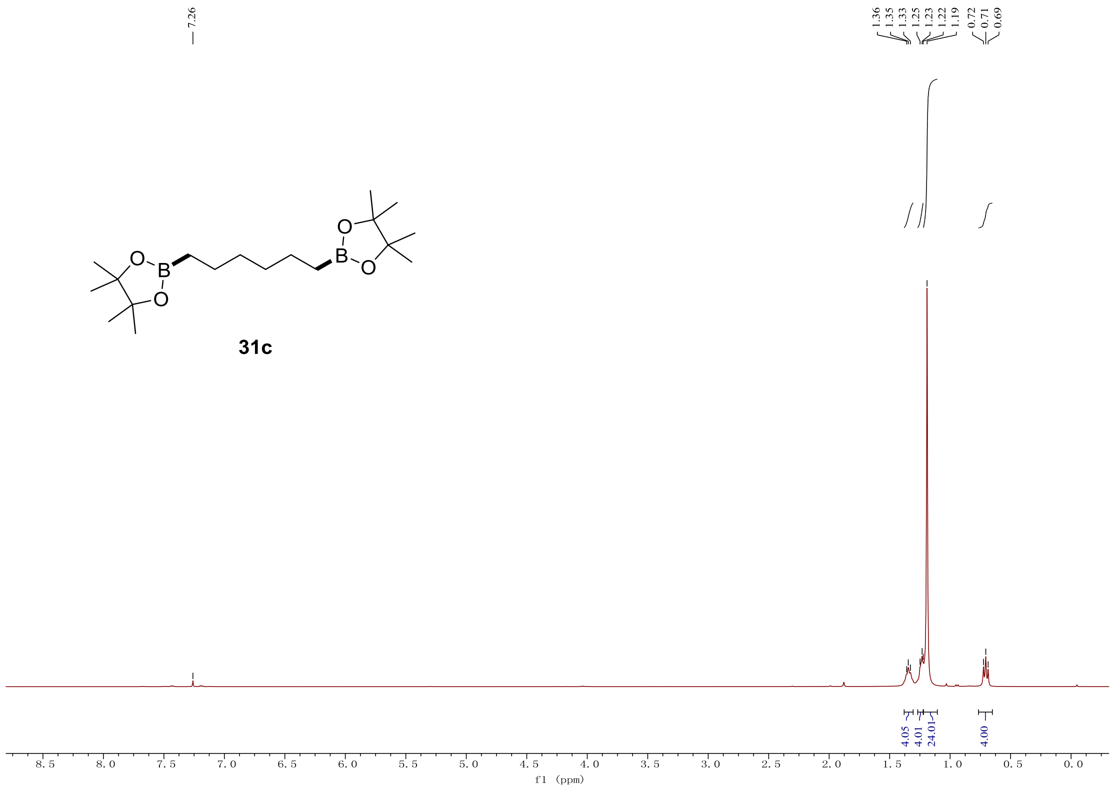

Supplementary Figure 210. ^{1}**H NMR spectrum for 31c.**

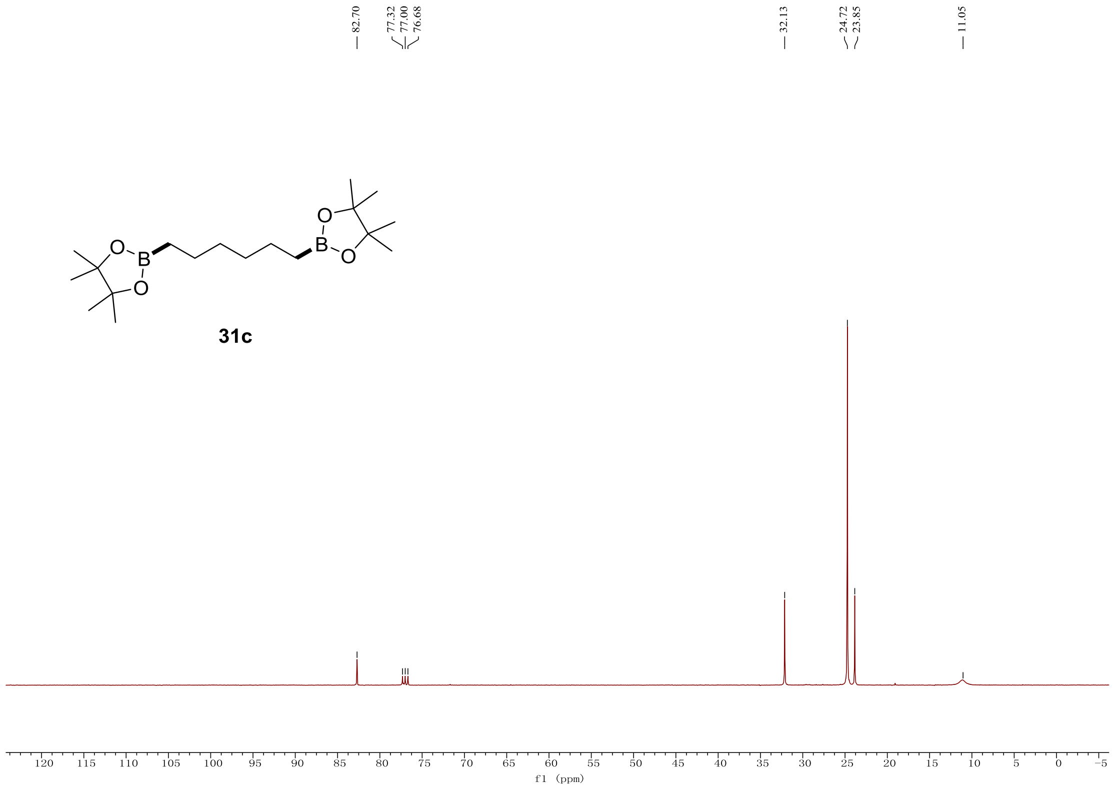

Supplementary Figure 211. ^{13}**C NMR spectrum for 31c.**

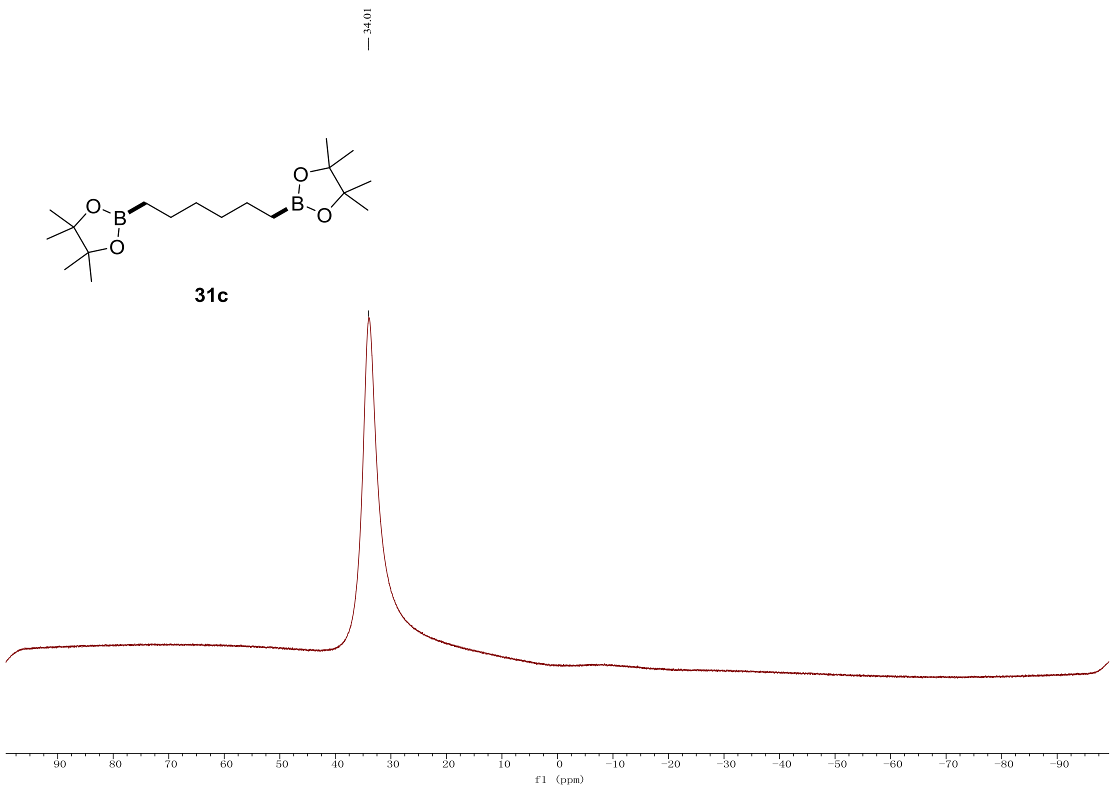

Supplementary Figure 212. ^{11}B NMR spectrum for 31c.

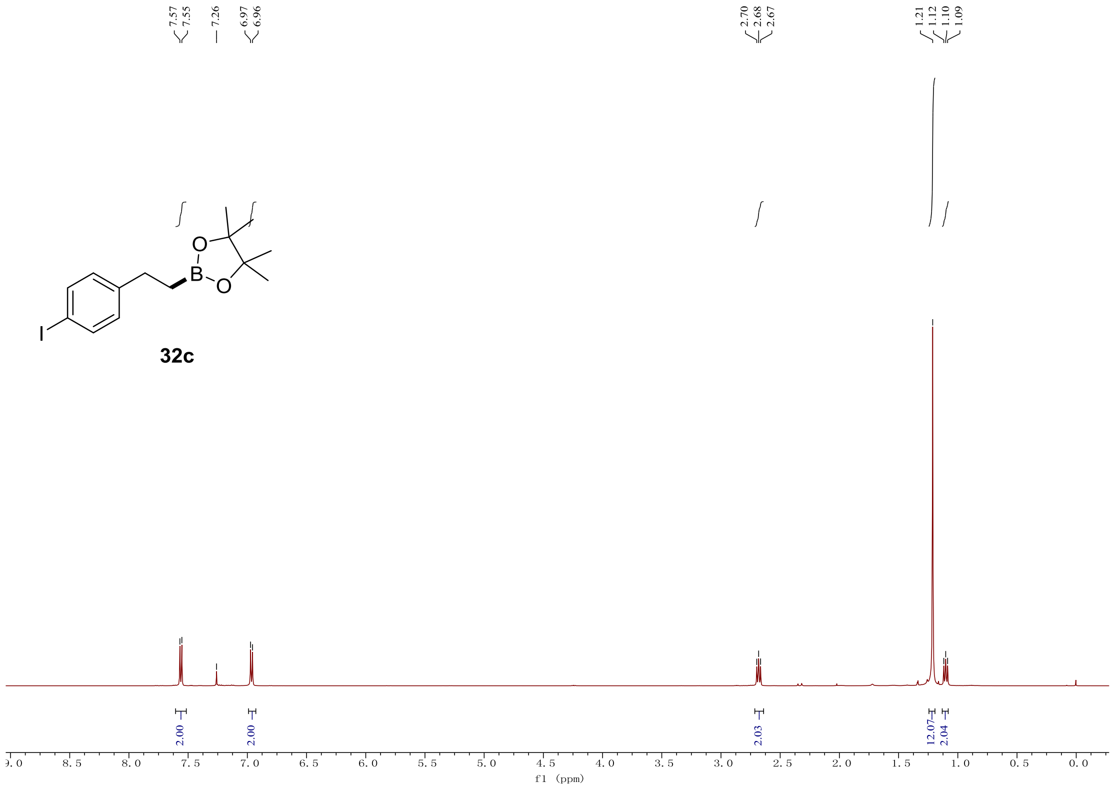

Supplementary Figure 213. ^{1}H NMR spectrum for 32c.

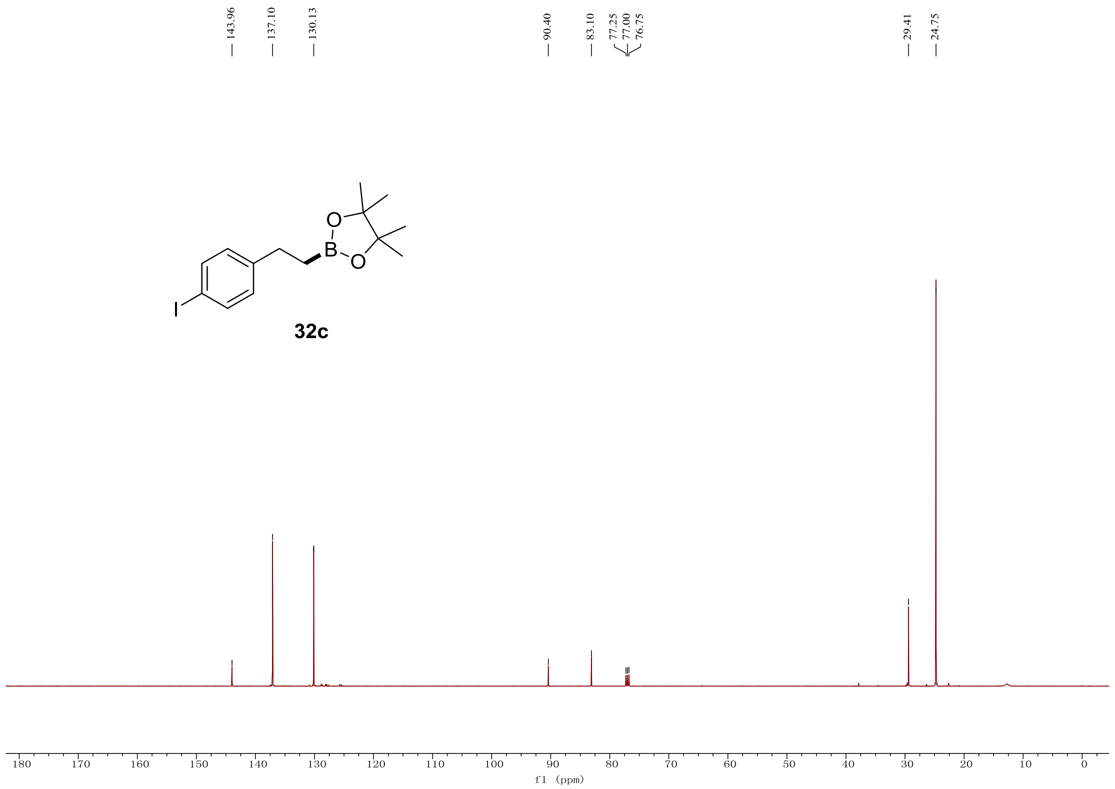

Supplementary Figure 214. ^{13}**C NMR spectrum for 32c**

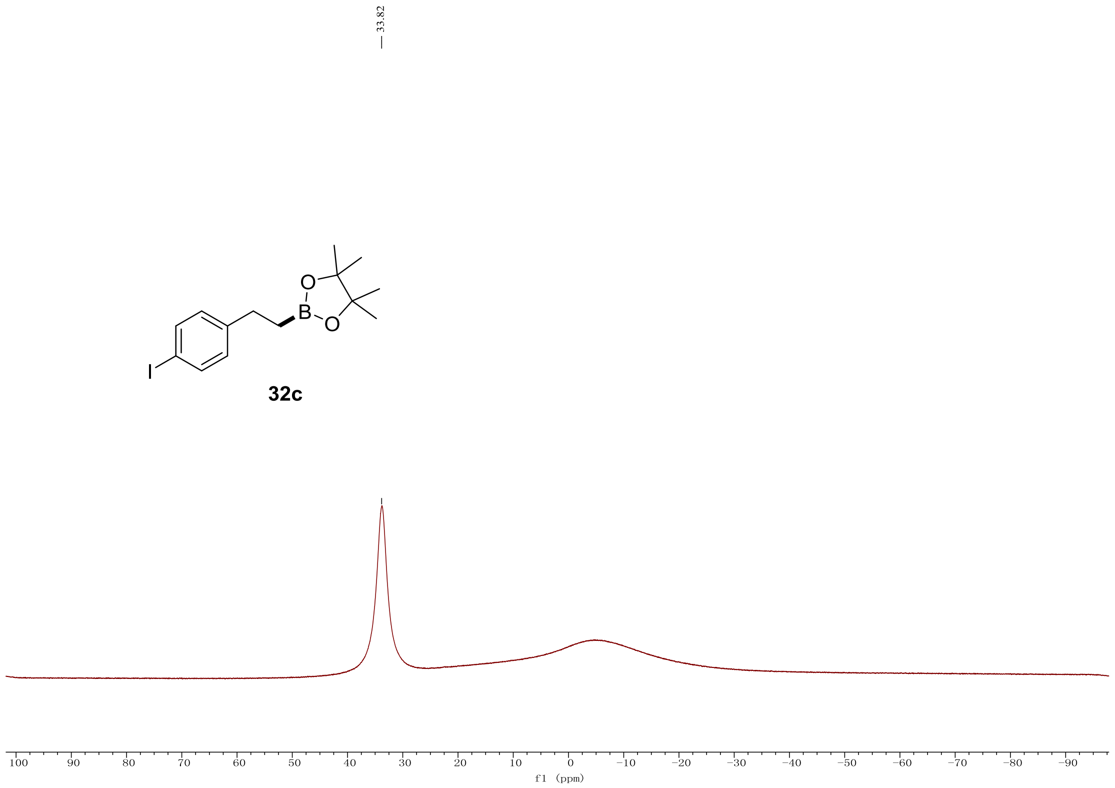

Supplementary Figure 215. ^{11}**B NMR spectrum for 32c.**

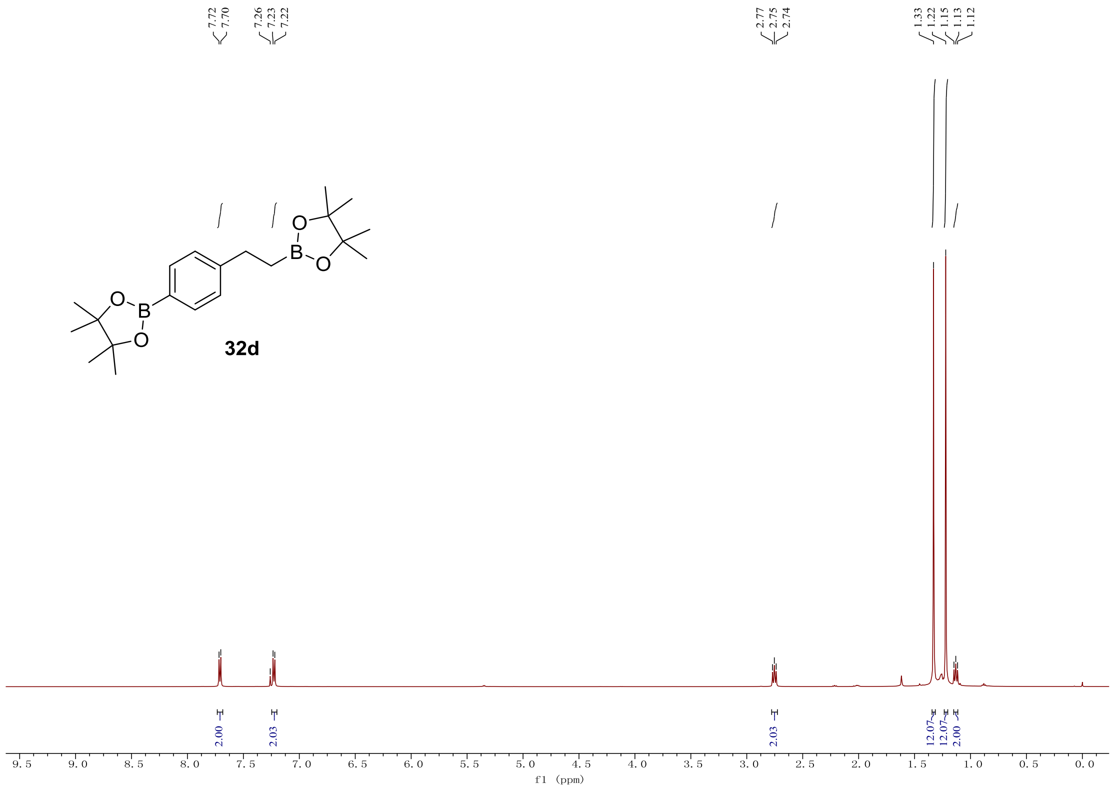

Supplementary Figure 216. ^{1}H NMR spectrum for 32d.

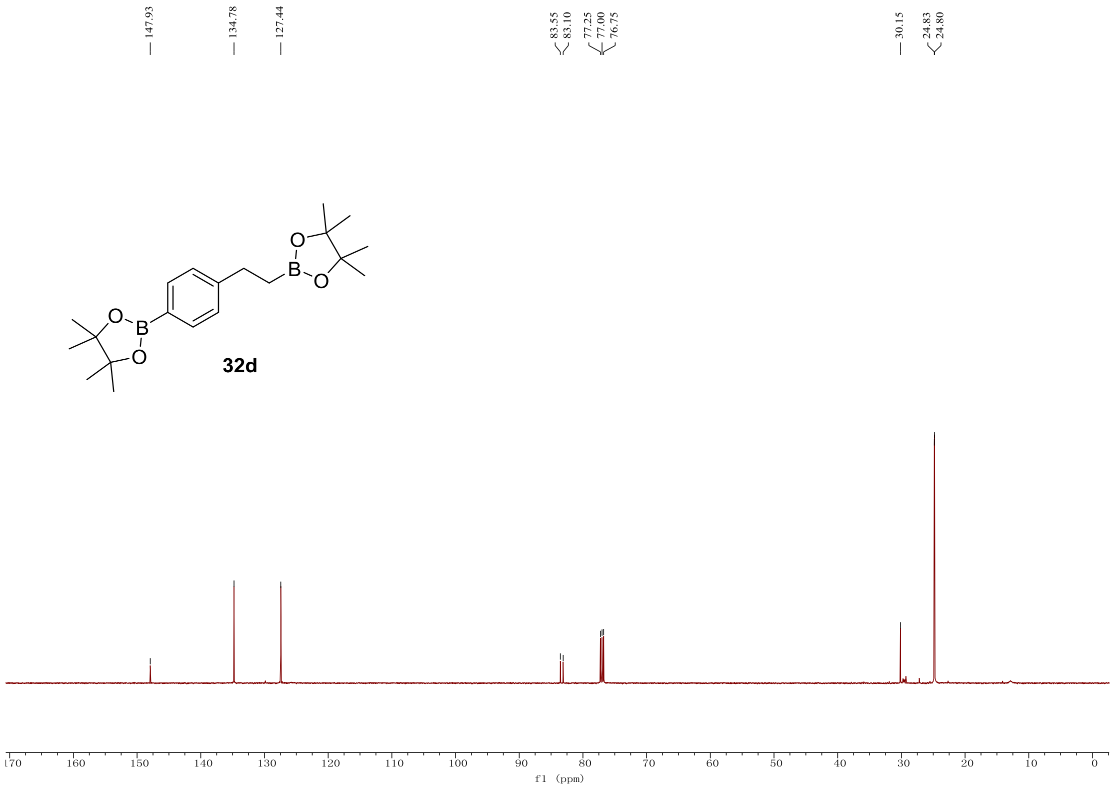

Supplementary Figure 217. ^{13}C NMR spectrum for 32d.

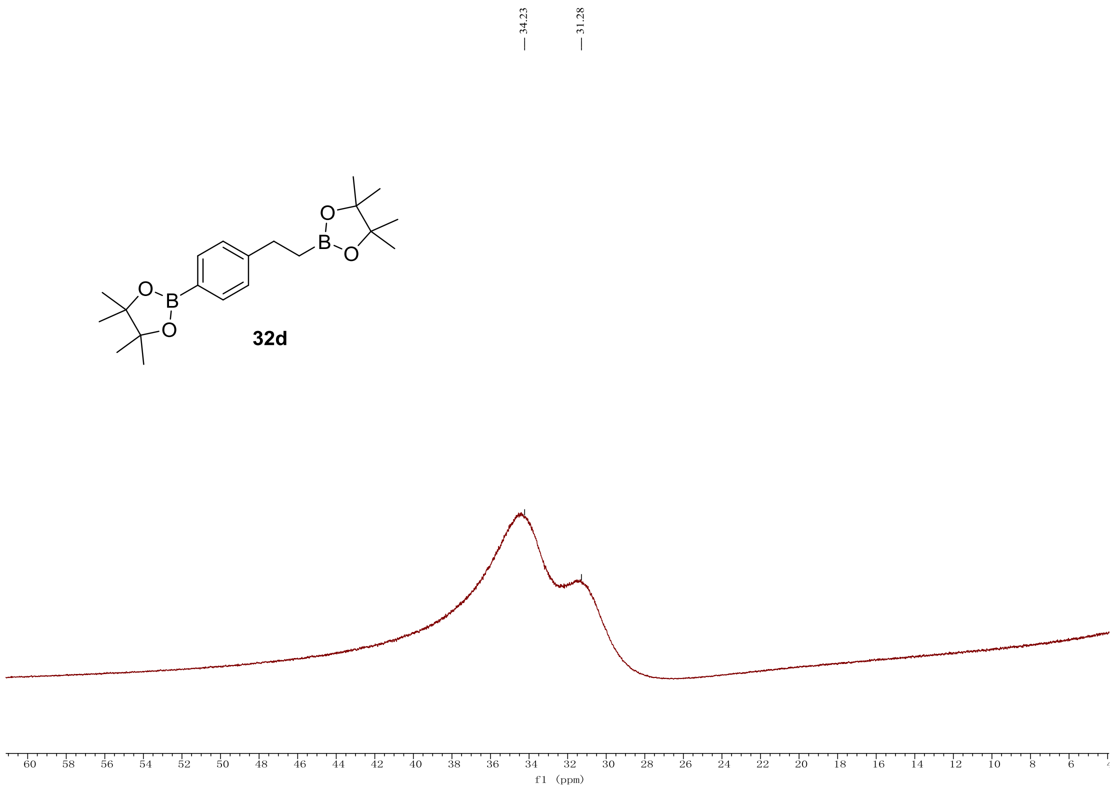

Supplementary Figure 218. ^{11}B NMR spectrum for 32d.

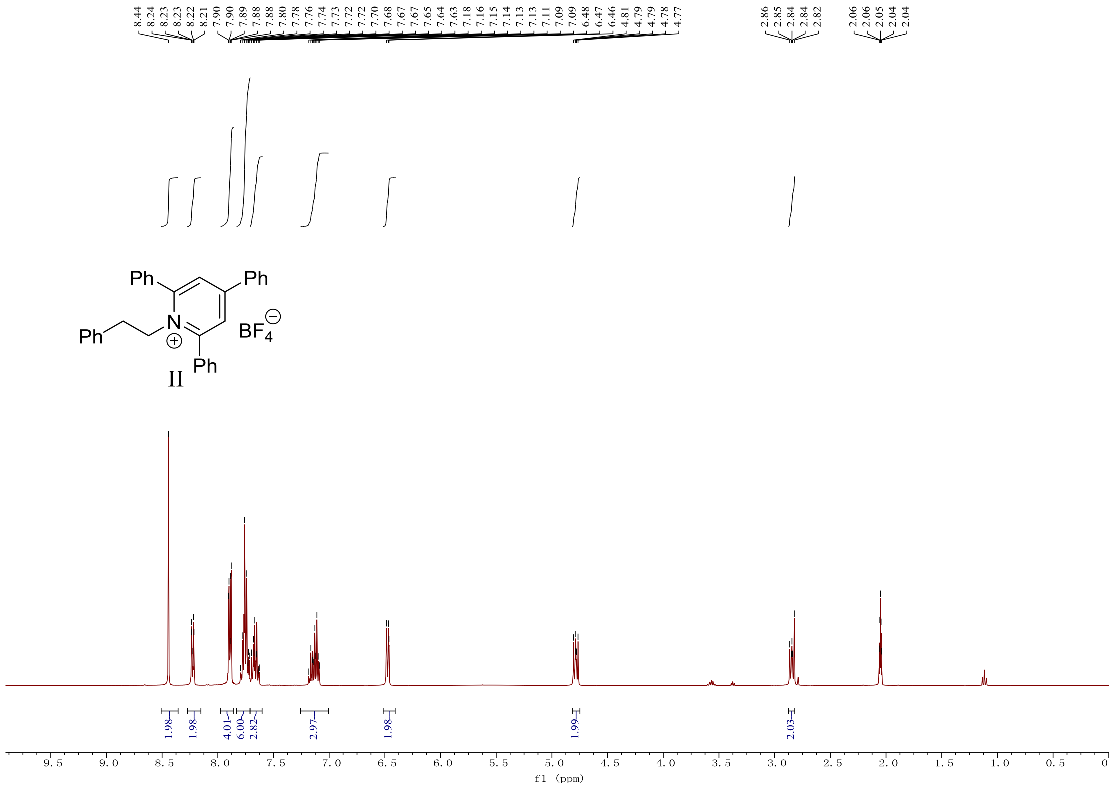

Supplementary Figure 219. ^{1}H NMR spectrum for II.

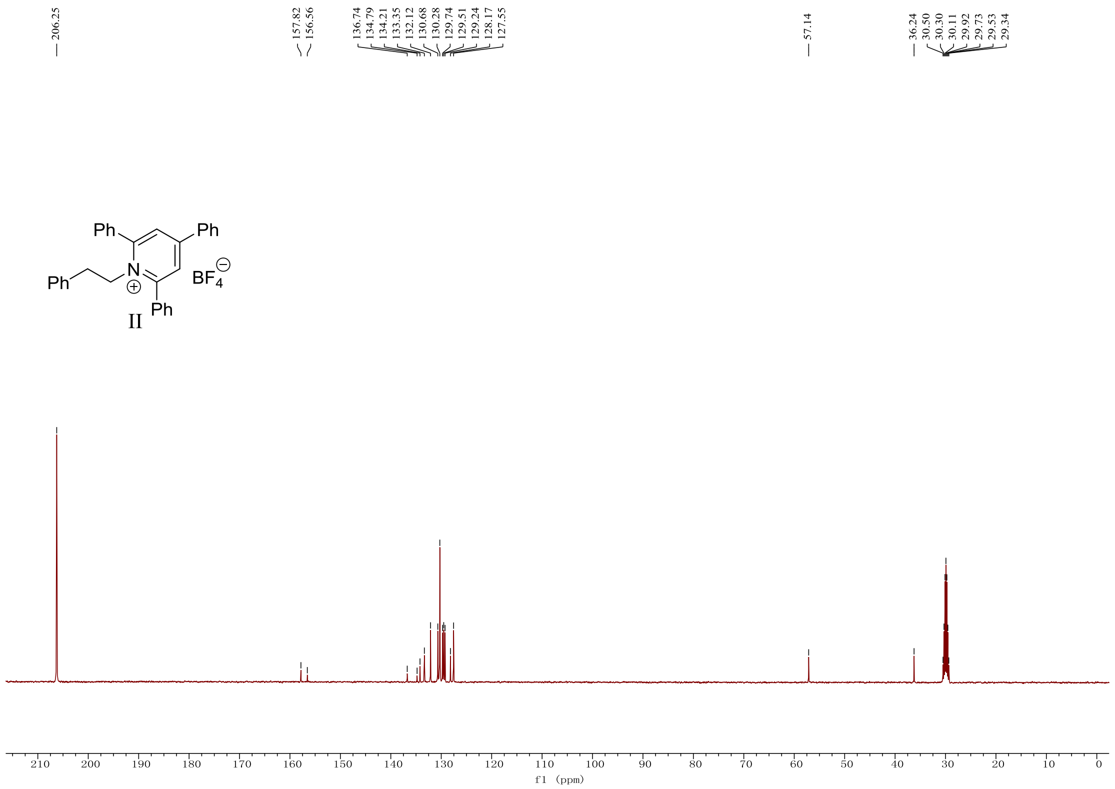

Supplementary Figure 220. ^{13}**C NMR spectrum for II.**

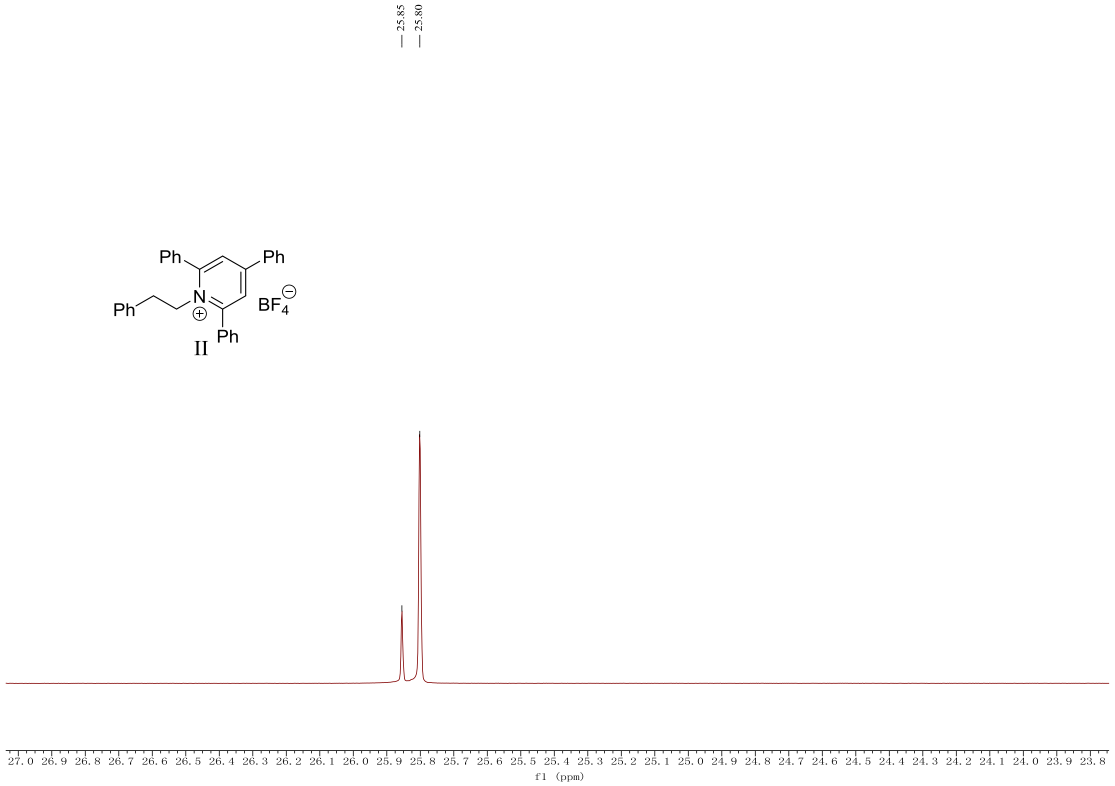

Supplementary Figure 221. ^{11}**B NMR spectrum for II.**

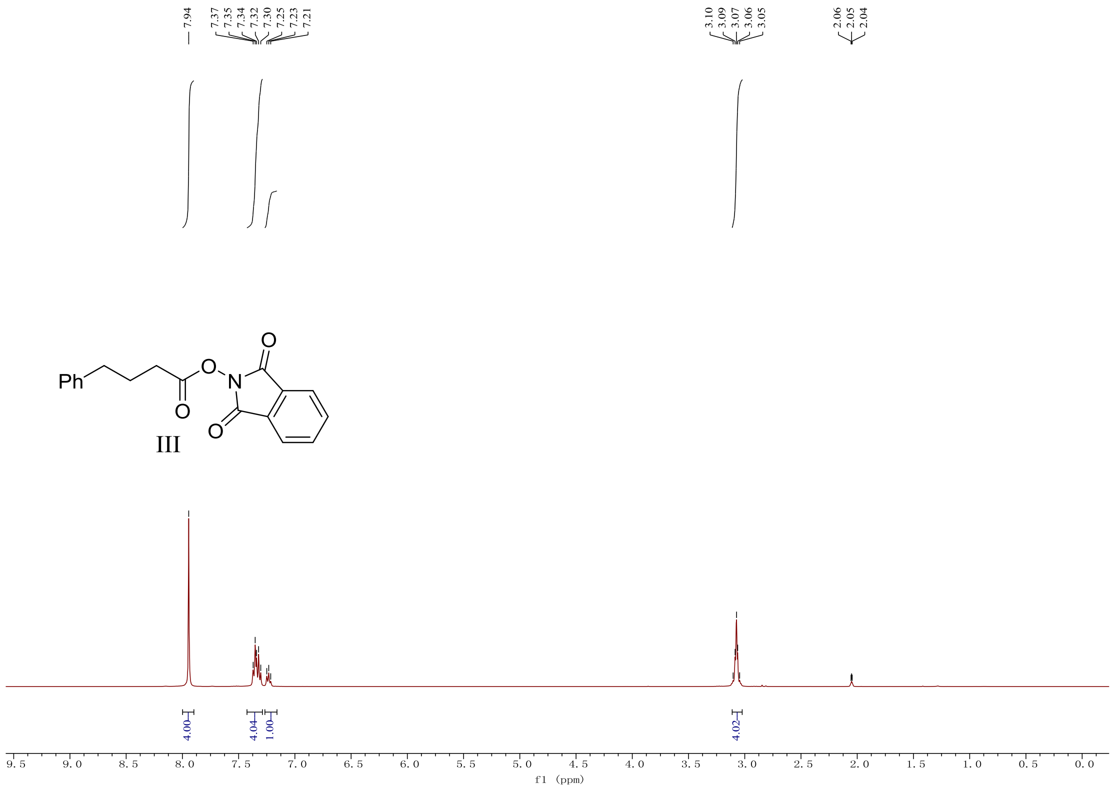

Supplementary Figure 222. **^{1}H NMR spectrum for III.**

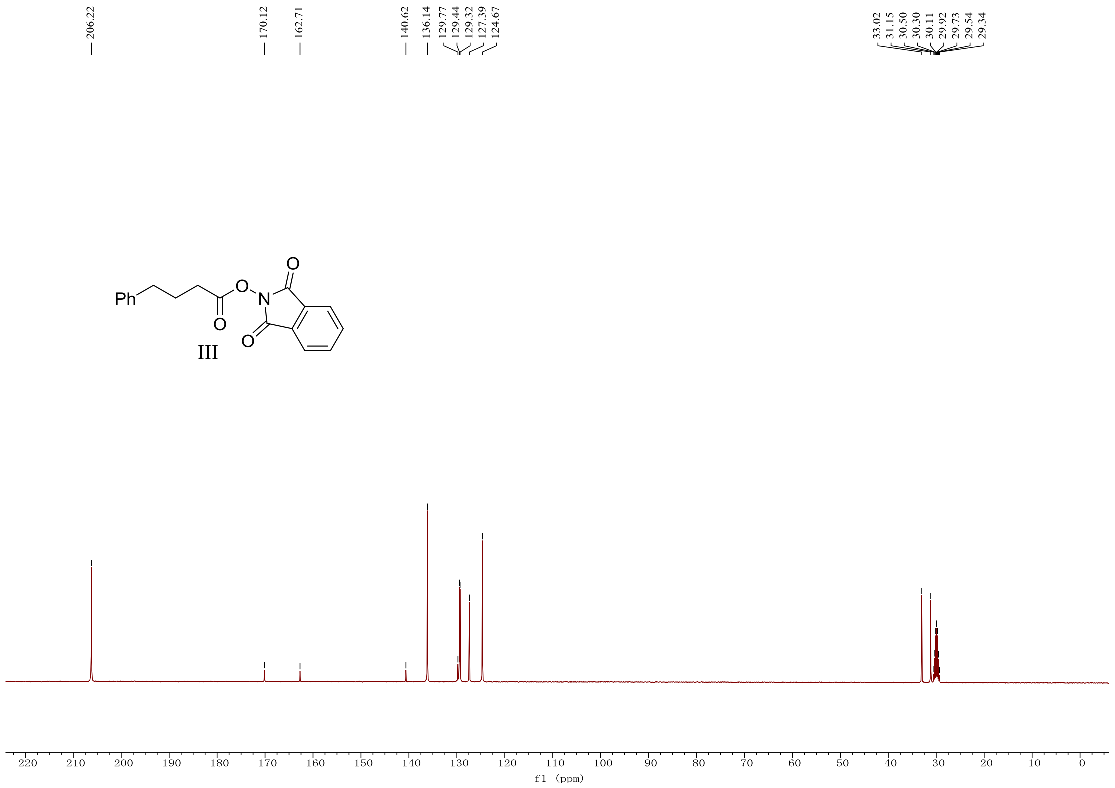

Supplementary Figure 223. **^{13}C NMR spectrum for III.**

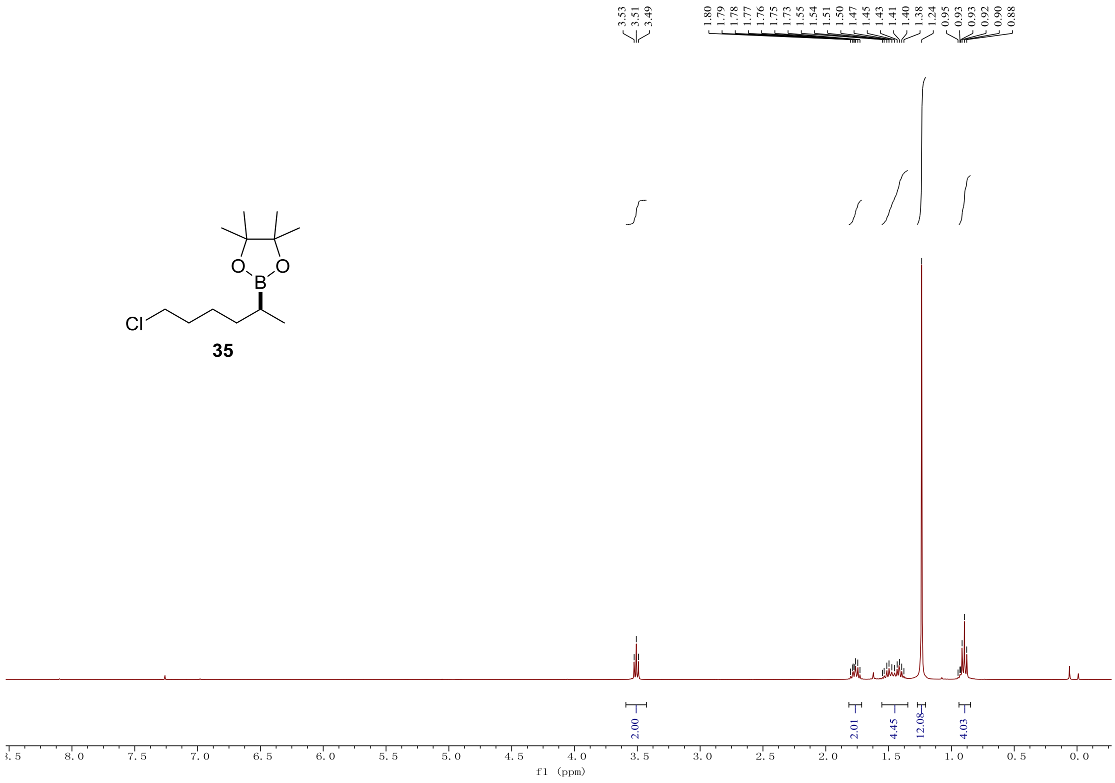

Supplementary Figure 224. ^{1}H NMR spectrum for 35.

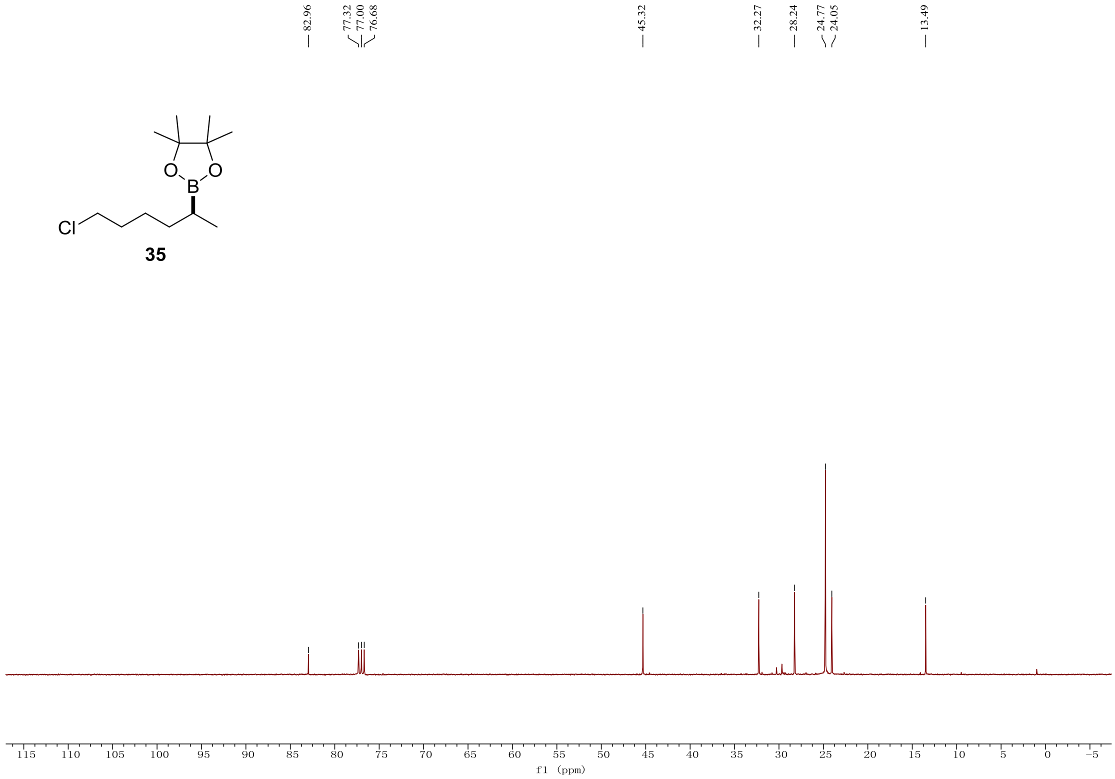

Supplementary Figure 225. ^{13}C NMR spectrum for 35.

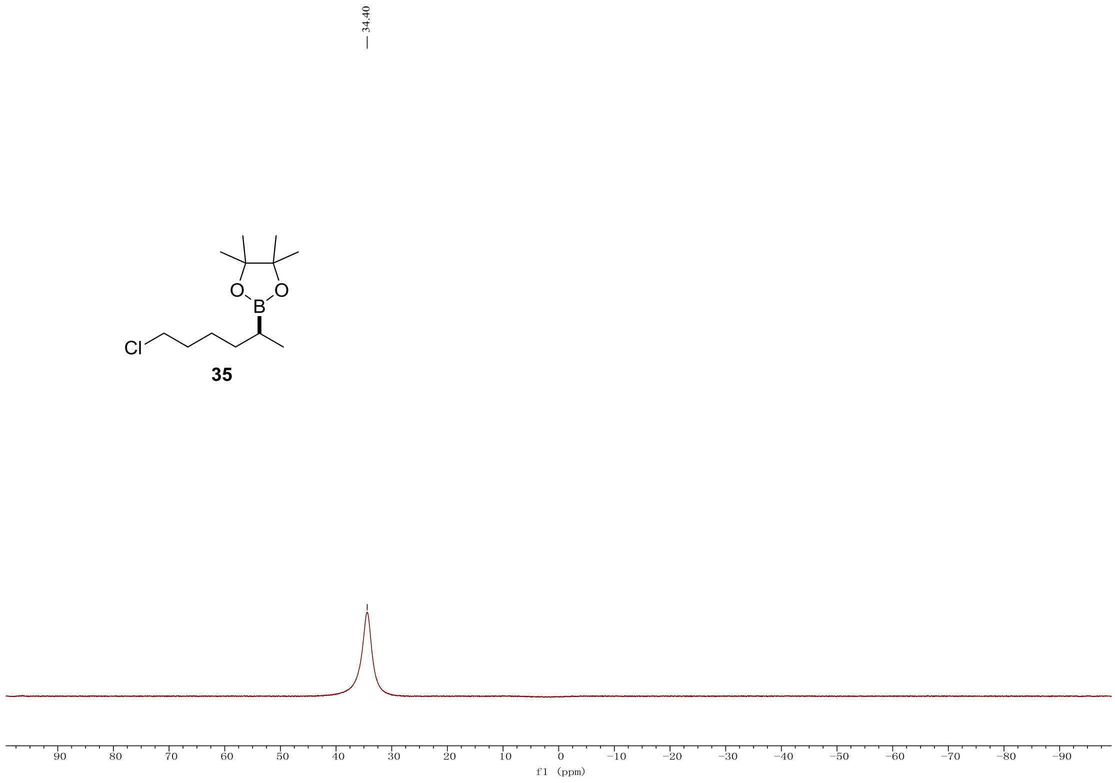

Supplementary Figure 226. ^{11}**B NMR spectrum for 35.**

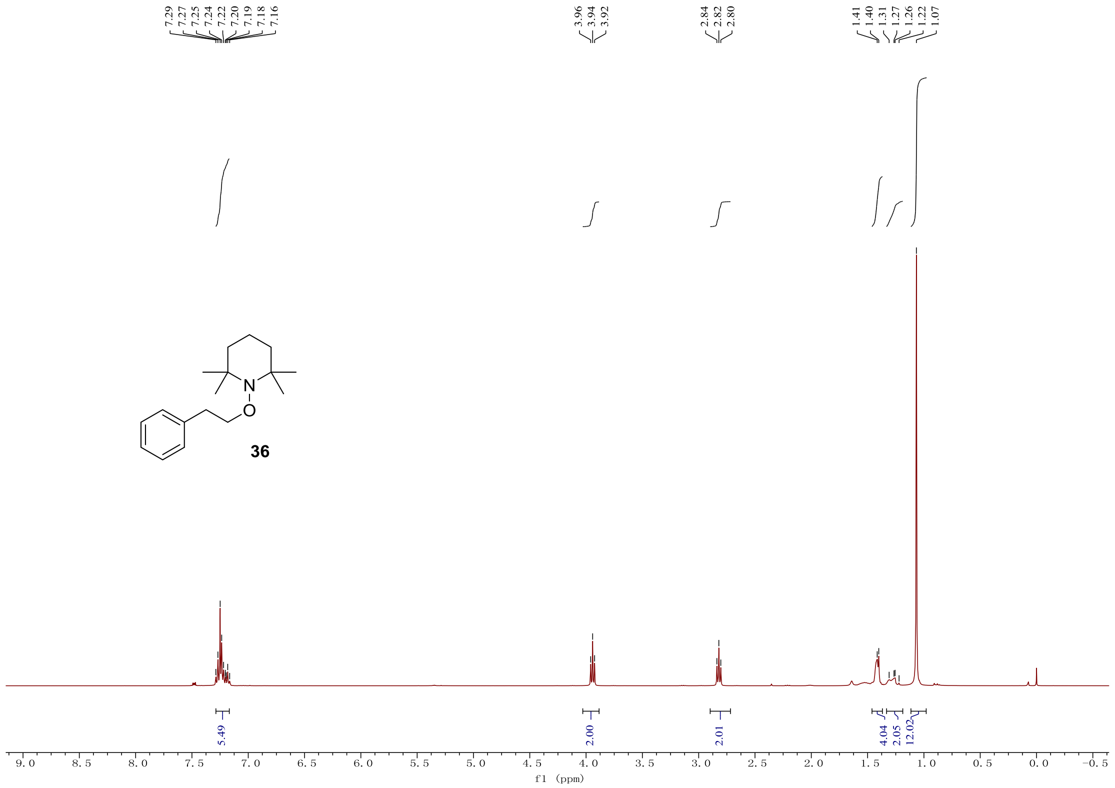

Supplementary Figure 227. ^{1}**H NMR spectrum for 36.**

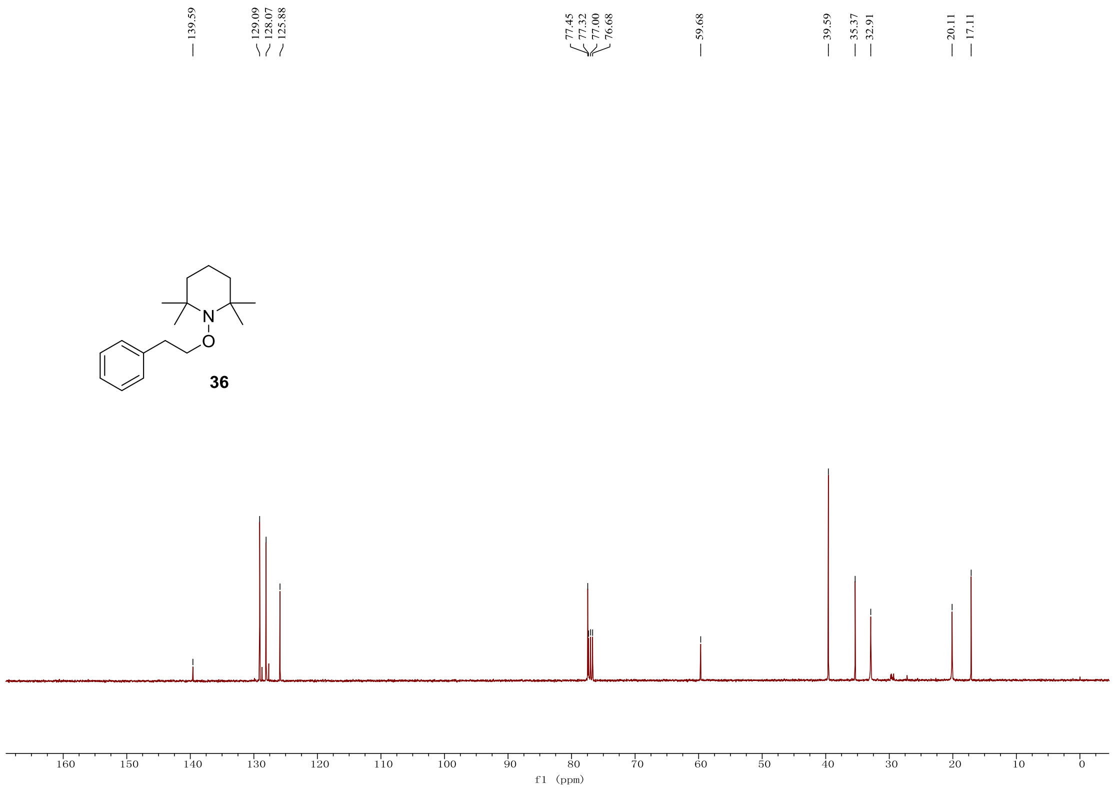

Supplementary Figure 228. ^{13}**C NMR spectrum for 36**.

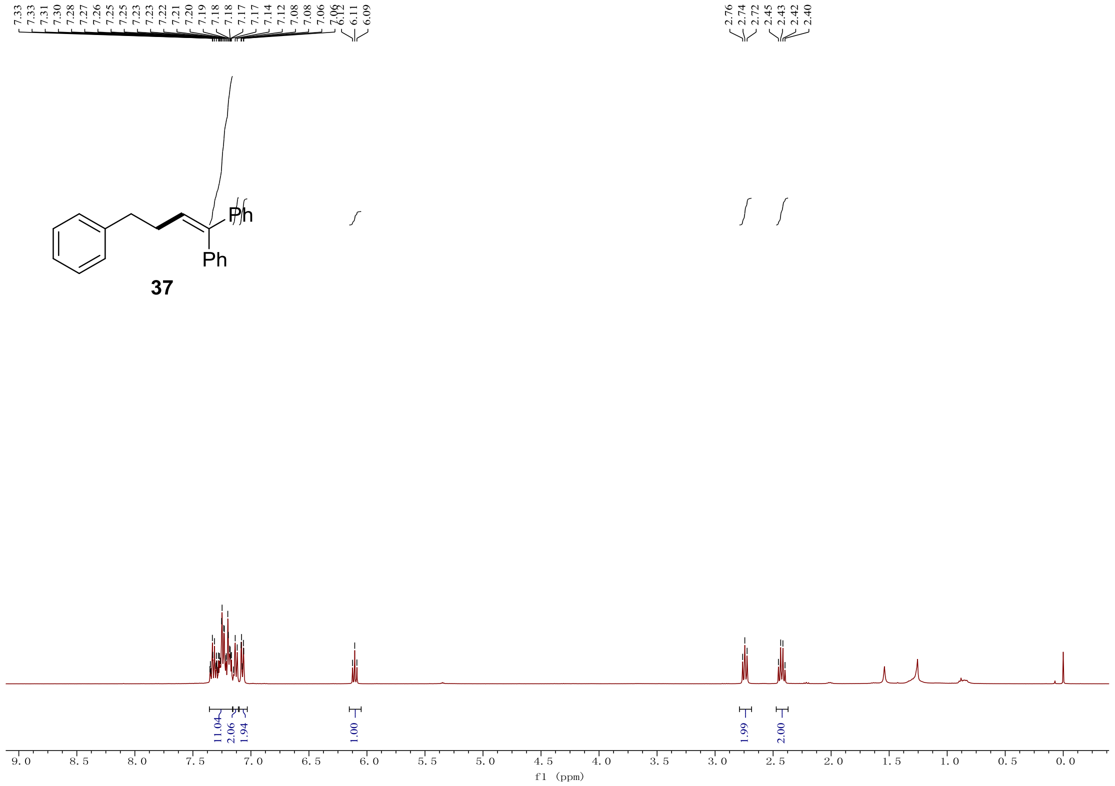

Supplementary Figure 229. ^{1}**H NMR spectrum for 37.**

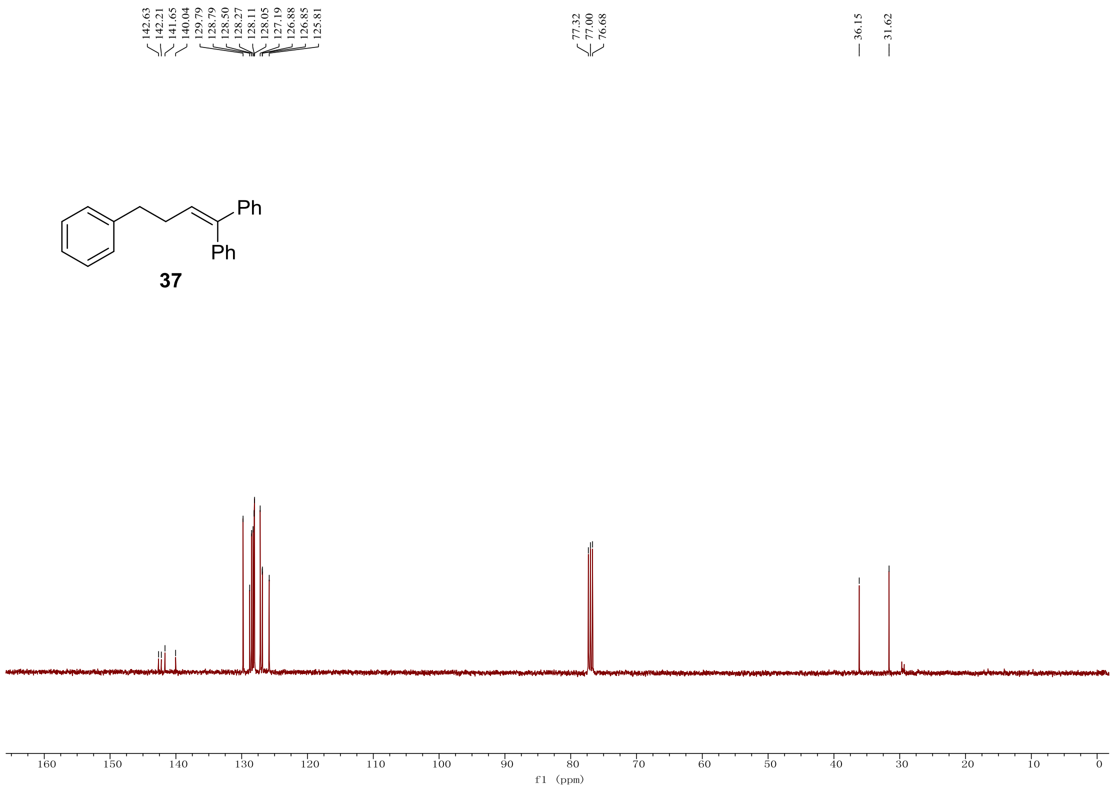

Supplementary Figure 230. ^{13}**C NMR spectrum for 37.**

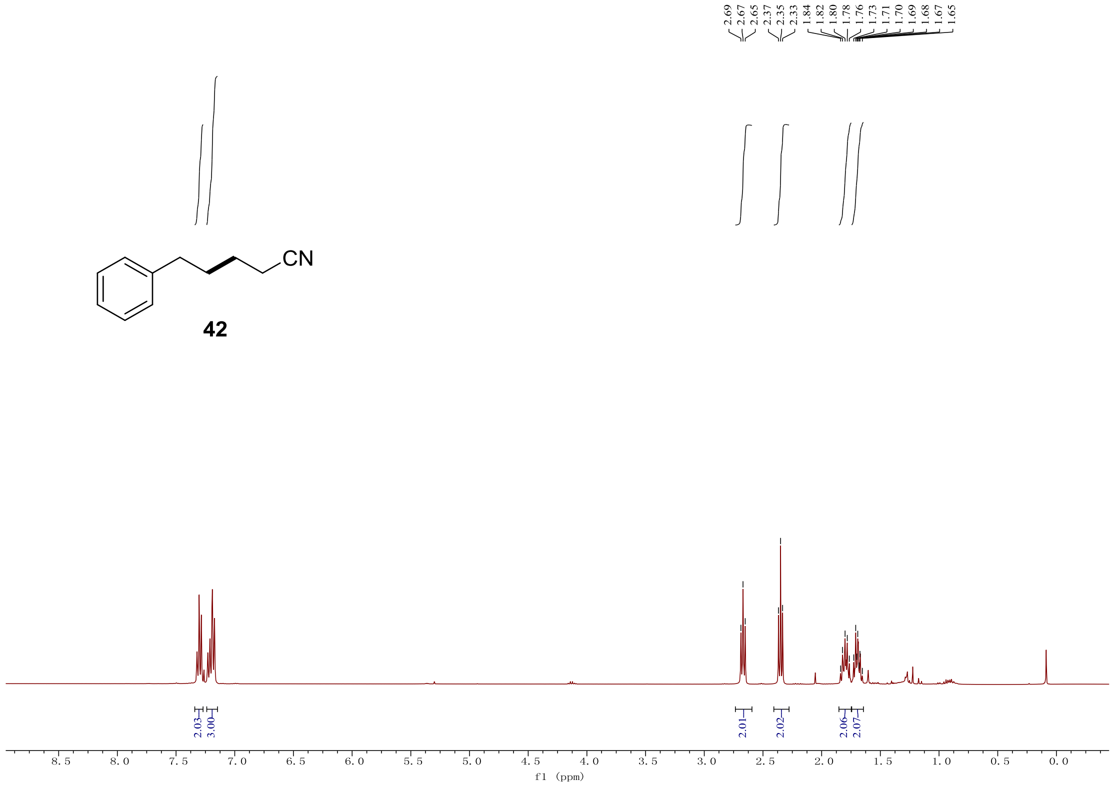

Supplementary Figure 231. ^{1}**H NMR spectrum for 42.**

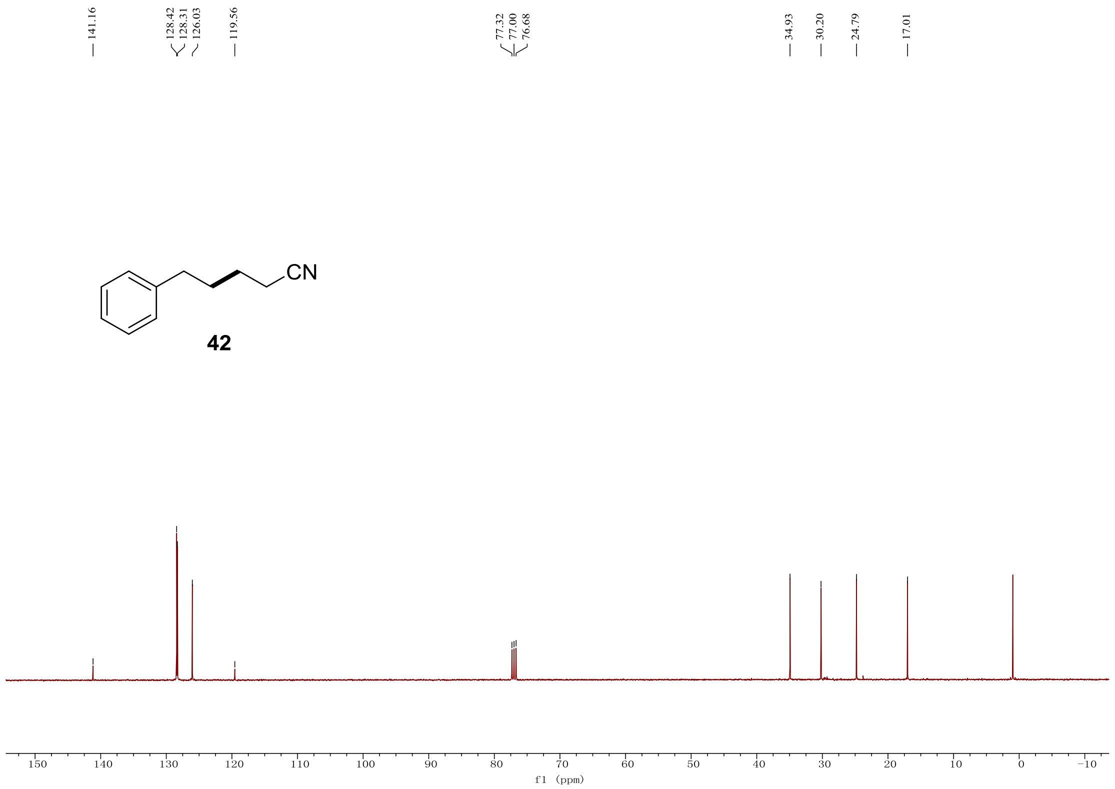

Supplementary Figure 232. ^{13}**C NMR spectrum for 42.**

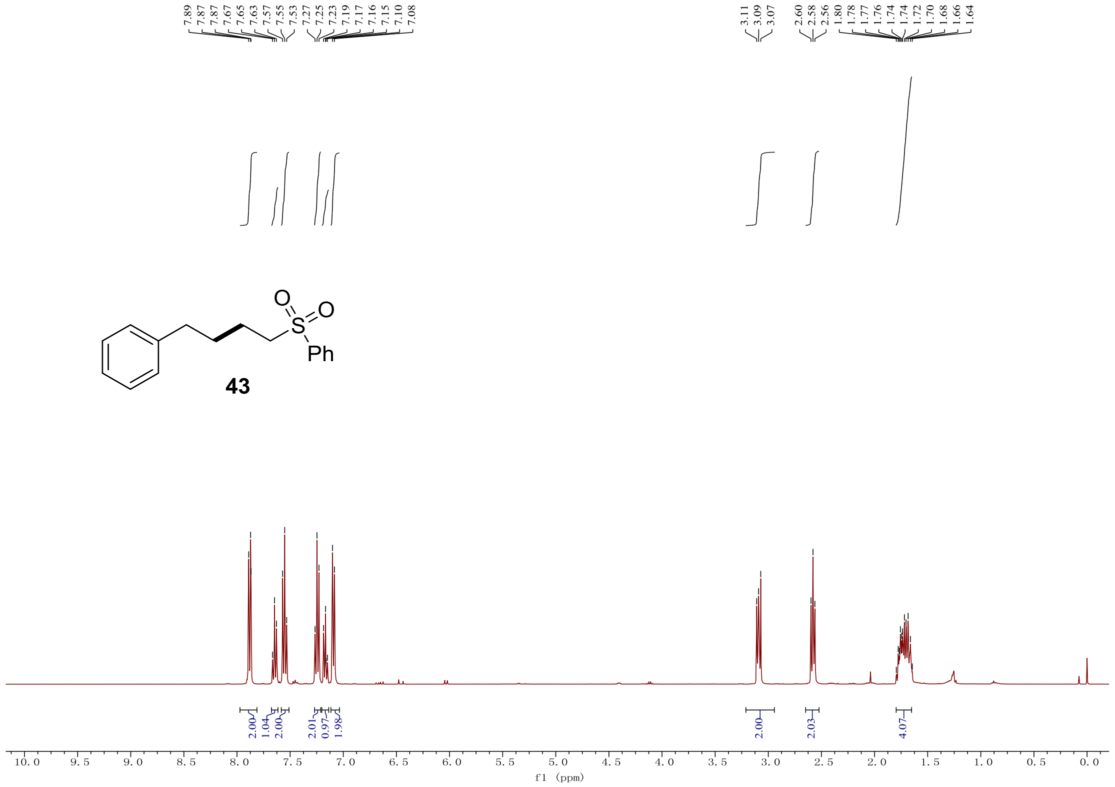

Supplementary Figure 233. ^{1}**H NMR spectrum for 43.**

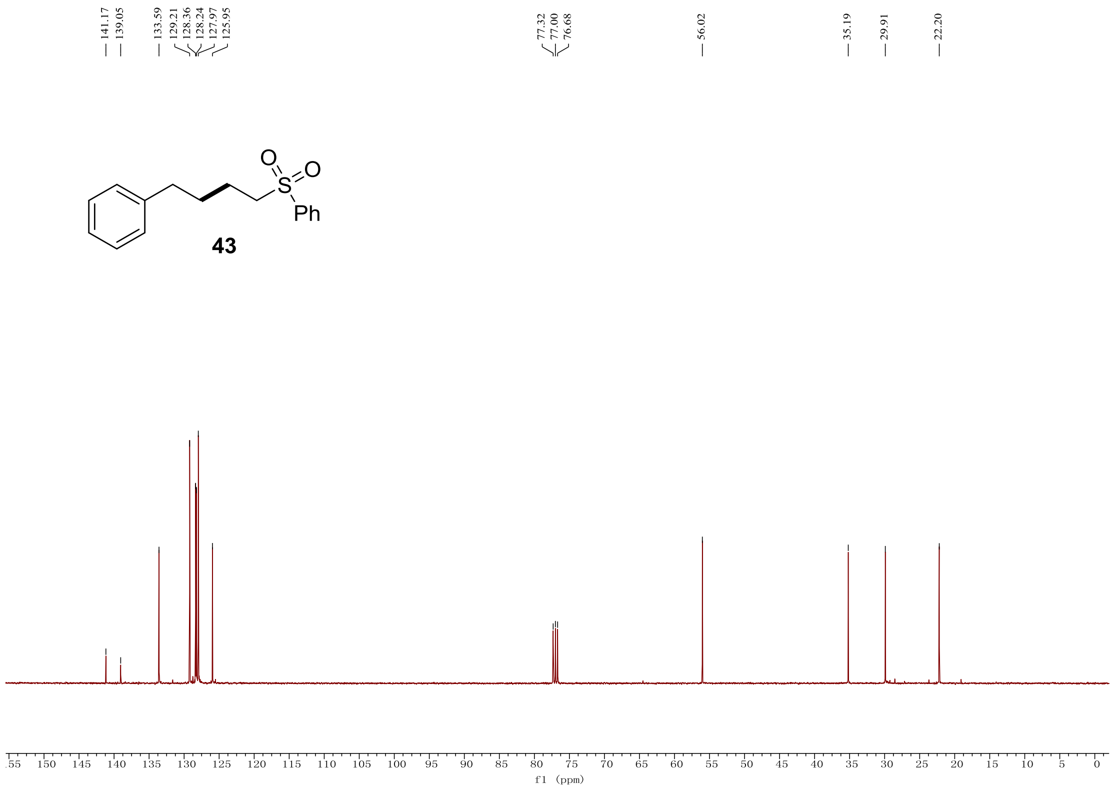

Supplementary Figure 234. ^{13}**C NMR spectrum for 43.**

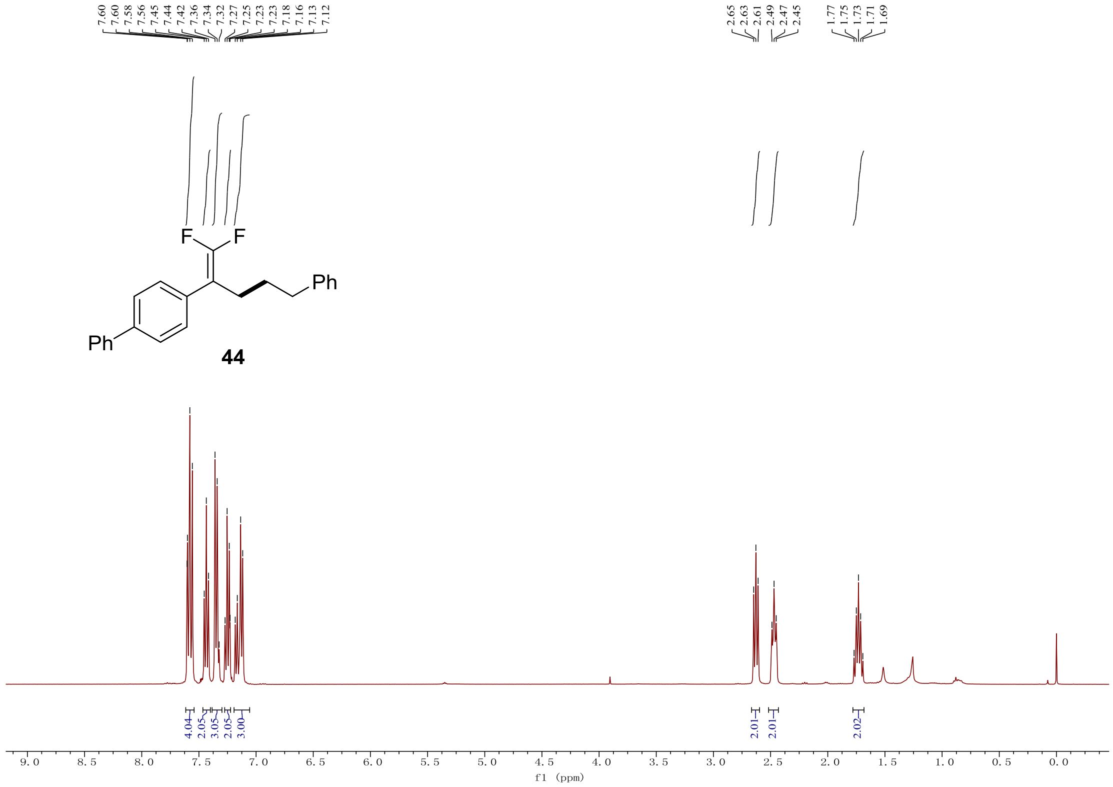

Supplementary Figure 235. ^{1}**H NMR spectrum for 44.**

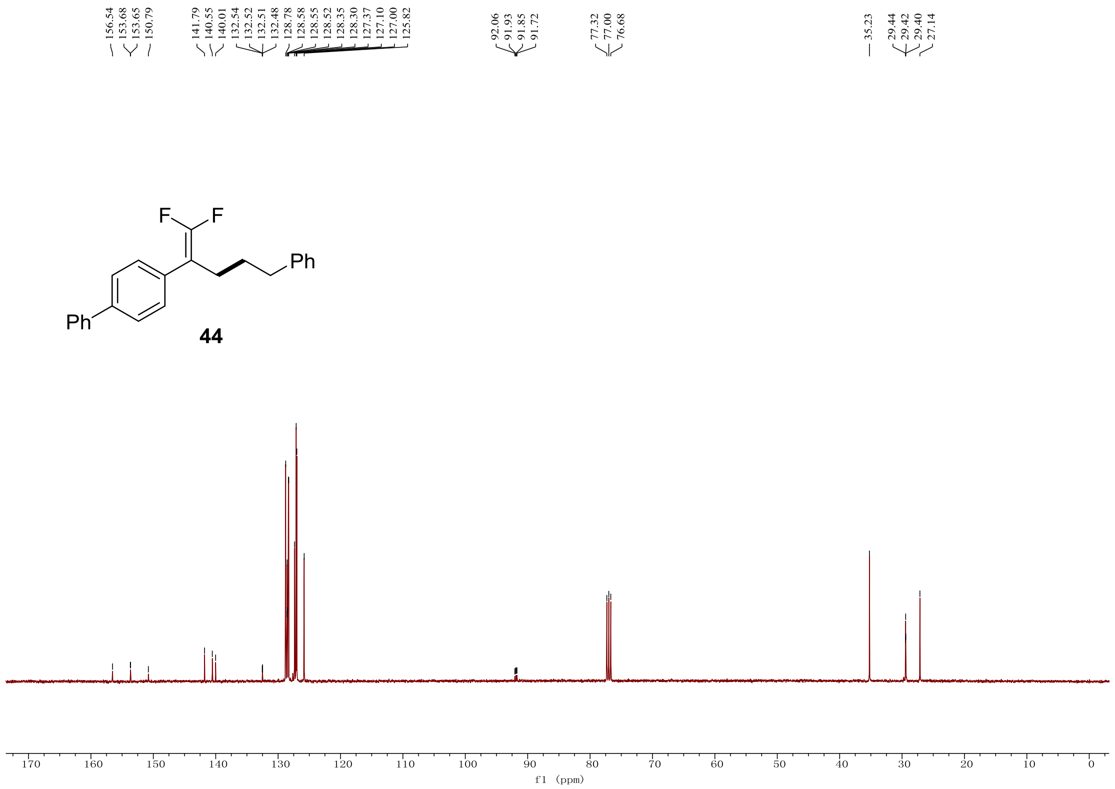

Supplementary Figure 236. ^{13}C NMR spectrum for 44.

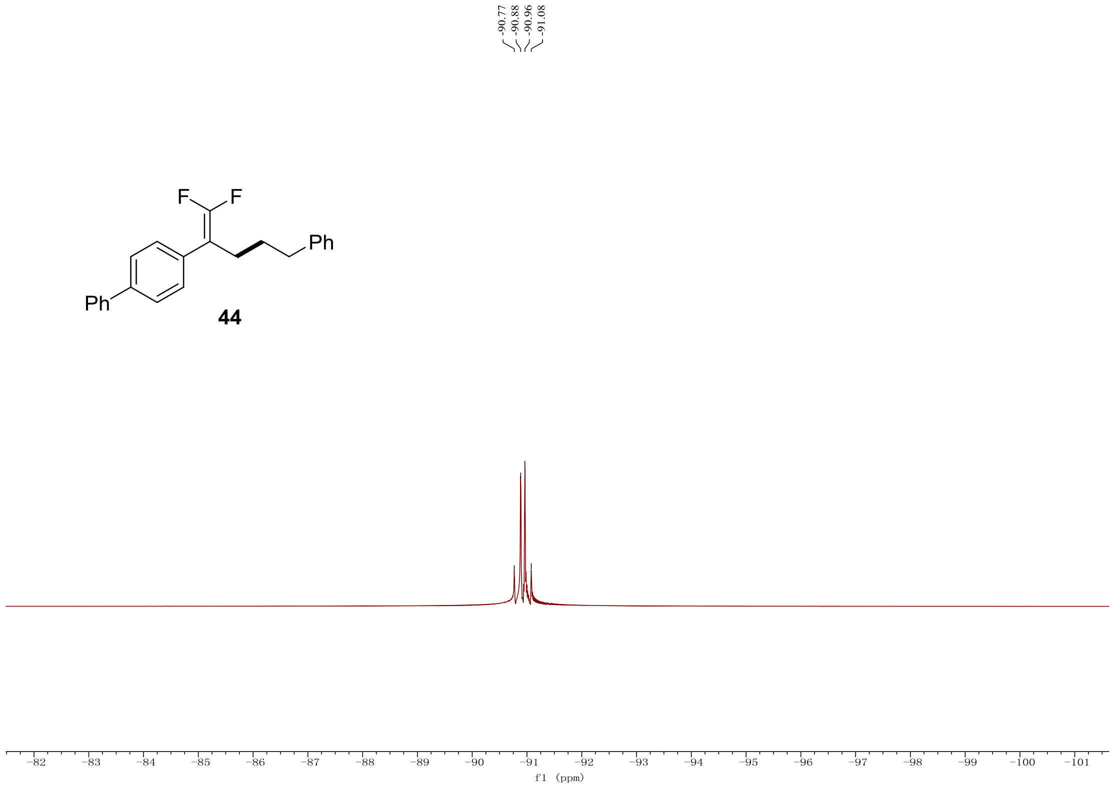

Supplementary Figure 237. ^{19}F NMR spectrum for 44.

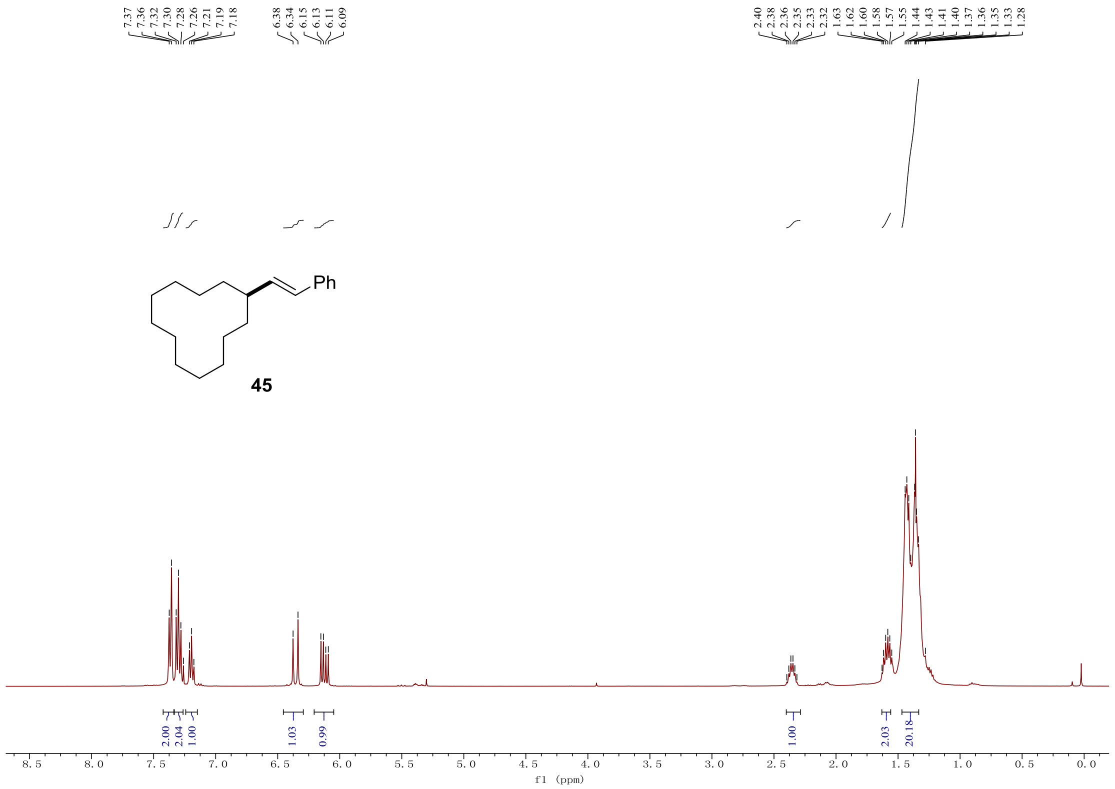

Supplementary Figure 238. ^{1}H NMR spectrum for 45.

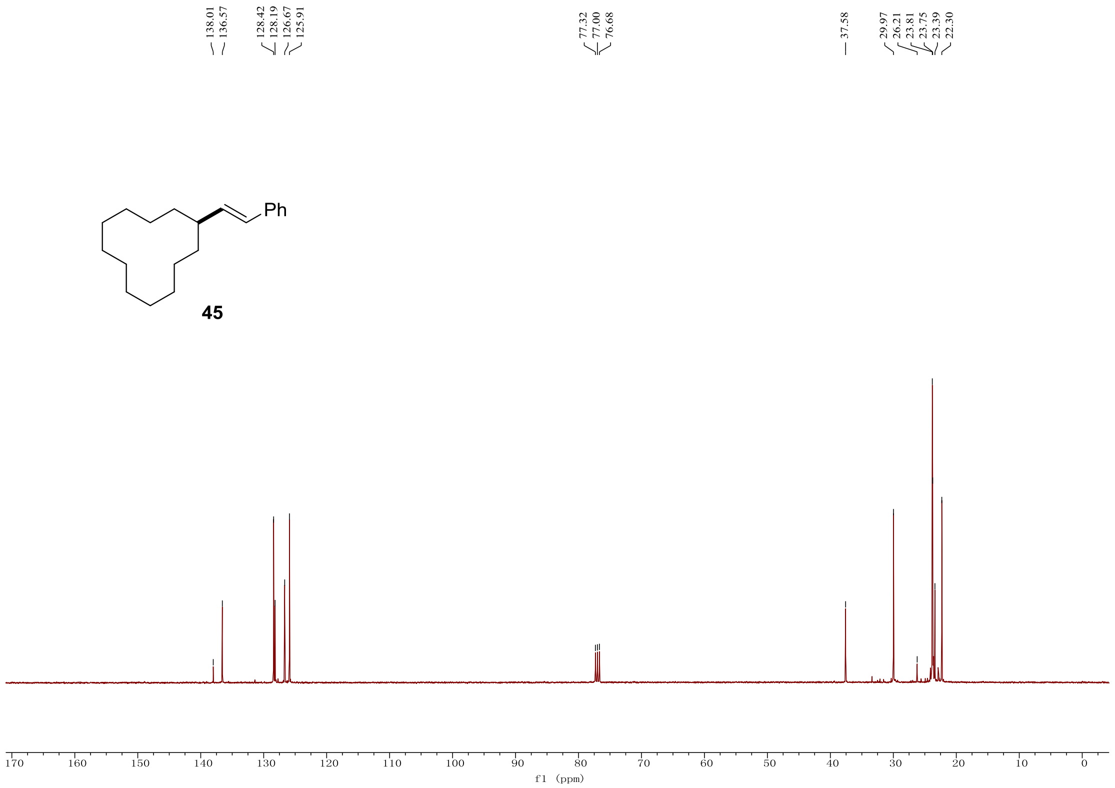

Supplementary Figure 239. ^{13}C NMR spectrum for 45.

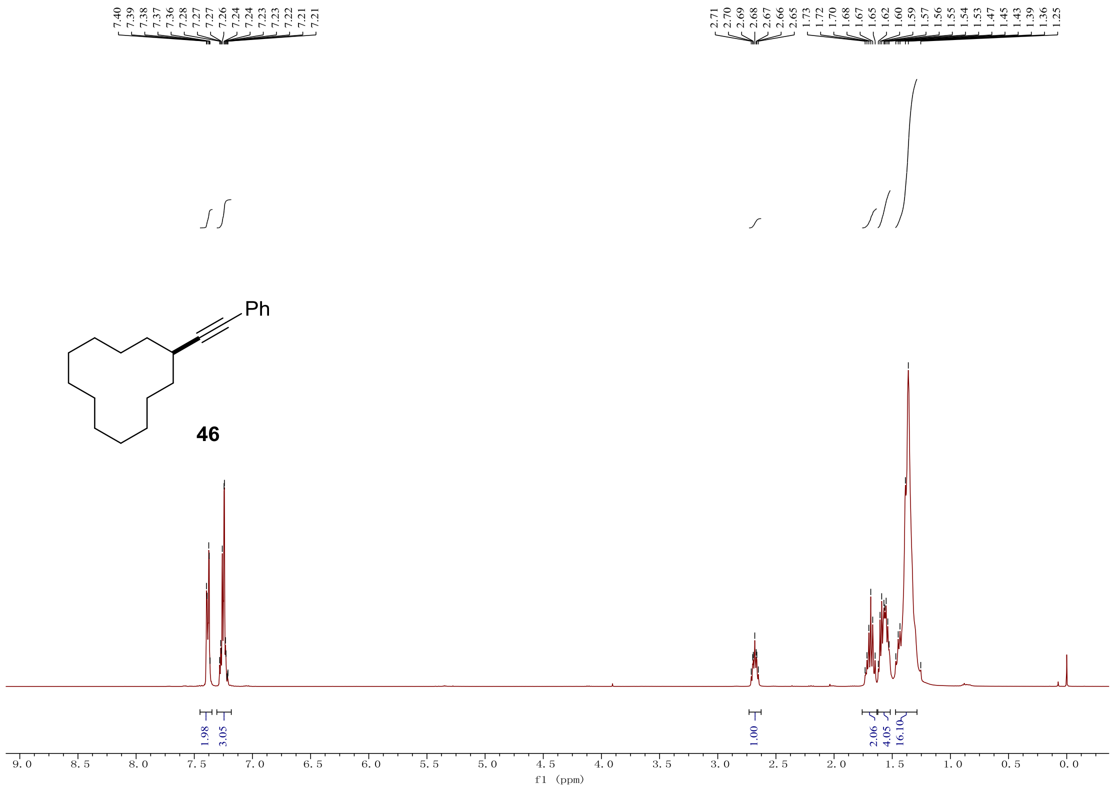

Supplementary Figure 240. ^{1}H NMR spectrum for 46.

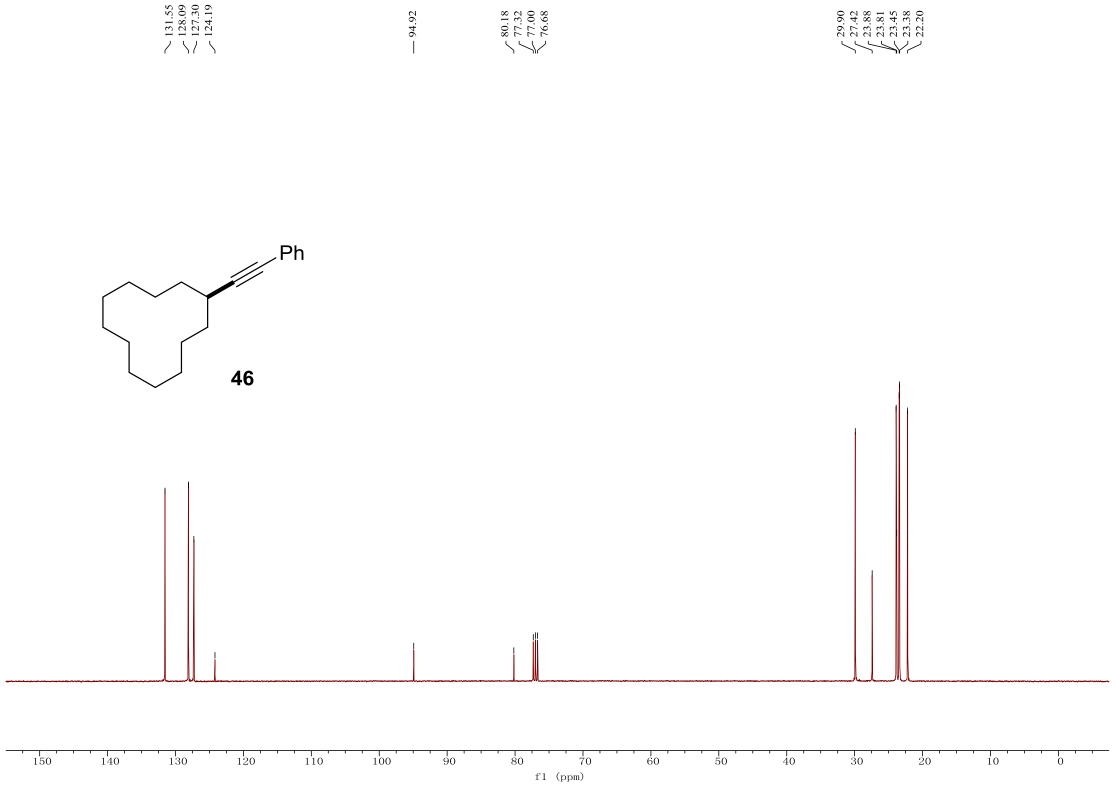

Supplementary Figure 241. ^{13}C NMR spectrum for 46.

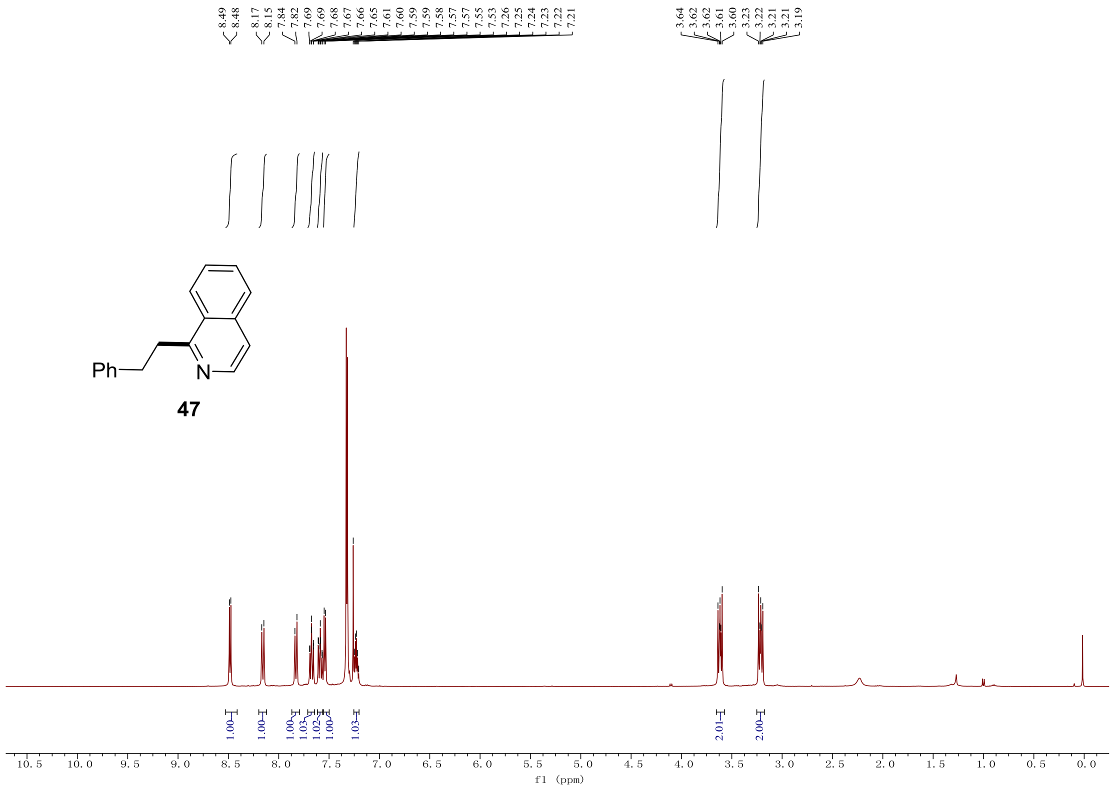

Supplementary Figure 242. ^{1}H NMR spectrum for 47.

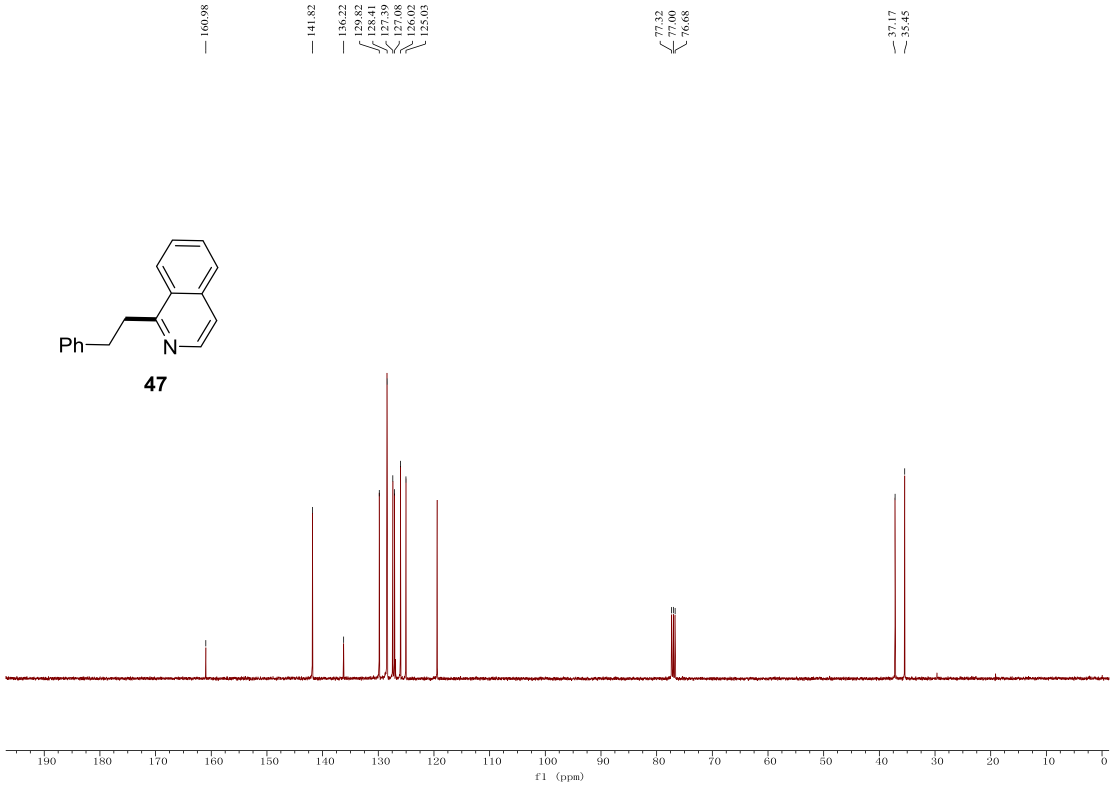

Supplementary Figure 243. ^{13}C NMR spectrum for 47.

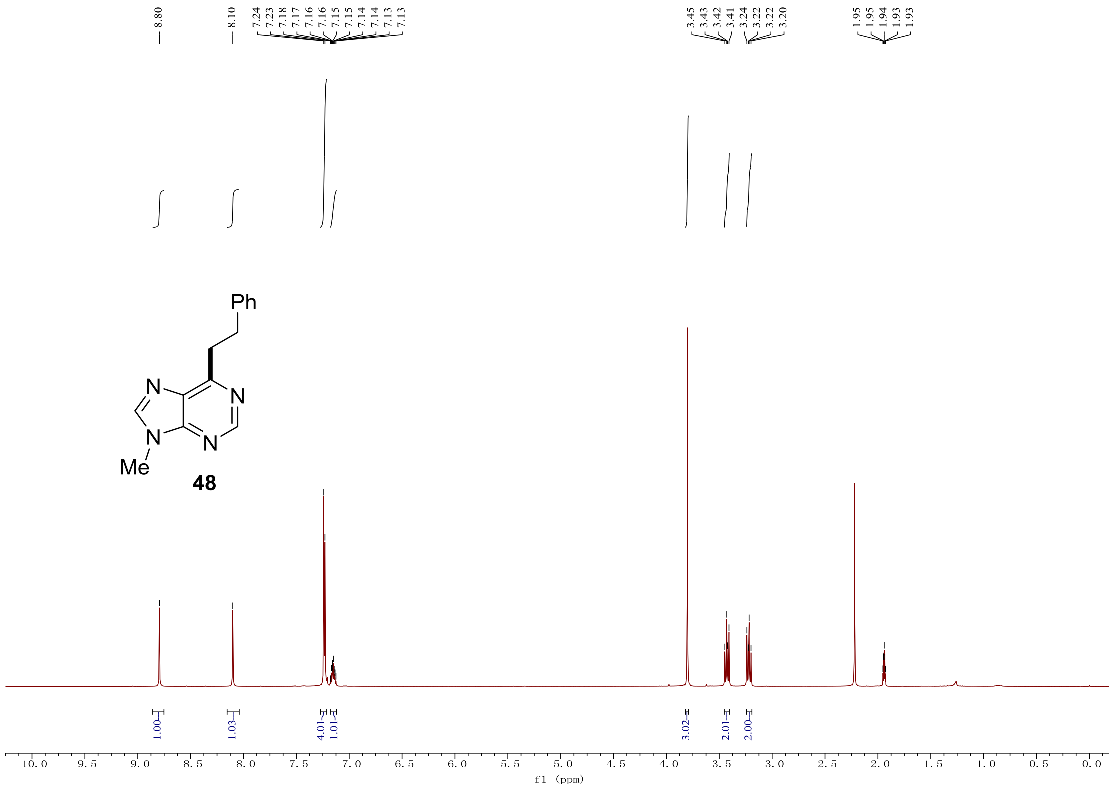

Supplementary Figure 244. ^{1}H NMR spectrum for 48.

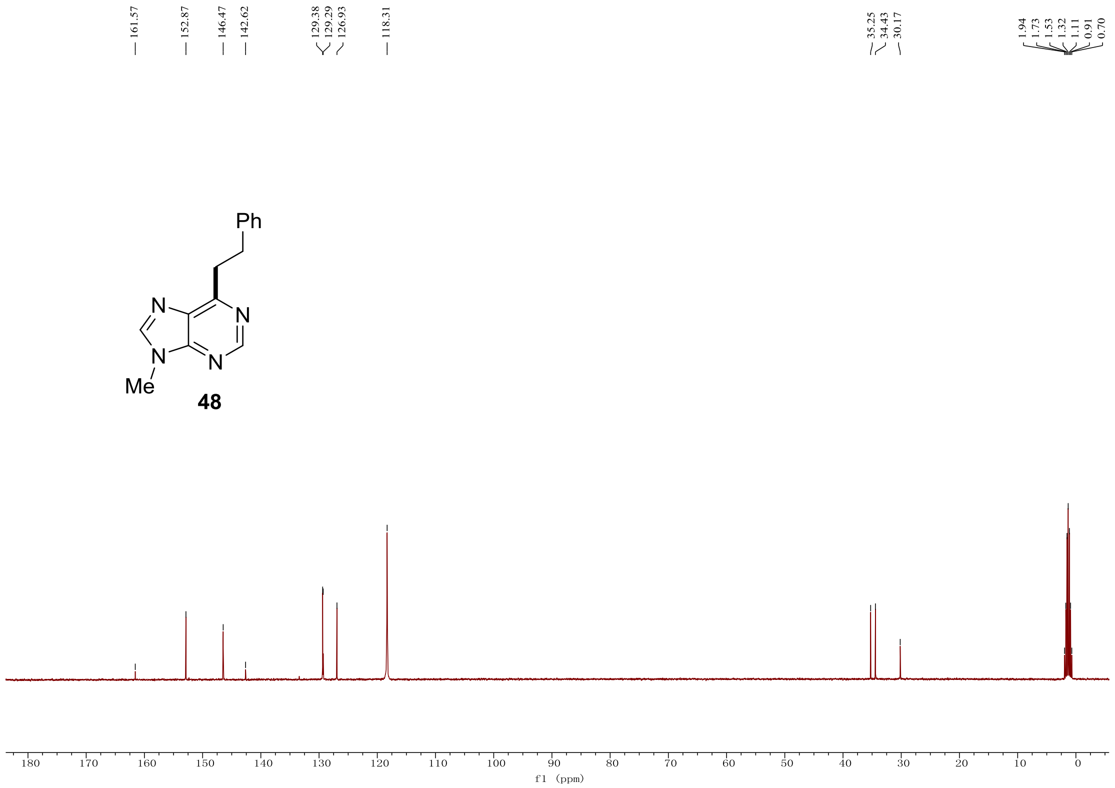

Supplementary Figure 245. ^{13}C NMR spectrum for 48.

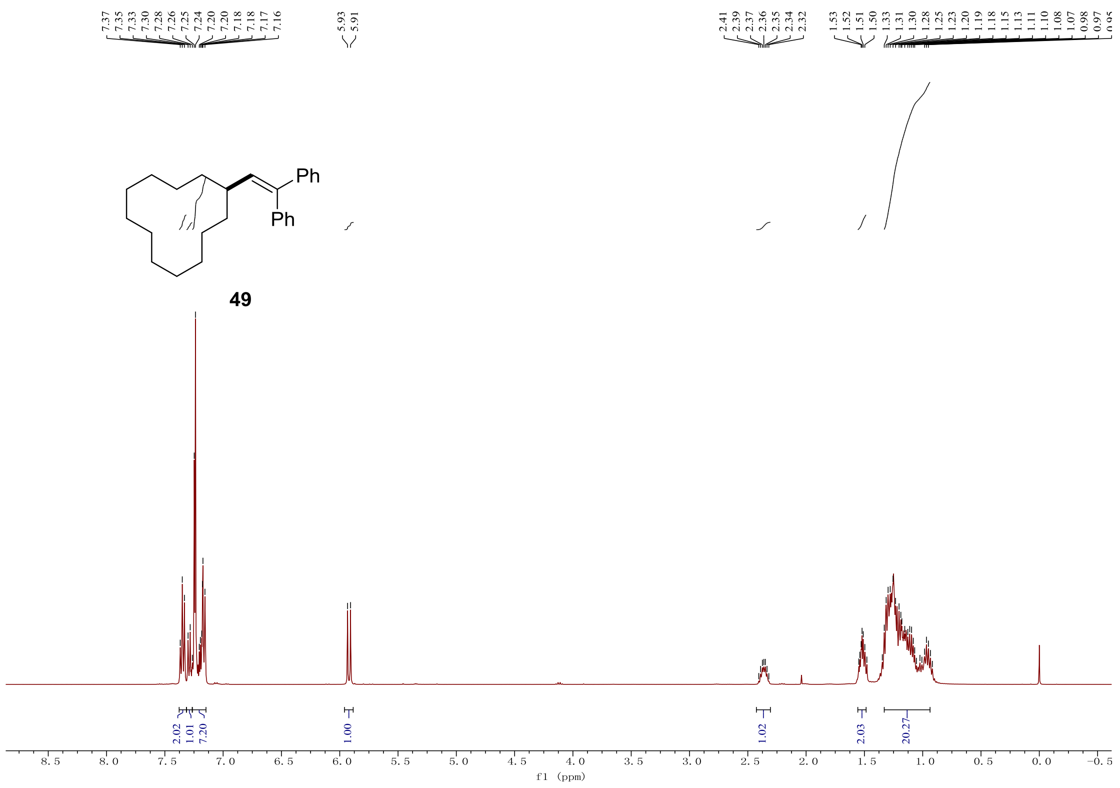

Supplementary Figure 246. ^{1}H NMR spectrum for 49.

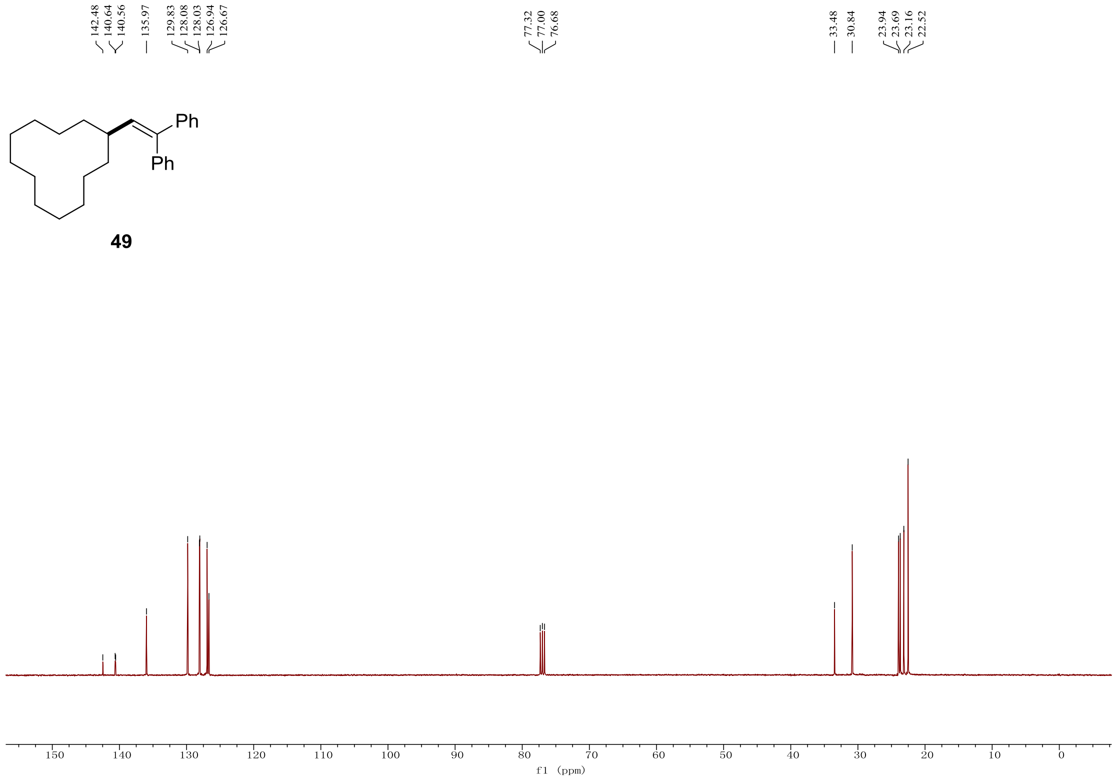

Supplementary Figure 247. ^{13}C NMR spectrum for 49.

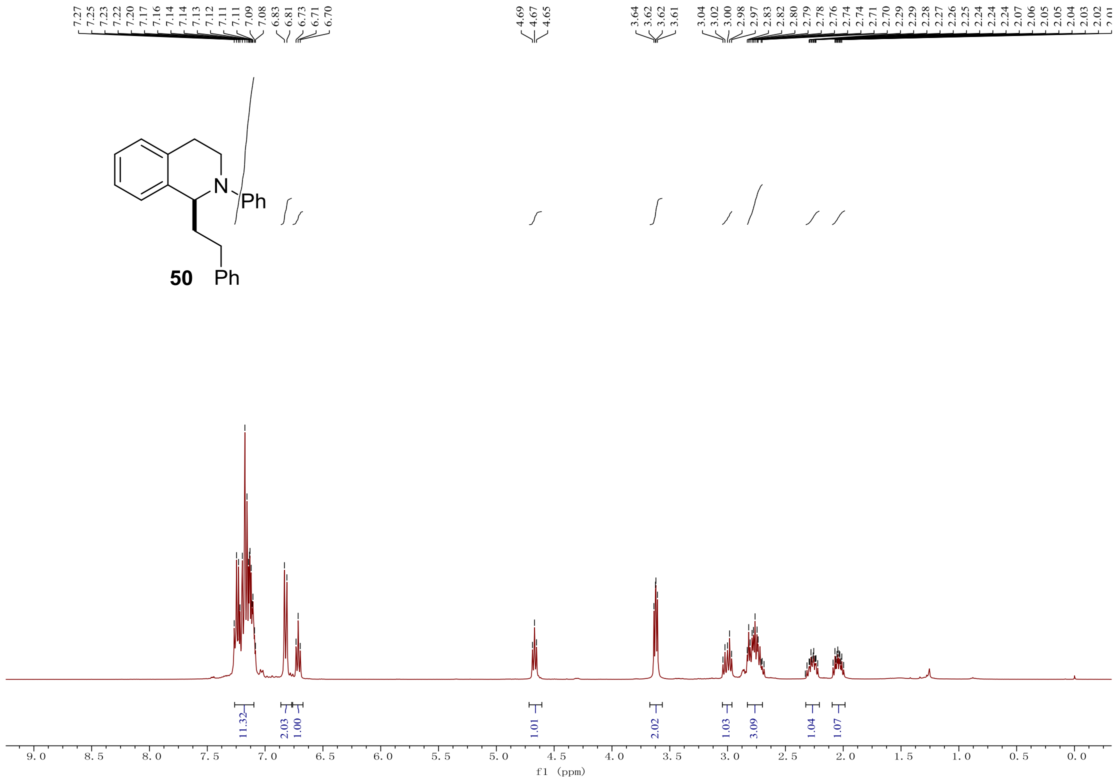

Supplementary Figure 248. ^{1}**H NMR spectrum for 50.**

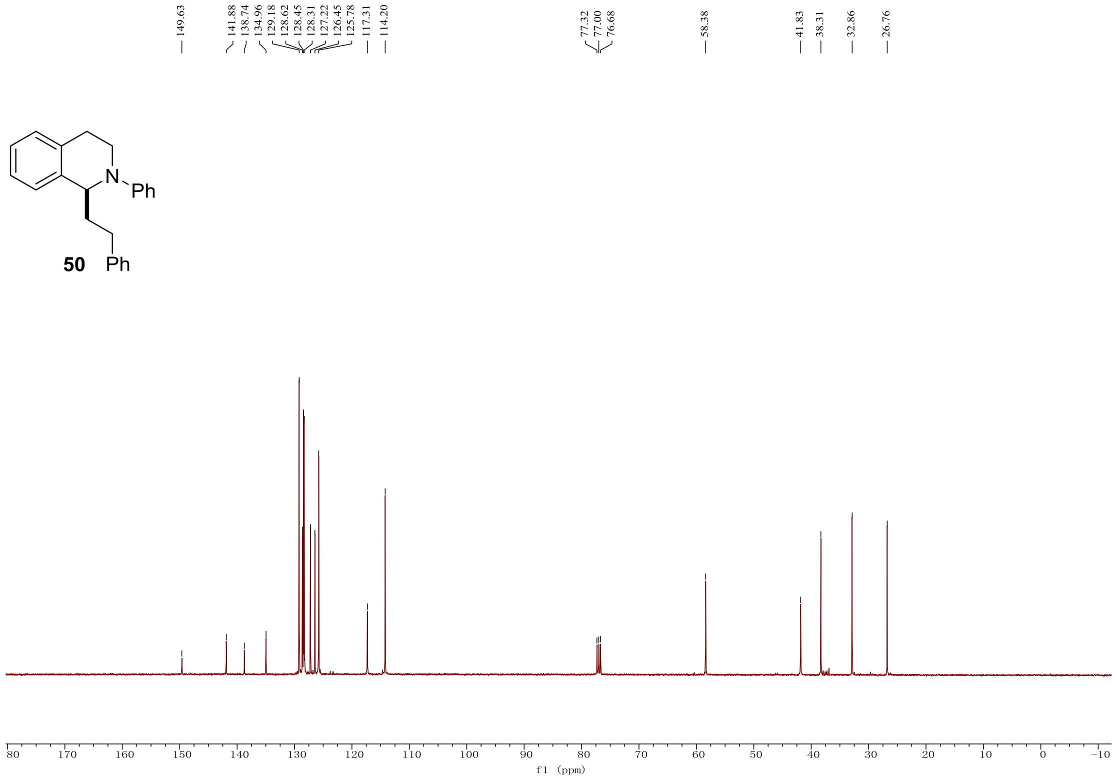

Supplementary Figure 249. ^{13}**C NMR spectrum for 50.**

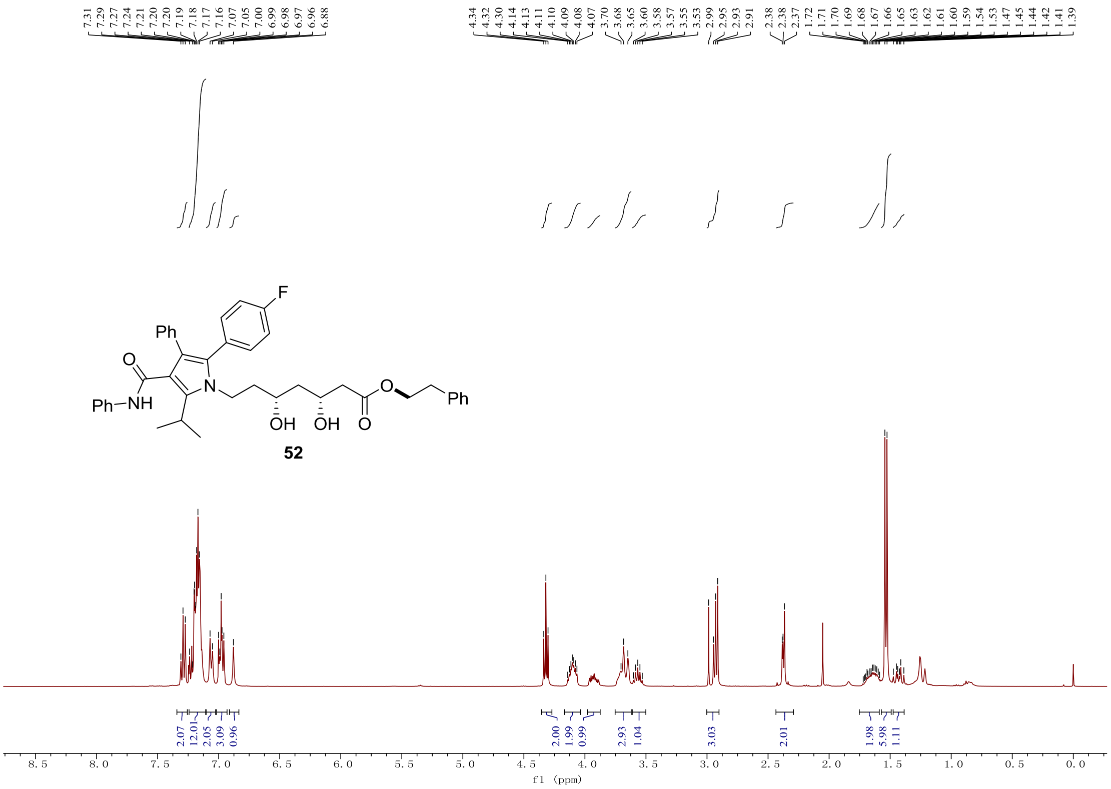

Supplementary Figure 250. ^{1}H NMR spectrum for 52.

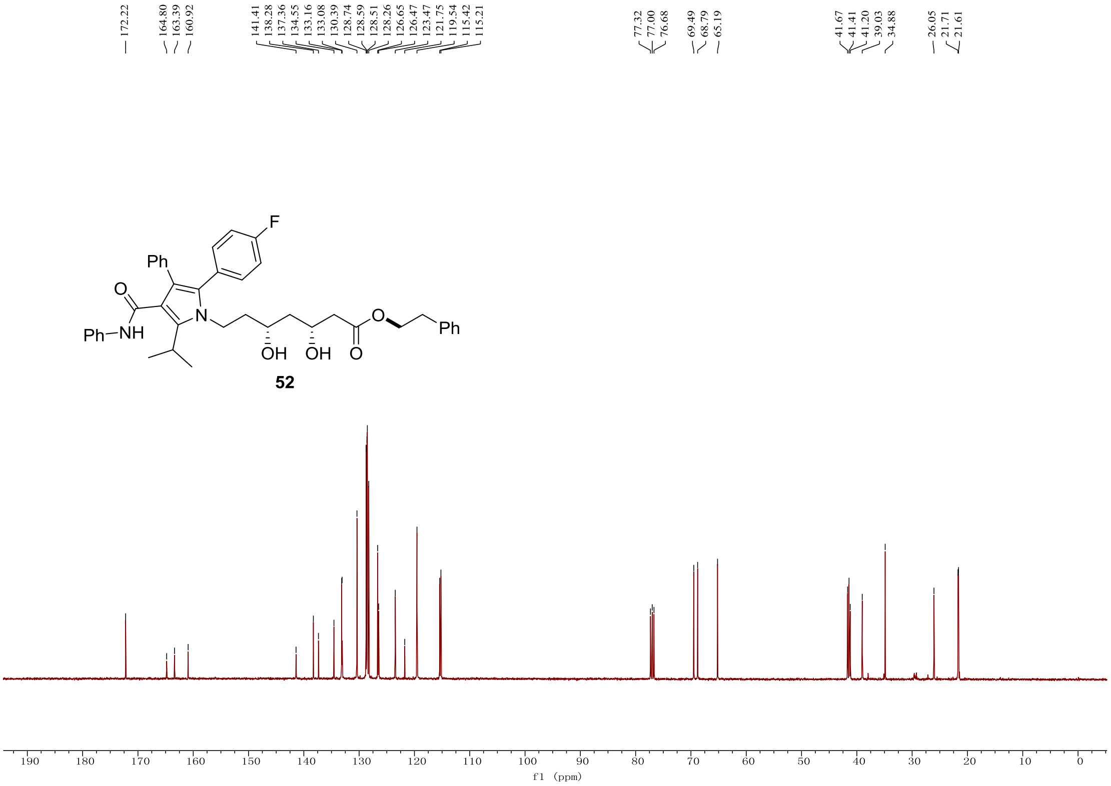

Supplementary Figure 251. ^{13}C NMR spectrum for 52.

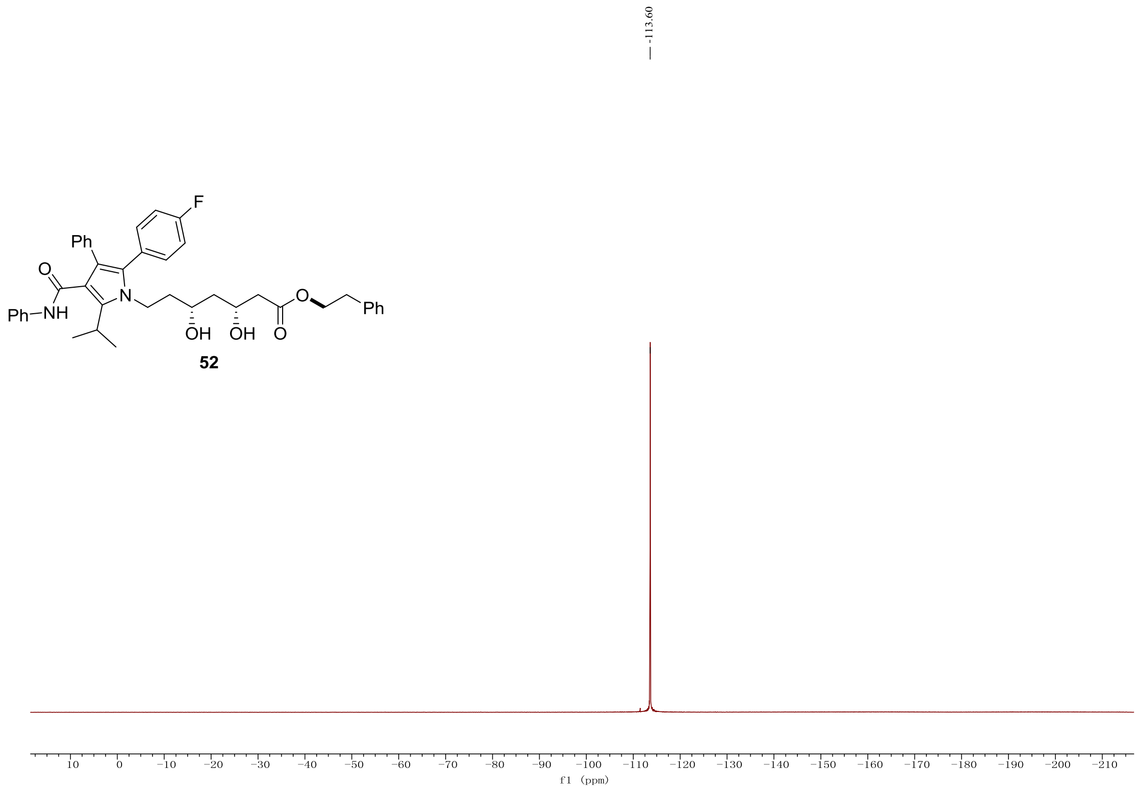

Supplementary Figure 252. ^{19}F NMR spectrum for 52.
