# Supplementary material for: Generation of non-stabilized alkyl radicals from thianthrenium salts for C–B and C–C bond formation
Source: Nat Commun. 2021 Jul 26;12:4526. doi: 10.1038/s41467-021-24716-2 (PMC8313578; doi:10.1038/s41467-021-24716-2)
Supplement: Supplementary file 2 — Description of Additional Supplementary Files [file 41467_2021_24716_MOESM2_ESM.pdf]

Description of Additional Supplementary Files

File Name: Supplementary Data 1
Description: Crystallographic Data
