# Supplementary material for: Generation of non-stabilized alkyl radicals from thianthrenium salts for C–B and C–C bond formation
Source: Nat Commun. 2021 Jul 26;12:4526. doi: 10.1038/s41467-021-24716-2 (PMC8313578; doi:10.1038/s41467-021-24716-2)

Supplementary Data 1

Crystallographic Data

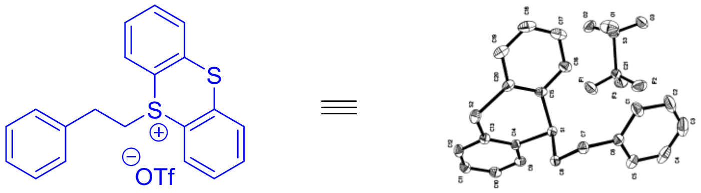

1a CCDC 2047564

Table 1. Crystal data and structure refinement for 20200921Lin_CC_1_SZZ_0m_a.

Identification code	20200921Lin_CC_1_SZZ_0m_a	
Empirical formula	C21 H17 F3 O3 S3	
Formula weight	470.53	
Temperature	193(2) K	
Wavelength	0.71073 Å	
Crystal system	Monoclinic	
Space group	$P2_1/n$	
Unit cell dimensions	a = 11.403(3) Å	α= 90 °.
	b = 12.129(4) Å	β= 107.652(13) °.
	c = 15.674(6) Å	γ = 90 °.
Volume	2065.7(12) $Å^3$	
Z	4	
Density (calculated)	1.513 Mg/m^3	
Absorption coefficient	0.407 mm^{-1}	
F(000)	968	
Crystal size	0.120 x 0.110 x 0.080 mm^3	
Theta range for data collection	2.163 to 28.290 °.	
Index ranges	-13<=h<=15, -16<=k<=16, -20<=l<=20	
Reflections collected	18224	
Independent reflections	4979 [R(int) = 0.0972]	
Completeness to theta = 25.242 °	97.9 %	
Refinement method	Full-matrix least-squares on F^2	
Data / restraints / parameters	4979 / 0 / 271	
Goodness-of-fit on F^2	1.011	
Final R indices [I>2sigma(I)]	R1 = 0.0633, wR2 = 0.1638	

R indices (all data)	R1 = 0.0964, wR2 = 0.1866
Extinction coefficient	n/a
Largest diff. peak and hole	0.458 and -0.467 e.Å^{-3}

Table 2. Atomic coordinates (x 10^4) and equivalent isotropic displacement parameters (Å^{2}x 10^3)
for 20200921Lin_CC_1_SZZ_0m_a. U(eq) is defined as one third of the trace of the orthogonalized U^{ij} tensor.

	x	y	z	U(eq)
C(1)	7488(3)	8720(3)	9444(2)	45(1)
C(2)	8743(4)	8837(4)	9619(3)	68(1)
C(3)	9502(4)	7951(5)	9813(3)	76(2)
C(4)	9018(4)	6911(4)	9802(2)	62(1)
C(5)	7746(3)	6769(3)	9630(2)	42(1)
C(6)	6984(3)	7680(2)	9466(2)	33(1)
C(7)	5620(3)	7589(2)	9351(2)	33(1)
C(8)	4948(3)	6634(2)	8780(2)	32(1)
C(9)	4503(3)	4678(2)	6877(2)	35(1)
C(10)	3656(3)	3913(3)	6429(2)	40(1)
C(11)	2413(3)	4170(3)	6146(2)	40(1)
C(12)	2004(3)	5192(3)	6310(2)	37(1)
C(13)	2841(3)	5988(2)	6778(2)	32(1)
C(14)	4081(3)	5708(2)	7054(2)	28(1)
C(15)	4667(3)	7933(2)	7230(2)	32(1)
C(16)	5541(3)	8691(3)	7155(2)	39(1)
C(17)	5153(4)	9740(3)	6847(2)	51(1)
C(18)	3928(4)	10012(3)	6614(2)	53(1)
C(19)	3051(4)	9249(3)	6654(2)	45(1)
C(20)	3407(3)	8185(2)	6962(2)	33(1)
C(21)	8540(3)	7219(2)	6421(2)	40(1)
F(1)	7466(2)	6769(2)	6369(2)	70(1)
F(2)	9169(3)	7347(2)	7282(2)	75(1)
F(3)	9148(2)	6473(2)	6096(2)	62(1)
O(1)	7749(3)	9194(2)	6271(2)	72(1)
O(2)	7714(3)	8186(2)	4905(2)	70(1)
O(3)	9605(2)	8843(2)	5905(2)	56(1)
S(1)	5221(1)	6636(1)	7685(1)	29(1)
S(2)	2247(1)	7250(1)	6997(1)	39(1)
S(3)	8368(1)	8511(1)	5806(1)	38(1)

Table 3. Bond lengths [Å] and angles [°] for 20200921Lin_CC_1_SZZ_0m_a.

C(1)-C(2)	1.380(5)
C(1)-C(6)	1.391(4)
C(1)-H(1)	0.9500
C(2)-C(3)	1.356(7)
C(2)-H(2)	0.9500
C(3)-C(4)	1.375(7)
C(3)-H(3)	0.9500
C(4)-C(5)	1.404(5)
C(4)-H(4)	0.9500
C(5)-C(6)	1.380(4)
C(5)-H(5)	0.9500
C(6)-C(7)	1.515(4)
C(7)-C(8)	1.521(4)
C(7)-H(7A)	0.9900
C(7)-H(7B)	0.9900
C(8)-S(1)	1.835(3)
C(8)-H(8A)	0.9900
C(8)-H(8B)	0.9900
C(9)-C(10)	1.370(4)
C(9)-C(14)	1.397(4)
C(9)-H(9)	0.9500
C(10)-C(11)	1.386(5)
C(10)-H(10)	0.9500
C(11)-C(12)	1.376(4)
C(11)-H(11)	0.9500
C(12)-C(13)	1.399(4)
C(12)-H(12)	0.9500
C(13)-C(14)	1.390(4)
C(13)-S(2)	1.749(3)
C(14)-S(1)	1.776(3)
C(15)-C(16)	1.387(4)
C(15)-C(20)	1.403(4)
C(15)-S(1)	1.763(3)
C(16)-C(17)	1.385(5)
C(16)-H(16)	0.9500
C(17)-C(18)	1.372(5)

C(17)-H(17)	0.9500
C(18)-C(19)	1.379(5)
C(18)-H(18)	0.9500
C(19)-C(20)	1.393(4)
C(19)-H(19)	0.9500
C(20)-S(2)	1.756(3)
C(21)-F(1)	1.321(4)
C(21)-F(2)	1.332(4)
C(21)-F(3)	1.331(4)
C(21)-S(3)	1.818(3)
O(1)-S(3)	1.423(3)
O(2)-S(3)	1.438(3)
O(3)-S(3)	1.430(3)

C(2)-C(1)-C(6)	120.0(4)
C(2)-C(1)-H(1)	120.0
C(6)-C(1)-H(1)	120.0
C(3)-C(2)-C(1)	121.1(4)
C(3)-C(2)-H(2)	119.5
C(1)-C(2)-H(2)	119.5
C(2)-C(3)-C(4)	119.9(4)
C(2)-C(3)-H(3)	120.1
C(4)-C(3)-H(3)	120.1
C(3)-C(4)-C(5)	120.1(4)
C(3)-C(4)-H(4)	119.9
C(5)-C(4)-H(4)	119.9
C(6)-C(5)-C(4)	119.6(4)
C(6)-C(5)-H(5)	120.2
C(4)-C(5)-H(5)	120.2
C(5)-C(6)-C(1)	119.3(3)
C(5)-C(6)-C(7)	121.9(3)
C(1)-C(6)-C(7)	118.7(3)
C(6)-C(7)-C(8)	116.2(2)
C(6)-C(7)-H(7A)	108.2
C(8)-C(7)-H(7A)	108.2
C(6)-C(7)-H(7B)	108.2
C(8)-C(7)-H(7B)	108.2
H(7A)-C(7)-H(7B)	107.4

C(7)-C(8)-S(1)	110.62(19)
C(7)-C(8)-H(8A)	109.5
S(1)-C(8)-H(8A)	109.5
C(7)-C(8)-H(8B)	109.5
S(1)-C(8)-H(8B)	109.5
H(8A)-C(8)-H(8B)	108.1
C(10)-C(9)-C(14)	118.4(3)
C(10)-C(9)-H(9)	120.8
C(14)-C(9)-H(9)	120.8
C(9)-C(10)-C(11)	120.3(3)
C(9)-C(10)-H(10)	119.8
C(11)-C(10)-H(10)	119.8
C(12)-C(11)-C(10)	121.0(3)
C(12)-C(11)-H(11)	119.5
C(10)-C(11)-H(11)	119.5
C(11)-C(12)-C(13)	120.2(3)
C(11)-C(12)-H(12)	119.9
C(13)-C(12)-H(12)	119.9
C(14)-C(13)-C(12)	117.6(3)
C(14)-C(13)-S(2)	124.8(2)
C(12)-C(13)-S(2)	117.6(2)
C(13)-C(14)-C(9)	122.4(3)
C(13)-C(14)-S(1)	121.5(2)
C(9)-C(14)-S(1)	116.1(2)
C(16)-C(15)-C(20)	121.8(3)
C(16)-C(15)-S(1)	116.5(2)
C(20)-C(15)-S(1)	121.7(2)
C(17)-C(16)-C(15)	118.5(3)
C(17)-C(16)-H(16)	120.8
C(15)-C(16)-H(16)	120.8
C(18)-C(17)-C(16)	120.3(3)
C(18)-C(17)-H(17)	119.8
C(16)-C(17)-H(17)	119.8
C(17)-C(18)-C(19)	121.3(3)
C(17)-C(18)-H(18)	119.3
C(19)-C(18)-H(18)	119.3
C(18)-C(19)-C(20)	119.9(3)
C(18)-C(19)-H(19)	120.0

C(20)-C(19)-H(19)	120.0
C(19)-C(20)-C(15)	118.0(3)
C(19)-C(20)-S(2)	117.8(3)
C(15)-C(20)-S(2)	124.2(2)
F(1)-C(21)-F(2)	108.3(3)
F(1)-C(21)-F(3)	106.0(3)
F(2)-C(21)-F(3)	106.5(3)
F(1)-C(21)-S(3)	112.0(2)
F(2)-C(21)-S(3)	112.0(2)
F(3)-C(21)-S(3)	111.6(2)
C(15)-S(1)-C(14)	103.22(14)
C(15)-S(1)-C(8)	103.73(13)
C(14)-S(1)-C(8)	102.51(13)
C(13)-S(2)-C(20)	102.66(14)
O(1)-S(3)-O(3)	113.98(18)
O(1)-S(3)-O(2)	118.2(2)
O(3)-S(3)-O(2)	112.60(19)
O(1)-S(3)-C(21)	102.78(16)
O(3)-S(3)-C(21)	103.95(16)
O(2)-S(3)-C(21)	103.02(16)

Symmetry transformations used to generate equivalent atoms:

Table 4. Anisotropic displacement parameters ($Å^2$x 10^3) for 20200921Lin_CC_1_SZZ_0m_a. The anisotropic displacement factor exponent takes the form: $-2\pi^2[h^2 a^{*2}U^{11} + ... + 2 h k a^* b^* U^{12}]$

	U^{11}	U^{22}	U^{33}	U^{23}	U^{13}	U^{12}
C(1)	49(2)	47(2)	40(2)	-10(2)	15(2)	-18(2)
C(2)	54(3)	97(3)	51(2)	-7(2)	14(2)	-42(3)
C(3)	42(2)	149(5)	36(2)	-2(3)	11(2)	-30(3)
C(4)	40(2)	115(4)	30(2)	6(2)	7(2)	24(2)
C(5)	38(2)	50(2)	34(2)	-1(2)	4(1)	7(1)
C(6)	36(2)	39(2)	22(1)	-4(1)	7(1)	-5(1)
C(7)	33(2)	36(2)	31(2)	-3(1)	10(1)	-1(1)
C(8)	30(2)	36(2)	27(2)	4(1)	5(1)	-7(1)
C(9)	39(2)	32(1)	35(2)	1(1)	13(1)	2(1)
C(10)	52(2)	33(2)	39(2)	-2(1)	19(2)	-4(1)
C(11)	44(2)	43(2)	32(2)	-7(1)	11(1)	-13(1)
C(12)	30(2)	53(2)	28(2)	-5(1)	6(1)	-6(1)
C(13)	32(2)	38(2)	26(1)	-2(1)	10(1)	1(1)
C(14)	29(2)	33(1)	22(1)	-1(1)	5(1)	-1(1)
C(15)	44(2)	28(1)	25(1)	1(1)	12(1)	0(1)
C(16)	51(2)	39(2)	29(2)	-1(1)	15(1)	-8(1)
C(17)	79(3)	35(2)	42(2)	1(1)	24(2)	-11(2)
C(18)	90(3)	31(2)	38(2)	4(1)	19(2)	11(2)
C(19)	58(2)	41(2)	33(2)	3(1)	11(2)	16(2)
C(20)	41(2)	34(1)	24(1)	-2(1)	9(1)	6(1)
C(21)	40(2)	29(1)	46(2)	-1(1)	7(1)	-3(1)
F(1)	50(1)	55(1)	106(2)	16(1)	26(1)	-12(1)
F(2)	114(2)	53(1)	43(1)	8(1)	0(1)	-15(1)
F(3)	62(2)	34(1)	85(2)	2(1)	19(1)	13(1)
O(1)	88(2)	42(1)	103(2)	5(2)	57(2)	22(1)
O(2)	83(2)	50(2)	50(2)	6(1)	-18(1)	-5(2)
O(3)	53(2)	49(1)	69(2)	3(1)	22(1)	-13(1)
S(1)	27(1)	31(1)	28(1)	1(1)	6(1)	-1(1)
S(2)	32(1)	44(1)	41(1)	-4(1)	10(1)	7(1)
S(3)	44(1)	28(1)	42(1)	0(1)	11(1)	3(1)

Table 5. Hydrogen coordinates (x 10^4) and isotropic displacement parameters ($Å^2$x 10^3) for 20200921Lin_CC_1_SZZ_0m_a.

	x	y	z	U(eq)
H(1)	6968	9350	9309	54
H(2)	9080	9550	9603	81
H(3)	10367	8048	9955	91
H(4)	9546	6286	9912	75
H(5)	7411	6052	9627	51
H(7A)	5220	8284	9083	40
H(7B)	5512	7522	9952	40
H(8A)	4055	6698	8695	38
H(8B)	5239	5929	9090	38
H(9)	5358	4512	7062	42
H(10)	3922	3202	6312	48
H(11)	1836	3632	5835	48
H(12)	1150	5358	6104	45
H(16)	6386	8495	7311	47
H(17)	5738	10274	6797	61
H(18)	3680	10742	6421	63
H(19)	2205	9447	6473	54

checkCIF/PLATON report

Structure factors have been supplied for datablock(s) 20200921Lin_CC_1_SZZ_0m_a

THIS REPORT IS FOR GUIDANCE ONLY. IF USED AS PART OF A REVIEW PROCEDURE FOR PUBLICATION, IT SHOULD NOT REPLACE THE EXPERTISE OF AN EXPERIENCED CRYSTALLOGRAPHIC REFEREE.

No syntax errors found. CIF dictionary Interpreting this report

Datablock: 20200921Lin_CC_1_SZZ_0m_a

```
Bond precision:  C-C = 0.0053 A          Wavelength=0.71073

Cell:            a=11.403(3)    b=12.129(4)          c=15.674(6)
                 alpha=90       beta=107.652(13)     gamma=90
Temperature:     193 K

                Calculated                 Reported
Volume          2065.8(12)                 2065.7(12)
Space group     P 21/n                     P 21/n
Hall group      -P 2yn                     -P 2yn
Moiety formula  C20 H17 S2, C F3 O3 S      ?
Sum formula     C21 H17 F3 O3 S3           C21 H17 F3 O3 S3
Mr              470.53                     470.53
Dx,g cm-3       1.513                      1.513
Z               4                          4
Mu (mm-1)       0.407                      0.407
F000            968.0                      968.0
F000'           970.02
h,k,lmax        15,16,20                   15,16,20
Nref            5122                       4979
Tmin,Tmax       0.952,0.968
Tmin'           0.952

Correction method= Not given

Data completeness= 0.972          Theta(max)= 28.290

R(reflections)= 0.0633( 3391)     wR2(reflections)= 0.1866( 4979)

S = 1.011                  Npar= 271
```

The following ALERTS were generated. Each ALERT has the format
test-name_ALERT_alert-type_alert-level.
Click on the hyperlinks for more details of the test.

Alert level C

```
PLAT029_ALERT_3_C _diffrn_measured_fraction_theta_full value Low .      0.979 Why?
PLAT052_ALERT_1_C Info on Absorption Correction Method   Not Given     Please Do !
PLAT244_ALERT_4_C Low    'Solvent' Ueq as Compared to Neighbors of        S3 Check
PLAT340_ALERT_3_C Low Bond Precision on  C-C Bonds ..............     0.0053 Ang.
PLAT906_ALERT_3_C Large K Value in the Analysis of Variance ......      9.348 Check
PLAT911_ALERT_3_C Missing FCF Refl Between Thmin & STh/L=    0.600        77 Report
PLAT913_ALERT_3_C Missing # of Very Strong Reflections in FCF ....         5 Note
```

Alert level G

```
PLAT244_ALERT_4_G Low    'Solvent' Ueq as Compared to Neighbors of       C21 Check
PLAT883_ALERT_1_G No Info/Value for _atom_sites_solution_primary .     Please Do !
PLAT910_ALERT_3_G Missing # of FCF Reflection(s) Below Theta(Min).         1 Note
PLAT912_ALERT_4_G Missing # of FCF Reflections Above STh/L=  0.600        66 Note
PLAT941_ALERT_3_G Average HKL Measurement Multiplicity ...........       3.7 Low
PLAT978_ALERT_2_G Number C-C Bonds with Positive Residual Density.         0 Info
```

0 **ALERT level A** = Most likely a serious problem - resolve or explain
0 **ALERT level B** = A potentially serious problem, consider carefully
7 **ALERT level C** = Check. Ensure it is not caused by an omission or oversight
6 **ALERT level G** = General information/check it is not something unexpected

2 ALERT type 1 CIF construction/syntax error, inconsistent or missing data
1 ALERT type 2 Indicator that the structure model may be wrong or deficient
7 ALERT type 3 Indicator that the structure quality may be low
3 ALERT type 4 Improvement, methodology, query or suggestion
0 ALERT type 5 Informative message, check

It is advisable to attempt to resolve as many as possible of the alerts in all categories. Often the minor alerts point to easily fixed oversights, errors and omissions in your CIF or refinement strategy, so attention to these fine details can be worthwhile. In order to resolve some of the more serious problems it may be necessary to carry out additional measurements or structure refinements. However, the purpose of your study may justify the reported deviations and the more serious of these should normally be commented upon in the discussion or experimental section of a paper or in the "special_details" fields of the CIF. checkCIF was carefully designed to identify outliers and unusual parameters, but every test has its limitations and alerts that are not important in a particular case may appear. Conversely, the absence of alerts does not guarantee there are no aspects of the results needing attention. It is up to the individual to critically assess their own results and, if necessary, seek expert advice.

Publication of your CIF in IUCr journals

A basic structural check has been run on your CIF. These basic checks will be run on all CIFs submitted for publication in IUCr journals (*Acta Crystallographica*, *Journal of Applied Crystallography*, *Journal of Synchrotron Radiation*); however, if you intend to submit to *Acta Crystallographica Section C* or *E* or *IUCrData*, you should make sure that full publication checks are run on the final version of your CIF prior to submission.

Publication of your CIF in other journals

Please refer to the *Notes for Authors* of the relevant journal for any special instructions relating to CIF submission.

PLATON version of 03/06/2021; check.def file version of 02/06/2021

Datablock 20200921Lin_CC_1_SZZ_0m_a - ellipsoid plot

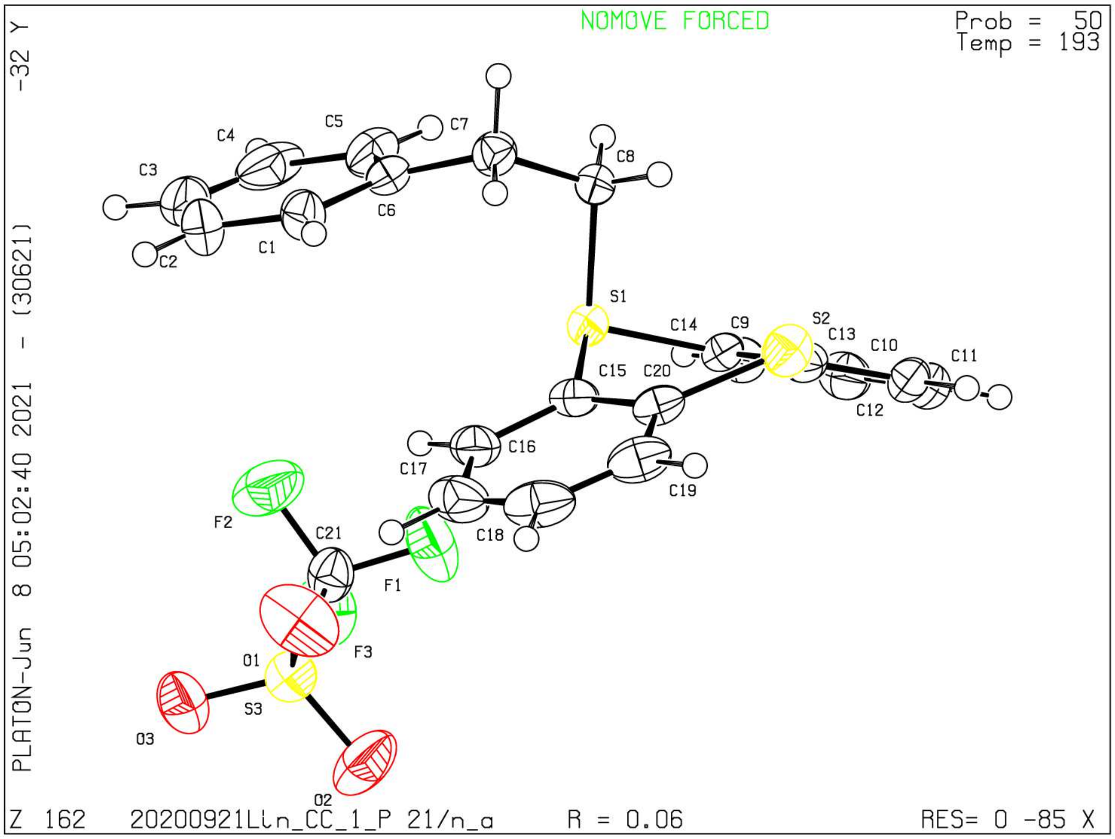

Supplement: Supplementary file 3 — Supplementary Data 1 [file 41467_2021_24716_MOESM3_ESM.pdf]
